# Photochemically Mediated Ring Expansion of Indoles and Pyrroles with Chlorodiazirines: Synthetic Methodology and Thermal Hazard Assessment

**DOI:** 10.1002/anie.202305081

**Published:** 2023-06-27

**Authors:** Ben W. Joynson, Graham R. Cumming, Liam T. Ball

**Affiliations:** ^1^ School of Chemistry University of Nottingham Nottingham NG7 2RD UK; ^2^ Centro de Investigación Lilly S. A. Avda. de la Industria 30, Alcobendas Madrid 28108 Spain

**Keywords:** Diazirines, Differential Scanning Calorimetry, Photochemistry, Ring Expansion, Skeletal Editing

## Abstract

We demonstrate that arylchlorodiazirines serve as photo‐activated halocarbene precursors for the selective one‐carbon ring expansion of *N*‐substituted pyrroles and indoles to the corresponding pyridinium and quinolinium salts. Preliminary investigations indicate that the same strategy also enables the conversion of *N*‐substituted pyrazoles to pyrimidinium salts. The *N*‐substituent of the substrate plays an essential role in: (1) increasing substrate scope by preventing product degradation, (2) enhancing yields by suppressing co‐product inhibition, and (3) activating the azinium products towards subsequent synthetic manipulations. This latter point is illustrated by subjecting the quinolinium salts to four complementary partial reductions, which provide concise access to ring‐expanded products with different degrees of increased C(sp^3^) character. Thermal analysis of the diazirines by differential scanning calorimetry (DSC) provides detailed insight into their energetic properties, and highlights the safety benefits of photolyzing—rather than thermolyzing—these reagents.

## Graphical abstract

We demonstrate that pyridinium and quinolinium salts can be accessed by the insertion of a single C‐atom from an arylchlorodiazirine into the parent *N*‐alkyl pyrrole or indole. The azinium products of ring expansion are activated towards oxygenation and hydrogenation, expediting further structural diversification. Safer methods for the use of diazirines as carbene precursors have been developed with insight from differential scanning calorimetry.

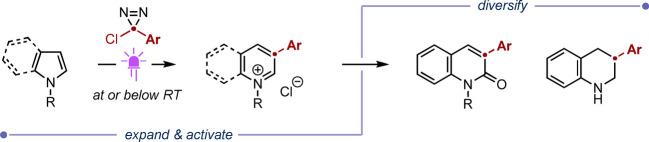

## Introduction

Methods for the precise modification of complex molecules are indispensable in contemporary organic synthesis. Not only do they enable the rapid diversification of existing compound libraries[^1^, ^2^, ^3^, ^4^] and the development of efficient routes to new targets,[^5^, ^6^] but they also provide a key testing‐ground for novel reactivity concepts.[^7^, ^8^] Within this arena, strategies that target the core skeleton of the substrate—rather than its periphery—are of particular value.[^9^, ^10^, ^11^, ^12^, ^13^] For example, interconversion between five‐ and six‐membered heteroarenes by the selective insertion or deletion of a single atom leads to stark changes in reactivity, retrosynthetic accessibility, and physicochemical properties.^[14]^

**Scheme 1:**
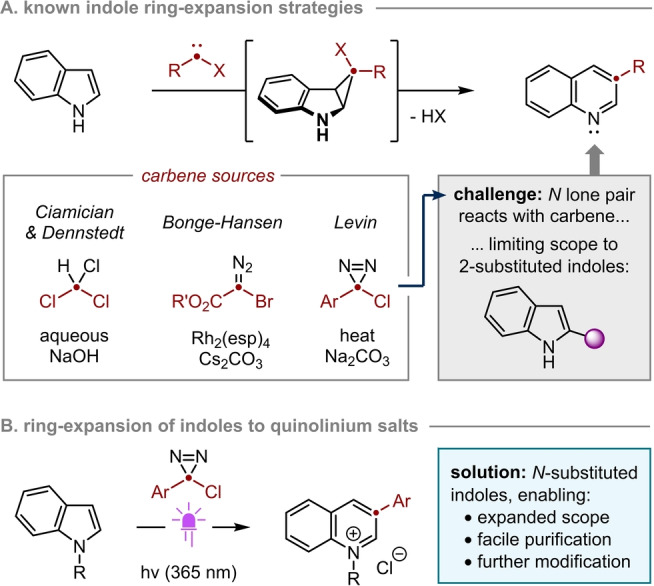
Carbon atom insertion into indoles via halocyclopropanation/fragmentation. a) evolution of halocarbene precursors; b) ring expansion of *N*‐substituted indoles as a direct route to azinium salts.

A particularly apposite case is the one‐carbon ring expansion of pyrroles and indoles. As established by Ciamician and Dennstedt,[^15^, ^16^] this “skeletal edit” can be achieved in practice via a halocyclopropanation/fragmentation sequence (Scheme 1A). However, the original incarnation of this transformation typically suffers from modest yields due to competing Reimer‐Tiemann formylation,^[17]^ is limited to insertion of a carbon‐halogen moiety (i.e., R=halogen in Scheme 1A), and requires strongly basic conditions that are rarely compatible with complex substrates.^[18]^

In 2015, Bonge‐Hansen and co‐workers demonstrated that the ring expansion of indoles can be achieved under much milder conditions—and therefore in higher yield and with greater functional group compatibility—by using halodiazoesters as carbenoid precursors.[^19^, ^20^] Unfortunately, the poor thermal stability^[21]^ of these reagents limits practicality, poses potential safety risks and ultimately requires that the inserted substituent be electron‐withdrawing (R=ester in Scheme 1A).^[22]^

The most significant advance in the field was made by Levin and co‐workers,^[23]^ who demonstrated the utility of arylchlorodiazirines for the ring expansion of both pyrroles and indoles (Scheme 1A). The mild thermolytic conditions employed for carbene generation conferred compatibility with synthetically‐valuable functionality and complex molecular architectures, while the higher stability^[24]^ of the diazirinyl moiety—relative to its diazo isomer—enabled installation of aryl and heteroaryl rings for the first time.

However, despite its broad utility, Levin's study revealed two substantial chemoselectivity challenges relating to the intermediate carbene.^[23]^ First, chloride generated as a co‐product of the ring expansion was found to react with the carbene, resulting in its non‐productive consumption. Second, the ring‐expanded quinoline was proposed to intercept the carbene,^[25]^ thereby consuming both the insertive agent and the desired product. While poisoning due to chloride was mitigated by addition of base, the latter challenge could not be addressed in a general sense. As such, high yields were obtained only for indoles or pyrroles bearing substituents adjacent to the *N*‐atom, presumably due to steric shielding of the basic nitrogen in the product (the potential for N−H insertion was not discussed, but presumably would also be suppressed by the 2‐substituent^[26]^). Yields were significantly lower for substrates lacking steric shielding around nitrogen, with just 22 % average yield obtained for 2‐unsubstituted indoles (twelve examples, 0 %–41 %) and 17 % average yield obtained for 2‐ and 2,6‐unsubstituted pyrroles (three examples, 0 %–34 %). This limitation on the scope of an otherwise extremely powerful methodology is especially pertinent given the prevalence of 2‐unsubstituted indoles in biologically relevant compounds (e.g., tryptophan and its derivatives).

Here we report the development of a broadly applicable method for the one‐carbon ring expansion of indoles and pyrroles that—crucially—affords high yields without the requirement for a 2‐substituted substrate (Scheme 1B). This significant extension in scope is made possible by the introduction of an *N*‐substituent, which acts as a cleavable protecting group for the azine nitrogen. Spontaneous precipitation of the product as a quinolinium/pyridinium salt not only enables chromatography‐free isolation, but also removes chloride poison from solution during the reaction. In addition, *N*‐substitution also serves to activate the heteroaromatic core of the product towards further functionalization, thereby facilitating rapid access to products with increased sp^3^ character. Finally, by generating the requisite carbene via photolysis rather than thermolysis, reactions can be performed at or below ambient temperature. In this way we avoid the risk associated with heating arylchlorodiazirines, the thermal properties of which we have quantified by differential scanning calorimetry.

## Results and Discussion

### Reaction Development

We initiated our studies with the photolysis[^27^, ^28^] of 3‐chloro‐3‐phenyl‐3*H*‐diazirine **1** in the presence of *N*‐benzyl 5‐fluoroindole **2** (Table 1; see Supporting Information for details). Consistent with our hypothesis, precipitation was observed within *ca* one hour of irradiation at 365 nm in non‐polar TBME (entry 1; *E*_T_^N^=0.124^[29]^). The reaction yield was improved in CH_2_Cl_2_ (entry 2; *E*_T_^N^=0.309^[29]^), but reduced in more polar MeCN (entry 3; *E*_T_^N^=0.460^[29]^). Only traces of product **3** were observed at room temperature in the absence of irradiation (entry 4), whereas thermolytic carbene generation led to moderate or very low yields (entries 5 and 6).

**Table 1:** Selected optimization and control reactions.

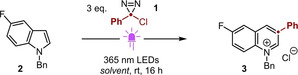			
Entry	Solvent	Deviation from Above	Yield **3** [%]^[a]^
1	TBME	None	49
2	CH_2_Cl_2_	None	82 (82)
3	MeCN	None	20
4	CH_2_Cl_2_	Dark	4
5	CH_2_Cl_2_	Dark; temperature=50 °C	64
6	MeCN	Dark; temperature=50 °C	16
7	CH_2_Cl_2_	3.0 equiv [Bu_4_N]Cl added	0
8	CH_2_Cl_2_	5‐Fluoroindole replaces **2**	<10
9	CH_2_Cl_2_	3.0 equiv quinoline added	7

[a] Yields determined by ^19^F NMR spectroscopic analysis vs internal standard; values in parentheses refer to isolated material.

The importance of removing chloride from solution—and the efficacy of precipitation as a means of achieving this—was evidenced by suppression of the reaction when an exogenous source of soluble chloride was added (entry 7). This sensitivity to chloride is congruent with the low yields obtained using MeCN (entries 3 and 6), a better solvent for the quinolinium salt. The beneficial role of the *N*‐benzyl substituent as a protecting group is reflected in the low yield obtained for the parent N−H indole (entry 8), consistent with earlier observations.^[23]^ Adding quinoline also suppressed yield (entry 9), confirming that basic nitrogen remains a poison under our conditions and that the low yield in entry 8 is not solely due to inherent changes in substate reactivity.

### Reaction Scope

**Scheme 2:**
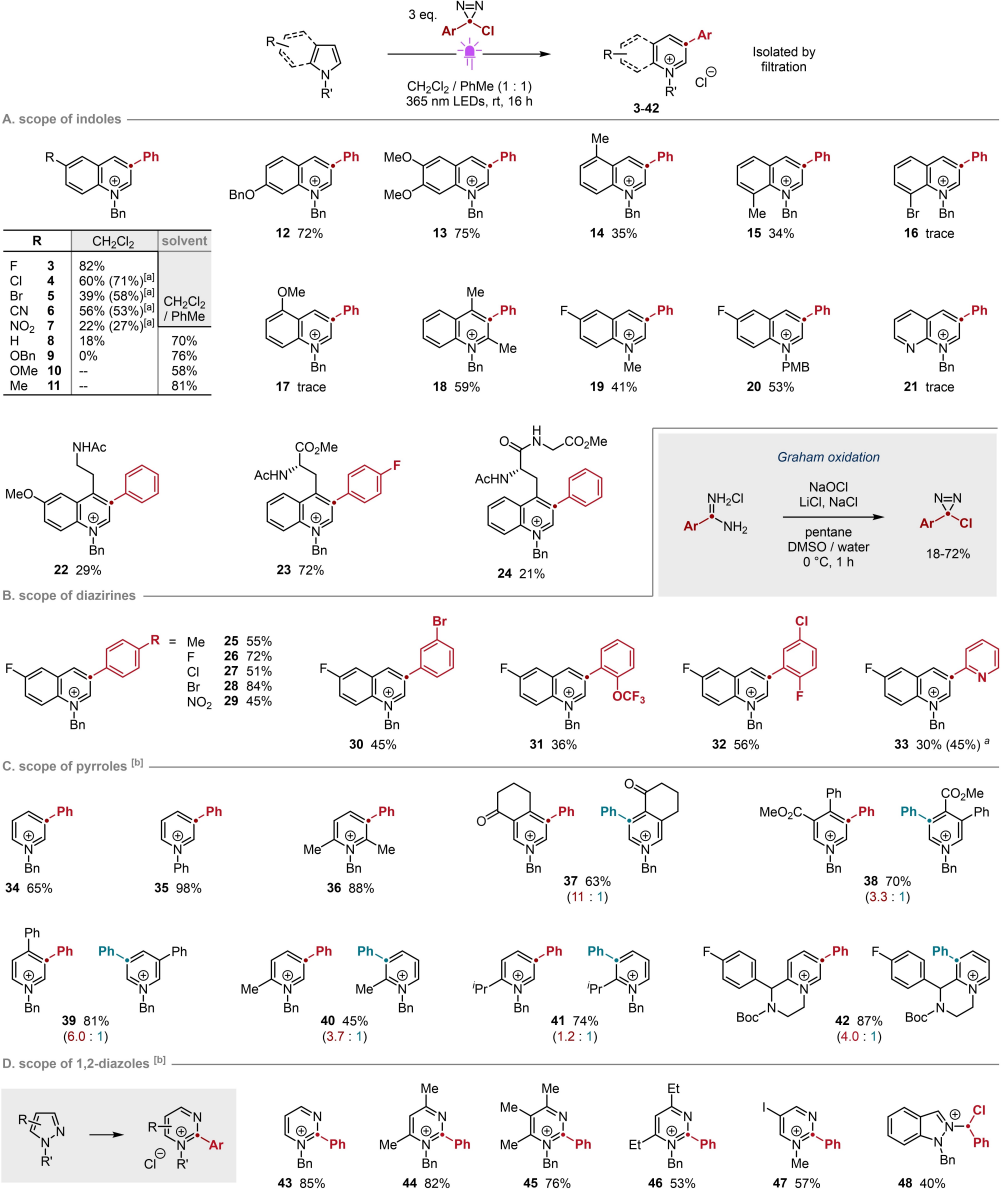
Scope of the one‐carbon ring expansion of azoles and diazoles. a) application to indoles; b) investigation of diazirine partners; c) application to pyrroles; d) application to pyrazoles. Yields refer to isolated material.^[a]^ Using 5 equivalents of diazirine.^[b]^ Solvent is TBME; regioisomeric ratio determined by NMR spectroscopic analysis following precipitation.

Having confirmed the dual role of the *N*‐benzyl substituent as a promoter of precipitation and a mask for the reactive lone pair of the product, we investigated substrate scope. The conditions detailed in Table 1, entry 2 proved applicable to a range of *N*‐benzyl indoles bearing electron‐withdrawing substituents at the 5‐position (**3**–**7**, Scheme 2A). As observed during reaction development, the corresponding quinolinium products precipitated during the reaction and were all isolable by simple filtration. Crucially, good to excellent yields were obtained without the need for a sterically‐shielding substituent at the 2‐position, thereby extending the substrate scope significantly beyond that established in previous diazirine‐mediated indole expansions.^[23]^ Furthermore, the compatibility of our methodology with indoles bearing electron‐withdrawing substituents stands in contrast to other Ciamician‐Dennstedt‐type ring expansions. Indeed, the use of nitro‐ and cyano‐substituted indoles has not been reported previously, irrespective of the insertive agent, whereas the four previous examples using bromo‐ and chloro‐substituted indoles all yield <25 %.[^23^, ^30^, ^31^, ^32^] It is therefore notable that the yields of **4** and **5**—bearing synthetically‐valuable halides—can be increased even further by using additional diazirine.

For electron‐neutral or ‐rich indoles (**8**, **9**), yields were improved in a binary solvent mixture of CH_2_Cl_2_ and PhMe (see Supporting Information for further details). The improvement relative to CH_2_Cl_2_ alone is ascribed to the lower solubility of the quinolinium salts in this mixed medium, driving the precipitation that is required to remove the chloride salt during the ring expansion process. Under these modified conditions, which were adopted for the remaining examples in Schemes 2A and B, ring expansion proceeded smoothly for a range of electron‐rich indoles (**9**–**13**). The resulting broad scope with respect to 2,3‐unsubstituted indoles is especially notable, given that this motif is not well‐tolerated by previously reported ring expansion methodology.^[23]^

Consistent with earlier observations,[^23^, ^30^, ^33^] substitution at the 4‐ or 7‐ positions of the indole is not well tolerated (**14**–**17**), with modest yields obtained only for methyl substituents in these positions (**14**, **15**). In contrast, substitution across the 2‐ and 3‐positions affords the corresponding quinolinium salt in good isolated yield (**18**). The ring expansion also occurs smoothly for *N*‐substituents other than benzyl (**19**, **20**); the compatibility of an *N*‐methyl indole is particularly valuable, because this motif is common to numerous ergot alkaloids^[34]^ and approved drugs such as zafirlukast^[35]^ and osimertinib.^[36]^

The potential for application of our methodology in complex molecule synthesis is illustrated by the ring expansion of protected melatonin (**22**) and tryptophan (**23**), and a tryptophan‐containing dipeptide (**24**). Notably, the synthesis of **23** and **24** proceeds with no loss in stereochemical integrity.

To explore the scope with respect to the inserted carbynyl fragment, a library of diazirines was prepared conveniently via Graham oxidation^[37]^ of the corresponding amidinium chloride (Scheme 2B, inset). Given the electronic trend observed in the stability of the diazirines (see below)—and hence in the safety of their synthesis and manipulation—we did not explore diazirines bearing strongly electron donating groups. Apropos of this, our methodology is applicable to moderately electron‐rich through very electron‐poor diazirines, with substitution tolerated at the *para* (**25**–**29**), *meta* (**30**, **32**) and *ortho* (**31**, **32**) positions of the aryl moiety. Use of a 2‐pyridyl diazirine provides access to the (orthogonally‐protected) biheteroaryl **33**, and represents an alternative approach to 2‐pyridyl arenes that are often challenging to prepare via Suzuki–Miyaura cross‐coupling.[^38^, ^39^]

By using TBME as solvent to facilitate precipitation, the methodology proved equally applicable to the ring expansion of pyrroles (Scheme 2C). As for the indole expansion, there is no requirement for 2,6‐disubstitution and generally good yields are achieved for pyrroles bearing no (**34**, **35**, **37**–**39**) or one (**40**–**42**) substituent adjacent to the nitrogen. Such substrates are not compatible with the previously reported diazirine‐mediated azole expansion.^[23]^ Regioisomeric mixtures are formed for non‐symmetrical pyrroles, with carbon atom insertion generally occurring at the more electron‐rich and less hindered site, consistent with earlier reports on the expansion of N−H pyrroles with chlorocarbenes.^[23]^

Photolytically‐generated chlorocarbenes can also be used to effect C‐atom insertion into the N−N bond of *N*‐alkyl pyrazoles (Scheme 2D). The corresponding pyrimidinium salts are formed in high yield for a range of (poly)substituted substrates (**43**–**47**), and are again isolated by simple filtration. Although superficially similar to the reactions of indoles and pyrroles, this ring expansion—first reported for N−H diazoles under thermolytic conditions^[40]^—is proposed to proceed via a mechanistically distinct pathway that involves initial *N* ^2^ quaternization of the pyrazole.^[40]^ Evidence that the same mechanism operates under our conditions is provided by the observation that *N* ^1^‐benzyl indazole reacts to give *N* ^2^‐chloroalkyl salt **48**, presumably via adventitious protonation of the key ylide intermediate. Other diazoles and triazoles either afforded analogous salts or proved unreactive (see Supporting Information).

**Scheme 3:**
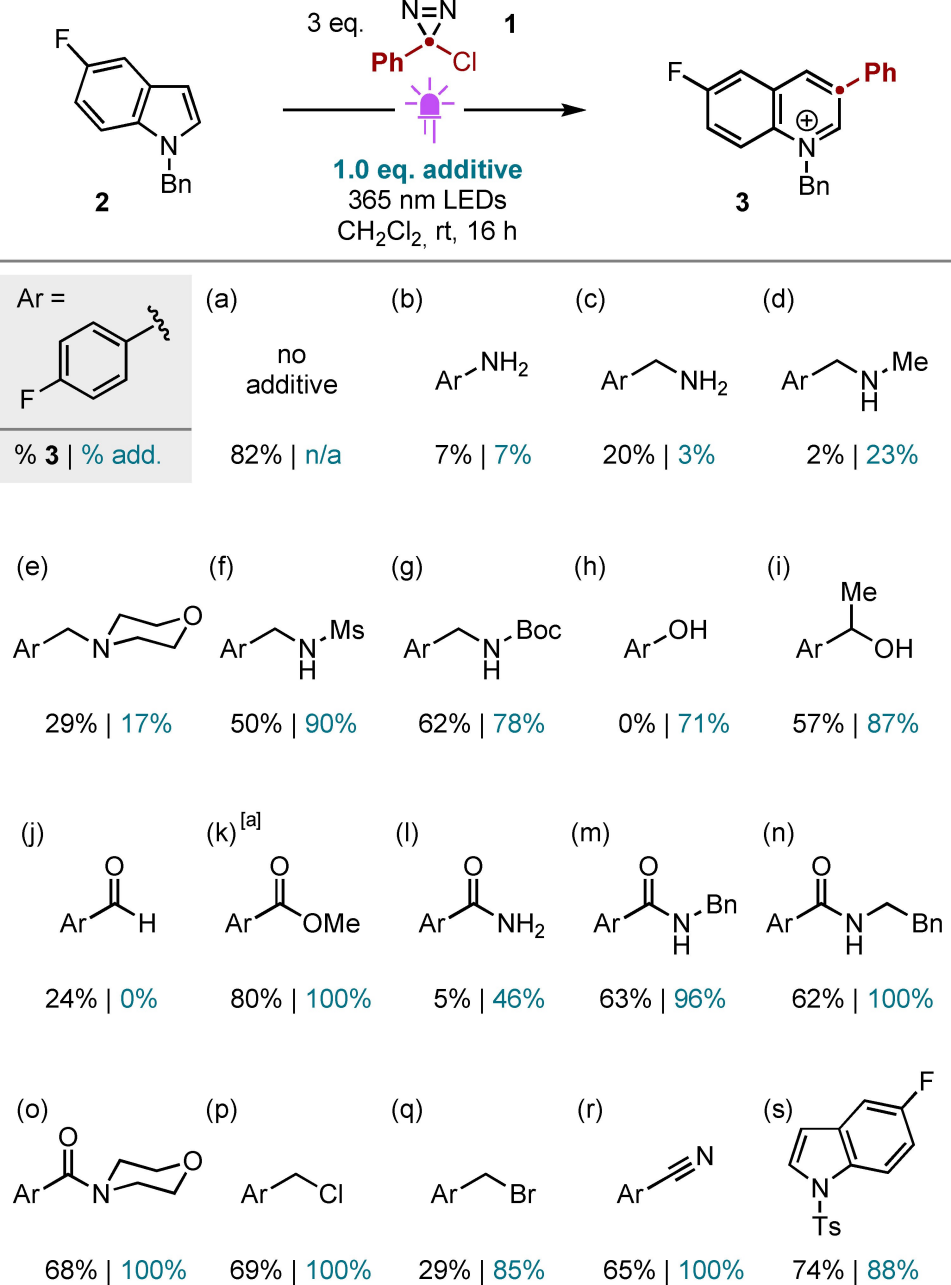
Robustness screen of indole ring expansion. Yields determined by ^19^F NMR spectroscopic analysis vs internal standard.^[a]^ Ar=4‐F_3_C‐C_6_H_4_.

A further assessment of functional group compatibility was made using a Glorius‐type robustness screen (Scheme 3).[^41^, ^42^] Consistent with the known reactivity of (halo)carbenes,[^43^, ^44^, ^45^, ^46^, ^47^] basic nitrogen both suppressed the desired transformation and was itself decomposed under the reaction conditions (Scheme 3, compare entry a to entries b–e). In contrast, nitrogen protected as a sulfonamide (f), carbamate (g), or secondary/tertiary amide (m‐o) was tolerated, consistent with the results presented in Scheme 2. While a phenol completely suppressed ring expansion, the additive was recovered largely unaffected (h); secondary alcohols (i), benzyl halides (p, q) and nitriles (r) proved innocent and were unaffected by the reaction conditions. Of note, an *N*‐tosyl indole proved similarly innocent (s), allowing for chemoselective modification of the more electron‐rich indole moiety. This latter result contrasts the observation that carbenes generated via the photolysis of aryldiazoacetates[^48^, ^49^] readily cyclopropanate indoles and pyrroles bearing *N*‐tosyl, ‐carbamoyl or ‐acyl substituents,[^50^, ^51^, ^52^] yet react with *N*‐alkyl indoles and pyrroles via C−H insertion.[^50^, ^53^]

### Structural Diversification of Azinium Salts

**Scheme 4:**
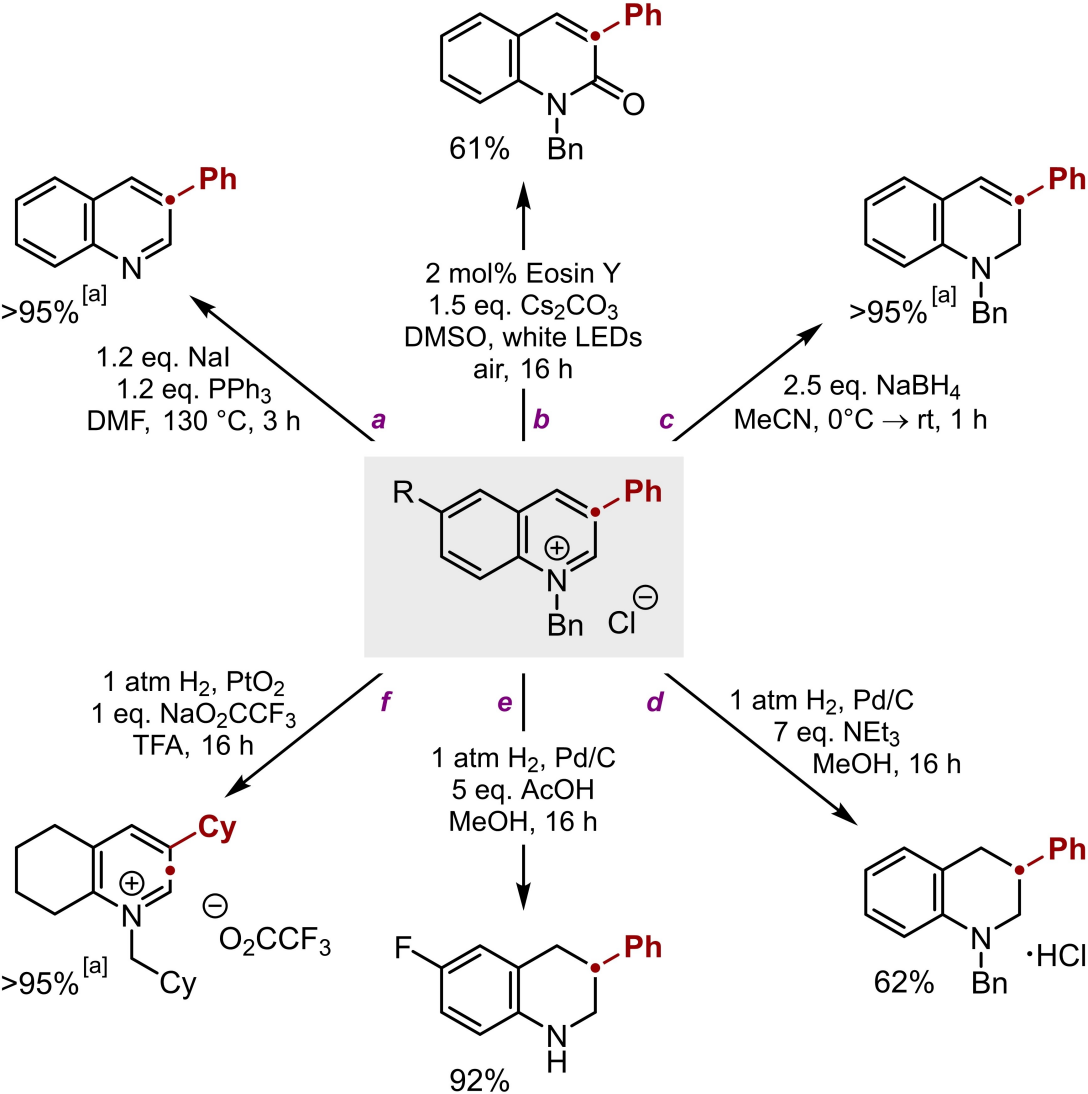
Derivatization of quinolinium salts. Yields refer to material isolated following purification.^[a]^ Yield determined by ^1^H NMR spectroscopic analysis vs internal standard.

In addition to its primary roles as a protecting group and a facilitator of product precipitation, the *N*‐substituent fulfills an important third purpose: as an activator of the heterocyclic core towards subsequent transformations. Thus, while the benzyl moiety can simply be removed under S_N_2 conditions[^54^, ^55^, ^56^] to liberate the corresponding quinoline (Scheme 4, pathway a), it also enables post‐expansion transformations that are accessible only to the azinium salt. For example, photocatalytic oxygenation affords the corresponding 2‐quinolone (pathway b),^[57]^ a key motif in numerous biologically‐active compounds,[^58^, ^59^, ^60^] whereas partial reduction with NaBH_4_ gives selectively the 1,2‐dihydroquinoline (pathway c). Complete reduction of the azinium core can also be achieved, and—by judicious choice of hydrogenation conditions—the benzyl substituent can either be retained or cleaved (pathways d and e). In this way it is possible to access the (un)protected tetrahydroquinoline in a concise fashion from an indole, a “skeletal edit” that involves not only ring expansion, but also a valuable increase in 3D character. Finally, hydrogenation with Adam's catalyst in TFA promotes complete reduction of all the benzenoid rings while leaving the azinium core untouched (pathway f).^[61]^

### Thermal Stability of Arylchlorodiazirines

To facilitate the safe utilization of arylchlorodiazirines, we performed a thermal hazard assessment using differential scanning calorimetry (DSC). To the best of our knowledge, only one DSC measurement of an arylchlorodiazirine has been reported previously,^[62]^ and this was made using an aluminum pan with a pierced lid as a gas vent, rather than in a sealed high‐pressure crucible of the type recommended for thermal safety profiling.^[63]^ While our measurements (see below) replicated the literature onset temperature well,^[62]^ we obtained an energetic yield that was larger by *ca* 30 kJ mol^−1^. To provide a broader assessment of the thermal hazards, we therefore performed DSC for seven electronically distinct diazirines using sealed high‐pressure crucibles (see Supporting Information for experimental details).

**Figure 1:**
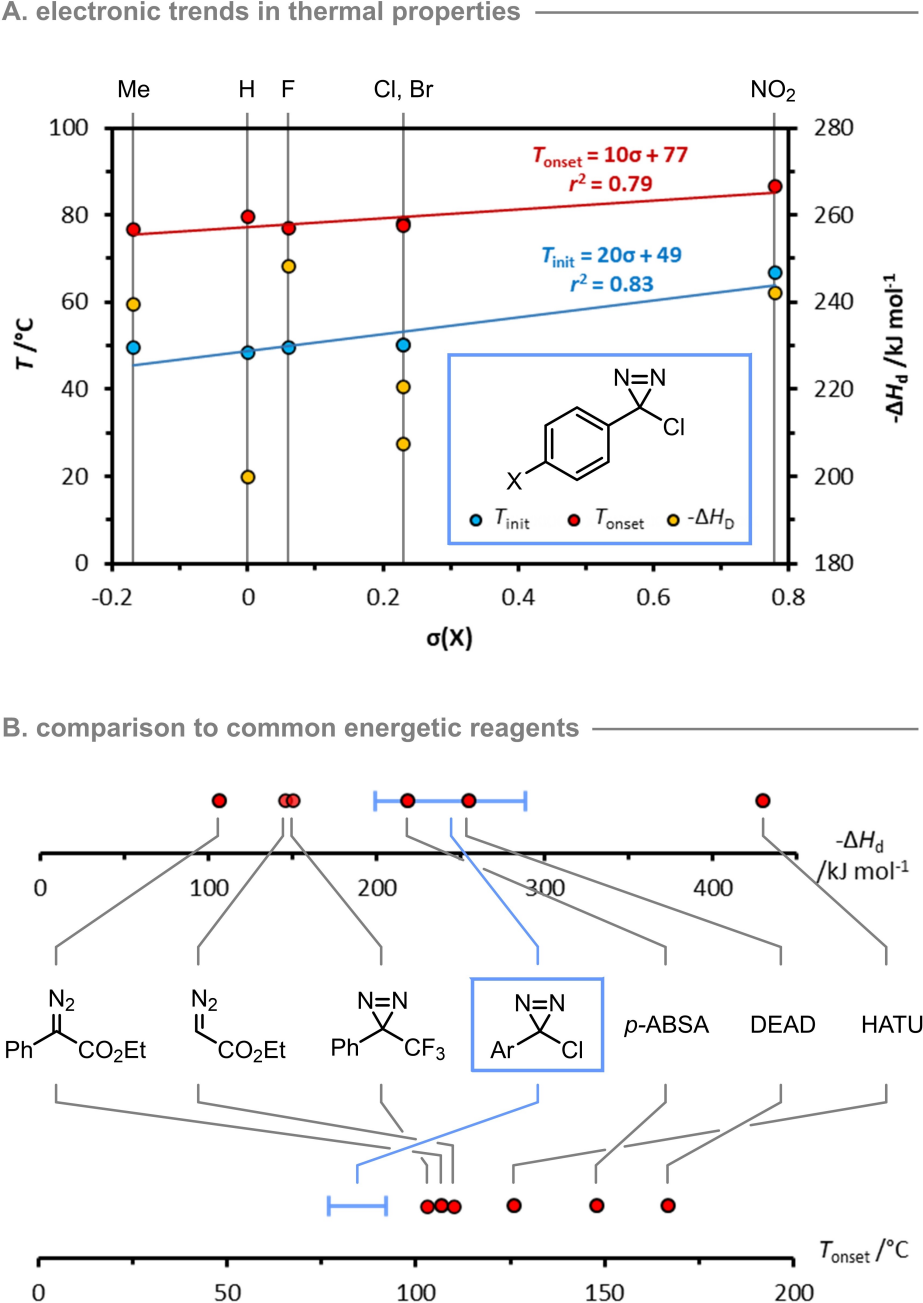
Graphical depiction of thermal stability data. a) dependence of *T*_init_, *T*_onset_ (left‐hand axis) and Δ*H*_d_ (right‐hand axis) on the electronic properties of arylchlorodiazirines; b) comparison of arylchlorodiazirines to common energetic laboratory reagents.[^62^, ^64^, ^66^]

As illustrated in Table 2, the diazirines exhibit initiation temperatures (*T*_init_, the temperature at which exothermic decomposition initiates) between 50 °C and 62 °C, and onset temperatures (*T*_onset_) between 77 °C and 92 °C. The *T*_init_ and *T*_onset_ values both correlate against the electronic properties of the aryl moiety (Figure 1A; red and blue data points), with electron donating groups destabilizing the diazirine and promoting decomposition at lower temperatures. The energetic yield of the decomposition process (Δ*H*_d_) spans nearly 50 kJ mol^−1^ for the phenylene‐substituted diazirines (200–248 kJ mol^−1^; Table 2, entries 1–6), whereas decomposition of the 2‐pyridyl diazirine is distinctly more energetic (entry 7). Unlike for *T*_init_ and *T*_onset_, the magnitude of Δ*H*_d_ does not show a significant correlation to the electronic properties of the diazirine (Figure 1A, yellow data points). While the Δ*H*_d_ values for the diazirines are similar to those of DEAD and *p*‐ABSA^[64]^—both of which are widely used in synthesis—the onset temperatures are significantly lower than for other common energetic reagents (Figure 1B).

**Table 2:** Thermal properties of arylchlorodiazirines measured by differential scanning calorimetry (DSC).^[a]^

Entry	Diazirine (Ar)	*T*_init_ [°C]	*T*_onset_ [°C]	−Δ*H*_d_ [J g^−1^]	−Δ*H*_d_ [kJ mol^−1^]	Yoshida		Pfizer		*T*_D24_ [°C]
						IS	EP	IS	EP	
1	4‐Me−C_6_H_4_	49.5	76.6	1438	240	0.32	0.22	0.81	0.47	−10
2	Ph	48.5	79.5	1309	200	0.27	0.17	0.78	0.43	−10
3	4‐F−C_6_H_4_	49.5	78.0	1455	248	0.32	0.22	0.81	0.48	−10
4	4‐Cl−C_6_H_4_	50.1	78.1	1109	207	0.20	0.10	0.69	0.35	−10
5	4‐Br−C_6_H_4_	50.2	77.7	952	220	0.14	0.03	0.62	0.29	−10
6	4‐NO_2_−C_6_H_4_	66.7	86.6	1225	242	0.20	0.12	0.61	0.33	0
7	2‐pyridyl	62.3	92.2	1884	289	0.36	0.29	0.82	0.53	0

[a] *T*_init_ is the temperature at which the normalized heatflow exceeds 0.01 W g^−1^ above the baseline; *T*_onset_ is the intersection of the maximum peak gradient and the baseline; Δ*H*_d_ is the integral area of the exotherm peak; IS (impact sensitivity) and EP (explosive propagation) calculated according to Equations (1) and (2) (Yoshida)^[65]^ or Equations (3) and (4) (Pfizer);^[66]^ *T*_D24_ calculated according to Equation (5),^[67]^ with values rounded to the nearest 5 °C.

The impact sensitivity (IS) and explosive propagation (EP) potential of the diazirines were calculated using the correlations developed by Yoshida^[65]^ [Eqs. (1) and (2)] and at Pfizer^[66]^ [Eqs. (3) and (4)]. In both cases, values above zero predict that the compound will exhibit impact sensitivity (IS) and/or explosive propagation (EP), although the method reported by Pfizer applies stricter limits on IS and EP by replacing *T*_onset_ with *T*_init_ in the relevant calculations. Irrespective of which correlation is applied to the DSC data, all the diazirines fall above the threshold for IS and EP (Table 2).

Yoshida:

(1) $$IS=\;{log}_{10}\left(Q\right)-0.72\left({log}_{10}\left(T_{onset}-25\right)\right)-0.98$$

(2) $$EP=\;{log}_{10}\left(Q\right)-0.38\left({log}_{10}\left(T_{onset}-25\right)\right)-1.67$$

Pfizer:

(3) $$IS=\;{log}_{10}\left(Q\right)-0.54\left({log}_{10}\left(T_{init}-25\right)\right)-0.98$$

(4) $$EP=\;{log}_{10}\left(Q\right)-0.285\left({log}_{10}\left(T_{init}-25\right)\right)-1.67$$

The final parameter of interest for the safe handling of arylchlorodiazirines is the recommended operating temperature. This has been defined by process chemists at GlaxoSmithKline as *T*_D24_ (equation 5)^[67]^—the temperature at which Time to Maximum Rate under adiabatic conditions is 24 hours—which provides a guide as to the highest recommended handling temperature for potentially explosive reagents.

(5) $$T_{D24}=0.7T_{init}-46$$

Due to the low *T*_init_ of the diazirines, their recommended handling temperatures all fall at or below 0 °C (Table 2). While this assessment does not present an issue when the diazirines are in storage at −20 °C, it suggests that significant hazards may arise during their synthesis, purification, and handling.

**Scheme 5:**
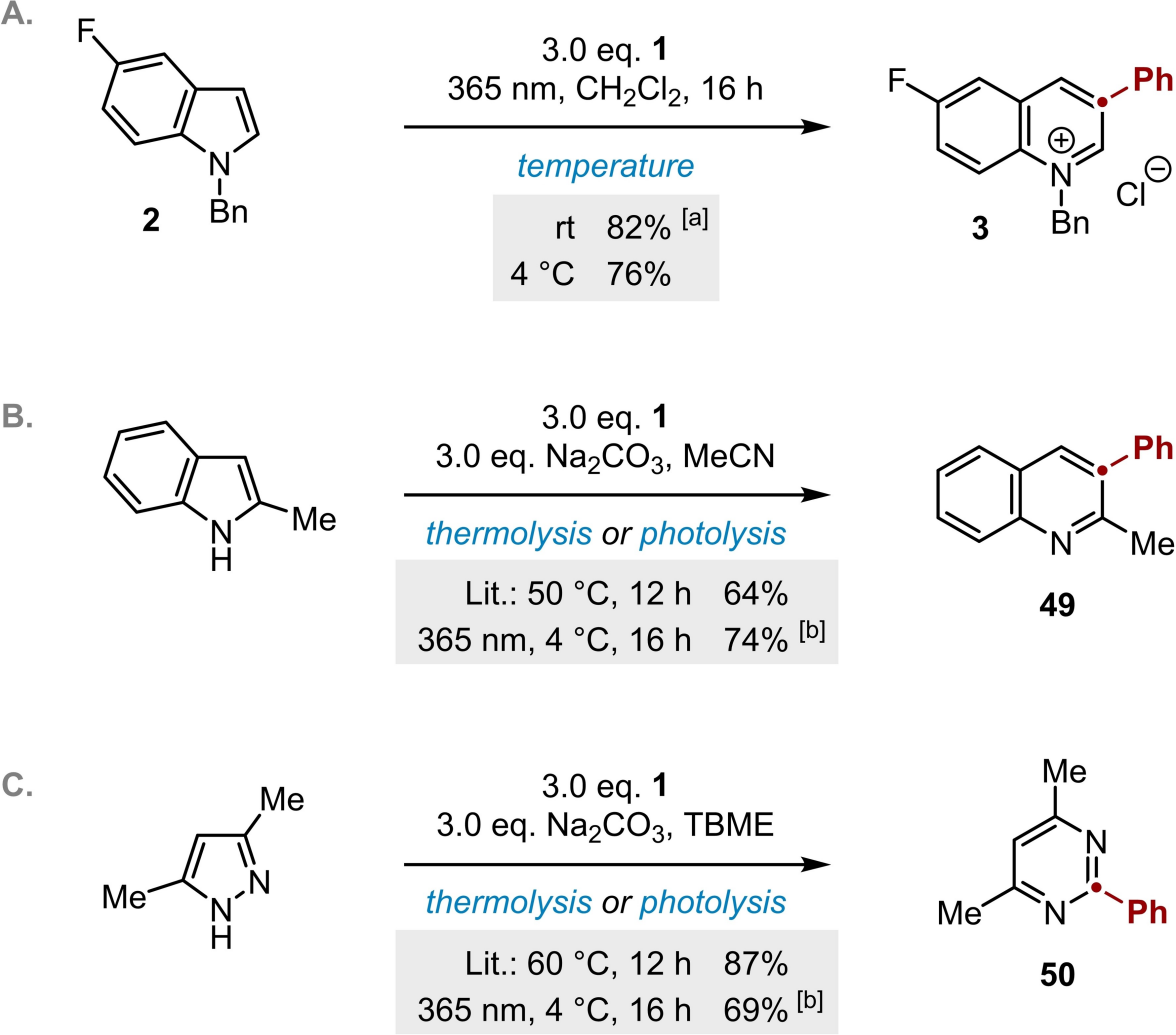
Applications of photolytic carbene generation at sub‐ambient temperatures. a) Synthesis of quinolinium salts; comparison to thermolytic methods for the synthesis of: b) quinolines,^[23]^ and c) pyrimidines.^[40]^ Yields refer to isolated material.^[a]^ “rt” refers to the internal temperature of the air‐cooled photoreactor (see Supporting Information), which did not rise more than 5 °C above ambient temperature over the course of a 16 h reaction.^[b]^ Yield determined by ^1^H NMR spectroscopic analysis vs internal standard.

The risks associated with diazirines can be reduced in our ring‐expansion methodology by performing the reaction at sub‐ambient temperatures. Indeed, comparable yields are obtained at room temperature and at 4 °C (Scheme 5A), with the lower temperature achieved conveniently by performing the reaction in a cold‐room. The ability to generate carbenes from diazirines at low temperature highlights a distinct safety benefit of photolysis over thermolysis. In preliminary studies, we have also found that this benefit can be extended to pioneering methods for azole^[23]^ and diazole^[40]^ ring expansion (Scheme 5B and C), which were originally reported as thermolytic procedures. As such, we hope that these extremely enabling synthetic methods might be used widely and with lower risk to the operator.

## Conclusion

We have developed a broadly applicable method for the one‐carbon ring‐expansion of *N*‐alkyl indoles, pyrroles and pyrazoles that uses arylchlorodiazirines as photo‐activated carbene precursors. A broad range of synthetically valuable functionality is tolerated and—for the first time—good yields are obtained for substrates where the azole nitrogen is not sterically shielded by adjacent substituents. The resulting aryl pyridinium, quinolinium and pyrimidinium salts are typically isolated by simple filtration, and can either be deprotected to the parent azine, or further functionalized by selective oxygenation or partial reductions. Although DSC analysis of the diazirines indicates that they are energetic compounds with low initiation and onset temperatures, safety can be improved by performing reactions at sub‐ambient temperatures. Overall, the scope of our methodology complements existing strategies for ring expansion, and we therefore anticipate that it will be of use in target‐oriented synthesis and library diversification.

## Conflict of interest

The authors declare no conflict of interest.

1

## Supporting information

As a service to our authors and readers, this journal provides supporting information supplied by the authors. Such materials are peer reviewed and may be re‐organized for online delivery, but are not copy‐edited or typeset. Technical support issues arising from supporting information (other than missing files) should be addressed to the authors.

Supporting Information

## Data Availability

The data that support the findings of this study are available in the Supporting Information of this article.
