# Supplementary material for: Photochemically Mediated Ring Expansion of Indoles and Pyrroles with Chlorodiazirines: Synthetic Methodology and Thermal Hazard Assessment
Source: Angew Chem Int Ed Engl. 2023 Jun 27;62(31):e202305081. doi: 10.1002/anie.202305081 (PMC11497286; doi:10.1002/anie.202305081)

Angewandte Chemie
Eine Zeitschrift der Gesellschaft Deutscher Chemiker

Supporting Information

Photochemically Mediated Ring Expansion of Indoles and Pyrroles with Chlorodiazirines: Synthetic Methodology and Thermal Hazard Assessment

*B. W. Joynson, G. R. Cumming, L. T. Ball**

Table of Contents

1. General Information 3
2. Synthesis and Characterization of Azinium Salts 5
   - 2.1. General Procedure 1 (GP-1): Ring Expansion of *N*-Alkylindoles 5
   - 2.2. General Procedure 2 (GP-2): Ring Expansion of *N*-Alkylpyrroles and *N*-Alkylpyrazoles 34
   - 2.3. General Procedure 3 (GP-3): *N*-Benzylation of Azoles 49
   - 2.4. General Procedure 4 (GP-4): Synthesis of Arylchlorodiazirines 89
   - 2.5. Synthesis of Amidine Hydrochlorides 95
3. Functionalisation of Azinium Salts 101
4. Robustness Screen 107
   - 4.1. General Procedure for robustness screen (GP-5) 107
5. Reaction Optimisation 110
   - 5.1. Assessment of *N*-Protecting Group 110
   - 5.2. Initial Solvent Screening 111
   - 5.3. Further Solvent Screening 112
   - 5.4. Comparison to Literature 113
   - 5.5. Reaction Robustness 114
6. Reaction Limitations 115
   - 6.1. Incompatible Substrates 115
   - 6.2. Poisoning Studies 117
7. DSC Analysis 118
8. References 121
9. NMR Spectra 123

1. General Information

Reagents were purchased from commercial suppliers and used as supplied. Sodium hypochlorite solution was either purchased from Sigma Aldrich (10-15%) or as commercial bleach (4.5%) and titrated against sodium thiosulfate in the presence of potassium iodide and HCl before use. Procedures requiring inert conditions were conducted in flame-dried glassware under an atmosphere of anhydrous dinitrogen using standard Schlenk techniques. Anhydrous solvents were obtained from in-house solvent purification systems (Inert® ProSolv; dried by passage through activated alumina columns under pressure of Ar) or by drying over activated 3 Å molecular sieves for 48 h followed by distillation. Deuterated chloroform was stored over 4 Å molecular sieves.

Flash column chromatography was accomplished using silica gel 60 Å (40-60 µm particle size) used as purchased from Sigma-Aldrich. Automated column chromatography was performed on disposable columns pre-packed with 50 µm silica gel using a Buchi Pure C-850 FlashPrep equipped with a UV-vis DAD (200-800 nm) and an ELSD. Analytical thin-layer chromatography was carried out on aluminium-backed silica gel plates (Merck/EMD Millipore, 60 Å pore size, precoated with a 254 nm-responsive fluorescent dye) and spots were visualised with UV irradiation (254 nm).

Photochemistry was achieved using a HepatoChem EvoluChem™ PhotoRedOx Box and an HCK1012-01-11 (365 nm) LED lamp operating at 18 W (relative irradiance: 9 mW cm^{-2}) with samples kept between 5–7 cm from the light source. Temperatures inside the photo-reactor were monitored and exceeded no more than 5 °C above ambient temperatures after 16 h of continuous operation.

NMR spectra were recorded at 298 K on Bruker-Avance 500 or 400 spectrometers (^{1}H, 500 / 400 MHz; ^{13}C, 125 / 101 MHz; ^{19}F, 471 / 376 MHz). Chemical shifts (δ) are reported in ppm; coupling constants, *J*, are reported in Hz. Signals are reported as singlet (s), doublet (d), triplet (t), quartet (q), multiplet (m), broad (br), apparent (app.) and combinations thereof. Chemical shifts are reported relative to tetramethylsilane (TMS) and referenced to the appropriate residual solvent peaks for ^{1}H and $^{13}C\{^{1}H\}$ NMR respectively:

$CDCl_3$: 7.26 ppm, 77.16 ppm

CD_3OD: 4.87, 3.34 ppm, 49.00 ppm

DMSO−d_6: 2.54 ppm, 39.52 ppm

A 30 s relaxation delay time (D_1) was used for quantitative ^{19}F NMR spectroscopy. NMR yields were calculated from ^{19}F NMR spectroscopy by comparison of integral ratios with the internal standard 4,4′-bis(trifluoromethyl)-1,1′-biphenyl (δ: -62.6 ppm in $CDCl_3$), which was prepared according to the literature method.[1]

High-resolution mass spectrometry (HRMS) was performed using a Bruker MicroTOF spectrometer, with an electrospray ionisation ion source. Infrared spectra of neat compounds were recorded over the range 4000-600 cm^{-1} using a PerkinElmerSpectrum 1000 Series FTIR spectrometer with an ATR diamond cell.

Differential Scanning Calorimetry (DSC) analysis was preformed using a TA Discovery DSC with reusable high pressure stainless steel capsules (TA Instruments; #900808.901) and gold-coated copper seals (TA Instruments; #900814.901). Calibration of the empty reference capsule was made against a capsule containing *ca.* 8 mg of indium metal. Analysis of DSC data was carried out in TRIOS software.

2. Synthesis and Characterization of Azinium Salts

2.1. General Procedure 1 (GP-1): Ring Expansion of *N*-Alkylindoles

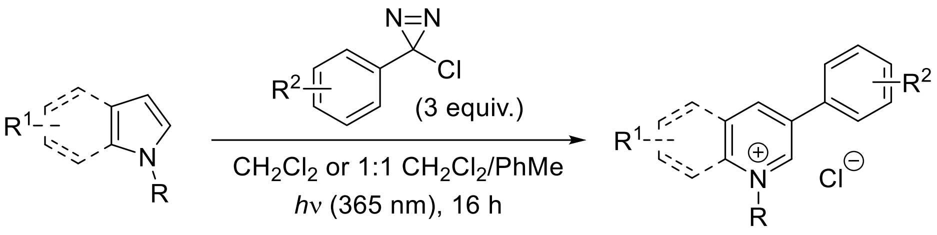

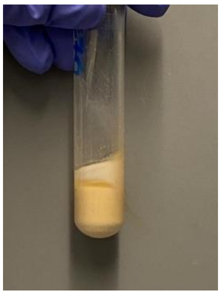

A 10 mL microwave tube was charged with *N*-alkylindole (0.2 mmol) which was then sealed with a crimp-cap fitted with a PTFE-faced silicone seal. The tube was evacuated and flushed with dinitrogen 3 times, then anhydrous CH_2Cl_2 or a 1:1 v/v mixture of anhydrous CH_2Cl_2/PhMe (2 mL) was added, followed by 3-chloro-3-aryldiazirine (0.6 mmol, unless specified otherwise). The cap of the reaction flask was then sealed with electrical tape. The reaction mixture was stirred under constant irradiation with UV light (365 nm, 18 W LED, 5 cm from light source) overnight. Unless specified otherwise, the resulting precipitate was isolated by filtration, washed with PhMe (2 × 5 mL), and dried under a flow of air to afford the pure product.

1-Benzyl-6-fluoro-3-phenylquinolin-1-ium chloride (3)

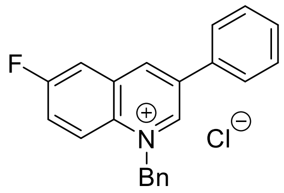

Synthesised according to **GP-1** from *N*-benzyl-5-fluoroindole (45 mg, 0.2 mmol) and 3-chloro-3-phenyldiazirine (92 mg, 0.6 mmol) in CH_2Cl_2 (2 mL) as an off-white solid (57.3 mg, 0.164 mmol, 82%).

^{1}H NMR (400 MHz, CD_3OD): δ 10.01 (d, *J* = 2.0 Hz, 1H), 9.53 (d, *J* = 2.0 Hz, 1H), 8.56 (dd, *J* = 9.7, 4.3 Hz, 1H), 8.23 (dd, *J* = 8.0, 2.9 Hz, 1H), 8.04–7.94 (m, 3H), 7.72–7.56 (m, 3H), 7.49–7.33 (m, 5H), 6.46 (s, 2H).

^{13}C{^{1}H} NMR (101 MHz, CD_3OD): δ 163.4 (d, *J* = 254.8 Hz), 149.9, 145.2 (d, *J* = 5.3 Hz), 137.6, 135.6, 134.7, 134.5, 133.7 (d, *J* = 11.0 Hz), 131.5, 130.9, 130.6, 130.4, 128.8, 128.3, 126.5 (d, *J* = 27.0 Hz), 123.6 (d, *J* = 9.5 Hz), 115.5 (d, *J* = 23.3 Hz), 62.9.

^{19}F NMR (376 MHz, CD_3OD): δ −107.84 (app td, *J* = 8.0, 4.3 Hz).

ν_{max} (neat) / cm^{-1}: 3025, 2941, 1584, 1492, 1454, 1364.

HRMS: calcd. for $C_{22}H_{17}FN$ [M-Cl]$^+$: 314.1340; found (ESI$^+$): 314.1351.

m.p. / °C: 256-258.

1-Benzyl-6-chloro-3-phenylquinolin-1-ium chloride (4)

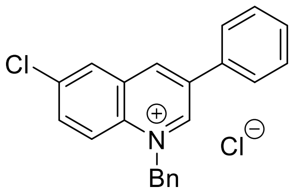

Synthesised according to **GP-1** from *N*-benzyl-5-chloroindole (48 mg, 0.2 mmol) and 3-chloro-3-phenyldiazirine (153 mg, 1.0 mmol) in CH_2Cl_2 (2 mL) as an off-white solid (52.1 mg, 0.142 mmol, 71%).

^{1}H NMR (400 MHz, CD_3OD): δ 10.03 (d, *J* = 2.0 Hz, 1H), 9.52 (d, *J* = 2.0 Hz, 1H), 8.58 (d, *J* = 2.4 Hz, 1H), 8.49 (d, *J* = 9.5 Hz, 1H), 8.14 (dd, *J* = 9.5, 2.4 Hz, 1H), 8.05–7.97 (m, 2H), 7.73–7.61 (m, 3H), 7.51–7.35 (m, 5H), 6.45 (s, 2H).

^{13}C{^{1}H} NMR (101 MHz, CD_3OD): δ 150.5, 144.9, 137.8, 137.7, 137.1, 137.0, 134.7, 134.4, 132.9, 131.5, 131.0, 130.72, 130.66, 130.5, 128.8, 128.3, 122.3, 62.8.

ν_{max} (neat) / cm^{-1}: 2925, 1523, 1492, 1376, 1357, 1096, 909, 833.

HRMS: calcd. for $C_{22}H_{17}NCl$ [M-Cl]$^+$: 330.1044; found (ESI$^+$): 330.1048.

m.p. / °C: 247-251.

1-Benzyl-6-bromo-3-phenylquinolin-1-ium chloride (5)

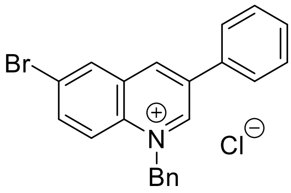

Synthesised according to **GP-1** from *N*-benzyl-5-bromoindole (57 mg, 0.2 mmol) and 3-chloro-3-phenyldiazirine (153 mg, 1.0 mmol) in CH_2Cl_2 (2 mL) as an off-white solid (47.3 mg, 0.115 mmol, 58%).

^{1}H NMR (400 MHz, CD_3OD): δ 10.03 (d, *J* = 2.0 Hz, 1H), 9.51 (d, *J* = 2.0 Hz, 1H), 8.75 (d, *J* = 2.2 Hz, 1H), 8.40 (d, *J* = 9.5 Hz, 1H), 8.26 (dd, *J* = 9.4, 2.2 Hz, 1H), 8.05 – 7.93 (m, 2H), 7.73 – 7.59 (m, 3H), 7.51 – 7.37 (m, 5H), 6.44 (s, 2H).

^{13}C{^{1}H} NMR (101 MHz, CD_3OD): δ 150.6, 144.8, 139.6, 137.8, 137.3, 134.7, 134.4, 134.1, 133.2, 131.5, 131.0, 130.7, 130.5, 128.8, 128.3, 125.7, 122.1, 62.7.

ν_{max} (neat) / cm^{-1}: 3017, 2922, 1521, 1491, 1452, 1375, 1354, 833.

HRMS: calcd. for $C_{22}H_{17}NBr$ [M-Cl]$^+$: 374.0539; found (ESI$^+$): 374.0542.

m.p. / °C: 236-238.

1-Benzyl-6-cyano-3-phenylquinolin-1-ium chloride (6)

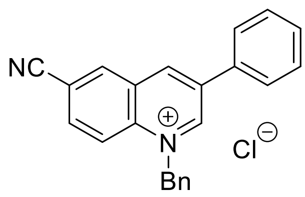

Synthesised according to **GP-1** from *N*-benzylindole-5-carbonitrile (50 mg, 0.2 mmol) and 3-chloro-3-phenyldiazirine (92 mg, 0.6 mmol) in CH_2Cl_2 (2 mL) as an off-white solid (39.8 mg, 0.111 mmol, 56%).

^{1}H NMR (400 MHz, CD_3OD): δ 10.19 (d, *J* = 2.1 Hz, 1H), 9.68 (d, *J* = 2.1 Hz, 1H), 9.01 (d, *J* = 1.9 Hz, 1H), 8.64 (d, *J* = 9.3 Hz, 1H), 8.35 (dd, *J* = 9.3, 1.9 Hz, 1H), 8.08 – 7.99 (m, 2H), 7.77 – 7.60 (m, 3H), 7.44 (m, 5H), 6.50 (s, 2H).

^{13}C{^{1}H} NMR (101 MHz, CD_3OD): δ 152.9, 146.3, 139.5, 138.3, 138.1, 136.9, 134.4, 134.2, 131.7, 131.6, 131.0, 130.7, 130.6, 128.8, 128.4, 122.0, 117.6, 115.6, 62.9.

ν_{max} (neat) / cm^{-1}: 2925, 1529, 1492, 1360, 836.

HRMS: calcd. for $C_{23}H_{17}N_2$ [M-Cl]$^+$: 341.1285; found (ESI$^+$): 341.1284.

m.p. / °C: 234-237.

1-Benzyl-6-nitro-3-phenylquinolin-1-ium chloride (7)

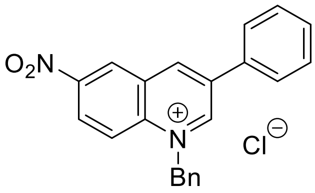

Synthesised according to **GP-1** from *N*-benzyl-5-nitroindole (50 mg, 0.2 mmol) and 3-chloro-3-phenyldiazirine (153 mg, 1.0 mmol) in CH_2Cl_2 (2 mL) as a yellow solid (19.9 mg, 0.54 mmol, 27%).

^{1}H NMR (400 MHz, CD_3OD): δ 10.23 (d, *J* = 2.0 Hz, 1H), 9.83 (d, *J* = 2.0 Hz, 1H), 9.46 (d, *J* = 2.5 Hz, 1H), 8.85 (dd, *J* = 9.7, 2.5 Hz, 1H), 8.71 (d, *J* = 9.7 Hz, 1H), 8.07–8.01 (m, 2H), 7.77–7.62 (m, 3H), 7.49–7.40 (m, 5H), 6.52 (s, 2H).

^{13}C{^{1}H} NMR (101 MHz, CD_3OD): δ 153.3, 149.0, 147.5, 140.3, 138.5, 134.3, 134.2, 131.9, 131.8, 131.0, 130.7, 130.6, 129.4, 128.9, 128.4, 128.1, 122.7, 63.2.

ν_{max} (neat) / cm^{-1}: 3002, 2924, 1631, 1609, 1345, 1184, 823.

HRMS: calcd. for $C_{22}H_{17}N_2O_2$ [M-Cl]$^+$: 341.1285; found (ESI$^+$): 341.1284.

m.p. / °C: 229-230.

1-Benzyl-3-phenylquinolin-1-ium chloride (8)

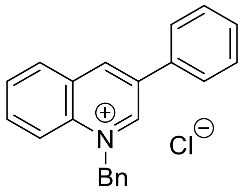

Synthesised according to **GP-1** from *N*-benzylindole (41 mg, 0.2 mmol) and 3-chloro-3-phenyldiazirine (92 mg, 0.6 mmol) in 1:1 CH_2Cl_2/PhMe (2 mL) as an off-white solid (46.4 mg, 0.140 mmol, 70%).

^{1}H NMR (400 MHz, CD_3OD): δ 10.00 (d, *J* = 2.1 Hz, 1H), 9.57 (d, *J* = 1.6 Hz, 1H), 8.53 (dd, *J* = 8.3, 1.4 Hz, 1H), 8.49 (d, *J* = 9.0 Hz, 1H), 8.16 (ddd, *J* = 8.7, 7.0, 1.6 Hz, 1H), 8.06–7.95 (m, 3H), 7.71–7.66 (m, 2H), 7.63-7.60 (m, 1H), 7.47–7.34 (m, 5H), 6.45 (s, 2H).

^{13}C{^{1}H} NMR (101 MHz, CD_3OD): δ 150.1, 146.0, 138.5, 136.9, 136.7, 135.0, 134.7, 132.4, 132.1, 131.7, 131.2, 130.9, 130.6, 130.3, 128.8, 128.2, 120.2, 62.5.

ν_{max} (neat) / cm^{-1}: 2941, 1584, 1528, 1492, 1364.

HRMS: calcd. for $C_{22}H_{18}N$ [M-Cl]$^+$: 296.1434; found (ESI$^+$): 296.1428.

m.p. / °C: 229-231.

1-Benzyl-6-(benzyloxy)-3-phenylquinolin-1-ium chloride (9)

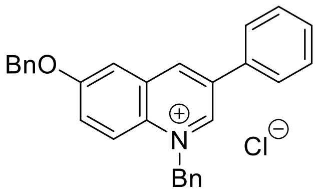

Synthesised according to **GP-1** from *N*-benzyl-5-benzyloxyindole (62 mg, 0.2 mmol) and 3-chloro-3-phenyldiazirine (92 mg, 0.6 mmol) in 1:1 CH_2Cl_2/PhMe (2 mL) as a yellow solid (66.2 mg, 0.151 mmol, 76%).

^{1}H NMR (400 MHz, CD_3OD): δ 9.78 (d, *J* = 2.0 Hz, 1H), 9.38 (d, *J* = 2.0 Hz, 1H), 8.38 (d, *J* = 9.7 Hz, 1H), 8.00-7.94 (m, 2H), 7.92 (d, *J* = 2.8 Hz, 1H), 7.83 (dd, *J* = 9.7, 2.8 Hz, 1H), 7.70–7.57 (m, 3H), 7.54–7.48 (m, 2H), 7.46–7.31 (m, 8H), 6.38 (s, 2H), 5.36 (s, 2H).

^{13}C{^{1}H} NMR (101 MHz, CD_3OD): δ 160.8, 147.0, 144.1, 137.1, 137.0, 135.2, 134.8, 134.3, 134.2, 131.2, 130.9, 130.6, 130.3, 129.8, 129.5, 129.0, 128.7, 128.1, 121.8, 110.5, 72.1, 62.5.

ν_{max} (neat) / cm^{-1}: 3030, 2947, 1612, 1531, 1453, 1397, 1272, 1212, 1153.

HRMS: calcd. for $C_{29}H_{24}NO$ [M-Cl]$^+$: 402.1853; found (ESI$^+$): 402.1868.

m.p. / °C: 200-203.

1-Benzyl-6-methoxy-3-phenylquinolin-1-ium chloride (10)

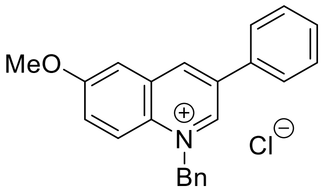

Synthesised according to **GP-1** from *N*-benzyl-5-methoxyindole (47 mg, 0.2 mmol) and 3-chloro-3-phenyldiazirine (92 mg, 0.6 mmol) in 1:1 CH_2Cl_2/PhMe (2 mL) as a red solid (42.2 mg, 0.117 mmol, 58%).

^{1}H NMR (400 MHz, CD_3OD): δ 9.80 (d, *J* = 2.0 Hz, 1H), 9.41 (dd, *J* = 2.0, 0.9 Hz, 1H), 8.38 (dd, *J* = 9.7, 0.9 Hz, 1H), 8.04–7.95 (m, 2H), 7.86 (d, *J* = 2.9 Hz, 1H), 7.77 (dd, *J* = 9.7, 2.9 Hz, 1H), 7.71–7.59 (m, 3H), 7.49–7.33 (m, 5H), 6.40 (s, 2H), 4.07 (s, 3H).

^{13}C{^{1}H} NMR (101 MHz, CD_3OD): δ 161.9, 146.8, 144.0, 137.0, 135.2, 134.8, 134.28, 134.27, 131.2, 130.9, 130.6, 130.3, 129.5, 128.7, 128.1, 121.7, 109.2, 62.5, 57.0.

ν_{max} (neat) / cm^{-1}: 2946, 1621, 1534, 1492, 1464, 1398, 1273, 1215.

HRMS: calcd. for $C_{23}H_{20}NO$ [M-Cl]$^{+}$: 326.1539; found (ESI^{+}): 326.1536.

m.p. / °C: 225-228.

1-Benzyl-6-methyl-3-phenylquinolin-1-ium chloride (11)

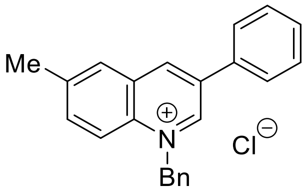

Synthesised according to **GP-1** from *N*-benzyl-5-methylindole (44 mg, 0.2 mmol) and 3-chloro-3-phenyldiazirine (92 mg, 0.6 mmol) in 1:1 CH_2Cl_2/PhMe (2 mL) as an off-white solid (50.5 mg, 0.163 mmol, 81%).

^{1}H NMR (400 MHz, CD_3OD): δ 9.93 (d, *J* = 2.1 Hz, 1H), 9.47 (dd, *J* = 2.1, 0.9 Hz, 1H), 8.37 (d, *J* = 9.1 Hz, 1H), 8.29 (s, 1H), 8.04 – 7.98 (m, 3H), 7.73 – 7.65 (m, 2H), 7.64 – 7.59 (m, 1H), 7.48 – 7.33 (m, 5H), 6.44 (s, 2H), 2.67 (s, 3H).

^{13}C{^{1}H} NMR (101 MHz, CD_3OD): δ 148.9, 145.1, 143.0, 139.0, 137.1, 136.6, 135.1, 134.8, 132.3, 131.2, 130.9, 130.8, 130.6, 130.3, 128.7, 128.2, 119.9, 62.4, 21.3.

ν_{max} (neat) / cm^{-1}: 2935, 1535, 1493, 1384, 1362, 817.

HRMS: calcd. for $C_{23}H_{20}N$ [M-Cl]$^+$: 310.1590; found (ESI$^+$): 310.1595.

m.p. / °C: 236-238.

1-Benzyl-7-(benzyloxy)-3-phenylquinolin-1-ium chloride (12)

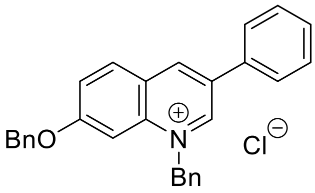

Synthesised according to **GP-1** from *N*-benzyl-6-methoxylindole (62 mg, 0.2 mmol) and 3-chloro-3-phenyldiazirine (92 mg, 0.6 mmol) in 1:1 CH_2Cl_2/PhMe (2 mL) as a yellow solid (62.7 mg, 0.143 mmol, 72%).

^{1}H NMR (400 MHz, CD_3OD): δ 9.81 (d, *J* = 2.0 Hz, 1H), 9.43 (d, *J* = 2.0 Hz, 1H), 8.41 (d, *J* = 9.7 Hz, 1H), 8.00 – 7.94 (m, 2H), 7.74 – 7.62 (m, 4H), 7.61 – 7.55 (m, 1H), 7.47 – 7.31 (m, 10H), 6.35 (s, 2H), 5.32 (s, 2H).

^{13}C{^{1}H} NMR (101 MHz, CD_3OD): δ 166.1, 148.4, 145.2, 141.1, 136.5, 135.2, 134.6, 134.2, 134.0, 130.8, 130.7, 130.3, 129.9, 129.7, 128.9, 128.5, 128.2, 127.8, 124.7, 100.8, 72.5, 62.1.

ν_{max} (neat) / cm^{-1}: 2960, 1630, 1607, 1496, 1454, 1383, 1269, 1201, 1020, 837.

HRMS: calcd. for $C_{29}H_{24}NO$ [M-Cl]$^+$: 402.1853; found (ESI$^+$): 402.1847.

m.p. / °C: 212-214.

1-Benzyl-6,7-dimethoxy-3-phenylquinolin-1-ium chloride (13)

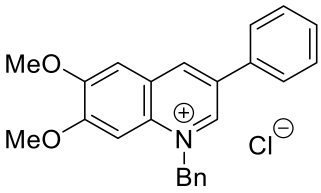

Synthesised according to **GP-1** from *N*-benzyl-5,6-dimethoxyindole (53 mg, 0.2 mmol) and 3-chloro-3-phenyldiazirine (92 mg, 0.6 mmol) in 1:1 CH_2Cl_2/PhMe (2 mL) as a yellow solid (58.5 mg, 0.150 mmol, 75%).

^{1}H NMR (400 MHz, CD_3OD): δ 9.64 (d, *J* = 1.9 Hz, 1H), 9.29 (d, *J* = 1.9 Hz, 1H), 8.00 – 7.86 (m, 2H), 7.80 (s, 1H), 7.69 – 7.62 (m, 2H), 7.62 – 7.55 (m, 2H), 7.49 – 7.39 (m, 5H), 6.38 (s, 2H), 4.09 (s, 3H), 4.01 (s, 3H).

^{13}C{^{1}H} NMR (101 MHz, CD_3OD): δ 159.1, 153.9, 144.8, 142.7, 136.6, 135.5, 134.8, 134.6, 130.8, 130.8, 130.6, 130.3, 129.1, 128.5, 128.4, 108.7, 99.5, 62.3, 57.7, 57.2.

ν_{max} (neat) / cm^{-1}: 2954, 1626, 1508, 1427, 1281, 1256, 1230, 990.

HRMS: calcd. for $C_{24}H_{22}NO_2$ [M-Cl]$^+$: 356.1645; found (ESI$^+$): 356.1647.

m.p. / °C: 211-212.

1-Benzyl-5-methyl-3-phenylquinolin-1-ium chloride (14)

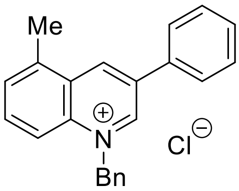

Synthesised according to **GP-1** from *N*-benzyl-4-methylindole (44 mg, 0.2 mmol) and 3-chloro-3-phenyldiazirine (92 mg, 0.6 mmol) in 1:1 CH_2Cl_2/PhMe (2 mL) as an amber solid (24.4 mg, 0.071 mmol, 35%).

^{1}H NMR (400 MHz, CD_3OD) δ 9.99 (d, *J* = 2.0 Hz, 1H), 9.57 (dd, *J* = 2.0, 1.0 Hz, 1H), 8.31 (d, *J* = 9.0 Hz, 1H), 8.08 – 7.98 (m, 3H), 7.87 (app dt, *J* = 7.1, 1.0 Hz, 1H), 7.74 – 7.66 (m, 2H), 7.66 – 7.59 (m, 1H), 7.48 – 7.35 (m, 5H), 6.45 (s, 2H), 3.00 (s, 3H).

^{13}C{^{1}H} NMR (101 MHz, CD_3OD): δ 149.5, 142.5, 140.9, 139.1, 136.6, 136.2, 135.3, 134.9, 132.1, 131.5, 131.1, 130.9, 130.6, 130.3, 129.0, 128.1, 118.2, 62.8, 19.2.

ν_{max} (neat) / cm^{-1}: 2946, 1587, 1491, 1434, 1367, 1343.

HRMS: calcd. for $C_{23}H_{20}N$ [M-Cl]$^+$: 310.1590; found (ESI$^+$): 310.1595.

m.p. / °C: 214-216.

1-Benzyl-8-methyl-3-phenylquinolin-1-ium chloride (15)

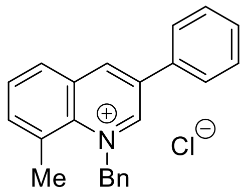

Synthesised according to **GP-1** from *N*-benzyl-7-methylindole (44 mg, 0.2 mmol) and 3-chloro-3-phenyldiazirine (92 mg, 0.6 mmol) in 1:1 CH_2Cl_2/PhMe (2 mL) as an amber solid (23.4 mg, 0.068 mmol, 34%).

^{1}H NMR (400 MHz, CD_3OD δ 9.77 (d, *J* = 2.2 Hz, 1H), 9.60 (d, *J* = 2.2 Hz, 1H), 8.45 – 8.36 (m, 1H), 8.05 (app dt, *J* = 7.0, 1.3 Hz, 1H), 8.00 – 7.89 (m, 3H), 7.69 – 7.52 (m, 3H), 7.47 – 7.37 (m, 3H), 7.12 – 6.99 (m, 2H), 6.66 (s, 2H), 3.01 (s, 3H).

^{13}C{^{1}H} NMR (101 MHz, CD_3OD) δ 152.3, 147.2, 142.0, 139.8, 137.0, 135.9, 134.4, 134.0, 131.9, 131.4, 131.3, 130.9, 130.7, 130.5, 129.9, 128.5, 126.6, 65.3, 24.5.

ν_{max} (neat) / cm^{-1}: 3031, 2957, 1533, 1494, 1451, 1352, 1231, 1170, 1022, 967, 818.

HRMS: calcd. for $C_{23}H_{20}N$ [M-Cl]$^+$: 310.1590; found (ESI$^+$): 310.1587.

m.p. / °C: 158-161.

1-Benzyl-2,4-dimethyl-3-phenylquinolin-1-ium chloride (18)

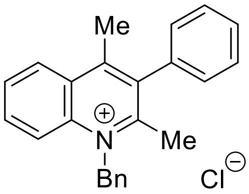

Synthesised according to **GP-1** from *N*-benzyl-2,3-dimethylindole (47 mg, 0.2 mmol) and 3-chloro-3-phenyldiazirine (92 mg, 0.6 mmol) in 1:1 CH_2Cl_2/PhMe (2 mL). The solvent was removed *in vacuo* and the product triturated from PhMe to afford the product as a brown solid (42.3 mg, 0.118 mmol, 59%).

^{1}H NMR (400 MHz, CD_3OD): δ 8.64 (dd, *J* = 8.5, 1.5 Hz, 1H), 8.37 (d, *J* = 8.9 Hz, 1H), 8.14 (ddd, *J* = 8.9, 7.0, 1.5 Hz, 1H), 8.03 (ddd, *J* = 8.2, 7.0, 1.0 Hz, 1H), 7.73 – 7.55 (m, 3H), 7.48 – 7.33 (m, 5H), 7.23 – 7.12 (m, 2H), 6.38 (s, 2H), 2.77 (s, 3H), 2.75 (s, 3H).

^{13}C{^{1}H} NMR (101 MHz, CD_3OD): δ 160.7, 157.6, 139.8, 139.0, 137.4, 136.3, 134.2, 130.8, 130.71, 130.66, 130.50, 130.48, 129.7, 128.6, 126.7, 120.7, 56.8, 22.0, 18.9.

ν_{max} (neat) / cm^{-1}: 3032, 1579, 1510, 1494, 1446, 1346, 1163.

HRMS: calcd. for $C_{24}H_{22}N$ [M-Cl]$^+$: 324.1747; found (ESI$^+$): 324.1751.

m.p. / °C: 94 (decomp.).

1-Methyl-6-fluoro-3-phenylquinolin-1-ium chloride (19)

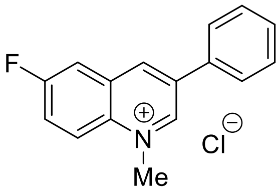

Synthesised according to **GP-1** from *N*-methyl-5-fluoroindole (30 mg, 0.2 mmol) and 3-chloro-3-phenyldiazirine (92 mg, 0.6 mmol) in 1:1 CH_2Cl_2/PhMe (2 mL) as a yellow solid (22.3 mg, 0.082 mmol, 41%).

^{1}H NMR (400 MHz, CD_3OD): δ 9.81 (d, *J* = 2.0 Hz, 1H), 9.45 (d, *J* = 2.0 Hz, 1H), 8.64 (dd, *J* = 9.7, 4.3 Hz, 1H), 8.22 (dd, *J* = 8.0, 2.8 Hz, 1H), 8.13 (ddd, *J* = 9.7, 8.0, 2.9 Hz, 1H), 8.04 – 7.94 (m, 2H), 7.78 – 7.57 (m, 3H), 4.81 (s, 3H).

^{19}F NMR (376 MHz, CD_3OD): δ -108.88 (app td, *J* = 8.0, 4.3 Hz).

^{13}C{^{1}H} NMR (101 MHz, CD_3OD): δ 163.5 (d, *J* = 254.2 Hz), 150.2, 144.0 (d, *J* = 5.3 Hz), 137.3, 136.3, 134.8, 132.9 (d, *J* = 10.9 Hz), 131.3, 130.9, 128.7, 126.3 (d, *J* = 26.9 Hz), 123.1 (d, *J* = 9.6 Hz), 115.0 (d, *J* = 23.2 Hz), 46.7.

ν_{max} (neat) / cm^{-1}: 3034, 2961, 1617, 1535, 1389, 1272, 1231, 1173, 967, 814.

HRMS: calcd. for $C_{16}H_{13}FN$ $[M-Cl]^+$: 238.1027; found (ESI^+): 238.1033.

m.p. / °C: 240 (decomp.).

1-(4-methoxybenzyl)-6-fluoro-3-phenylquinolin-1-ium chloride (20)

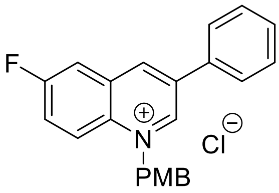

Synthesised according to **GP-1** from *N*-(4-methoxybenzyl)-5-fluoroindole (51 mg, 0.2 mmol) and 3-chloro-3-phenyldiazirine (92 mg, 0.6 mmol) in 1:1 CH_2Cl_2/PhMe (2 mL) as a yellow solid (40.4 mg, 0.106 mmol, 53%).

^{1}H NMR (400 MHz, CD_3OD): δ 9.92 (d, *J* = 1.8 Hz, 1H), 9.50 (d, *J* = 1.8 Hz, 1H), 8.64 (dd, *J* = 9.7, 4.3 Hz, 1H), 8.22 (dd, *J* = 8.1, 2.8 Hz, 1H), 8.03 (ddd, *J* = 9.7, 8.1, 2.8 Hz, 1H), 7.99 – 7.94 (m, 2H), 7.70 – 7.61 (m, 3H), 7.41 (d, *J* = 8.8 Hz, 2H), 7.00 (d, *J* = 8.8 Hz, 2H), 6.36 (s, 2H), 3.80 (s, 3H).

^{13}C{^{1}H} NMR (101 MHz, CD_3OD): δ 163.4 (d, *J* = 254.5 Hz), 162.1, 149.4 (d, *J* = 1.2 Hz), 145.0 (d, *J* = 5.3 Hz), 137.5, 135.6, 134.8, 133.7 (d, *J* = 10.9 Hz), 131.4, 131.0, 130.3, 128.8, 126.4 (d, *J* = 26.9 Hz), 125.9, 123.6 (d, *J* = 9.5 Hz), 116.0, 115.4 (d, *J* = 23.2 Hz), 62.6, 55.9.

ν_{max} (neat) / cm^{-1}: 3083, 2929, 1535, 1253, 1183, 1023, 831.

HRMS: calcd. for $C_{23}H_{19}FNO$ [M-Cl]$^+$: 344.1445; found (ESI$^+$): 344.1444.

m.p. / °C: 183-185.

4-(2-Acetamidoethyl)-1-benzyl-6-methoxy-3-phenylquinolin-1-ium chloride (22)

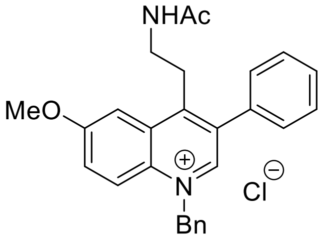

Synthesised according to **GP-1** from *N*-benzylmelatonin (64 mg, 0.2 mmol) and 3-chloro-3-phenyldiazirine (92 mg, 0.6 mmol) in 1:1 CH_2Cl_2/PhMe (2 mL) as a yellow solid (25.7 mg, 0.056 mmol, 29%).

^{1}H NMR (400 MHz, CD_3OD): δ 9.31 (s, 1H), 8.40 (d, J = 9.7 Hz, 1H), 8.33 – 8.28 (m, 1H), 8.23 (d, J = 2.7 Hz, 1H), 7.79 (dd, J = 9.7, 2.5 Hz, 1H), 7.69 – 7.58 (m, 5H), 7.46 – 7.34 (m, 5H), 6.29 (s, 2H), 4.16 (s, 3H), 3.56 – 3.45 (m, 4H), 1.86 (s, 3H).

^{13}C{^{1}H} NMR (101 MHz, CD_3OD): δ 173.8, 162.1, 155.9, 147.1, 138.3, 136.1, 134.9, 134.1, 133.4, 130.8, 130.6, 130.6, 130.4, 130.3, 128.9, 128.1, 122.4, 106.5, 62.0, 57.4, 40.5, 31.7, 22.4.

ν_{max} (neat) / cm^{-1}: 2931, 1616, 1532, 1368, 1242, 1027.

HRMS: calcd. for $C_{27}H_{27}N_2O_2$ [M-Cl]$^+$: 411.2067; found (ESI$^+$): 411.2076.

m.p. / °C: 115 (decomp.).

Methyl (*S*)-4-(2-acetamido-3-(4-fluorophenyl)-1-benzyl-3-(4-fluorophenyl)quinolin-1-ium chloride (23)

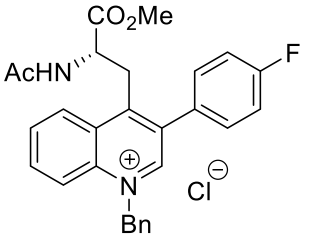

Synthesised according to **GP-1** from *N*-acetyl-1-benzyltryptophan methyl ester (70 mg, 0.2 mmol) and 3-chloro-3-(4-fluorophenyl)diazirine (102 mg, 0.6 mmol) in 1:1 CH_2Cl_2/PhMe (2 mL) as an orange solid (69.2 mg, 0.144 mmol, 72%).

^{1}H NMR (400 MHz, CD_3OD): δ 9.55 (s, 1H), 8.75 (dd, *J* = 8.8, 1.4 Hz, 1H), 8.47 (d, *J* = 8.8 Hz, 1H), 8.18 (ddd, *J* = 8.8, 7.0, 1.4 Hz, 1H), 8.13 – 8.04 (m, 1H), 7.70 (dd, *J* = 8.7, 5.2 Hz, 2H), 7.48 – 7.37 (m, 5H), 7.37 – 7.28 (m, 2H), 6.39 (d, *J* = 15.8 Hz, 1H), 6.34 (d, *J* = 15.8 Hz, 1H), 4.77 (dd, *J* = 9.7, 5.3 Hz, 1H), 4.01 (dd, *J* = 13.7, 5.3 Hz, 1H), 3.89 (dd, *J* = 13.7, 9.7 Hz, 1H), 3.64 (s, 3H), 1.60 (s, 3H).

^{13}C{^{1}H} NMR (101 MHz, CD_3OD): δ 172.7, 171.2, 164.9 (d, *J* = 248.7 Hz), 157.7, 150.6, 138.5, 137.6, 136.4, 134.8, 133.4 (d, *J* = 8.6 Hz), 131.8 (d, *J* = 3.4 Hz), 131.5, 131.4, 130.6, 130.2, 128.8, 127.9, 121.0, 117.4 (d, *J* = 22.2 Hz), 62.1, 53.7, 53.3, 33.4, 22.1.

^{19}F NMR (376 MHz, CD_3OD): δ -113.40 (tt, *J* = 9.1, 5.3 Hz).

ν_{max} (neat) / cm^{-1}: 2948, 1727, 1653, 1509, 1371, 1219, 1162, 846.

HRMS: calcd. for $C_{28}H_{26}FN_2O_3$ [M-Cl]$^+$: 457.1922; found (ESI$^+$): 457.1955.

m.p. / °C: 195 (decomp.).

(*S*)-4-(2-Acetamido-3-((2-methoxy-2-oxoethyl)amino)-3-oxopropyl)-1-benzyl-3-phenylquinolin-1-ium chloride (24)

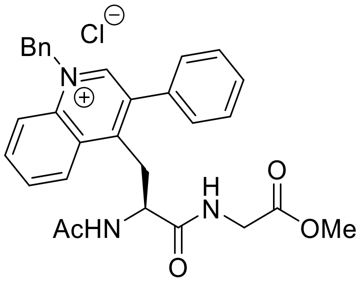

Synthesised according to modified **GP-1** from methyl *N*α-acetyl-1-benzyl-*L*-tryptophylglycinate (82 mg, 0.2 mmol) and 3-chloro-3-phenyldiazirine (92 mg, 0.6 mmol) in CH_2Cl_2 (2 mL). The solvent was removed *in vacuo* and the crude material purified by column chromatography (C_{18}, MeCN) to afford the product as an orange solid (22.5 mg, 0.041 mmol, 21%).

^{1}H NMR (400 MHz, CD$_3$OD): δ 9.51 (s, 1H), 8.83 (dd, *J* = 8.6, 1.3 Hz, 1H), 8.48 (d, *J* = 8.8 Hz, 1H), 8.18 (ddd, *J* = 8.8, 6.9, 1.3 Hz, 1H), 8.13 – 8.03 (m, 1H), 7.68 – 7.60 (m, 5H), 7.47 – 7.36 (m, 5H), 6.38 (s, 2H), 4.80 (dd, *J* = 8.5, 6.0 Hz, 1H), 4.08 (dd, *J* = 13.6, 6.1 Hz, 1H), 3.93 – 3.75 (m, 3H), 3.71 (s, 3H), 1.69 (s, 3H).

^{13}C{^{1}H} NMR (101 MHz, CD$_3$OD): δ 172.9, 171.62, 171.58, 157.6, 150.6, 138.6, 138.4, 136.2, 135.8, 134.8, 131.5, 131.4, 131.2, 130.6, 130.5, 130.4, 130.2, 129.0, 128.1, 120.9, 62.0, 54.8, 52.7, 41.8, 33.5, 22.4.

HRMS: calcd. for $C_{30}H_{30}N_3O_4$ [M-Cl]$^+$: 496.2231; found (ESI$^+$): 496.2241.

1-Benzyl-6-fluoro-3-(4-methylphenyl)quinolin-1-ium chloride (25)

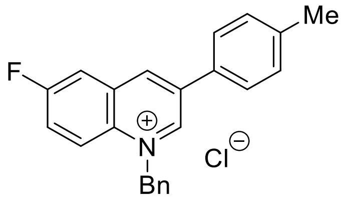

Synthesised according to **GP-1** from *N*-benzyl-5-fluoroindole (45 mg, 0.2 mmol) and 3-chloro-3-(4-methylphenyl)diazirine (112 mg, 0.6 mmol) in 1:1 CH_2Cl_2/PhMe (2 mL) as a tan solid (40.0 mg, 0.110 mmol, 55%).

^{1}H NMR (400 MHz, CD_3OD): δ 9.99 (d, *J* = 2.2 Hz, 1H), 9.50 (d, *J* = 2.2 Hz, 1H), 8.55 (dd, *J* = 9.7, 4.4 Hz, 1H), 8.21 (dd, *J* = 8.2, 3.0 Hz, 1H), 7.99 (ddd, *J* = 10.1, 7.9, 3.0 Hz, 1H), 7.95 – 7.86 (m, 2H), 7.56 – 7.47 (m, 2H), 7.42 (m, 5H), 6.44 (s, 2H), 2.49 (s, 3H).

^{13}C{^{1}H} NMR (101 MHz, CD_3OD): δ 163.4 (d, *J* = 254.8 Hz), 149.8, 144.6 (d, *J* = 5.3 Hz), 142.2, 137.6, 135.4, 134.5, 133.8 (d, *J* = 11.0 Hz), 131.8, 131.6, 130.6, 130.4, 128.6, 128.2, 126.3 (d, *J* = 27.0 Hz), 123.6 (d, *J* = 9.5 Hz), 115.3 (d, *J* = 23.2 Hz), 62.9, 21.3.

^{19}F NMR (377 MHz, CD_3OD) δ -108.51 (app td, *J* = 8.2, 4.4 Hz).

ν_{max} (neat) / cm^{-1}: 3030, 2934, 1632, 1535, 1384, 1194, 834, 816.

HRMS: calcd. for $C_{23}H_{19}FN$ [M-Cl]$^+$: 328.1496; found (ESI$^+$): 328.1506.

m.p. / °C: 231-234.

1-Benzyl-6-fluoro-3-(4-fluorophenyl)quinolin-1-ium chloride (26)

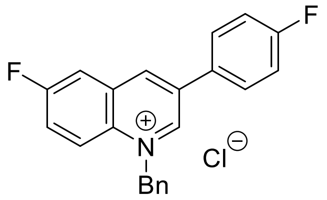

Synthesised according to **GP-1** from *N*-benzyl-5-fluoroindole (45 mg, 0.2 mmol) and 3-chloro-3-(4-fluorophenyl)diazirine (102 mg, 0.6 mmol) in 1:1 CH_2Cl_2/PhMe (2 mL) as an off-white solid (52.9 mg, 0.144 mmol, 72%).

^{1}H NMR (400 MHz, CD_3OD): δ 10.02 (d, *J* = 2.0 Hz, 1H), 9.52 (d, *J* = 2.0 Hz, 1H), 8.56 (dd, *J* = 9.7, 4.4 Hz, 1H), 8.23 (dd, *J* = 8.2, 2.9 Hz, 1H), 8.12 – 7.90 (m, 3H), 7.43 (m, 7H), 6.46 (s, 2H)**.**

^{13}C{^{1}H} NMR (101 MHz, CD_3OD): δ 165.7 (d, *J* = 250.4 Hz), 163.4 (d, *J* = 254.5 Hz), 149.9, 145.1 (d, *J* = 5.1 Hz), 136.6, 135.5, 134.5, 133.7 (d, *J* = 10.8 Hz), 131.2 (d, *J* = 8.7 Hz), 131.1, 130.6, 130.5, 128.2, 126.6 (d, *J* = 27.1 Hz), 123.6 (d, *J* = 9.5 Hz), 117.9 (d, *J* = 22.3 Hz), 115.4 (d, *J* = 23.4 Hz), 63.0.

^{19}F NMR (376 MHz, CD_3OD): -108.26 (app td, *J* = 8.2, 4.5 Hz), -112.71 (tt, *J* = 8.7, 4.4 Hz).

ν_{max} (neat) / cm^{-1}: 3004, 2950, 1601, 1513, 1492, 1384, 1245, 1203, 1166, 842.

HRMS: calcd. for $C_{22}H_{16}F_2N$ [M-Cl]$^+$: 332.1245; found (ESI$^+$): 332.1261.

m.p. / °C: 226-229.

1-Benzyl-6-fluoro-3-(4-bromophenyl)quinolin-1-ium chloride (27)

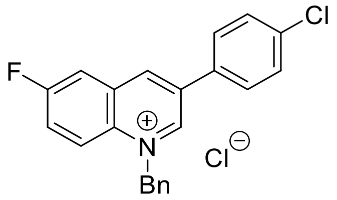

Synthesised according to **GP-1** from *N*-benzyl-5-fluoroindole (45 mg, 0.2 mmol) and 3-chloro-3-(4-chlorophenyl)diazirine (112 mg, 0.6 mmol) in 1:1 CH_2Cl_2/PhMe (2 mL) as a pale yellow solid (38.9 mg, 0.101 mmol, 51%).

^{1}H NMR (400 MHz, CD_3OD): δ 10.03 (d, *J* = 2.0 Hz, 1H), 9.55 (d, *J* = 2.0 Hz, 1H), 8.56 (dd, *J* = 9.8, 4.2 Hz, 1H), 8.23 (dd, *J* = 8.1, 2.9 Hz, 1H), 8.05 – 7.98 (m, 3H), 7.71 (d, *J* = 8.6 Hz, 2H), 7.48 – 7.39 (m, 5H), 6.46 (s, 2H).

^{13}C{^{1}H} NMR (101 MHz, CD_3OD): δ 163.4 (d, *J* = 255.0 Hz), 149.9, 145.3 (d, *J* = 5.3 Hz), 137.8, 136.4, 135.6, 134.5, 133.7 (d, *J* = 11.1 Hz), 133.4, 131.0, 130.6, 130.4, 128.2, 126.9, 126.6, 123.6 (d, *J* = 9.5 Hz), 115.5 (d, *J* = 23.3 Hz), 63.0.

^{19}F NMR (376 MHz, CD_3OD): δ -108.19 (app td, *J* = 8.1, 4.2 Hz).

ν_{max} (neat) / cm^{-1}: 3027, 2948, 1632, 1536, 1498, 1454, 1384, 1350, 1278, 1213, 1091, 1036, 1010.

HRMS: calcd. for $C_{22}H_{16}FNCl$ [M-Cl]$^+$: 348.0950; found (ESI$^+$): 348.0961.

m.p. / °C: 238-241.

1-Benzyl-6-fluoro-3-(4-bromophenyl)quinolin-1-ium chloride (28)

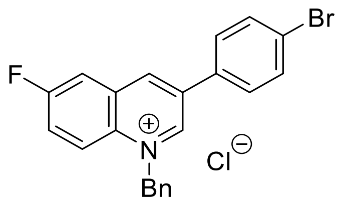

Synthesised according to **GP-1** from *N*-benzyl-5-fluoroindole (45 mg, 0.2 mmol) and 3-chloro-3-(4-bromophenyl)diazirine (139 mg, 0.6 mmol) in 1:1 CH_2Cl_2/PhMe (2 mL) as a pale yellow solid (76.1 mg, 0.163 mmol, 84%).

^{1}H NMR (400 MHz, CD_3OD): δ 10.03 (d, *J* = 2.0 Hz, 1H), 9.55 (d, *J* = 2.0 Hz, 1H), 8.56 (dd, *J* = 9.7, 4.4 Hz, 1H), 8.23 (dd, *J* = 8.1, 2.9 Hz, 1H), 8.02 (ddd, *J* = 9.7, 8.1, 2.9 Hz, 1H), 7.97 – 7.90 (m, 2H), 7.90 – 7.81 (m, 2H), 7.51 – 7.36 (m, 5H), 6.45 (s, 2H).

^{13}C{^{1}H} NMR (101 MHz, CD_3OD): δ 163.4 (d, *J* = 255.1 Hz), 149.9, 145.3, 136.4, 135.7, 134.4, 134.1, 133.9, 133.7, 133.6, 130.6 (d, *J* = 5.1 Hz), 130.5, 128.2, 126.8 (d, *J* = 27.0 Hz), 126.0, 123.6 (d, *J* = 9.6 Hz), 115.5 (d, *J* = 23.4 Hz), 63.0.

^{19}F NMR (376 MHz, CD_3OD): δ -108.16 (app td, *J* = 8.1, 4.4 Hz).

ν_{max} (neat) / cm^{-1}: 2947, 1631, 1535, 1491, 1454, 1349, 1278, 1213, 1075, 1004, 827.

HRMS: calcd. for $C_{22}H_{16}FNBr$ [M-Cl]$^+$: 392.0445; found (ESI$^+$): 392.0439.

m.p. / °C: 239-243.

1-Benzyl-6-fluoro-3-(4-nitrophenyl)quinolin-1-ium chloride (29)

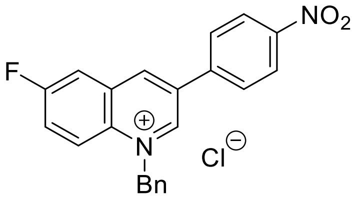

Synthesised according to **GP-1** from *N*-benzyl-5-fluoroindole (45 mg, 0.2 mmol) and 3-chloro-3-(4-nitrophenyl)diazirine (119 mg, 0.6 mmol) in 1:1 CH_2Cl_2/PhMe (2 mL) as a brown solid (35.9 mg, 0.091 mmol, 45%).

^{1}H NMR (400 MHz, CD_3OD): δ 10.13 (d, *J* = 1.8 Hz, 1H), 9.68 (d, *J* = 1.8 Hz, 1H), 8.60 (dd, *J* = 9.7, 4.2 Hz, 1H), 8.58 – 8.52 (m, 2H), 8.35 – 8.25 (m, 3H), 8.07 (ddd, *J* = 9.7, 7.9, 2.9 Hz, 1H), 7.51 – 7.37 (m, 5H), 6.49 (s, 2H).

^{13}C{^{1}H} NMR (101 MHz, CD_3OD): δ 163.5 (d, *J* = 255.4 Hz), 150.3, 150.2, 146.5 (d, *J* = 5.2 Hz), 140.9, 136.0, 135.3, 134.4, 133.6 (d, *J* = 11.1 Hz), 130.7, 130.5, 130.2, 128.2, 127.4 (d, *J* = 26.9 Hz), 125.7, 123.7 (d, *J* = 9.6 Hz), 115.8 (d, *J* = 23.4 Hz), 63.2.

^{19}F NMR (376 MHz, CD_3OD): δ -107.76 (app td, *J* = 7.9, 4.2 Hz).

ν_{max} (neat) / cm^{-1}: 3084, 2947, 1518, 1345, 830.

HRMS: calcd. for $C_{22}H_{16}FN_2O_2$ [M-Cl]$^+$: 359.1190; found (ESI$^+$): 359.1202.

m.p. / °C: 228-231.

1-Benzyl-6-fluoro-3-(3-bromophenyl)quinolin-1-ium chloride (30)

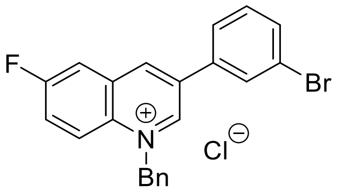

Synthesised according to **GP-1** from *N*-benzyl-5-fluoroindole (45 mg, 0.2 mmol) and 3-chloro-3-(3-bromophenyl)diazirine (139 mg, 0.6 mmol) in 1:1 CH_2Cl_2/PhMe (2 mL) as a brown solid (38.8 mg, 0.090 mmol, 45%).

^{1}H NMR (400 MHz, CD_3OD): δ 10.06 (d, *J* = 2.0 Hz, 1H), 9.57 (d, *J* = 2.0 Hz, 1H), 8.57 (dd, *J* = 9.7, 4.3 Hz, 1H), 8.26 – 8.22 (m, 2H), 8.04 (dd, *J* = 8.0, 2.9 Hz, 1H), 7.99 (dd, J = 8.7, 2.5 Hz, 1H), 7.81 (ddd, *J* = 8.0, 2.0, 0.9 Hz, 1H), 7.61 (app t, *J* = 8.0 Hz, 1H), 7.48 – 7.39 (m, 5H), 6.46 (s, 2H).

^{13}C{^{1}H} NMR (101 MHz, CD_3OD): δ 163.4 (d, *J* = 255.0 Hz), 150.1, 145.8, 145.7 (d, *J* = 5.4 Hz), 137.0, 136.0, 134.5, 134.4, 133.6 (d, *J* = 10.8 Hz), 132.6, 131.8, 130.6, 130.4, 128.2, 127.7, 126.9 (d, *J* = 27.0 Hz), 124.7, 123.6 (d, *J* = 9.6 Hz), 115.6 (d, *J* = 23.2 Hz), 63.1.

^{19}F NMR (376 MHz, CD_3OD): δ -108.15 (app td, J = 7.9, 4.3 Hz).

ν_{max} (neat) / cm^{-1}: 3000, 2933, 1630, 1566, 1535, 1487, 1388, 1275, 1197, 835.

HRMS: calcd. for $C_{22}H_{16}FNBr$ [M-Cl]$^+$: 392.0445; found (ESI$^+$): 392.0446.

m.p. / °C: 196-199.

1-Benzyl-6-fluoro-3-(2-(trifluoromethoxy)phenyl)quinolin-1-ium chloride (31)

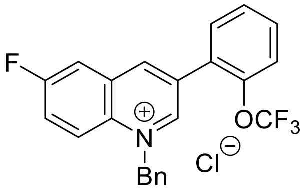

Synthesised according to **GP-1** from *N*-benzyl-5-fluoroindole (45 mg, 0.2 mmol) and 3-chloro-3-(2-trifluoromethoxy)diazirine (142 mg, 0.6 mmol) in 1:1 CH_2Cl_2/PhMe (2 mL) as an orange solid (30.0 mg, 0.072 mmol, 36%).

^{1}H NMR (400 MHz, CD_3OD): δ 9.86 (d, *J* = 1.9 Hz, 1H), 9.45 (d, *J* = 1.9 Hz, 1H), 8.69 (dd, *J* = 9.7, 4.3 Hz, 1H), 8.28 (dd, *J* = 8.0, 2.9 Hz, 1H), 8.11 (ddd, *J* = 9.7, 8.0, 2.9 Hz, 1H), 7.88 (dd, *J* = 7.6, 1.8 Hz, 1H), 7.77 (td, *J* = 7.8, 1.8 Hz, 1H), 7.72 – 7.60 (m, 2H), 7.51 – 7.38 (m, 5H), 6.45 (s, 2H).

^{13}C{^{1}H} NMR (101 MHz, CD_3OD): δ 163.5 (d, *J* = 255.6 Hz), 151.0 (d, *J* = 1.9 Hz), 148.3 (d, *J* = 5.2 Hz), 147.5 (q, *J* = 1.7 Hz), 135.8, 134.1, 133.45 (d, *J* = 11.0 Hz), 133.32, 133.30 (d, *J* = 15.2 Hz), 130.70, 130.68, 129.6, 128.8, 128.6, 127.4 (d, *J* = 26.9 Hz), 123.7 (d, *J* = 9.5 Hz), 122.7 (d, *J* = 1.7 Hz), 121.7 (q, *J* = 258.0 Hz), 115.6 (d, *J* = 23.3 Hz), 62.8

^{19}F NMR (376 MHz, CD_3OD): δ -58.80 (s, 3F), -107.67 (app td, *J* = 7.9, 4.3 Hz, 1F).

ν_{max} (neat) / cm^{-1}: 2949, 1531, 1252, 1220, 1202, 1160, 1035.

HRMS: calcd. for $C_{23}H_{16}F_4NO$ $[M-Cl]^+$ 398.1163; found (ESI^+): 398.1158.

m.p. / °C: 162-164.

1-Benzyl-6-fluoro-3-(3-bromophenyl)quinolin-1-ium chloride (32)

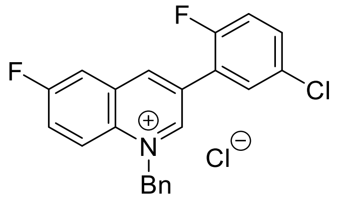

Synthesised according to **GP-1** from *N*-benzyl-5-fluoroindole (45 mg, 0.2 mmol) and 3-chloro-3-(5-chloro-2-fluorophenyl)diazirine (123 mg, 0.6 mmol) in 1:1 CH_2Cl_2/PhMe (2 mL) as an orange solid (45.3 mg, 0.113 mmol, 56%).

^{1}H NMR (400 MHz, CD_3OD): δ 9.93 (s, 1H), 9.53 (s, 1H), 8.63 (dd, *J* = 9.8, 4.3 Hz, 1H), 8.27 (dd, *J* = 8.0, 2.9 Hz, 1H), 8.09 (ddd, *J* = 9.8, 8.0, 2.9 Hz, 1H), 7.98 (dd, *J* = 6.7, 2.7 Hz, 1H), 7.68 (ddd, *J* = 8.9, 4.3, 2.7 Hz, 1H), 7.51 – 7.38 (m, 6H), 6.45 (s, 2H).

^{13}C{^{1}H} NMR (101 MHz, CD_3OD): δ 163.5 (d, *J* = 255.4 Hz), 159.8 (d, *J* = 249.4 Hz), 150.8, 148.1, 135.9, 134.2, 133.5 (d, *J* = 8.9 Hz), 133.5 (d, *J* = 11.2 Hz), 131.9, 131.8 (d, *J* = 2.4 Hz), 131.1, 130.7, 130.6, 128.5, 127.4 (d, *J* = 27.0 Hz), 124.5 (d, *J* = 14.5 Hz), 123.7 (d, *J* = 9.6 Hz), 119.4 (d, *J* = 24.2 Hz), 115.8 (d, *J* = 23.4 Hz), 63.0.

^{19}F NMR (376 MHz, CD_3OD): δ -107.82 (app td, *J* = 8.0, 4.3 Hz, 1F), -122.17 (app dt, *J* = 10.8, 5.7 Hz, 1F).

ν_{max} (neat) / cm^{-1}: 3047, 3009, 2936, 1633, 1535, 1491, 1387, 1271, 1213, 965, 809.

HRMS: calcd. for $C_{22}H_{15}NF_2Cl$ [M-Cl]$^+$: 366.0856; found (ESI$^+$): 366.0853.

m.p. / °C: 216-219.

1-benzyl-6-fluoro-3-(pyridin-2-yl)quinolin-1-ium chloride (33)

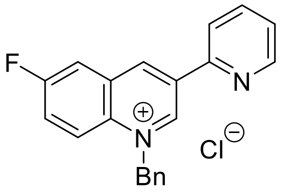

Synthesised according to **GP-1** from *N*-benzyl-5-fluoroindole (45 mg, 0.2 mmol) and 3-chloro-3-(2-pyridyl)diazirine (154 mg, 1.0 mmol) in 1:1 CH_2Cl_2/PhMe (2 mL) as a brown solid (30.7 mg, 0.091 mmol, 45%).

^{1}H NMR (400 MHz, CD_3OD): δ 10.34 (d, *J* = 1.9 Hz, 1H), 9.86 (d, *J* = 1.9 Hz, 1H), 8.83 (ddd, *J* = 4.9, 1.9, 1.0 Hz, 1H), 8.60 (dd, *J* = 9.7, 4.3 Hz, 1H), 8.34 (app dt, *J* = 7.9, 1.0 Hz, 1H), 8.27 (dd, *J* = 8.0, 2.8 Hz, 1H), 8.10 (app td, *J* = 7.9, 1.8 Hz, 1H), 8.04 (ddd, *J* = 9.7, 7.9, 2.9 Hz, 1H), 7.59 (ddd, *J* = 7.9, 4.9, 1.0 Hz, 1H), 7.48 – 7.38 (m, 5H), 6.48 (s, 2H).

^{13}C{^{1}H} NMR (101 MHz, CD_3OD): δ 163.4 (d, *J* = 255.2 Hz), 151.6, 151.5, 149.9, 144.9 (d, *J* = 5.1 Hz), 139.5, 136.3, 135.6, 134.3, 133.5 (d, *J* = 11.0 Hz), 130.7, 130.5, 128.6, 127.0 (d, *J* = 26.8 Hz), 126.3, 123.7 (d, *J* = 9.5 Hz), 122.9, 115.9 (d, *J* = 23.4 Hz), 63.0.

^{19}F NMR (376 MHz, CD_3OD): δ -108.25 (app td, *J* = 8.0, 4.3 Hz).

ν_{max} (neat) / cm^{-1}: 3002, 2946, 1526, 1384, 1196, 1147.

HRMS: calcd. for $C_{21}H_{16}N_2F$ [M-Cl]$^+$: 315.1292; found (ESI$^+$) 315.1295.

m.p. / °C: 210 (decomp.).

2.2. General Procedure 2 (GP-2): Ring Expansion of *N*-Alkylpyrroles and *N*-Alkylpyrazoles

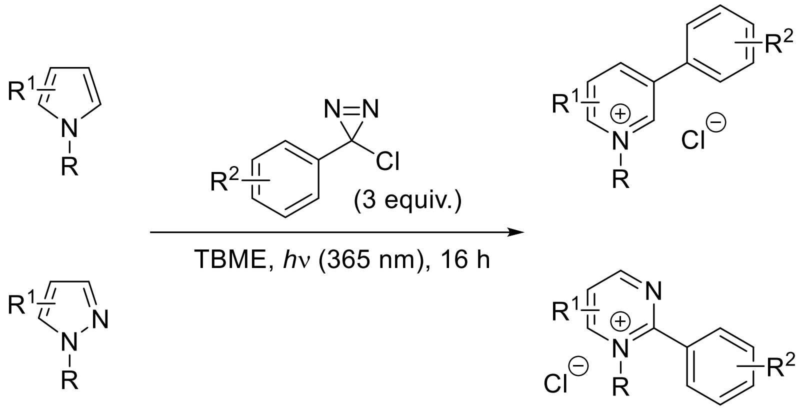

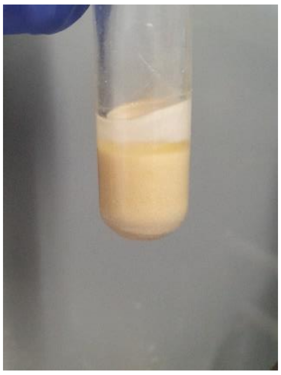

A 10 mL microwave tube was charged with *N*-alkylpyrrole or *N*-alkylpyrazole (0.2 mmol) which was then sealed with a crimp-cap fitted with a PTFE-faced silicone septum. The tube was evacuated and flushed with dinitrogen 3 times, then anhydrous TBME (2 mL) was added, followed by 3-chloro-3-aryldiazirine (0.6 mmol). The cap of the reaction flask was then sealed with electrical tape. The reaction mixture was stirred under constant irradiation with UV light (365 nm, 18 W LED, 5 cm from light source) overnight. The resulting precipitate was isolated by filtration, washed with TBME (2 × 5 mL), and dried under a flow of air to afford the pure product. For products that did not present as free-flowing solids, the isolated filtrand was dissolved from the sinter into a second pre-weighed flask with MeOH or CH_2Cl_2. The resulting solution was concentrated *in vacuo* to afford the pure product.

1-Benzyl-3-phenylpyridinium chloride (34)

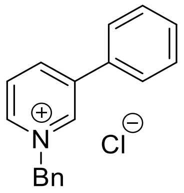

Synthesised according to **GP-2** from *N*-benzylpyrrole (31 mg, 0.2 mmol) and 3-chloro-3-phenyldiazirine (92 mg, 0.6 mmol) in TBME (2 mL) as a viscous gum (36.5 mg, 0.130 mmol, 65%).

^{1}H NMR (400 MHz, CD_3OD): δ 9.47 (s, 1H), 8.98 (d, *J* = 6.0 Hz, 1H), 8.89 (d, *J* = 8.1 Hz, 1H), 8.27 – 8.16 (m, 1H), 7.85 – 7.82 (m, 2H), 7.64 – 7.50 (m, 8H), 5.93 (s, 2H).

^{13}C{^{1}H} NMR (101 MHz, CD_3OD): δ 143.4, 142.6, 142.4, 141.8, 133.3, 133.2, 130.3, 129.7, 129.5, 129.4, 128.7, 128.3, 127.2, 64.5.

ν_{max} (neat) / cm^{-1}: 3025, 2984, 1678, 1488, 1433, 1153.

HRMS: calcd. for $C_{18}H_{16}N$ [M-Cl]$^+$: 246.1277; found (ESI$^+$): 246.1273.

1,3-Diphenylpyridin-1-ium chloride (35)

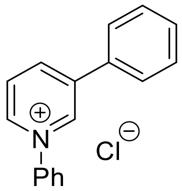

Synthesised according to **GP-2** from 1-phenylpyrrole (29 mg, 0.2 mmol) and 3-chloro-3-phenyldiazirine (92 mg, 0.6 mmol) in TBME (2 mL) as a yellow solid (50.2 mg, 0.196 mmol, 98%).

^{1}H NMR (400 MHz, CD_3OD): δ 9.51 (app t, *J* = 1.7 Hz, 1H), 9.18 (app dt, *J* = 6.2, 1.4 Hz, 1H), 9.04 (app dt, *J* = 8.3, 1.4 Hz, 1H), 8.32 (dd, *J* = 8.3, 6.1 Hz, 1H), 7.91 (ddd, *J* = 7.7, 4.4, 2.3 Hz, 4H), 7.81 – 7.75 (m, 3H), 7.66 – 7.57 (m, 3H).

^{13}C{^{1}H} NMR (101 MHz, CD_3OD): δ 145.4, 144.2, 144.1, 143.2, 134.6, 132.8, 131.71, 131.67, 130.9, 129.4, 128.9, 125.8.

ν_{max} (neat) / cm^{-1}: 3025, 1573, 1482, 1413, 1309, 1229, 1024.

HRMS: calcd. for $C_{17}H_{14}N$ [M-Cl]$^+$: 232.1121; found (ESI$^+$): 232.1125.

m.p. / °C: 100-103.

1-Benzyl-2,6-dimethyl-3-phenylpyridin-1-ium chloride (36)

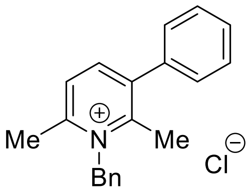

Synthesised according to **GP-2** from *N*-benzyl-2,5-dimethylpyrrole (38 mg, 0.2 mmol) and 3-chloro-3-phenyldiazirine (92 mg, 0.6 mmol) in TBME (2 mL) as a viscous gum (55.0 mg, 0.189 mmol, 88%).

^{1}H NMR (400 MHz, DMSO-d$_6$): δ 8.48 (d, *J* = 8.1 Hz, 1H), 8.09 (d, *J* = 8.2 Hz, 1H), 7.62 – 7.54 (m, 3H), 7.52 – 7.48 (m, 2H), 7.48 – 7.37 (m, 3H), 7.17 – 7.11 (m, 2H), 6.01 (s, 2H), 2.81 (s, 3H), 2.63 (s, 3H).

^{13}C{^{1}H} NMR (101 MHz, DMSO-d$_6$): δ 155.9, 154.8, 146.2, 140.5, 136.7, 132.7, 129.8, 129.6, 129.4, 128.7, 127.9, 126.0, 56.8, 21.6, 19.7.

ν_{max} (neat) / cm^{-1}: 2971, 2818, 1615, 1481, 1447, 1029.

HRMS: calcd. for $C_{20}H_{20}N$ [M-Cl]$^+$: 274.1590; found (ESI$^+$): 274.1597.

2-Benzyl-8-oxo-4-phenyl-5,6,7,8-tetrahydroisoquinolin-2-ium chloride and 2-Benzyl-5-oxo-4-phenyl-5,6,7,8-tetrahydroisoquinolin-2-ium chloride (37)

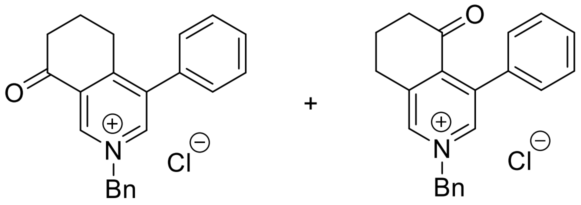

Synthesised according to **GP-2** from 2-benzyl-2,5,6,7-tetrahydro-4H-isoindol-4-one (45 mg, 0.2 mmol) and 3-chloro-3-phenyldiazirine (92 mg, 0.6 mmol) in TBME (2 mL) as a viscous gum (43.9 mg, 0.125 mmol, 63%, 11:1 mixture of isomers).

^{1}H NMR (400 MHz, DMSO-d$_6$): δ 9.56[a] (d, *J* = 1.5 Hz, 1H), 9.46[b] (d, *J* = 1.4 Hz, 0.1H), 9.40[a] (d, *J* = 1.5 Hz, 1H), 9.26[b] (d, *J* = 1.4 Hz, 0.1H), 7.68 – 7.59[a] (m, 5H), 7.57 – 7.53[a] (m, 2H), 7.50 – 7.41[a,b] (m, 4H), 7.39 – 7.33[b] (m, 0.4H), 7.33 – 7.25[b] (m, 0.5H), 6.68[b] (d, *J* = 2.1 Hz, 0.1H), 5.95[a] (s, 2H), 5.87[b] (s, 0.2H), 3.08[a] (t, *J* = 6.0 Hz, 2H), 2.79[a] (t, *J* = 6.5 Hz, 2H), 2.58[b] (t, *J* = 6.1 Hz, 0.2H), 2.29[b] (t, *J* = 7.1 Hz, 0.2H), 2.13 – 2.03[a] (m, 2H), 1.91[b] (app p, *J* = 6.3 Hz, 0.2H).

^{13}C{^{1}H} NMR (101 MHz, DMSO-d$_6$): δ 194.3[a], 193.6[b], 160.8[a], 145.2[a], 141.9[a], 141.1, 140.0, 137.8[b], 134.3, 132.6, 131.5, 129.8[a], 129.5[a], 129.4[a], 129.2[a], 129.2, 129.0[a], 128.9[a], 128.6[b], 128.3[b], 127.8[b], 127.7[b], 126.4, 122.0, 117.3[b], 63.5[b] 63.0[a], 52.6, 37.3[a], 27.9[a], 26.6, 24.8, 21.1, 20.8[a].

ν_{max} (neat) / cm^{-1}: 2932, 1702, 1627, 1160, 1029, 905.

HRMS: calcd. for $C_{22}H_{20}NO$ [M-Cl]$^{+}$: 314.1539; found (ESI^{+}): 314.1546.

Peak assignment for regioisomers determined by relative integrations and 2D multinuclear correlation spectroscopy. Ambiguous peaks have been left unassigned.

1-Benzyl-3-(methoxycarbonyl)-4,5-diphenylpyridin-1-ium chloride and 1-benzyl-4-(methoxycarbonyl)-3,5-diphenylpyridin-1-ium chloride (38)

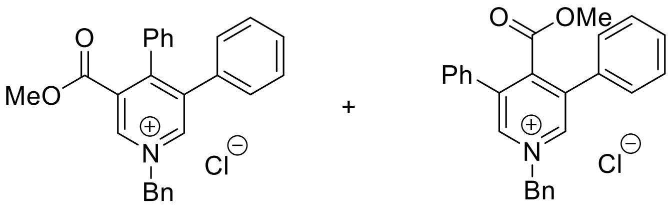

Synthesised according to **GP-2** from *N*-benzyl-3-phenyl-4-(carboxymethyl)pyrrole (58 mg, 0.2 mmol) and 3-chloro-3-phenyldiazirine (92 mg, 0.6 mmol) in TBME (2 mL) as a tan solid (58.5 mg, 0.141 mmol, 70%, 3.3:1 mixture of isomers).

^{1}H NMR (400 MHz, DMSO-d$_6$): δ 9.70[a] (d, *J* = 2.1 Hz, 1H), 9.65[b] (d, *J* = 2.0 Hz, 0.6H), 9.63[a] (d, *J* = 1.7 Hz, 1H), 7.75 – 7.71[a] (m, 3H), 7.62 (dd, *J* = 7.3, 3.3 Hz, 2H), 7.54 – 7.45[a] (m, 6H), 7.40 – 7.28 (m, 8H), 7.23 – 7.19 (m, 2H), 7.17 – 7.12 (m, 2H), 7.08 (d, *J* = 2.5 Hz, 0.3H), 6.00[a] (s, 2H), 3.65[a] (s, 3H), 3.53[b] (s, 0.9H).

^{13}C{^{1}H} NMR (101 MHz, DMSO-d$_6$): δ 164.8[b], 164.1, 163.4[a], 155.1, 146.5[a], 144.8[b], 144.4, 143.2[a], 141.5, 138.4, 137.7, 134.5, 133.9, 133.7, 133.2, 132.5, 132.1, 130.1, 129.9, 129.5, 129.4, 129.4, 129.3, 129.2, 129.2, 129.2, 129.0, 128.8, 128.7, 128.58, 128.57, 128.4, 128.2, 127.8, 127.7, 127.6, 126.0, 125.8, 122.1, 111.6, 63.7[b], 63.2[a], 53.3[a], 52.6, 50.5.

ν_{max} (neat) / cm^{-1}: 2947, 1737, 1627, 1432, 1330, 1275, 1219, 1167, 1100.

HRMS: calcd. for $C_{26}H_{22}NO_2$ [M-Cl]$^+$: 380.1645; found (ESI$^+$): 380.1640.

Peak assignment for regioisomers determined by relative integrations and 2D multinuclear correlation spectroscopy. Ambiguous peaks have been left unassigned.

1-Benzyl-2-isopropyl-5-phenylpyridin-1-ium chloride and 1-benzyl-2-isopropyl-3-phenylpyridin-1-ium chloride (39)

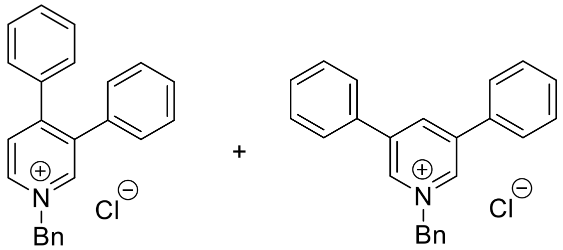

Synthesised according to **GP-2** from *N*-benzyl-3-phenylpyrrole (49 mg, 0.2 mmol) and 3-chloro-3-phenyldiazirine (92 mg, 0.6 mmol) in TBME (2 mL) as a tan solid (58.3 mg, 0.163 mmol, 81%, 6.0:1 mixture of isomers).

^{1}H NMR (400 MHz, CD_3OD): δ 9.77[b] (d, *J* = 1.7 Hz, 0.3H), 9.50[a] (d, *J* = 1.5 Hz, 1H), 9.29[a] (dd, *J* = 6.4, 1.5 Hz, 1H), 9.17[b] (app t, *J* = 1.7 Hz, 0.15H), 8.25[a] (d, *J* = 6.4 Hz, 1H), 8.08 – 8.02[b] (m, 0.75H), 7.80 – 7.74[b] (m, 0.3H), 7.74 – 7.69[a] (m, 2H), 7.67 – 7.56[b] (m, 1H), 7.54 – 7.36[a,b] (m, 10H), 7.33 – 7.23[a,b] (m, 5H), 6.05[b] (s, 0.3H), 5.96[a] (s, 2H).

^{13}C{^{1}H} NMR (101 MHz, CD_3OD): δ 155.5[a], 145.2[a], 142.8[a], 141.0[b], 140.4, 140.2[b], 139.3, 135.2, 134.6, 134.4, 133.9[a], 133.1[b], 130.3, 130.0, 129.8, 129.41, 129.37, 129.23, 129.17, 129.1, 129.07, 129.05, 128.9, 128.8, 128.7, 128.5, 127.9[b], 63.5[b], 62.6[a].

ν_{max} (neat) / cm^{-1}: 2933, 2819, 1630, 1496, 1439, 1153, 1029.

HRMS: calcd. for $C_{24}H_{20}N$ [M-Cl]$^+$: 322.1590; found (ESI$^+$): 322.1599.

Peak assignment for regioisomers determined by relative integrations and 2D multinuclear correlation spectroscopy. Ambiguous peaks have been left unassigned.

1-Benzyl-2-methyl-5-phenylpyridin-1-ium chloride and 1-benzyl-2-methyl-3-phenylpyridin-1-ium chloride (40)

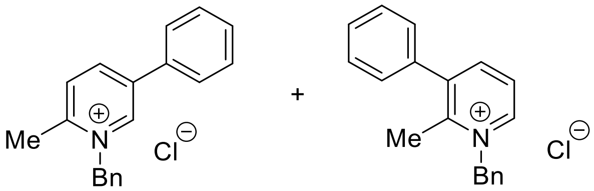

Synthesised according to **GP-2** from *N*-benzyl-2-methylpyrrole (34 mg, 0.2 mmol) and 3-chloro-3-phenyldiazirine (92 mg, 0.6 mmol) in TBME (2 mL) as a tan solid (26.7 mg, 0.090 mmol, 45%, 3.7:1 mixture of isomers).

^{1}H NMR (400 MHz, CD_3OD): δ 9.65[a] (d, *J* = 2.1 Hz, 1H), 9.14[b] (d, *J* = 7.2 Hz, 0.25H), 8.94[a] (dd, *J* = 8.3, 2.1 Hz, 1H), 8.54[b] (d, *J* = 6.8 Hz, 0.25H), 8.18[a] (d, *J* = 8.4 Hz, 1H), 8.13[b] (d, *J* = 7.7 Hz, 0.25H), 7.97 – 7.91 (m, 2H), 7.68 – 7.55 (m, 3H), 7.52 – 7.40 (m, 3H), 7.37 – 7.30 (m, 3H), 6.00[b] (s, 0.5H), 5.98[a] (s, 2H), 2.74 (s, 3H), 2.62 (s, 0.75H).

^{13}C{^{1}H} NMR (101 MHz, CD_3OD): δ 153.9[a], 153.8[b], 146.1[b], 145.5[b], 144.1[a], 143.7, 143.1[a], 142.3, 141.7, 137.6[a], 133.2, 132.9, 132.6, 130.5, 130.0, 129.5, 129.3, 129.3, 129.2, 128.9, 128.8, 128.7, 127.6, 127.4, 127.33, 125.3[b], 61.1[b], 60.7[a], 19.5[a], 18.3[b].

ν_{max} (neat) / cm^{-1}: 3064, 2964, 2820, 1628, 1538, 1493, 1476, 1454, 1028.

HRMS: calcd. for $C_{26}H_{22}NO_2$ [M-Cl]$^+$: 380.1645; found (ESI$^+$): 380.1640.

Peak assignment for regioisomers determined by relative integrations and 2D multinuclear correlation spectroscopy. Ambiguous peaks have been left unassigned.

1-benzyl-2-isopropyl-5-phenylpyridin-1-ium chloride and 1-benzyl-2-isopropyl-3-phenylpyridin-1-ium chloride

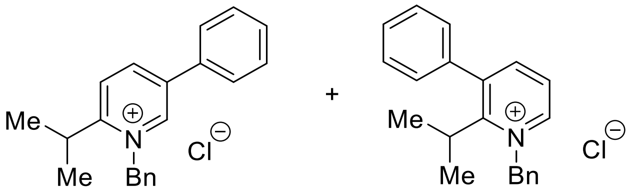

Synthesised according to **GP-2** from *N*-benzyl-2-isopropylpyrrole (40 mg, 0.2 mmol) and 3-chloro-3-phenyldiazirine (92 mg, 0.6 mmol) in TBME (2 mL) as a tan solid (47.9 mg, 0.148 mmol, 74%, 1.2:1 mixture of isomers).

^{1}H NMR (400 MHz, CD_3OD): δ 9.28^{a} (app t, J = 1.6 Hz, 1H), 9.00^{a} (d, J = 1.6 Hz, 1H), 8.98 – 8.92^{a,b} (m, 1.8H), 8.81 – 8.76^{a} (m, 1H), 8.18^{b} (d, J = 6.4 Hz, 0.8H), 7.87 – 7.81^{a} (m, 2H), 7.67 – 7.54 (m, 9H), 7.54 – 7.43 (m, 8H), 5.93^{a} (s, 2H), 5.83^{b} (s, 1.6H), 3.31 – 3.26^{a,b} (m, 1.8H), 1.44^{a} (d, J = 7.0 Hz, 6H), 1.28^{b} (d, J = 6.8 Hz, 5H).

^{13}C{^{1}H} NMR (101 MHz, CD_3OD): δ 168.6^{a}, 151.7^{a}, 145.2^{b}, 144.0^{b}, 143.1^{a}, 143.0, 142.9, 142.3^{a}, 141.7^{a}, 135.0, 134.9, 134.82, 134.79, 131.5, 131.00, 130.96, 130.8, 130.7, 130.4, 130.2, 130.1, 129.8, 128.7^{a}, 127.0^{b}, 65.8^{a}, 64.9^{b}, 33.5^{a}, 31.9^{b}, 23.3^{a}, 22.8^{b}.

ν_{max} (neat) / cm^{-1}: 2964, 2931, 2819, 1632, 1489, 1454, 1032.

HRMS: calcd. for $C_{21}H_{22}N$ [M-Cl]$^{+}$: 288.1747; found (ESI^{+}): 288.1752.

Peak assignment for regioisomers determined by relative integrations and 2D multinuclear correlation spectroscopy. Ambiguous peaks have been left unassigned.

2-Boc-1-(4-fluorophenyl)-7-phenyl-1,2,3,4-tetrahydropyrido[1,2-a]pyrazin-5-ium chloride (42)

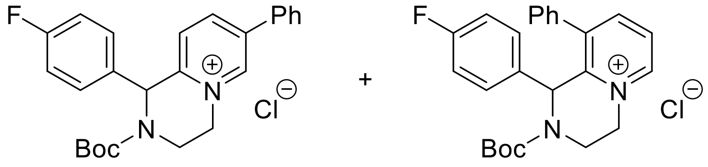

Synthesised according to **GP-2** from 1-phenylpyrrole (65 mg, 0.2 mmol) and 3-chloro-3-phenyldiazirine (92 mg, 0.6 mmol) in TBME (2 mL) as a tan solid (76.4 mg, 0.173 mmol, 87%, 4.0:1 mixture of isomers).

^{1}H NMR (400 MHz, CD_3OD): δ 9.48^{a} (d, J = 2.1 Hz, 1H), 9.16^{b} (d, J = 6.1 Hz, 0.25H), 8.92^{a} (d, J = 8.5 Hz, 1H), 8.58^{b} (d, J = 7.9 Hz, 0.25H), 8.26^{b} (dd, J = 8.1, 6.1 Hz, 0.25H), 7.95 – 7.86^{a} (m, 2H), 7.69 – 7.55^{a} (m, 3H), 7.49^{a} (s, 2H), 7.28 (t, J = 8.8 Hz, 2H), 7.12^{b} (app t, J = 8.7 Hz, 0.5H), 7.03 – 6.90^{b} (m, 0.5H), 4.88 (app dt, J = 11.4, 5.5 Hz, 1H), 4.74^{a} (br s, 1H), 4.51^{b} (br s, 0.25H), 4.11 (br s, 1H), 4.02 – 3.99^{b} (m, 1H), 3.99 – 3.88^{a} (m, 1H), 1.43 (s, 12H)^{a,b}.

^{13}C{^{1}H} NMR (101 MHz, CD_3OD): δ 163.2, 163.0, 160.7, 160.6, 153.1, 150.1^{a}, 149.8^{b}, 146.8, 146.0^{b}, 143.9, 142.5^{a}, 140.1, 137.5^{a}, 134.2, 132.9^{a}, 130.2^{a}, 129.8, 129.5, 129.4, 128.7, 128.2, 127.3^{a}, 126.5, 116.0, 115.9, 115.6, 81.0, 80.9, 55.7, 54.1^{a}, 53.6, 27.9$^{a.b}$.

^{19}F NMR (376 MHz, CD_3OD): δ 134.45 (tt, J = 9.8, 5.5 Hz), -113.59 (br).

ν_{max} (neat) / cm^{-1}: 2975, 1690, 1507, 1391, 1365, 1224, 1160, 1138, 959, 842.

HRMS: calcd. for $C_{25}H_{26}FN_2O_2$ [M-Cl]$^{+}$: 405.1973; found (ESI^{+}): 405.1986.

Peak assignment for regioisomers determined by relative integrations and 2D multinuclear correlation spectroscopy. Ambiguous peaks have been left unassigned.

1-Benzyl-2-phenylpyrimidin-1-ium chloride (43)

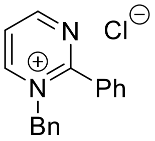

Synthesised according to **GP-2** from 1-benzylpyrazole (32 mg, 0.2 mmol) and 3-chloro-3-phenyldiazirine (92 mg, 0.6 mmol) as a viscous gum (47.9 mg, 0.169 mmol, 85%).

Compound showed poor stability over 1 h in CD_3OD and 12 h in DMSO-d_6.

^{1}H NMR (400 MHz, DMSO-d_6): δ 9.78 (dd, J = 6.4, 1.9 Hz, 1H), 9.61 (dd, J = 4.8, 1.9 Hz, 1H), 8.39 (dd, J = 6.4, 4.8 Hz, 1H), 7.76 – 7.64 (m, 3H), 7.59 (dd, J = 8.2, 6.8 Hz, 2H), 7.37 – 7.27 (m, 3H), 7.18 – 7.01 (m, 2H), 5.91 (s, 2H).

^{13}C{^{1}H} NMR (101 MHz, DMSO-d_6): δ 165.2, 162.5, 154.7, 133.5, 132.4, 132.0, 129.7, 129.4, 129.3, 129.3, 128.8, 123.2, 61.9.

ν_{max} (neat) / cm^{-1}: 1608, 1557, 1468, 1454, 1425, 1268, 1183, 1069, 1013.

HRMS: calcd. for $C_{17}H_{15}N_2$ [M-Cl]$^+$: 247.1230; found (ESI$^+$): 247.1233.

1-Benzyl-2-phenyl-4,6-dimethylpyrimidin-1-ium chloride (44)

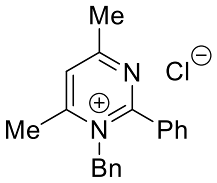

Synthesised according to **GP-2** from 1-benzyl-3,5-dimethylpyrazole (39 mg, 0.2 mmol) and 3-chloro-3-phenyldiazirine (92 mg, 0.6 mmol) as a viscous gum (51.1 mg, 0.164 mmol, 82%).

Compound showed poor stability over 12 h in both CD_3OD and DMSO-d_6.

^{1}H NMR (400 MHz, CD_3OD): δ 8.07 (s, 1H), 7.68 – 7.59 (m, 3H), 7.56 – 7.50 (m, 2H), 7.41 – 7.36 (m, 3H), 7.05 – 7.00 (m, 2H), 5.80 (s, 2H), 2.86 (s, 3H), 2.81 (s, 3H).

^{13}C{^{1}H} NMR (101 MHz, CD_3OD): δ 177.2, 165.5, 164.8, 134.1, 133.7, 133.1, 130.6, 130.1, 129.9, 129.8, 126.9, 125.4, 58.1, 25.1, 21.4.

ν_{max} (neat) / cm^{-1}: 1615, 1545, 1446, 1372, 1339, 1262, 1141, 1028, 987, 759.

HRMS: calcd. for $C_{19}H_{19}N_2$ [M-Cl]$^+$: 275.1543; found (ESI$^+$): 275.1551.

1-Benzyl-2-phenyl-4,6-dimethylpyrimidin-1-ium chloride (45)

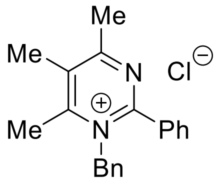

Synthesised according to **GP-2** from 1-benzyl-3,4,5-trimethylpyrazole (40 mg, 0.2 mmol) and 3-chloro-3-phenyldiazirine (92 mg, 0.6 mmol) as a viscous gum (49.3 mg, 0.151 mmol, 76%).

^{1}H NMR (400 MHz, DMSO-d$_6$): δ 7.66 – 7.58 (m, 3H), 7.53 (dd, *J* = 8.7, 6.5 Hz, 2H), 7.37 – 7.35 (m, 3H), 7.11 (dd, *J* = 7.4, 2.2 Hz, 2H), 5.78 (s, 2H), 2.81 (s, 3H), 2.69 (s, 3H), 2.50 (s, 3H).

^{13}C{^{1}H} NMR (101 MHz, DMSO): δ 172.5, 161.6, 159.6, 133.4, 132.7, 131.8, 131.4, 129.1, 128.8, 128.4, 128.2, 126.0, 57.3, 24.3, 18.3, 15.0.

ν_{max} (neat) / cm^{-1}: 1591, 1544, 1453, 1386, 1201, 1077, 1000.

HRMS: calcd. for $C_{20}H_{21}N_2$ [M-Cl]$^+$: 289.1699; found (ESI$^+$): 289.1708.

1-Benzyl-2-phenyl-4,6-diethylpyrimidin-1-ium chloride (46)

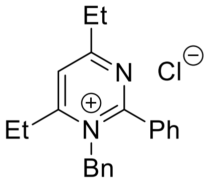

Synthesised according to **GP-2** from 1-benzyl-3,5-diethylpyrazole (42 mg, 0.2 mmol) and 3-chloro-3-phenyldiazirine (92 mg, 0.6 mmol) as a viscous gum (35.9 mg, 0.106 mmol, 53%).

Compound showed poor stability over 12 h in both CD_3OD and DMSO-d_6.

^{1}H NMR (400 MHz, CD_3OD): δ 8.10 (s, 1H), 7.67 – 7.57 (m, 3H), 7.56 – 7.48 (m, 2H), 7.40 – 7.34 (m, 3H), 7.06 – 6.94 (m, 2H), 5.82 (s, 2H), 3.17 (q, *J* = 7.5 Hz, 2H), 3.11 (q, *J* = 7.3 Hz, 2H), 1.46 (t, *J* = 7.5 Hz, 3H), 1.41 (t, *J* = 7.3 Hz, 3H).

^{13}C{^{1}H} NMR (101 MHz, CD_3OD): δ 181.5, 169.9, 165.0, 134.6, 134.0, 133.0, 130.6, 130.0, 129.8, 129.7, 126.9, 122.1, 57.6, 32.6, 27.9, 12.0, 11.9.

ν_{max} (neat) / cm^{-1}: 1610, 1542, 1452, 1407, 1379, 1078, 1027, 907.

HRMS: calcd. for $C_{21}H_{23}N_2$ [M-Cl]$^+$: 303.1856; found (ESI$^+$): 303.1864.

1-Methyl-2-phenyl-5-iodopyrimidin-1-ium chloride (47)

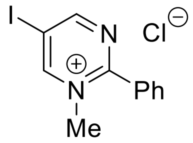

Synthesised according to **GP-2** from 1-methyl-4-iodopyrazole (42 mg, 0.2 mmol) and 3-chloro-3-phenyldiazirine (92 mg, 0.6 mmol) as a yellow solid (37.9 mg, 0.114 mmol, 57%).

Compound showed poor stability over 1 h in CD_3OD.

^{1}H NMR (400 MHz, DMSO-d_6): δ 9.94 (d, J = 2.2 Hz, 1H), 9.77 (d, J = 2.2 Hz, 1H), 7.85 – 7.77 (m, 2H), 7.76 – 7.71 (m, 1H), 7.70 – 7.64 (m, 2H), 4.12 (s, 3H).

^{13}C{^{1}H} NMR (101 MHz, DMSO-d_6): δ 168.7, 160.2, 159.1, 132.2, 130.9, 129.6, 128.9, 93.3, 47.0.

ν_{max} (neat) / cm^{-1}: 1641, 1580, 1546, 1530, 1433, 1367, 1288, 1265, 1215, 1072, 1013.

HRMS: calcd. for $C_{11}H_{10}N_2I$ [M-Cl]$^+$: 296.9883; found (ESI$^+$): 296.9875.

2.3. General Procedure 3 (GP-3): *N*-Benzylation of Azoles

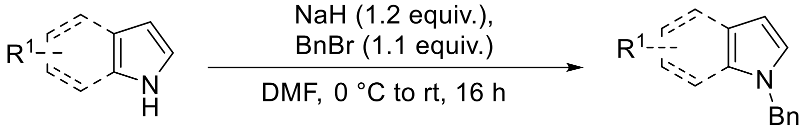

Representative procedure: A solution of the appropriate azole (2.0 mmol) in anhydrous DMF (2 mL) was prepared in a flame-dried flask under an atmosphere of dinitrogen. This was then added drop-wise to a flame-dried Schlenk tube containing a suspension of NaH (60% in mineral oil, 2.4 mmol) in anhydrous DMF (2 mL) cooled to 0 °C. The reaction mixture was warmed to rt and stirred for 30 mins. After cooling once more to 0 °C, benzyl bromide (261 µL, 2.2 mmol) was added drop-wise and the reaction mixture warmed to rt and stirred overnight. The reaction was quenched with saturated aqueous NH_4Cl (5 mL) and extracted with EtOAc (3 × 10 mL). The combined organic portions were washed with a 10% w/w aqueous solution of LiCl (10 mL), then dried over $MgSO_4$, filtered, and concentrated *in vacuo*. Purification by column chromatography using the described eluents or recrystallisation from EtOH afforded the pure product.

1-Benzyl-5-fluoroindole (2)

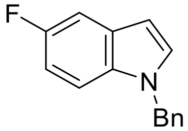

Synthesised according to **GP-3** from 5-fluoroindole (5.00 g, 37.0 mmol), NaH (60% on mineral oil, 1.78 g, 44.4 mmol), and benzyl bromide (6.6 mL, 55.5 mmol). Purification by column chromatography (silica gel; 10% EtOAc in CyH) afforded the product as a colourless solid (6.67 g, 29.6 mmol, 80%).

^{1}H NMR (400 MHz, $CDCl_3$): δ 7.34 – 7.26 (m, 4H), 7.19 – 7.14 (m, 2H), 7.12 – 7.07 (m, 2H), 6.92 (app td, *J* = 9.1, 2.5 Hz, 1H), 6.52 (d, *J* = 3.1 Hz, 1H), 5.31 (s, 2H).

^{13}C{^{1}H} NMR (101 MHz, $CDCl_3$): δ 158.0 (d, *J* = 234.2 Hz), 137.4, 133.0, 130.0, 129.1 (d, *J* = 10.3 Hz), 129.0, 127.9, 126.8, 110.5 (d, *J* = 9.8 Hz), 110.2 (d, *J* = 26.4 Hz), 105.8 (d, *J* = 23.3 Hz), 101.7 (d, *J* = 4.7 Hz), 50.5.

^{19}F NMR (376 MHz, $CDCl_3$): δ −125.37 (app td, *J* = 9.4, 4.3 Hz).

ν_{max} (neat) / cm^{-1}: 2921, 1853, 1486, 1439, 1222, 1184, 1116, 866, 800.

HRMS: calcd. for $C_{15}H_{13}FN$ $[M+H]^+$: 226.1027; found (ESI^+) 226.1025.

m.p. / °C: 63–65.

Characterisation data are consistent with literature values.[2]

1-Benzyl-5-chloroindole

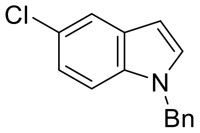

Synthesised according to **GP-3** from 5-chloroindole (303 mg, 2.0 mmol), NaH (60% in mineral oil, 96 mg, 2.4 mmol), and benzyl bromide (0.26 mL, 2.2 mmol). Purification by column chromatography (silica gel; 0-5% EtOAc in CyH) afforded the product as a tan solid (360 mg, 1.49 mmol, 74%).

^{1}H NMR (400 MHz, $CDCl_3$): δ 7.63 (d, *J* = 1.4 Hz, 1H), 7.37–7.26 (m, 3H), 7.23–7.07 (m, 5H), 6.52 (d, *J* = 2.9 Hz, 1H), 5.33 (s, 2H).

^{13}C{^{1}H} NMR (101 MHz, $CDCl_3$): δ 137.2, 134.8, 129.9, 129.8, 129.0, 128.0, 126.8, 125.5, 122.2, 120.5, 110.9, 101.5, 50.5.

ν_{max} (neat) / cm^{-1}: 1470, 1435, 1330, 1289, 1183, 1062, 1049, 1025, 872.

HRMS: calcd. for $C_{15}H_{13}ClN$ [M+H]$^+$: 242.0731; found (ESI$^+$): 242.0718.

m.p. / °C: 62-64.

Characterisation data are consistent with literature values.[3]

1-Benzyl-5-bromoindole

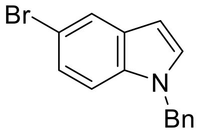

Synthesised according to **GP-3** from 5-bromoindole (388 mg, 2.0 mmol), NaH (60% in mineral oil, 96 mg, 2.4 mmol), and benzyl bromide (0.26 mL, 2.2 mmol). Purification by column chromatography (silica gel; 0-5% EtOAc in CyH) afforded the product as a colourless solid (423 mg, 1.48 mmol, 74%).

^{1}H NMR (400 MHz, $CDCl_3$): δ 7.80 (d, *J* = 1.9 Hz, 1H), 7.33–7.27 (m, 3H), 7.25 (dd, *J* = 8.7, 1.9 Hz, 1H), 7.14 (dd, *J* = 5.9, 3.0 Hz, 2H), 7.08 (dd, *J* = 7.8, 1.7 Hz, 2H), 6.49 (d, *J* = 3.0 Hz, 1H), 5.33 (s, 2H).

^{13}C{^{1}H} NMR (101 MHz, $CDCl_3$): δ 137.2, 135.1, 130.6, 129.6, 129.0, 127.9, 126.8, 124.7, 123.6, 113.0, 111.3, 101.4, 50.4.

ν_{max} (neat) / cm^{-1}: 1493, 1468, 1436, 1391, 1351, 1326, 1286, 1182, 1048, 897, 857.

HRMS: calcd. for $C_{15}H_{13}NBr$ $[M+H]^+$: 286.0226; found (ESI^+): 286.0219.

m.p. / °C: 94-96.

Characterisation data are consistent with literature values.[2]

1-Benzylindole-5-carbonitrile

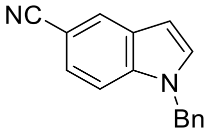

Synthesised according to **GP-3** from 5-cyanoindole (284 mg, 2.0 mmol), NaH (60% in mineral oil, 96 mg, 2.4 mmol), and benzyl bromide (0.26 mL, 2.2 mmol). Recrystallisation from EtOH afforded the product as a colourless solid (324 mg, 1.40 mmol, 70%).

^{1}H NMR (400 MHz, $CDCl_3$): δ 7.99 (d, *J* = 1.5 Hz, 1H), 7.40 (dd, *J* = 8.6, 1.5 Hz, 1H), 7.39–7.30 (m, 4H), 7.28 (d, *J* = 3.5 Hz, 1H), 7.12 (dd, *J* = 7.7, 1.8 Hz, 2H), 6.66 (dd, *J* = 3.3, 0.8 Hz, 1H), 5.38 (s, 2H).

^{13}C{^{1}H} NMR (101 MHz, $CDCl_3$): δ 137.9, 136.5, 130.7, 129.1, 128.6, 128.2, 126.9, 126.7, 124.8, 120.9, 110.7, 102.9, 102.8, 50.5.

ν_{max} (neat) / cm^{-1}: 2221, 1604, 1482, 1451, 1436, 1338, 1301, 1183, 884, 805.

HRMS: calcd. for $C_{16}H_{13}N_2$ $[M+H]^+$: 233.1073; found (ESI^+): 233.1074.

m.p. / °C: 107-109.

Characterisation data are consistent with literature values.[4]

1-Benzyl-5-nitroindole

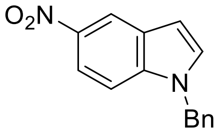

Synthesised according to **GP-3** from 5-nitroindole (324 mg, 2.0 mmol), NaH (60% on mineral oil, 96 mg, 2.4 mmol), and benzyl bromide (0.26 mL, 2.2 mmol). Purification by column chromatography (silica gel; 10% EtOAc in CyH) afforded the product as a yellow solid (297 mg, 1.18 mmol, 59%).

^{1}H NMR (400 MHz, $CDCl_3$): δ 8.62 (d, *J* = 2.2 Hz, 1H), 8.08 (dd, *J* = 9.0, 2.2 Hz, 1H), 7.38–7.26 (m, 5H), 7.16–7.06 (m, 2H), 6.74 (dd, *J* = 3.3, 0.9 Hz, 1H), 5.37 (s, 2H).

^{13}C{^{1}H} NMR (101 MHz, $CDCl_3$): δ 141.8, 139.1, 136.2, 131.5, 129.1, 128.2, 128.0, 126.8, 118.3, 117.5, 109.6, 104.5, 50.8.

ν_{max} (neat) / cm^{-1}: 3094, 2928, 1606, 1505, 1477, 1440, 1401, 1326, 1311, 1292, 1173, 1069, 906, 808.

HRMS: calcd. for $C_{15}H_{12}N2O_2Na$ $[M+Na]^+$: 275.0796; found (ESI^+): 275.0801.

m.p. / °C: 106-108.

Characterisation data are consistent with literature values.[5]

1-Benzylindole

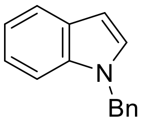

Synthesised according to **GP-3** from indole (234 mg, 2.0 mmol), NaH (60% in mineral oil, 96 mg, 2.4 mmol), and benzyl bromide (0.26 mL, 2.2 mmol). Purification by column chromatography (silica gel; 0-5% EtOAc in CyH) afforded the product as a brown solid (302 mg, 1.46 mmol, 73%).

^{1}H NMR (400 MHz, $CDCl_3$): δ 7.66 (app dt, *J* = 7.8, 1.2 Hz, 1H), 7.34–7.26 (m, 4H), 7.18 (app dt, *J* = 7.2, 1.2 Hz, 1H), 7.15–7.09 (m, 4H), 6.57 (dd, *J* = 3.2, 0.9 Hz, 1H), 5.34 (s, 2H).

^{13}C{^{1}H} NMR (101 MHz, $CDCl_3$): δ 137.7, 136.4, 128.9, 128.8, 128.4, 127.7, 126.9, 121.8, 121.1, 119.7, 109.8, 101.8, 50.2.

ν_{max} (neat) / cm^{-1}: 1703, 1610, 1509, 1462, 1316, 1158.

HRMS: calcd. for $C_{15}H_{14}N$ [M+H]$^{+}$: 208.1121; found (ESI^{+}): 208.1106.

m.p. / °C: 42-44.

Characterisation data are consistent with literature values.[3]

1-Benzyl-5-(benzyloxy)indole

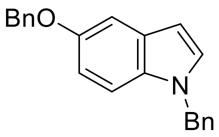

Synthesised according to **GP-3** from 5-hydroxyindole (266 mg, 2.0 mmol), NaH (60% on mineral oil, 192 mg, 4.8 mmol), and benzyl bromide (0.53 mL, 4.4 mmol). Purification by recrystallisation from EtOH afforded the product as a white solid (344 mg, 1.10 mmol, 55%).

^{1}H NMR (400 MHz, $CDCl_3$): δ 7.47 (d, *J* = 7.0 Hz, 2H), 7.38 (app t, *J* = 7.4 Hz, 2H), 7.35 – 7.26 (m, 4H), 7.19 (d, *J* = 2.5 Hz, 1H), 7.16 (d, *J* = 8.9 Hz, 1H), 7.13 – 7.07 (m, 3H), 6.91 (dd, *J* = 8.9, 2.5 Hz, 1H), 6.46 (d, *J* = 3.1 Hz, 1H), 5.29 (s, 2H), 5.10 (s, 2H).

^{13}C{^{1}H} NMR (101 MHz, $CDCl_3$): δ 153.5, 137.9, 137.7, 132.0, 129.2, 129.0, 128.9, 128.7, 127.9, 127.73, 127.68, 126.9, 112.9, 110.6, 104.3, 101.4, 71.0, 50.4.

ν_{max} (neat) / cm^{-1}: 1614, 1511, 1486, 1452, 1433, 1363, 1316, 1264, 1219, 1183, 1015, 963, 948.

HRMS: calcd. for $C_{22}H_{20}NO$ $[M+H]^+$: 314.1539; found (ESI^+): 314.1545.

m.p. / °C: 111-113.

Characterisation data are consistent with literature values.[6]

1-Benzyl-5-methoxyindole

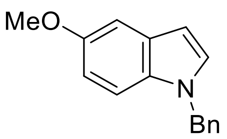

Synthesised according to **GP-3** from 5-methoxyindole (294 mg, 2.0 mmol), NaH (60% in mineral oil, 96 mg, 2.4 mmol), and benzyl bromide (0.26 mL, 2.2 mmol). Recrystallisation from EtOH afforded the product as an off-white solid (332 mg, 1.40 mmol, 70%).

^{1}H NMR (400 MHz, $CDCl_3$): δ 7.33 – 7.22 (m, 3H), 7.16 (d, *J* = 8.9 Hz, 1H), 7.14 – 7.07 (m, 4H), 6.84 (dd, *J* = 8.9, 2.4 Hz, 1H), 6.48 (d, *J* = 3.0 Hz, 1H), 5.29 (s, 2H), 3.85 (s, 3H).

^{13}C{^{1}H} NMR (101 MHz, $CDCl_3$): δ 154.2, 137.8, 131.8, 129.2, 129.0, 128.9, 127.7, 126.8, 112.2, 110.6, 102.7, 101.3, 56.0, 50.4.

ν_{max} (neat) / cm^{-1}: 1619, 1574, 1486, 1444, 1402, 1344, 1234, 1151, 1132, 1028, 828.

HRMS: calcd. for $C_{16}H_{16}NO$ $[M+H]^+$: 238.1225; found (ESI^+): 238.1226.

m.p. / °C: 77-80.

Characterisation data are consistent with literature values.[2]

1-Benzyl-5-methylindole

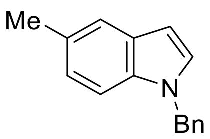

Synthesised according to **GP-3** from 5-methylindole (262 mg, 2.0 mmol), NaH (60% in mineral oil, 96 mg, 2.4 mmol), and benzyl bromide (0.26 mL, 2.2 mmol). Purification by column chromatography (silica gel; CyH) followed by recrystallisation from EtOH afforded the product as a tan solid (176 mg, 0.797 mmol, 40%).

^{1}H NMR (400 MHz, CDCl$_3$): δ 7.43 (m, 1H), 7.33 – 7.21 (m, 3H), 7.16 (d, *J* = 8.4 Hz, 1H), 7.13 – 7.05 (m, 3H), 6.99 (dd, *J* = 8.4, 1.8 Hz, 1H), 6.46 (dd, *J* = 3.1, 0.9 Hz, 1H), 5.30 (s, 2H), 2.44 (s, 3H).

^{13}C{^{1}H} NMR (101 MHz, CDCl$_3$): δ 137.8, 134.9, 129.1, 128.9, 128.5, 127.7, 126.8, 123.4, 120.8, 109.5, 101.2, 50.3, 21.5.

ν_{max} (neat) / cm^{-1}: 1710, 1486, 1451, 1330, 1234, 1192, 1181.

HRMS: calcd. for $C_{16}H_{16}N$ [M+H]$^+$: 222.1277; found (ESI$^+$): 222.1290

m.p. / °C: 37-38 °C.

Characterisation data are consistent with literature values.[7]

1-Benzyl-5-hydroxyindole

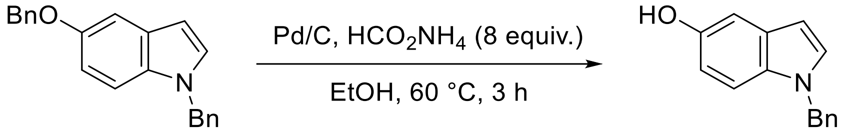

Prior to reaction, EtOH was degassed by sparging with dinitrogen for 30 mins. An oven-dried Schlenk flask was charged with Pd/C (10 mg) and 1-benzyl-5-(benzyloxy)indole (94 mg, 0.3 mmol) which was then evacuated and back-filled with dinitrogen three times. Ammonium formate (151 mmol, 2.4 mmol) was then added followed by degassed EtOH (1.5 mL). The reaction was heated to 60 °C and stirred for 3 hrs until full consumption of starting material by TLC (10% EtOAc in CyH). After cooling to rt, the reaction mixture was diluted with CH_2Cl_2 (10 mL) and filtered through Celite. The filtrate was washed with water (10 mL) and the aqueous layer was extracted with CH_2Cl_2 (3 × 10 mL). The combined organic portions were dried over $MgSO_4$, filtered, and concentrated *in vacuo* to afford the product as a colourless solid (56.4 mg, 0.252 mmol, 84%).

^{1}H NMR (400 MHz, $CDCl_3$): δ 7.37 – 7.22 (m, 4H), 7.14 – 7.07 (m, 3H), 7.05 (d, *J* = 2.5 Hz, 1H), 6.74 (dd, *J* = 8.7, 2.5 Hz, 1H), 6.42 (dd, *J* = 3.1, 0.8 Hz, 1H), 5.28 (s, 2H), 4.42 (brs, 1H).

^{13}C{^{1}H} NMR (101 MHz, $CDCl_3$): δ 149.6, 137.7, 132.0, 129.5, 129.4, 128.9, 127.7, 126.9, 111.6, 110.5, 105.5, 101.0, 50.5.

ν_{max} (neat) / cm^{-1}: 3199, 1618, 1506, 1451, 1433, 1362, 1229, 1185, 1142, 1129.

HRMS: calcd. for $C_{15}H_{13}NO$ $[M+H]^+$: 224.1070; found (ESI^+): 224.1072.

m.p. / °C: 86-88.

1-Benzyl-6-(benzyloxy)indole

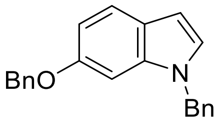

Synthesised according to **GP-3** from 6-benzyloxyindole (446 mg, 2.0 mmol), NaH (60% in mineral oil, 96 mg, 2.4 mmol), and benzyl bromide (0.26 mL, 2.2 mmol). Recrystallisation from EtOH afforded the product as a white solid (306 mg, 0.98 mmol, 49%).

^{1}H NMR (400 MHz, $CDCl_3$): δ 7.52 (d, *J* = 8.5 Hz, 1H), 7.46 – 7.40 (m, 2H), 7.40 – 7.34 (m, 2H), 7.34 – 7.24 (m, 4H), 7.14 – 7.06 (m, 2H), 7.03 (d, *J* = 3.2 Hz, 1H), 6.87 (dd, *J* = 8.6, 2.3 Hz, 1H), 6.82 (d, *J* = 2.3 Hz, 1H), 6.48 (dd, *J* = 3.2, 0.8 Hz, 1H), 5.25 (s, 2H), 5.05 (s, 2H).

^{13}C{^{1}H} NMR (101 MHz, $CDCl_3$): δ 155.6, 137.58, 137.55, 137.1, 128.9, 128.7, 128.0, 127.7, 127.5, 126.9, 123.4, 121.6, 110.2, 101.7, 95.1, 70.8, 50.2.

ν_{max} (neat) / cm^{-1}: 1613, 1511, 1486, 1452, 1433, 1363, 1316, 1263, 1219, 1182, 1014, 962, 948.

HRMS: calcd. for $C_{22}H_{20}NO$ [M+H]$^{+}$: 314.1539; found (ESI^{+}): 314.1537.

m.p. / °C: 79-81.

1-Benzyl-5,6-dimethoxyindole

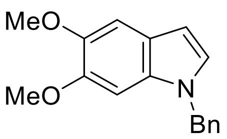

Synthesised according to **GP-3** from 5,6-dimethylindole (354 mg, 2.0 mmol), NaH (60% in mineral oil, 96 mg, 2.4 mmol), and benzyl bromide (0.26 mL, 2.2 mmol). Purification by column chromatography (silica gel; 0-5% EtOAc in CyH) afforded the product as a white solid (358 mg, 1.34 mmol, 67%).

^{1}H NMR (400 MHz, CDCl$_3$): δ 7.34 – 7.26 (m, 3H), 7.13 – 7.07 (m, 3H), 7.01 (d, *J* = 3.1 Hz, 1H), 6.72 (s, 1H), 6.44 (dd, *J* = 3.1, 0.8 Hz, 1H), 5.28 (s, 2H), 3.92 (s, 3H), 3.84 (s, 3H).

^{13}C{^{1}H} NMR (101 MHz, CDCl$_3$): δ 147.0, 145.2, 137.7, 130.9, 128.9, 127.7, 126.9, 126.8, 121.5, 102.7, 101.3, 93.4, 56.4, 56.4, 50.4.

ν_{max} (neat) / cm^{-1}: 1487, 1447, 1361, 1257, 1238, 1203, 1144, 1045, 846, 810.

HRMS: calcd. for $C_{17}H_{18}NO_2$ [M+H]$^+$: 268.1332; found (ESI$^+$): 268.1318

m.p. / °C: 92-93.

1-Benzyl-4-methylindole

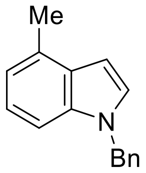

Synthesised according to **GP-3** from 4-methylindole (262 mg, 2.0 mmol), NaH (60% in mineral oil, 96 mg, 2.4 mmol), and benzyl bromide (0.26 mL, 2.2 mmol). Purification by column chromatography (silica gel; CyH) afforded the product as an off-white solid (263 mg, 1.19 mmol, 59%).

^{1}H NMR (400 MHz, $CDCl_3$): δ 7.34 – 7.22 (m, 3H), 7.18 – 7.03 (m, 5H), 6.92 (app dt, *J* = 6.9, 1.0 Hz, 1H), 6.57 (dd, *J* = 3.2, 1.0 Hz, 1H), 5.32 (s, 2H), 2.58 (s, 3H).

^{13}C{^{1}H} NMR (101 MHz, $CDCl_3$): δ 137.8, 136.2, 130.6, 128.9, 128.7, 127.74, 127.69, 126.9, 122.0, 119.9, 107.5, 100.3, 50.3, 18.9.

ν_{max} (neat) / cm^{-1}: 1604, 1583, 1493, 1452, 1423, 1336, 1300, 1212, 1157.

HRMS: calcd. for $C_{16}H_{16}N$ [M+H]$^+$: 222.1277; found (ESI$^+$): 222.1282

m.p. / °C: 50-53.

1-Benzyl-7-methylindole

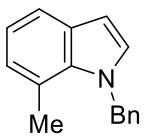

Synthesised according to **GP-3** from 7-methylindole (262 mg, 2.0 mmol), NaH (60% in mineral oil, 96 mg, 2.4 mmol), and benzyl bromide (0.26 mL, 2.2 mmol). Purification by column chromatography (silica gel; CyH) afforded the product as a colourless solid (326 mg, 1.47 mmol, 74%).

^{1}H NMR (400 MHz, $CDCl_3$): δ 7.54 (d, J = 7.9 Hz, 1H), 7.34 – 7.24 (m, 3H), 7.09 (d, J = 3.1 Hz, 1H), 7.03 (app t, J = 7.5 Hz, 1H), 6.96 – 6.86 (m, 3H), 6.58 (d, J = 3.1 Hz, 1H), 5.62 (s, 2H), 2.56 (s, 3H).

^{13}C{^{1}H} NMR (101 MHz, $CDCl_3$): δ 139.8, 135.2, 130.3, 129.9, 129.0, 127.4, 125.6, 124.7, 121.2, 120.0, 119.3, 102.2, 52.4, 19.7.

ν_{max} (neat) / cm^{-1}: 1489, 1445, 1413, 1357, 1312, 1179, 1073, 1031, 961.

HRMS: calcd. for $C_{16}H_{16}N$ $[M+H]^+$: 222.1277; found (ESI$^+$): 222.1271

m.p. / °C: 58-61.

Characterisation data are consistent with literature values.[8]

1-Benzyl-7-bromoindole

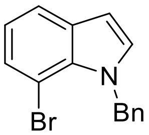

Synthesised according to **GP-3** from 7-bromoindole (392 mg, 2.0 mmol), NaH (60% in mineral oil, 96 mg, 2.4 mmol), and benzyl bromide (0.26 mL, 2.2 mmol). Recrystallisation from EtOH afforded the product as a colourless solid (304 mg, 1.06 mmol, 53%).

^{1}H NMR (400 MHz, CDCl$_3$): δ 7.60 (d, *J* = 7.7 Hz, 1H), 7.35 (d, *J* = 7.7 Hz, 1H), 7.27 (m, 3H), 7.11 (d, *J* = 3.2 Hz, 1H), 7.02 (d, *J* = 7.0 Hz, 2H), 6.96 (app t, *J* = 7.7 Hz, 1H), 6.58 (d, *J* = 3.2 Hz, 1H), 5.84 (s, 2H).

^{13}C{^{1}H} NMR (101 MHz, CDCl$_3$): δ 139.2, 132.8, 132.0, 131.4, 128.8, 127.4, 127.3, 126.3, 120.9, 120.6, 103.9, 102.5, 51.5.

ν_{max} (neat) / cm^{-1}: 1554, 1479, 1440, 1416, 1355, 1311, 1176, 1040, 914, 810.

HRMS: calcd. for $C_{15}H_{13}NBr$ [M+H]$^+$: 286.0226; found (ESI$^+$): 286.0200.

m.p. / °C: 71-74.

Characterisation data are consistent with literature values.[3]

1-Benzyl-4-methoxyindole

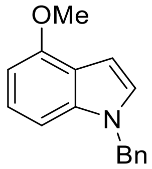

Synthesised according to **GP-3** from 4-methoxyindole (294 mg, 2.0 mmol), NaH (60% in mineral oil, 96 mg, 2.4 mmol), and benzyl bromide (0.26 mL, 2.2 mmol). Recrystallisation from EtOH afforded the product as a colourless solid (413 mg, 1.74 mmol, 87%).

^{1}H NMR (400 MHz, $CDCl_3$): δ 7.36 – 7.21 (m, 3H), 7.14 – 7.07 (m, 3H), 7.04 (d, *J* = 3.2 Hz, 1H), 6.91 (d, *J* = 8.2 Hz, 1H), 6.66 (dd, *J* = 3.2, 0.8 Hz, 1H), 6.53 (d, *J* = 7.7 Hz, 1H), 5.31 (s, 2H), 3.97 (s, 3H).

^{13}C{^{1}H} NMR (101 MHz, $CDCl_3$): δ 153.6, 137.9, 137.7, 128.9, 127.7, 126.93, 126.87, 122.7, 119.3, 103.3, 99.6, 99.1, 55.5, 50.4.

ν_{max} (neat) / cm^{-1}: 1582, 1494, 1450, 1353, 1253, 1221, 1059.

HRMS: calcd. for $C_{16}H_{16}NO$ [M+H]$^+$: 238.1227; found (ESI$^+$): 238.1229.

m.p. / °C: 92-93.

1-Benzyl-2,3-dimethylindole

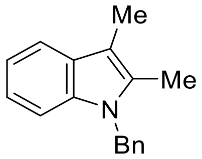

Synthesised according to **GP-3** from 2,3-dimethylindole (266 mg, 2.0 mmol), NaH (60% in mineral oil, 96 mg, 2.4 mmol), and benzyl bromide (0.26 mL, 2.2 mmol). Purification by column chromatography (silica gel; 0-5% EtOAc in CyH) afforded the product as a colourless solid (174 mg, 0.74 mmol, 37%).

^{1}H NMR (400 MHz, $CDCl_3$): δ 7.56 – 7.51 (m, 1H), 7.29 – 7.17 (m, 4H), 7.14 – 7.07 (m, 2H), 7.00 – 6.94 (m, 2H), 5.30 (s, 2H), 2.29 (app s, 6H).

^{13}C{^{1}H} NMR (101 MHz, $CDCl_3$): δ 138.3, 136.4, 132.4, 128.72, 128.67, 127.2, 126.0, 120.8, 118.8, 118.0, 108.8, 107.0, 46.5, 10.2, 8.9.

ν_{max} (neat) / cm^{-1}: 1469, 1450, 1357, 1331, 1196.

HRMS: calcd. for $C_{17}H_{18}N$ [M+H]$^+$: 236.1434; found (ESI$^+$): 236.1442

m.p. / °C: 52-55.

Characterisation data are consistent with literature values.[9]

1-Methyl-5-fluoroindole

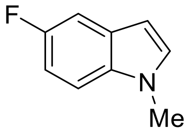

Synthesised according to a *modified* **GP-3** from 5-fluoroindole (676 mg, 5.0 mmol), NaH (60% in mineral oil, 300 mg, 7.5 mmol), and methyl iodide (0.41 mL, 6.5 mmol) in THF (15 mL). Purification by column chromatography (silica gel; 10% EtOAc in CyH) afforded the product as a red solid (637 mg, 4.27 mmol, 85%).

^{1}H NMR (400 MHz, $CDCl_3$): δ 7.30 (dd, *J* = 9.7, 2.5 Hz, 1H), 7.24 (dd, *J* = 8.9, 4.3 Hz, 1H), 7.10 (d, *J* = 3.1 Hz, 1H), 7.00 (app td, *J* = 9.1, 2.5 Hz, 1H), 6.46 (dd, *J* = 3.1, 0.9 Hz, 1H), 3.79 (s, 3H).

^{13}C{^{1}H} NMR (101 MHz, $CDCl_3$): δ 157.9 (d, *J* = 233.7 Hz), 133.4, 130.4, 128.7 (d, *J* = 10.2 Hz), 110.0, 109.9, 109.8, 105.5 (d, *J* = 23.4 Hz), 100.9 (d, *J* = 4.7 Hz).

^{19}F NMR (376 MHz, $CDCl_3$): δ -125.74 (app td, *J* = 9.5, 4.3 Hz).

ν_{max} (neat) / cm^{-1}: 1622, 1574, 1491, 1447, 1424, 1338, 1281, 1237, 1222, 1118, 1078, 947, 857.

m.p. / °C: 54-57.

Characterisation data are consistent with literature values.[10]

1-(4-Methoxybenzyl)-5-fluoroindole

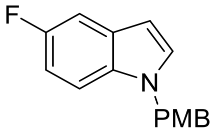

Synthesised according to a *modified* **GP-3** from 5-fluoroindole (1.35 g, 10.0 mmol), NaH (60% in mineral oil, 480 mg, 12 mmol), and 4-methoxybenzyl chloride (2.0 mL, 15 mmol). Purification by column chromatography (silica gel; 0-10% EtOAc in CyH) afforded the product as a yellow oil (1.05 g, 3.88 mmol, 39%).

^{1}H NMR (400 MHz, $CDCl_3$): δ 7.28 (dd, *J* = 9.7, 2.5 Hz, 1H), 7.18 (dd, *J* = 8.9, 4.3 Hz, 1H), 7.15 (d, *J* = 3.1 Hz, 1H), 7.05 (d, *J* = 8.6 Hz, 2H), 6.91 (app td, *J* = 9.1, 2.5 Hz, 1H), 6.84 (d, *J* = 8.6 Hz, 2H), 6.48 (d, *J* = 3.0 Hz, 1H), 5.24 (s, 2H), 3.78 (s, 3H).

^{13}C{^{1}H} NMR (101 MHz, $CDCl_3$): δ 159.3, 158.0 (d, *J* = 234.1 Hz), 133.0, 129.8, 129.3, 129.1 (d, *J* = 10.2 Hz), 128.3, 114.3, 110.5 (d, *J* = 9.8 Hz), 110.1 (d, *J* = 26.4 Hz), 105.8 (d, *J* = 23.3 Hz), 101.6 (d, *J* = 4.7 Hz), 55.4, 50.1.

^{19}F NMR (376 MHz, $CDCl_3$): δ -125.43 (app td, *J* = 9.4, 4.3 Hz).

ν_{max} (neat) / cm^{-1}: 2836, 1612, 1511, 1485,1463, 1244, 1227, 1175, 1116, 1031, 845, 807.

HRMS: calcd. for $C_{16}H_{15}FNO$ $[M+H]^+$: 256.1132; found (ESI$^+$): 256.1136.

Characterisation data are consistent with literature values.[11]

1-Benzyl-7-azaindole

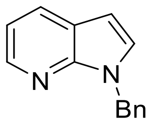

Synthesised according to **GP-3** from 7-azaindole (236 mg, 2.0 mmol), NaH (60% on mineral oil, 96 mg, 2.4 mmol), and benzyl bromide (0.26 mL, 2.2 mmol). Purification by column chromatography (silica gel; 10-20% EtOAc in CyH) afforded the product as a colourless solid (294 mg, 1.41 mmol, 70%).

^{1}H NMR (400 MHz, $CDCl_3$): δ 8.36 (dd, *J* = 4.7, 1.6 Hz, 1H), 7.93 (dd, *J* = 7.8, 1.6 Hz, 1H), 7.35 – 7.24 (m, 3H), 7.24 – 7.16 (m, 3H), 7.08 (dd, *J* = 7.8, 4.7 Hz, 1H), 6.49 (d, *J* = 3.5 Hz, 1H), 5.52 (s, 2H).

^{13}C{^{1}H} NMR (101 MHz, $CDCl_3$): δ 147.9, 143.2, 138.0, 128.9, 128.8, 128.0, 127.7, 127.6, 120.6, 116.0, 100.2, 47.9.

ν_{max} (neat) / cm^{-1}: 1591, 1566, 1485, 1433, 1420, 1347, 1313, 1297, 1253, 1209, 1183, 889.

HRMS: calcd. for $C_{14}H_{13}N_2$ $[M+H]^+$: 209.1073; found (ESI^+): 209.1078.

m.p. / °C: 77-78.

Characterisation data are consistent with literature values.[12]

1-Benzylmelatonin

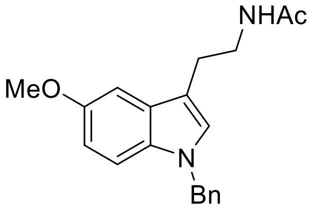

Synthesised according to **GP-3** from melatonin (465 mg, 2.0 mmol), NaH (60% in mineral oil, 96 mg, 2.4 mmol), and benzyl bromide (0.26 mL, 2.2 mmol). Recrystallisation from EtOAc afforded the product as a colourless solid (379 mg, 1.18 mmol, 59%).

^{1}H NMR (400 MHz, $CDCl_3$): δ 7.38 – 7.25 (m, 3H), 7.19 (d, *J* = 8.9 Hz, 1H), 7.15 – 7.09 (m, 2H), 7.07 (d, *J* = 2.4 Hz, 1H), 6.96 (s, 1H), 6.87 (dd, *J* = 8.9, 2.4 Hz, 1H), 5.56 (br s, 1H), 5.27 (s, 2H), 3.88 (s, 3H), δ 3.58 (app q, *J* = 6.6 Hz, 1H), 2.96 (t, *J* = 6.6 Hz, 2H), 1.94 (s, 3H).

^{13}C{^{1}H} NMR (101 MHz, $CDCl_3$): 170.1, 154.1, 137.7, 132.2, 128.9, 128.5, 127.8, 126.91, 126.85, 112.4, 111.8, 110.8, 100.8, 56.1, 50.3, 39.9, 25.4, 23.5.

ν_{max} (neat) / cm^{-1}: 3313, 2913, 1637, 1556, 1488, 1434, 1230, 1057, 1042, 857.

HRMS: calcd. for $C_{20}H_{23}N_2O_2$ [M+H]$^+$: 323.1755; found (ESI$^+$): 323.1754.

m.p. / °C: 118-120.

Characterisation data are consistent with literature values.[13]

Nα-Acetyl-1-benzyltryptophan

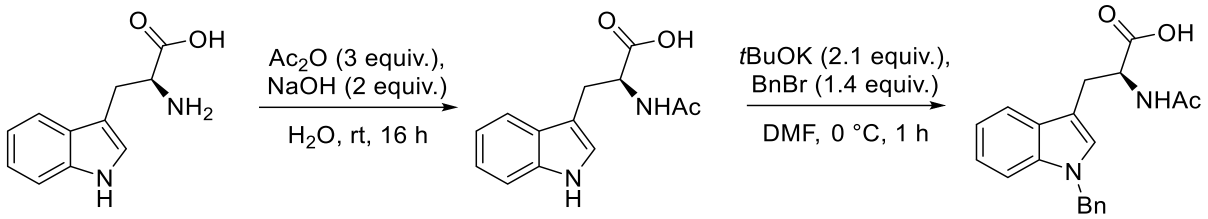

Step 1: To a suspension of *L*-tryptophan (2.04 g, 10 mmol) in water (20 mL) was added pulverised NaOH (800 mg, 20 mmol) in one portion. Full dissolution of the suspended solid was observed, and the reaction mixture was stirred at rt for 30 mins. Ac_2O (2.84 mL, 30 mmol) was then added, and a white precipitate formed. The reaction was stirred at rt overnight after which time the precipitate was collected by vacuum filtration, washed with water (10 mL) and dried under reduced pressure to afford the pure product as a colourless solid (1.34 g, 5.44 mmol, 54%).

^{1}H NMR (400 MHz, DMSO-d$_6$): δ 12.61 (brs, 1H), 10.83 (s, 1H), 8.12 (d, *J* = 7.8 Hz, 1H), 7.53 (d, *J* = 7.8 Hz, 1H), 7.39 – 7.30 (m, 1H), 7.14 (d, *J* = 2.4 Hz, 1H), 7.07 (ddd, *J* = 8.2, 7.0, 1.2 Hz, 1H), 6.98 (ddd, *J* = 8.2, 7.0, 1.2 Hz, 1H), 4.45 (app td, *J* = 8.2, 5.1 Hz, 1H), 3.16 (dd, *J* = 14.7, 5.1 Hz, 1H), 2.98 (dd, *J* = 14.7, 8.7 Hz, 1H), 1.80 (s, 3H).

^{13}C{^{1}H} NMR (101 MHz, DMSO-d$_6$): δ 173.6, 169.2, 136.1, 127.2, 123.5, 120.9, 118.3, 118.1, 111.4, 110.0, 53.0, 27.1, 22.4.

HRMS: calcd. for $C_{13}H_{14}N_2O_3Na$ $[M+Na]^+$: 269.0897; found (ESI$^+$): 269.0894.

Step 2: According to the literature procedure,[14] the *N*-acetyl tryptophan (1.23 g, 5.0 mmol) was transferred to a Schlenk flask under an atmosphere of dinitrogen, and dissolved in anhydrous DMF (25 mL). *t*BuOK (1.18 g, 10.5 mmol) was added in one portion at rt and stirred for 5 mins until all solids fully dissolved. The reaction mixture was cooled to 0 °C and BnBr (0.84 mL, 7.0 mmol) was added drop-wise. The resulting solution was warmed to rt and stirred for 1 hr. The reaction was quenched by addition of 1 M aqueous HCl (10 mL) and then extracted with EtOAc (3 × 20 mL). The combined organics were washed with a 10 wt% aqueous LiCl solution (2 × 30 mL) and dried over Na_2SO_4, filtered, and concentrated *in vacuo*. Purification by recrystallisation from EtOH afforded the product as a colourless solid (1.05 g, 3.12 mmol, 42%).

^{1}H NMR (400 MHz, DMSO-d$_6$): δ 12.64 (s, 1H), 8.17 (d, *J* = 7.9 Hz, 1H), 7.56 (d, *J* = 7.8 Hz, 1H), 7.38 (d, *J* = 8.1 Hz, 1H), 7.28 (d, *J* = 7.9 Hz, 3H), 7.25 – 7.20 (m, 1H), 7.18 – 7.12 (m, 2H), 7.09 (d, *J* = 7.4 Hz, 1H), 7.02 (d, *J* = 7.5 Hz, 1H), 5.37 (s, 2H), 4.49 (td, *J* = 8.4, 5.2 Hz, 1H), 3.19 (dd, *J* = 14.5, 5.2 Hz, 1H), 3.00 (dd, *J* = 14.6, 8.8 Hz, 1H), 1.80 (s, 3H).

$^{13}C\{^{1}H\}$ NMR (101 MHz, $CDCl_3$): δ 173.5, 169.2, 138.4, 136.0, 128.5, 127.8, 127.5, 127.2, 126.9, 121.2, 118.7, 118.6, 110.1, 110.0, 52.9, 48.9, 27.1, 22.4.

ν_{max} (neat) / cm^{-1}: 3373, 1713, 1582, 1534, 1438, 1327, 1195, 1130.

HRMS: calcd. for $C_{20}H_{19}N_2O_3$ $[M-H]^-$: 335.1401; found (ESI^-): 335.1386.

m.p. / °C: 157-159.

Nα-Acetyl-1-benzyl-*L*-tryptophan methyl ester

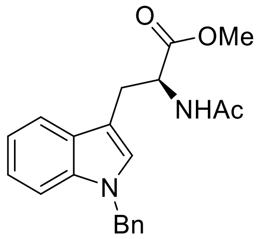

To a solution of N^{α}-Acetyl-1-benzyltryptophan (1.68 g, 5.0 mmol) in MeOH (15 mL) was added $SOCl_2$ (0.51 mL, 7.0 mmol) drop-wise and the reaction stirred at 0 °C for 30 mins. It was then heated to 50 °C and stirred for 1 hr until consumption of starting material by TLC. The volatiles were then removed *in vacuo* and the residue purified by column chromatography (silica gel; 50% EtOAc in CyH) to afford the product as a colourless solid (918 mg, 2.62 mmol, 48%).

^{1}H NMR (400 MHz, $CDCl_3$): δ 7.54 (d, *J* = 7.8 Hz, 1H), 7.33 – 7.23 (m, 4H), 7.18 (app td, *J* = 6.9, 0.9 Hz, 1H), 7.15 – 7.10 (m, 1H), 7.08 (m, 2H), 6.87 (s, 1H), 5.98 (d, *J* = 7.7 Hz, 1H), 5.28 (s, 2H), 4.95 (app dt, *J* = 7.9, 5.2 Hz, 1H), 3.64 (s, 3H), 3.38 – 3.25 (m, 2H), 1.94 (s, 3H).

^{13}C{^{1}H} NMR (101 MHz, $CDCl_3$): δ 172.5, 169.7, 137.5, 136.7, 128.9, 128.6, 127.8, 126.9, 126.8, 122.2, 119.6, 119.0, 109.9, 109.5, 53.3, 52.4, 50.0, 27.8, 23.4.

ν_{max} (neat) / cm^{-1}: 3309, 1752, 1646, 1543, 1467, 1431, 1372, 1338, 1272, 1210, 1176, 1126.

HRMS: calcd. for $C_{21}H_{23}N_2O_3$ $[M+H]^+$: 351.1703; found (ESI^+): 351.1693.

m.p. / °C: 152-155.

Methyl N$^{\alpha}$-acetyl-1-benzyltryptophylglycinate

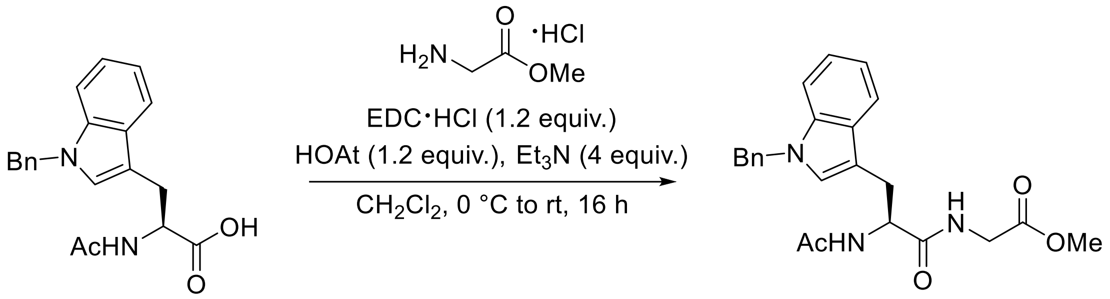

To a suspension of glycine methyl ester hydrochloride (252 mg, 2.0 mmol) in CH_2Cl_2 (20 mL) at 0 °C was added sequentially Et_3N (1.1 mL, 8.0 mmol), HOAt (327 mg, 2.4 mmol), and N$^{\alpha}$-acetyl-1-benzyltryptophan (673 mg, 2.0 mmol). The mixture was stirred until homogenous, then EDC·HCl (460 mg, 2.4 mmol) was added and the reaction mixture warmed to rt and stirred overnight. The reaction was quenched by addition of 1 M aqueous HCl (10 mL) and extracted with CH_2Cl_2 (3 × 10 mL). The combined organic portions were washed with sat. aqueous $NaHCO_3$ (50 mL) and the resulting aqueous layer phase was extracted with CH_2Cl_2 (3 × 10 mL). The combined organic portions were dried over $MgSO_4$, filtered, and concentrated *in vacuo*. Purification by column chromatography (silica gel; 2-5% MeOH in CH_2Cl_2) afforded the product as a colourless solid (481 mg, 1.18 mmol, 59%).

^{1}H NMR (400 MHz, DMSO-d$_6$): δ 8.46 (app t, *J* = 5.9 Hz, 1H), 8.08 (d, *J* = 8.3 Hz, 1H), 7.70 – 7.54 (m, 1H), 7.37 (d, *J* = 8.3 Hz, 1H), 7.32 – 7.17 (m, 4H), 7.14 (dd, *J* = 6.9, 1.6 Hz, 2H), 7.07 (ddd, *J* = 8.2, 7.0, 1.2 Hz, 1H), 7.00 (ddd, *J* = 7.8, 7.1, 1.1 Hz, 1H), 5.35 (s, 2H), 4.58 (app td, *J* = 9.0, 4.8 Hz, 1H), 3.84 (d, *J* = 5.9 Hz, 1H), 3.62 (s, 3H), 3.14 (dd, *J* = 14.6, 4.8 Hz, 1H), 2.88 (dd, *J* = 14.6, 9.4 Hz, 1H), 1.75 (s, 3H).

^{13}C{^{1}H} NMR (101 MHz, $CDCl_3$): δ 172.2, 170.2, 169.0, 138.4, 135.9, 128.4, 127.9, 127.4, 127.2, 126.9, 121.1, 118.8, 118.5, 110.2, 109.9, 53.0, 51.7, 48.9, 27.8, 22.5.

ν_{max} (neat) / cm^{-1}: 3300, 1751, 1632, 1545, 1468, 1359, 1199, 1176.

HRMS: calcd. for $C_{23}H_{26}N_3O_4$ [M+H]$^+$: 408.1918; found (ESI$^+$): 408.1924.

m.p. / °C: 116-117.

1-Benzylpyrrole

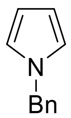

Synthesised according to **GP-3** from pyrrole (2.01 g, 30 mmol), NaH (60% in mineral oil; 1.32 g, 33 mmol), and benzyl bromide (3.6 mL, 30 mmol). Purification by column chromatography (silica gel; CyH) afforded the product as a yellow oil (2.64 g, 16.8 mmol, 56%).

^{1}H NMR (400 MHz, CDCl$_3$): δ 7.37 – 7.27 (m, 3H), 7.17 – 7.09 (m, 2H), 6.71 (app q, *J* = 1.9 Hz, 2H), 6.21 (app dt, *J* = 3.5, 1.9 Hz, 2H), 5.08 (s, 2H).

^{13}C{^{1}H} NMR (101 MHz, CDCl$_3$): δ 138.3, 128.8, 127.7, 127.1, 121.2, 108.6, 53.4.

ν_{max} (neat) / cm^{-1}: 1496, 1453, 1439, 1355, 1301, 1278, 1087, 1067, 967.

HRMS: calcd. for $C_{11}H_{12}N$ [M+H]$^+$: 158.0965; found (ESI$^+$): 158.0967.

Characterisation data are consistent with literature values.[15]

1-Benzyl-2,5-dimethylpyrrole

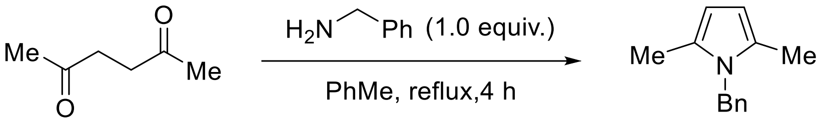

According to literature procedure,[16] 2,5-hexanedione (0.23 mL, 2.0 mmol) and benzylamine (0.22 mL, 2.0 mmol) were dissolved in PhMe (5 mL) and heated to reflux for 4 h. After cooling to rt, the solvent was removed *in vacuo* and the resulting residue purified by column chromatography (silica gel; 2% EtOAc in CyH) to afford the product as a colourless solid (308 mg, 1.42 mmol, 71%).

^{1}H NMR (400 MHz, $CDCl_3$): δ 7.35 – 7.19 (m, 3H), 6.89 (d, *J* = 7.0 Hz, 2H), 5.87 (s, 2H), 5.02 (s, 2H), 2.15 (s, 6H).

^{13}C{^{1}H} NMR (101 MHz, $CDCl_3$): δ 138.7, 128.8, 128.2, 127.1, 125.8, 105.5, 46.9, 12.6.

HRMS: calcd. for $C_{13}H_{16}N$ $[M+H]^+$: 186.1277; found (ESI^+): 186.1267.

ν_{max} (neat) / cm^{-1}: 1658, 1494, 1447, 1406, 1355, 1302.

m.p. / °C: 46-47.

Characterisation data are consistent with literature values.[17]

1-Benzylpyrrole-3-methyl ester

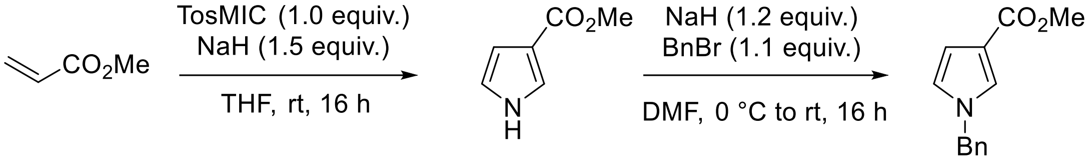

Step 1: A solution of methyl acrylate (0.45 mL, 5.0 mmol) and TosMIC (976 mg, 5.0 mmol) in anhydrous THF (5 mL) was added drop-wise to a flame-dried flask containing a suspension of *t*BuOK (1.12 g, 10 mmol) in anhydrous THF (5 mL). The resulting suspension was stirred for 1 hr at rt. The reaction was quenched by addition of water (10 mL), then EtOAc (20 mL) was added. The organic phase was separated and the aqueous phase was extracted with EtOAc (3 × 10 mL). The combined organic portions were washed with brine, dried over $MgSO_4$, filtered, and concentrated *in vacuo*. Purification by column chromatography (silica gel; 20% EtOAc in CyH) afforded the product as a yellow oil (130 mg, 1.04 mmol, 21%).

^{1}H NMR (400 MHz, $CDCl_3$): δ 8.48 (br s, 1H), 7.44 (app dt, *J* = 3.3, 1.6 Hz, 1H), 6.76 (app q, *J* = 2.4 Hz, 1H), 6.66 (app td, *J* = 2.8, 1.6 Hz, 1H), 3.82 (s, 3H).

Step 2: Synthesised according to **GP-3** from pyrrole-3-methyl ester (188 mg, 1.5 mmol), NaH (72 mg, 1.8 mmol), and benzyl bromide (0.20 mL, 1.65 mmol). Purification by column chromatography (silica gel; 10% EtOAc in CyH) afforded the product as a yellow oil (203 mg, 0.942 mmol, 63%).

^{1}H NMR (400 MHz, $CDCl_3$): δ 7.38 – 7.28 (m, 4H), 7.17 – 7.11 (m, 2H), 6.65 – 6.59 (m, 2H), 5.05 (s, 2H), 3.79 (s, 3H).

^{13}C{^{1}H} NMR (101 MHz, $CDCl_3$): δ 165.3, 136.7, 129.0, 128.2, 127.3, 126.4, 122.2, 116.2, 110.5, 53.9, 51.1.

ν_{max} (neat) / cm^{-1}: 2947, 1697, 1539, 1543, 1440, 1362, 1218, 181, 1112, 991, 923.

HRMS: calcd. for $C_{13}H_{14}NO_2$ $[M+H]^+$: 216.1019; found (ESI^+): 216.1024.

Characterisation data are consistent with literature values.[18]

2-Benzyl-2,5,6,7-tetrahydro-4*H*-isoindol-4-one

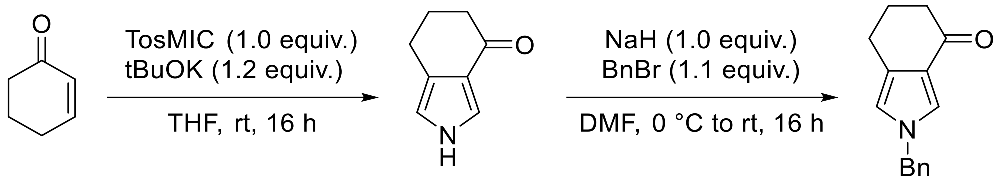

Step 1: According to literature procedure,[19] a solution of 2-cyclohexenone (0.48 mL, 5.0 mmol) and TosMIC (976 mg, 5.0 mmol) in anhydrous THF (10 mL) was added drop-wise to a flame-dried flask containing a suspension of *t*BuOK (673 mg, 6.0 mmol) in anhydrous THF (10 mL). The resulting suspension was stirred overnight at rt. The reaction was quenched by addition of water (20 mL), then EtOAc (20 mL) was added. The organic layer was separated and the aqueous layer extracted with EtOAc (3 × 20 mL). The combined organic portions were washed with brine, dried over $MgSO_4$, filtered, and concentrated *in vacuo*. Purification by column chromatography (silica gel; 50% EtOAc in CyH) afforded the product as a yellow oil (249 mg, 1.84 mmol, 37%).

^{1}H NMR (400 MHz, $CDCl_3$): δ 8.83 (br s, 1H), 7.37 (dd, *J* = 3.2, 1.9 Hz, 1H), 6.56 (br s, *J* = 2.1, 1.1 Hz, 1H), 2.72 (td, *J* = 6.2, 1.1 Hz, 2H), 2.49 (dd, *J* = 7.0, 5.6 Hz, 2H), 2.15 – 1.97 (m, 2H).

^{13}C{^{1}H} NMR (101 MHz, $CDCl_3$): δ 196.4, 126.5, 122.1, 119.6, 113.8, 39.5, 25.2, 21.6.

Step 2: Carried out according to **GP-3** employing 2,5,6,7-tetrahydro-4*H*-isoindol-4-one (135 mg, 1.0 mmol), NaH (40 mg, 1.0 mmol), and BnBr (188 mg, 1.1 mmol). Purification by column chromatography (30% EtOAc in CyH) afforded the product as a colourless solid (109 mg, 0.482 mmol, 48%).

^{1}H NMR (400 MHz, $CDCl_3$): δ 7.38 – 7.30 (m, 3H), 7.27 (d, *J* = 2.0 Hz, 1H), 7.20 – 7.13 (m, 2H), 6.41 (d, *J* = 2.0 Hz, 1H), 5.01 (s, 2H), 2.66 (t, *J* = 6.5 Hz, 1H), 2.58 – 2.36 (m, 1H), 2.04 (app p, *J* = 6.2 Hz, 1H).

^{13}C{^{1}H} NMR (101 MHz, $CDCl_3$): δ 195.8, 136.5, 129.1, 128.4, 127.8, 127.7, 122.4, 122.2, 117.2, 54.1, 39.4, 25.3, 21.8.

ν_{max} (neat) / cm^{-1}: 1645, 1518, 1453, 1380, 1250, 1242, 1174, 1135, 1002, 898.

HRMS: calcd. for $C_{15}H_{15}NONa$ [M+Na]$^+$: 248.1046; found (ESI$^+$): 248.1055.

m.p. / °C: 77-78.

Methyl 1-benzyl-4-phenyl-1*H*-pyrrole-3-carboxylate

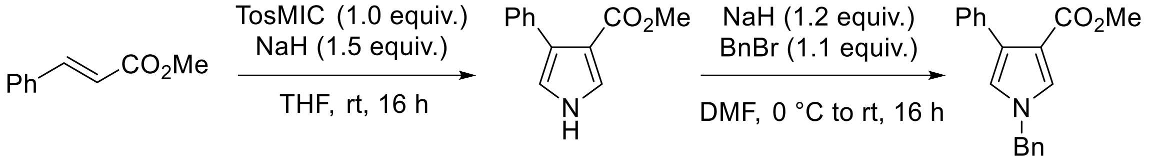

Step 1: A solution of methyl cinnamate (811 mg, 5.0 mmol) and TosMIC (976 mg, 5.0 mmol) in anhydrous THF (10 mL) was added drop-wise to a flame-dried flask containing a suspension of NaH (60% in mineral oil; 300 mg, 7.5 mmol) in anhydrous THF (10 mL). The resulting suspension was stirred overnight at rt. The reaction was quenched by addition of water (20 mL), then EtOAc (20 mL) was added. The organic pahse was separated and the aqueous phase was extracted with EtOAc (3 × 20 mL). The combined organic portions were washed with brine, dried over $MgSO_4$, filtered, and concentrated *in vacuo*. Purification by column chromatography (silica gel; 30% EtOAc in CyH) afforded the product as a yellow oil (621 mg, 2.59 mmol, 52%).

^{1}H NMR (400 MHz, $CDCl_3$): δ 8.53 (brs, 1H), 7.56 – 7.42 (m, 3H), 7.39 – 7.33 (m, 2H), 7.31 – 7.27 (m, 1H), 6.78 (app t, *J* = 2.4 Hz, 1H), 3.74 (s, 3H).

^{13}C{^{1}H} NMR (101 MHz, $CDCl_3$): δ 165.3, 134.8, 129.4, 127.9, 127.0, 126.7, 125.5, 118.4, 113.7, 51.1.

Characterisation data are consistent with literature values.[20]

Step 2: Carried out according to **GP-3** employing methyl 4-phenyl-1*H*-pyrrole-3-carboxylate (402 mg, 1.5 mmol), NaH (60% in mineral oil; 72 mg, 1.8 mmol), and BnBr (0.18 mL, 1.65 mmol). Purification by column chromatography (silica gel; 5% EtOAc in CyH) afforded the product as a colourless oil (218 mg, 0.749 mmol, 49%).

^{1}H NMR (400 MHz, $CDCl_3$): δ 7.52 – 7.47 (m, 2H), 7.40 (d, *J* = 2.5 Hz, 1H), 7.39 – 7.30 (m, 5H), 7.29 – 7.24 (m, 1H), 7.24 – 7.19 (m, 2H), 6.68 (d, *J* = 2.5 Hz, 1H), 5.07 (s, 1H), 3.72 (s, 2H).

^{13}C{^{1}H} NMR (101 MHz, $CDCl_3$): δ 165.1, 136.5, 134.7, 129.3, 129.1, 128.4, 128.3, 127.9, 127.6, 127.5, 126.6, 121.7, 113.3, 54.1, 50.9.

ν_{max} (neat) / cm^{-1}: 1707, 1521, 1446, 1386, 1271, 1164, 1101, 994, 941.

HRMS: calcd. for $C_{19}H_{18}NO_2$ [M+H]$^+$: 292.1332; found (ESI$^+$): 292.1333.

1-Benzyl-3-phenylpyrrole

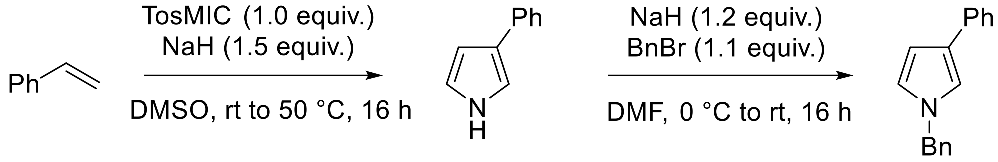

Step 1: According to literature procedure,[21] a solution of styrene (0.57 mL, 5.0 mmol) and TosMIC (1.27 g, 6.5 mmol) in anhydrous THF (12 mL) was added drop-wise to a flame-dried flask containing a solution of *t*BuOK (961 mg, 10 mmol) in anhydrous DMSO (12 mL). The resulting solution was heated to 50 °C and stirred overnight. The reaction was quenched by addition of water (20 mL), then EtOAc (20 mL) was added. The organic phase was separated and the aqueous phase was extracted with EtOAc (3 × 20 mL). The combined organic portions were washed with brine, dried over Na_2SO_4, filtered, and concentrated *in vacuo*. Purification by column chromatography (silica gel; 10% EtOAc in CyH) afforded the product as a colourless solid (214 mg, 1.49 mmol, 30%).

^{1}H NMR (400 MHz, $CDCl_3$): δ 8.25 (brs, 1H), 7.60 – 7.52 (m, 2H), 7.40 – 7.31 (m, 2H), 7.22 – 7.15 (m, 1H), 7.10 (app dt, *J* = 2.7, 1.9 Hz, 1H), 6.85 (app td, *J* = 2.7, 1.9 Hz, 1H), 6.56 (app td, *J* = 2.7, 1.6 Hz, 1H).

Characterisation data are consistent with literature values.[21]

Step 2: Carried out according to **GP-3** employing methyl 3-phenylpyrrole (143 mg, 1.0 mmol), NaH (60% in mineral oil, 48 mg, 1.2 mmol), and BnBr (0.13 mL, 1.1 mmol). Purification by column chromatography (silica gel; CyH) afforded the product as a white solid (131 mg, 0.561 mmol, 56%).

^{1}H NMR (400 MHz, $CDCl_3$): δ 7.55 – 7.47 (m, 2H), 7.38 – 7.27 (m, 5H), 7.22 – 7.11 (m, 3H), 6.99 (app t, *J* = 2.0 Hz, 1H), 6.71 (app t, *J* = 2.5 Hz, 1H), 6.50 (dd, *J* = 2.8, 1.8 Hz, 1H), 5.09 (s, 2H).

^{13}C{^{1}H} NMR (101 MHz, $CDCl_3$): δ 138.0, 136.0, 128.9, 128.7, 127.9, 127.2, 125.5, 125.4, 125.1, 122.4, 118.1, 106.8, 53.7.

ν_{max} (neat) / cm^{-1}: 1701, 1602, 1494, 1449, 1353, 1265, 1072, 1028.

HRMS: calcd. for $C_{17}H_{16}N$ $[M+H]^+$: 234.1278; found (ESI^+): 234.1285.

Characterisation data are consistent with literature values.[15]

1-Benzyl-2-methylpyrrole

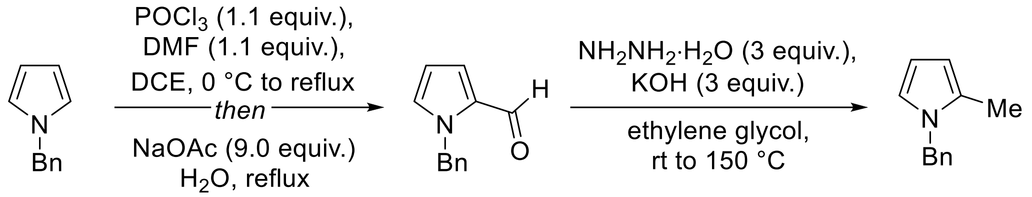

Step 1: $POCl_3$ (206 µL, 2.2 mmol) was added drop-wise to a flask containing DMF (171 µL, 2.2 mmol) cooled to 0 °C to give a white solid. 1,2-DCE (2.5 mL) was then added and the mixture was warmed to rt until full dissolution was observed. A solution of *N*-benzylpyrrole (346 µL, 2.0 mmol) in 1,2-DCE (2.5 mL) was added drop-wise, then heated to reflux and stirred for 15 mins. After cooling to rt, a solution of NaOAc (1.48 g, 18 mmol) in water (9 mL) was added and the mixture was heated to reflux for a further 10 mins. After cooling to rt once more, the reaction mixture was diluted with Et_2O (20 mL) and the aqueous phase was removed. The organic phase was washed with sat. aqueous $NaHCO_3$ (20 mL), dried over $MgSO_4$, filtered, and concentrated *in vacuo*. Purification by column chromatography (silica gel; 10% EtOAc in CyH) afforded the product as a yellow liquid (212 mg, 1.14 mmol, 57%).

Step 2: To a mixture of 1-benzyl-2-formylpyrrole (212 mg, 1.14 mmol) in ethylene glycol (4.5 mL) was added KOH (202 mg, 3.6 mmol) and hydrazine hydrate (187 µL, 3.6 mmol) after which the mixture was stirred for 30 mins at rt to give a pale green colour. The reaction mixture was then heated to 150 °C and stirred for 2 h. After cooling to rt, water (10 mL) was added followed by Et_2O (10 mL). The organic phase was separated and the aqueous layer was extracted with Et_2O (3 × 10 mL). The combined organic portions were washed with brine, dried over Na_2SO_4, filtered, and concentrated *in vacuo* to give a yellow oil that was used without further purification (195 mg, 1.14 mmol, 99%).

^{1}H NMR (400 MHz, $CDCl_3$): δ 7.37 – 7.25 (m, 3H), 7.03 (m, 2H), 6.66 (dd, *J* = 2.8, 1.9 Hz, 1H), 6.14 (app t, *J* = 3.1 Hz, 1H), 5.97 (ddd, *J* = 3.5, 1.9, 1.0 Hz, 1H), 5.06 (s, 2H), 2.17 (s, 3H).

^{13}C NMR (101 MHz, $CDCl_3$): δ 138.6, 128.9, 128.8, 127.4, 126.5, 121.0, 107.2, 77.2, 50.5, 12.1.

ν_{max} (neat) / cm^{-1}: 1493, 1452, 1420, 1355, 1299, 1074, 1028.

HRMS: calcd. for $C_{15}H_{18}N_3$ $[M+C_3H_5N_2]^+$: 240.1495; found (ESI$^+$): 240.1489.

1-Benzyl-2-isopropylpyrrole

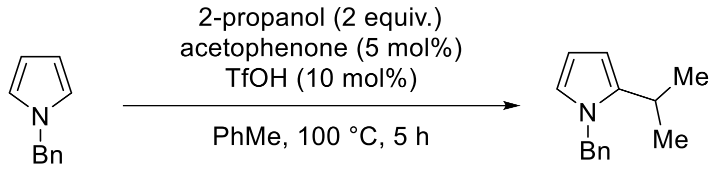

According to literature procedure,[22] to a solution of *N*-benzylpyrrole (314 mg, 2.0 mmol), acetophenone (12.0 mg, 5 mol%), and 2-propanol (305 µL, 4.0 mmol) in PhMe (10 mL) was added TfOH (18 µL, 10 mol%) at rt. The resulting red solution was heated to 100 °C and stirred for 5 hrs. After cooling to rt, the reaction was diluted with EtOAc (10 mL) and washed with sat. aqueous $NaHCO_3$ (20 mL).The aqueous phase was extracted with EtOAc (3 × 10 mL), then the combined organic portions were washed with brine, dried over $MgSO_4$, filtered, and concentrated *in vacuo*. Purification by column chromatography (silica gel; CyH) afforded the product as a yellow oil (148 mg, 0.741 mmol, 37%).

^{1}H NMR (400 MHz, $CDCl_3$): δ 7.40 – 7.29 (m, 3H), 7.19 – 7.11 (m, 2H), 6.63 (app t, *J* = 2.5 Hz, 1H), 6.51 – 6.46 (m, 1H), 6.10 (dd, *J* = 2.5, 1.8 Hz, 1H), 5.03 (s, 2H), 2.85 (hept, *J* = 6.8 Hz, 1H), 1.23 (d, *J* = 6.8 Hz, 6H).

^{13}C{^{1}H} NMR (101 MHz, $CDCl_3$): δ 138.5, 132.4, 128.8, 127.7, 127.2, 121.0, 117.1, 107.1, 53.5, 26.6, 24.2.

ν_{max} (neat) / cm^{-1}: 2956, 1497, 1453, 1358, 1294, 1176, 1076.

HRMS: calcd. for $C_{14}H_{17}NNa$ [M+Na]$^+$: 222.1253; found (ESI$^+$): 222.1246.

Characterisation data are consistent with literature values.[22]

2-(*tert*-Butoxycarbonyl)-1-(4-Fluorophenyl)-1,2,3,4-tetrahydropyrrolo[1,2-*a*]pyrazine

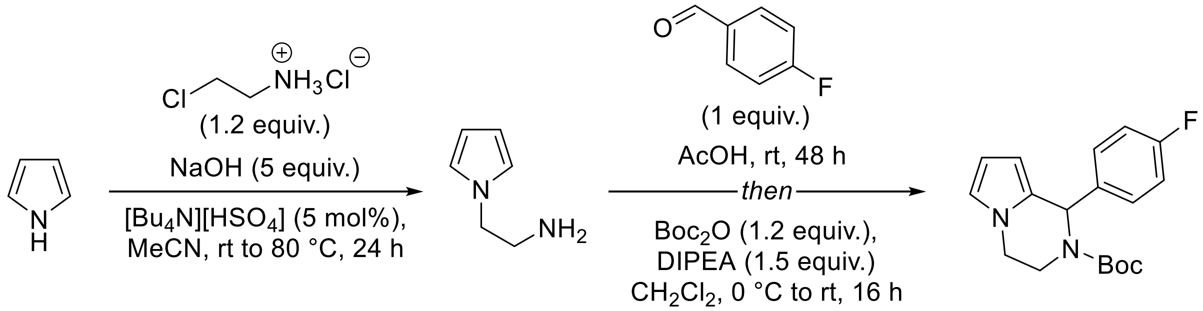

Step 1: To a solution of pyrrole (335 mg, 5.0 mmol) and tetrabutylammonium hydrogensulfate (TBAS, 85 mg, 5 mol%) in MeCN (15 mL) was added pulverised NaOH (1.00 g, 25 mmol) in one portion. The resulting mixture was stirred at rt for 30 mins, then 2-chloroethylamine hydrochloride (696 mg, 6.0 mmol) was added in one portion. The reaction mixture was heated to reflux and stirred for 24 h. After cooling to rt, the mixture was poured onto water (50 mL) and extracted with Et_2O (3 × 20 mL). The combined organic portions were dried over $MgSO_4$, filtered, and concentrated *in vacuo* to afford the product as a yellow oil which was used without further purification.

^{1}H NMR (400 MHz, CDCl$_3$): δ 6.71 (app t, *J* = 2.1 Hz, 2H), 6.19 (app t, *J* = 2.1 Hz, 2H), 4.04 – 3.89 (m, 2H), 3.14 – 2.99 (m, 2H), 1.03 (brs, 2H).

Step 2: To a solution of 1-(2-aminoethyl)pyrrole (551 mg, 5.0 mmol) in AcOH (12.5 mL) was added 4-fluorobenzaldehyde (621 mg, 5.0 mmol) in one portion. The mixture was stirred at rt for 48 h, then poured onto sat. aqueous Na_2CO_3 (30 mL) and extracted with CH_2Cl_2 (3 × 10 mL). The combined organic portions were washed with brine, dried over Na_2SO_4, filtered, and concentrated *in vacuo*. Purification by recrystallisation from 2-propanol afforded the product as a beige solid (523 mg, 2.42 mmol, 48%).

^{1}H NMR (400 MHz, CDCl$_3$): δ 7.50 – 7.34 (m, 2H), 7.18 – 6.97 (m, 2H), 6.63 (app t, *J* = 2.2 Hz, 1H), 6.14 (dd, *J* = 3.5, 2.7 Hz, 1H), 5.55 (app dt, *J* = 3.0, 1.4 Hz, 1H), 5.09 (s, 1H), 4.11 (ddd, *J* = 11.7, 10.1, 4.8 Hz, 1H), 4.01 (ddd, *J* = 11.7, 4.3, 3.1 Hz, 1H), 3.38 (ddd, *J* = 12.6, 4.8, 3.1 Hz, 1H), 3.30 (ddd, *J* = 12.6, 10.1, 4.3 Hz, 1H).

^{13}C{^{1}H} NMR (101 MHz, CDCl$_3$): δ 162.5 (d, *J* = 245.6 Hz), 138.9 (d, *J* = 3.2 Hz), 130.7, 130.0 (d, *J* = 8.0 Hz), 119.2, 115.3 (d, *J* = 21.3 Hz), 107.8, 105.0, 58.6, 45.6, 43.3.

^{19}F NMR (376 MHz, CDCl$_3$): δ -114.93 (tt, *J* = 8.6, 5.4 Hz).

HRMS: calcd. for $C_{13}H_{14}FN_2O$ $[M+H]^+$: 217.1136; found (ESI$^+$): 217.1135.

Step 3: To a solution of 1-(4-fluorophenyl)-1,2,3,4-tetrahydropyrrolo[1,2-a]pyrazine (198 mg, 1.0 mmol) and DIPEA (0.26 mL, 1.5 mmol) in CH_2Cl_2 (10 mL) cooled to 0 °C was added Boc_2O (0.28 mL, 1.2 mmol) drop-wise. The reaction mixture was warmed to rt and stirred overnight. The volatiles were removed *in vacuo*. Purification by column chromatography (silica gel; 5% EtOAc in pentane) afforded the product as a colourless solid (274 mg, 0.867 mmol, 87%).

^{1}H NMR (400 MHz, $CDCl_3$): δ 7.23 (dd, *J* = 8.6, 5.5 Hz, 2H), 6.97 (app t, *J* = 8.7 Hz, 2H), 6.64 (dd, *J* = 2.7, 1.7 Hz, 1H), 6.38 (s, 1H), 6.21 (dd, *J* = 3.5, 2.7 Hz, 1H), 5.91 (s, 1H), 4.22 (brs, 1H), 4.02 (td, *J* = 11.6, 4.3 Hz, 1H), 3.94 (ddd, *J* = 12.1, 4.3, 2.5 Hz, 1H), 3.27 (ddd, *J* = 13.7, 11.1, 4.3 Hz, 1H), 1.49 (s, 9H).

^{13}C{^{1}H} NMR (101 MHz, $CDCl_3$): δ 162.1 (d, *J* = 245.9 Hz), 154.2, 138.0 (d, *J* = 3.2 Hz), 129.0 (d, *J* = 8.0 Hz), 126.6, 119.2, 115.1 (d, *J* = 21.4 Hz), 108.4, 105.9, 80.8, 53.8, 44.4, 38.3, 28.5.

^{19}F NMR (376 MHz, $CDCl_3$): δ -115.29 (br s).

ν_{max} (neat) / cm^{-1}: 1682, 1502, 1411, 1287, 1218, 1168, 1149, 1098, 1077, 980, 863.

HRMS: calcd. for $C_{18}H_{21}FN_2O_2$ $[M+H]^+$: 317.1660; found (ESI^+): 317.1678.

m.p. / °C: 68-70.

1-Benzyl-1*H*-pyrazole

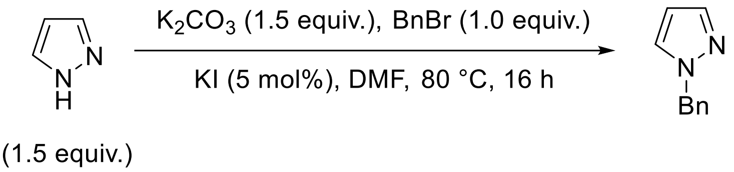

According to the literature procedure,[23] a round-bottom flask was charged with pyrazole (511 mg, 7.5 mmol), K_2CO_3 (1.04 g, 7.5 mmol), KI (42 mg, 0.25 mmol), and DMF (6 mL). BnBr (0.59 mL, 5.0 mmol) was then added and the resulting mixture heated to 80 °C and stirred overnight. After cooling to rt, the reaction was diluted with water (20 mL) and EtOAc (20 mL). The organic layer was separated and the aqueous layer extracted with EtOAc (3 × 20 mL). The combined organic portions were washed with a 10 wt% aqueous LiCl solution (50 mL), dried over Na_2SO_4, filtered, and concentrated *in vacuo*. Purification by column chromatography (silica gel; 5% EtOAc in CyH) afforded the product as a colourless oil (467 mg, 2.94 mmol, 59%).

^{1}H NMR (400 MHz, $CDCl_3$): δ 7.56 (d, *J* = 1.8 Hz, 1H), 7.38 (d, *J* = 2.2 Hz, 1H), 7.37 – 7.27 (m, 3H), 7.25 – 7.18 (m, 2H), 6.28 (app t, *J* = 2.2 Hz, 1H), 5.33 (s, 2H).

^{13}C{^{1}H} NMR (101 MHz, $CDCl_3$): δ 139.6, 136.7, 129.3, 128.8, 128.0, 127.7, 106.0, 55.9.

ν_{max} (neat) / cm^{-1}: 1511, 1495, 1454, 1393, 1359, 1288, 1275, 1087, 1047, 968, 749.

HRMS: calcd. for $C_{10}H_{11}N_2$ $[M+H]^+$: 159.0917; found (ESI^+): 159.0918.

Characterisation data are consistent with literature values.[23]

1-Benzyl-3,5-dimethyl-1*H*-pyrazole

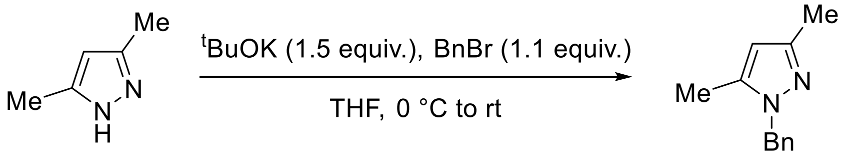

According to the literature procedure,[24] a flame-dried flask under an atmosphere of dinitrogen was charged with 3,5-dimethylpyrazole (341 mg, 5.0 mmol) and anhydrous THF (15 mL). The solution was cooled to 0 °C and ᵗBuOK (842 mg, 7.5 mmol) was added in one portion. After stirring at 0 °C for 30 mins, BnBr (0.65 mL, 5.5 mmol) was added dropwise to afford a white suspension. The reaction mixture was warmed to rt and stirred overnight. Water (10 mL) was added followed by CH_2Cl_2 (30 mL) and the organic layer separated. The aqueous layer was extracted with CH_2Cl_2 (3 × 20 mL). The combined organics were washed with brine (50 mL), dried over Na_2SO_4, filtered, and concentrated *in vacuo*. Purification by column chromatography (10% EtOAc in CyH) afforded the product as a colourless oil (477 mg, 2.56 mmol, 51%).

^{1}H NMR (400 MHz, $CDCl_3$): δ 7.36 – 7.18 (m, 3H), 7.10 – 7.03 (m, 2H), 5.85 (s, 1H), 5.22 (s, 2H), 2.25 (s, 3H), 2.14 (s, 3H).

^{13}C{^{1}H} NMR (101 MHz, $CDCl_3$): δ 147.7, 139.3, 137.6, 128.8, 127.5, 126.7, 105.7, 52.8, 13.7, 11.3.

ν_{max} (neat) / cm^{-1}: 1553, 1495, 1454, 1421, 1383, 1356, 1313, 1027, 776.

HRMS: calcd. for $C_{12}H_{15}N_2$ $[M+H]^+$: 187.1230; found (ESI^+): 187.1324.

Characterisation data are consistent with literature values.[25]

1-Benzyl-3,5-diethyl-1*H*-pyrazole

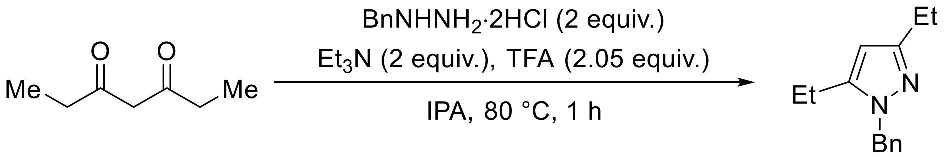

3,5-Heptanedione (256 mg, 2.0 mmol) and benzylhydrazine dihydrochloride (780 mg, 4.0 mmol) were dissolved in IPA (10 mL). Et_3N (0.56 mL, 4.0 mmol) was added and the mixture solidified. TFA (0.32 mL, 2.1 mmol) was then added drop-wise and the resulting solution stirred at 80 °C for 1 h. After cooling to rt, the volatiles were removed *in vacuo* and sat. aqueous $NaHCO_3$ (10 mL) and EtOAc (10 mL) were added. The organic layer was separated and the aqueous layer was extracted with EtOAc (3 × 10 mL). The combined organic portions were dried over Na_2SO_4, filtered, and concentrated *in*

vacuo. Purification by column chromatography (silica gel; 10% EtOAc in CyH) afforded the product as a yellow oil (282 mg, 1.32 mmol, 66%).

^{1}H NMR (400 MHz, CDCl$_3$: δ 7.33 – 7.19 (m, 3H), 7.09 – 7.00 (m, 2H), 5.91 (s, 1H), 5.23 (s, 2H), 2.65 (q, *J* = 7.6 Hz, 2H), 2.47 (q, *J* = 7.6 Hz, 2H), 1.26 (t, *J* = 7.6 Hz, 3H), 1.17 (t, *J* = 7.6 Hz, 3H).

^{13}C{^{1}H} NMR (101 MHz, CDCl$_3$): δ 153.9, 145.4, 137.7, 128.7, 127.4, 126.6, 102.1, 52.6, 21.7, 19.0, 14.2, 12.7.

ν_{max} (neat) / cm^{-1}: 1547, 1496, 1472, 1454, 1378, 1355, 1298, 1055, 1029, 804.

HRMS: calcd. for $C_{14}H_{19}N_2$ $[M+H]^+$: 215.1543; found (ESI$^+$): 215.1549.

Characterisation data are consistent with literature values.[26]

1-Benzyl-3,4,5-trimethyl-1*H*-pyrazole

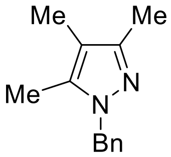

Prepared according to the above procedure from 3-methylpentane-2,4-dione (228 mg, 2.0 mmol). Purification by column chromatography (silica gel; 5% EtOAc in CyH) afforded the product as a colourless oil (366 mg, 1.83 mmol, 91%).

^{1}H NMR (400 MHz, CDCl$_3$): δ 7.31 – 7.20 (m, 3H), 7.12 – 7.00 (m, 2H), 5.20 (s, 2H), 2.19 (s, 3H), 2.05 (s, 3H), 1.91 (s, 3H).

^{13}C{^{1}H} NMR (101 MHz, CDCl$_3$): δ 146.4, 137.8, 136.0, 128.8, 127.5, 126.7, 112.0, 52.9, 12.0, 9.8, 8.3.

ν_{max} (neat) / cm^{-1}: 2918, 1579, 1494, 1471, 1453, 1437, 1385, 1372, 1355, 1319, 1120, 1028.

HRMS: calcd. for $C_{13}H_{17}N_2$ $[M+H]^+$: 201.1387; found (ESI$^+$): 201.1392.

1-Benzyl-1*H*-indazole and 2-Benzyl-2*H*-indazole

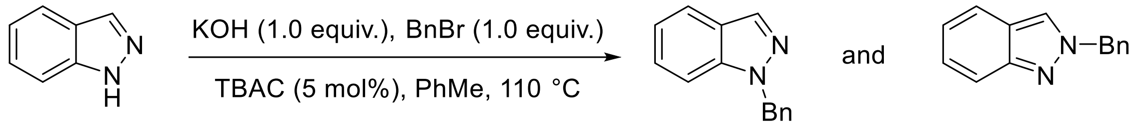

According to the literature procedure,[23] a round-bottom flask was charged with indazole (156 mg, 1.32 mmol), BnBr (0.16 mL, 1.32 mmol), TBAC (18.1 mg, 0.065 mmol), and PhMe (7 mL). Pulverised KOH (73 mg, 1.32 mmol) was then added and the reaction mixture heated to 110 °C and stirred overnight. After cooling to rt, the mixture was poured onto water (10 mL) and EtOAc (10 mL) was then added. The organic layer was separated and the aqueous layer extracted with EtOAc (3 × 10 mL). The combined organic portions were washed with brine (30 mL), dried over Na_2SO_4, filtered, and concentrated *in vacuo*. Purification by column chromatography (10% EtOAc in CyH) afforded 1-benzyl-1*H*-indazole as a yellow oil (91 mg, 0.437 mmol, 33%) and 2-benzyl-2*H*-indazole as a yellow oil (86.8 mg, 0.417 mmol, 32%).

1-Benzyl-1*H*-indazole:

^{1}H NMR (400 MHz, $CDCl_3$): δ 8.05 (d, *J* = 0.7 Hz, 1H), 7.75 (app dt, *J* = 8.0, 1.0 Hz, 1H), 7.39 – 7.24 (m, 5H), 7.23 – 7.18 (m, 2H), 7.15 (ddd, *J* = 8.0, 5.8, 1.9 Hz, 1H), 5.61 (s, 2H).

^{13}C{^{1}H} NMR (101 MHz, $CDCl_3$): δ 139.7, 137.0, 133.5, 128.9, 127.9, 127.3, 126.5, 124.5, 121.3, 120.8, 109.4, 53.1

ν_{max} (neat) / cm^{-1}: 1615, 1496, 1463, 1454, 1420, 1355, 1314, 1295, 1247, 1004, 908, 828.

HRMS: calcd. for $C_{14}H_{13}N_2$ $[M+H]^+$: 209.1073; found (ESI^+): 209.1078.

Characterisation data are consistent with literature values.[23]

2-Benzyl-2*H*-indazole:

^{1}H NMR (400 MHz, $CDCl_3$): δ 7.89 (d, *J* = 1.0 Hz, 1H), 7.73 (app dq, *J* = 8.8, 1.0 Hz, 1H), 7.63 (app dt, *J* = 8.5, 1.1 Hz, 1H), 7.39 – 7.27 (m, 6H), 7.08 (ddd, *J* = 8.5, 6.6, 0.9 Hz, 1H), 5.61 (s, 2H).

^{13}C{^{1}H} NMR (101 MHz, $CDCl_3$): δ 149.1, 135.9, 129.1, 128.5, 128.1, 126.1, 123.0, 122.3, 121.9, 120.3, 117.7, 57.7.

ν_{max} (neat) / cm^{-1}: 1625, 1512, 1451, 1387, 1354, 1294, 1139, 1123, 1073, 1025, 734, 712.

HRMS: calcd. for $C_{14}H_{13}N_2$ $[M+H]^+$: 209.1073; found (ESI^+): 209.1079.

Characterisation data are consistent with literature values.[23]

2.4. General Procedure 4 (GP-4): Synthesis of Arylchlorodiazirines

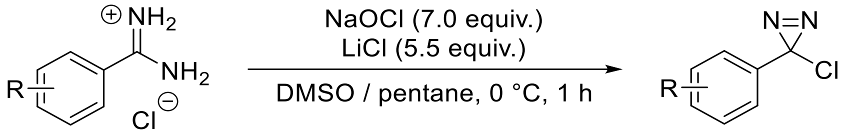

CAUTION! *As indicated by the DSC data in the manuscript, arylchlorodiazirines are thermally and photolytically unstable and are potentially explosive at temperatures at and above ambient. Any operations involving the use or isolation of these diazirines should be performed behind a blast shield and shielded from light.*

According to a modified literature procedure,[27] a two-necked round-bottom flask fitted with a thermometer and a pressure-equalising dropping funnel was charged with DMSO (18 mL) followed by LiCl (1.17 g, 27.5 mmol) and amidine hydrochloride (5.0 mmol). The solution was cooled to 0 °C by use of an external ice-water bath, then pentane (10 mL) was added. A solution of aqueous sodium hypochlorite (0.48 M, 73 mL, 35 mmol) saturated with NaCl was added drop-wise from the addition funnel at a rate that maintained the temperature below 30 °C. After complete addition, the resulting mixture was stirred at 0 °C for a further 1 hr and then poured onto ice-cold water (50 mL). The pentane layer was separated, and the aqueous layer was extracted with Et_2O (3 × 20 mL). The combined organic portions were washed with water (2 × 20 mL) and dried over $MgSO_4$, filtered and concentrated *in vacuo* by rotary evaporation (bath temperature 25 °C and the flask shielded from light with aluminium foil behind a blast shield; see below). Purification by column chromatography (silica gel) eluting with solvents listed afforded the pure product after additional careful rotary evaporation. The diazirines were typically isolated >95% pure with the remaining mass balance being residual solvent, and were stored in a freezer (-20 °C).

Arylchlorodiazirines are known to be energetic materials. Therefore, we recommend the following safety precautions when isolating the pure diazirine *via* rotary evaporation.

- Set cooling bath to 25 °C
- Avoid pressures below 100 mbar
- Cover RBF in elastic mesh to contain fragments in case of explosion
- Shield flask from light with aluminium foil
- Employ a blast shield at all times, and work with the fume-hood sash lowered

3-Chloro-3-phenyl-3*H*-diazirine (1)

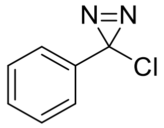

Synthesised according to **GP-4** from benzamidine hydrochloride hydrate (3.13 g, 20.0 mmol), LiCl (4.66 g, 110 mmol), and NaOCl (292 mL, 140 mmol). Purification by column chromatography (silica gel; pentane) afforded a yellow liquid (1.81 g, 11.9 mmol, 59%).

^{1}H NMR (400 MHz, $CDCl_3$): δ 7.48-7.34 (m, 3 H), 7.21-7.05 (m, 2 H).

^{13}C{^{1}H} NMR (101 MHz, $CDCl_3$): δ 135.7, 129.3, 128.5, 126.0, 47.1.

HRMS: calcd. for C_7H_6Cl $[M-N_2+H]^+$: 125.0153; found (ESI^+): 125.0157.

Characterisation data are consistent with literature values.[28]

3-Chloro-3-(4-methylphenyl)-3*H*-diazirine

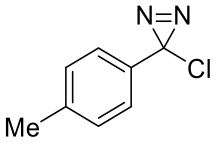

Synthesised according to **GP-4** from 4-methylbenzamidine hydrochloride (853 mg, 5.0 mmol), LiCl (1.16 g, 27.5 mmol), and NaOCl (73 mL, 35 mmol). Purification by column chromatography (silica gel; pentane) afforded a yellow liquid (464 mg, 2.79 mmol, 56%).

^{1}H NMR (400 MHz, $CDCl_3$): δ 7.24 – 7.20 (m, 2H), 7.08 – 6.96 (m, 2H), 2.40 (s, 3H).

^{13}C{^{1}H} NMR (101 MHz, $CDCl_3$): 139.6, 133.0, 129.3, 126.0, 47.3, 21.3.

HRMS: calcd. for C_8H_8Cl $[M-N_2+H]^+$: 139.0309; found (ESI^+): 139.0308.

Characterisation data are consistent with literature values.[28]

3-Chloro-3-(4-fluorophenyl)-3*H*-diazirine

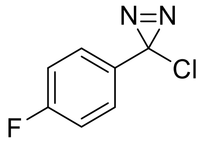

Synthesised according to **GP-4** from 4-fluorobenzamidine hydrochloride (1.75 g, 10 mmol), LiCl (2.33 g, 55 mmol), and NaOCl (146 mL, 70 mmol). Purification by column chromatography (silica gel; pentane) afforded a yellow liquid (1.14 g, 6.65 mmol, 67%).

^{1}H NMR (400 MHz, $CDCl_3$): δ 7.15-7.05 (m, 4H).

^{13}C{^{1}H} NMR (101 MHz, $CDCl_3$): δ 163.3 (d, *J* = 250.2 Hz), 131.6 (d, *J* = 3.2 Hz), 128.0 (d, *J* = 8.9 Hz), 115.7 (d, *J* = 22.3 Hz), 46.6.

^{19}F NMR (376 MHz, $CDCl_3$): δ −111.14 (tt, *J* = 8.9, 5.1 Hz).

HRMS: calcd. for C_7H_5FCl $[M-N_2+H]^+$: 143.0059; found (ESI^+): 143.0049.

Characterisation data are consistent with literature values.[28]

3-Chloro-3-(4-chlorophenyl)-3*H*-diazirine

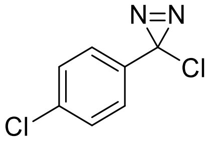

Synthesised according to **GP-4** from 4-chlorobenzamidine hydrochloride (955 mg, 5.0 mmol) LiCl (1.16 g, 27.5 mmol), and NaOCl (73 mL, 35 mmol). Purification by column chromatography (silica gel; pentane) afforded a colourless liquid (320 mg, 1.71 mmol 34%).

^{1}H NMR (400 MHz, $CDCl_3$): δ 7.43 – 7.34 (m, 2H), 7.11 – 6.97 (m, 2H).

^{13}C{^{1}H} NMR (101 MHz, $CDCl_3$): δ 135.8, 134.4, 128.9, 127.5, 46.6.

HRMS: calcd. for $C_7H_5Cl_2$ $[M-N_2+H]^+$: 160.9734; found (ESI^+): 160.9747.

Characterisation data are consistent with literature values.[28]

3-Chloro-3-(4-bromophenyl)-3*H*-diazirine

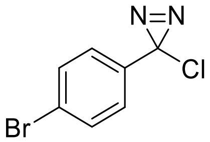

Synthesised according to **GP-4** from 4-bromobenzamidine hydrochloride (1.18 g, 5.0 mmol), LiCl (1.16 g, 27.5 mmol), and NaOCl (73 mL, 35 mmol). Purification by column chromatography (silica gel; pentane) afforded a colourless liquid (246 mg, 1.06 mmol, 21%).

^{1}H NMR (400 MHz, $CDCl_3$): δ 7.60 – 7.51 (m, 2H), 7.05 – 6.95 (m, 2H).

^{13}C{^{1}H} NMR (101 MHz, $CDCl_3$): δ 134.9, 131.9, 127.7, 124.0, 46.7.

HRMS: calcd. for C_7H_5BrCl [M-N_2+H]$^+$: 202.9258; found (ESI$^+$): 202.9253.

Characterisation data are consistent with literature values.[28]

3-Chloro-3-(4-nitrophenyl)-3*H*-diazirine

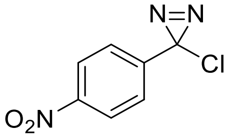

Synthesised according to **GP-4** from 4-nitrobenzamidine hydrochloride (1.01 g, 5.0 mmol), LiCl (1.16 g, 27.5 mmol), and NaOCl (73 mL, 35 mmol). Purification by column chromatography (silica gel; 5% Et_2O in pentane) afforded the product as a yellow solid (227 mg, 1.15 mmol, 23%).

^{1}H NMR (400 MHz, $CDCl_3$): δ 8.26 (d, *J* = 8.9 Hz, 1H), 7.30 (d, *J* = 8.9 Hz, 1H).

^{13}C{^{1}H} NMR (101 MHz, $CDCl_3$): δ 148.5, 142.3, 127.2, 123.9, 46.0.

m.p. / °C: 58-63 (DSC).[i]

Characterisation data are consistent with literature values.[28]

---

[i] Melting point obtained as part of DSC analysis. We strongly discourage obtaining melting points of diazirines using traditional melting point apparatus due to the risk of violent decomposition.

3-Chloro-3-(3-bromophenyl)-3*H*-diazirine

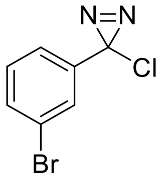

Synthesised according to **GP-4** from 3-bromobenzamidine hydrochloride (1.18 g, 5.0 mmol), LiCl (1.16 g, 27.5 mmol), and NaOCl (73 mL, 35 mmol). Purification by column chromatography (silica gel; pentane) afforded a colourless liquid (446 mg, 1.93 mmol, 39%).

^{1}H NMR (400 MHz, $CDCl_3$): δ 7.53 (ddd, *J* = 7.9, 1.9, 1.0 Hz, 1H), 7.31 (app t, *J* = 1.9 Hz, 1H), 7.26 (app t, *J* = 7.9 Hz, 1H), 7.00 (ddd, *J* = 7.9, 1.9, 1.0 Hz, 1H).

^{13}C{^{1}H} NMR (101 MHz, $CDCl_3$): δ 137.9, 132.6, 130.1, 129.4, 124.7, 123.0, 46.2.

Characterisation data are consistent with literature values.[28]

3-Chloro-3-(2-trifluoromethoxy)-3*H*-diazirine

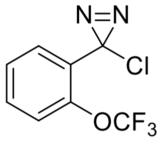

Synthesised according to **GP-4** from 2-(trifluoromethoxy)benzamidine hydrochloride (1.20 g, 10.0 mmol), LiCl (1.16 g, 27.5 mmol), and NaOCl (73 mL, 35 mmol). Purification by column chromatography (silica gel; pentane) afforded a colourless liquid (219 mg, 0.926 mmol, 19%).

^{1}H NMR (400 MHz, $CDCl_3$): δ 7.62 (dd, *J* = 7.7, 0.4 Hz, 1H), 7.46 (ddd, *J* = 8.3, 7.5, 1.7 Hz, 1H), 7.34 (td, *J* = 7.6, 1.2 Hz, 1H), 7.32 – 7.29 (m, 1H).

^{13}C NMR (101 MHz, $CDCl_3$): δ 147.5 (q, *J* = 1.8 Hz), 131.9, 129.4, 128.0, 127.3, 121.4 (q, *J* = 1.6 Hz), 120.5 (q, *J* = 258.9 Hz), 43.1.

^{19}F NMR (376 MHz, $CDCl_3$): δ -57.12 (s).

3-Chloro-3-(2-fluoro-5-chlorophenyl)-3*H*-diazirine

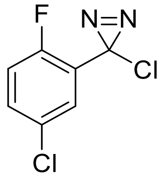

Synthesised according to **GP-4** from 2-fluoro-5-chlorobenzamidine hydrochloride (2.09 g, 10.0 mmol), LiCl (2.33 g, 55 mmol), and NaOCl (146 mL, 70 mmol). Purification by column chromatography (silica gel; pentane) afforded a colourless liquid (1.47 g, 7.18 mmol, 72%).

^{1}H NMR (400 MHz, $CDCl_3$): δ 7.50 (dd, *J* = 6.3, 2.6 Hz, 1H), 7.35 (ddd, *J* = 8.8, 4.2, 2.6 Hz, 1H), 7.04 (dd, *J* = 10.1, 8.8 Hz, 1H).

^{13}C{^{1}H} NMR (101 MHz, $CDCl_3$): δ 159.0 (d, *J* = 254.3 Hz), 132.0 (d, *J* = 8.4 Hz), 129.9 (d, *J* = 3.6 Hz), 129.2 (d, *J* = 2.2 Hz), 124.2 (d, *J* = 12.9 Hz), 118.2 (d, *J* = 22.5 Hz), 42.4.

^{19}F NMR (376 MHz, $CDCl_3$): δ -115.89 (ddd, *J* = 10.1, 6.3, 4.2 Hz).

2-(3-chloro-3*H*-diazirin-3-yl)pyridine

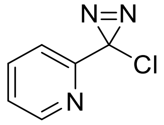

Synthesised according to **GP-4** from 2-pyridine carboximidamide hydrochloride (788 mg, 5.0 mmol), LiCl (1.16 g, 27.5 mmol), and NaOCl (73 mL, 35 mmol). Purification by column chromatography (silica gel; 10% Et_2O in pentane) afforded a yellow liquid (309 mg, 2.01 mmol, 40%).

^{1}H NMR (400 MHz, $CDCl_3$): δ 8.55 (app dt, *J* = 4.8, 1.3 Hz, 1H), 7.82 (td, *J* = 7.9, 1.8 Hz, 1H), 7.72 (app dt, *J* = 7.9, 1.3 Hz, 1H), 7.32 (ddd, *J* = 7.6, 4.8, 1.1 Hz, 1H).

^{13}C{^{1}H} NMR (101 MHz, $CDCl_3$): δ 153.7, 149.2, 137.1, 123.6, 122.8, 47.4.

HRMS: calcd. for C_6H_5NCl $[M-N_2+H]^+$: 126.0106; found (ESI^+): 126.0113.

Characterisation data are consistent with literature values.[28]

2.5. Synthesis of Amidine Hydrochlorides

4-Fluorobenzamidine hydrochloride

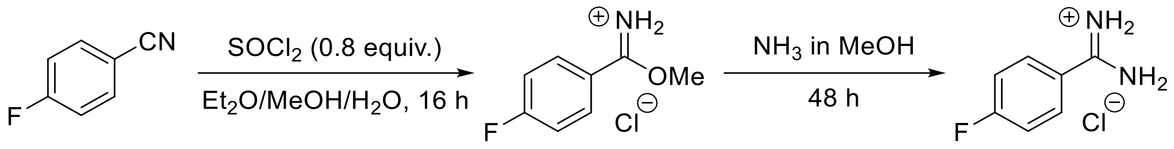

4-Fluorobenzonitrile (3.03 g, 25 mmol) was dissolved in a 6:2:1 mixture of Et_2O/MeOH/water (4 mL) and the solution was cooled to 0 °C by use of an external ice-water bath. $SOCl_2$ (1.46 mL, 20 mmol) was then added drop-wise and the reaction was stirred at 0 °C for 1 h. The reaction was warmed to rt and stirred overnight. The resulting precipitate was isolated by filtration and transferred to a second flask and suspended in MeOH (5 mL). Ammonia (2 M in MeOH; 18.8 mL, 37.5 mmol) was then added. The suspension fully dissolved within 2 h, and the resulting solution was stirred at rt for 48 h. The volatiles were removed *in vacuo* and the resulting solid was washed with Et_2O (3 × 20 mL) to afford the pure product as an off-white solid (3.68 g, 21.1 mmol, 84%).

^{1}H NMR (400 MHz, DMSO-d$_6$): δ 9.23 (br s, 4H), 8.01 – 7.81 (m, 2H), 7.53 – 7.46 (m, 2H).

^{13}C NMR (101 MHz, DMSO-d$_6$): δ 166.4 (d, *J* = 252.7 Hz), 164.72, 131.2 (d, *J* = 9.7 Hz), 124.5 (d, *J* = 3.2 Hz), 116.1 (d, *J* = 22.3 Hz).

^{19}F NMR (376 MHz, DMSO-d$_6$): δ -105.13 (tt, *J* = 8.5, 5.5 Hz).

ν_{max} (neat) / cm^{-1}: 3251, 3052, 1656, 1609, 1491, 1245, 1088, 848.

HRMS: calcd. for $C_7H_8FN_2$ [M-Cl]$^+$: 139.0667; found (ESI$^+$): 139.0663.

m.p. / °C: 210-211.

4-Bromobenzamidine hydrochloride

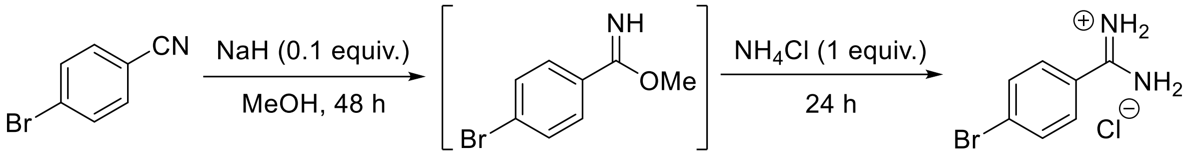

A flame-dried flask under an atmosphere of dinitrogen was charged 4-bromobenzonitrile (1.82 g, 10.0 mmol) and MeOH (30 mL). NaH (60% in mineral oil, 40 mg, 1.0 mmol) was then added in one portion and the reaction was stirred at rt for 48 h. Anhydrous NH_4Cl (535 mg, 10.0 mmol) was added in one portion and the reaction was stirred for a further 24 h. The mixture was filtered and the reaction mixture was concentrated *in vacuo*. The resulting solid was suspended in Et_2O (20 mL) and basified by addition of 2 M NaOH (20 mL). The organic layer was separated and dried over Na_2SO_4, filtered, and the volatiles were removed *in vacuo.* The residue was re-dissolved in Et_2O (10 mL) and HCl (4 M in dioxane, 4 mL, 16 mmol) was added at 0 °C and the suspension stirred at rt for 1 hr. The resulting solid isolated by filtration and washed with Et_2O (2 × 10 mL) to afford the pure product as a colourless solid (1.43 g, 6.09 mmol, 61%).

^{1}H NMR (400 MHz, DMSO-d$_6$): δ 8.92 (s, 4H), 7.82 (d, *J* = 8.6 Hz, 2H), 7.75 (d, *J* = 8.6 Hz, 2H).

^{13}C{^{1}H} NMR (101 MHz, DMSO-d$_6$): δ 162.9, 131.9, 131.4, 129.4, 125.1.

ν_{max} (neat) / cm^{-1}: 3070, 1673, 1594, 1479, 1072, 1012, 842.

HRMS: calcd. for $C_7H_8N_2Br$ [M-Cl]$^+$: 198.9865; found (ESI$^+$): 198.9874.

m.p. / °C: 258 (decomp.)

4-Chlorobenzamidine hydrochloride

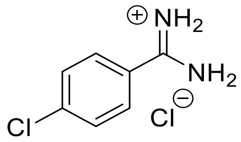

Synthesised according to the above procedure from 4-chlorobenzonitrile (4.13 g, 30 mmol). Colourless solid (3.38 g, 17.8 mmol, 59%).

^{1}H NMR (400 MHz, DMSO-d$_6$): δ 8.75 (s, 4H), 7.84 (app dt, *J* = 6.5, 2.2 Hz, 2H), 7.65 (d, *J* = 8.0 Hz, 2H).

^{13}C{^{1}H} NMR (101 MHz, DMSO-d$_6$): δ 164.0, 137.7, 129.7, 128.81, 128.75.

ν_{max} (neat) / cm^{-1}: 3239, 3123, 1672, 1595, 1482, 1089, 1013, 845.

HRMS: calcd. for $C_7H_8N_2Cl$ [M-Cl]$^+$: 155.0371; found (ESI$^+$): 155.0374.

m.p. / °C: 247-250.

3-Bromobenzamidine hydrochloride

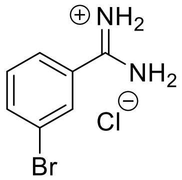

Synthesised according to the above procedure from 3-bromobenzonitrile (1.82 g, 10 mmol). Colourless solid (1.41 g, 6.00 mmol, 60%).

^{1}H NMR (400 MHz, DMSO-d$_6$): δ 9.34 (br s, 4H), 8.05 (app t, *J* = 2.0 Hz, 1H), 7.92 (ddd, *J* = 8.0, 2.0, 1.0 Hz, 1H), 7.84 (app ddt, *J* = 8.0, 2.0, 1.0 Hz, 1H), 7.56 (app t, *J* = 8.0 Hz, 1H).

^{13}C{^{1}H} NMR (101 MHz, DMSO-d$_6$): δ 164.3, 136.2, 131.0, 130.74, 130.66, 127.2, 121.9.

ν_{max} (neat) / cm^{-1}: 3035, 1579, 1516, 1463, 1411, 1078.

HRMS: calcd. for $C_7H_8N_2Br$ [M-Cl]$^+$: 198.9865; found (ESI$^+$): 198.9884.

m.p. / °C: 121-124.

5-Chloro-2-fluorobenzamidine hydrochloride

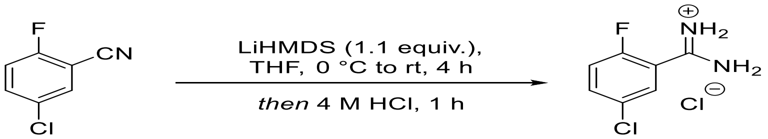

To a flame-dried flask under an atmosphere of dinitrogen was added a solution of LiHMDS (1 M in THF; 22 mL, 22 mmol). The flask was cooled to 0 °C and 5-chloro-2-fluorobenzonitrile (3.11 g, 20 mmol) was added portion-wise over 5 mins. The reaction mixture was warmed to rt and stirred for 4 h. The mixture was cooled to 0 °C and aqueous HCl (1 M; 25 mL, 25 mmol) was added and the reaction was stirred for a further 1 h. EtOAc (20 mL) was added and the organic layer was separated. The aqueous layer was washed with EtOAc (3 × 20 mL), then basified with 2M NaOH until pH > 10. The aqueous solution was extracted with CH_2Cl_2 (3 × 30 mL), and the combined CH_2Cl_2 extracts were dried over Na_2SO_4, filtered, and concentrated *in vacuo* to give an orange oil which solidified over time. The crude material was dissolved in Et_2O (30 mL) and cooled to 0 °C. HCl (4 M in dioxane, 6 mL, 24 mmol) was then added drop-wise to produce a yellow precipitate. The reaction mixture was stirred at rt for 1 h, the solid was isolated by filtration, then washed with Et_2O (2 × 20 mL) to afford the product as a yellow solid (3.15 g, 15.1 mmol, 75%).

^{1}H NMR (400 MHz, DMSO-d$_6$): δ 9.66 (br s, 2H), 9.65 (br s, 2H), 7.87 (dd, *J* = 5.9, 2.7 Hz, 1H), 7.81 (ddd, *J* = 9.0, 4.5, 2.7 Hz, 1H), 7.55 (t, *J* = 9.3 Hz, 1H).

^{13}C{^{1}H} NMR (101 MHz, DMSO-d$_6$): δ 160.9, 158.7 (d, *J* = 252.4 Hz), 134.3 (d, *J* = 8.9 Hz), 129.9 (d, *J* = 2.0 Hz), 128.6 (d, *J* = 3.3 Hz), 119.3 (d, *J* = 14.9 Hz), 118.5 (d, *J* = 22.9 Hz).

^{19}F NMR (376 MHz, DMSO-*d*$_6$): δ -115.70 (app td, *J* = 9.7, 9.0, 4.5 Hz).

ν_{max} (neat) / cm^{-1}: 3034, 2917, 2849, 1674, 1617, 1540, 1476, 1236, 1105.

HRMS: calcd. for $C_7H_7N_2FCl$ [M-Cl]$^+$: 173.0276; found (ESI$^+$): 173.0287.

m.p. / °C: 219-221.

2-(Trifluoromethoxy)benzamidine hydrochloride

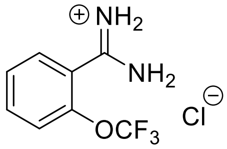

Synthesised as described above from 2-(trifluoromethoxy)benzonitrile (3.74 g, 20 mmol) as a colourless solid (715 mg, 2.97 mmol, 15%).

^{1}H NMR (400 MHz, DMSO-d$_6$): 9.58 (s, 4H), 7.85 – 7.72 (m, 2H), 7.65 – 7.58 (m, 2H).

^{13}C NMR (101 MHz, DMSO-d$_6$): δ 163.0 (q, *J* = 6.0 Hz), 144.8 (q, 1.6 Hz), 134.1, 130.6, 127.9, 123.7 (q, *J* = 2.9 Hz), 121.7, 119.9 (q, *J* = 258.6 Hz).

^{19}F NMR (376 MHz, DMSO-*d*$_6$): δ -56.79

ν_{max} (neat) / cm^{-1}: 3049, 1674, 1477, 1259, 1217, 1158.

HRMS: calcd. for $C_8H_7N_2OF_3$ [M-Cl]$^+$: 205.0583; found (ESI$^+$): 205.0597.

m.p. / °C: 276-278.

6-Fluoro-3-phenylquinolinium hydrochloride

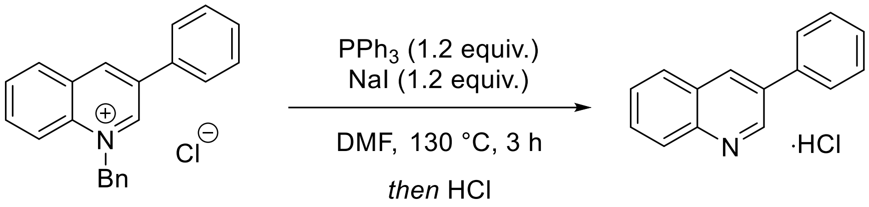

A round bottom flask was charged with 1-benzyl-3-phenylquinolin-1-ium chloride (66 mg, 0.2 mmol), PPh_3 (63 mg, 0.24 mmol), NaI (36 mg, 0.24 mmol) and DMF (2 mL). The solution was heated to 130 °C and stirred for 3 h. After cooling to rt, water (2 mL) and the mixture was poured onto Et_2O (5 mL). The ether layer was separated and the aqueous layer was washed with Et_2O (3 × 5 mL). The combined organic portions were dried over Na_2SO_4, filtered, and concentrated *in vacuo*. Et_2O (5 mL) was then added followed by HCl (4 M in dioxane, 0.1 mL). The resulting precipitate was isolated by filtration to afford the product as a colourless powder (38.1 mg, 0.158 mmol, 79%).

^{1}H-NMR (400 MHz, DMSO-d_6): δ 9.51 (d, *J* = 2.3 Hz, 1H), 9.13 (d, *J* = 1.1 Hz, 1H), 8.29 – 8.21 (m, 2H), 8.02 – 7.94 (m, 3H), 7.84 (ddd, *J* = 8.0, 6.9, 1.1 Hz, 1H), 7.65 – 7.58 (m, 2H), 7.56 – 7.49 (m, 1H).

^{13}C{^{1}H} NMR (101 MHz, DMSO-d_6): δ 145.6, 139.7, 134.9, 133.5, 132.9, 129.4, 129.2, 129.1, 128.3, 127.5, 123.0.

ν_{max} (neat) / cm^{-1}: 2536, 2030, 1571, 1501, 1360, 1328, 1301, 1150, 1030, 900.

HRMS: calcd. for $C_{15}H_{12}N$ [M-Cl]$^+$: 206.0964; found (ESI$^+$): 206.0967.

m.p. / °C: 160 (decomp.)

1-Benzyl-3-phenylquinolin-2(1*H*)-one

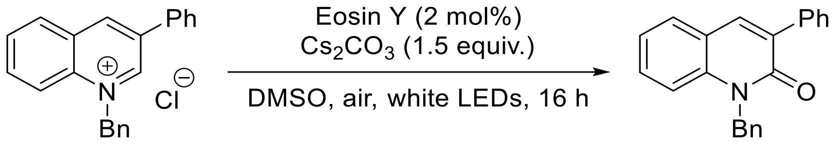

According to literature procedure,[29] a microwave tube was charged with the quinolinium salt (66 mg, 0.2 mmol), Eosin Y (2.8 mg, 0.004 mmol, 2.0 mol%), Cs_2CO_3 (98 mg, 0.3 mmol) and DMSO (2 mL). The reaction mixture was stirred under constant irradiation (white LEDs, 6200K) overnight. The reaction mixture was poured onto EtOAc (10 mL), filtered through a silica plug and washed through with EtOAc. The filtrate was washed with water (10 mL) and the aqueous layer was extracted with EtOAc (3 × 10 mL). The combined organic portions were washed with brine, dried over $MgSO_4$, filtered, and concentrated *in vacuo*. Purification by column chromatography (silica gel; 20% EtOAc in CyH) afforded the product as a colourless solid (37.5 mg, 0.120 mmol, 60%).

^{1}H NMR (400 MHz, $CDCl_3$): δ 7.88 (s, 1H), 7.79 (dd, *J* = 7.2, 1.8 Hz, 2H), 7.63 (dd, *J* = 7.8, 1.5 Hz, 1H), 7.50 – 7.36 (m, 4H), 7.34 – 7.28 (m, 5H), 7.26 – 7.18 (m, 2H), 5.64 (s, 2H).

^{13}C{^{1}H} NMR (101 MHz, $CDCl_3$): δ 161.8, 139.3, 137.5, 136.8, 136.7, 132.5, 130.4, 129.2, 129.1, 128.9, 128.3, 128.3, 127.4, 127.0, 122.4, 121.2, 115.0, 46.7.

ν_{max} (neat) / cm^{-1}: 1633, 1576, 1493, 1448, 1238, 1216, 1183.

HRMS: calcd. for $C_{22}H_{17}NO$ $[M+H]^+$: 312.1383; found (ESI^+): 312.1385.

m.p. / °C: 102-104.

1-Benzyl-3-phenyl-1,2-dihydroquinoline

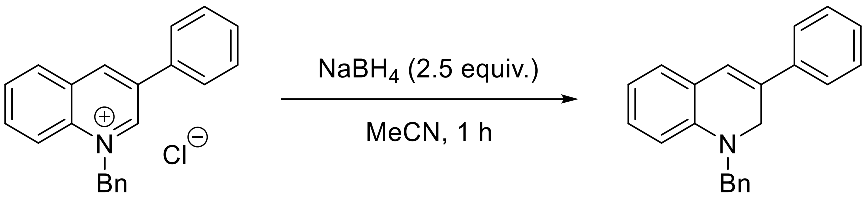

To a suspension of quinolinium salt (66 mg, 0.2 mmol) in MeCN (1 mL) cooled to 0 °C was added $NaBH_4$ (19 mg, 0.5 mmol) in one portion. Dissolution of the solids was accompanied by formation of a fluorescent yellow colour. The reaction was stirred for 1 h, then quenched with sat. aqueous NH_4Cl (5 mL) and diluted with CH_2Cl_2 (5 mL). The CH_2Cl_2 layer was separated and the aqueous layer extracted with CH_2Cl_2 (3 × 10 mL). The combined organic portions were dried over Na_2SO_4, filtered and concentrated *in vacuo* to afford the crude product as a fluorescent yellow oil (51.1 mg, 0.172 mmol, 86%).

^{1}H NMR (400 MHz, $CDCl_3$): δ 7.47 – 7.40 (m, 2H), 7.36 (dt, *J* = 8.2, 5.5 Hz, 6H), 7.32 – 7.24 (m, 2H), 7.03 (app t, *J* = 7.5 Hz, 2H), 6.85 (s, 1H), 6.65 (app t, *J* = 7.5 Hz, 1H), 6.56 (d, *J* = 8.1 Hz, 1H), 4.55 (d, *J* = 1.4 Hz, 2H), 4.54 (s, 2H).

^{13}C{^{1}H} NMR (101 MHz, $CDCl_3$): δ 144.5, 138.0, 137.3, 130.5, 129.3, 128.8, 128.6, 127.9, 127.7, 127.3, 127.2, 124.3, 122.7, 122.4, 117.2, 110.1, 54.0, 52.2.

HRMS: calcd. for $C_{22}H_{20}N$ $[M+H]^+$: 298.1590; found (ESI^+): 298.1591.

3-Phenyl-6-fluoro-1,2,3,4-tetrahydroquinoline

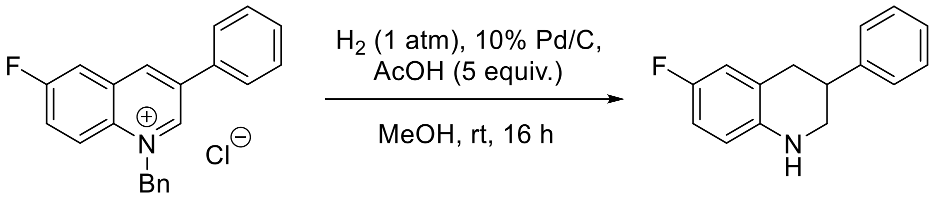

Prior to reaction, MeOH was degassed by sparging with dinitrogen for 30 minutes. An oven-dried round-bottom flask was charged with 1-benzyl-3-phenyl-6-fluoroquinolinium chloride (70 mg, 0.2 mmol) and Pd/C (30 mg), then sealed with a rubber septum. The flask was evacuated and back-filled with dinitrogen three times, then degassed MeOH (2 mL) was added slowly followed by AcOH (57 µL, 1.0 mmol). The flask was then evacuated by use of a water aspirator until boiling of the solvent was observed and then filled with H_2 *via* balloon; this process was repeated five times. The reaction was stirred for 16 h at rt under balloon pressure of hydrogen. The reaction mixture was filtered through Celite and washed with MeOH (10 mL). The solvent was removed *in vacuo*, then water (10 mL) and Et_2O (10 mL) were added. The biphasic mixture was separated, and the organic phase was washed with sat. aqueous $NaHCO_3$ (20 mL). The combined aqueous portions were extracted with Et_2O (3 × 5 mL) and the combined organic portions were dried over Na_2SO_4, filtered, and concentrated *in vacuo*. Purification by column chromatography (silica gel; 10% EtOAc in CyH) afforded the product as a colourless powder (41.8 mg, 0.184 mmol, 92%).

^{1}H NMR (400 MHz, $CDCl_3$): δ 7.44 – 7.33 (m, 2H), 7.32 – 7.24 (m, 3H), 6.76 (m, 2H), 6.51 (dd, *J* = 9.5, 4.8 Hz, 1H), 3.94 (s, 1H), 3.48 (ddd, *J* = 11.2, 3.6, 1.9 Hz, 1H), 3.34 (app t, *J* = 10.7 Hz, 1H), 3.16 (tdd, *J* = 10.0, 5.7, 3.6 Hz, 1H), 3.09 – 2.93 (m, 2H).

^{13}C{^{1}H} NMR (101 MHz, $CDCl_3$): δ 155.7 (d, *J* = 235.1 Hz), 143.6, 140.4 (d, *J* = 1.8 Hz), 128.8, 127.3, 126.9, 122.8 (d, *J* = 6.7 Hz), 115.7 (d, *J* = 21.7 Hz), 115.0 (d, *J* = 7.7 Hz), 113.7 (d, *J* = 22.5 Hz), 48.6, 38.7, 34.7.

^{19}F NMR (376 MHz, $CDCl_3$): δ -128.11 (app td, *J* = 8.8, 4.8 Hz).

ν_{max} (neat) / cm^{-1}: 3390, 1503, 1490, 1236, 1214, 1140, 1098, 1078, 952, 869.

HRMS: calcd. for $C_{15}H_{15}FN$ [M+H]$^+$: 228.1183; found (ESI$^+$): 228.1192.

m.p. / °C: 112-113.

3-Phenyl-1,2,3,4-tetrahydroquinoline

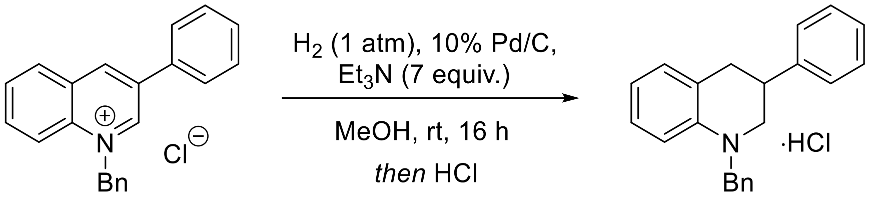

Prior to reaction, MeOH was degassed by sparging with dinitrogen for 30 minutes. An oven-dried round-bottom flask was charged with 1-benzyl-3-phenylquinolinium chloride (66 mg, 0.2 mmol) and Pd/C (30 mg), then sealed with a rubber septum. The flask was evacuated and back-filled with dinitrogen three times, then degassed MeOH (2 mL) was added slowly followed by Et_3N (0.20 mL, 1.4 mmol). The flask was then evacuated by use of a water aspirator until boiling of the solvent was observed and then filled with H_2 *via* balloon; this process was repeated five times. The reaction was then stirred for 16 h under balloon pressure of hydrogen. The reaction mixture was filtered through Celite and eluted with MeOH (10 mL). The solvent was removed *in vacuo* and the resulting residue was taken up in Et_2O. The solids were removed by filtration, then HCl (4 M in dioxane, 0.1 mL) was added to the filtrate. After standing in a fridge (4 °C) overnight, the resulting solid was isolated by filtration to afford the product as a yellow solid (41.6 mg, 0.124 mmol, 62%).

^{1}H NMR (400 MHz, DMSO-d$_6$): δ 7.36 – 7.18 (m, 10H), 6.98 (dd, *J* = 7.4, 1.6 Hz, 1H), 6.96 – 6.89 (m, 1H), 6.56 – 6.48 (m, 2H), 4.59 (d, *J* = 16.9 Hz, 1H), 4.49 (d, *J* = 16.9 Hz, 1H), 3.51 (app q, *J* = 11.3 Hz, 1H), 3.44 (ddd, *J* = 11.3, 4.4, 2.0 Hz, 1H), 3.26 – 3.12 (m, 2H), 3.05 (dd, *J* = 15.5, 11.3 Hz, 1H), 2.92 (ddd, *J* = 15.5, 4.4, 2.0 Hz, 1H).

^{13}C{^{1}H} NMR (101 MHz, DMSO-d$_6$): δ 144.4, 143.4, 138.9, 128.9, 128.4, 128.4, 127.2, 127.0, 126.7, 126.6, 126.5, 121.8, 115.5, 110.8, 55.7, 54.2, 37.7, 34.8.

ν_{max} (neat) / cm^{-1}: 1601, 1495, 1449, 1354, 1336, 1276, 1241, 1112, 1076, 1058, 1022, 962.

HRMS: calcd. for $C_{22}H_{22}N$ [M+H]$^+$**:** 300.1747; found 300.1747.

m.p. / °C: 145 (decomp.)

3-Cyclohexyl-1-(cyclohexylmethyl)-5,6,7,8-tetrahydroquinolin-1-ium trifluoroacetate

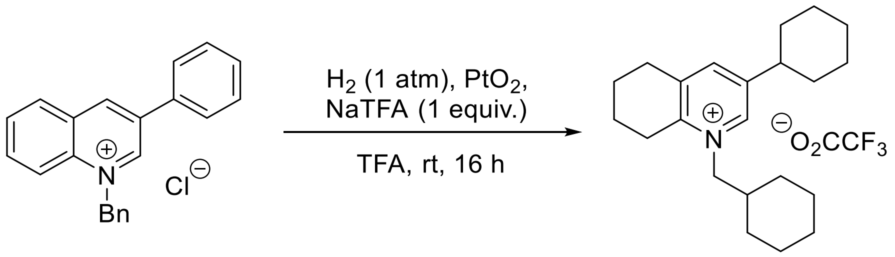

Prior to reaction, trifluoroacetic acid (TFA) was degassed by sparging with dinitrogen for 30 minutes. According to the literature procedure,[30] an oven-dried round-bottom flask was charged with 1-benzyl-3-phenylquinolinium chloride (66 mg, 0.2 mmol), PtO_2 hydrate (10 mg), and sodium trifluoroacetate (27 mg, 0.2 mmol), then sealed with a rubber septum. The flask was evacuated and back-filled with dinitrogen three times, then degassed TFA (1 mL) was added slowly. The flask was evacuated by use of a water aspirator until boiling of the solvent was observed and then filled with H_2 *via* balloon; this process was repeated five times. The reaction was then stirred for 16 hrs at rt under balloon pressure. The reaction mixture was diluted with EtOAc (5 mL) and filtered through Celite. The organics were washed with H_2O (10 mL) and the aqueous layer extracted with EtOAc (3 × 10 mL). The combined organics were dried over Na_2SO_4, filtered, and concentrated *in vacuo* to afford the product as a viscous oil (55.6 mg, 0.131 mmol, 65%).

^{1}H NMR (400 MHz, $CDCl_3$): δ 8.21 (s, 1H), 7.87 (s, 1H), 4.33 (d, *J* = 7.2 Hz, 2H), 3.21 – 2.84 (m, 4H), 2.64 (d, *J* = 10.6 Hz, 1H), 2.15 – 0.98 (m, 25H).

^{13}C{^{1}H} NMR (101 MHz, $CDCl_3$): δ 158.2 (q, *J* = 34.7 Hz), 151.4, 143.7, 143.1, 142.5, 138.6, 116.2 (q, *J* = 294.8 Hz), 61.9, 36.8, 32.8, 32.7, 29.3, 28.2, 26.0, 25.8, 25.6, 25.1, 25.0, 21.2, 20.2.

^{19}F NMR (376 MHz, DMSO-*d*$_6$): δ -74.32 (s).

ν_{max} (neat) / cm^{-1}: 2928, 2856, 1736, 1450, 1180, 1132, 932.

HRMS: calcd. for $C_{22}H_{34}N$ [M-O_2CCF_3]$^+$: 312.2686; found 312.2702.

4. Robustness Screen

4.1. General Procedure for robustness screen (GP-5)

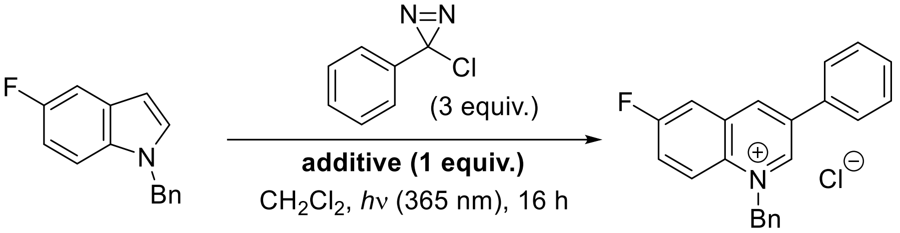

Procedure: A 10 mL microwave tube containing 1-benzyl-5-fluoroindole (46 mg, 0.2 mmol), the additive (if solid; 0.2 mmol) and 4,4′-bis(trifluoromethyl)-1,1′-biphenyl (internal standard for ^{19}F NMR spectroscopy) was sealed, evacuated and backfilled with dinitrogen 3 times. Anhydrous CH_2Cl_2 (2 mL) was added, followed by the additive (if liquid; 0.2 mmol) and 3-chloro-3-phenyldiazirine (92 mg, 0.6 mmol), then an aliquot was removed for initial ^{19}F NMR spectroscopic analysis. The cap of the reaction flask was then sealed with electrical tape, and the reaction mixture was stirred under constant irradiation with UV light (365 nm, 18 W LED, 5 cm from light source) overnight. If solids precipitated, the reaction was diluted with MeOH until homogenous and an aliquot was taken and analysed by ^{19}F NMR spectroscopy. Results are presented in Table S1.

Entry	Additive	Yield of 3 / %	Additive remaining / %
1[a]	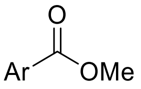	80	100
2	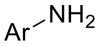	7	7
3	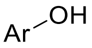	0	71
4	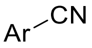	65	100
5	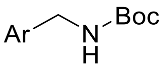	62	78
6	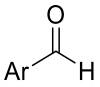	24	0
7	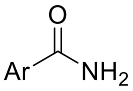	5	46
8	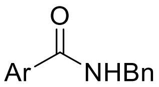	63	96
9	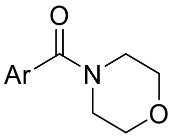	68	100
10	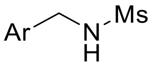	50	90
11	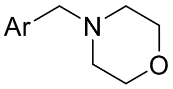	29	17

12	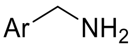	20	3
13	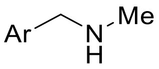	2	23
14	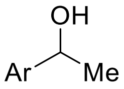	57	87
15	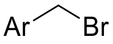	29	85
16	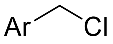	69	100
17	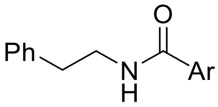	62	100
18	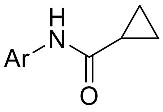	13	0
19	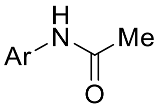	17	0
20	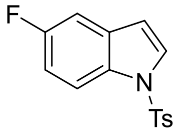	74	88

Supplementary Table S1. Robustness screen of ring expansion reaction. Yields of **3** and remaining additive determined by ^{19}F NMR spectroscopy against internal standard. Ar = 4-F-C_6H_4. [a] Ar = 4-F_3C-C_6H_4.

5. Reaction Optimisation

5.1. Assessment of *N*-Protecting Group

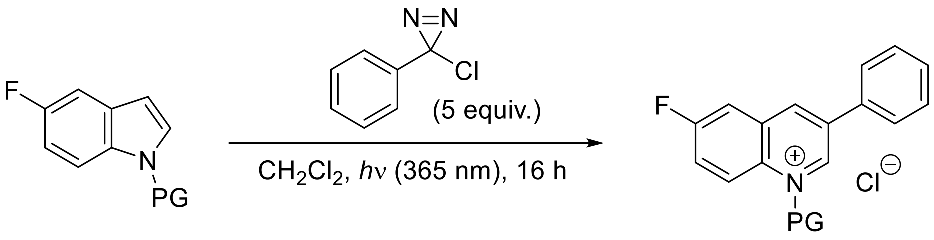

Entry	Protecting Group (PG)	Yield / %
**1**	**Bn**	**82**
2	PMB	51
3	Me	28
4	Allyl	6
5	MOM	5
6	Boc	3[a]
7	TIPS	0
8	SEM	0
9	Piv	0
10	Ts	0

Supplementary Table S2. Protecting group screen of ring expansion reaction. 0.2 mmol scale; yield determined by ^{19}F NMR spectroscopy. [a] Product = neutral 3-phenylquinoline. PMB = *para*-methoxybenzyl, MOM = methoxymethyl, Boc = *tert*-butoxycarbonyl, TIPS = triisopropylsilyl, SEM = trimethylsilylethoxymethyl, Piv = 2,2-dimethylpropanoyl, Ts = *para*-toluenesulfonyl.

Procedure: A 10 mL microwave tube containing *N*-protected indole (0.2 mmol) and 4,4′-bis(trifluoromethyl)-1,1′-biphenyl (internal standard for ^{19}F NMR spectroscopy) was sealed, evacuated, and back-filled with dinitrogen 3 times. CH_2Cl_2 (2 mL) was added, followed by 3-chloro-3-phenyldiazirine (153 mg, 1.0 mmol). The cap of the reaction flask was then sealed with electrical tape, and the reaction mixture was stirred under constant irradiation with UV light (365 nm, 18 W LED, 5 cm from light source) overnight. If any solids precipitated over the course of the reaction, MeOH was added until the mixture was homogenous and an aliquot was taken an analysed by ^{19}F NMR spectroscopy.

5.2. Initial Solvent Screening

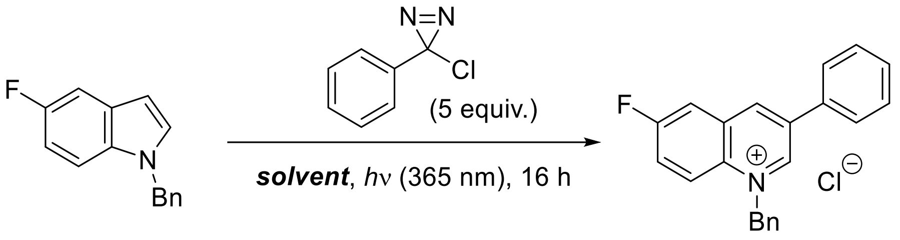

Entry	Solvent	Yield / %
1	CH_2Cl_2	82
2	TBME	49
3	PhMe	37
4	MeCN	20
5	MeOH	0

Supplementary Table S3. Solvent optimisation. 0.2 mmol scale; yields determined by ^{19}F NMR spectroscopy. TBME = *tert*-butyl methyl ether.

Procedure: A 10 mL microwave tube containing 1-benzyl-5-fluoroindole (45 mg, 0.2 mmol) and 4,4′-bis(trifluoromethyl)-1,1′-biphenyl (internal standard for ^{19}F NMR spectroscopy) was sealed, evacuated, and back-filled with dinitrogen 3 times. Anhydrous solvent (2 mL) was added, followed by 3-chloro-3-phenyldiazirine (153 mg, 1.0 mmol). The cap of the reaction flask was then sealed with electrical tape, and the reaction mixture was stirred under constant irradiation with UV light (365 nm, 18 W LED, 5 cm from light source) overnight. If any solids precipitated over the course of the reaction, MeOH was added until the mixture was homogenous and an aliquot was taken an analysed by ^{19}F NMR spectroscopy.

5.3. Further Solvent Screening

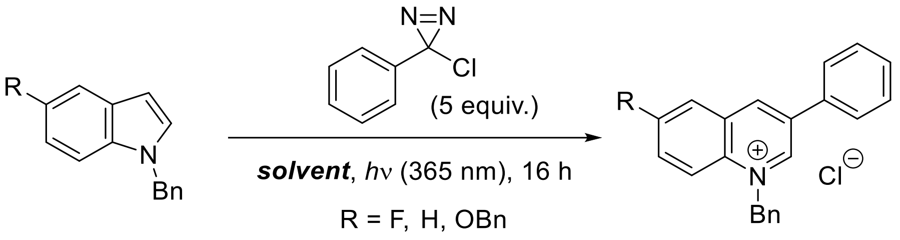

Entry	Solvent	Yield (F) / %	Yield (H) / %	Yield (OBn) / %
1	CH_2Cl_2	82	18	0
2	3:1 CH_2Cl_2/PhMe	84	57	20
3	1:1 CH_2Cl_2/PhMe	54	70	76
4	3:1 CH_2Cl_2/TBME	49	66	44
5	1:1 CH_2Cl_2/TBME	69	70	74

Supplementary Table S4. Mixed solvent system optimisation. 0.2 mmol scale; yields refer to isolated material. TBME = *tert*-butyl methyl ether.

Procedure: A 10 mL microwave tube containing *N*-benzylindole (0.2 mmol) which was then sealed, evacuated and flushed with dinitrogen 3 times. The solvent mixture (2 mL) was then added, followed by 3-chloro-3-phenyldiazirine (153 mg, 1.0 mmol). The cap of the reaction flask was then sealed with electrical tape, and the reaction mixture was stirred under constant irradiation with UV light (365 nm, 18 W LED, 5 cm from light source) overnight. The resulting precipitate was isolated by filtration and washed with PhMe (2 × 5 mL) and dried under a flow of air.

5.4. Comparison to Literature

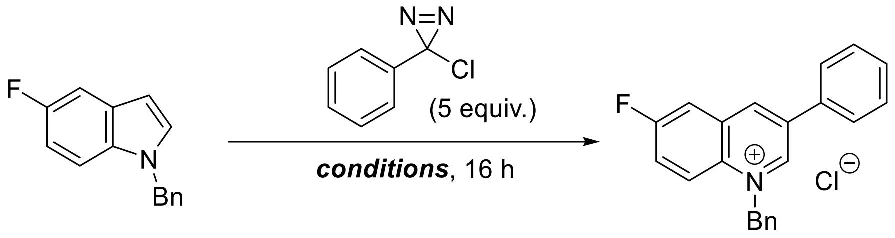

Entry	Conditions	Product / %	Starting material / %
1	CH_2Cl_2, *h*ν, rt	82	2
2	CH_2Cl_2, dark, 50 °C	64	19
3	MeCN, *h*ν, rt	20	35
4	MeCN, dark, 50 °C	16	34
5	MeCN, 3.0 equiv. Na_2CO_3, dark, 50 °C	0	30
6	1 mol% $Rh_2(OAc)_4$, CH_2Cl_2, dark, rt	0	>95

Supplementary Table S5. Comparison to previous literature conditions.[28,31] 0.2 mmol scale; yields determined by ^{19}F NMR spectroscopy.

Procedure: A 10 mL microwave tube containing 1-benzyl-5-fluoroindole (45 mg, 0.2 mmol), Na_2CO_3 (for **entry 5** only; 64 mg, 0.6 mmol), $Rh_2(OAc)_4$ (for **entry 6** only; 0.88 mg, 0.002 mmol), and 4,4′-bis(trifluoromethyl)-1,1′-biphenyl (internal standard for ^{19}F NMR spectroscopy) was sealed, evacuated, and back-filled with dinitrogen 3 times. Anhydrous solvent (2 mL) was added, followed by 3-chloro-3-phenyldiazirine (153 mg, 1.0 mmol). The cap of the reaction flask was then sealed with electrical tape. The reaction mixture was stirred overnight with either constant UV irradiation (365 nm, 18 W LED, 5 cm from light source; **entries 1 and 3**), or in the dark (**entries 2, 4 and 5**: 50 °C; **entry 6**: rt). If any solids precipitated over the course of the reaction, MeOH was added until the mixture was homogenous and an aliquot was taken an analysed by ^{19}F NMR spectroscopy.

5.5. Reaction Robustness

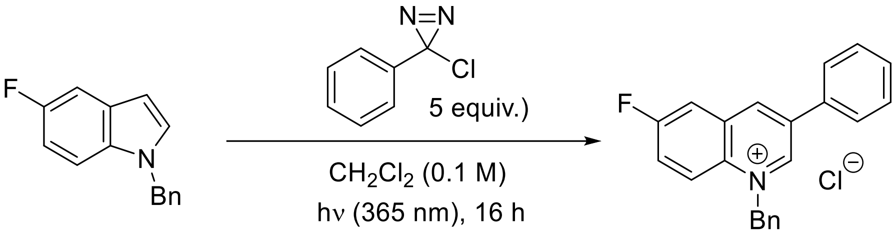

Entry	Deviation from above	Yield / %
1	None	82
2	Winchester-grade CH_2Cl_2	42
3	Degassed, Winchester-grade CH_2Cl_2	55
4	Initial concentration was 0.2 M	37
5	Initial concentration was 0.05 M	80
6	Scratched MW tube	81
7	6 h reaction time	65

Supplementary Table S6. Deviations from standard reaction conditions. 'Scratched' refers to a used microwave tube that was visibly etched / scratched as a test for light penetration. 0.2 mmol scale; yields determined by ^{19}F NMR spectroscopy.

Procedure: As Supplementary Table S3, with the variations noted in the individual entries.

6. Reaction Limitations

6.1. Incompatible Substrates

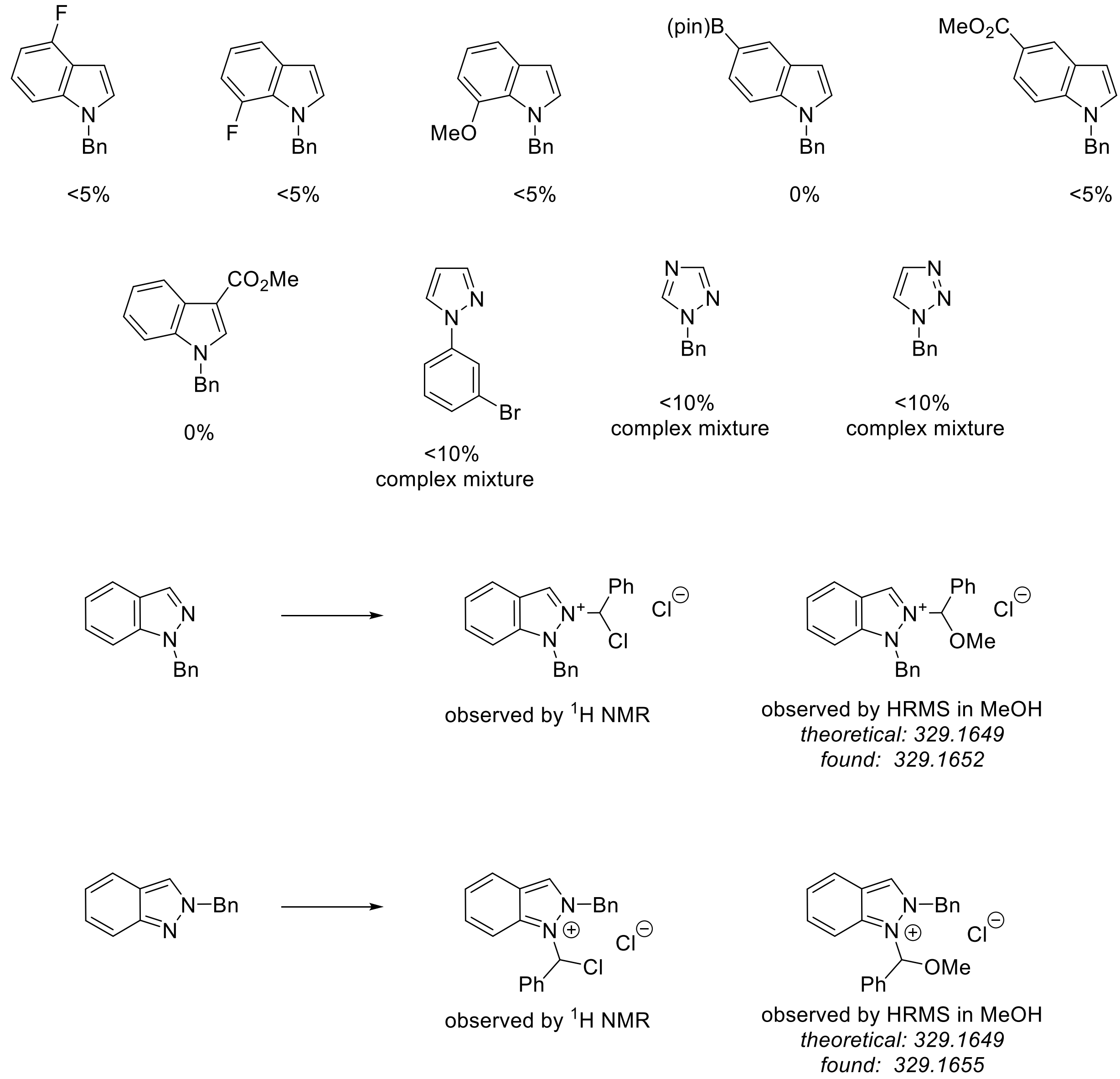

Supplementary Figure S1: Additional substrates leading to no or trace formation of ring-expanded product.

Discussion: The low yields obtained for reactions of 4- and 7-substituted indoles (**14-17**, manuscript Scheme 2; Supplementary Figure S1) cannot be reliably explained by the steric or electronic properties of the substituent alone, but are consistent with prior literature observations.[23] For example, F is smaller

in size than Me (wB_1(F) = 1.52; wB_1(Me) = 1.88),[32] yet 4- and 7-fluoroindoles are outperformed by the analogous 4- and 7-methylindoles. This suggests that steric factors are not solely responsible for the poor performance of 4- and 7-fluoroindoles, which is further substantiated by the good reactivity observed for 2- and 3-substituted indoles (**18**, **23**, manuscript Scheme 2). Electronically very different (Br, F, Me, MeO) substituents are not well tolerated at the 4- or 7-positions, but are compatible at the 5- and 6-positions, suggesting that electronic factors are also not solely responsible for the observed reactivity of 4- and 7-substituted indoles. Thus, while the individual contributions of sterics and electronics to the performance of 4- and 7-substituted indoles is not currently clear, the combined effect is clearly detrimental for our methodology.

Characterisation Data for *N*²-Chloroalkylated *N*¹-Benzylindazole (48)

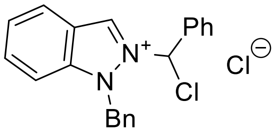

¹H NMR (400 MHz, CD$_3$OD): δ 7.82 – 7.74 (m, 3H), 7.72 – 7.61 (m, 3H), 7.61 – 7.49 (m, 3H), 7.34 – 7.22 (m, 3H), 7.21 – 7.13 (m, 2H), 6.11 (s, 1H), 5.50 (d, *J* = 16.9 Hz, 1H), 5.36 (d, *J* = 16.9 Hz, 1H).

HRMS: calcd. for $C_{12}H_{15}N_2$ [M-Cl$_2$+OCH$_3$]$^+$ [ii]: 329.1649; found (ESI$^+$): 329.1652.

[ii] HRMS analysis carried out in MeOH solvent. Observed adduct is consistent with displacement of Cl by MeOH.

6.2. Poisoning Studies

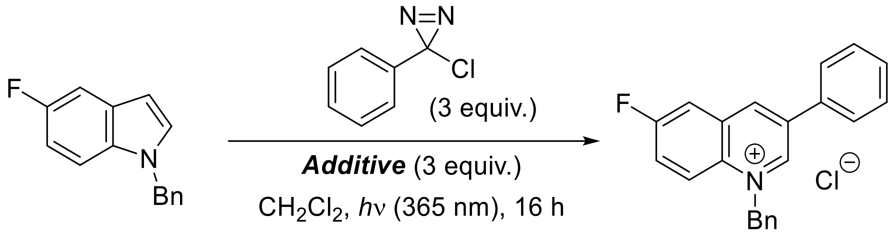

Entry	Additive	Yield / %
1	[^{n}Bu$_4$N][Cl]	0
2	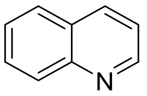	7
3	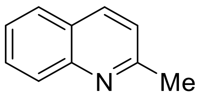	21

Supplementary Table S7. Effects of additives on the ring expansion reaction. 0.2 mmol scale; yields determined by ^{19}F NMR spectroscopy.

Procedure: A 10 mL microwave tube containing 1-benzyl-5-fluoroindole (45 mg, 0.2 mmol), the additive (if solid; 0.6 mmol) and 4,4′-bis(trifluoromethyl)-1,1′-biphenyl (internal standard for ^{19}F NMR spectroscopy) was sealed, evacuated, and back-filled with dinitrogen 3 times. CH_2Cl_2 (2 mL) was added, followed by 3-chloro-3-phenyldiazirine (92 mg, 0.6 mmol). An aliquot was taken for ^{19}F NMR analysis, then the cap of the reaction flask was then sealed with electrical tape, and the reaction mixture was stirred under constant irradiation with UV light (365 nm, 18 W LED, 5 cm from light source) overnight. If solids precipitated, the reaction was diluted with MeOH until homogenous and an aliquot was taken an analysed by ^{19}F NMR spectroscopy.

7. DSC Analysis

DSC Method:

Approximately 5 mg of material was weighed into a high pressure stainless-steel crucible (TA Instruments; #900808.901) using a 7-place balance. The crucible was fitted with a disposable gold-coated copper seal (TA Instruments; 900814.901), then sealed under air. After equilibrating the sample at 25 °C, the sample was heated at 5 °C/min. Initial measurements were made to 250 °C; repeat measurements were made to 200 °C or 180 °C once it was clear that the exotherms concluded <160 °C.

Temperatures denoted by the arrow are T_{init} and correspond to temperatures 0.01 W g^{-1} above the baseline value (not necessarily 0 W g^{-1}). Exothermic events are positive in the *y*-axis.

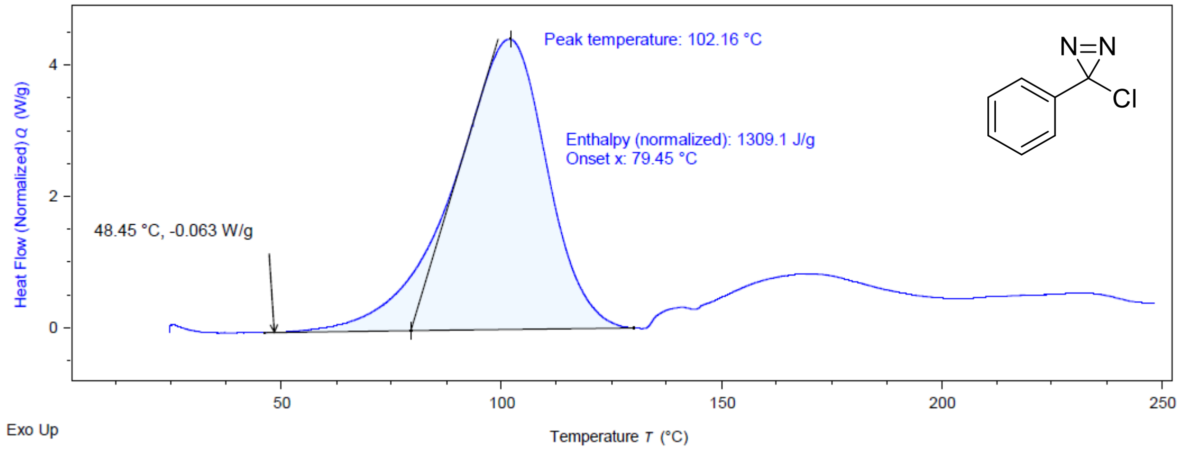

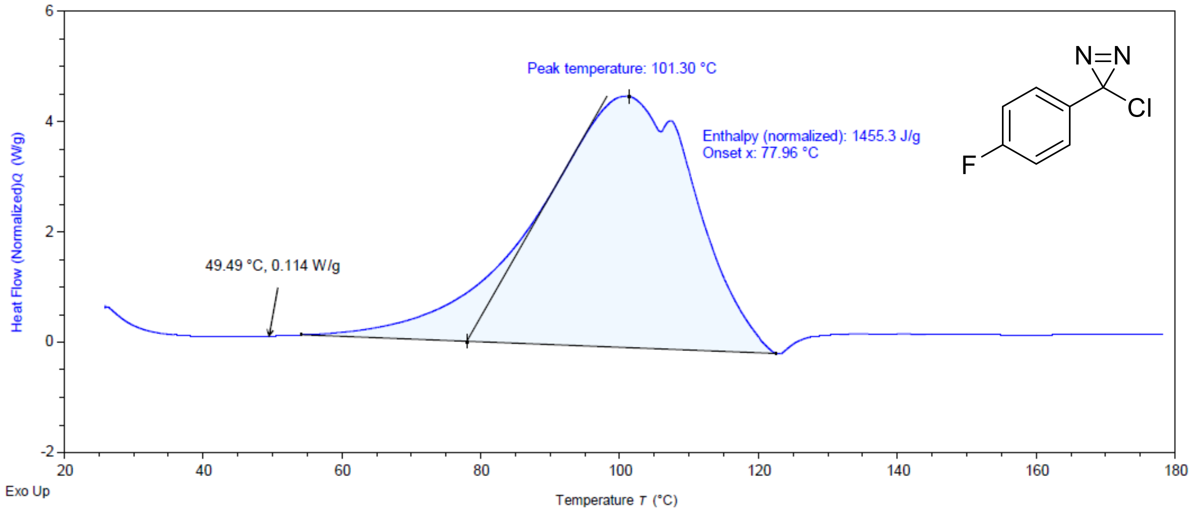

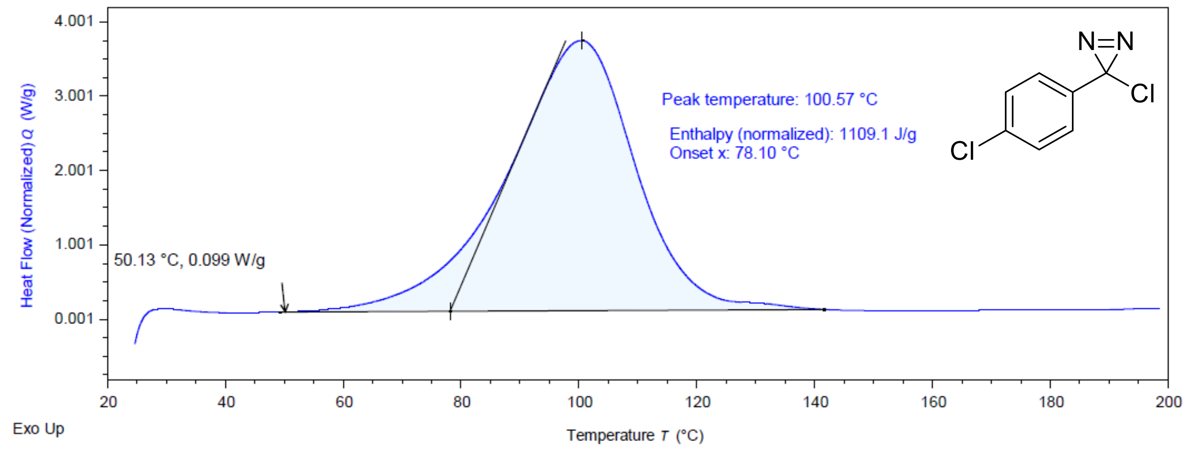

Heat Flow (Normalized) Q (W/g)
4.001
3.001
2.001
1.001
0.001
50.13 °C, 0.099 W/g
Peak temperature: 100.57 °C
Enthalpy (normalized): 1109.1 J/g
Onset x: 78.10 °C
N=N
Cl
Cl
20
40
60
80
100
120
140
160
180
200
Exo Up
Temperature T (°C)

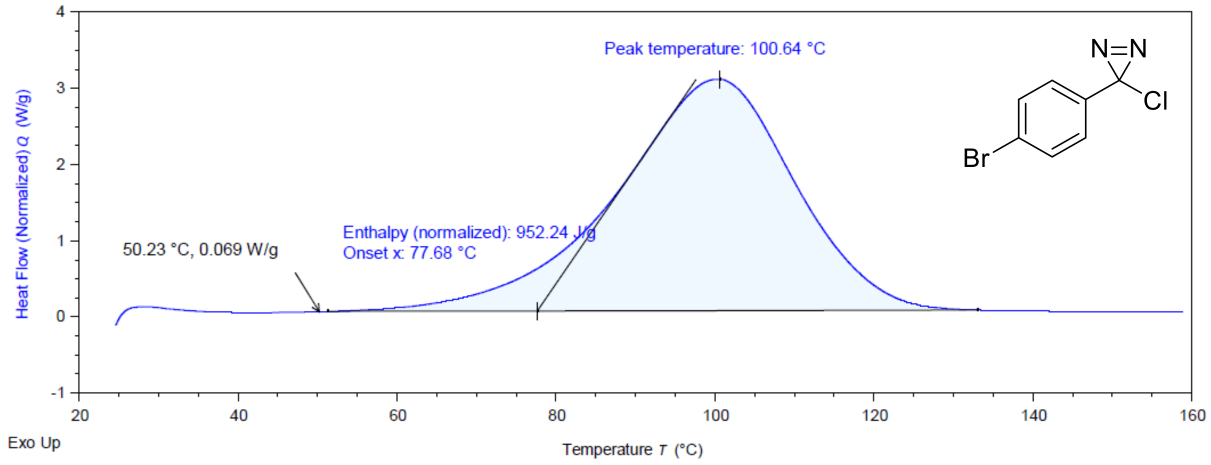

Heat Flow (Normalized) Q (W/g)
4
3
2
1
0
-1
Peak temperature: 100.64 °C
50.23 °C, 0.069 W/g
Enthalpy (normalized): 952.24 J/g
Onset x: 77.68 °C
N=N
Cl
Br
20
40
60
80
100
120
140
160
Exo Up
Temperature T (°C)

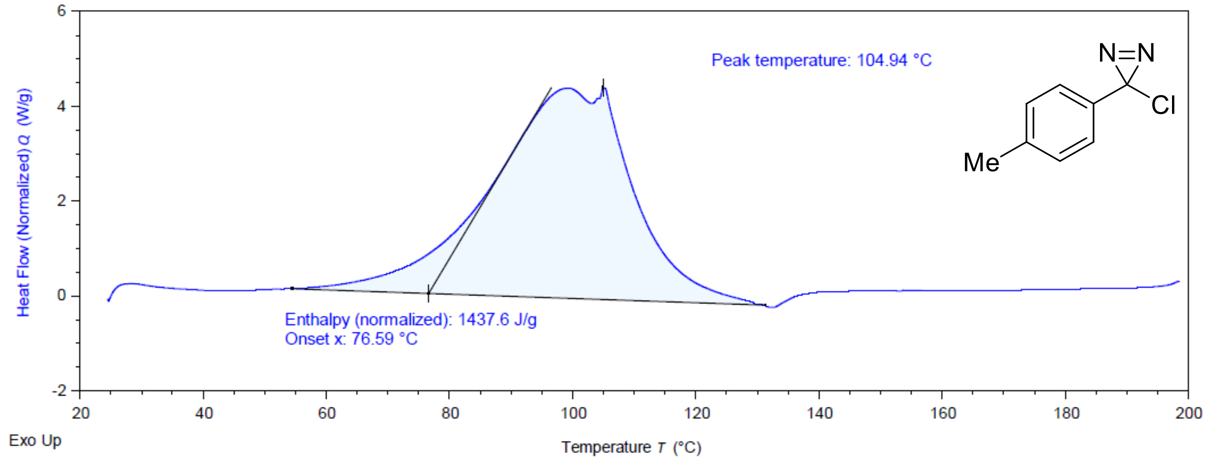

Heat Flow (Normalized) Q (W/g)
6
4
2
0
-2
Peak temperature: 104.94 °C
Enthalpy (normalized): 1437.6 J/g
Onset x: 76.59 °C
N=N
Cl
Me
20
40
60
80
100
120
140
160
180
200
Exo Up
Temperature T (°C)

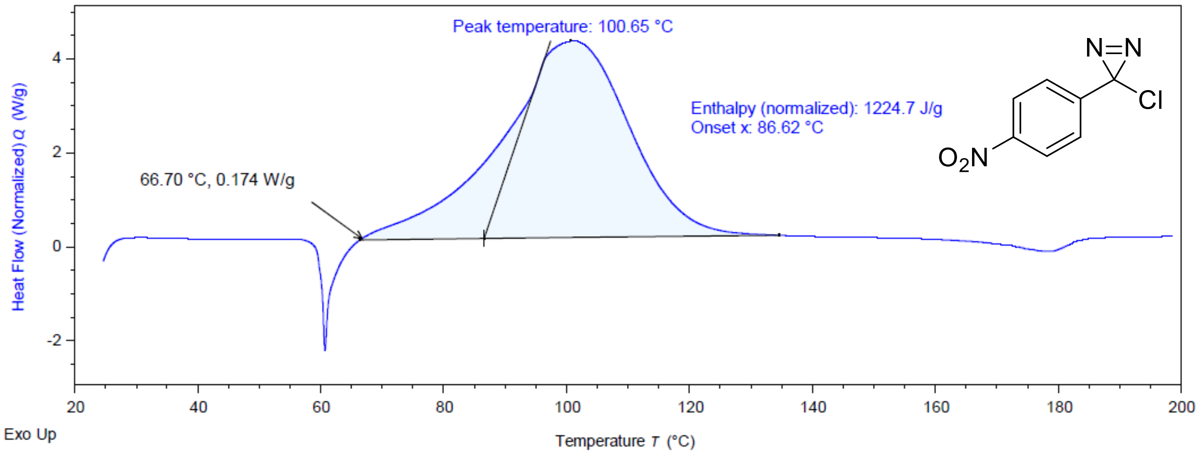

Peak temperature: 100.65 °C
Enthalpy (normalized): 1224.7 J/g
Onset x: 86.62 °C
66.70 °C, 0.174 W/g
Heat Flow (Normalized) Q (W/g)
Temperature T (°C)
Exo Up
N=N
Cl
O2N

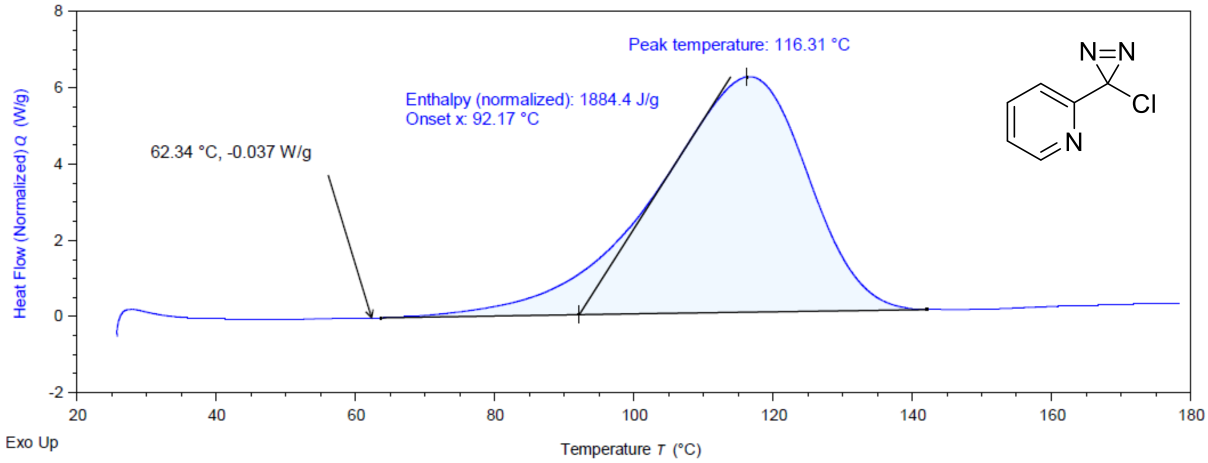

Peak temperature: 116.31 °C
Enthalpy (normalized): 1884.4 J/g
Onset x: 92.17 °C
62.34 °C, -0.037 W/g
Heat Flow (Normalized) Q (W/g)
Temperature T (°C)
Exo Up
N=N
Cl
N

8. References

[1] Y.-Y. Zhang, J.-D. Lin, X.-L. Xu, J.-H. Li, *Synth. Comm.* **2010**, *40*, 2556–2563.

[2] J. C. Borghs, V. Zubar, L. M. Azofra, J. Sklyaruk, M. Rueping, *Org. Lett.* **2020**, *22*, 4222–4227.

[3] S. Islam, I. Larrosa, *Chem. Eur. J.* **2013**, *19*, 15093–15096.

[4] M. Shee, S. S. Shah, N. D. P. Singh, *Chem. Commun.* **2020**, *56*, 4240–4243.

[5] H. Chen, H. Yang, N. Li, X. Xue, Z. He, Q. Zeng, *Org. Process Res. Dev.* **2019**, *23*, 1679–1685.

[6] M. J. Aalam, Deepa, S. Singh, *Eur. J. Org. Chem.* **2022**, *2022*, e202200978.

[7] V. Yadav, S. G. Jagtap, E. Balaraman, S. B. Mhaske, *Org. Lett.* **2022**, *24*, 9054–9059.

[8] R. Lang, L. Shi, D. Li, C. Xia, F. Li, *Org. Lett.* **2012**, *14*, 4130–4133.

[9] A. F. G. Maier, S. Tussing, T. Schneider, U. Flörke, Z.-W. Qu, S. Grimme, J. Paradies, *Angew. Chem. Int. Ed.* **2016**, *55*, 12219–12223.

[10] Y. A. Ho, M. Leiendecker, X. Liu, C. Wang, N. Alandini, M. Rueping, *Org. Lett.* **2018**, *20*, 5644–5647.

[11] S. H. Son, J.-W. Shin, H.-J. Won, H.-S. Yoo, Y. Y. Cho, S. L. Kim, Y. H. Jang, B. Y. Park, N.-J. Kim, *Org. Lett.* **2021**, *23*, 7467–7471.

[12] P. Kannaboina, K. A. Kumar, P. Das, *Org. Lett.* **2016**, *18*, 900–903.

[13] M. Righi, F. Topi, S. Bartolucci, A. Bedini, G. Piersanti, G. Spadoni, *J. Org. Chem.* **2012**, *77*, 6351–6357.

[14] L. Junk, E. Papadopoulos, U. Kazmaier, *Synthesis* **2021**, *53*, 2503–2511.

[15] B. Emayavaramban, M. Sen, B. Sundararaju, *Org. Lett.* **2017**, *19*, 6–9.

[16] R. Honeker, J. B. Ernst, F. Glorius, *Chem. Eur. J.* **2015**, *21*, 8047–8051.

[17] W. Fu, L. Zhu, S. Tan, Z. Zhao, X. Yu, L. Wang, *J. Org. Chem.* **2022**, *87*, 13389–13395.

[18] S. Liu, C. C. Tzschucke, *Eur. J. Org. Chem.* **2016**, *2016*, 3509–3513.

[19] Y. Li, J. Han, H. Luo, Q. An, X.-P. Cao, B. Li, *Org. Lett.* **2019**, *21*, 6050–6053.

[20] S. Sengmany, S. Vasseur, A. Lajnef, E. Le Gall, E. Léonel, *Eur. J. Org. Chem.* **2016**, *2016*, 4865–4871.

[21] N. D. Smith, D. Huang, N. D. P. Cosford, *Org. Lett.* **2002**, *4*, 3537–3539.

[22] X. Han, J. Wu, *Angew. Chem. Int. Ed.* **2013**, *52*, 4637–4640.

[23] P. Wang, M. E. Farmer, X. Huo, P. Jain, P.-X. Shen, M. Ishoey, J. E. Bradner, S. R. Wisniewski, M. D. Eastgate, J.-Q. Yu, *J. Am. Chem. Soc.* **2016**, *138*, 9269–9276.

[24] G. Wu, Z. Yang, X. Xu, L. Hao, L. Chen, Y. Wang, Y. Ji, *Org. Lett.* **2022**, *24*, 3570–3575.

[25] Y. Schneider, J. Prévost, M. Gobin, C. Y. Legault, *Org. Lett.* **2014**, *16*, 596–599.

[26] R. Katla, R. Chowrasia, P. S. Manjari, C. D. G. da Silva, B. F. dos Santos, N. L. C. Domingues, *New J. Chem.* **2016**, *40*, 9471–9476.

[27] M. G. Rosenberg, U. H. Brinker, *J. Org. Chem.* **2003**, *68*, 4819–4832.

[28] B. D. Dherange, P. Q. Kelly, J. P. Liles, M. S. Sigman, M. D. Levin, *J. Am. Chem. Soc.* **2021**, *143*, 11337–11344.

[29] Y. Jin, L. Ou, H. Yang, H. Fu, *J. Am. Chem. Soc.* **2017**, *139*, 14237–14243.

[30] M. Hönel, F. W. Vierhapper, *J. Chem. Soc., Perkin Trans. 1* **1982**, 2607–2610.

[31] M. Mortén, M. Hennum, T. Bonge-Hansen, *Beilstein J. Org. Chem.* **2015**, *11*, 1944–1949.

[32] A. V. Brethomé, S. P. Fletcher, R. S. Paton, *ACS Catal.* **2019**, *9*, 2313–2323.

9. NMR Spectra

3: 1-Benzyl-3-phenyl-6-fluoroquinolinium chloride – ^{1}H NMR (400 MHz, CD_3OD)

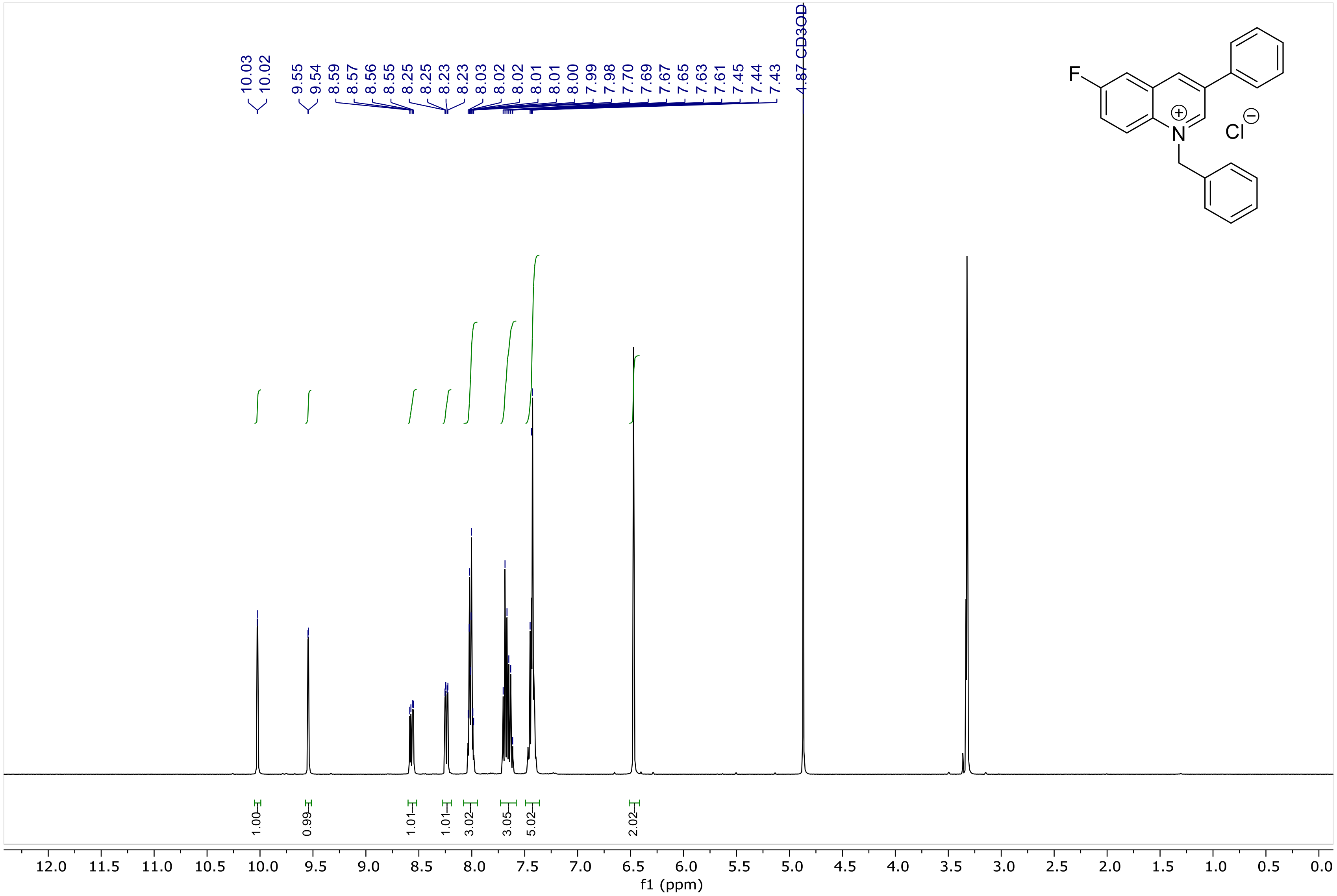

3: 1-Benzyl-3-phenyl-6-fluoroquinolinium chloride – $^{13}C\{^1H\}$ NMR (101 MHz, CD_3OD)

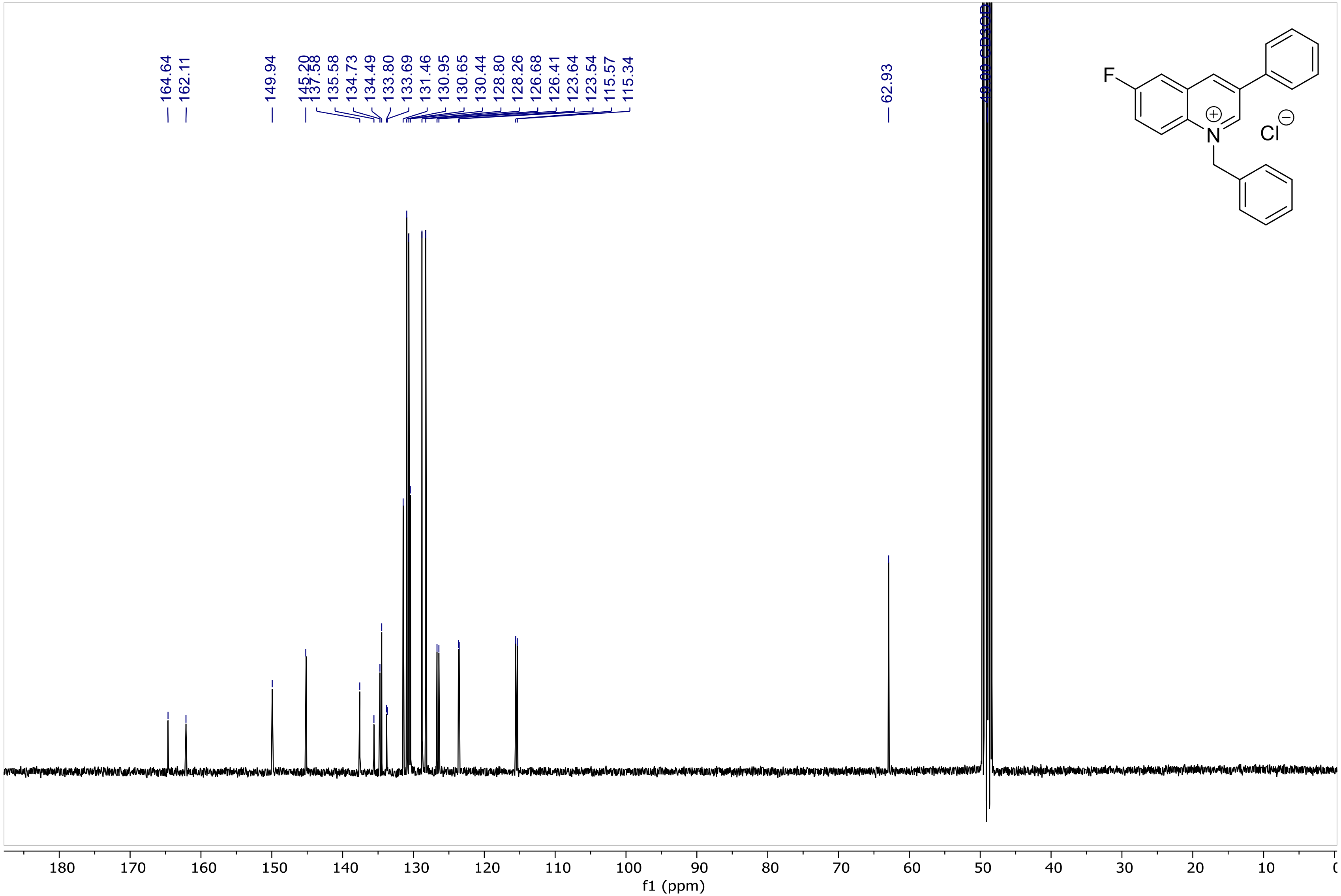

3: 1-Benzyl-3-phenyl-6-fluoroquinolinium chloride – ^{19}F NMR (376 MHz, CD_3OD)

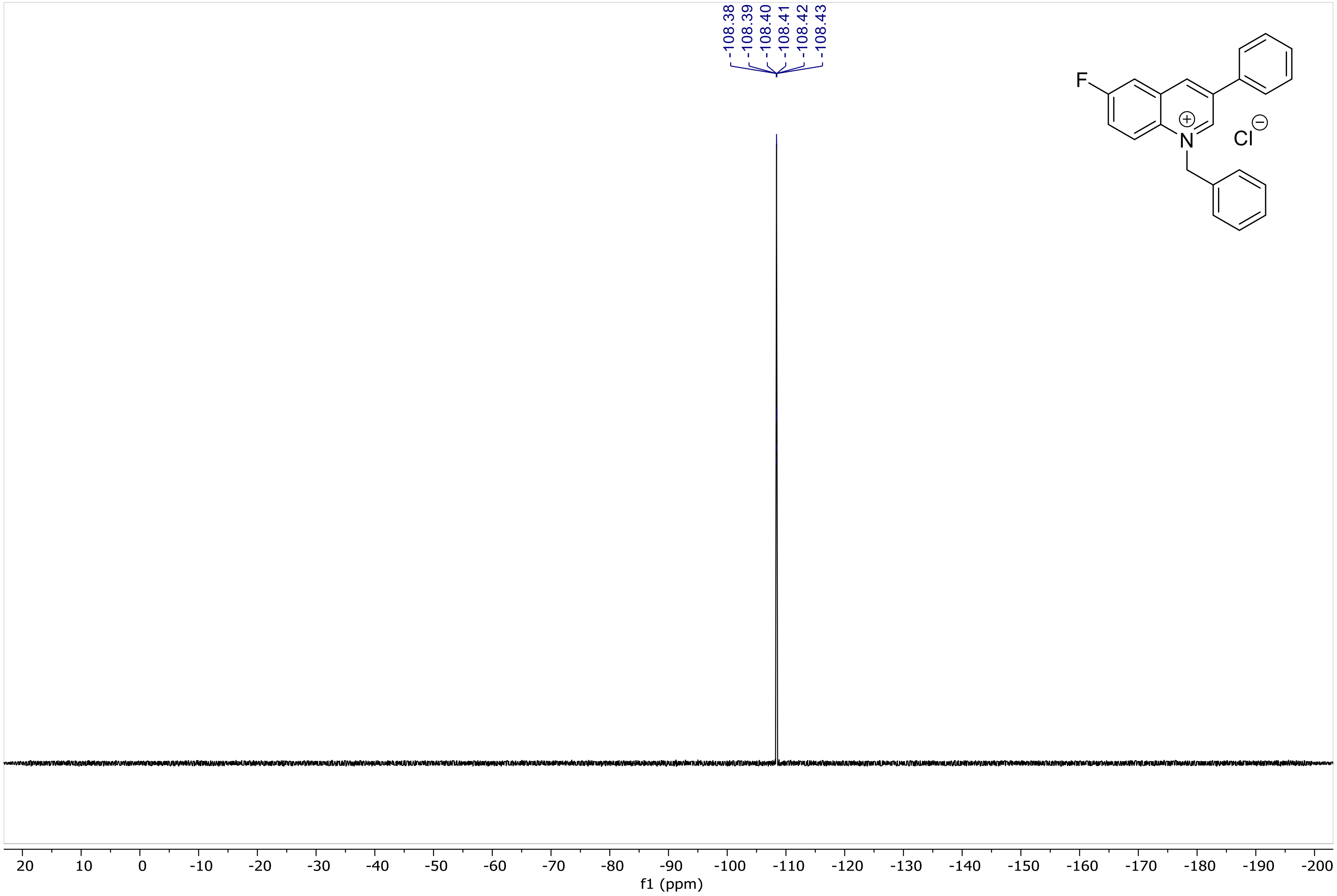

4: 1-Benzyl-3-phenyl-6-chloroquinolinium chloride – ^{1}H NMR (400 MHz, CD_3OD)

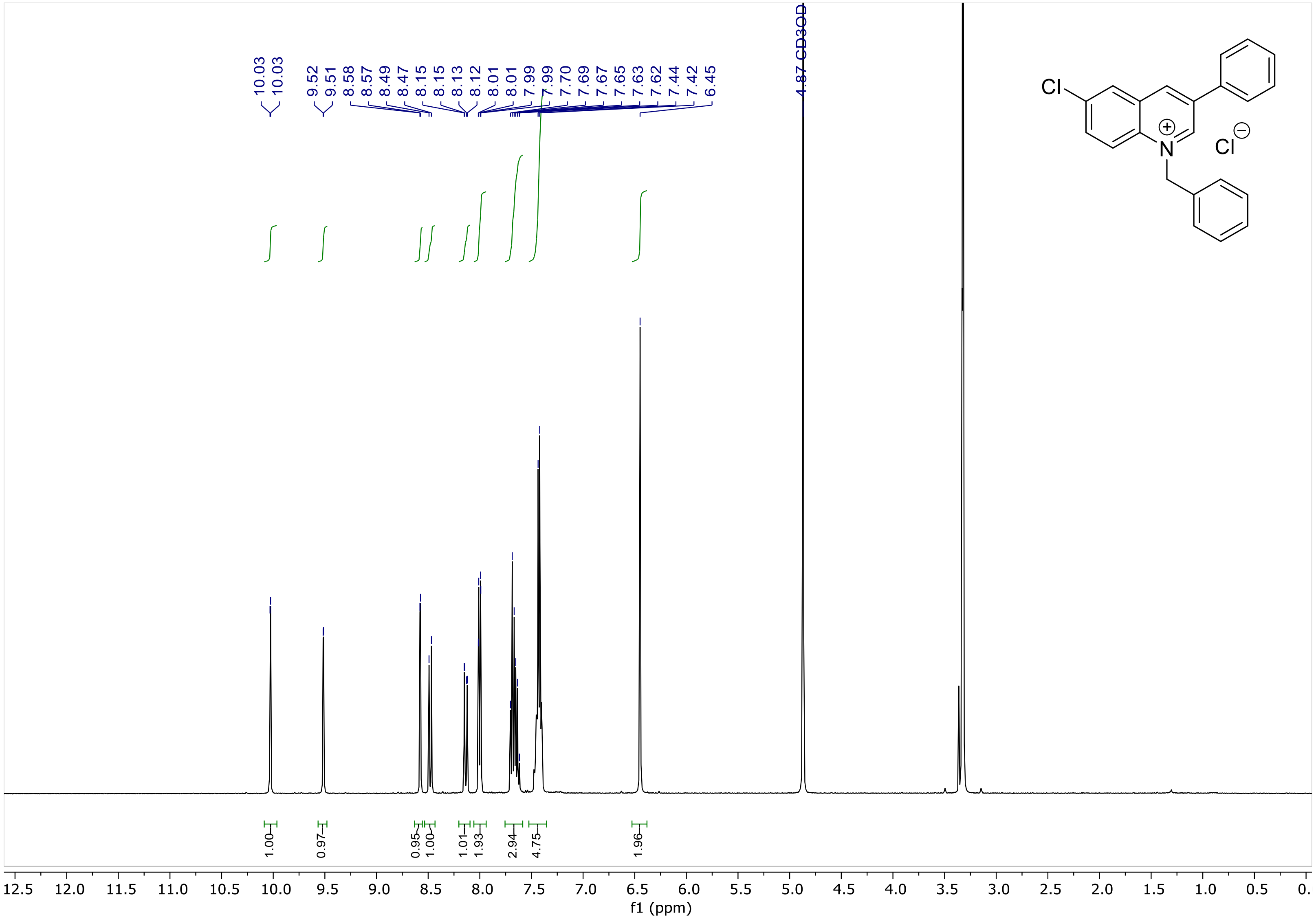

4: 1-Benzyl-3-phenyl-6-chloroquinolinium chloride – $^{13}C\{^1H\}$ NMR (101 MHz, CD_3OD)

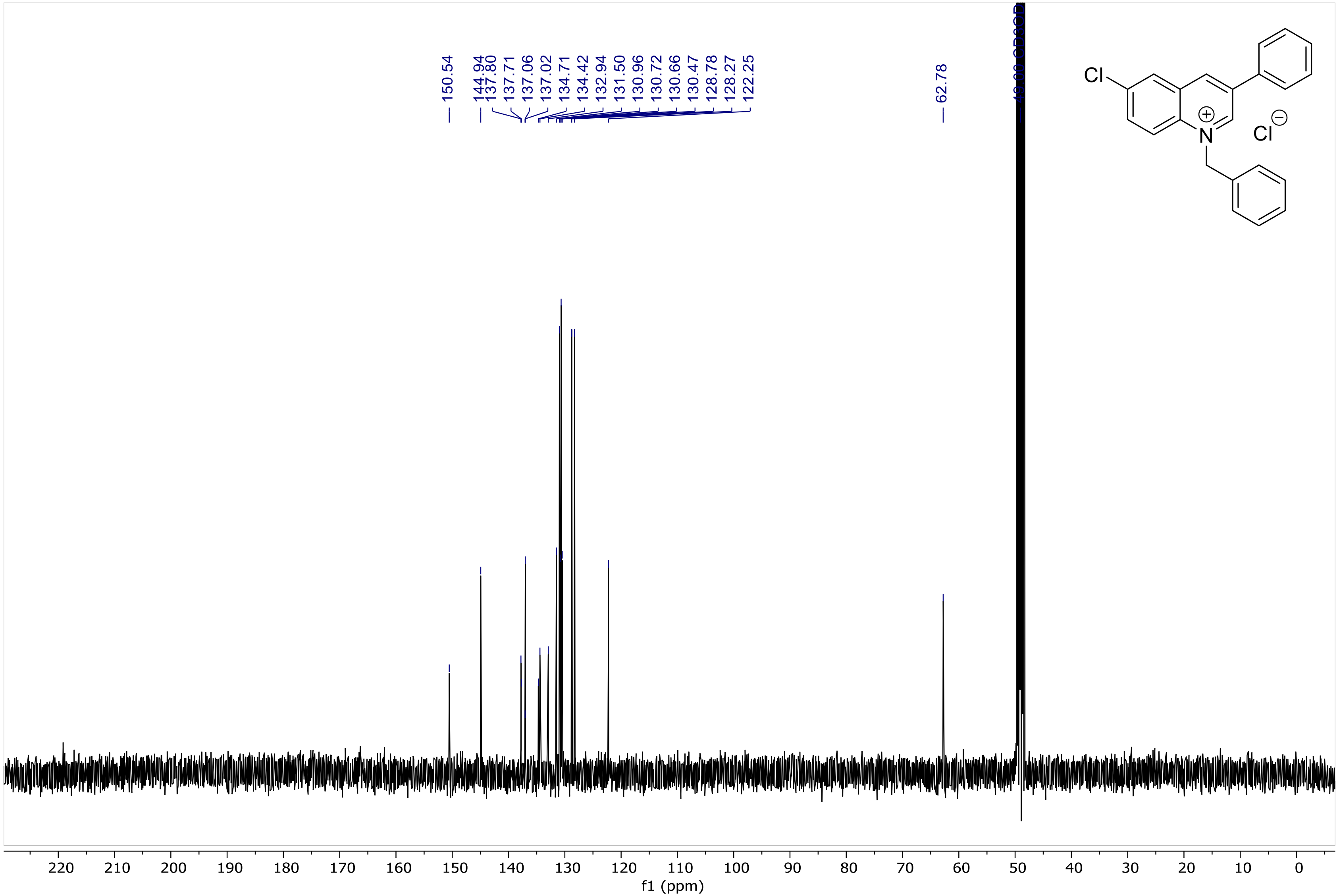

5: 1-Benzyl-3-phenyl-6-bromoquinolinium chloride – ^{1}H NMR (400 MHz, CD_3OD)

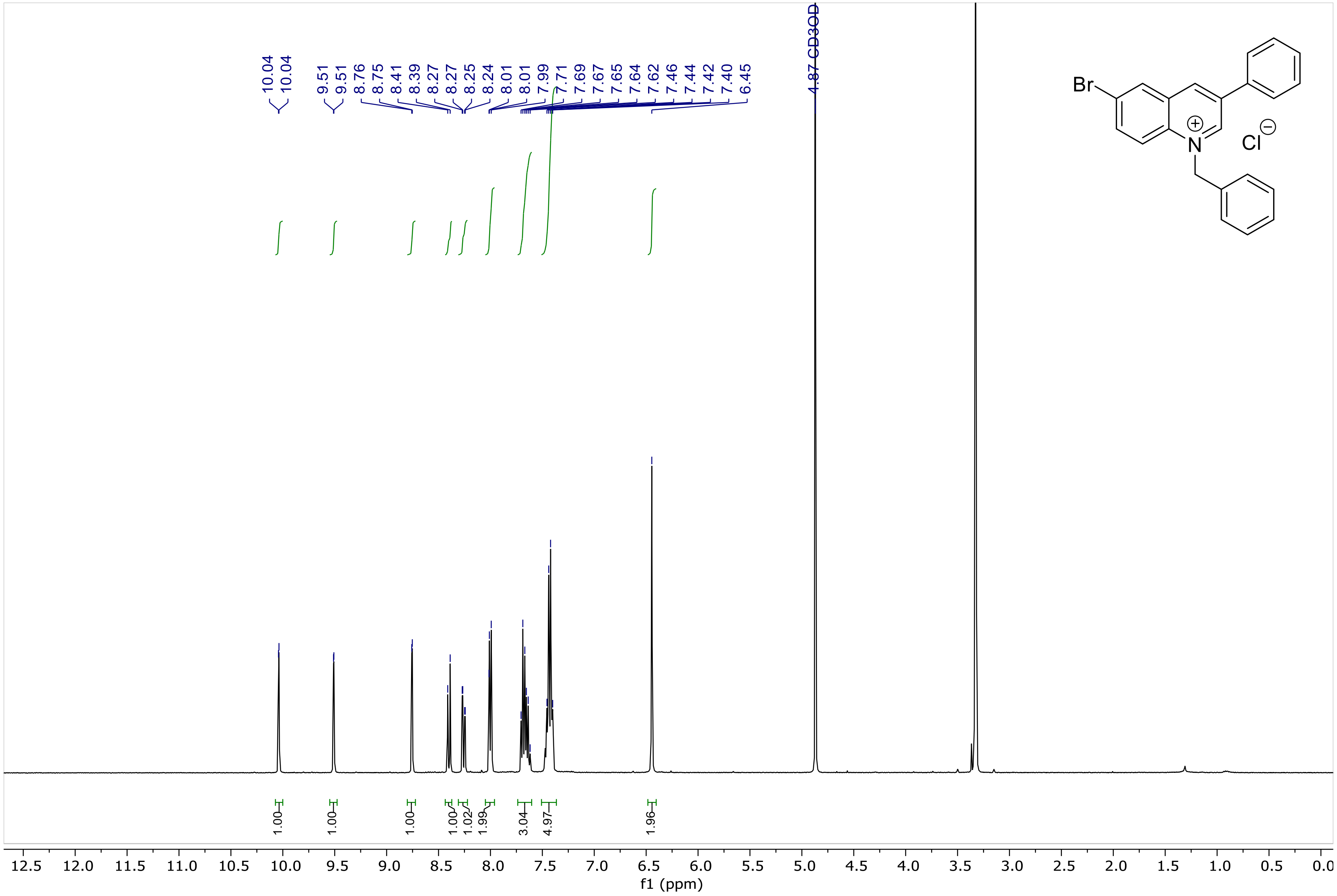

5: 1-Benzyl-3-phenyl-6-bromoquinolinium chloride – $^{13}C\{^1H\}$ NMR (101 MHz, CD_3OD)

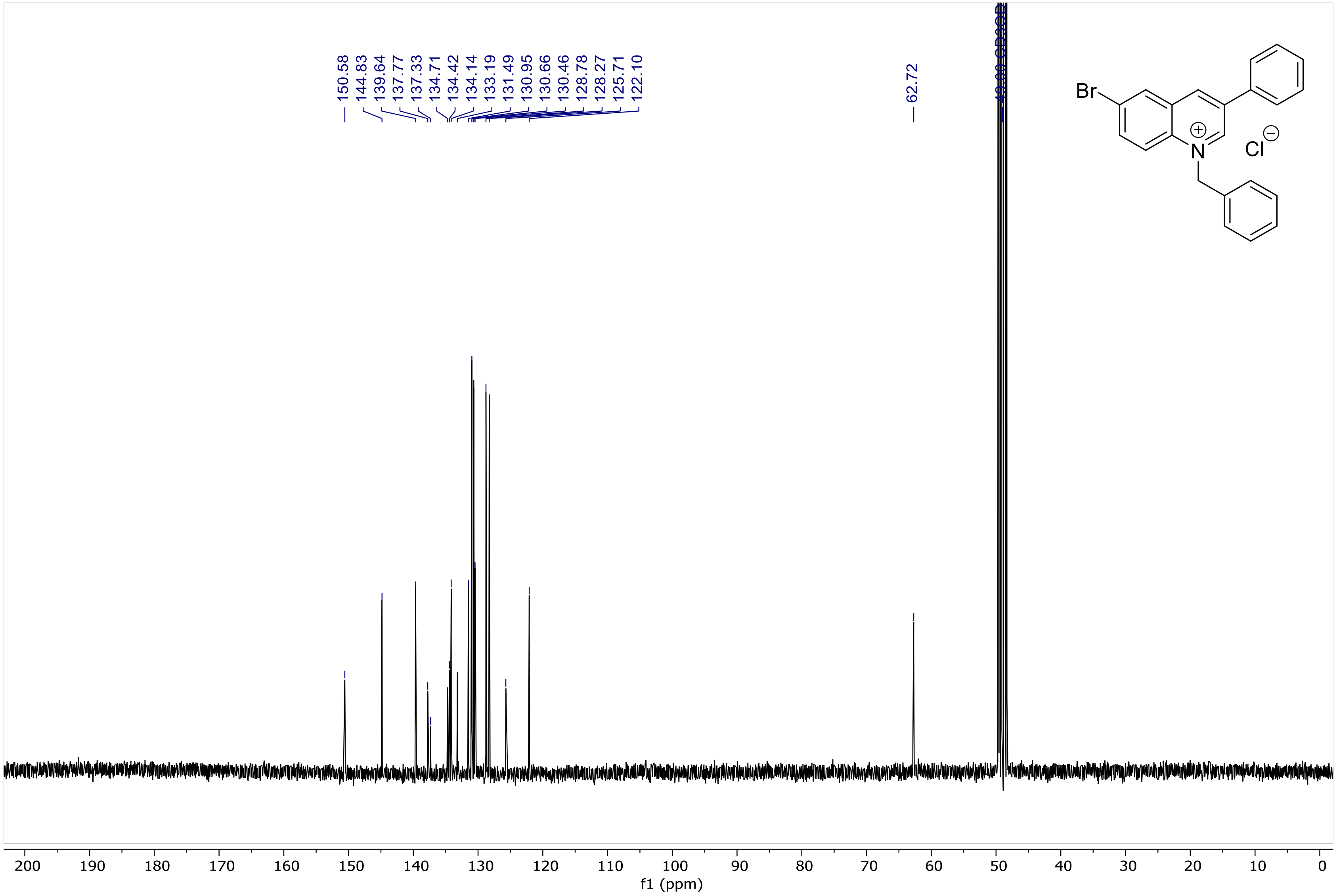

6: 1-Benzyl-3-phenyl-6-cyanoquinolinium chloride – ^{1}H NMR (400 MHz, CD_3OD)

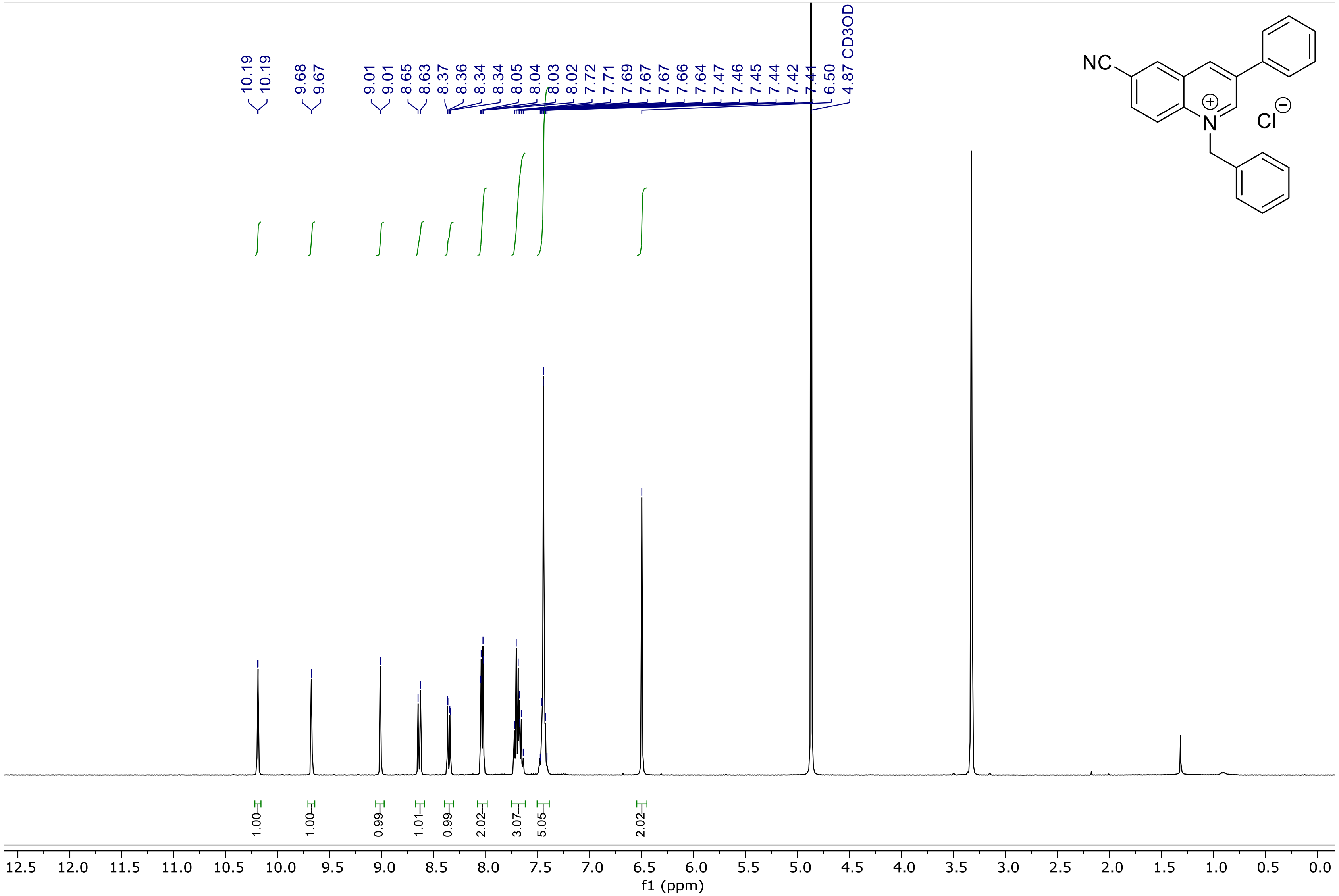

6: 1-Benzyl-3-phenyl-6-cyanoquinolinium chloride – $^{13}C\{^1H\}$ NMR (101 MHz, CD_3OD)

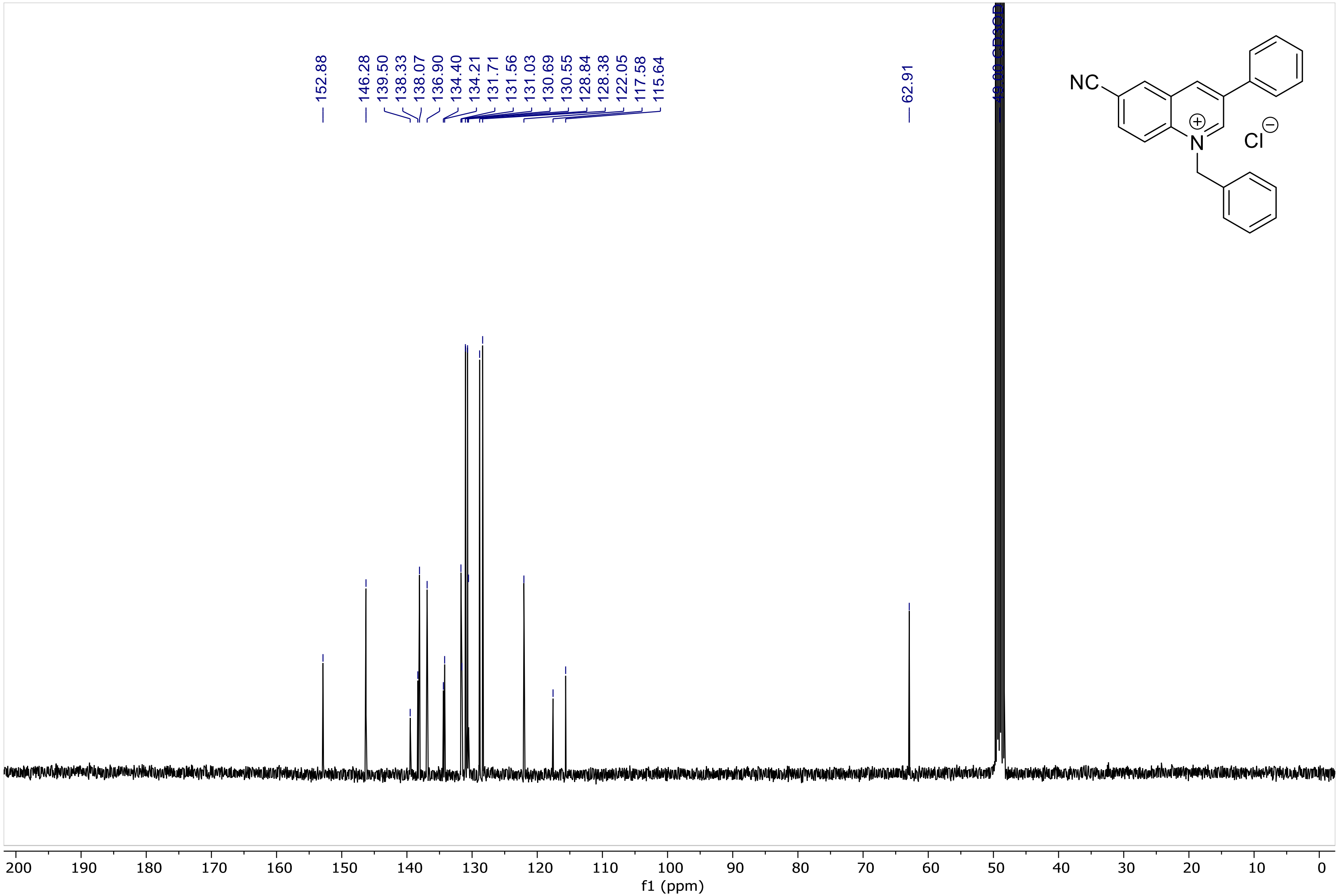

7: 1-Benzyl-3-phenyl-6-nitroquinolinium chloride – ^{1}H NMR (400 MHz, CD_3OD)

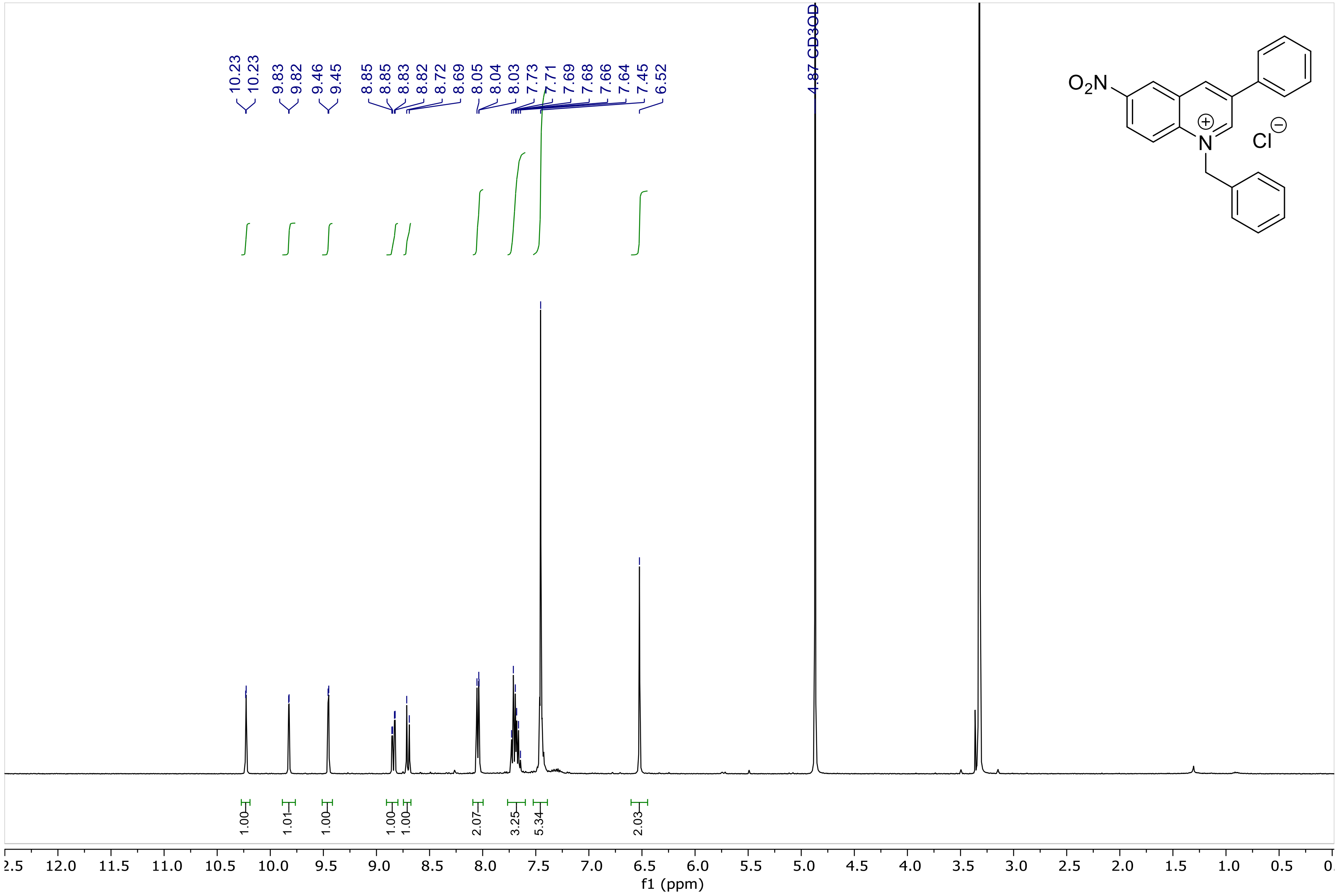

7: 1-Benzyl-3-phenyl-6-nitroquinolinium chloride – $^{13}C\{^1H\}$ NMR (101 MHz, CD_3OD)

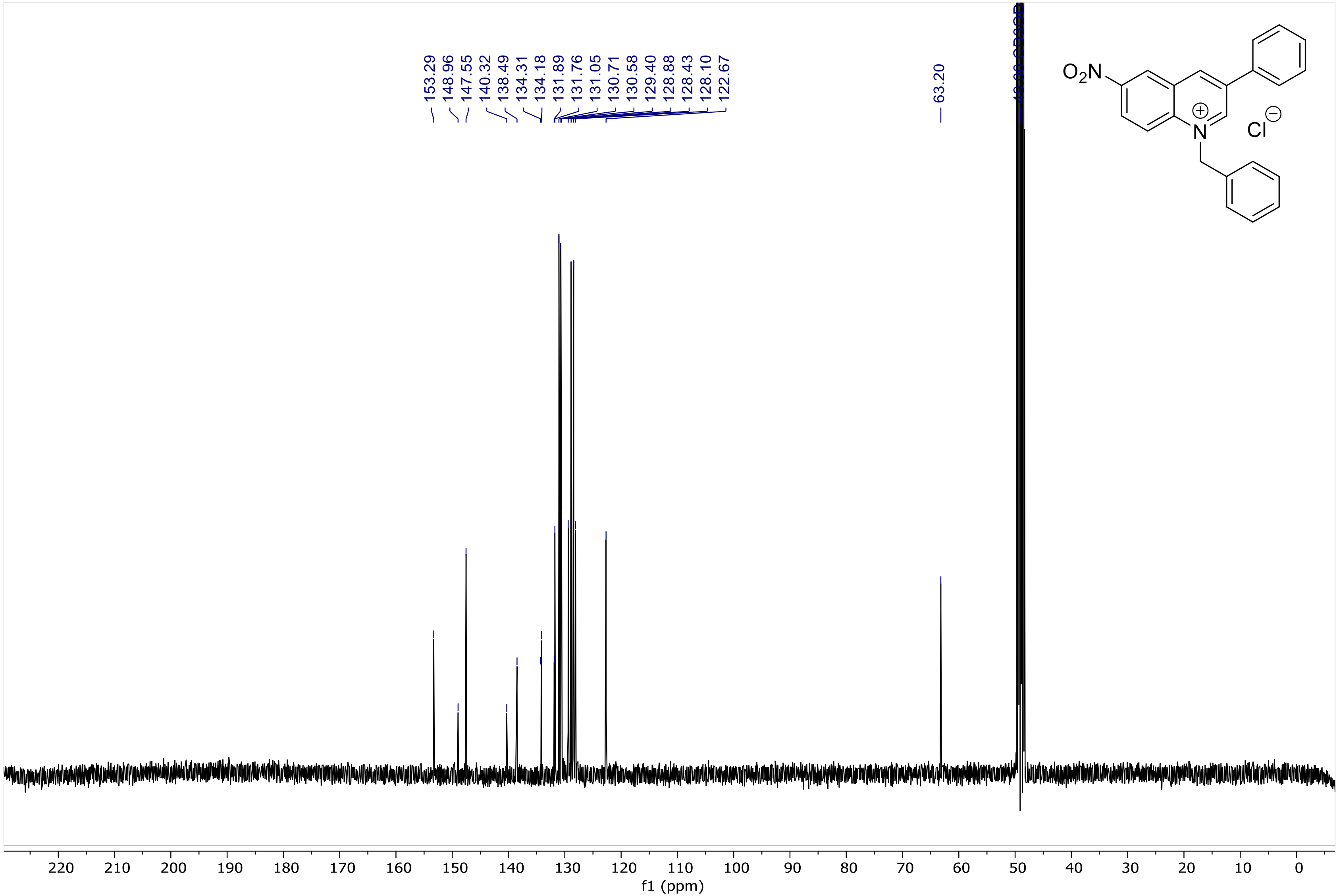

8: 1-Benzyl-3-phenylquinolinium chloride – ¹H NMR (400 MHz, CD_3OD)

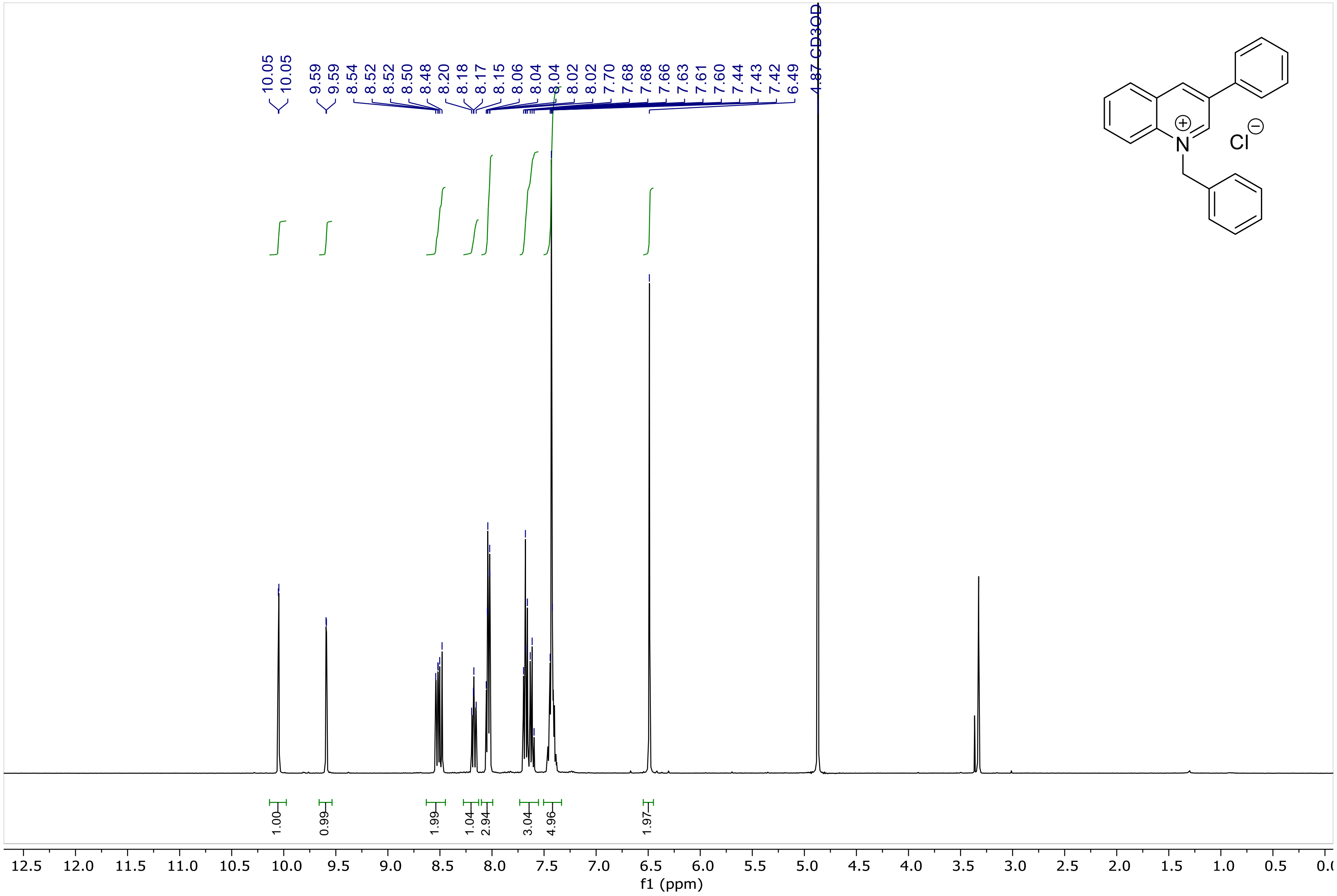

8: 1-Benzyl-3-phenylquinolinium chloride – $^{13}C\{^{1}H\}$ NMR (101 MHz, CD_3OD)

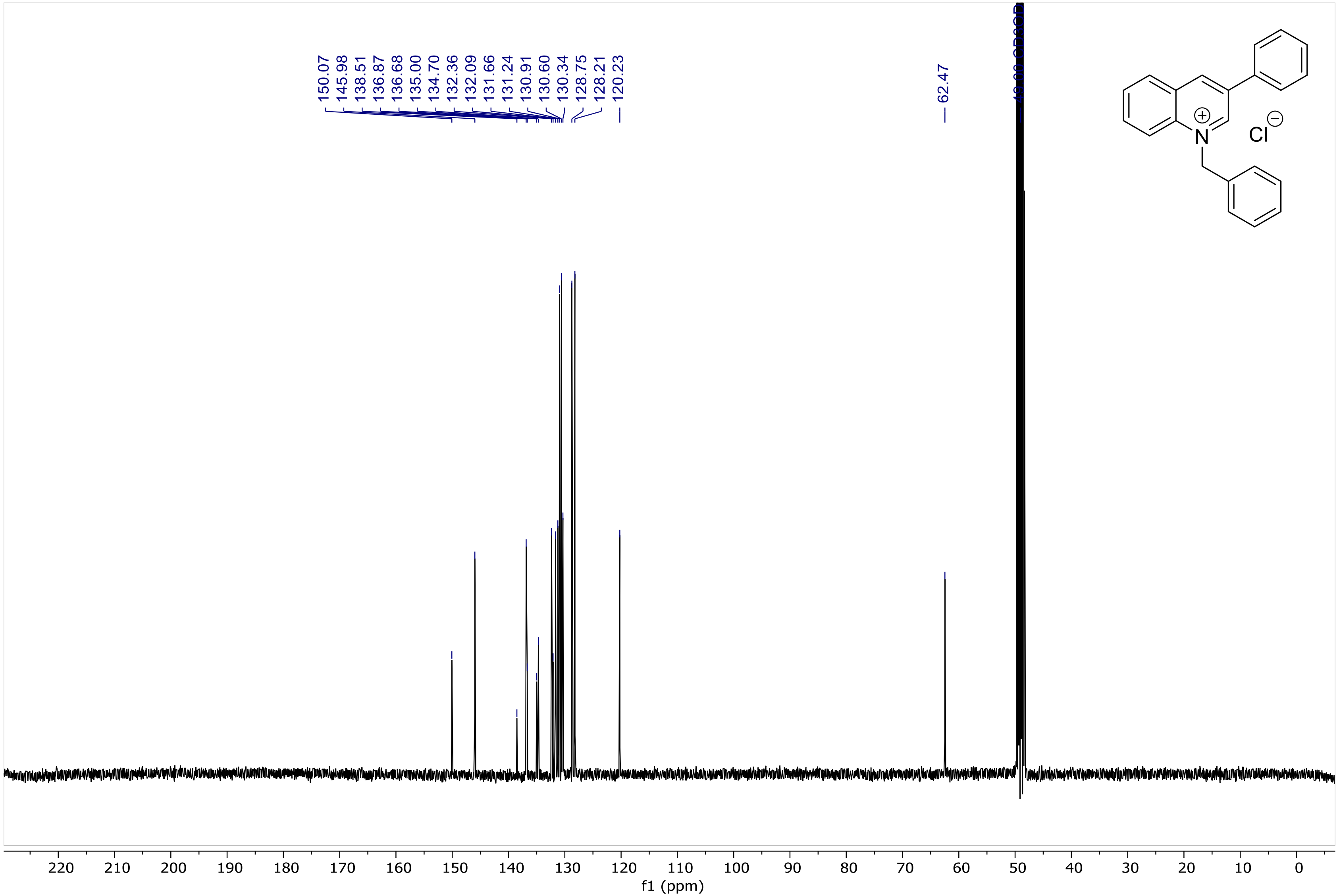

9: 1-Benzyl-3-phenyl-6-(benzyloxy)quinolinium chloride – ^{1}H NMR (400 MHz, CD_3OD)

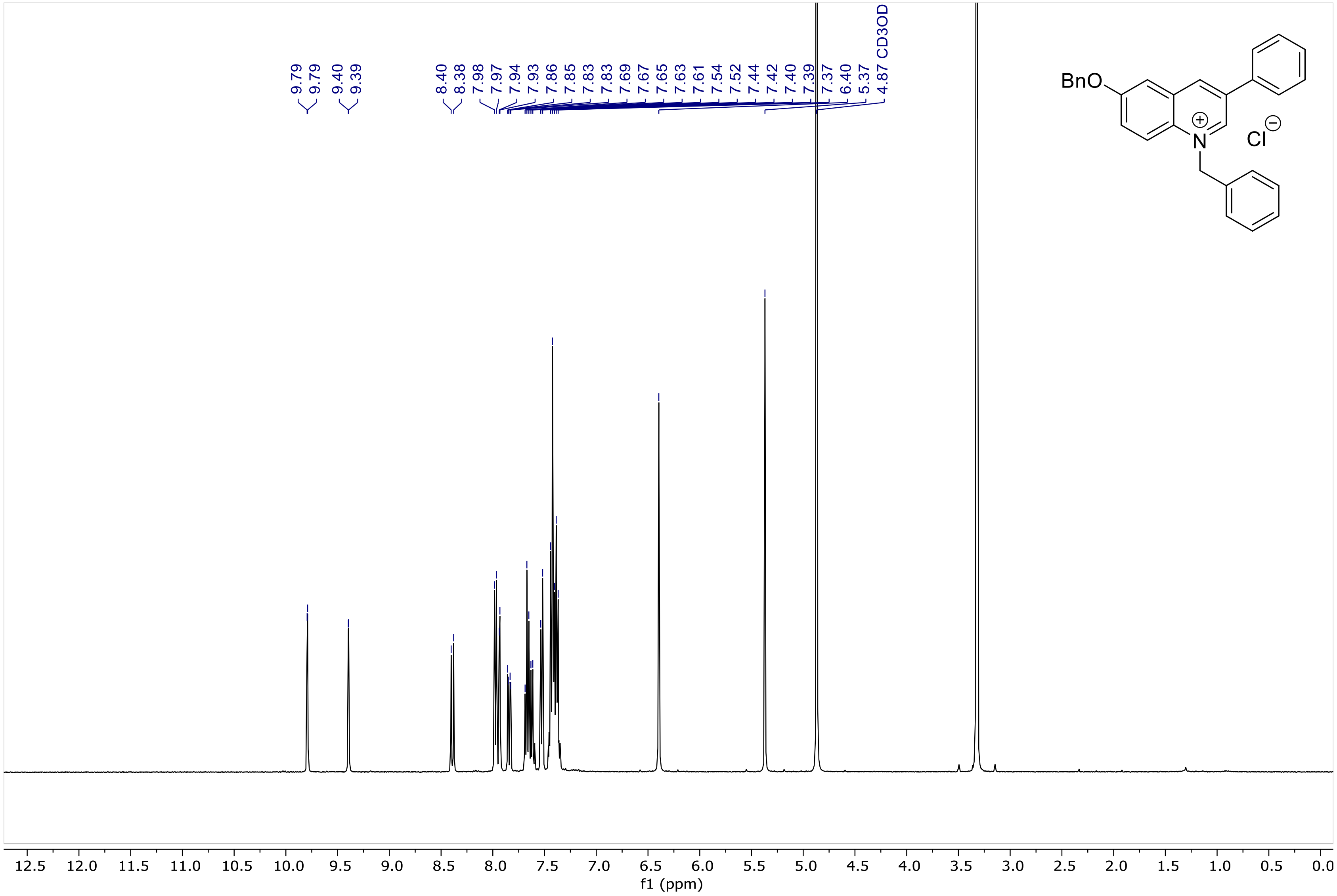

9: 1-Benzyl-3-phenyl-6-(benzyloxy)quinolinium chloride – $^{13}C\{^{1}H\}$ NMR (101 MHz, CD_3OD)

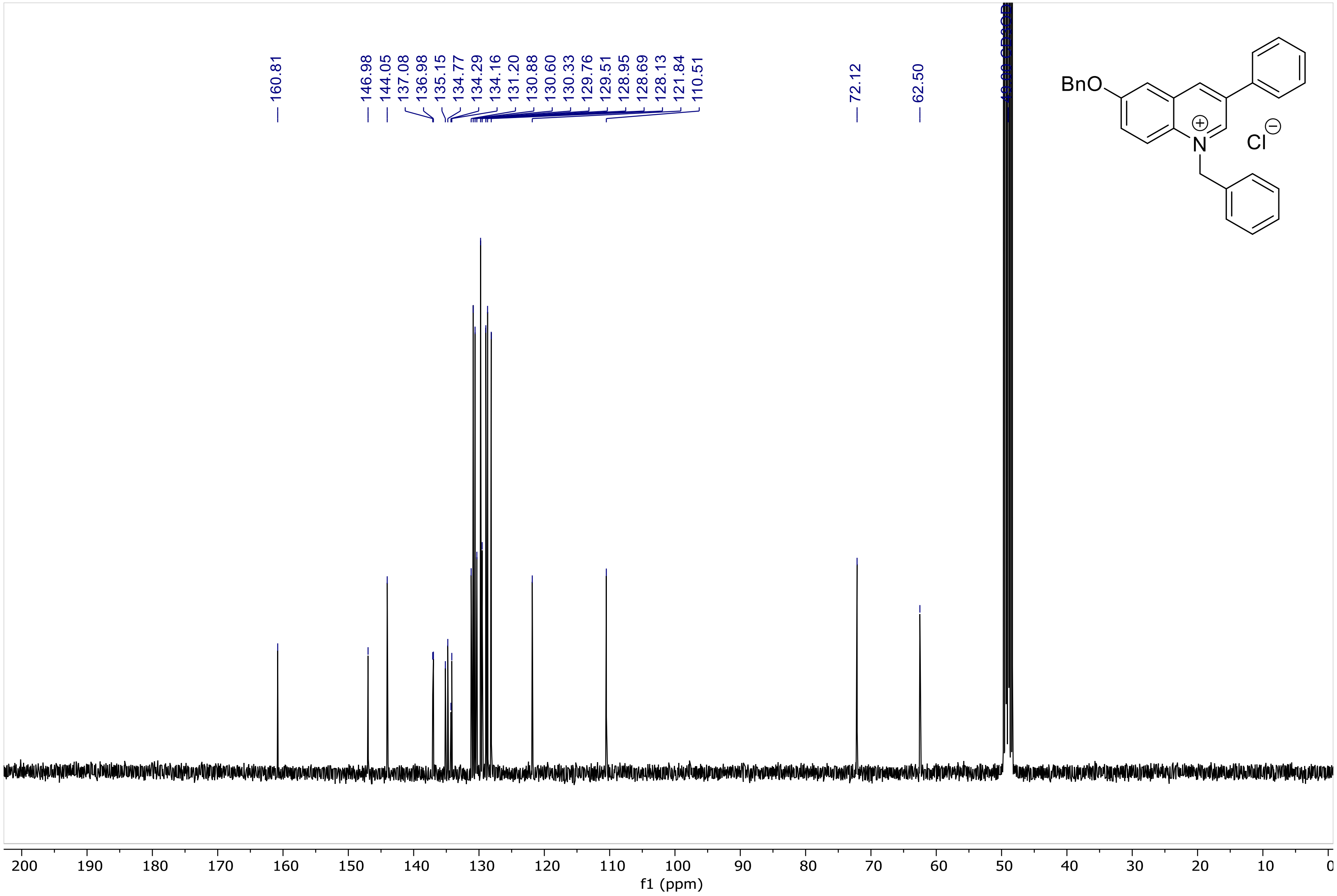

10: 1-Benzyl-3-phenyl-6-methoxyquinolinium chloride – ^{1}H NMR (400 MHz, CD_3OD)

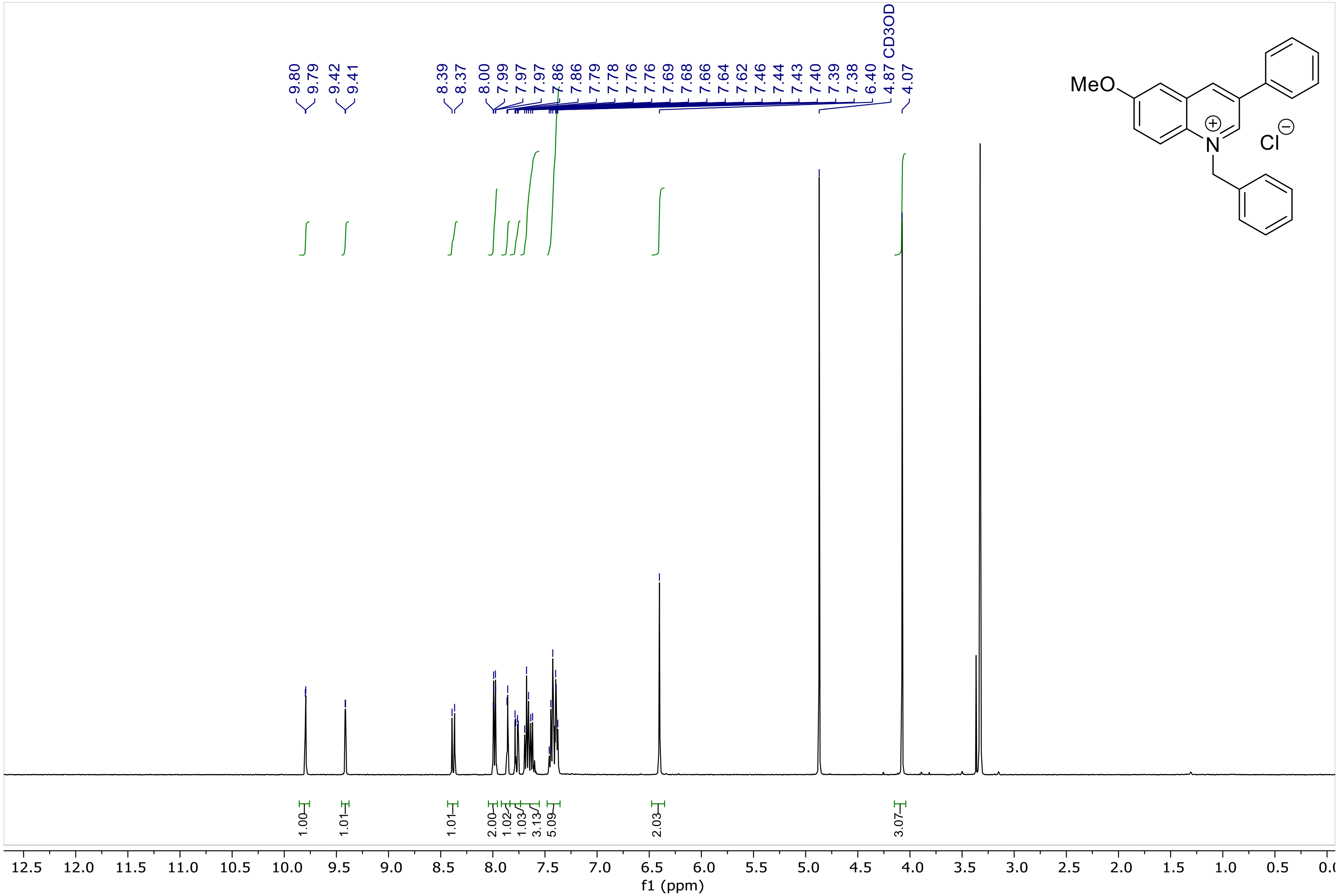

10: 1-Benzyl-3-phenyl-6-methoxyquinolinium chloride – $^{13}C\{^{1}H\}$ NMR (101 MHz, CD_3OD)

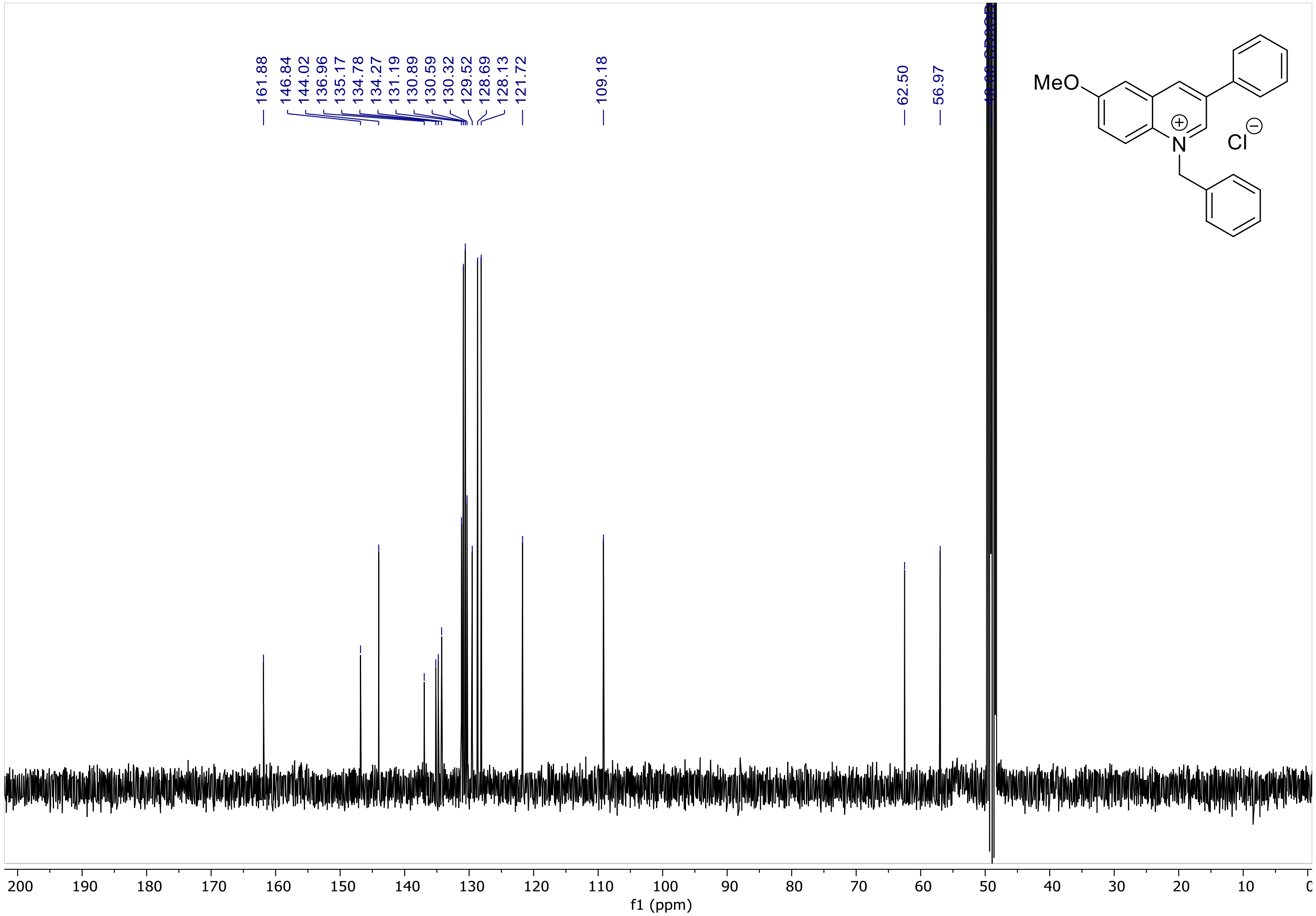

11: 1-Benzyl-3-phenyl-6-methylquinolinium chloride – ^{1}H NMR (400 MHz, CD_3OD)

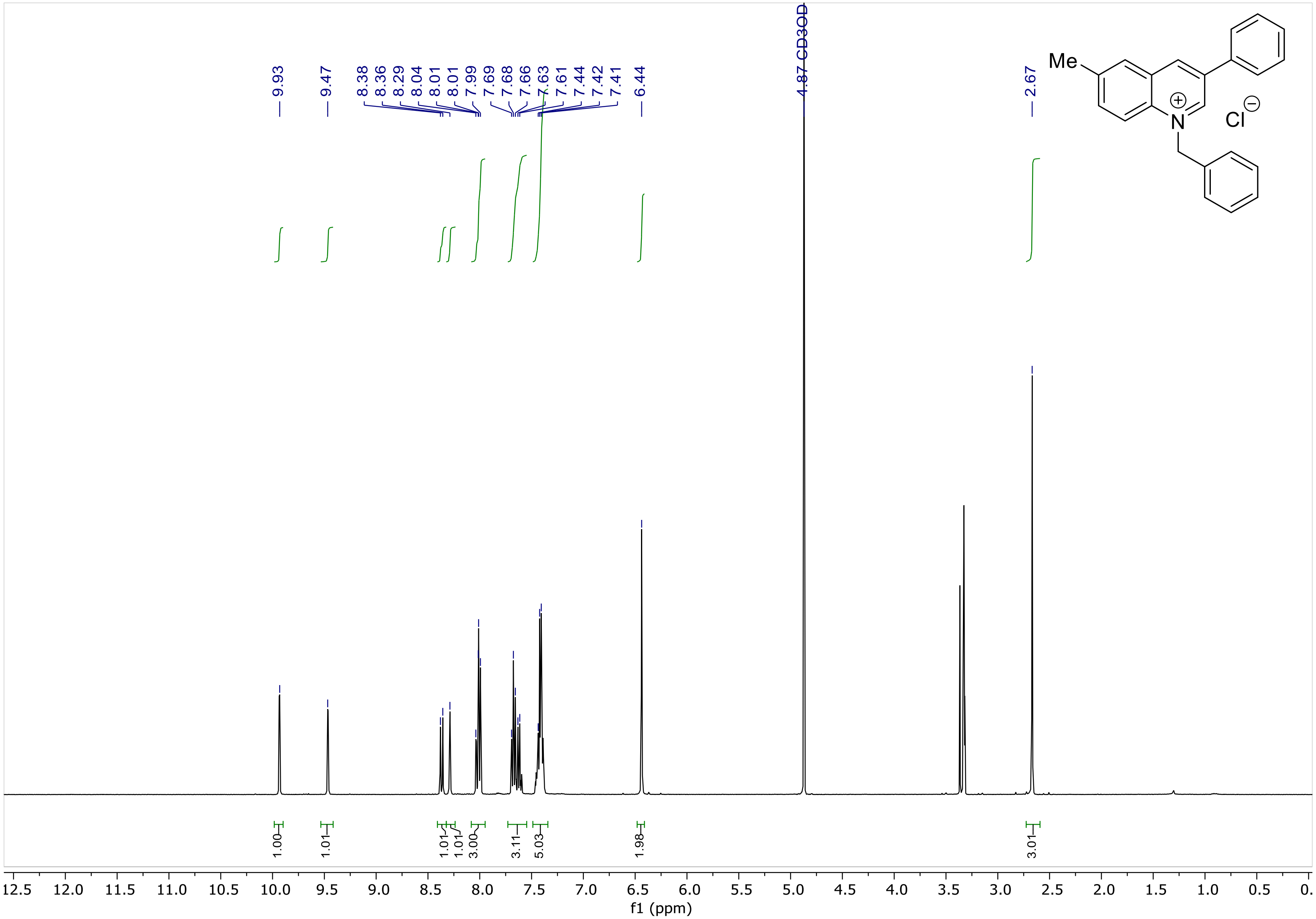

11: 1-Benzyl-3-phenyl-6-methylquinolinium chloride – $^{13}C\{^1H\}$ NMR (101 MHz, CD_3OD)

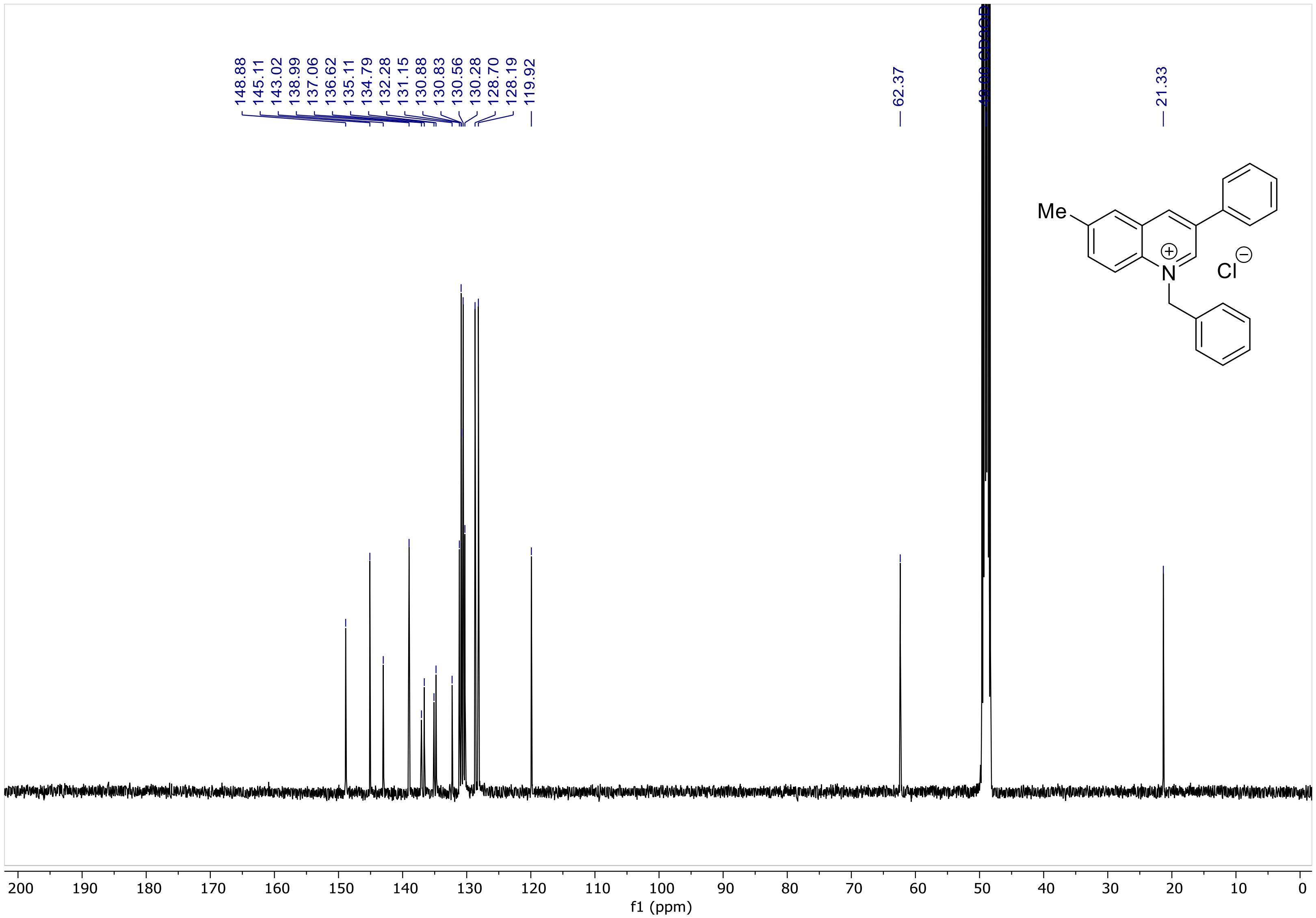

12: 1-Benzyl-3-phenyl-7-(benzyloxy)quinolinium chloride – ^{1}H NMR (400 MHz, CD_3OD)

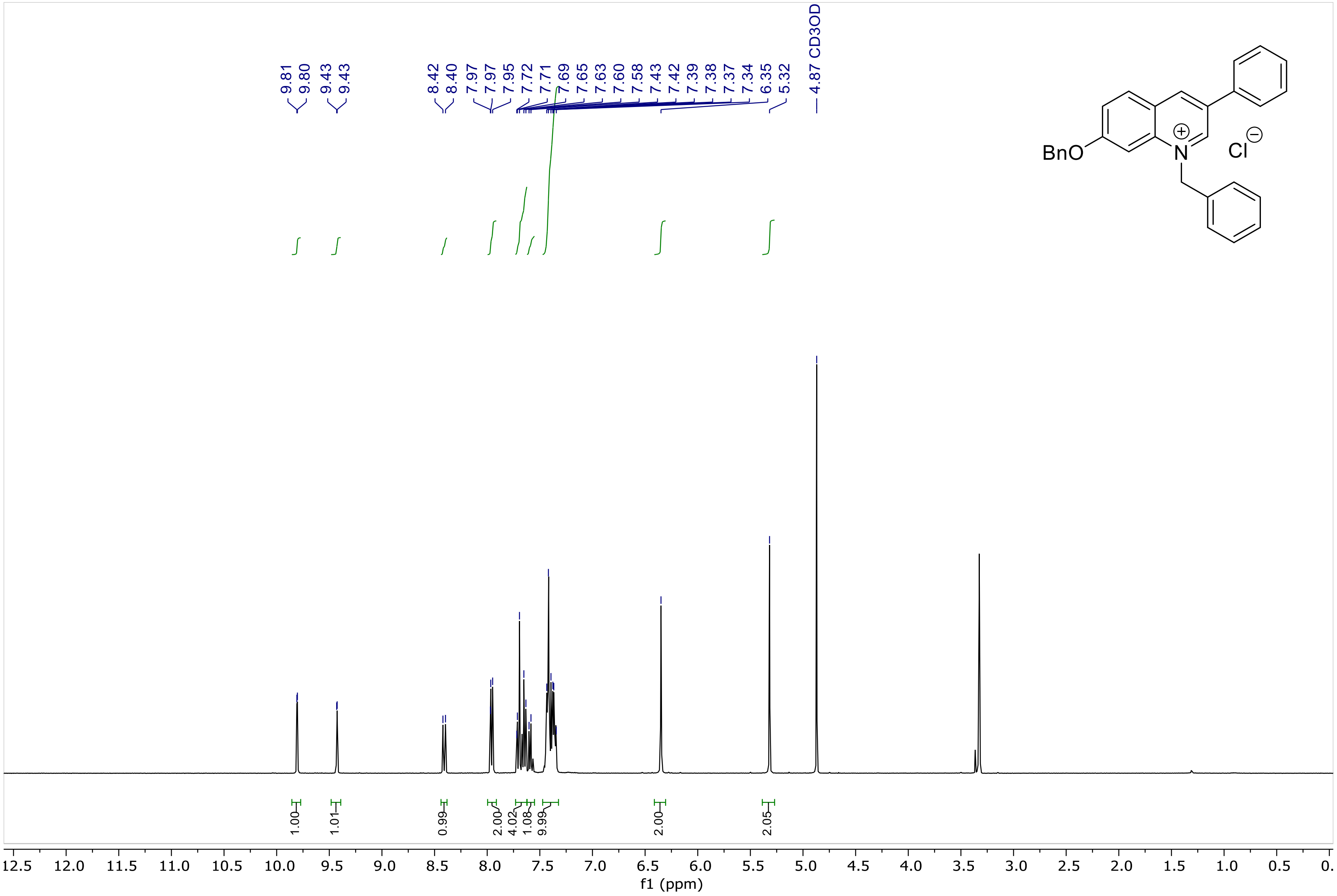

12: 1-Benzyl-3-phenyl-7-(benzyloxy)quinolinium chloride – $^{13}C\{^{1}H\}$ NMR (101 MHz, CD_3OD)

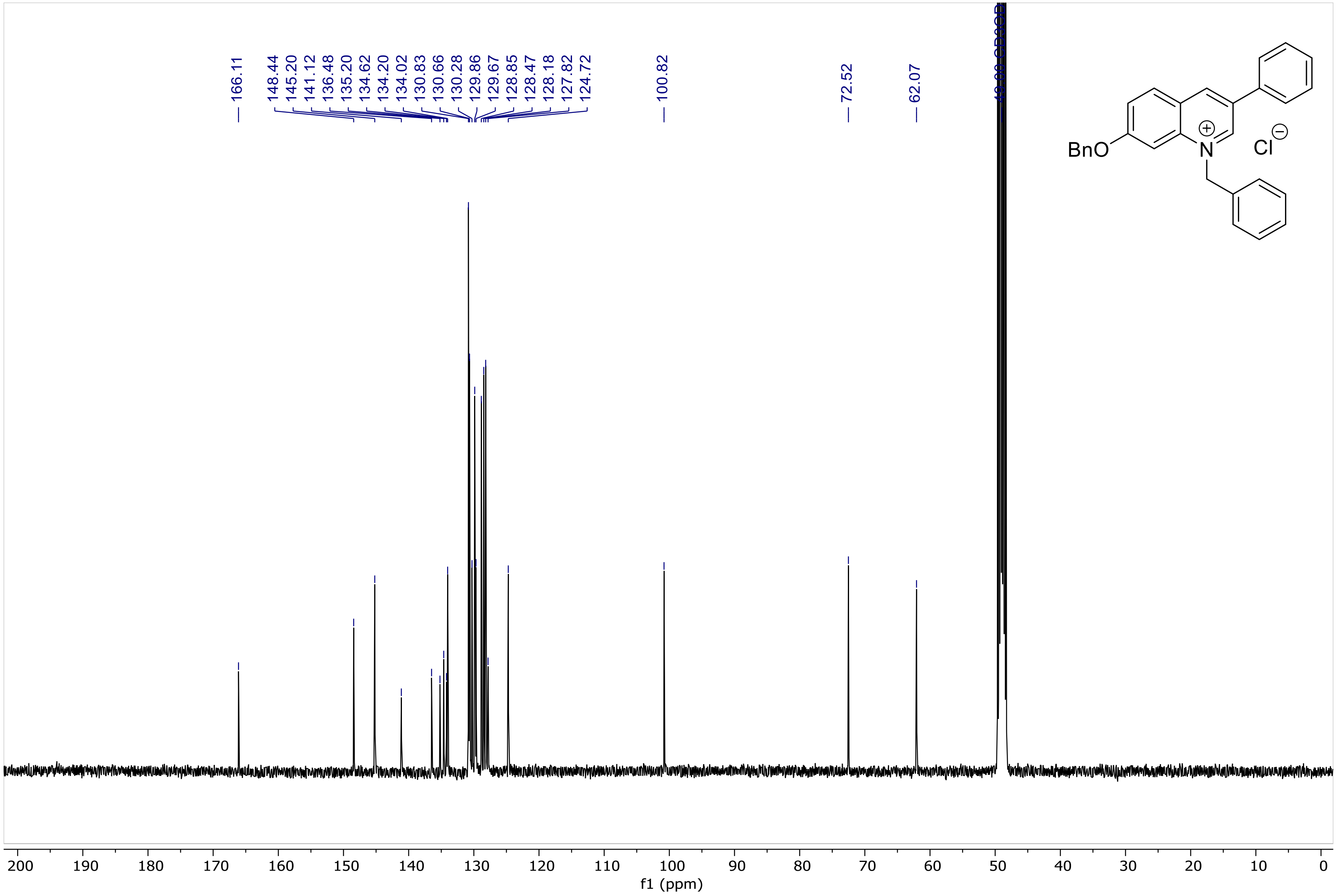

13: 1-Benzyl-3-phenyl-6,7-dimethoxyquinolinium chloride – ^{1}H NMR (400 MHz, CD_3OD)

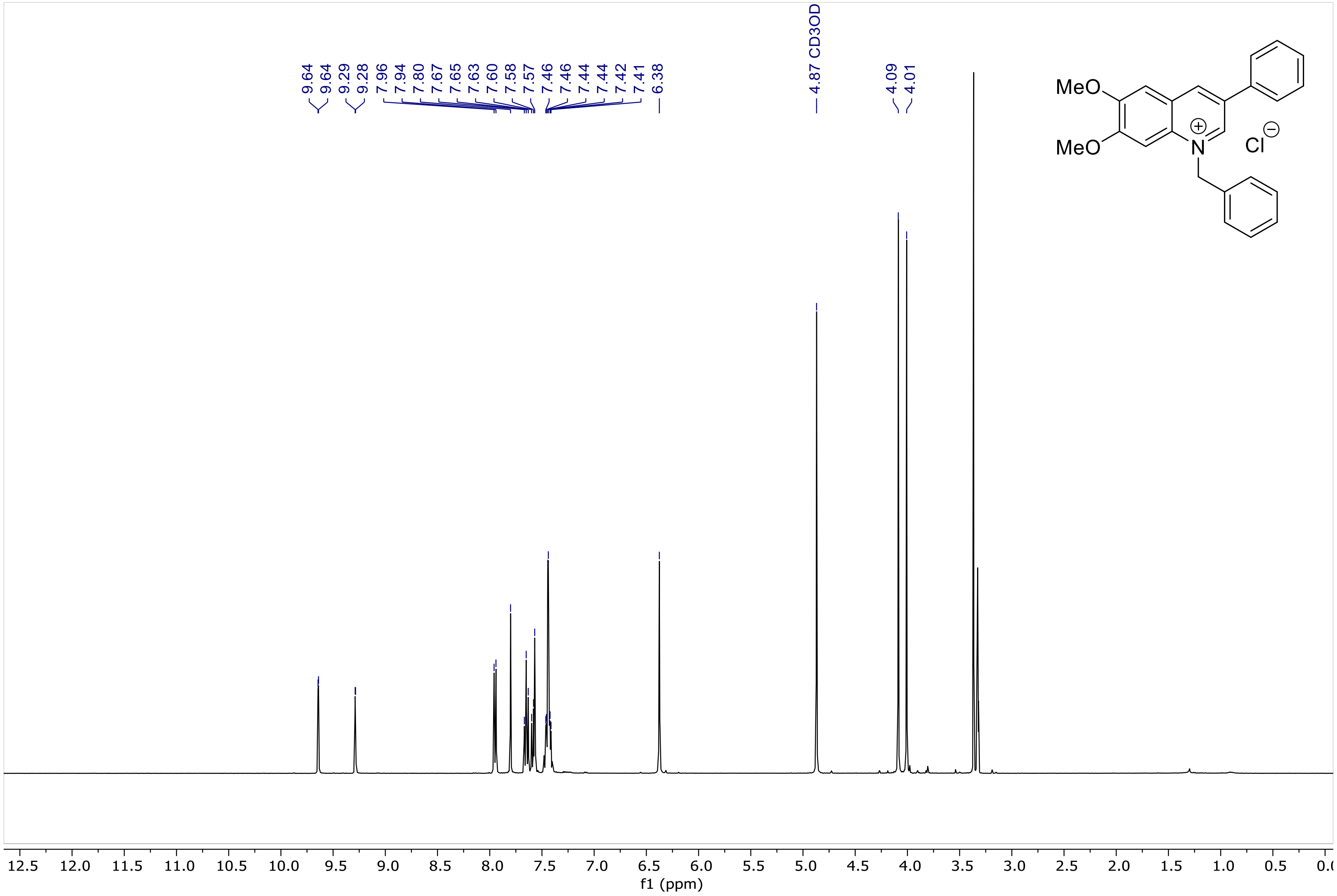

13: 1-Benzyl-3-phenyl-6,7-dimethoxyquinolinium chloride – $^{13}C\{^{1}H\}$ NMR (101 MHz, CD_3OD)

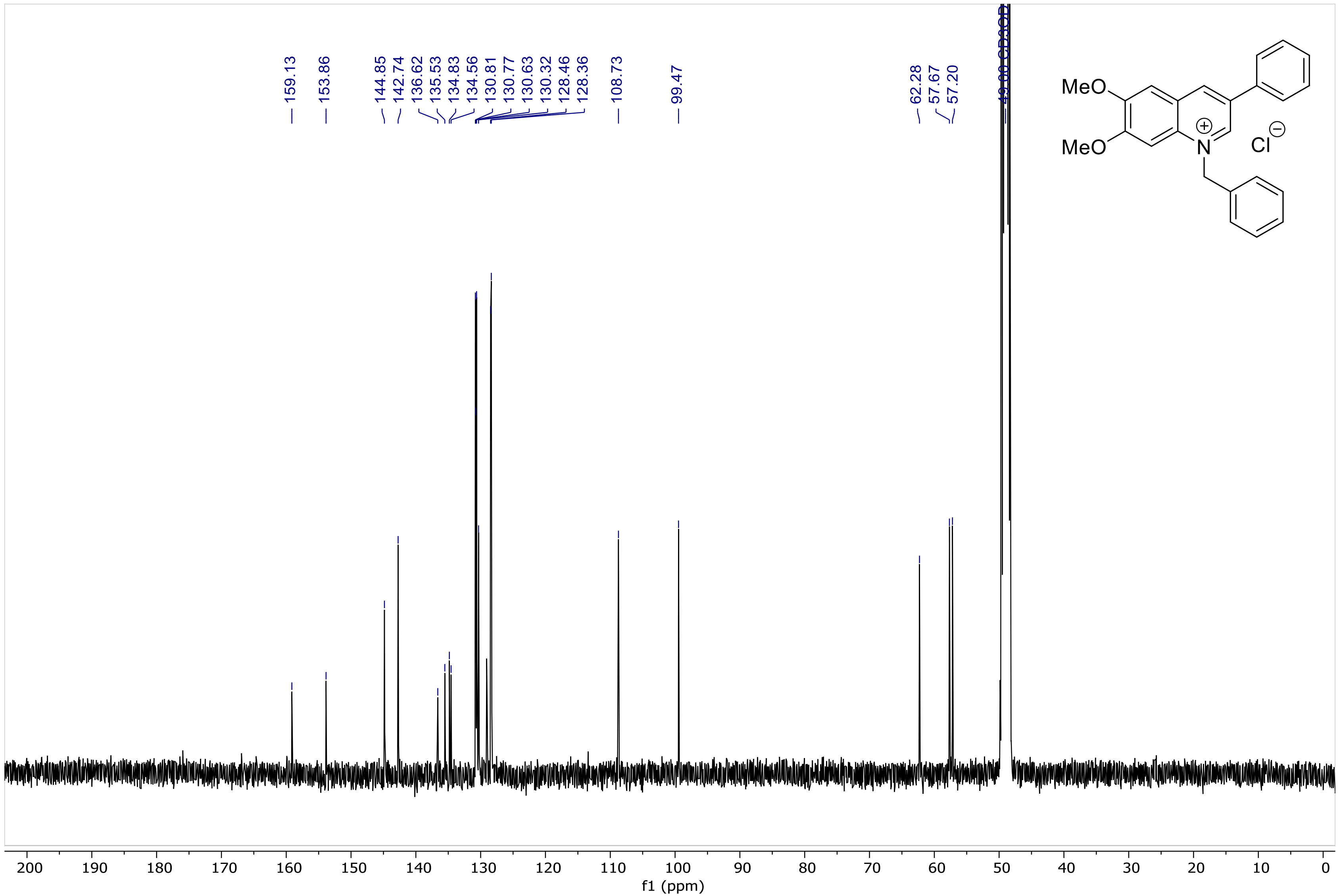

14: 1-Benzyl-3-phenyl-5-methylquinolinium chloride – ^{1}H NMR (400 MHz, CD_3OD)

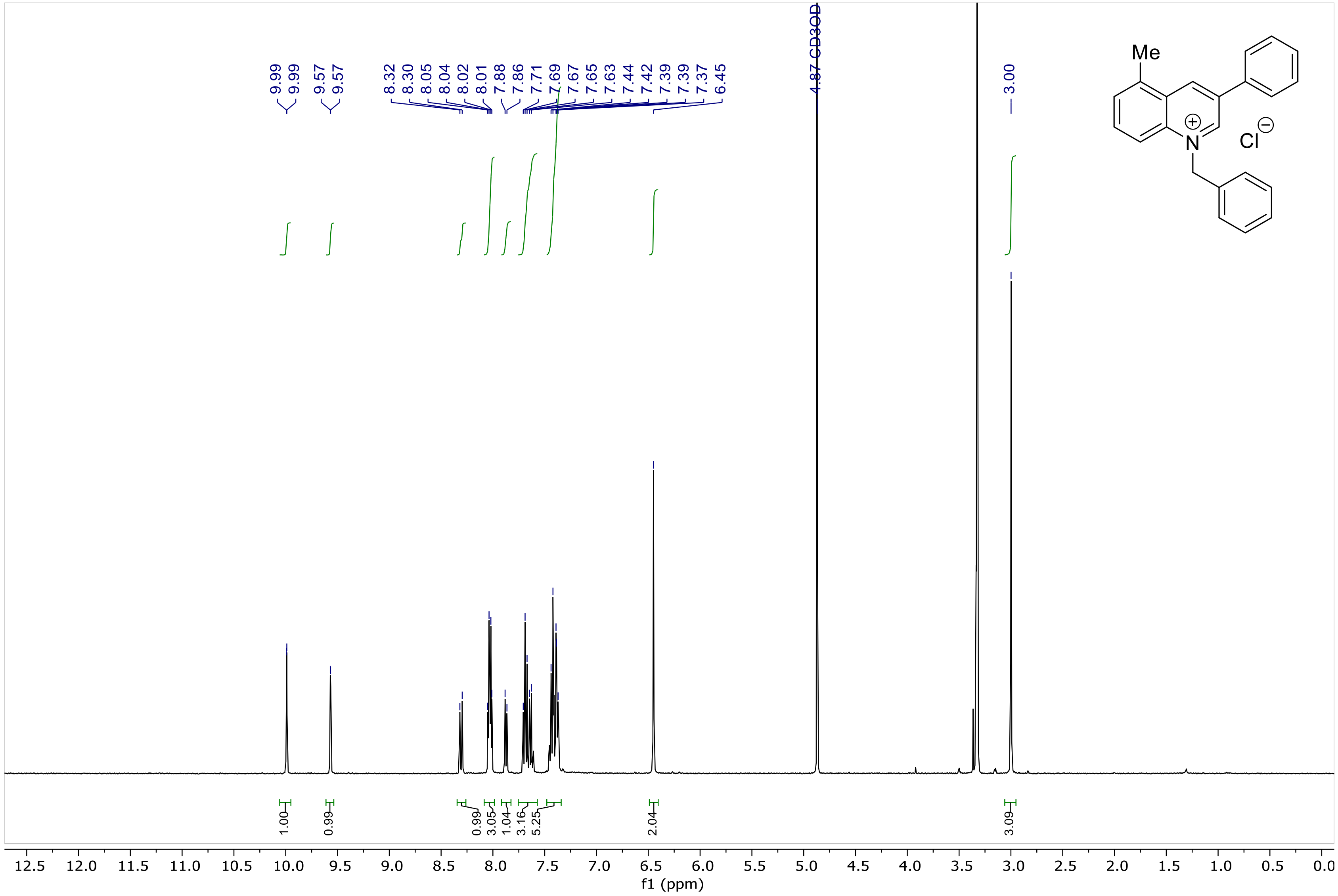

14: 1-Benzyl-3-phenyl-5-methylquinolinium chloride – $^{13}C\{^1H\}$ NMR (101 MHz, CD_3OD)

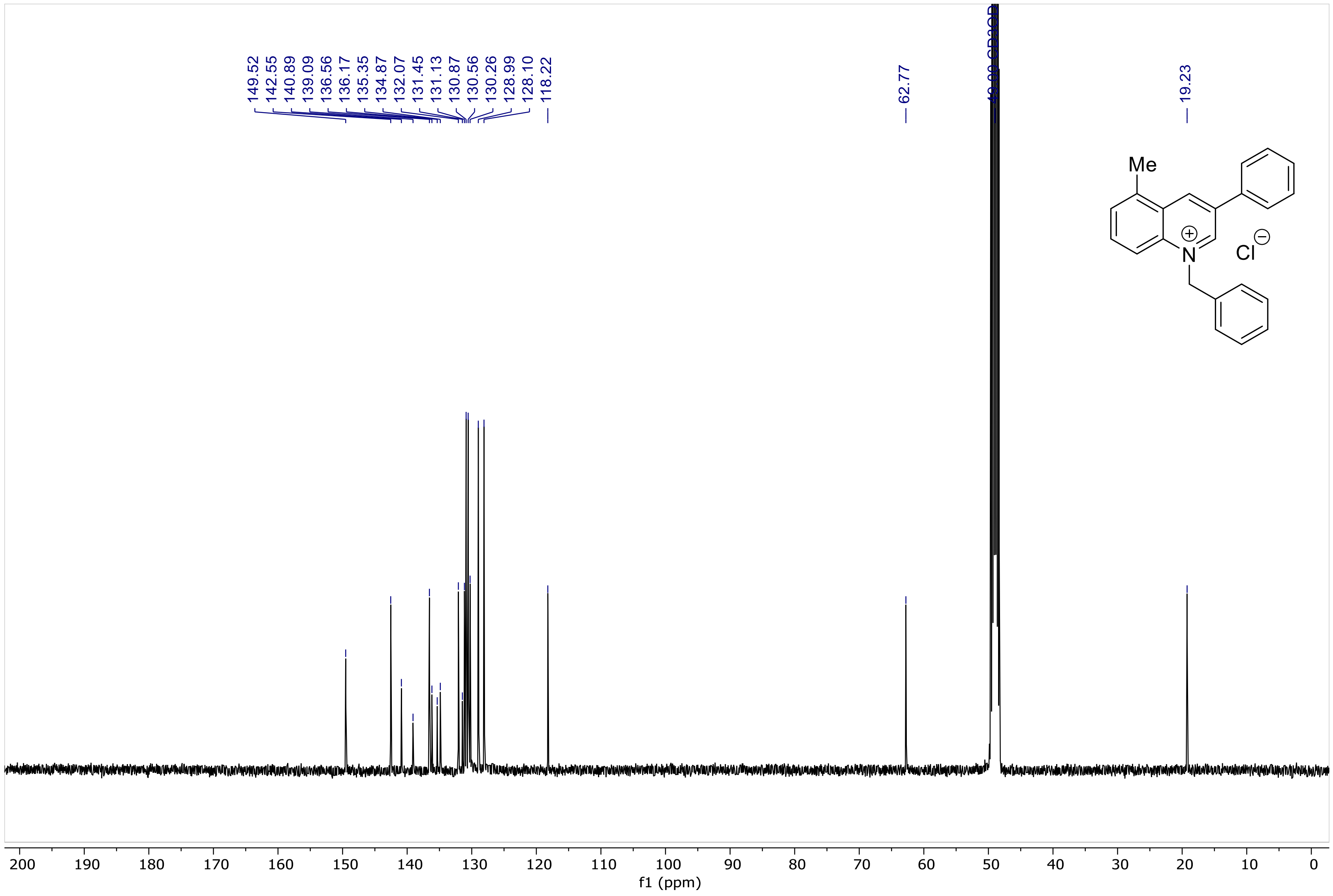

15: 1-Benzyl-3-phenyl-8-methylquinolinium chloride – ^{1}H NMR (400 MHz, CD_3OD)

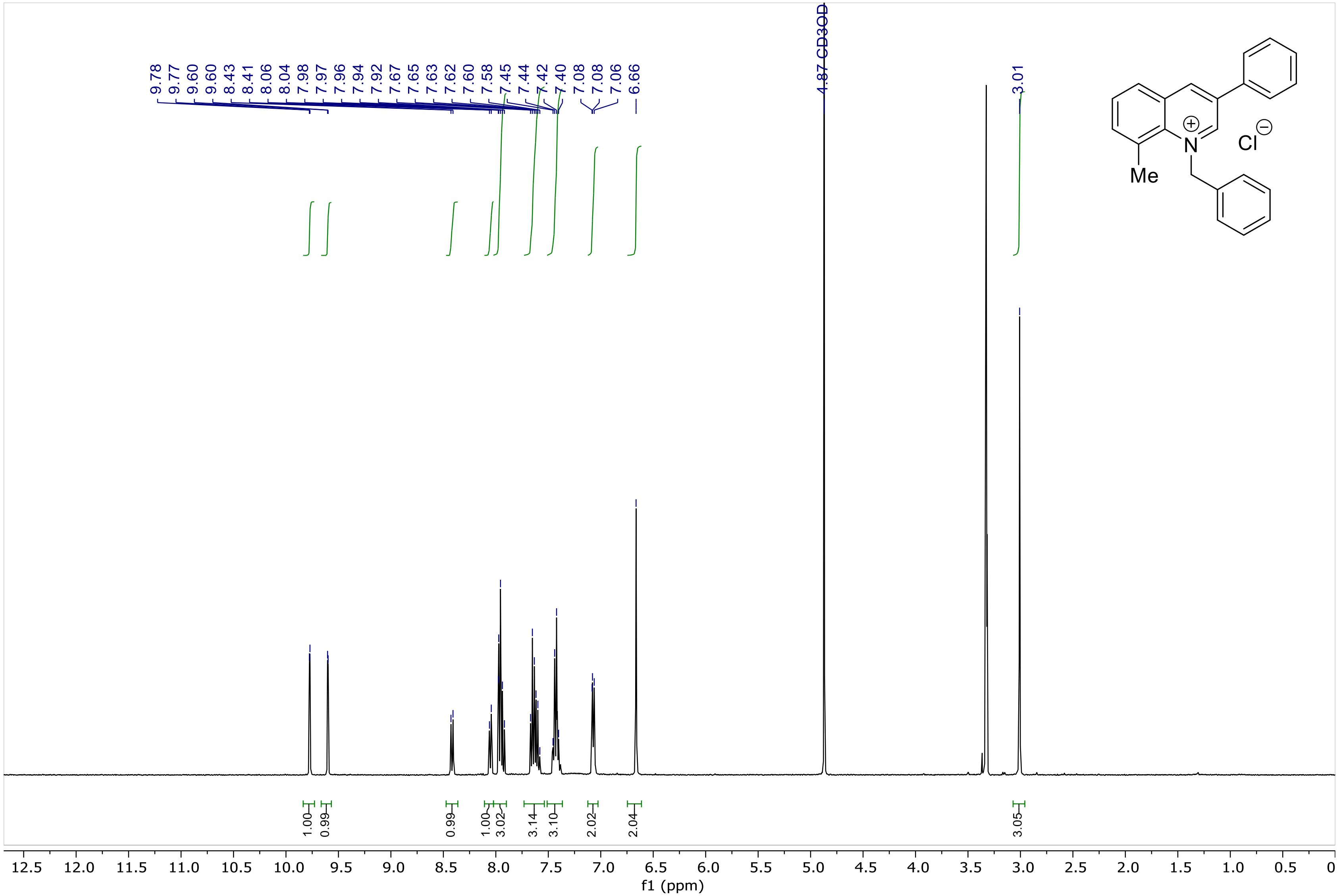

15: 1-Benzyl-3-phenyl-8-methylquinolinium chloride – $^{13}C\{^1H\}$ NMR (101 MHz, CD_3OD)

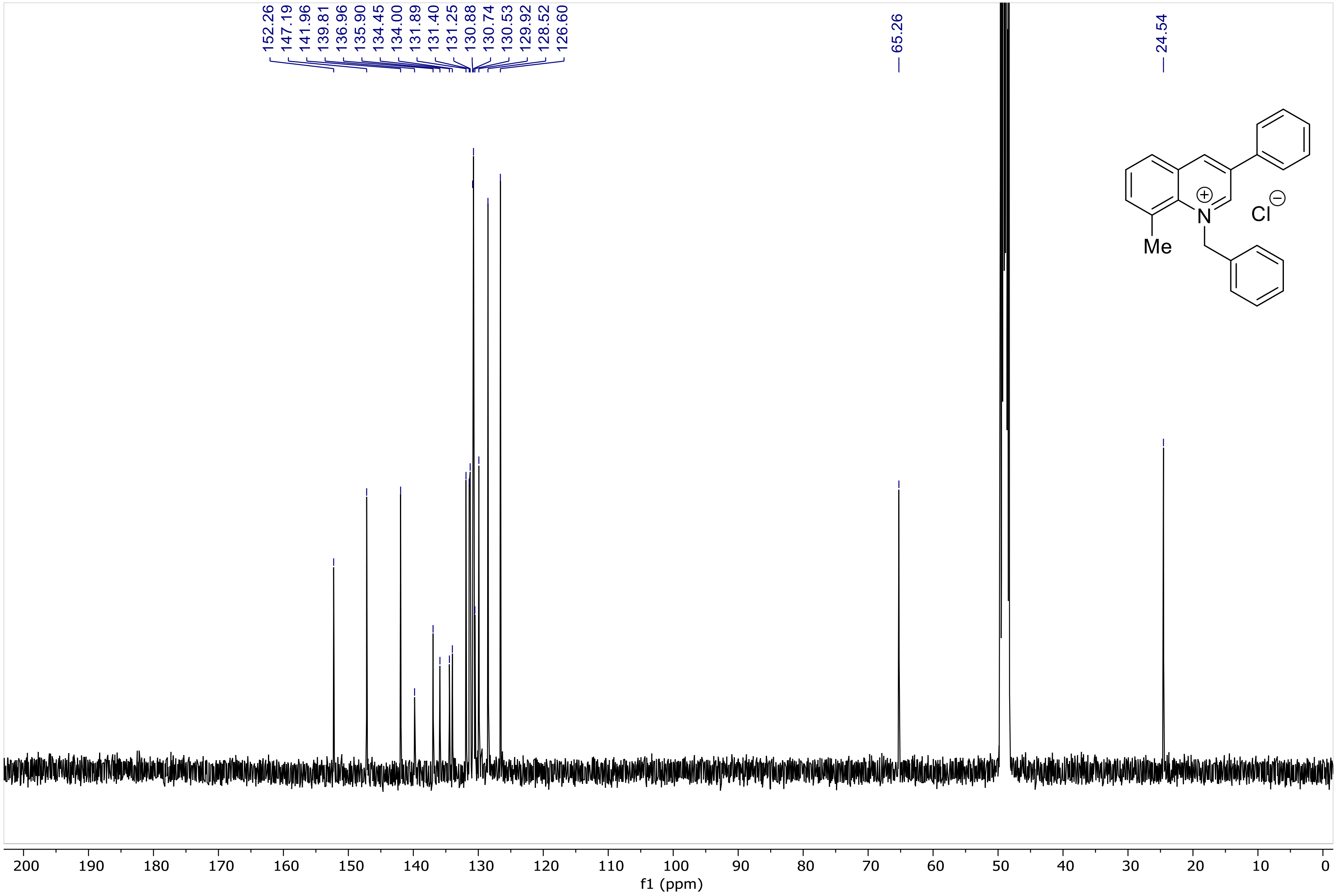

18: 1-Benzyl-3-phenyl-2,4-dimethylquinolinium chloride – ^{1}H NMR (400 MHz, CD_3OD)

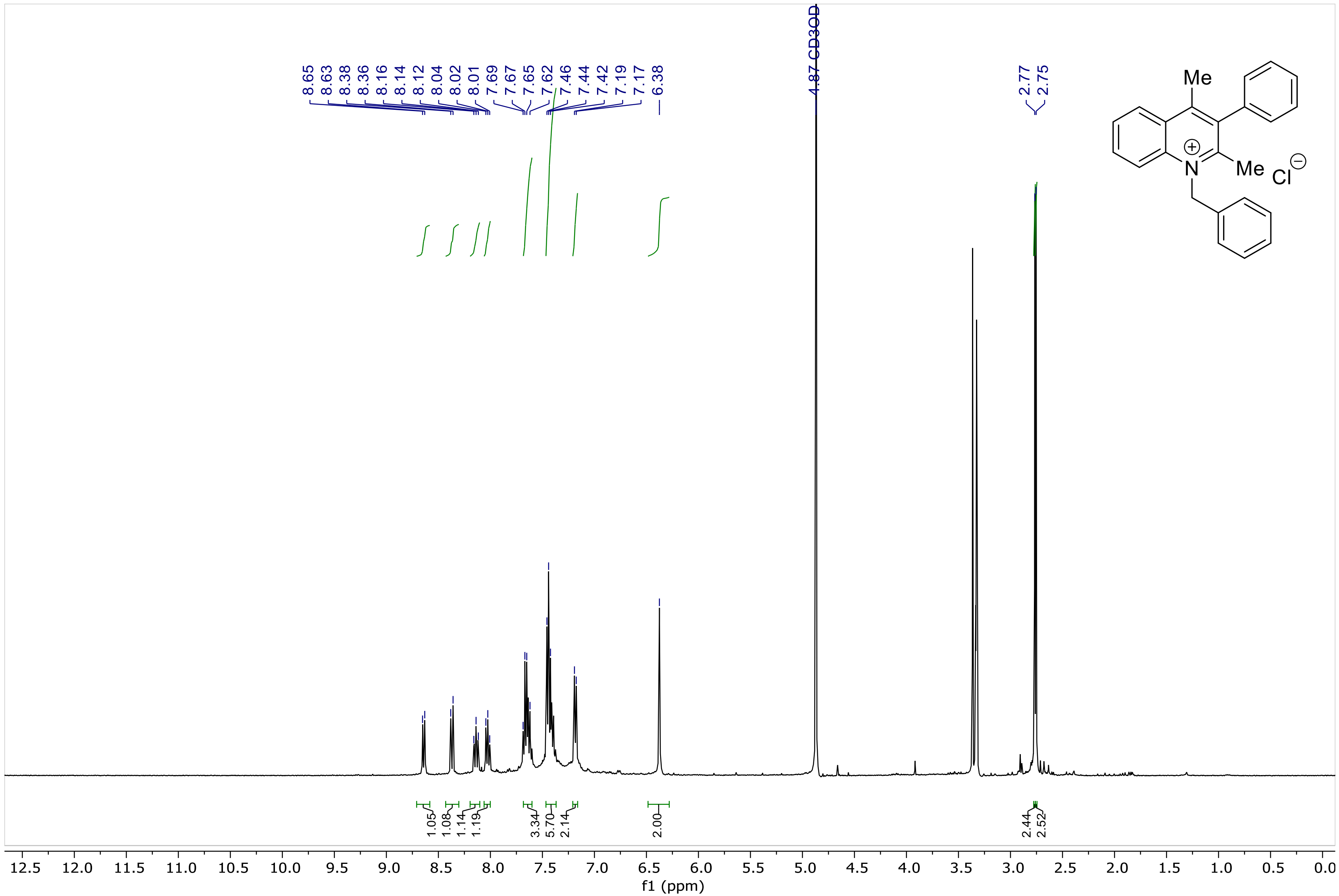

18: 1-Benzyl-3-phenyl-2,4-dimethylquinolinium chloride – $^{13}C\{^1H\}$ NMR (101 MHz, CD_3OD)

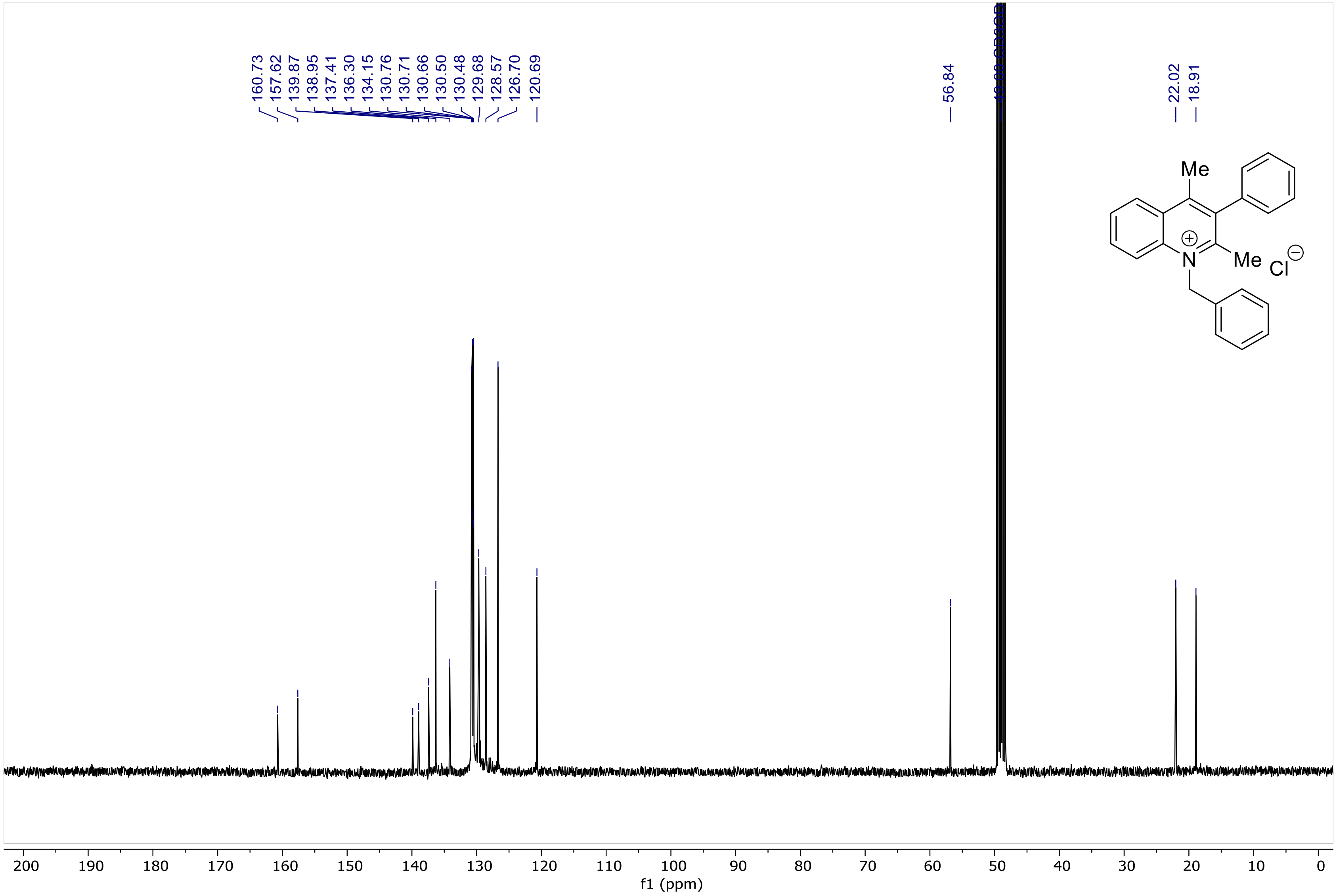

19: 1-methyl-6-fluoro-3-phenylquinolinium chloride – ^{1}H NMR (400 MHz, CD_3OD)

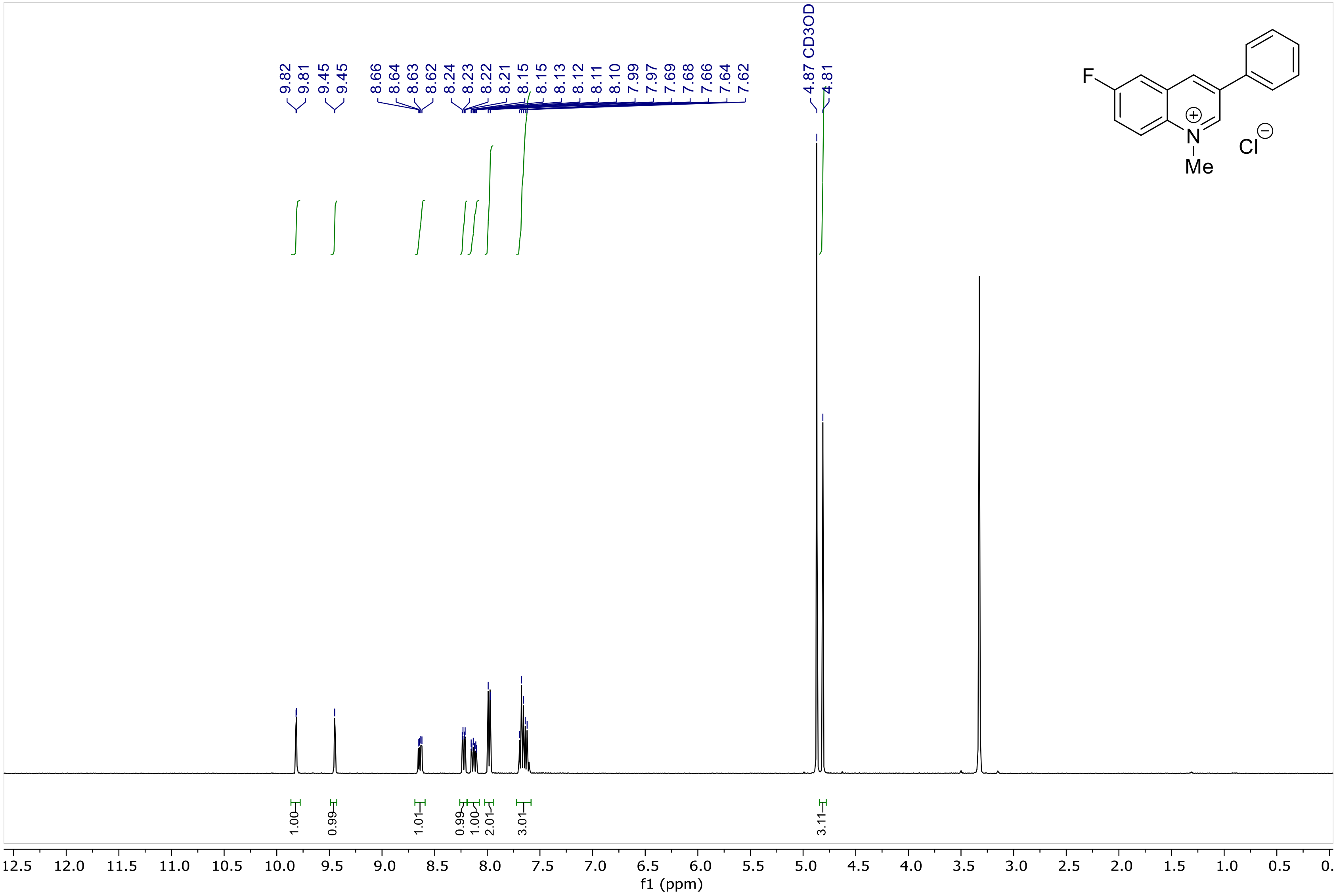

19: 1-methyl-6-fluoro-3-phenylquinolinium chloride – $^{13}C\{^1H\}$ NMR (101 MHz, CD_3OD)

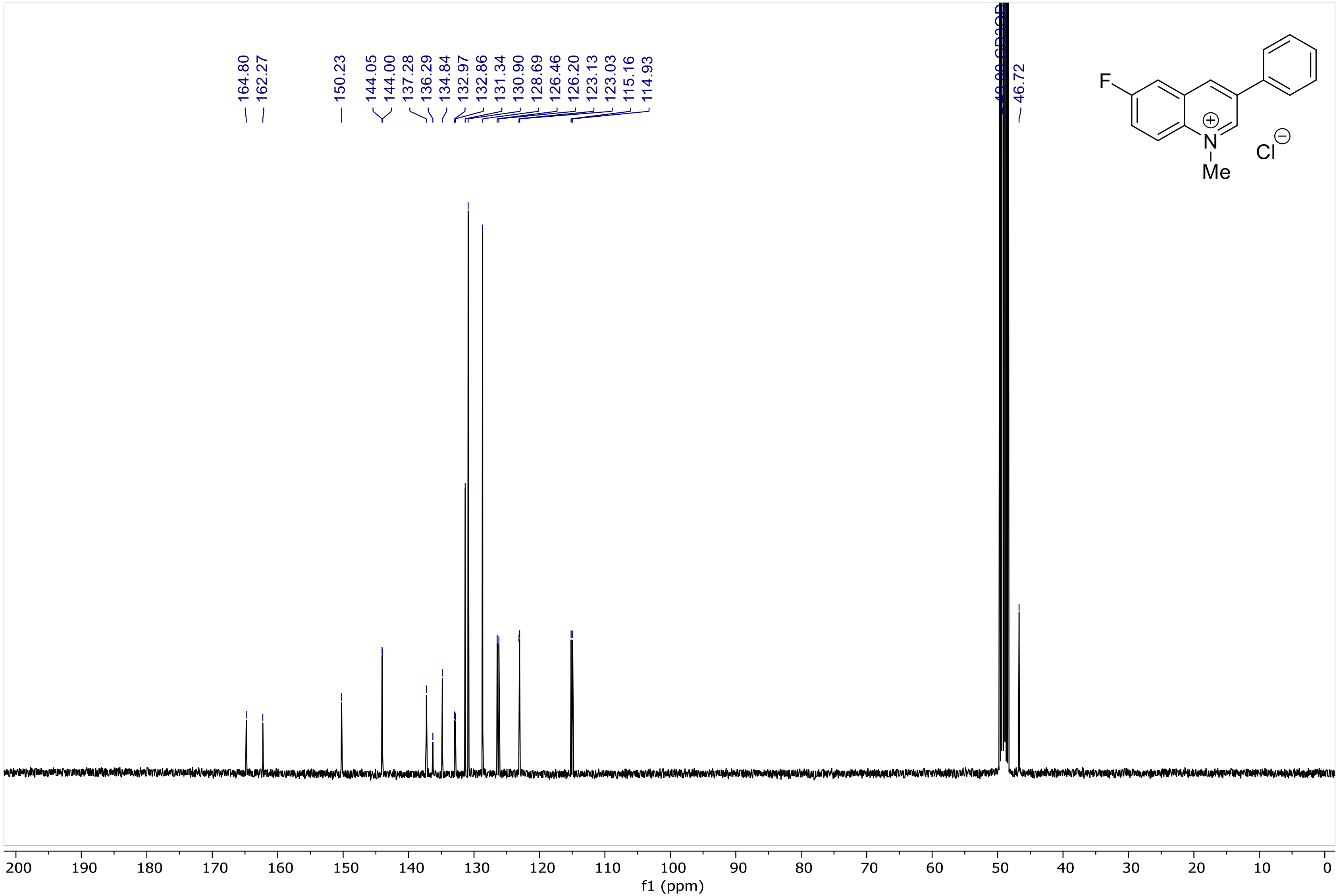

19: 1-methyl-6-fluoro-3-phenylquinolinium chloride – ^{19}F NMR (376 MHz, CD_3OD)

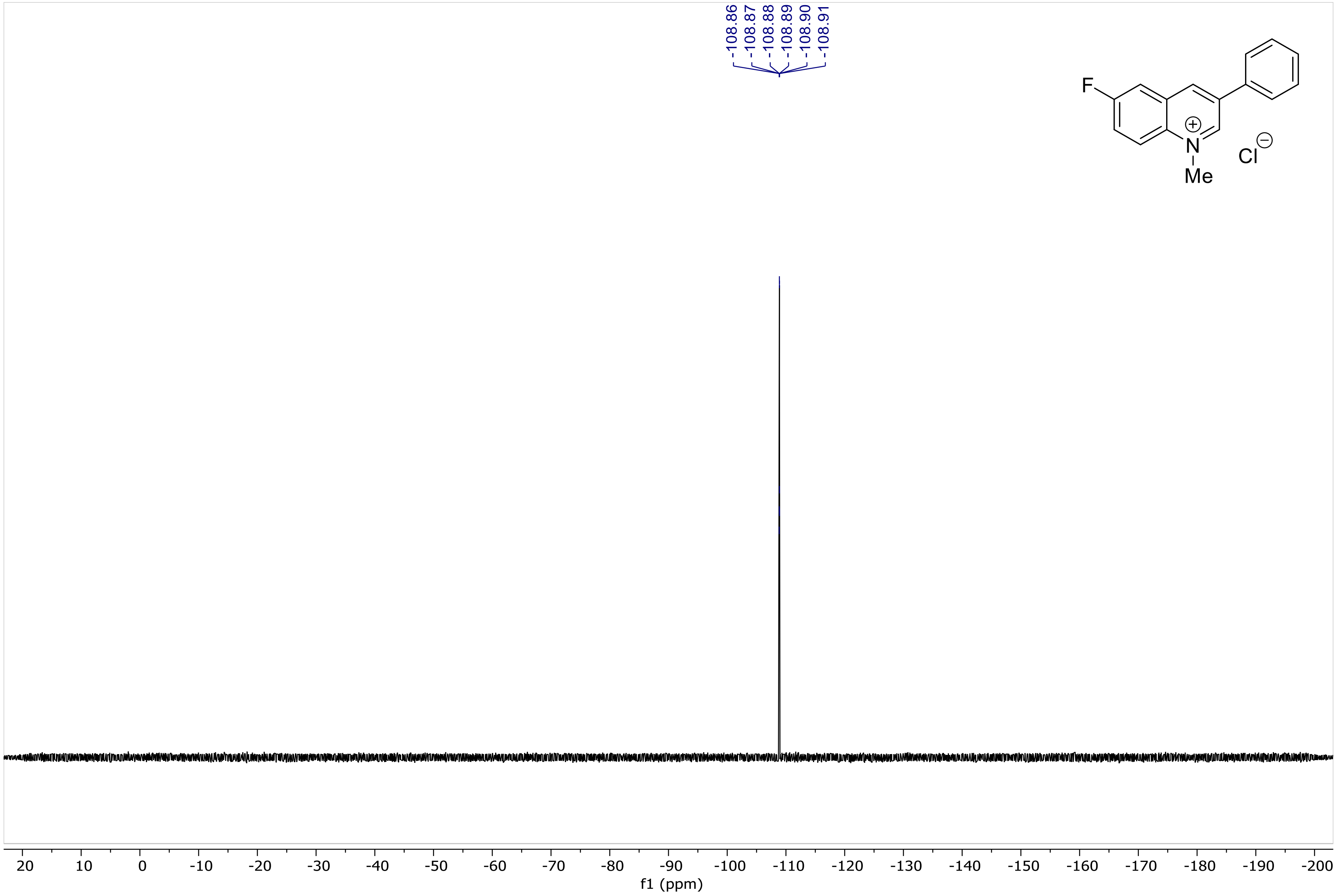

20: 1-(4-methoxybenzyl)-6-fluoro-3-phenylquinolinium chloride – ^{1}H NMR (400 MHz, CD_3OD)

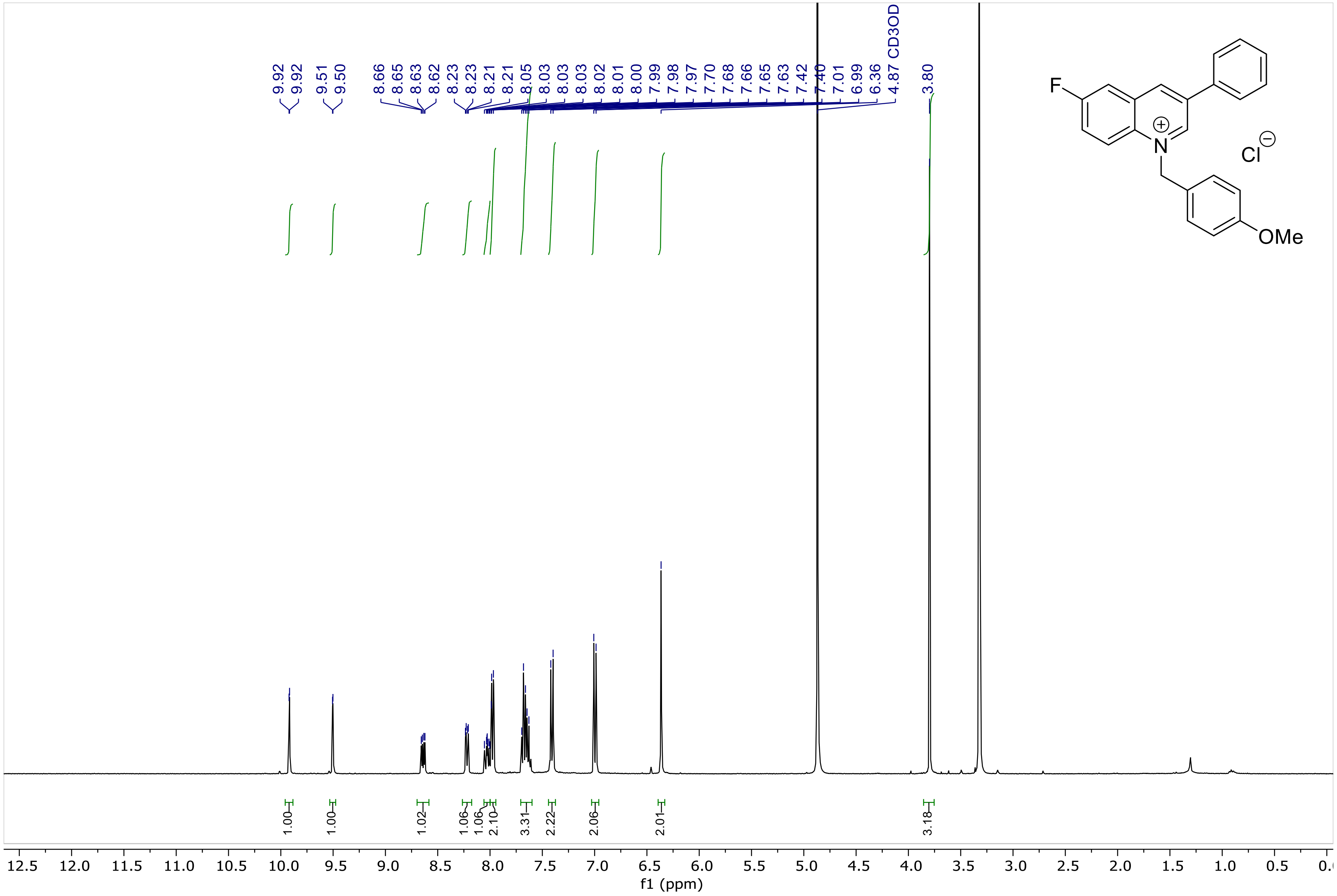

20: 1-(4-methoxybenzyl)-6-fluoro-3-phenylquinolinium chloride – $^{13}C\{^1H\}$ NMR (101 MHz, CD_3OD)

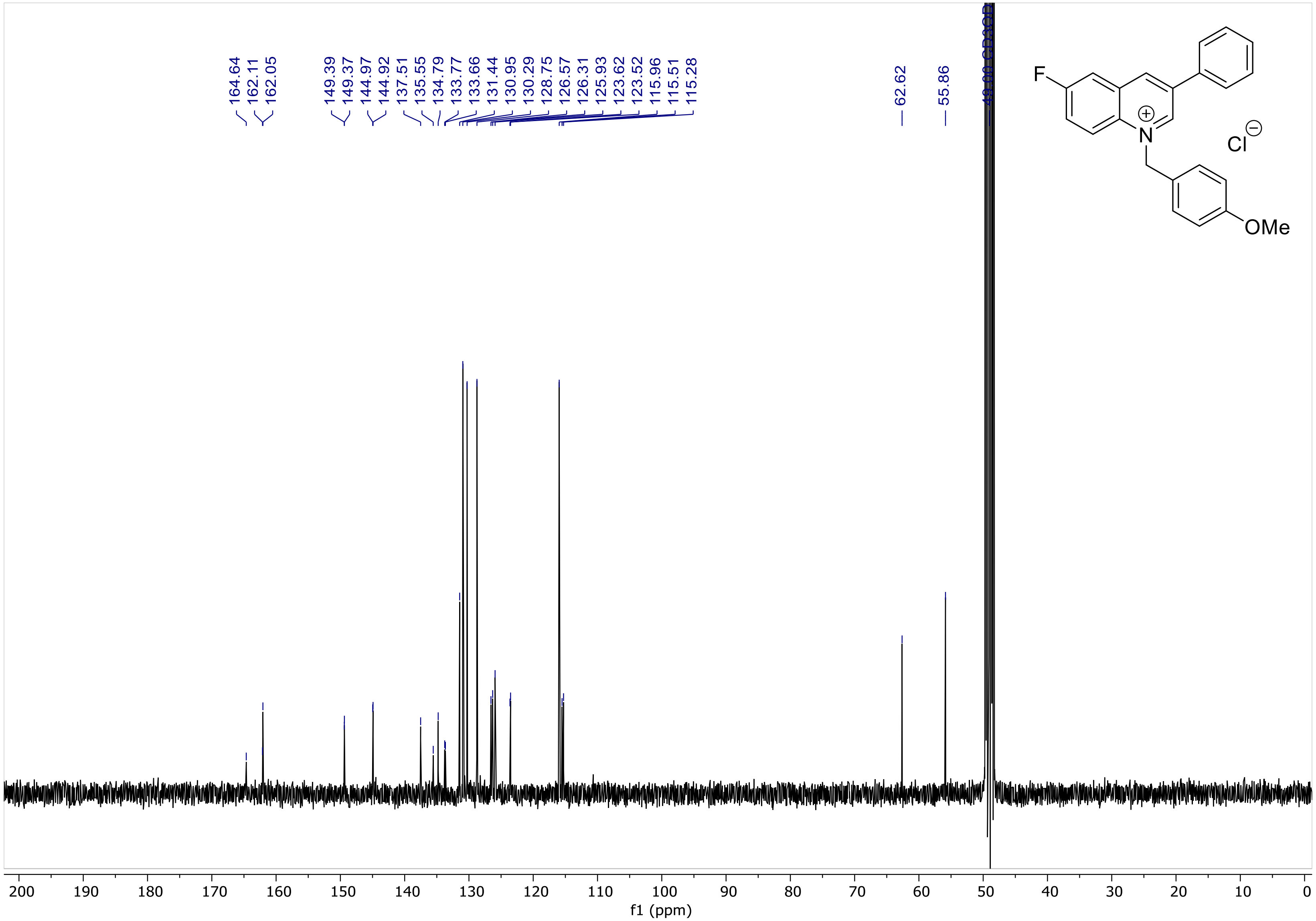

20: 1-(4-methoxybenzyl)-6-fluoro-3-phenylquinolinium chloride – ^{19}F NMR (376 MHz, CD_3OD)

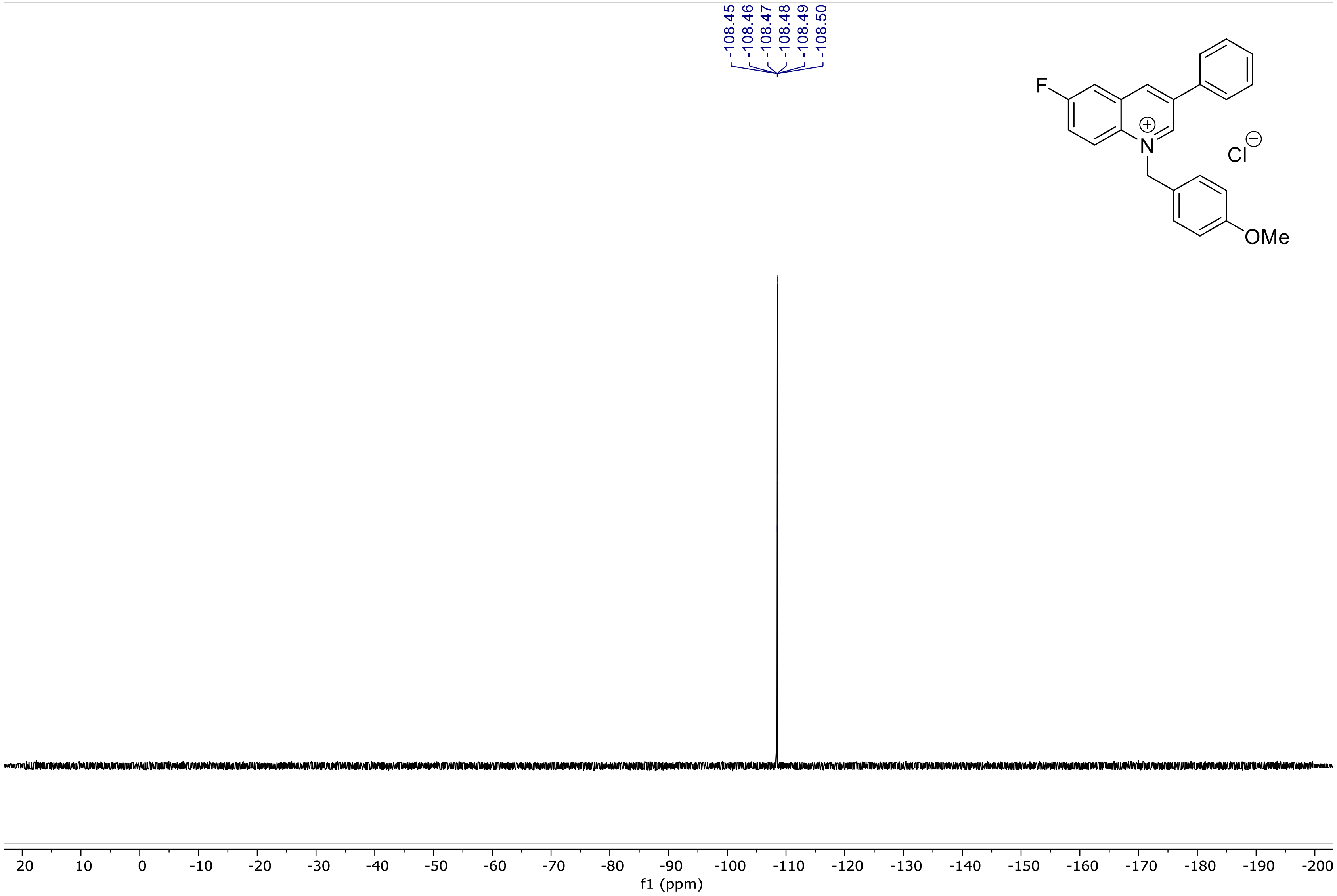

22: 4-(2-acetamidoethyl)-1-benzyl-6-methoxy-3-phenylquinolinium chloride – ^{1}H NMR (400 MHz, CD_3OD)

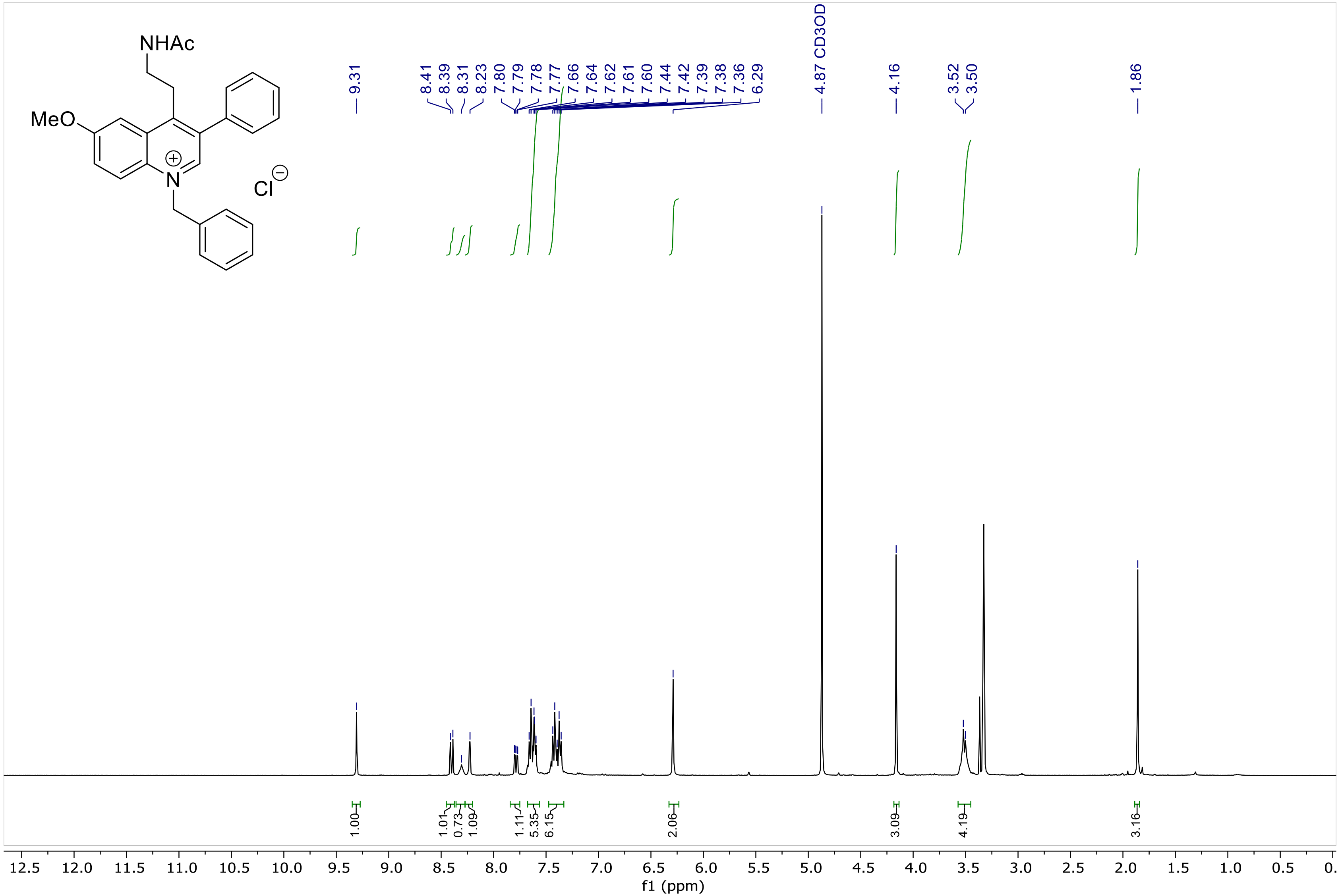

22: 4-(2-acetamidoethyl)-1-benzyl-6-methoxy-3-phenylquinolinium chloride – $^{13}C\{^{1}H\}$ NMR (101 MHz, CD_3OD)

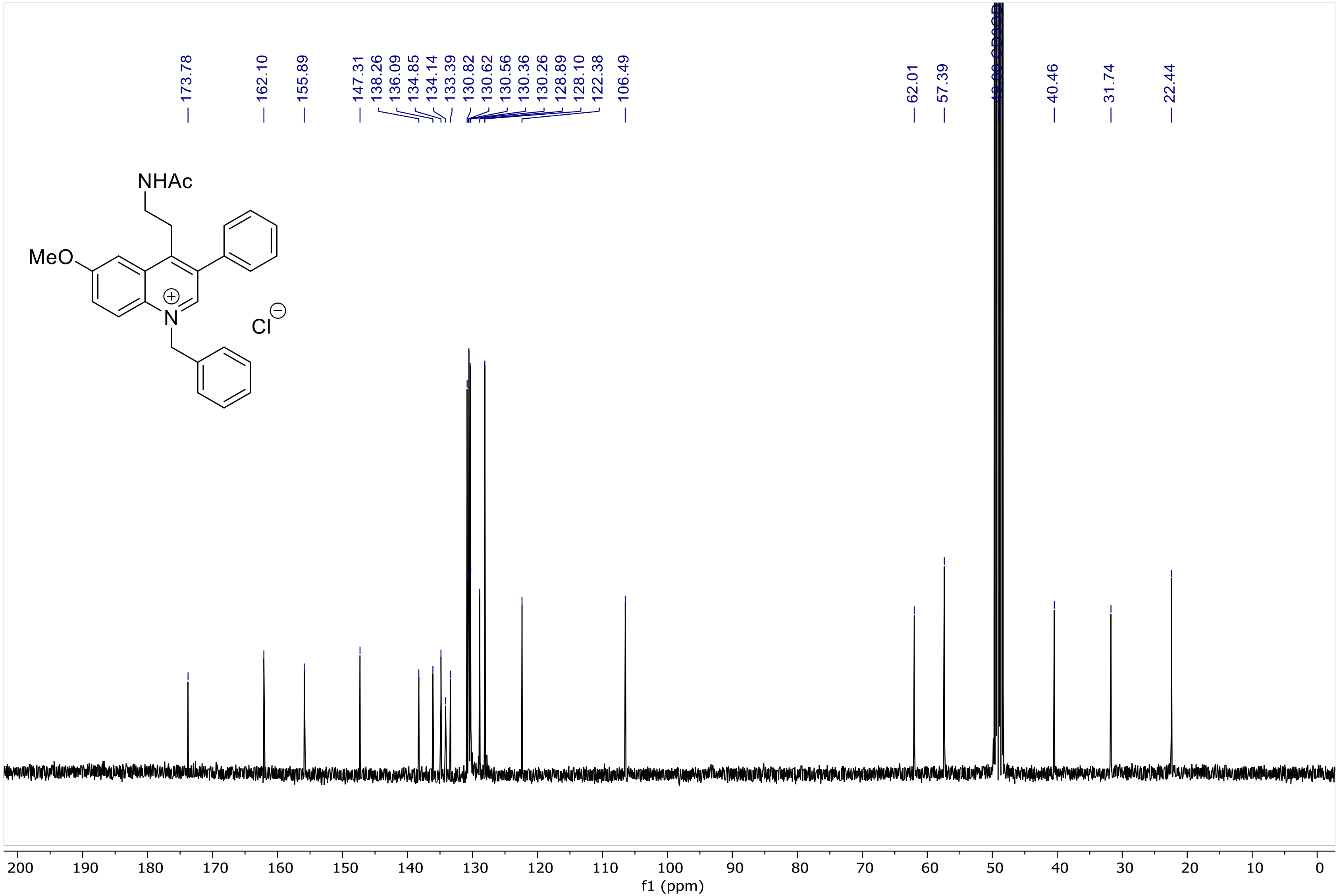

23: (*S*)-4-(2-acetamido-3-methoxy-3-oxopropyl)-1-benzyl-3-(4-fluorophenyl)quinolinium chloride – ^{1}H NMR (400 MHz, CD_3OD)

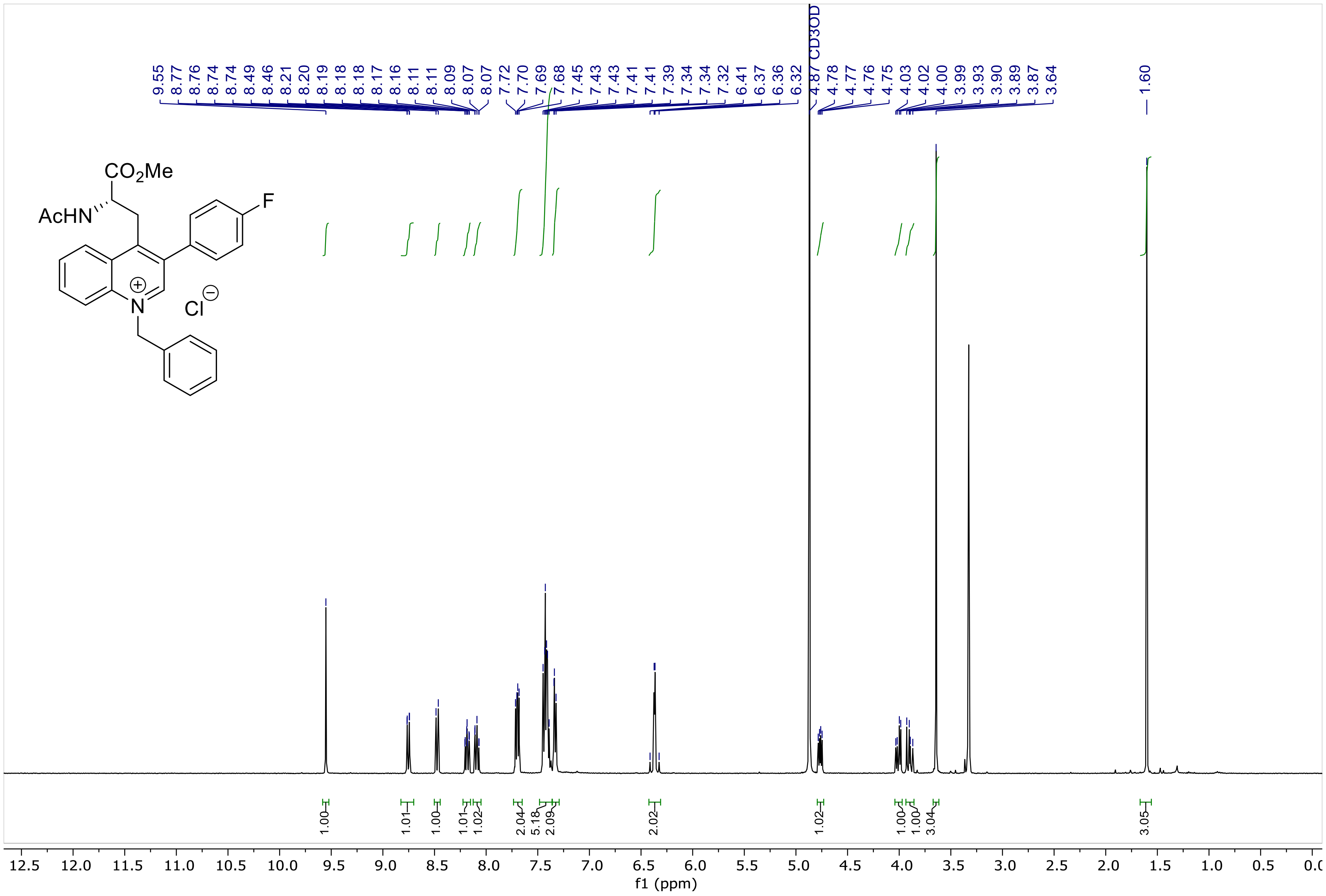

23: (*S*)-4-(2-acetamido-3-methoxy-3-oxopropyl)-1-benzyl-3-(4-fluorophenyl)quinolinium chloride – $^{13}C\{^1H\}$ NMR (101 MHz, CD_3OD)

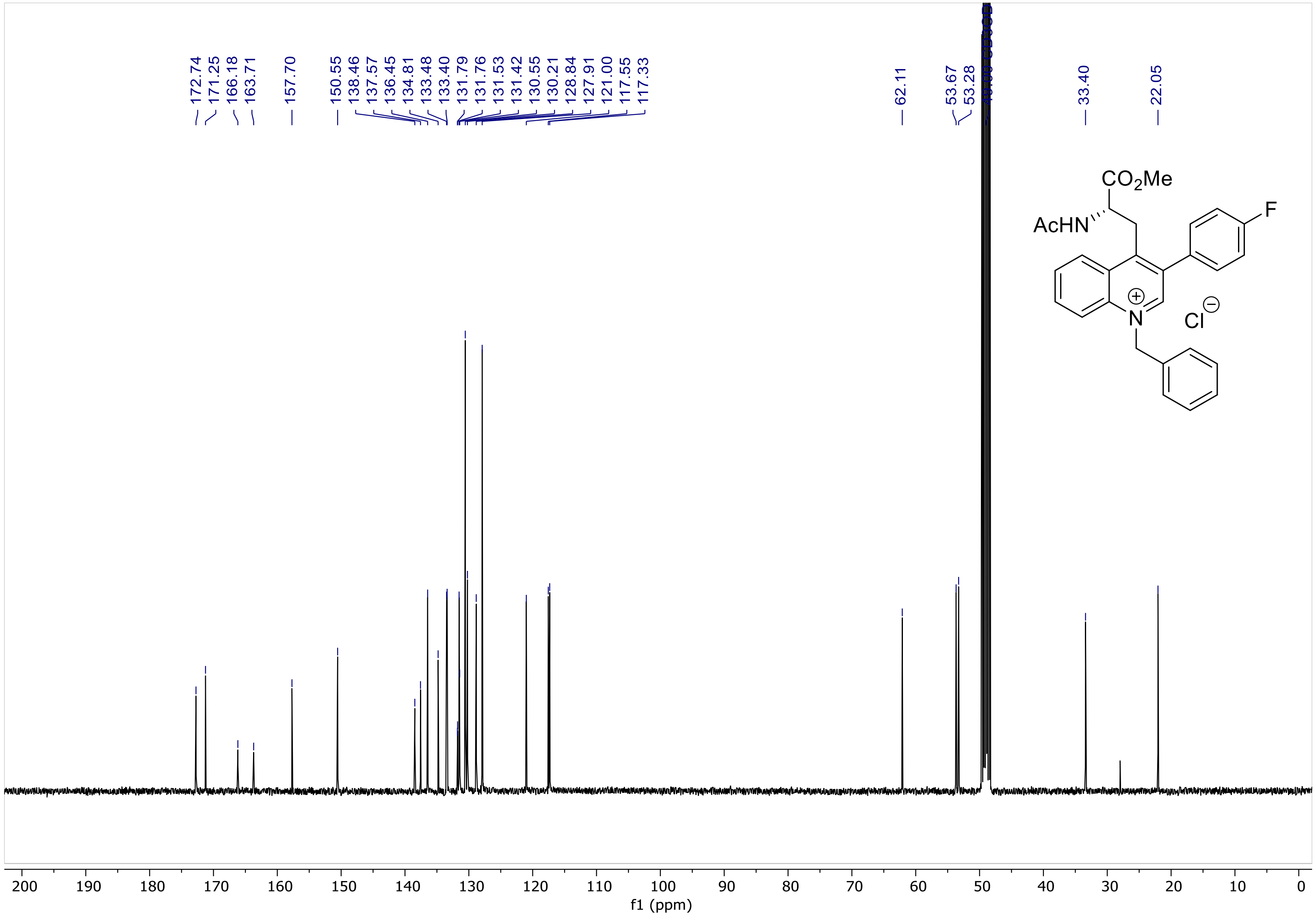

23: (*S*)-4-(2-acetamido-3-methoxy-3-oxopropyl)-1-benzyl-3-(4-fluorophenyl)quinolinium chloride – ^{19}F NMR (376 MHz, CD_3OD)

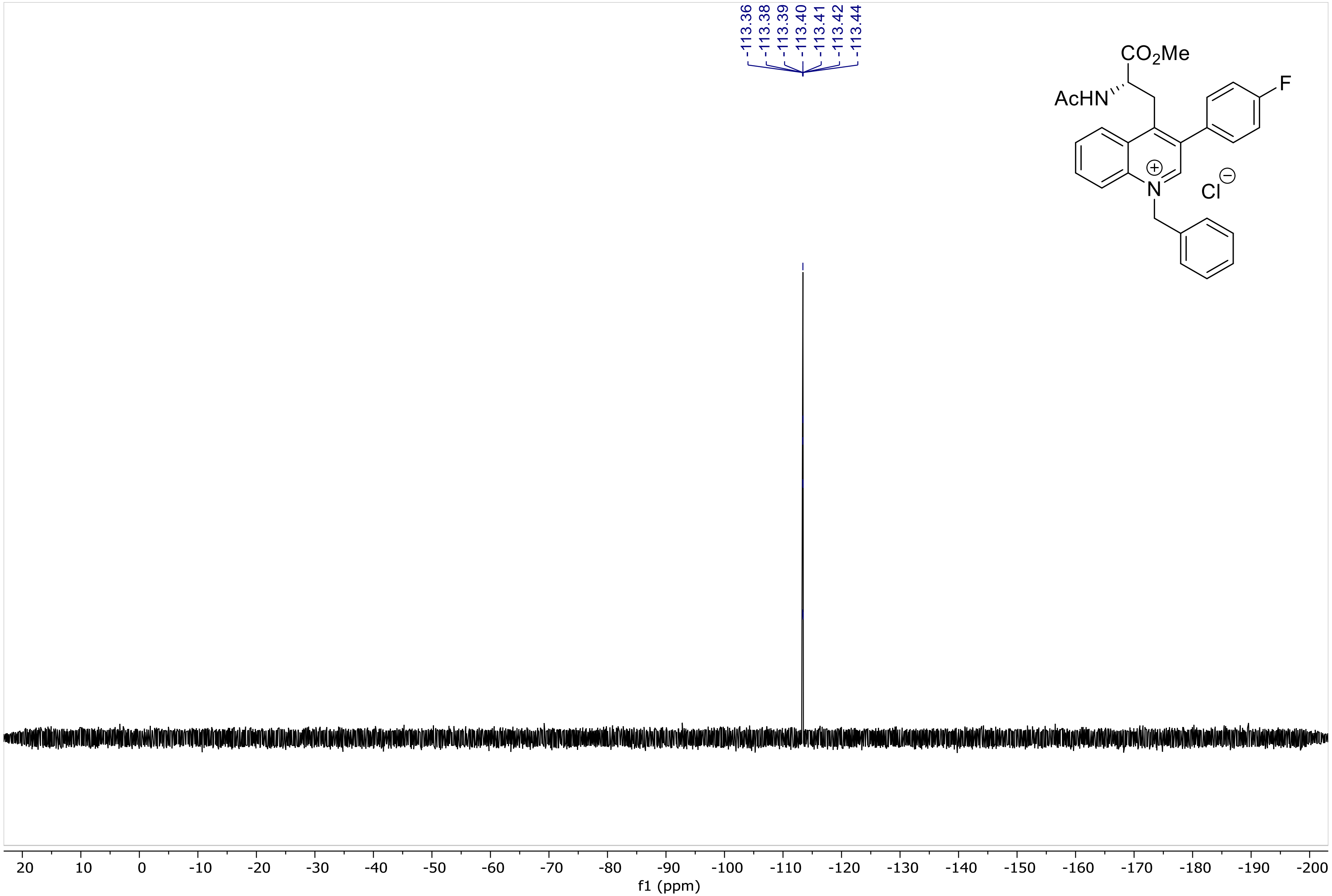

24: (*S*)-4-(2-acetamido-3-((2-methoxy-2-oxoethyl)amino)-3-oxopropyl)-1-benzyl-3-phenylquinolin-1-ium chloride – ^{1}H NMR (400 MHz, CD_3OD)

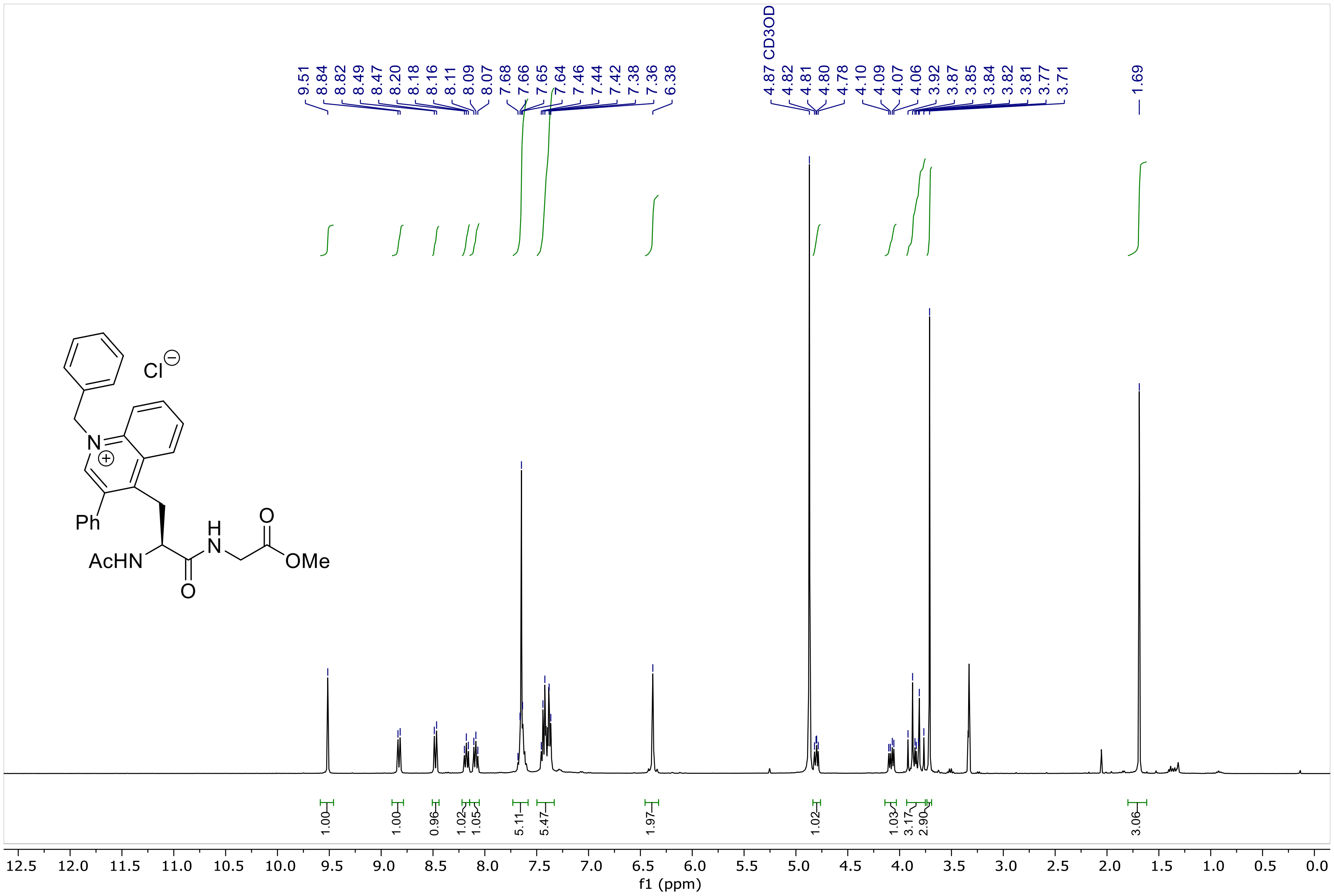

24: (*S*)-4-(2-acetamido-3-((2-methoxy-2-oxoethyl)amino)-3-oxopropyl)-1-benzyl-3-phenylquinolin-1-ium chloride – $^{13}C\{^{1}H\}$ NMR (101 MHz, CD_3OD)

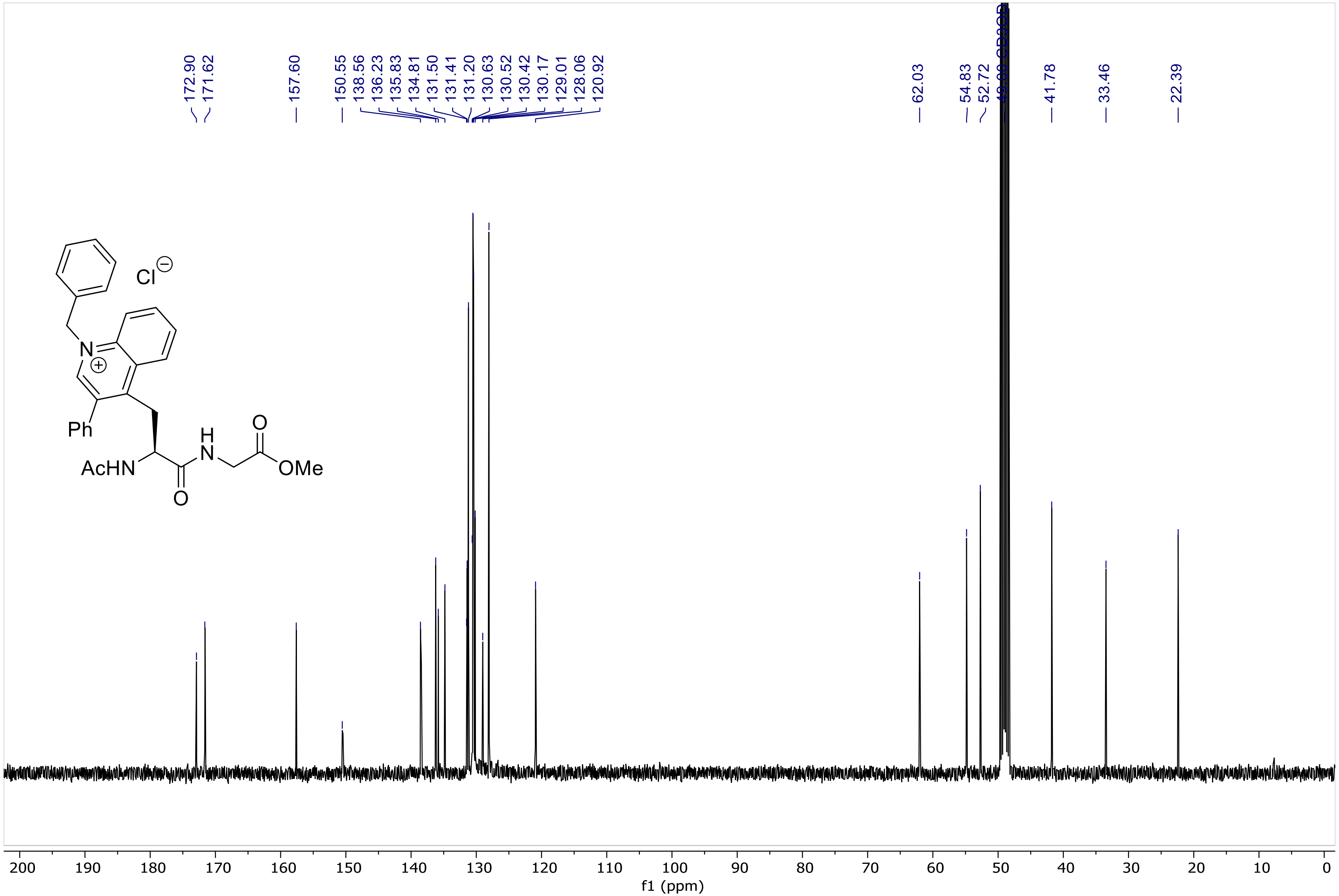

25: 1-benzyl-6-fluoro-3-(4-methylphenyl)quinolinium chloride – ^{1}H NMR (400 MHz, CD_3OD)

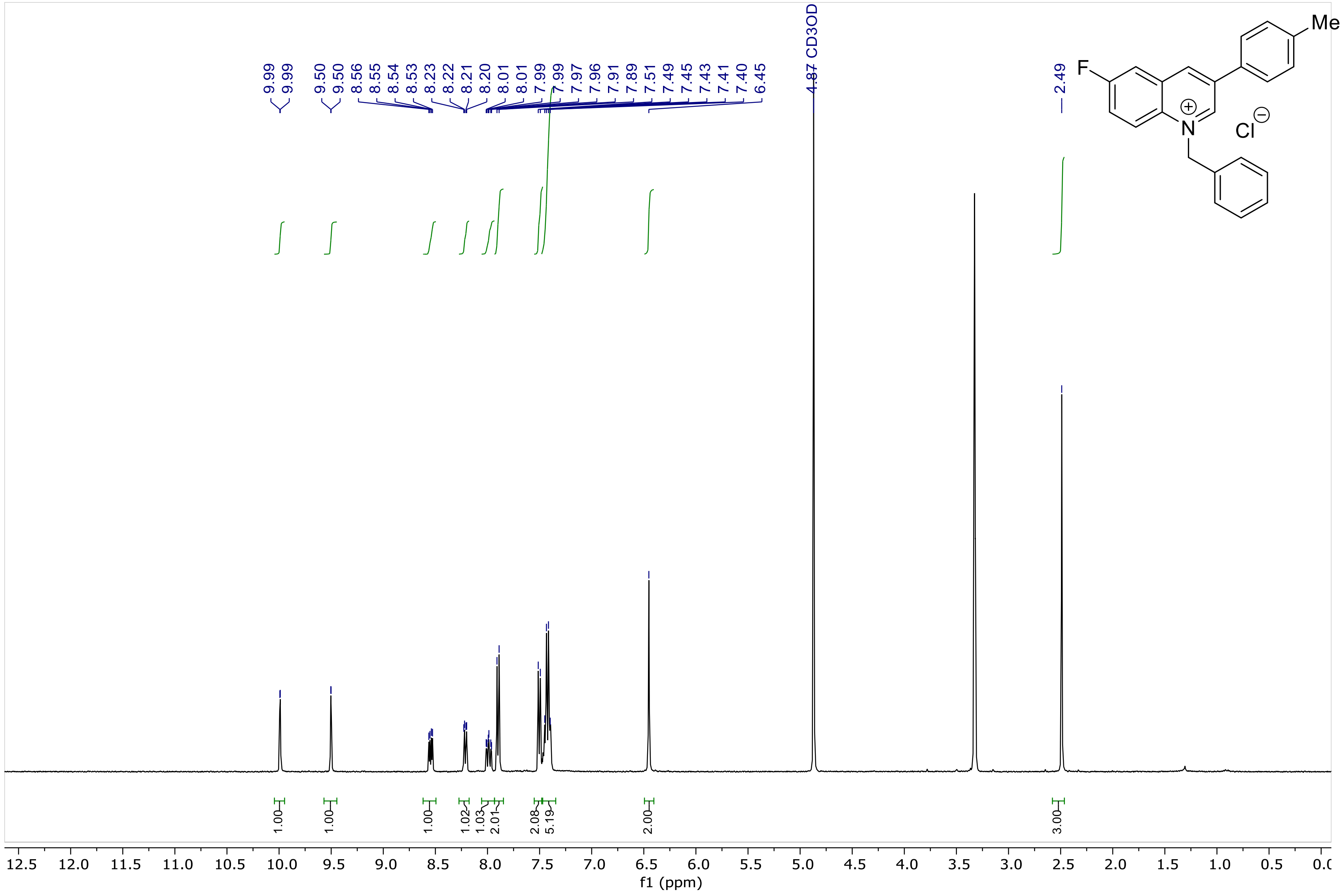

25: 1-benzyl-6-fluoro-3-(4-methylphenyl)quinolinium chloride – $^{13}C\{^{1}H\}$ NMR (101 MHz, CD_3OD)

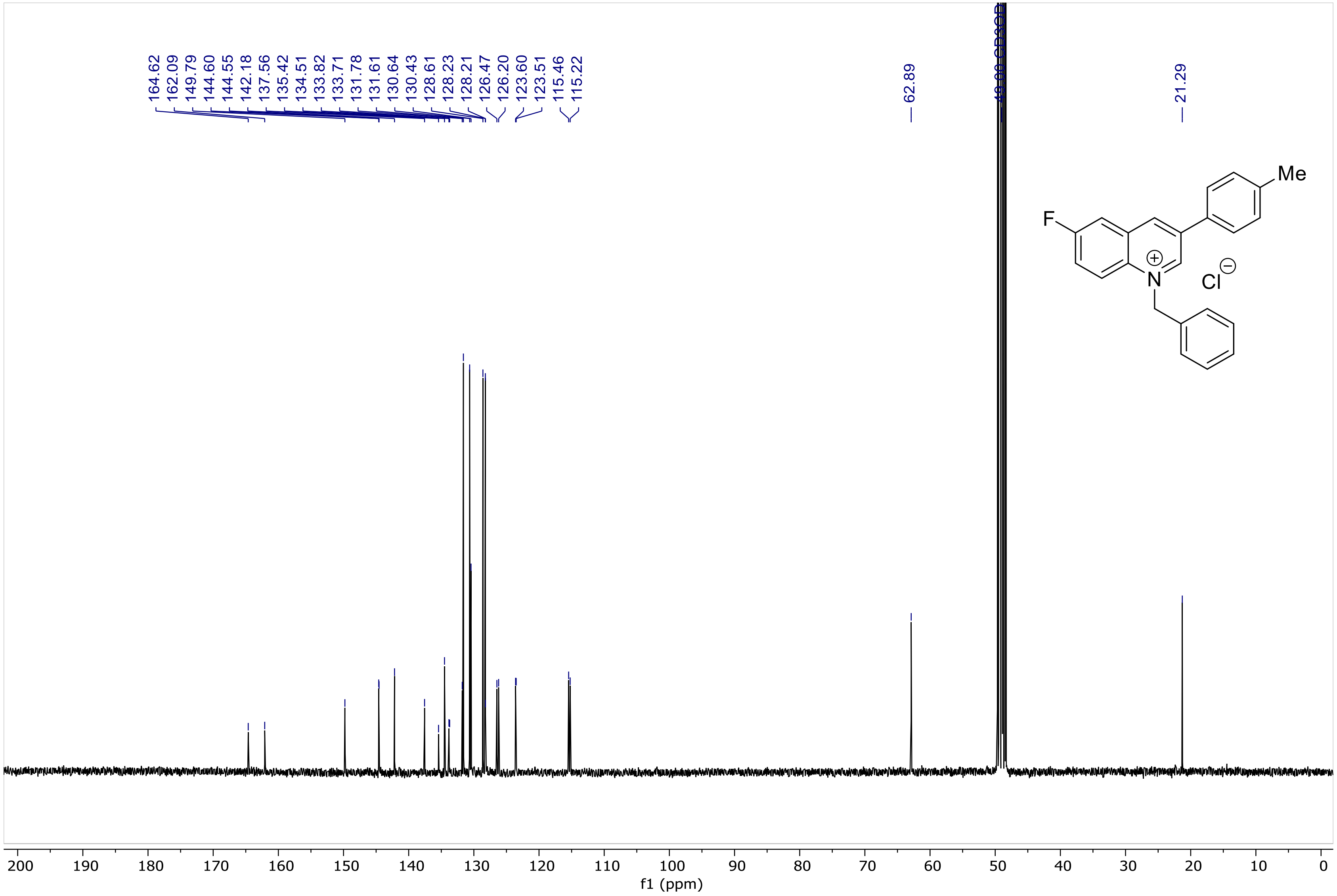

25: 1-benzyl-6-fluoro-3-(4-methylphenyl)quinolinium chloride – ^{19}F NMR (376 MHz, CD_3OD)

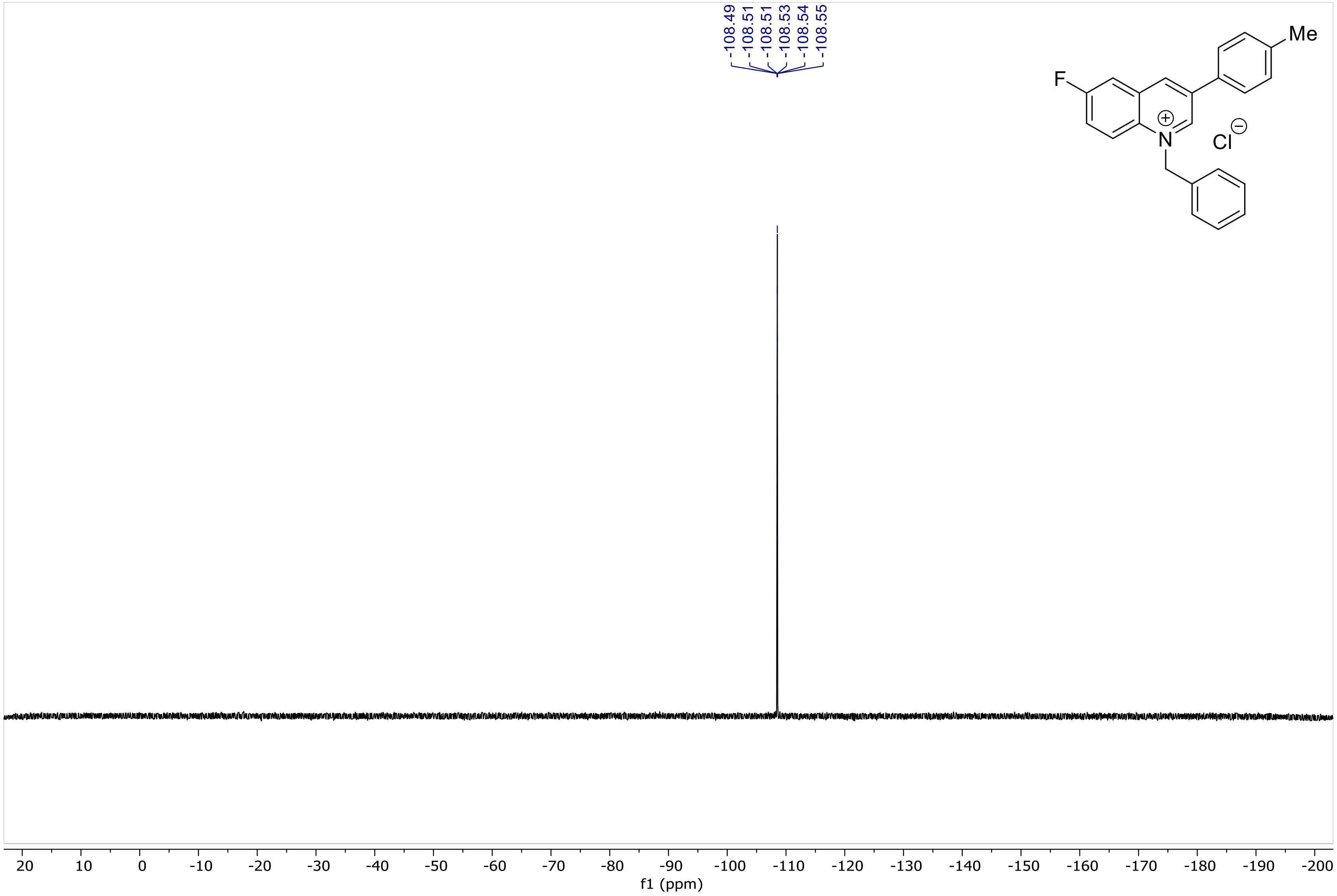

26: 1-benzyl-6-fluoro-3-(4-fluorophenyl)quinolinium chloride – ^{1}H NMR (400 MHz, CD_3OD)

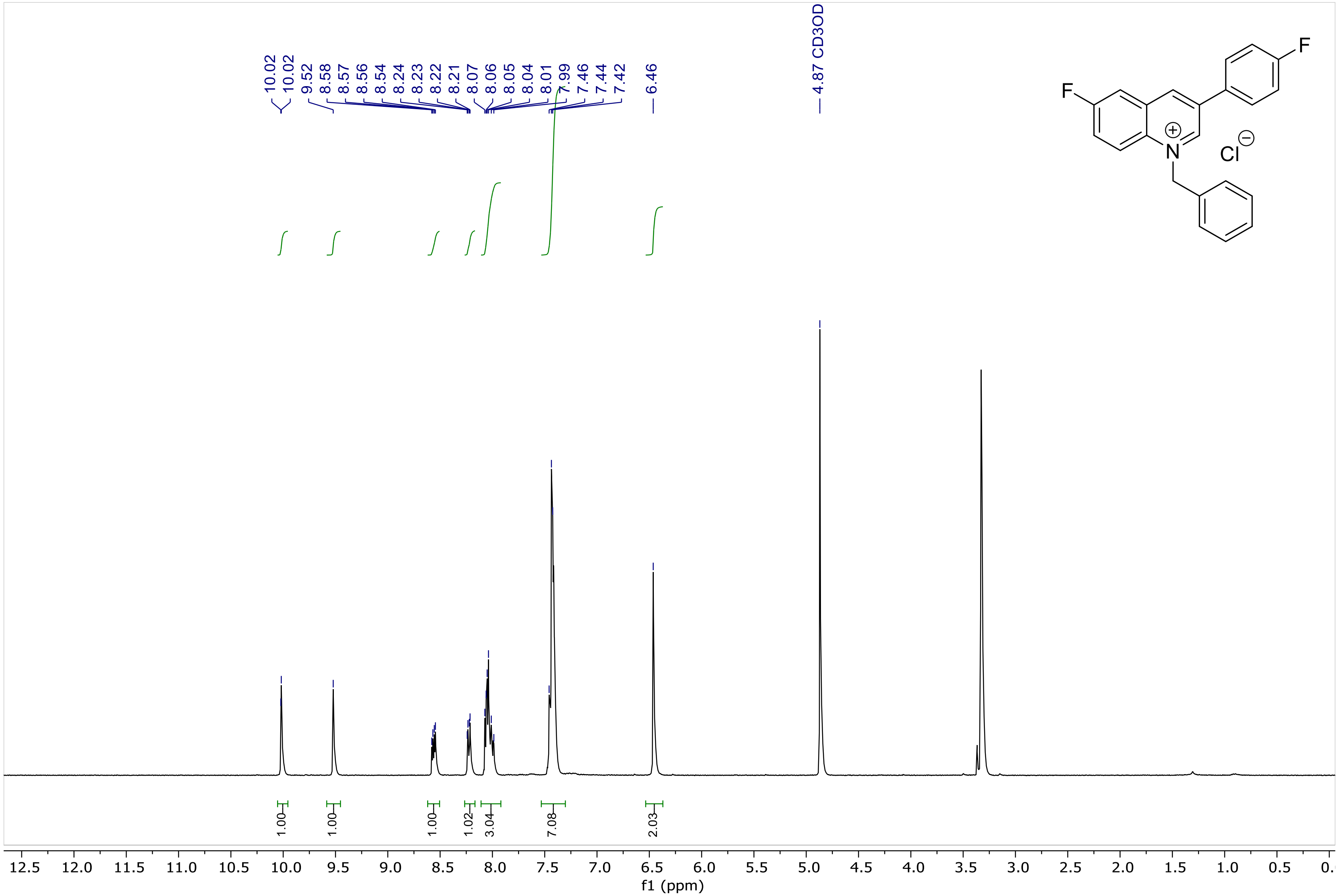

26: 1-benzyl-6-fluoro-3-(4-fluorophenyl)quinolinium chloride – $^{13}C\{^1H\}$ NMR (101 MHz, CD_3OD)

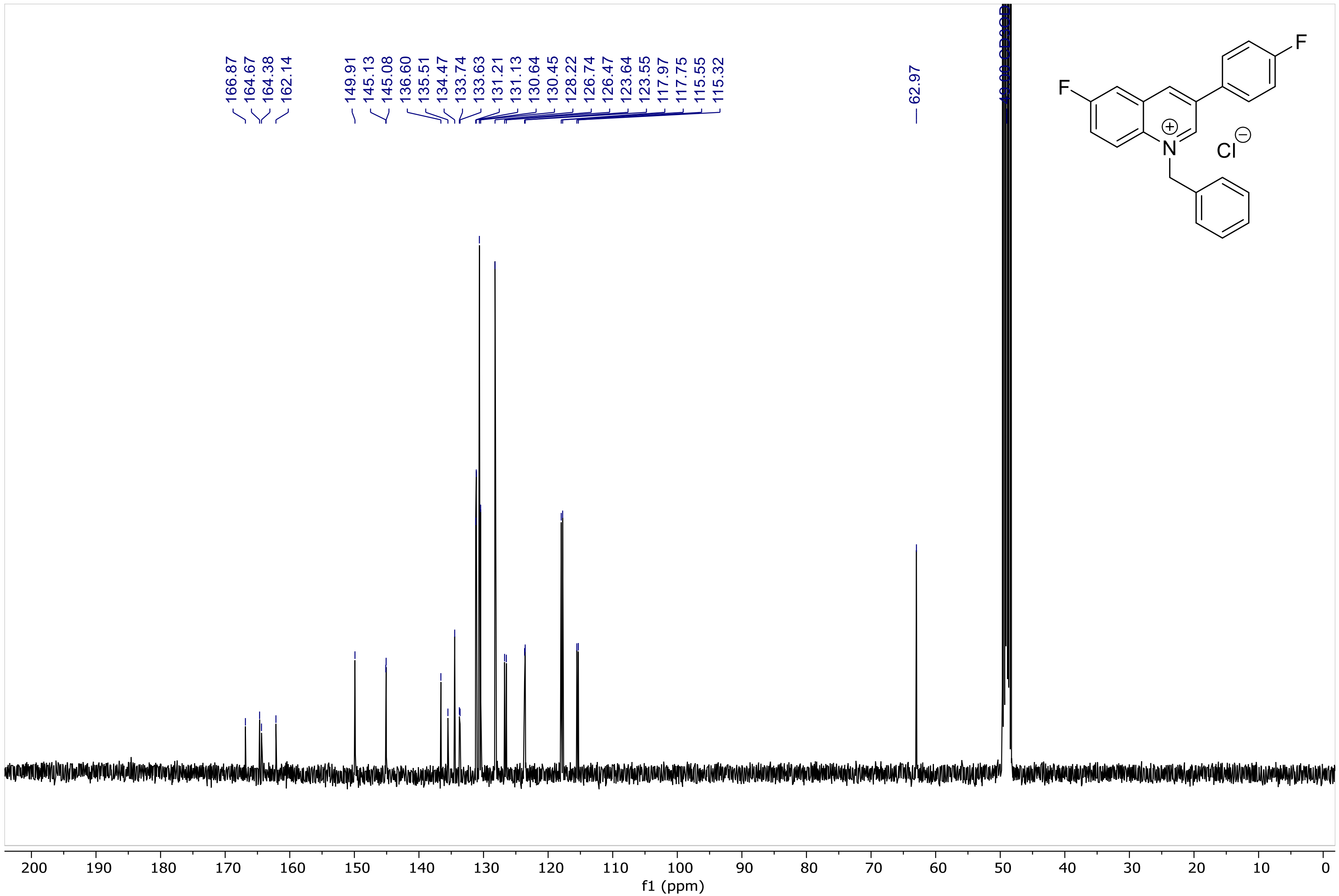

26: 1-benzyl-6-fluoro-3-(4-fluorophenyl)quinolinium chloride – ^{19}F NMR (376 MHz, CD_3OD)

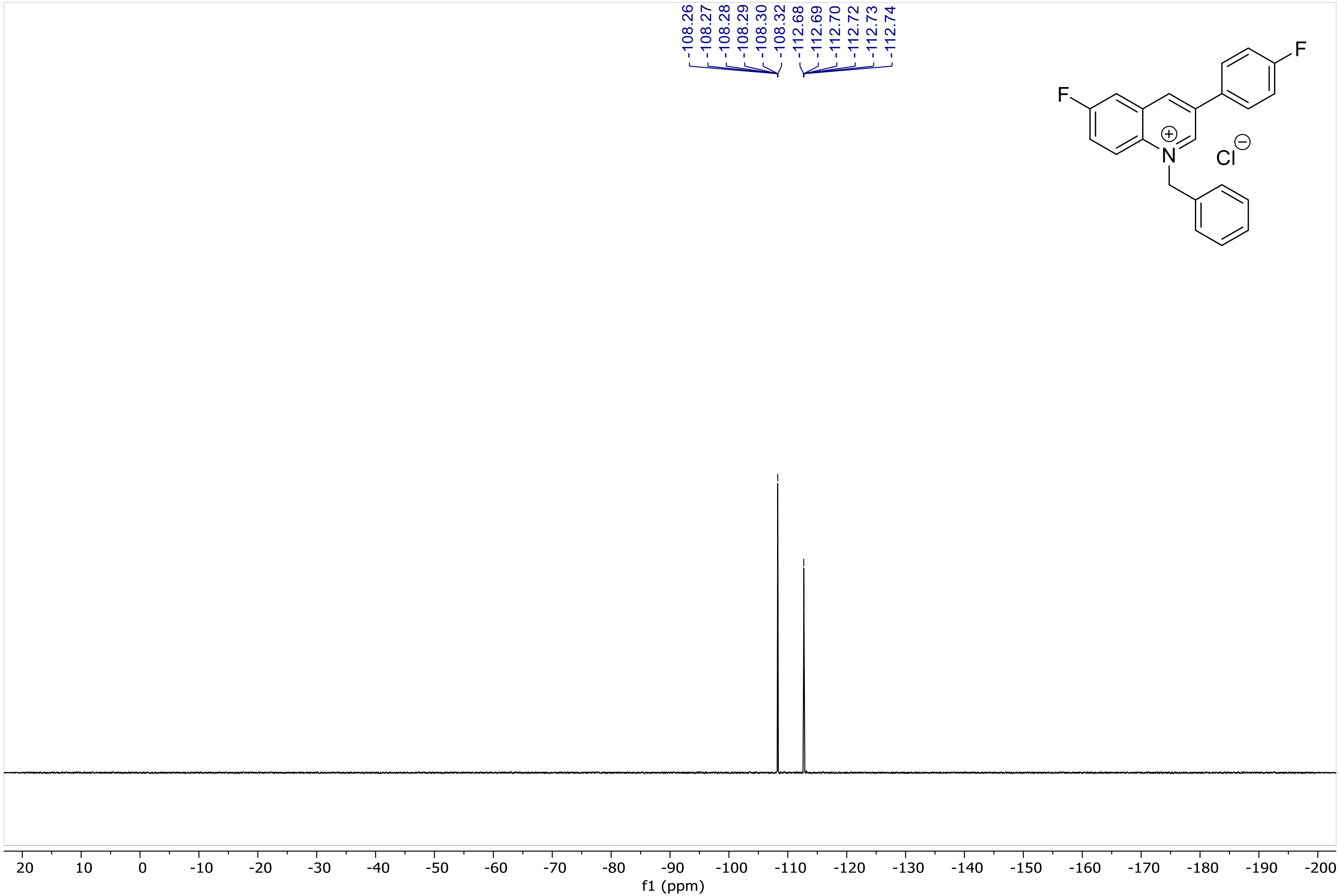

27: 1-benzyl-6-fluoro-3-(4-chlorophenyl)quinolinium chloride – ^{1}H NMR (400 MHz, CD_3OD)

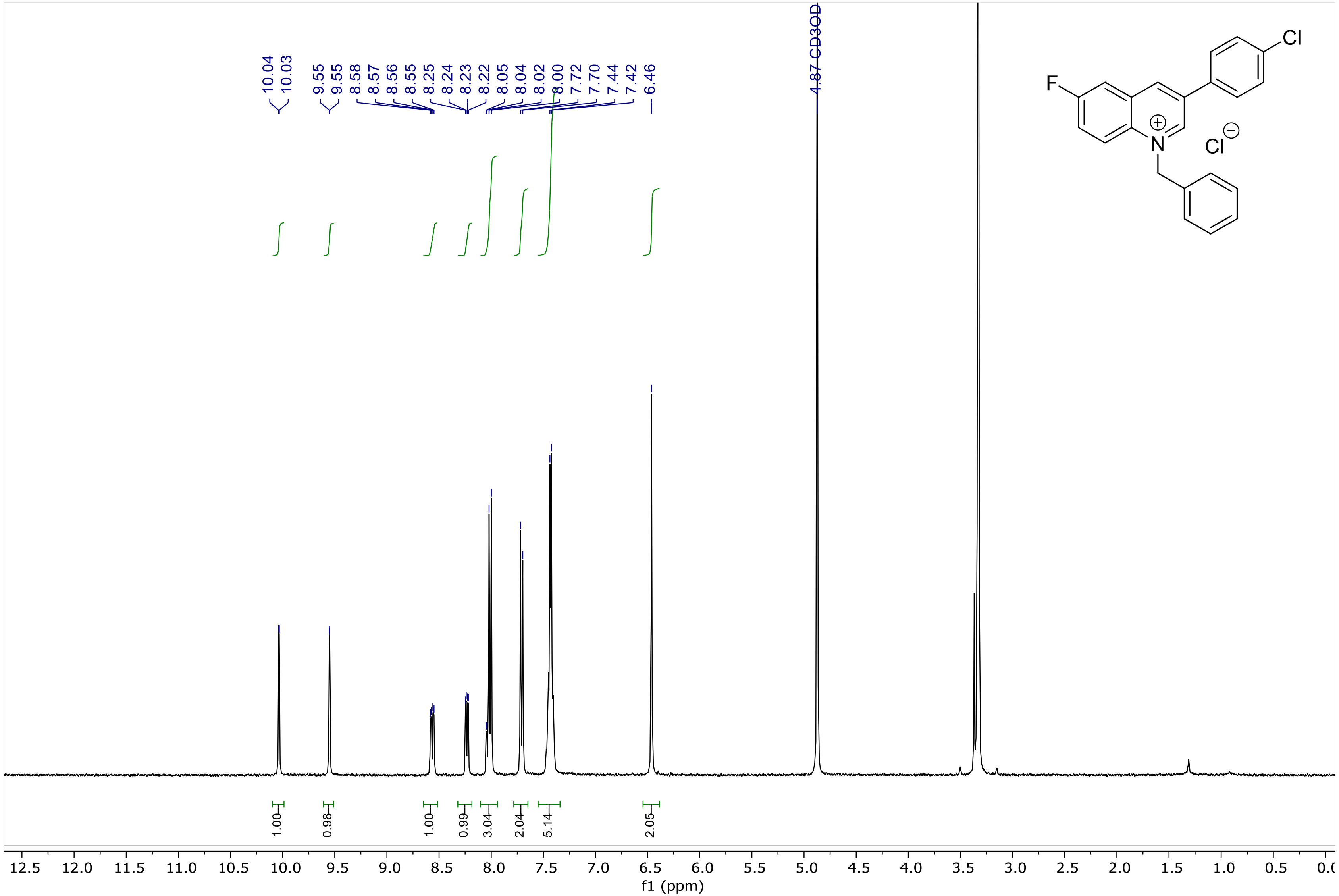

27: 1-benzyl-6-fluoro-3-(4-chlorophenyl)quinolinium chloride – $^{13}C\{^{1}H\}$ NMR (101 MHz, CD_3OD)

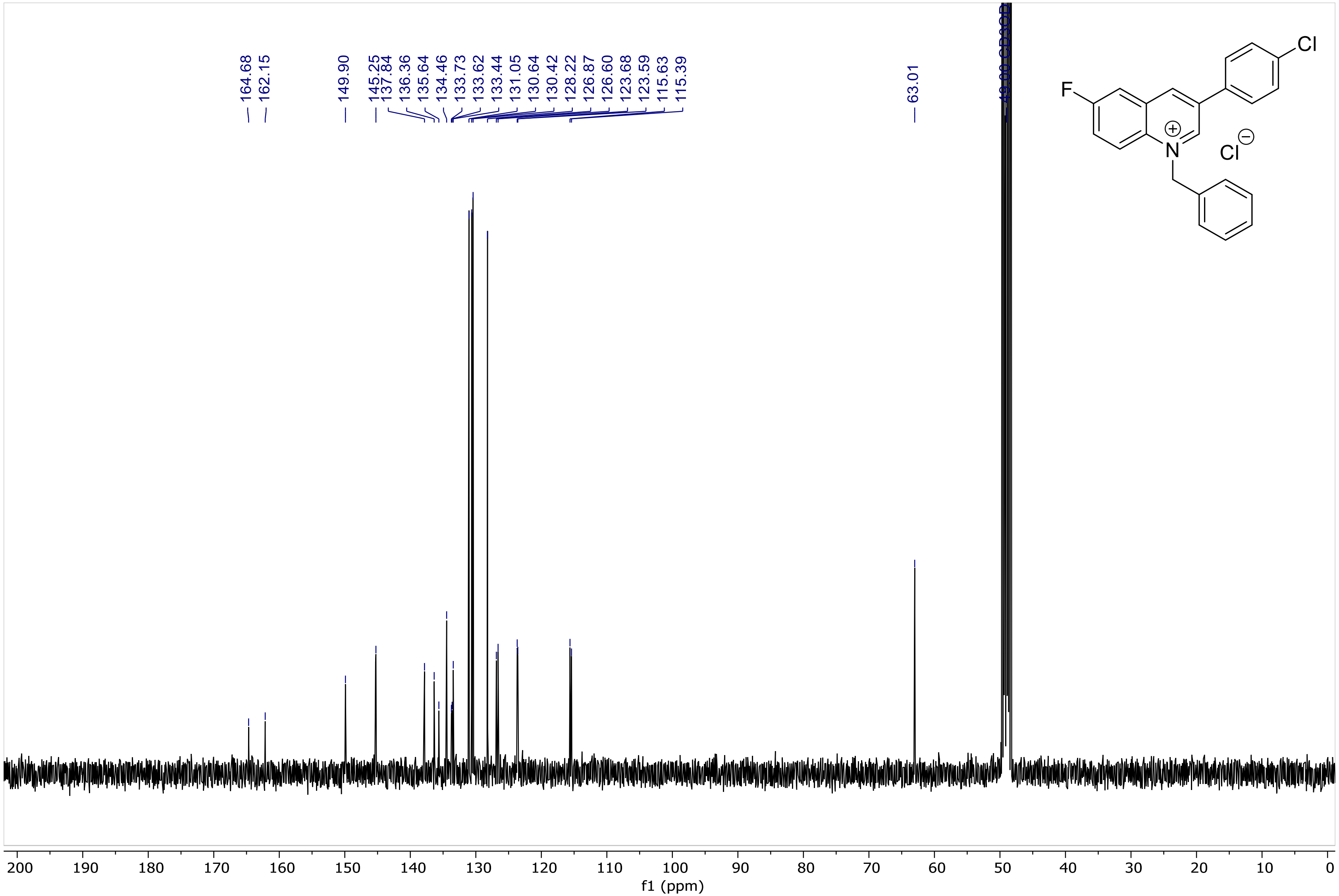

27: 1-benzyl-6-fluoro-3-(4-chlorophenyl)quinolinium chloride – ^{19}F NMR (376 MHz, CD_3OD)

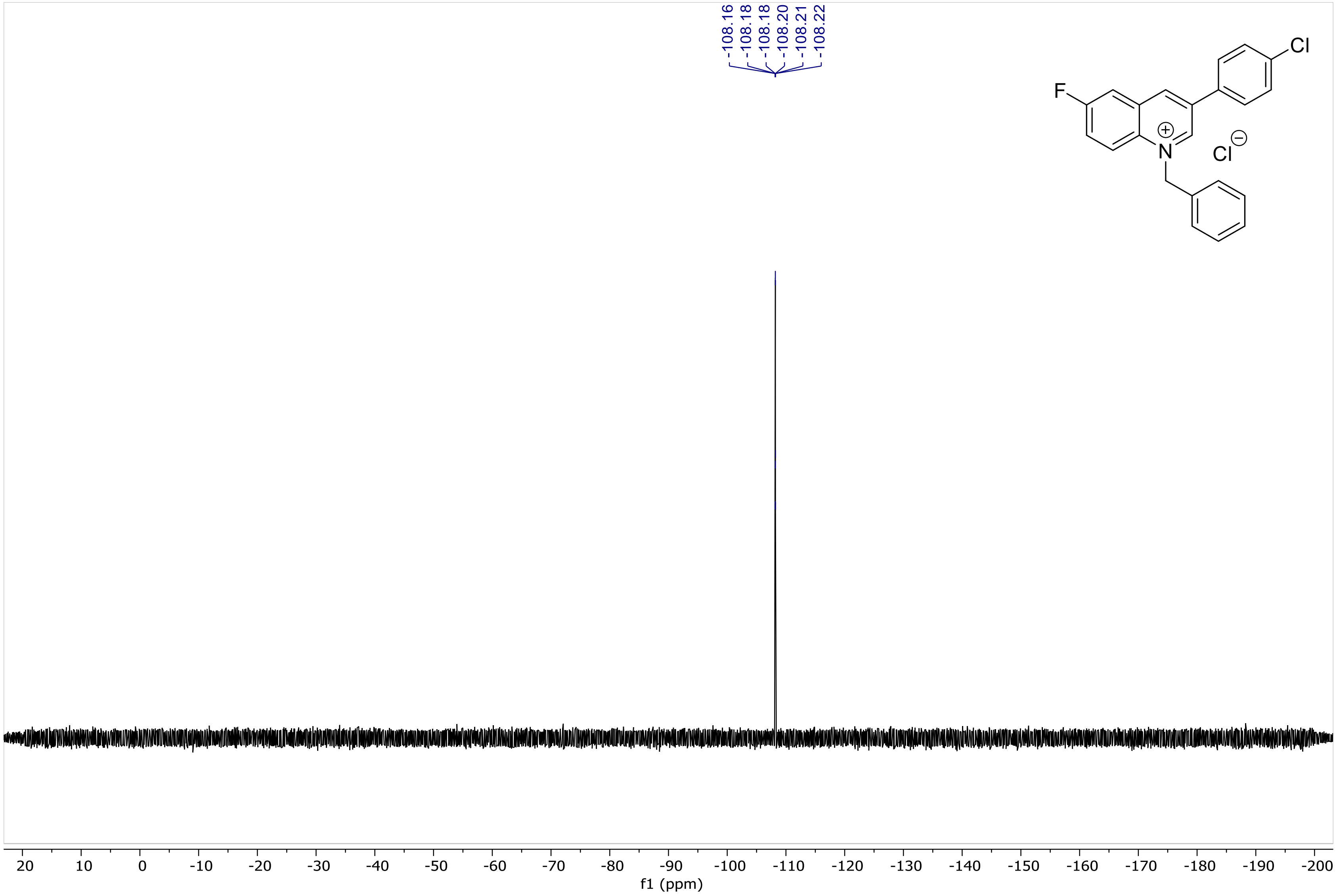

28: 1-benzyl-6-fluoro-3-(4-bromophenyl)quinolinium chloride – ^{1}H NMR (400 MHz, CD_3OD)

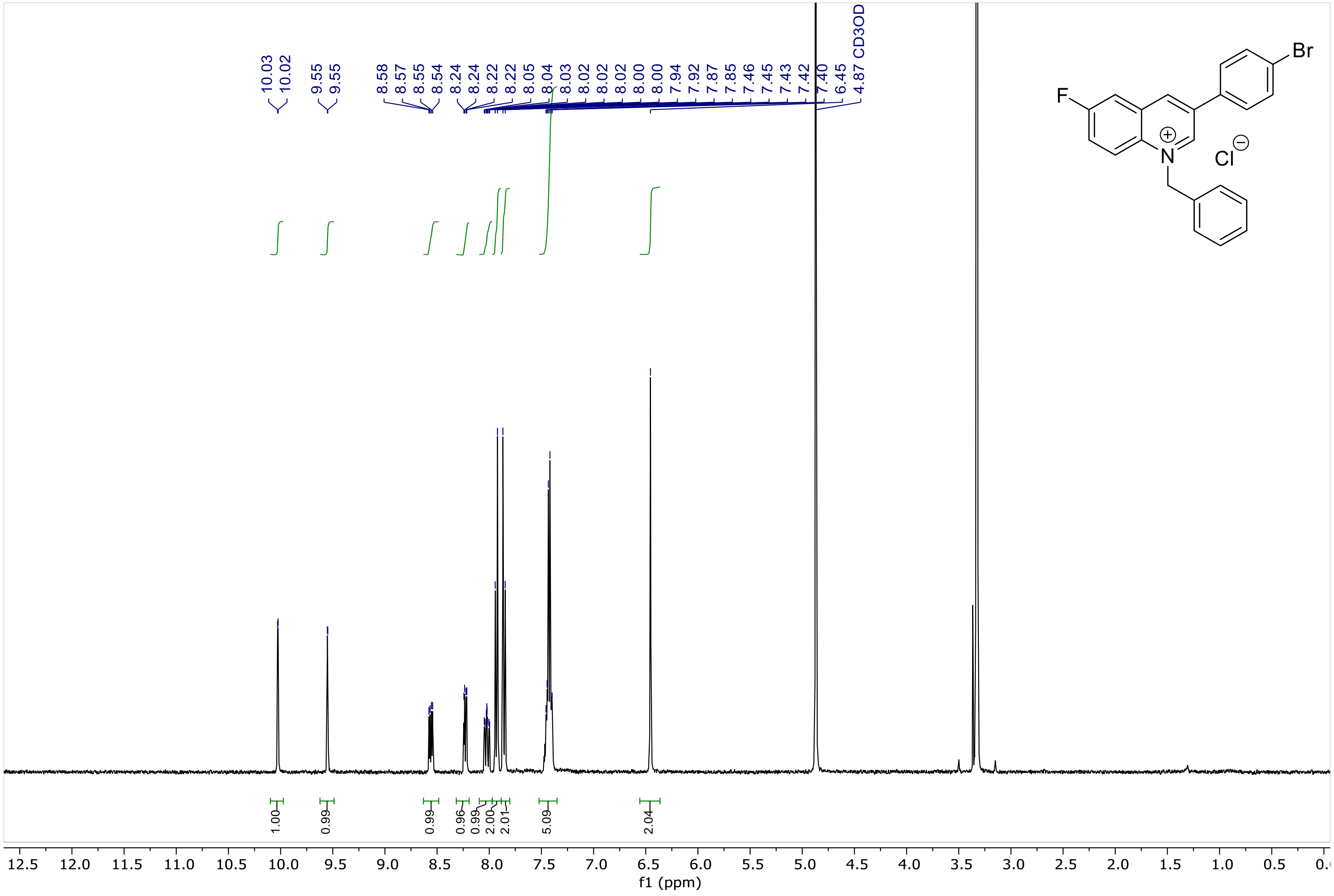

28: 1-benzyl-6-fluoro-3-(4-bromophenyl)quinolinium chloride – $^{13}C\{^{1}H\}$ NMR (101 MHz, CD_3OD)

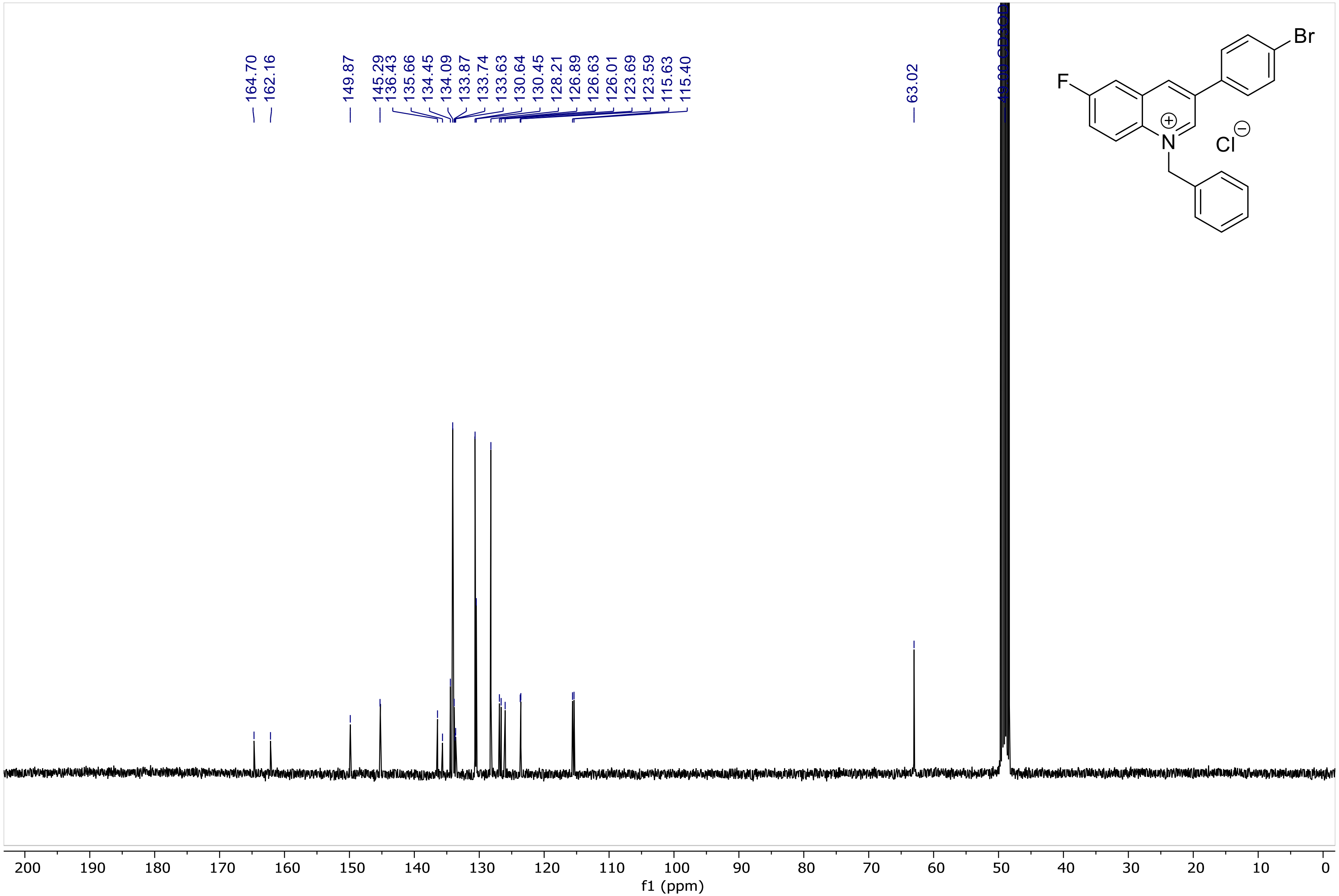

28: 1-benzyl-6-fluoro-3-(4-bromophenyl)quinolinium chloride – ^{19}F NMR (376 MHz, CD_3OD)

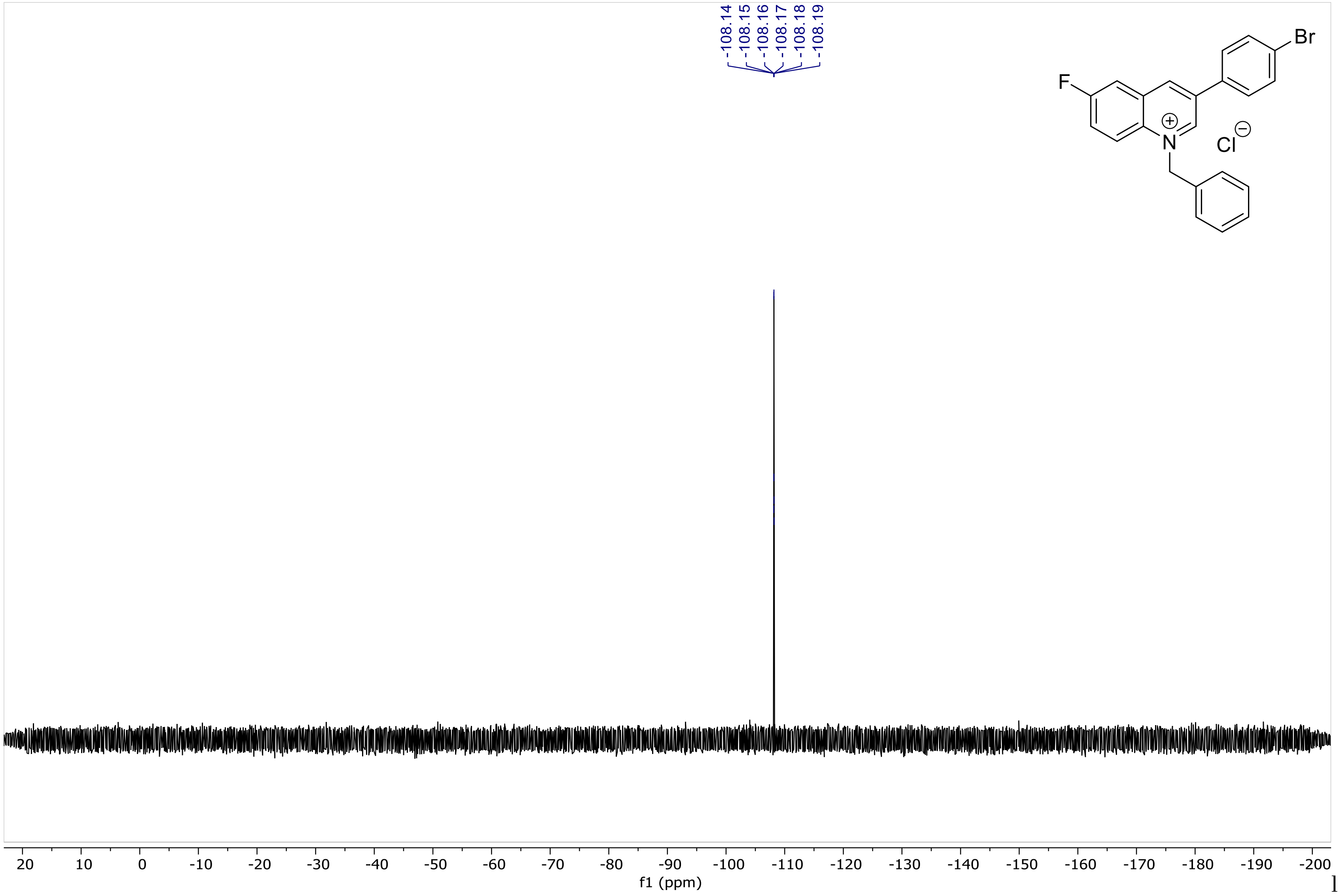

29: 1-Benzyl-6-fluoro-3-(4-nitrophenyl)quinolinium chloride – ^{1}H NMR (400 MHz, CD_3OD)

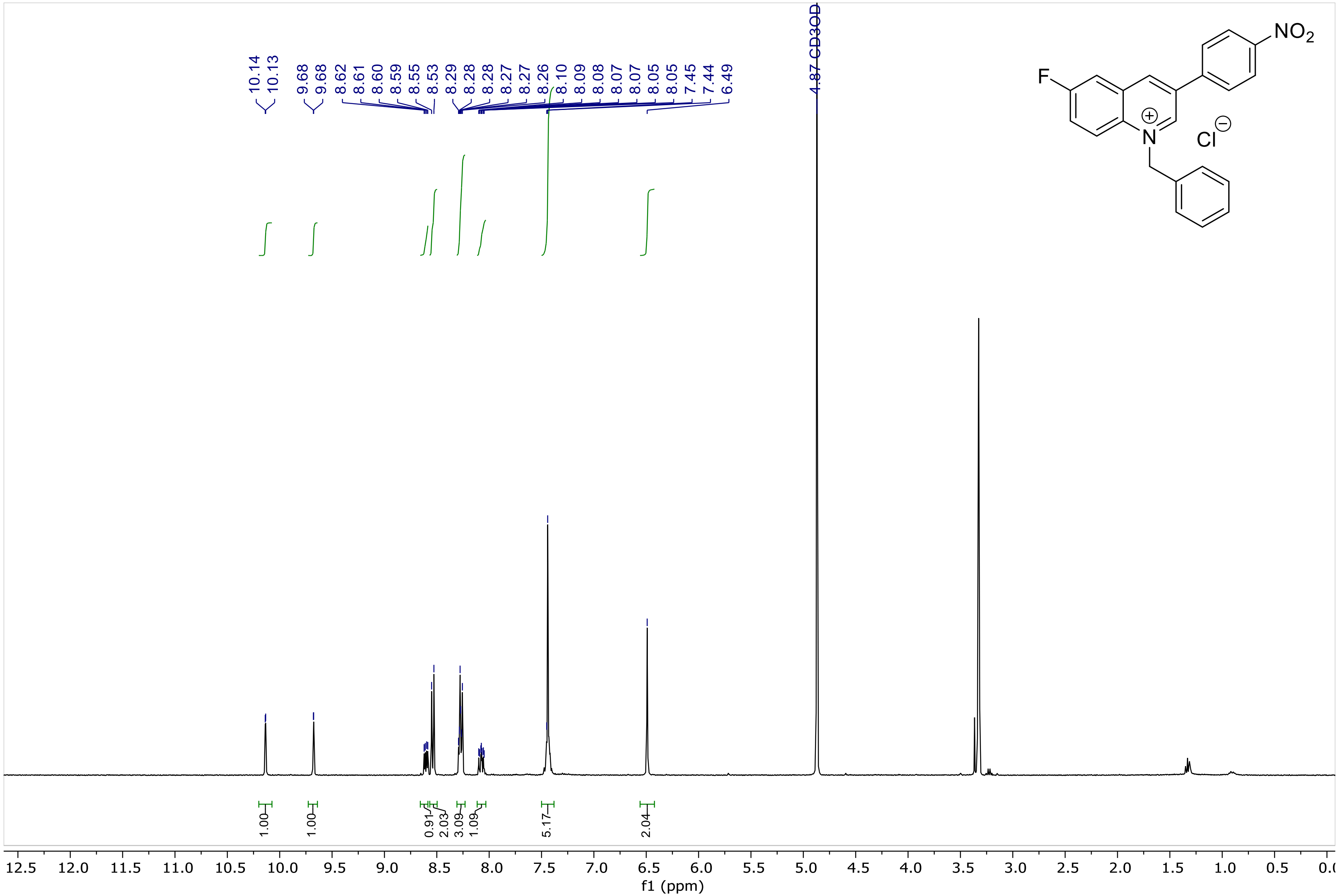

29: 1-Benzyl-6-fluoro-3-(4-nitrophenyl)quinolinium chloride – $^{13}C\{^{1}H\}$ NMR (101 MHz, CD_3OD)

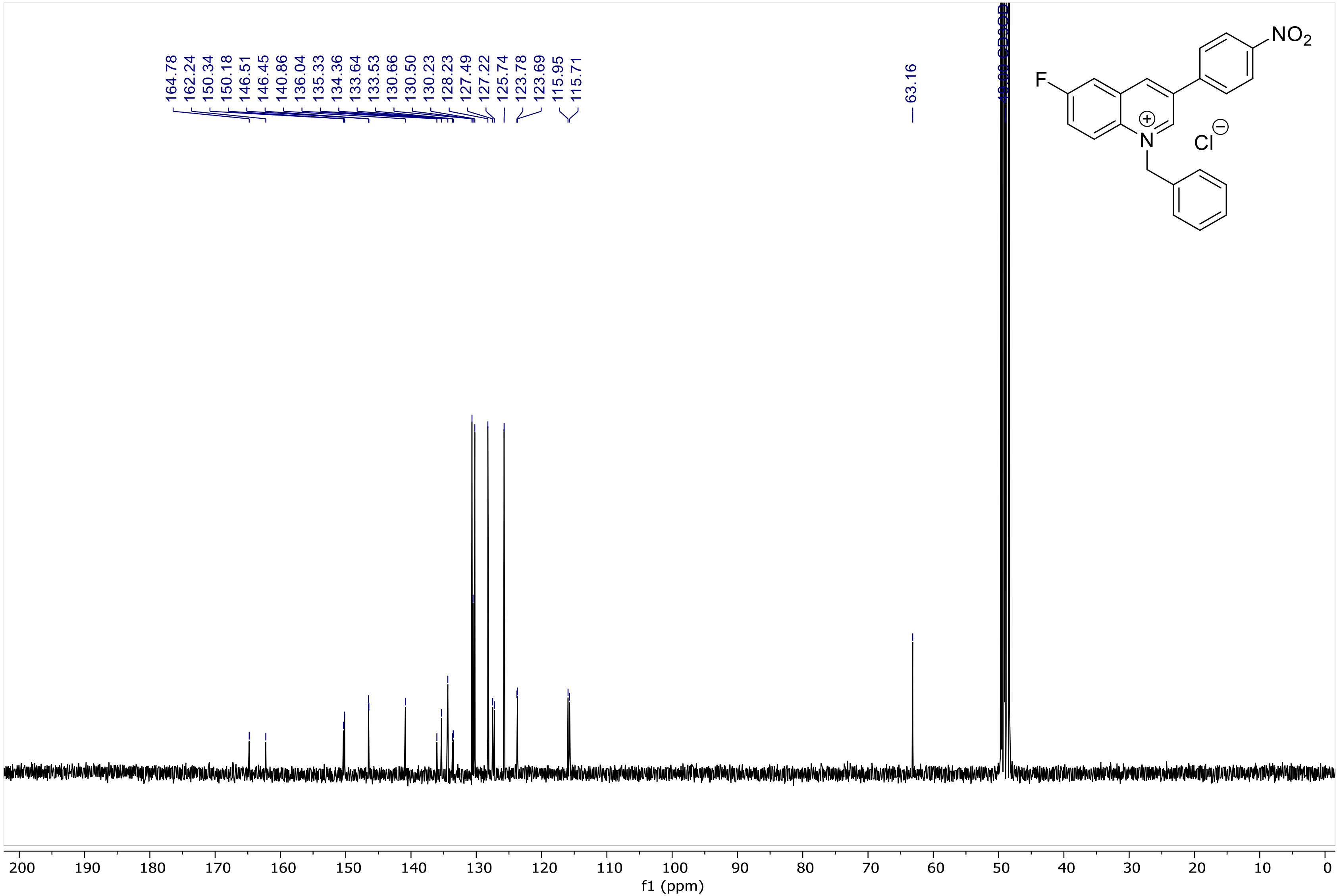

29: 1-Benzyl-6-fluoro-3-(4-nitrophenyl)quinolinium chloride – ^{19}F NMR (376 MHz, CD_3OD)

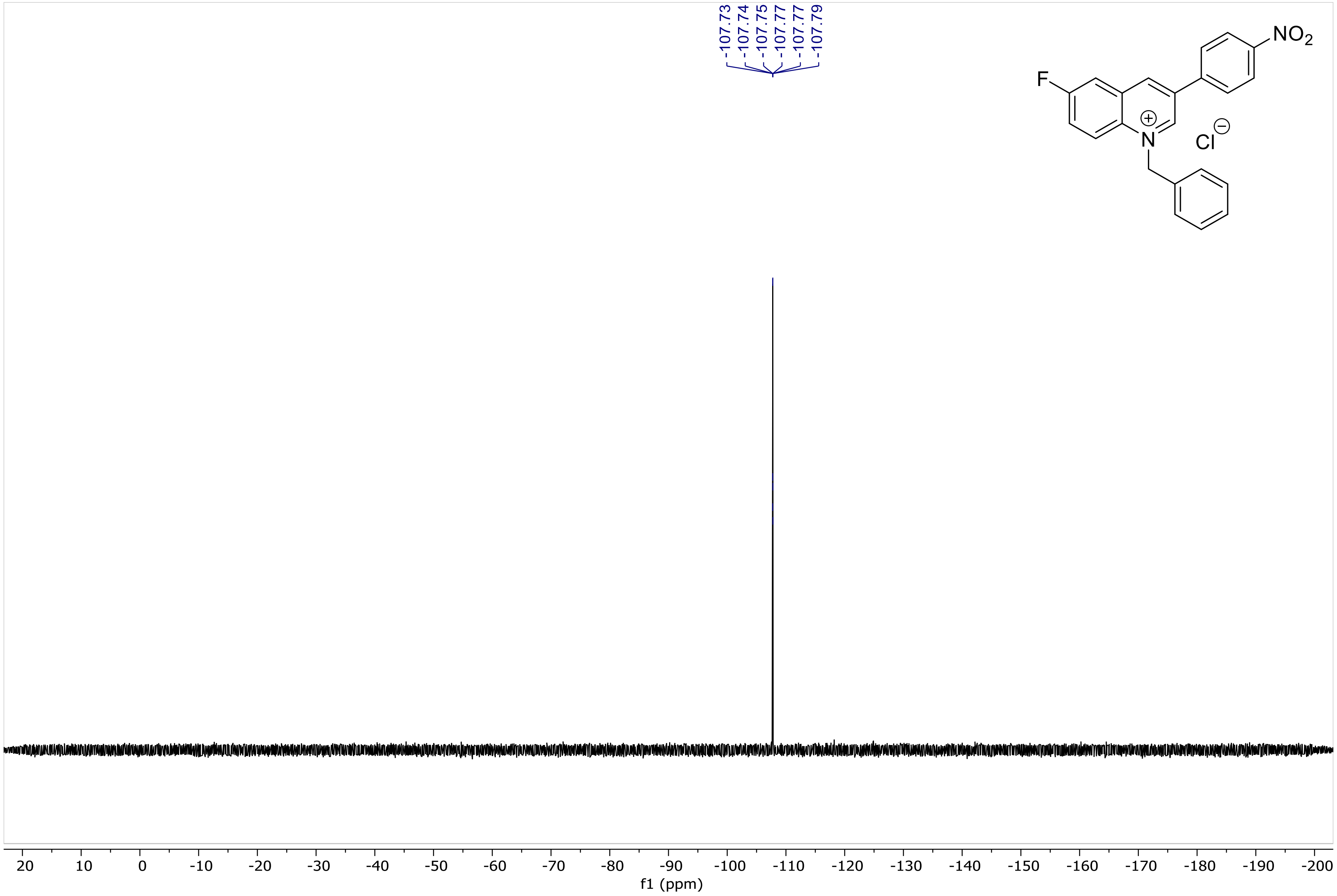

30: 1-Benzyl-6-fluoro-3-(3-bromophenyl)quinolinium chloride – ^{1}H NMR (400 MHz, CD_3OD)

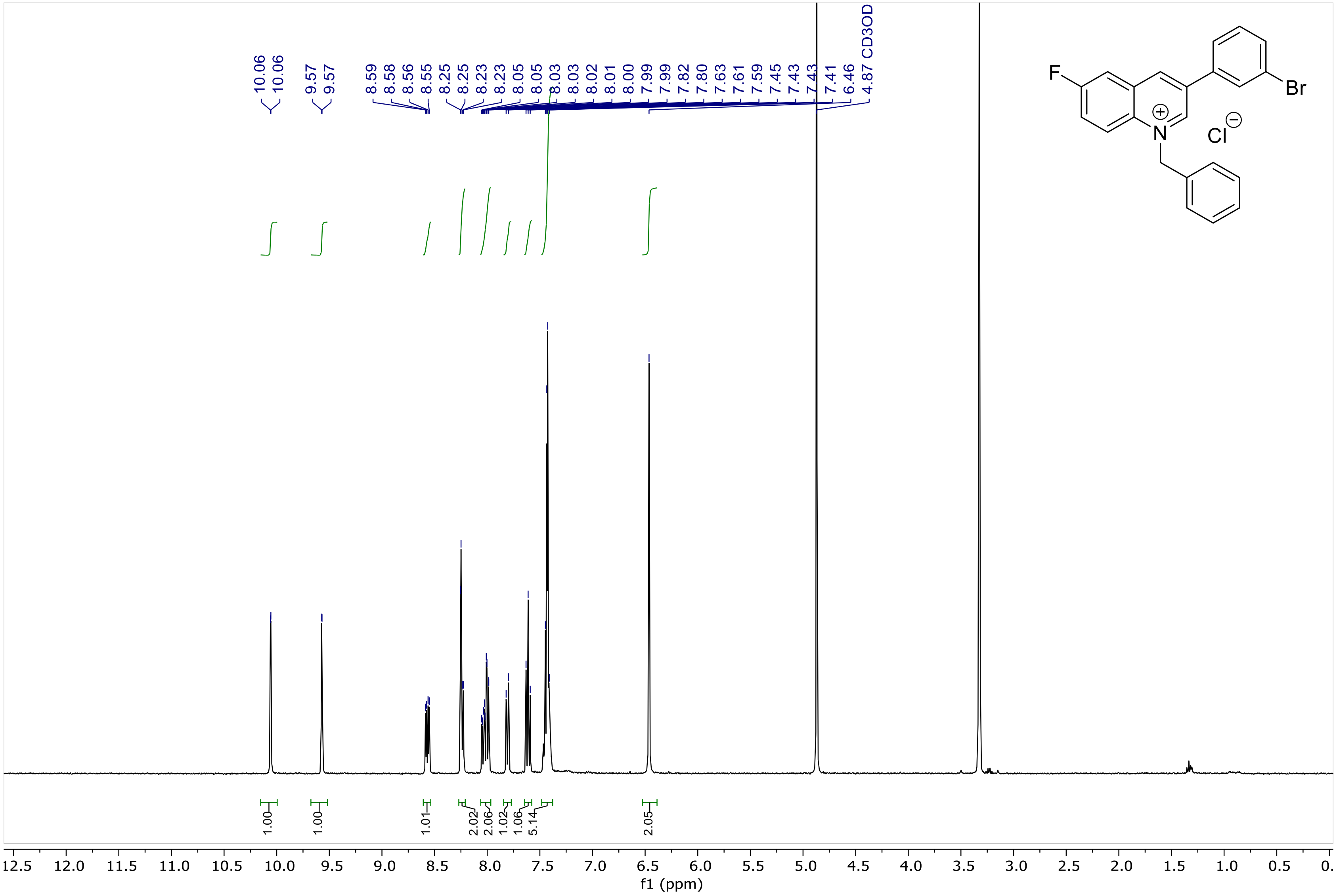

30: 1-Benzyl-6-fluoro-3-(3-bromophenyl)quinolinium chloride – $^{13}C\{^{1}H\}$ NMR (101 MHz, CD_3OD)

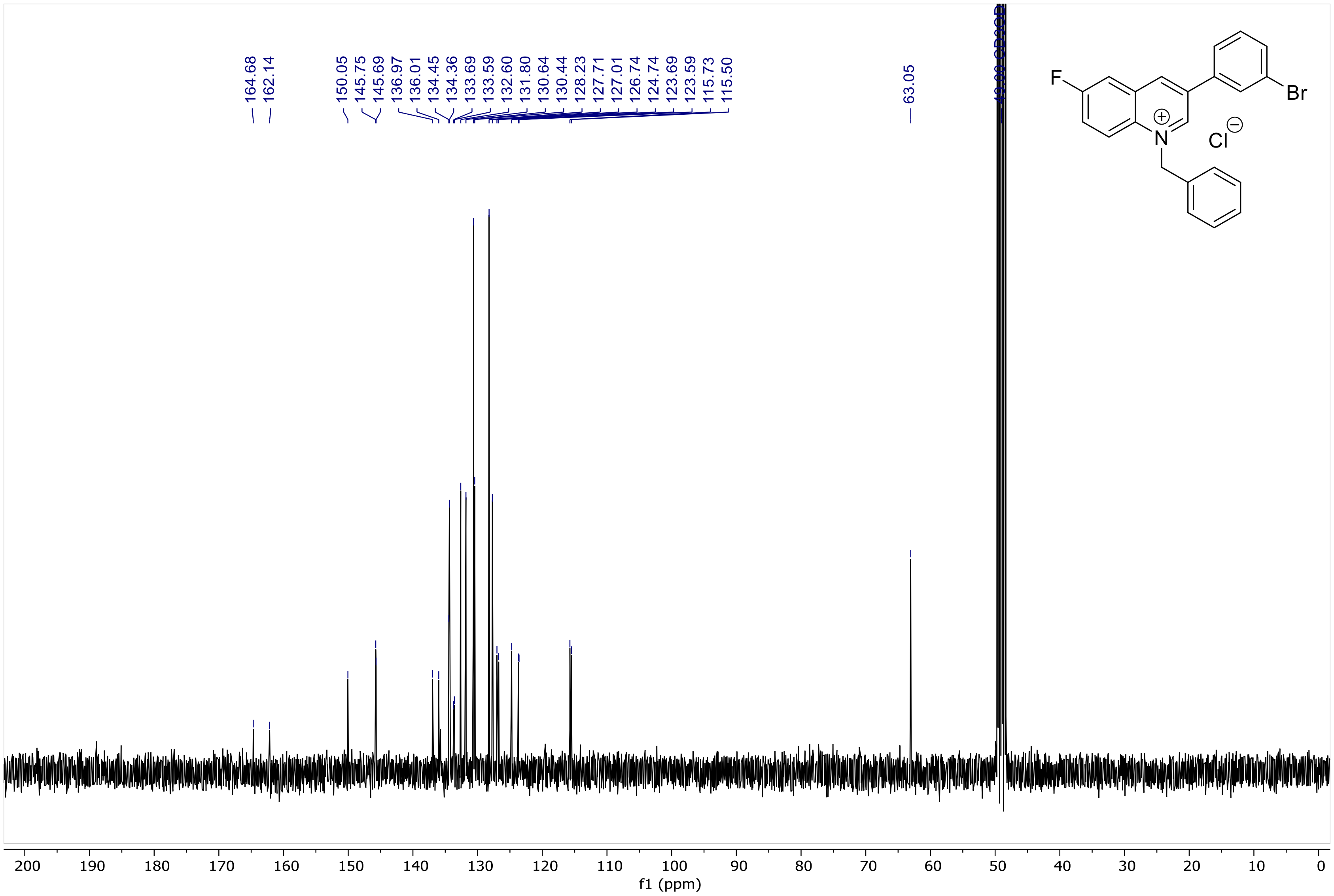

30: 1-Benzyl-6-fluoro-3-(3-bromophenyl)quinolinium chloride – ^{19}F NMR (376 MHz, CD_3OD)

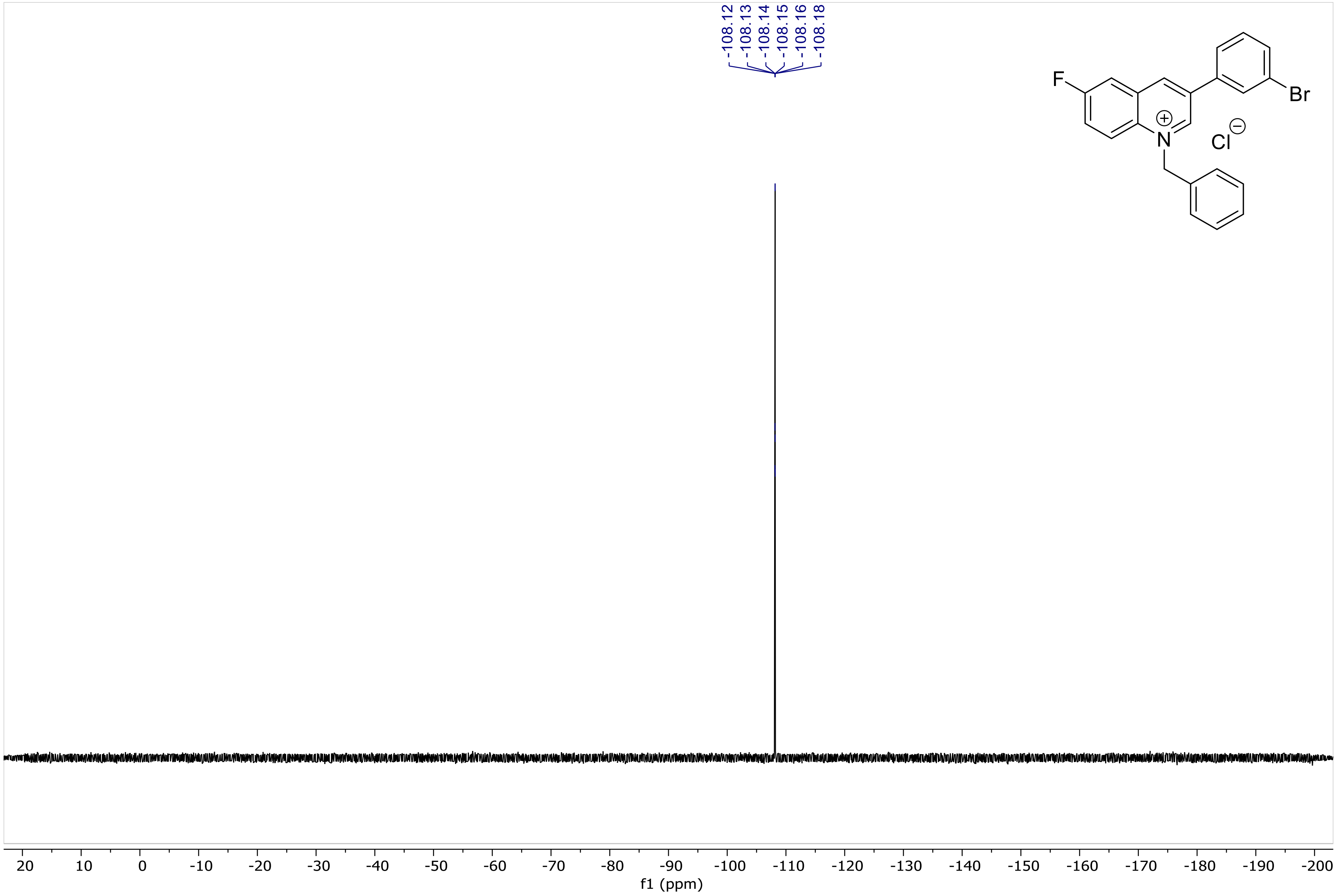

31: 1-Benzyl-6-fluoro-3-(2-trifluoromethoxyphenyl)quinolinium chloride – ^{1}H NMR (400 MHz, CD_3OD)

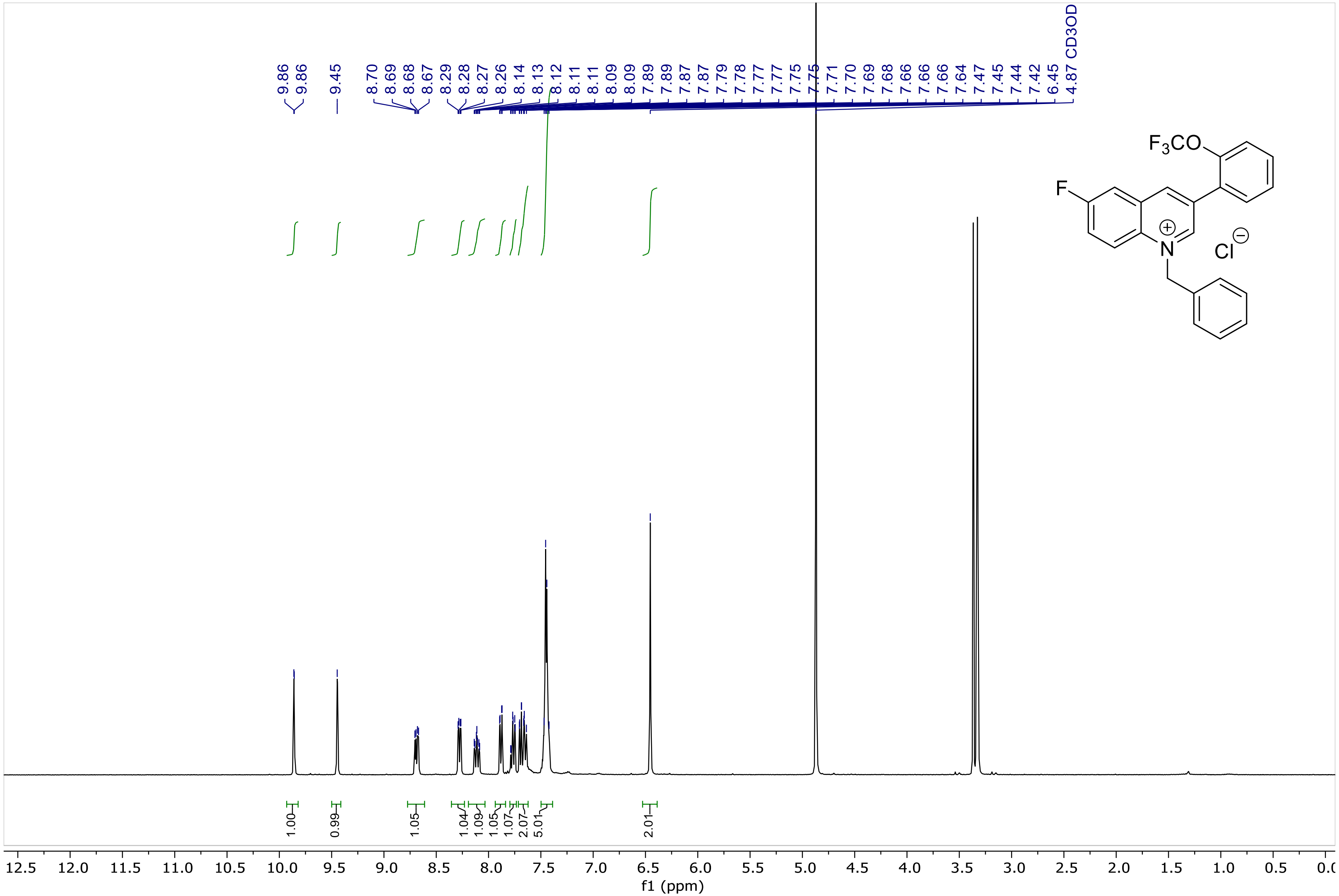

31: 1-Benzyl-6-fluoro-3-(2-trifluoromethoxyphenyl)quinolinium chloride – $^{13}C\{^1H\}$ NMR (101 MHz, CD_3OD)

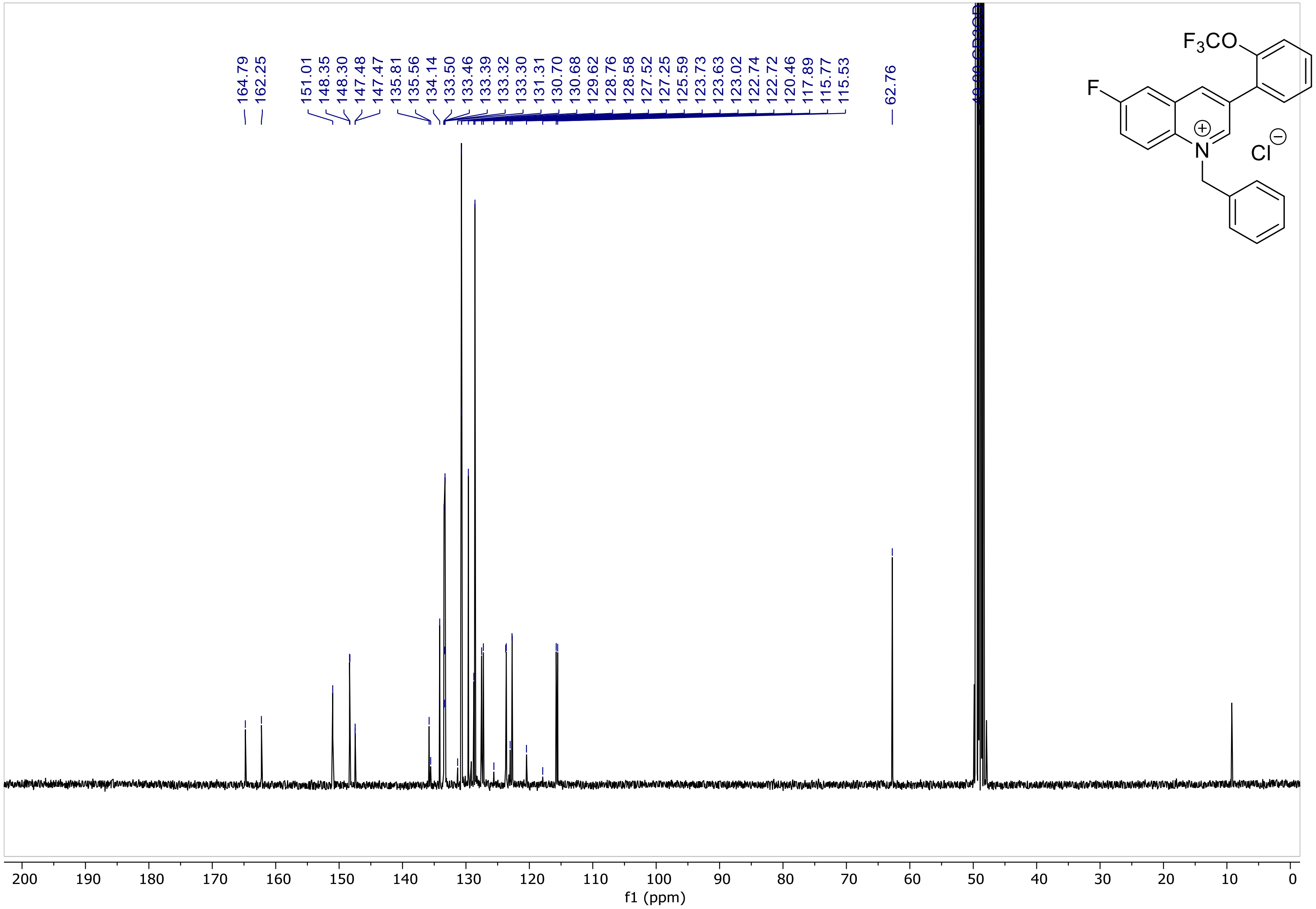

31: 1-Benzyl-6-fluoro-3-(2-trifluoromethoxyphenyl)quinolinium chloride – ^{19}F NMR (376 MHz, CD_3OD)

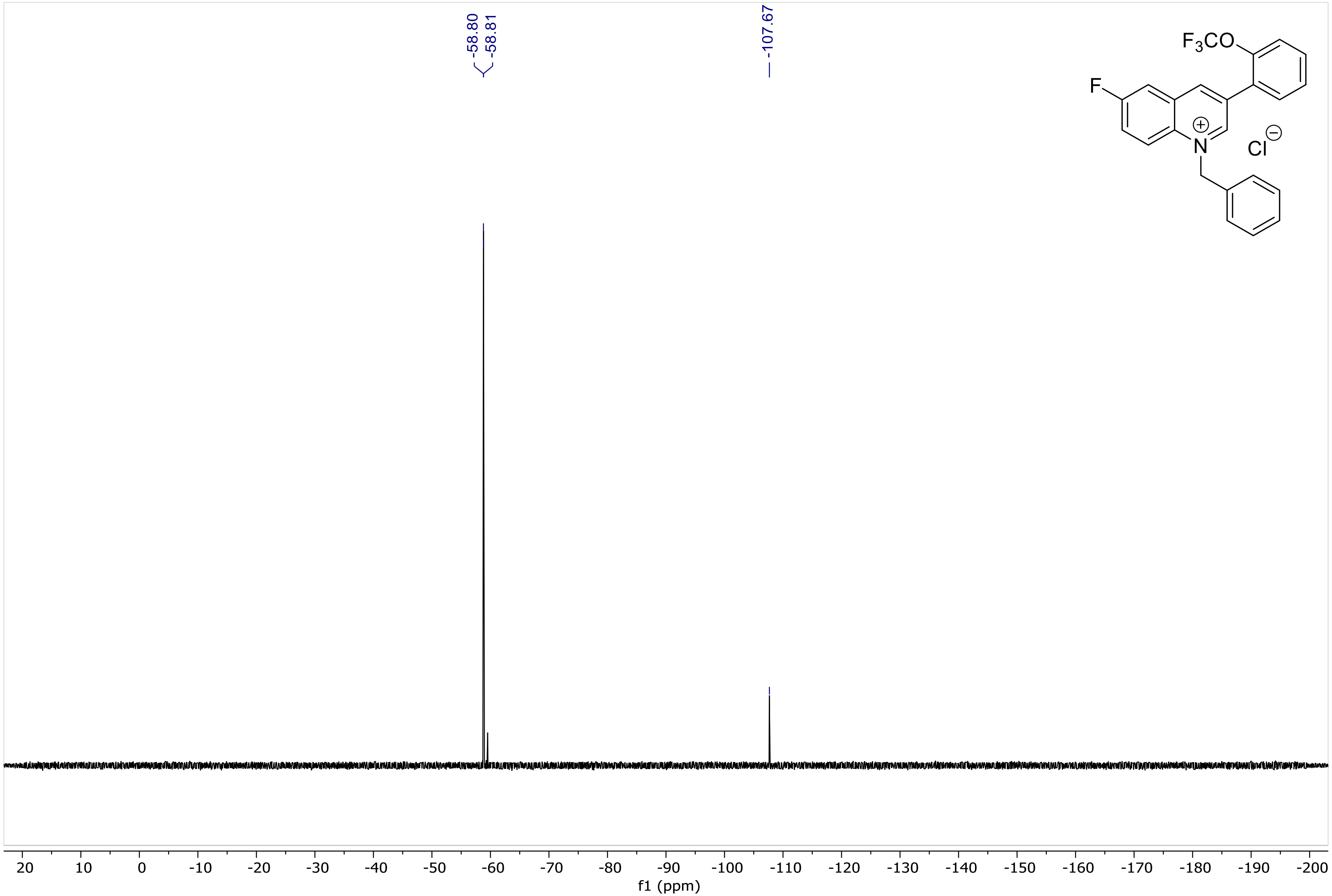

32: 1-Benzyl-6-fluoro-3-(2-fluoro-5-chlorophenyl)quinolinium chloride – ^{1}H NMR (400 MHz, CD_3OD)

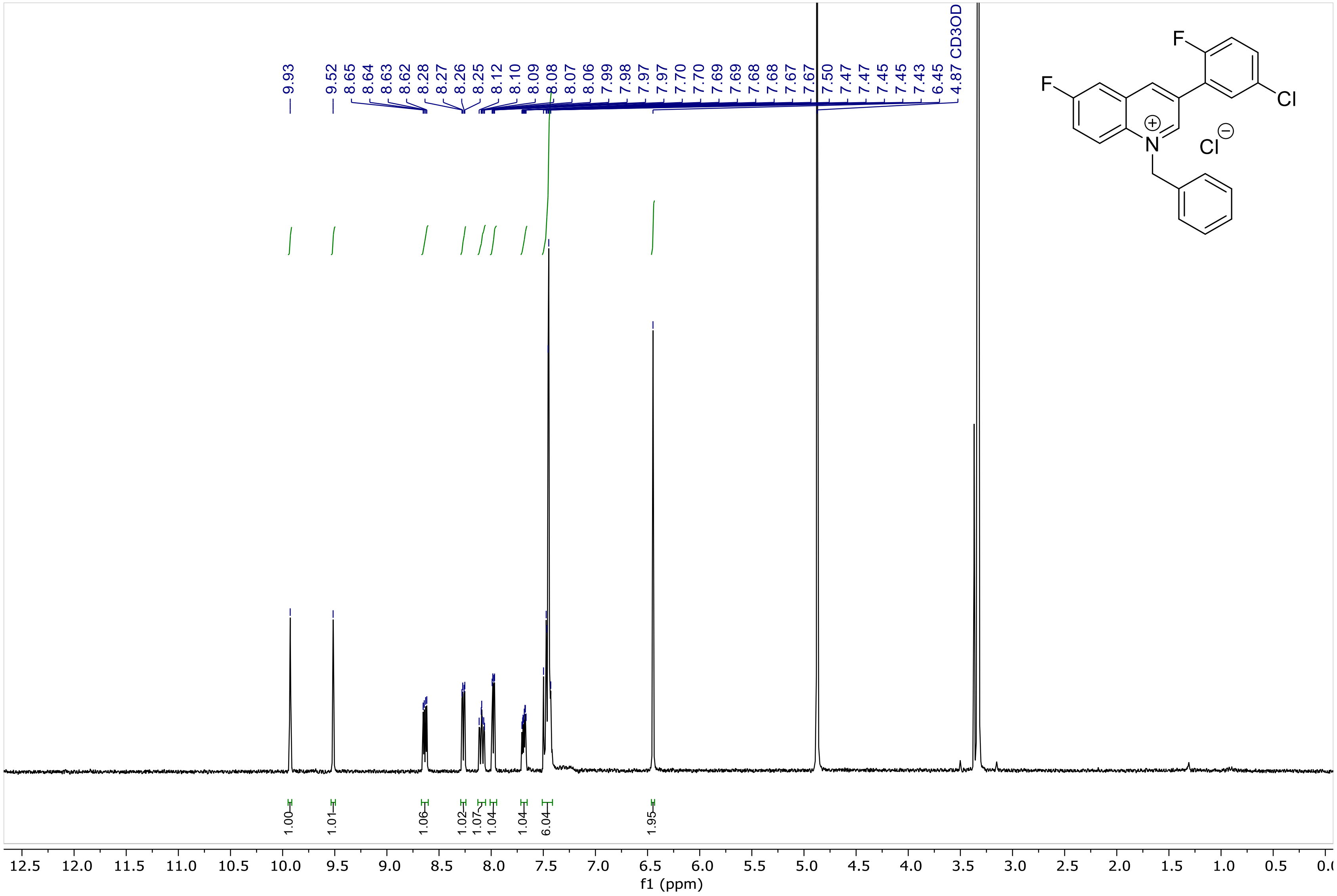

32: 1-Benzyl-6-fluoro-3-(2-fluoro-5-chlorophenyl)quinolinium chloride – $^{13}C\{^{1}H\}$ NMR (101 MHz, CD_3OD)

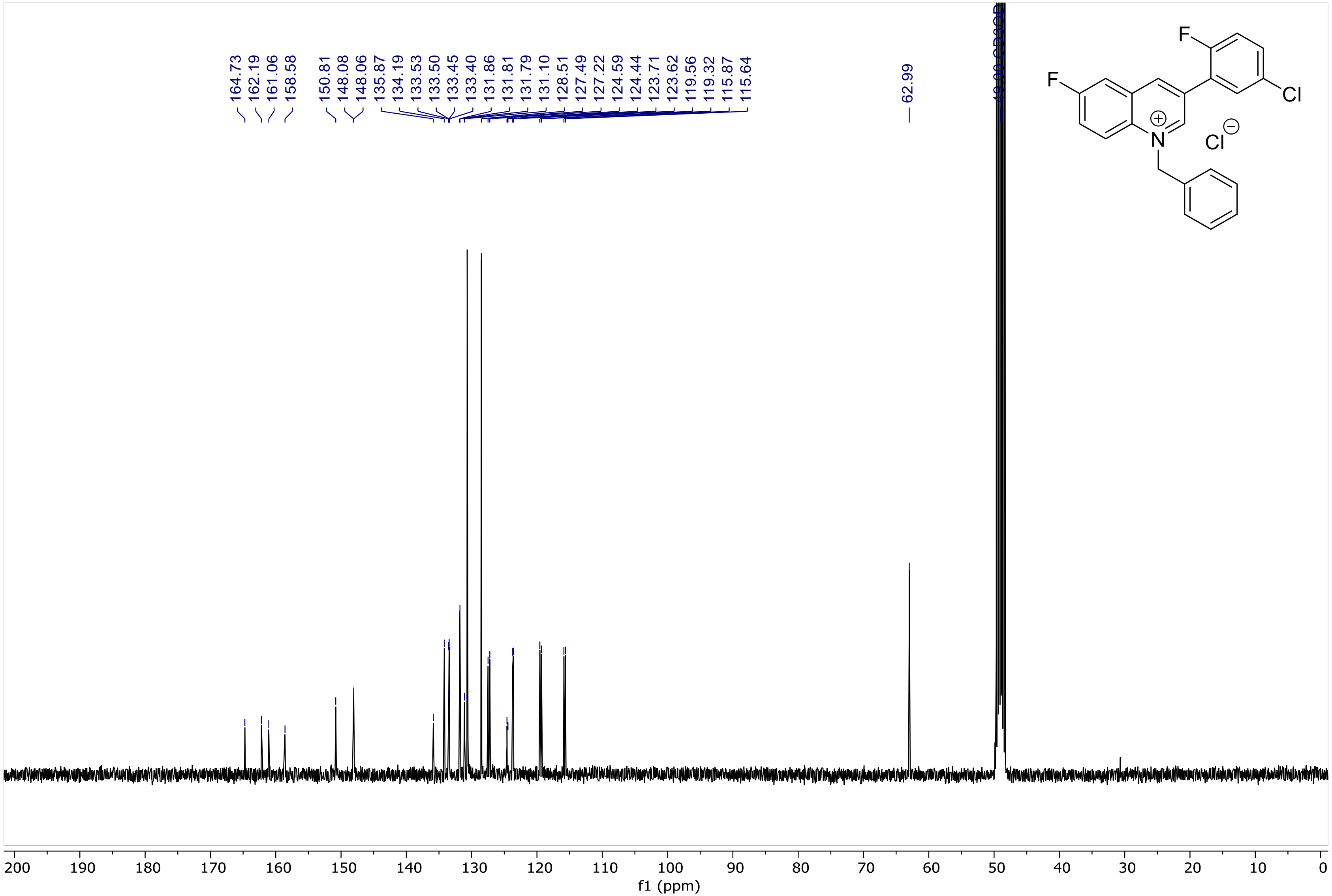

32: 1-Benzyl-6-fluoro-3-(2-fluoro-5-chlorophenyl)quinolinium chloride – ^{19}F NMR (376 MHz, CD_3OD)

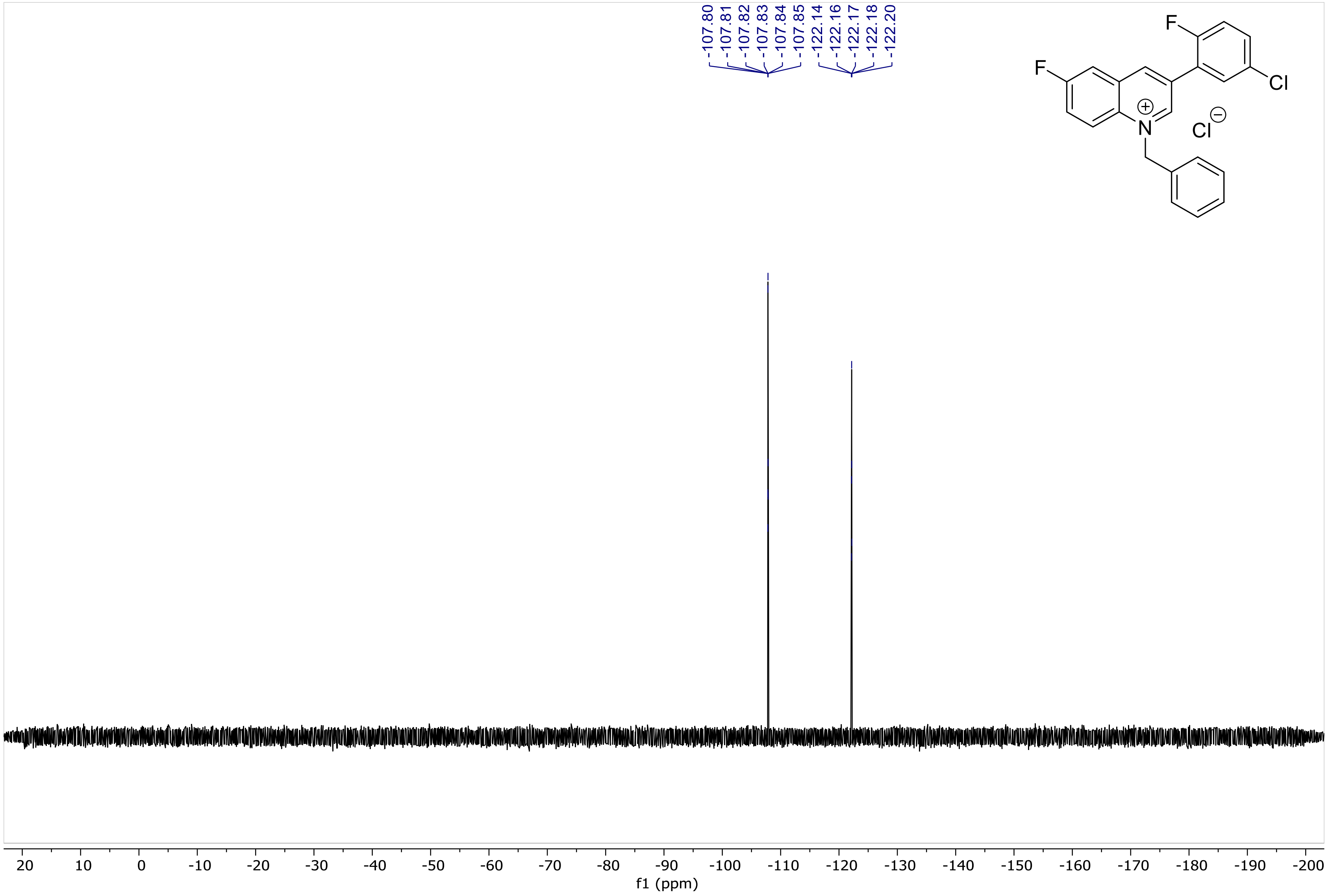

33: 1-Benzyl-6-fluoro-3-(2-pyridyl)quinolinium chloride – ^{1}H NMR (400 MHz, CD_3OD)

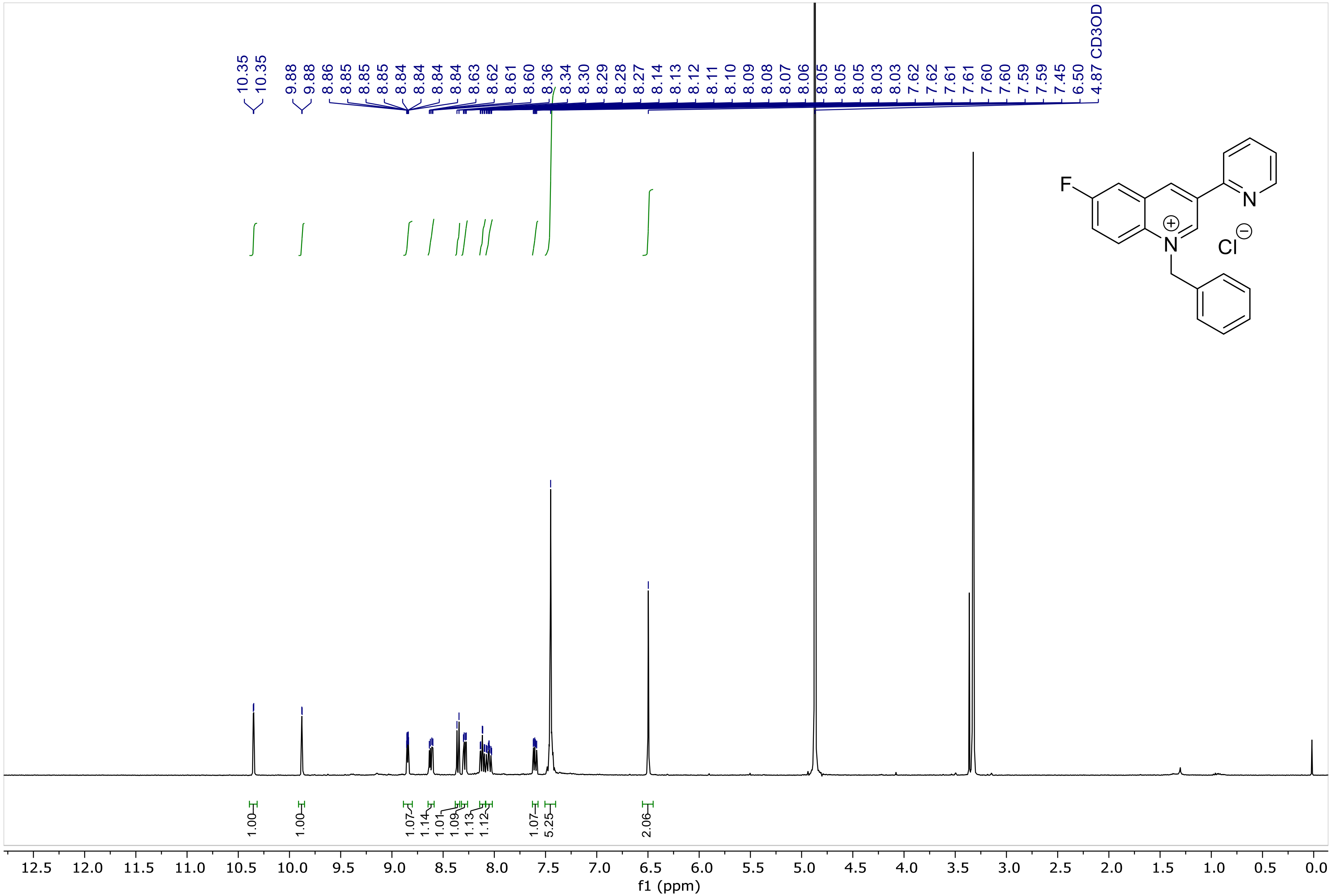

33: 1-Benzyl-6-fluoro-3-(2-pyridyl)quinolinium chloride – $^{13}C\{^{1}H\}$ NMR (101 MHz, CD_3OD)

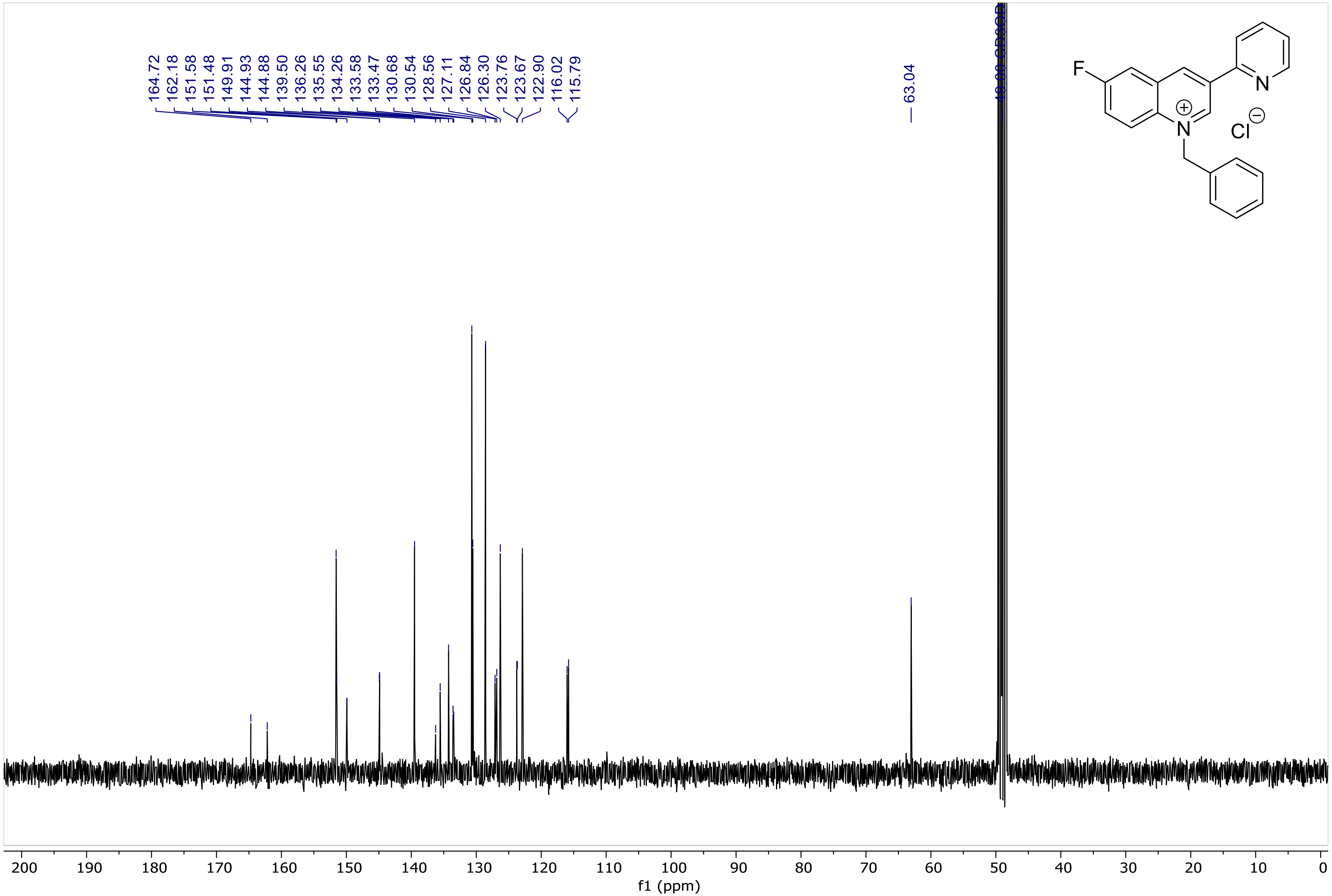

33: 1-Benzyl-6-fluoro-3-(2-pyridyl)quinolinium chloride – ^{19}F NMR (376 MHz, CD_3OD)

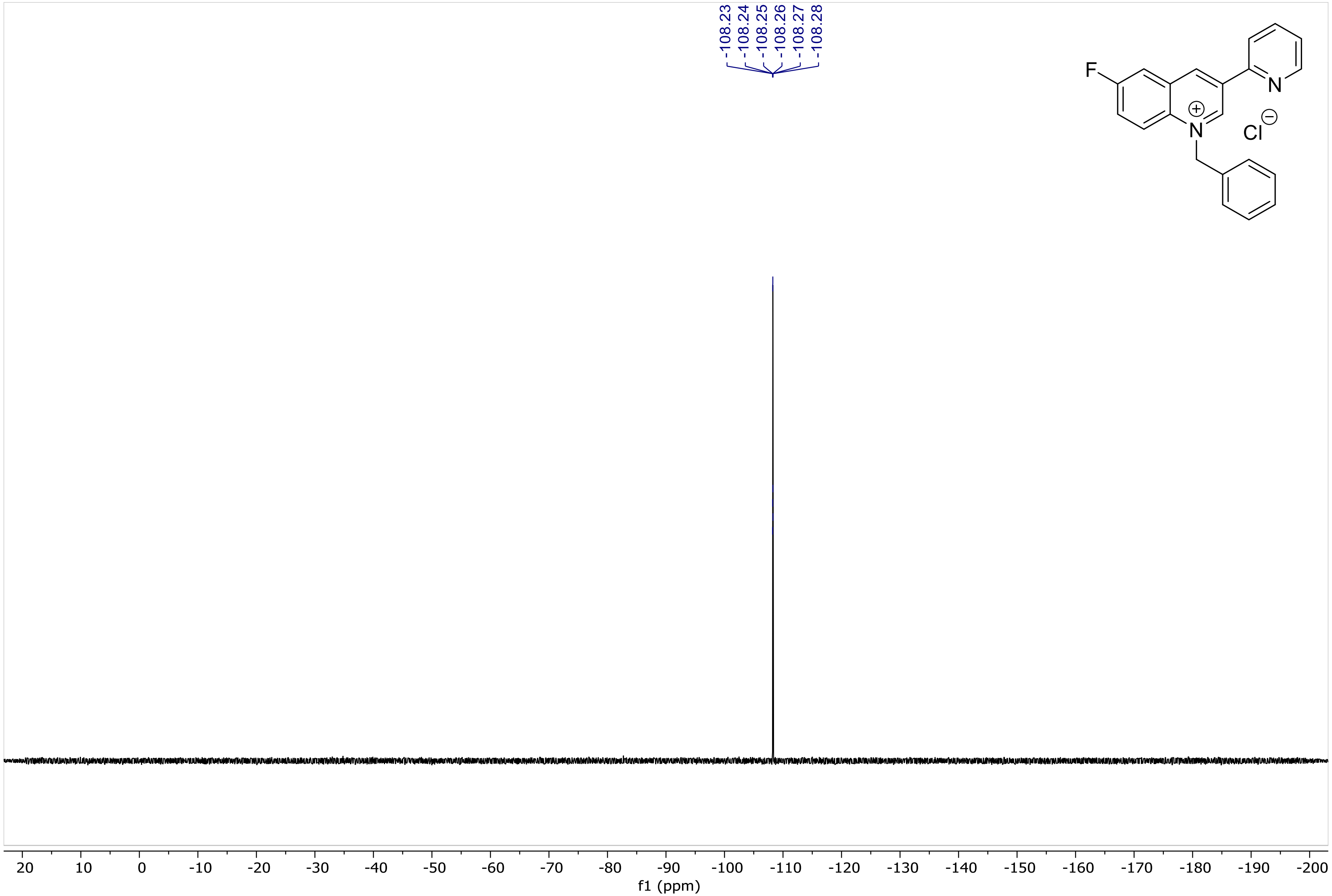

34: 1-Benzyl-3-phenylpyridinium chloride – ^{1}H NMR (400 MHz, CD_3OD)

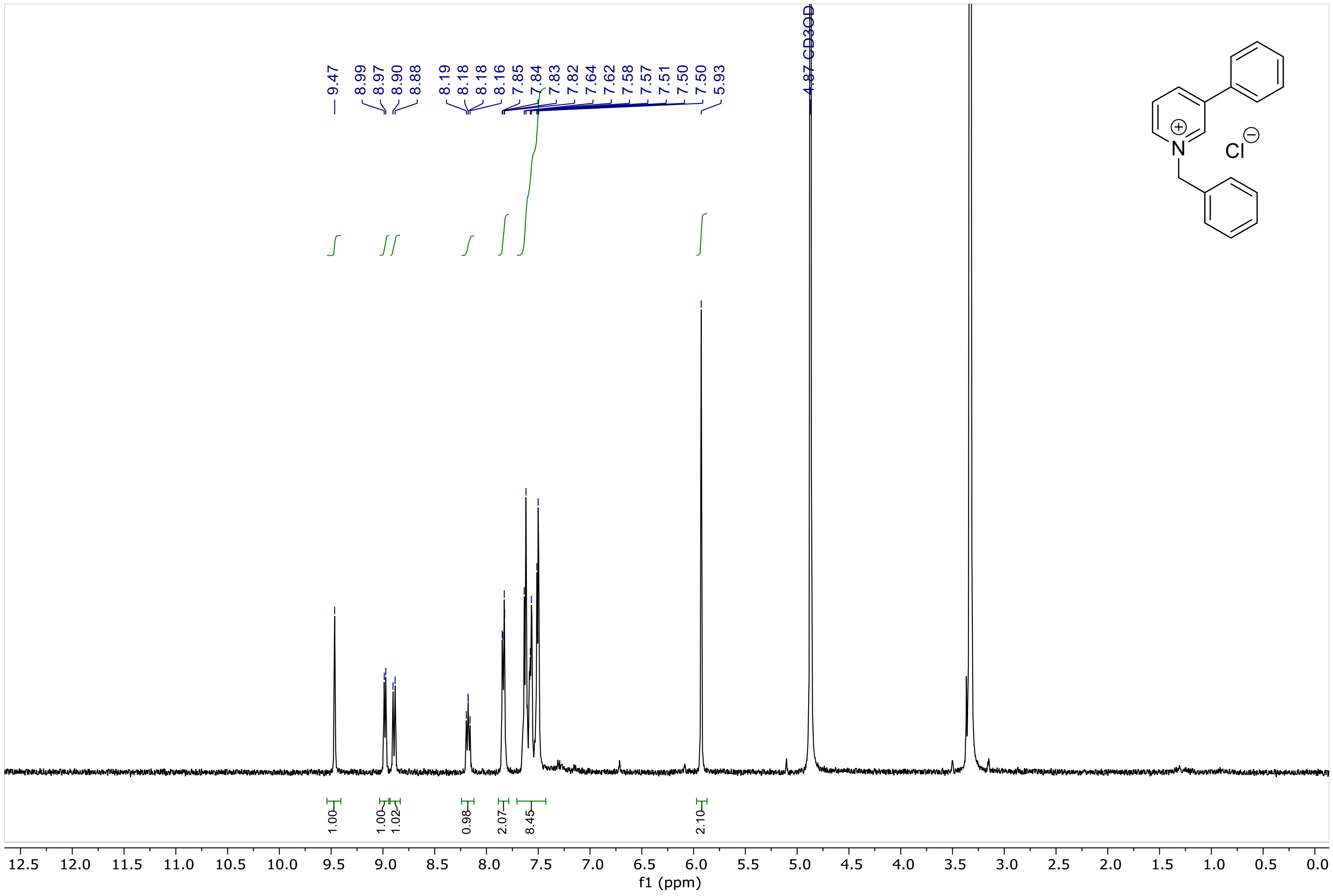

34: 1-Benzyl-3-phenylpyridinium chloride – $^{13}C\{^{1}H\}$ NMR (101 MHz, CD_3OD)

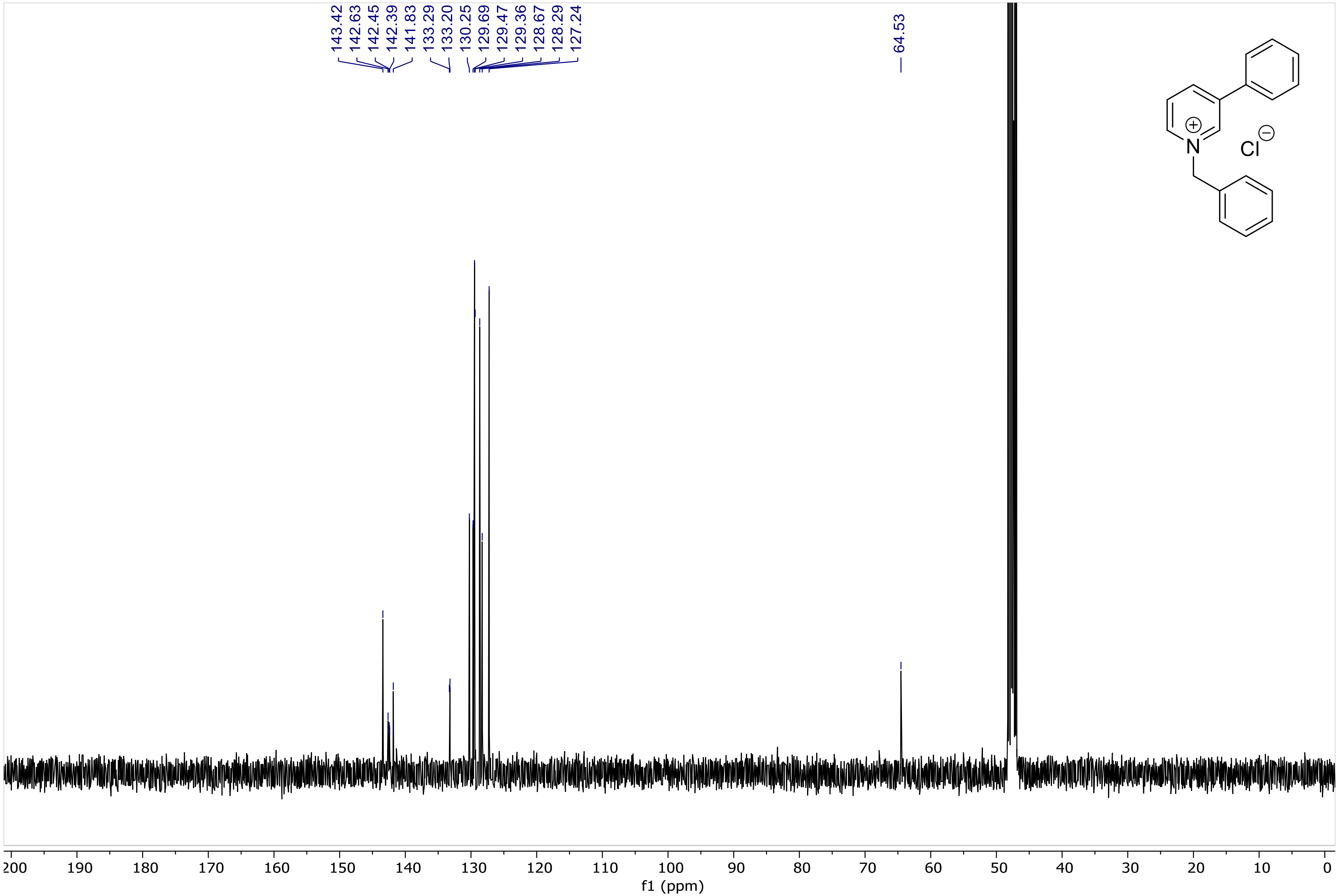

35: 1,3-Diphenylpyridin-1-ium chloride – ^{1}H NMR (400 MHz, CD_3OD)

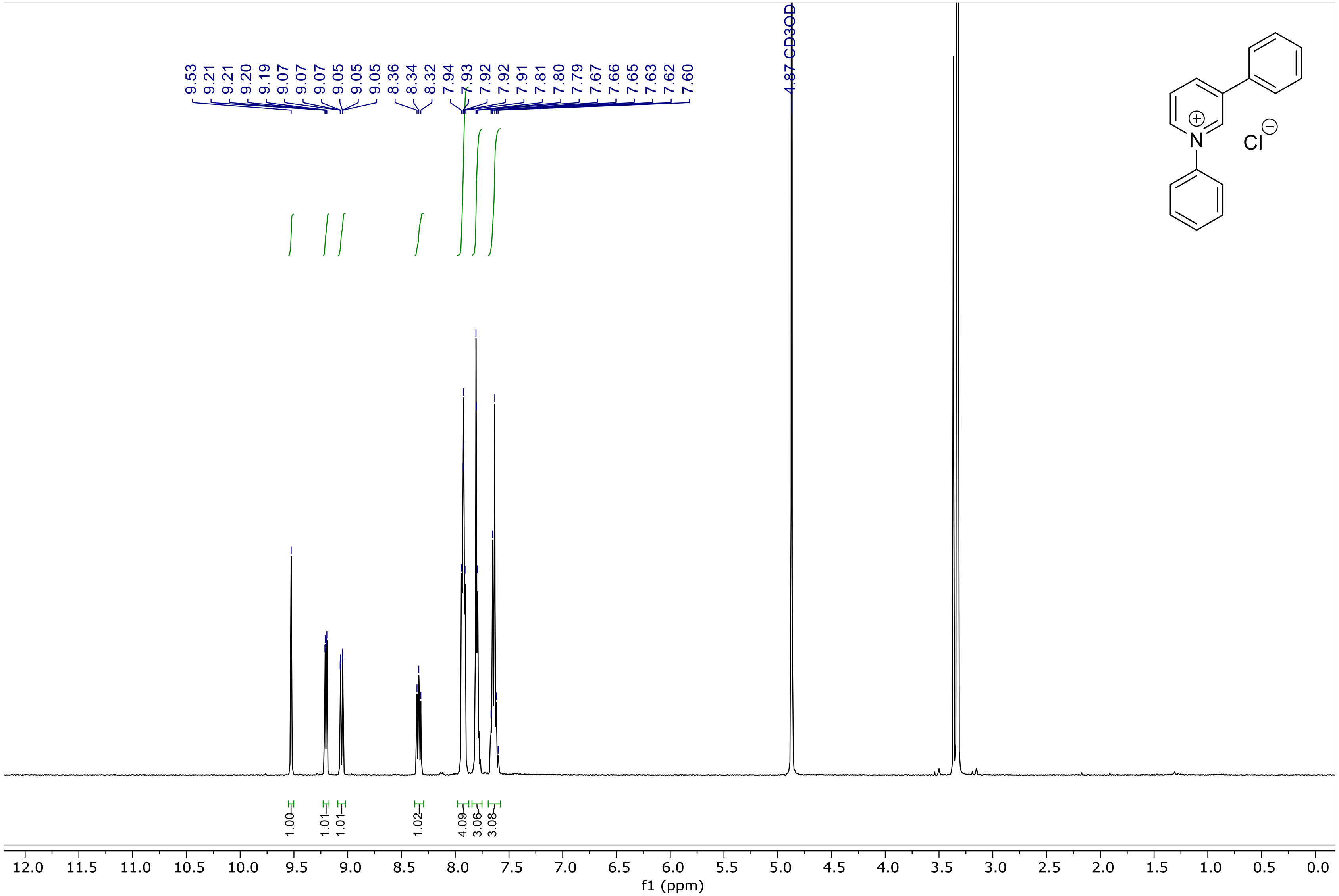

35: 1,3-Diphenylpyridin-1-ium chloride – $^{13}C\{^{1}H\}$ NMR (101 MHz, CD_3OD)

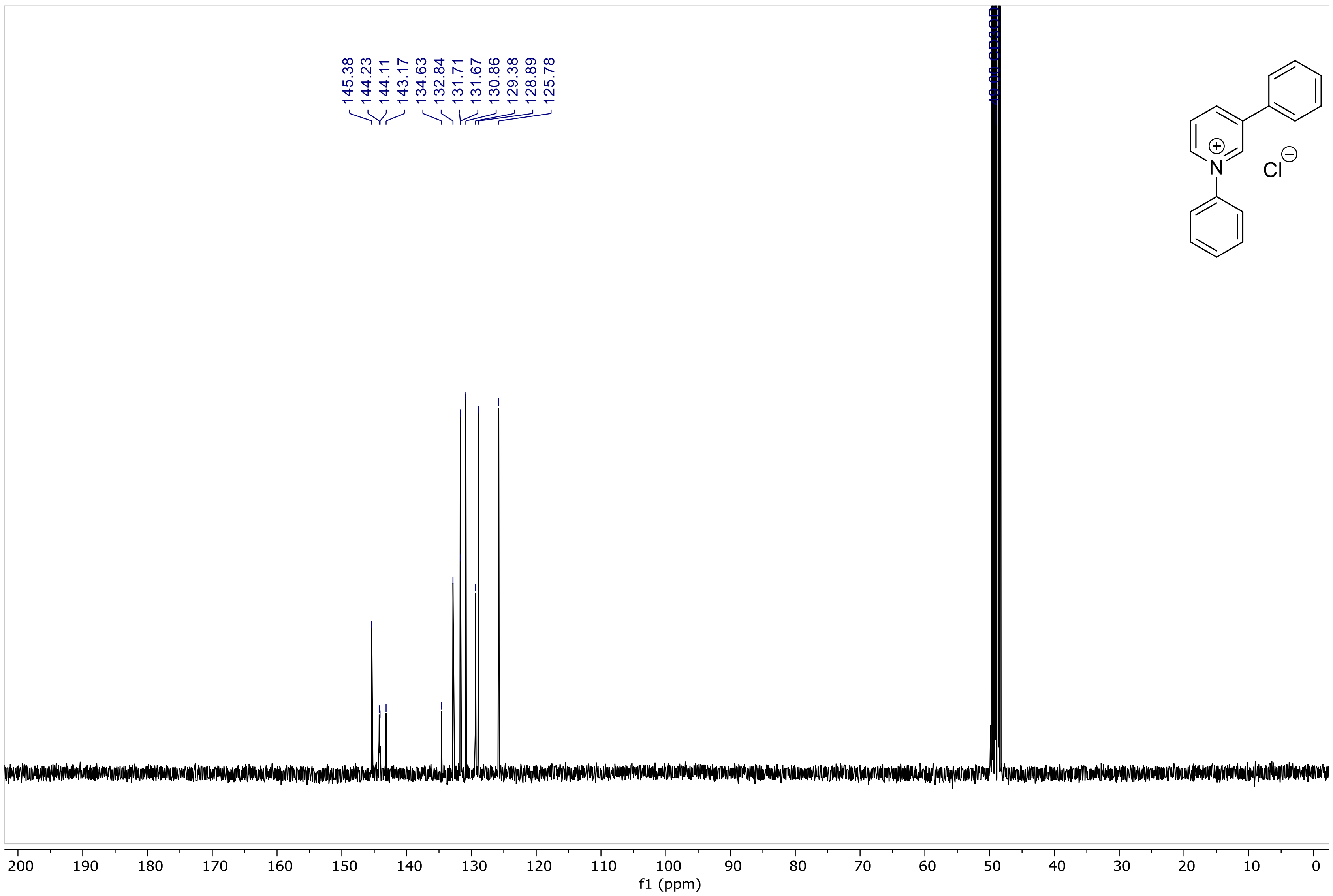

36: 1-Benzyl-3-phenyl-2,4-dimethylpyridinium chloride – ^{1}H NMR (400 MHz, DMSO-d_6)

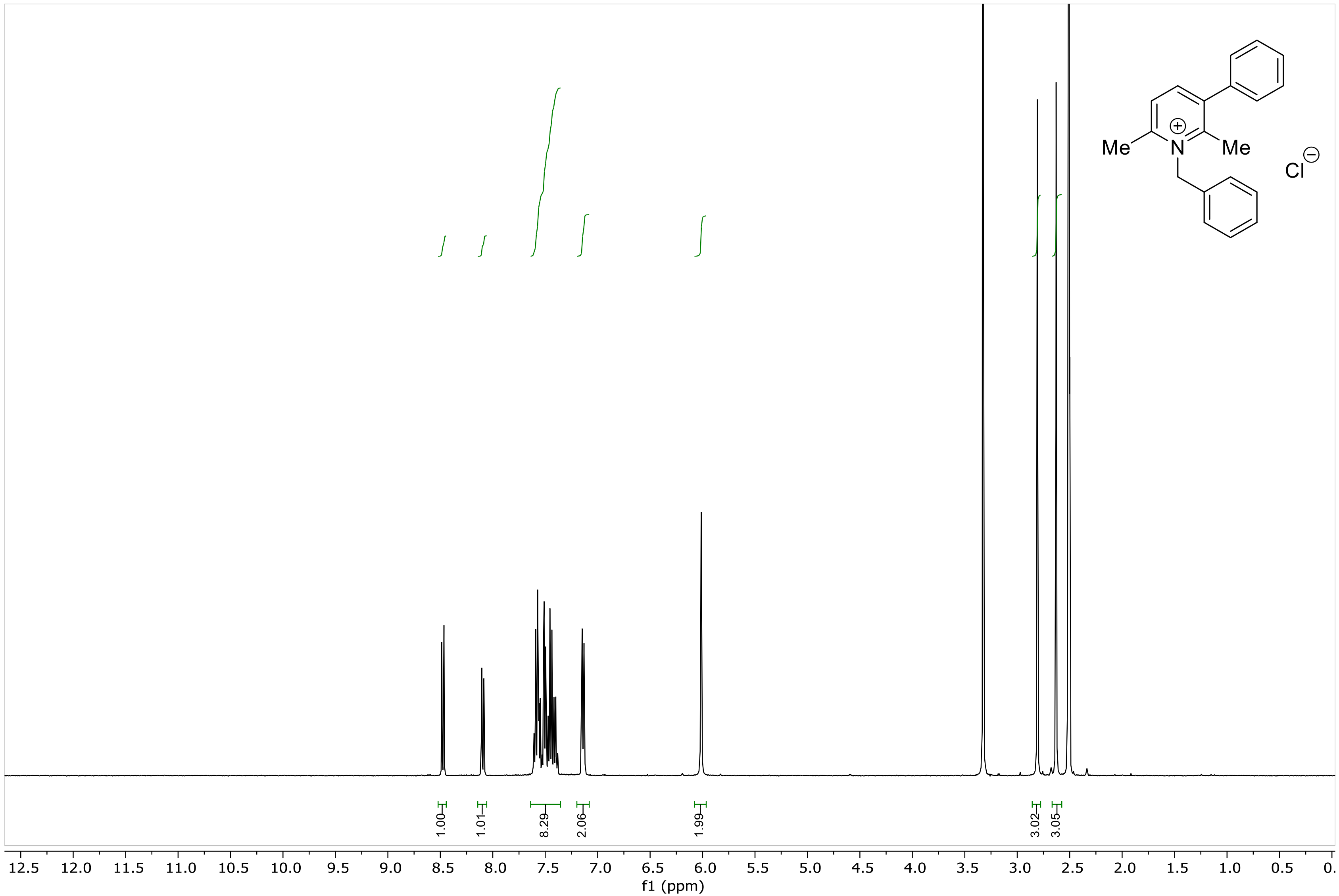

36: 1-Benzyl-3-phenyl-2,4-dimethylpyridinium chloride – $^{13}C\{^1H\}$ NMR (101 MHz, DMSO-d_6)

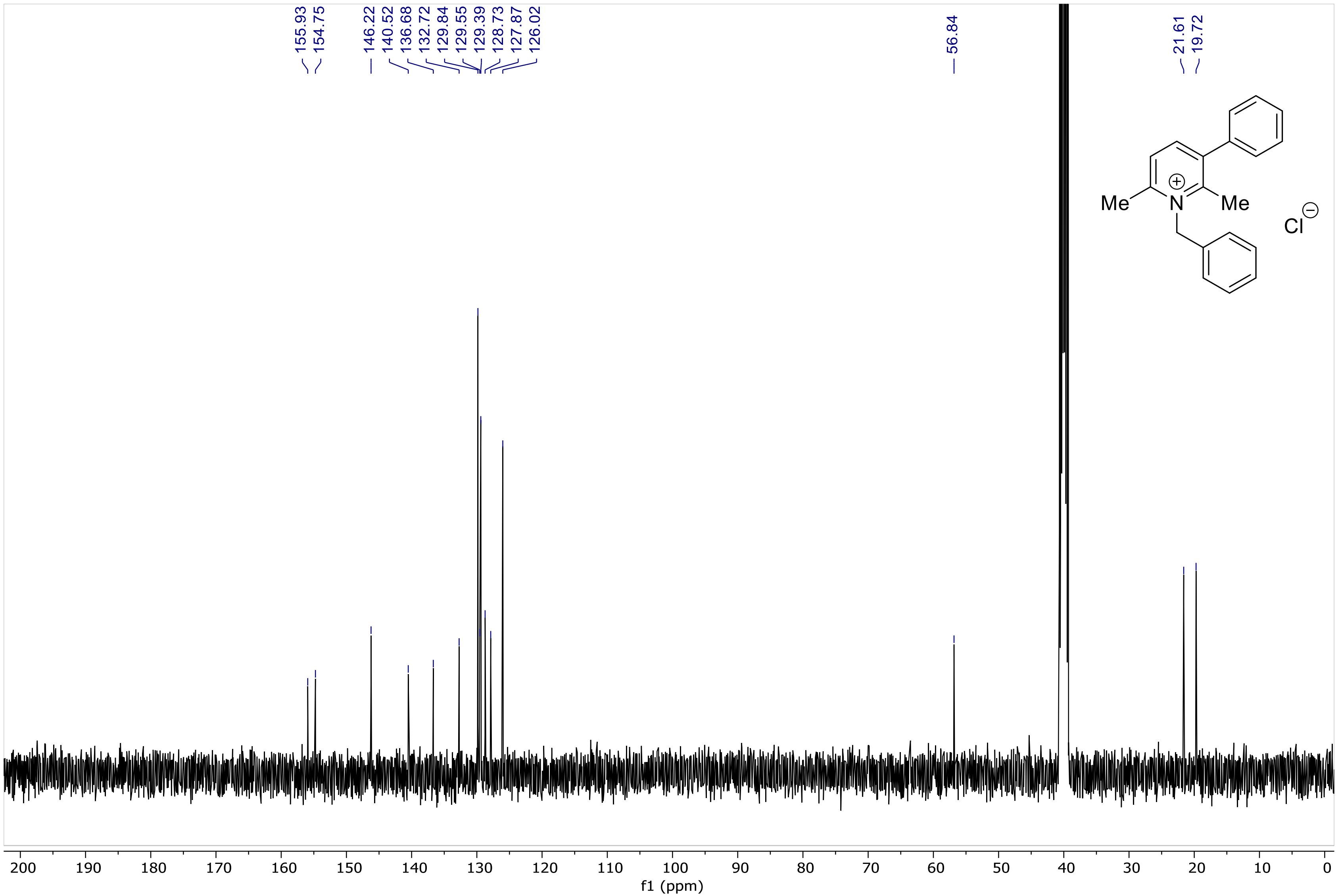

37: 2-Benzyl-8-oxo-4-phenyl-5,6,7,8-tetrahydroisoquinolin-2-ium chloride and 2-benzyl-8-oxo-4-phenyl-5,6,7,8-tetrahydroisoquinolin-2-ium chloride– ^{1}H NMR (400 MHz, DMSO-d$_6$)

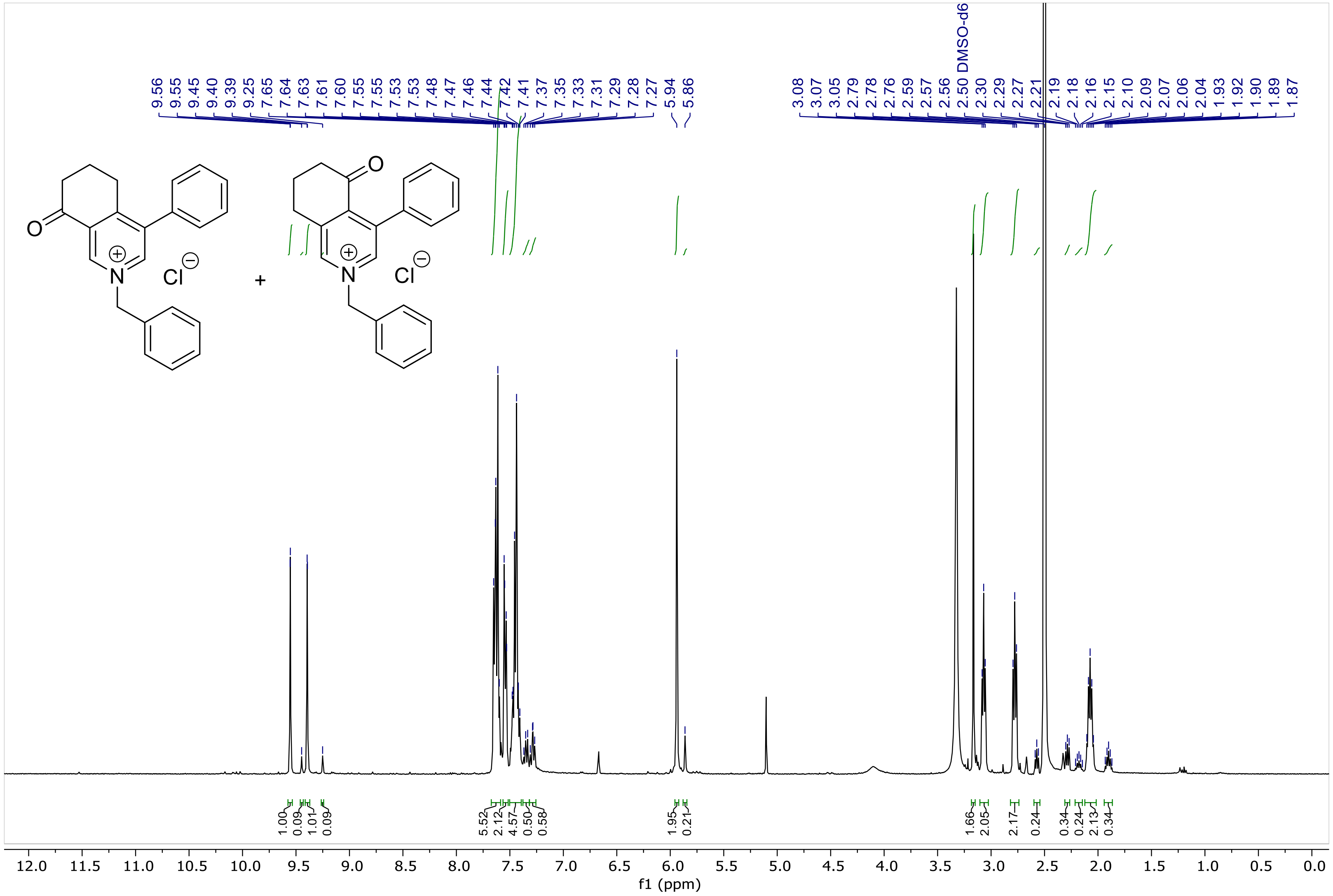

37: 2-Benzyl-8-oxo-4-phenyl-5,6,7,8-tetrahydroisoquinolin-2-ium chloride and 2-benzyl-5-oxo-4-phenyl-5,6,7,8-tetrahydroisoquinolin-2-ium chloride– $^{13}C\{^{1}H\}$ NMR (101 MHz, DMSO-d$_6$)

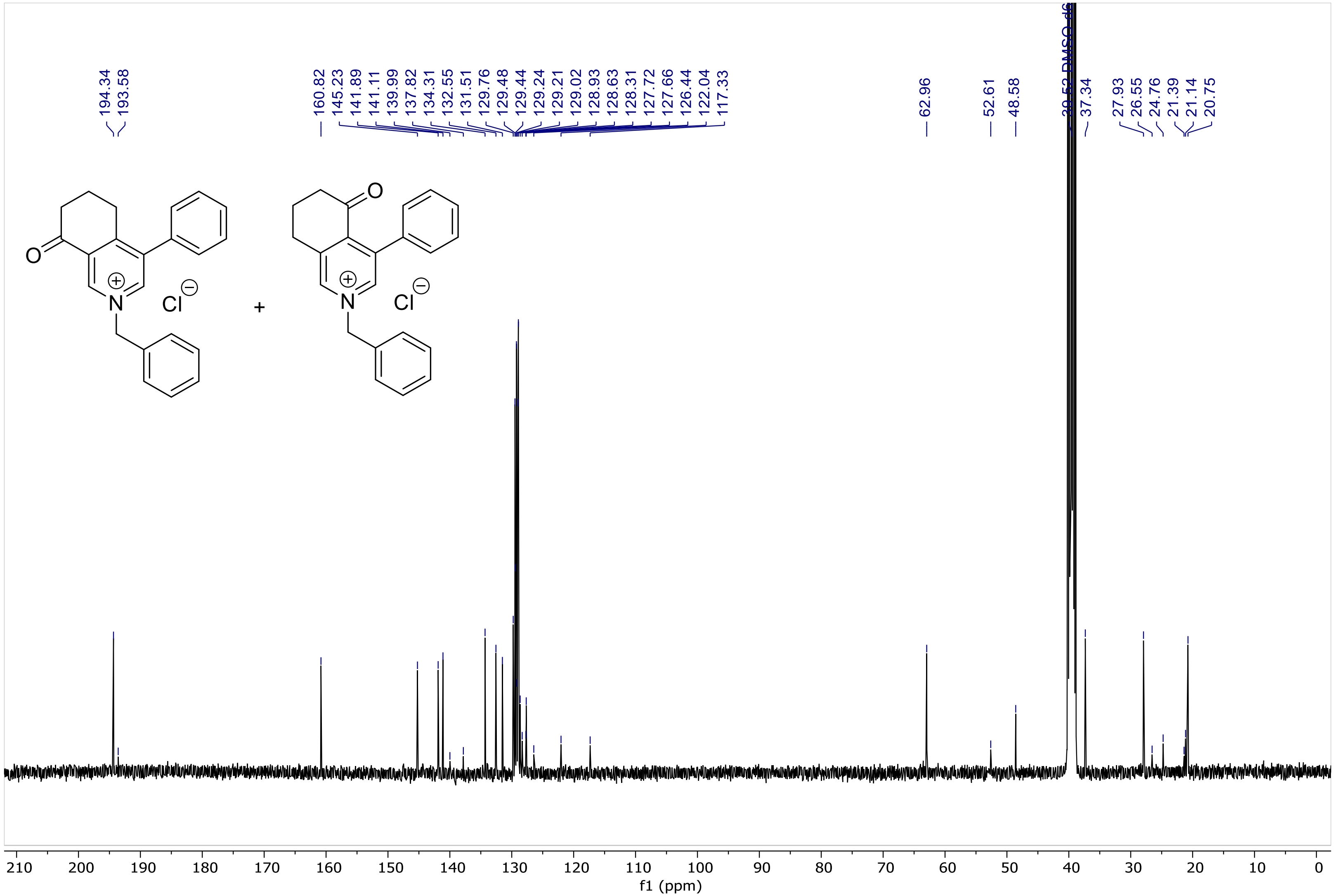

38: 1-benzyl-3-(methoxycarbonyl)-4,5-diphenylpyridin-1-ium chloride and 1-benzyl-4-(methoxycarbonyl)-3,5-diphenylpyridin-1-ium chloride– ^{1}H NMR (400 MHz, DMSO-d$_6$)

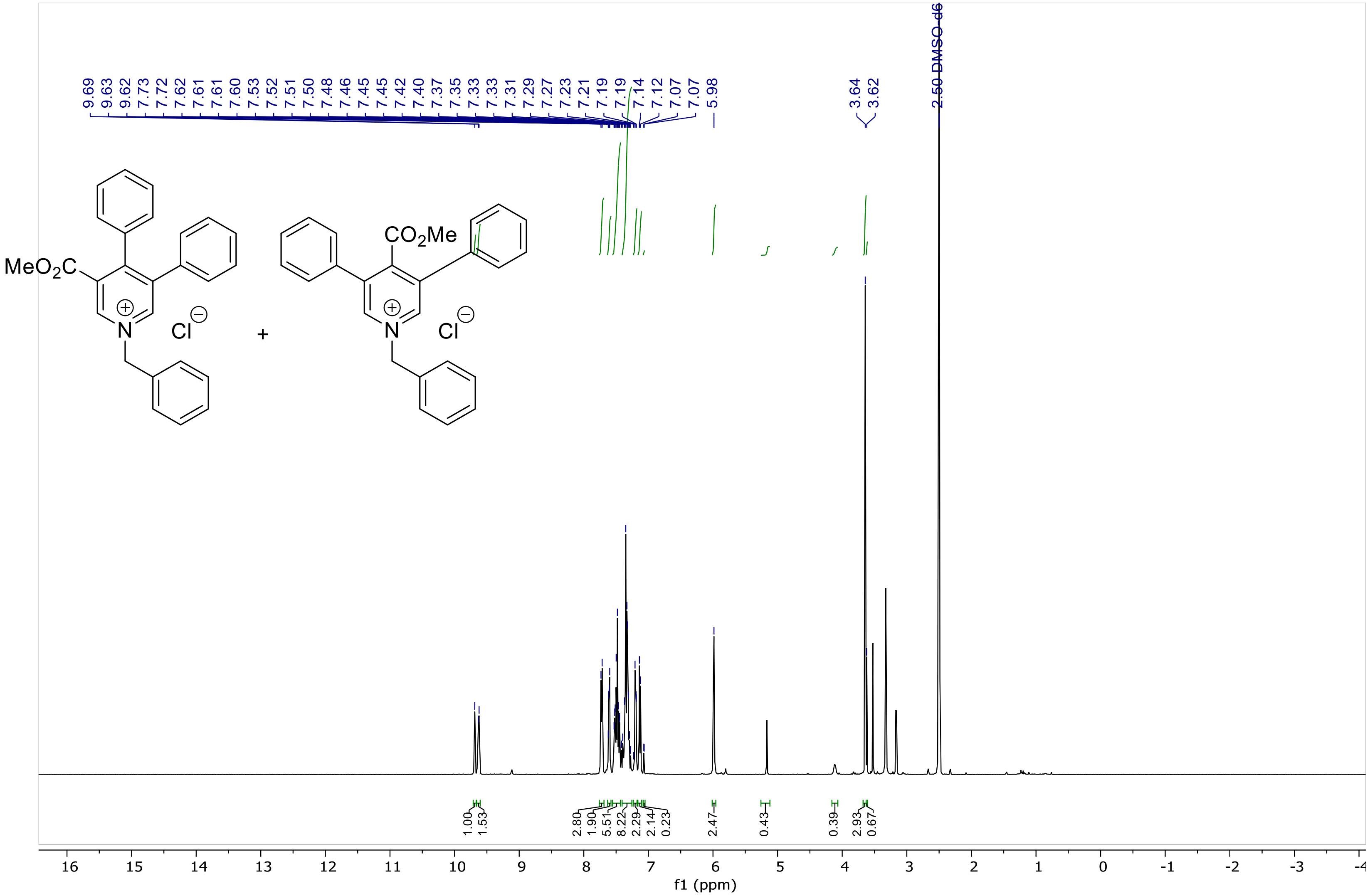

38: 1-benzyl-3-(methoxycarbonyl)-4,5-diphenylpyridin-1-ium chloride and 1-benzyl-4-(methoxycarbonyl)-3,5-diphenylpyridin-1-ium chloride – $^{13}C\{^{1}H\}$ NMR (101 MHz, DMSO-d_6)

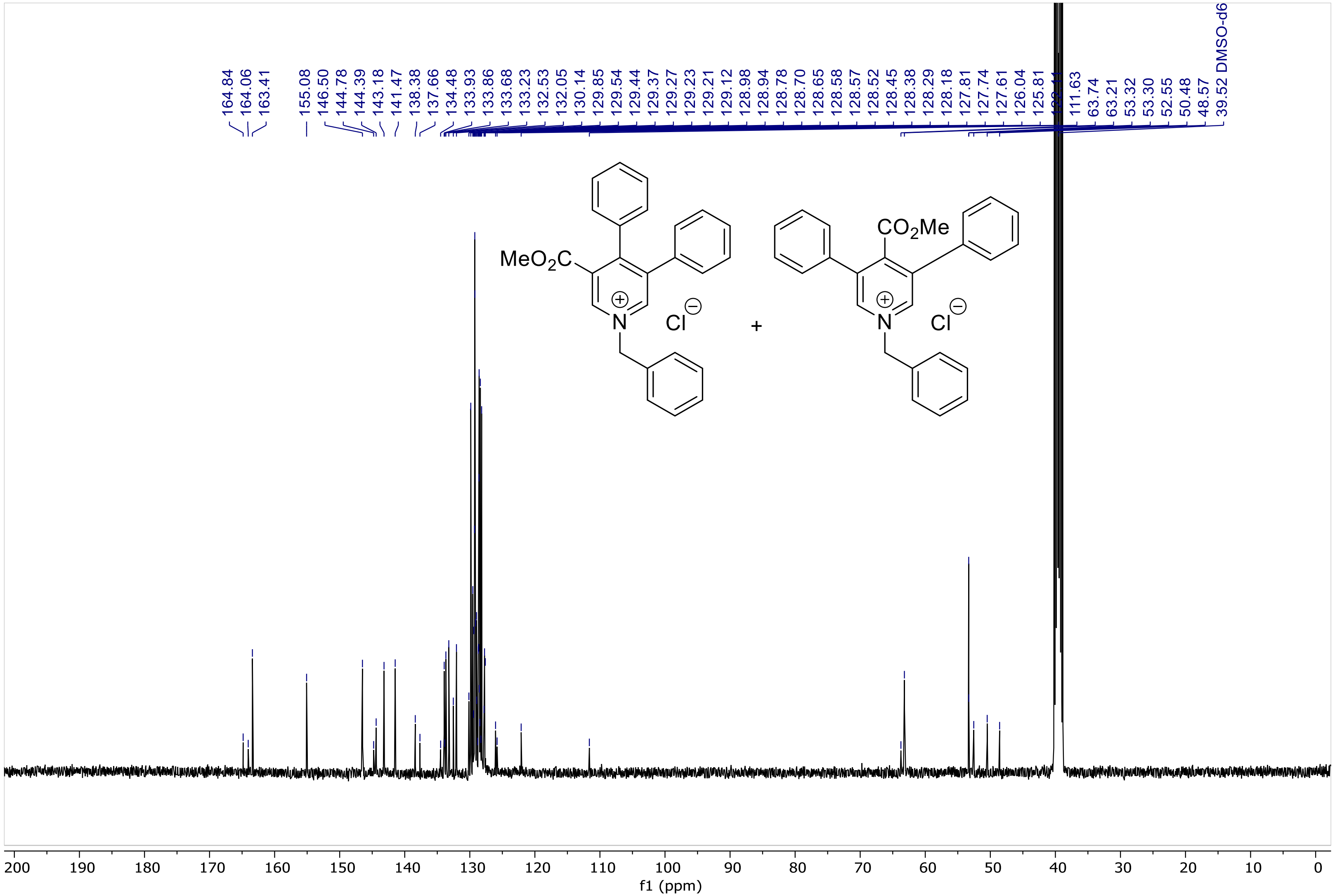

39: 1-benzyl-3,4-diphenylpyridin-1-ium chloride and 1-benzyl-3,5-diphenylpyridin-1-ium chloride – ^{1}H NMR (400 MHz, DMSO-d$_6$)

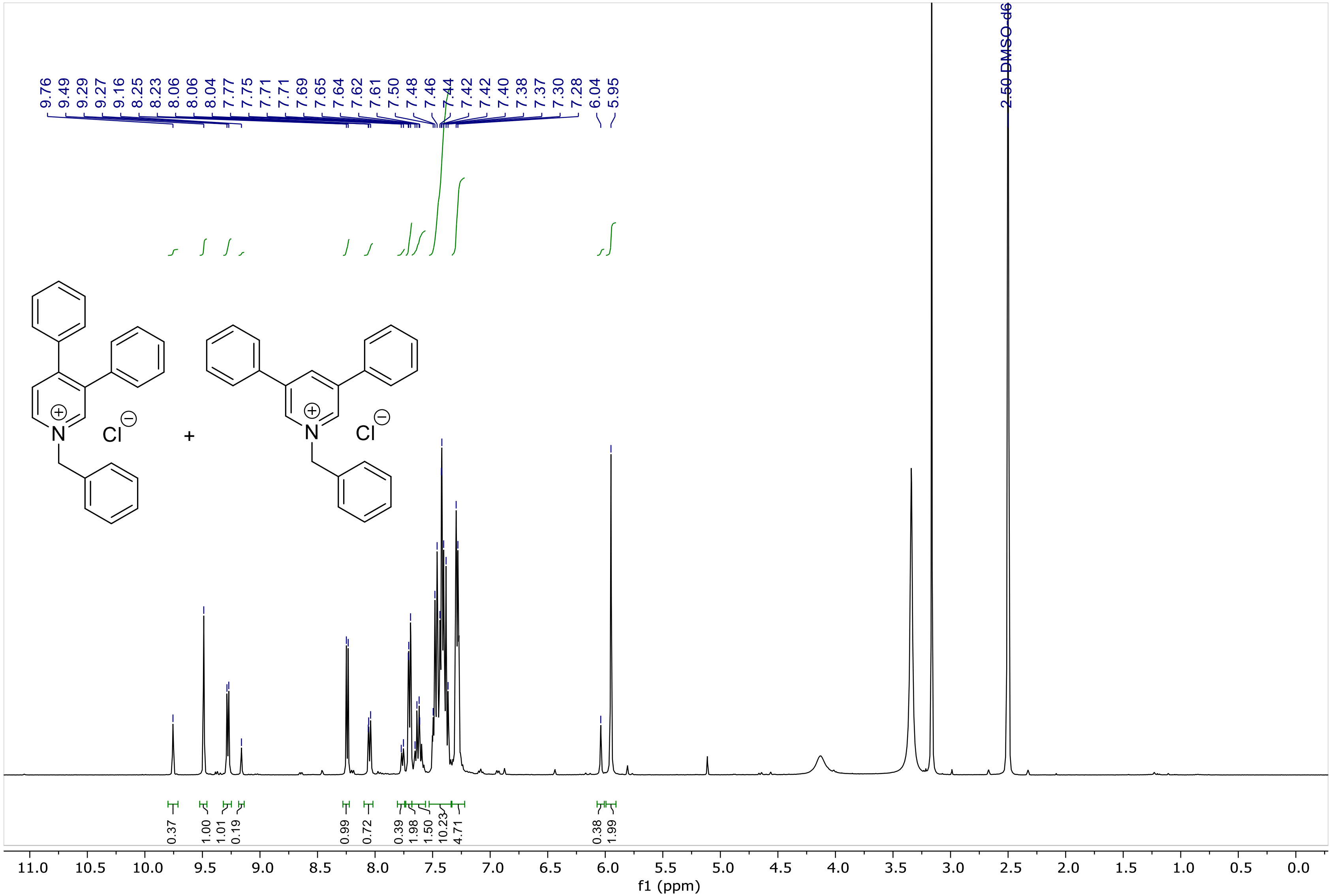

39: 1-benzyl-3,4-diphenylpyridin-1-ium chloride and 1-benzyl-3,5-diphenylpyridin-1-ium chloride – $^{13}C\{^{1}H\}$ NMR (101 MHz, DMSO-d$_6$)

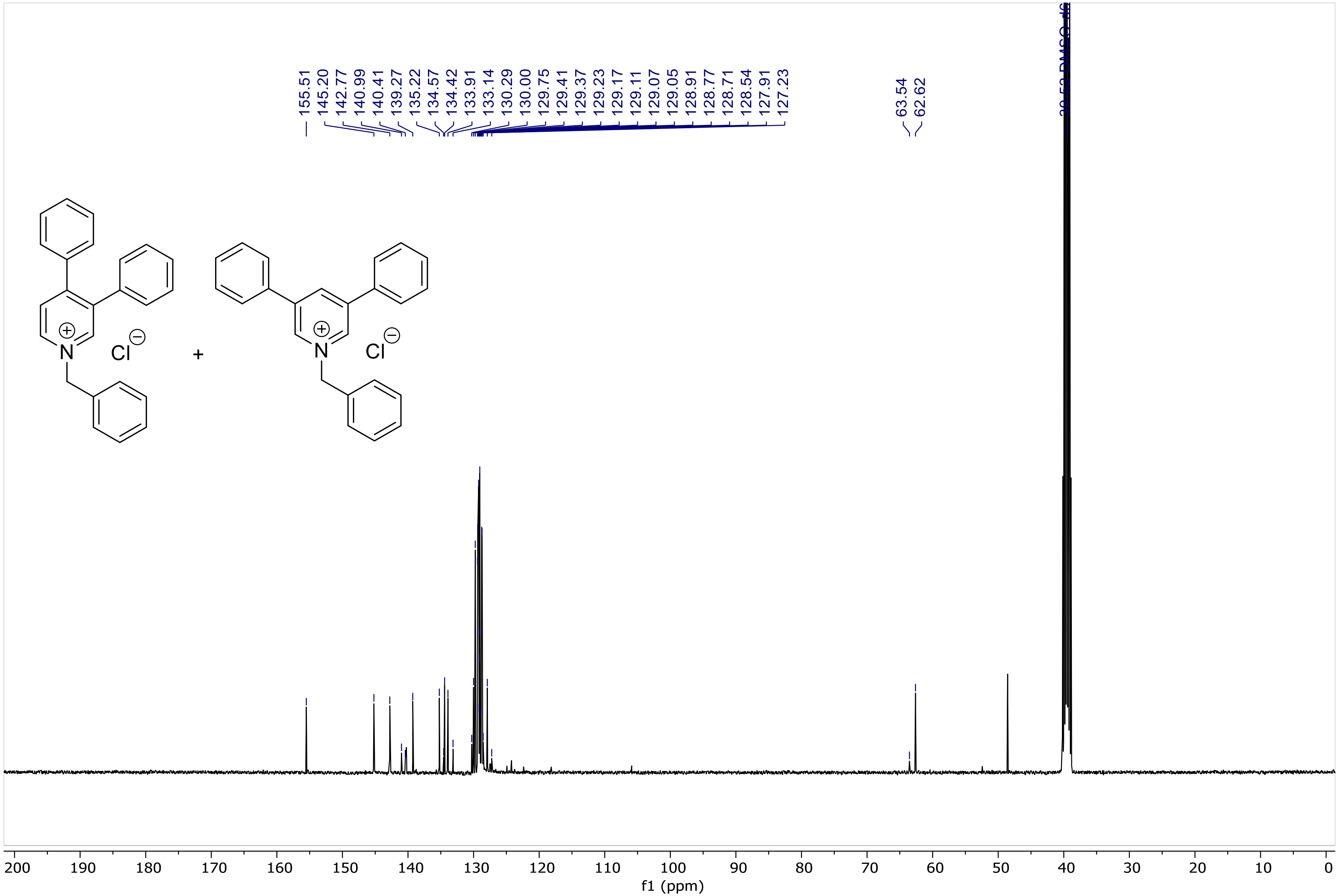

40: 1-Benzyl-2-methyl-5-phenylpyridin-1-ium chloride and 1-benzyl-2-methyl-3-phenylpyridin-1-ium chloride – ^{1}H NMR (400 MHz, DMSO-d$_6$)

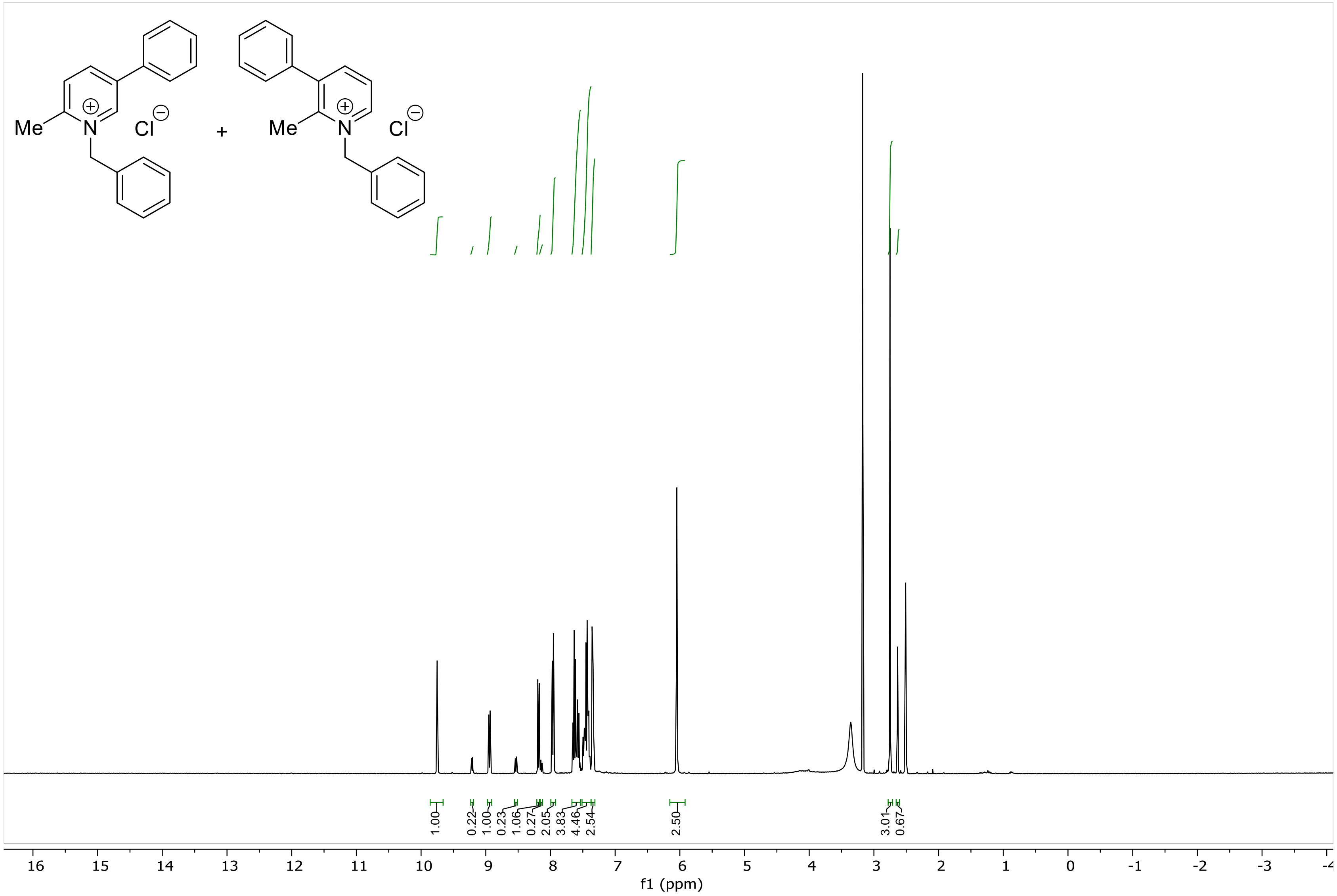

40: 1-Benzyl-2-methyl-5-phenylpyridin-1-ium chloride and 1-benzyl-2-methyl-3-phenylpyridin-1-ium chloride – $^{13}C\{^{1}H\}$ NMR (101 MHz, DMSO-d$_6$)

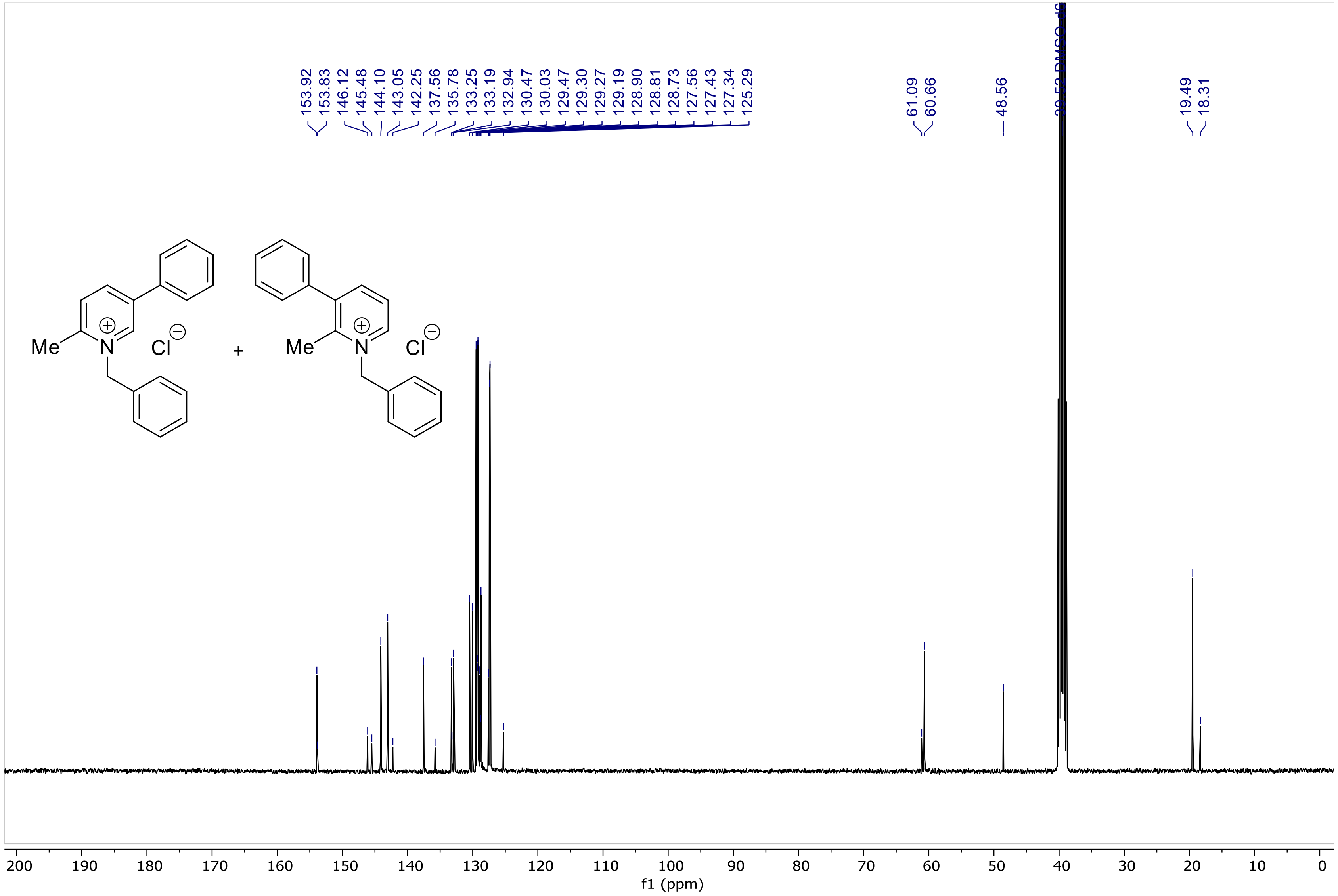

41: 1-benzyl-2-isopropyl-5-phenylpyridin-1-ium chloride and 1-benzyl-2-isopropyl-3-phenylpyridin-1-ium chloride – ^{1}H NMR (400 MHz, CD_3OD)

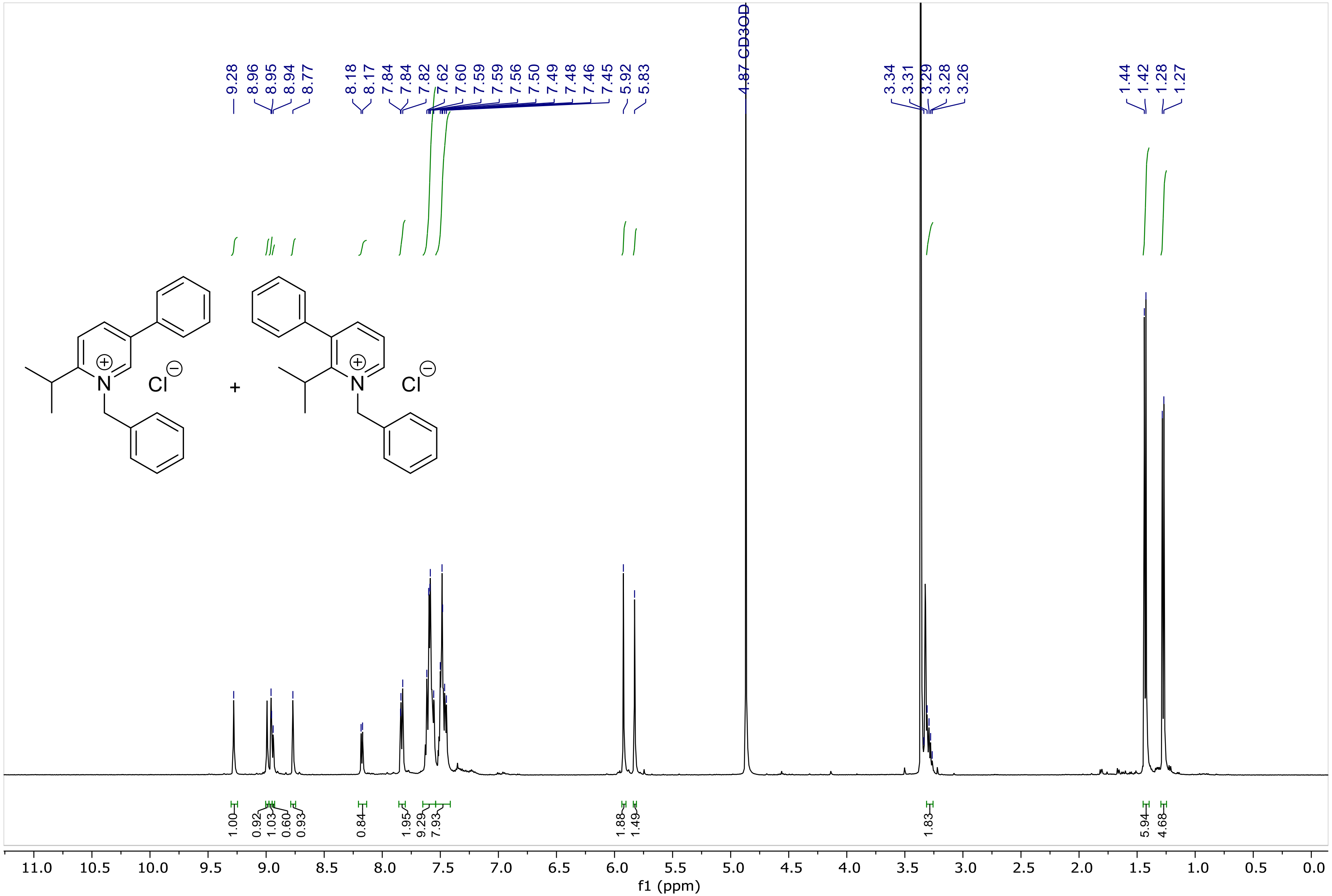

41: 1-benzyl-2-isopropyl-5-phenylpyridin-1-ium chloride and 1-benzyl-2-isopropyl-3-phenylpyridin-1-ium chloride – $^{13}C\{^1H\}$ NMR (101 MHz, CD_3OD)

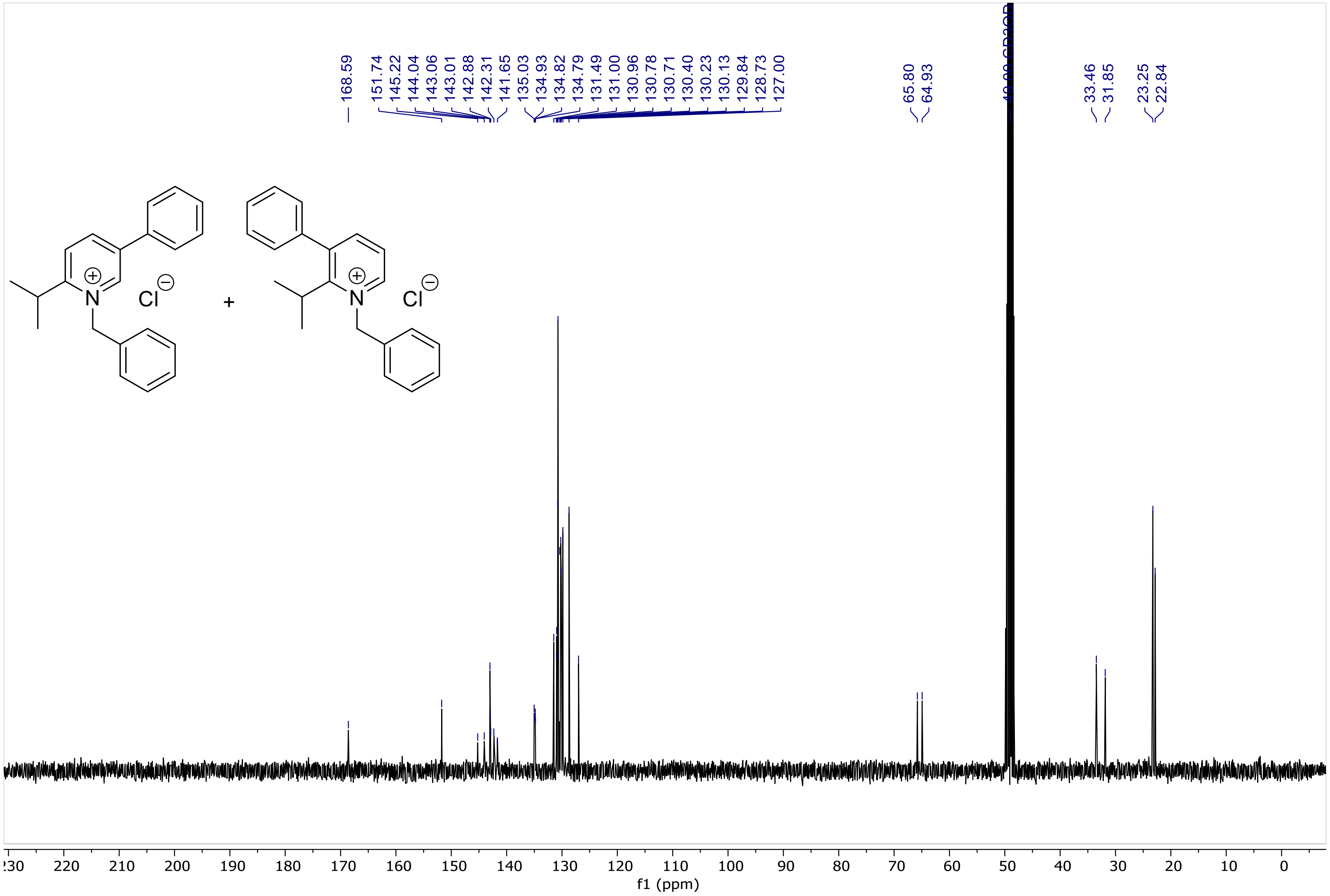

42: 2-(*tert*-Butoxycarbonyl)-1-(4-fluorophenyl)-7-phenyl-1,2,3,4-tetrahydropyrido[1,2-a]pyrazin-5-ium chloride and 2-(*tert*-butoxycarbonyl)-1-(4-fluorophenyl)-9-phenyl-1,2,3,4-tetrahydropyrido[1,2-a]pyrazin-5-ium chloride – ^{1}H NMR (400 MHz, CD_3OD)

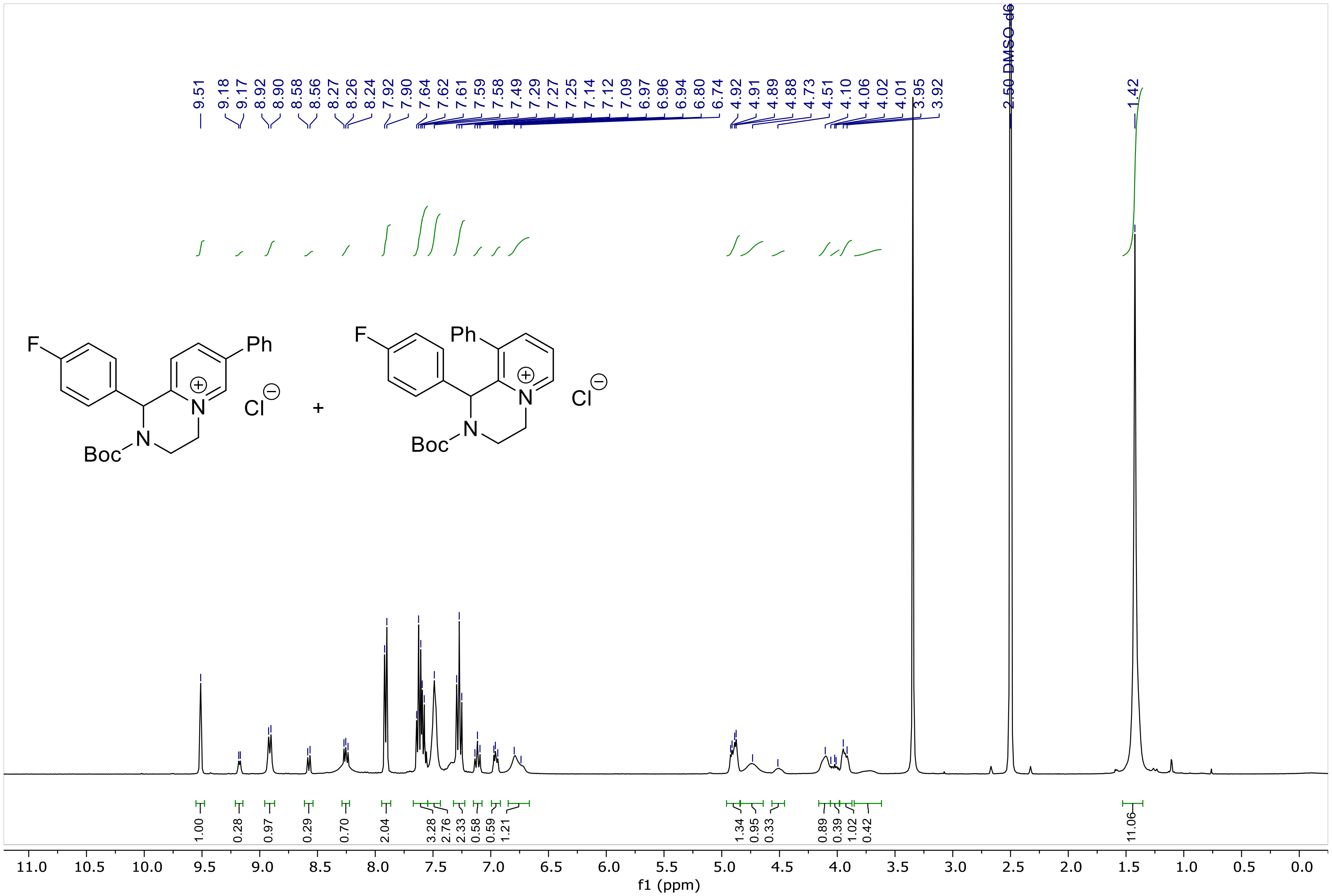

42: 2-(*tert*-Butoxycarbonyl)-1-(4-fluorophenyl)-7-phenyl-1,2,3,4-tetrahydropyrido[1,2-a]pyrazin-5-ium chloride and 2-(*tert*-butoxycarbonyl)-1-(4-fluorophenyl)-9-phenyl-1,2,3,4-tetrahydropyrido[1,2-a]pyrazin-5-ium chloride – $^{13}C\{^1H\}$ NMR (101 MHz, CD_3OD)

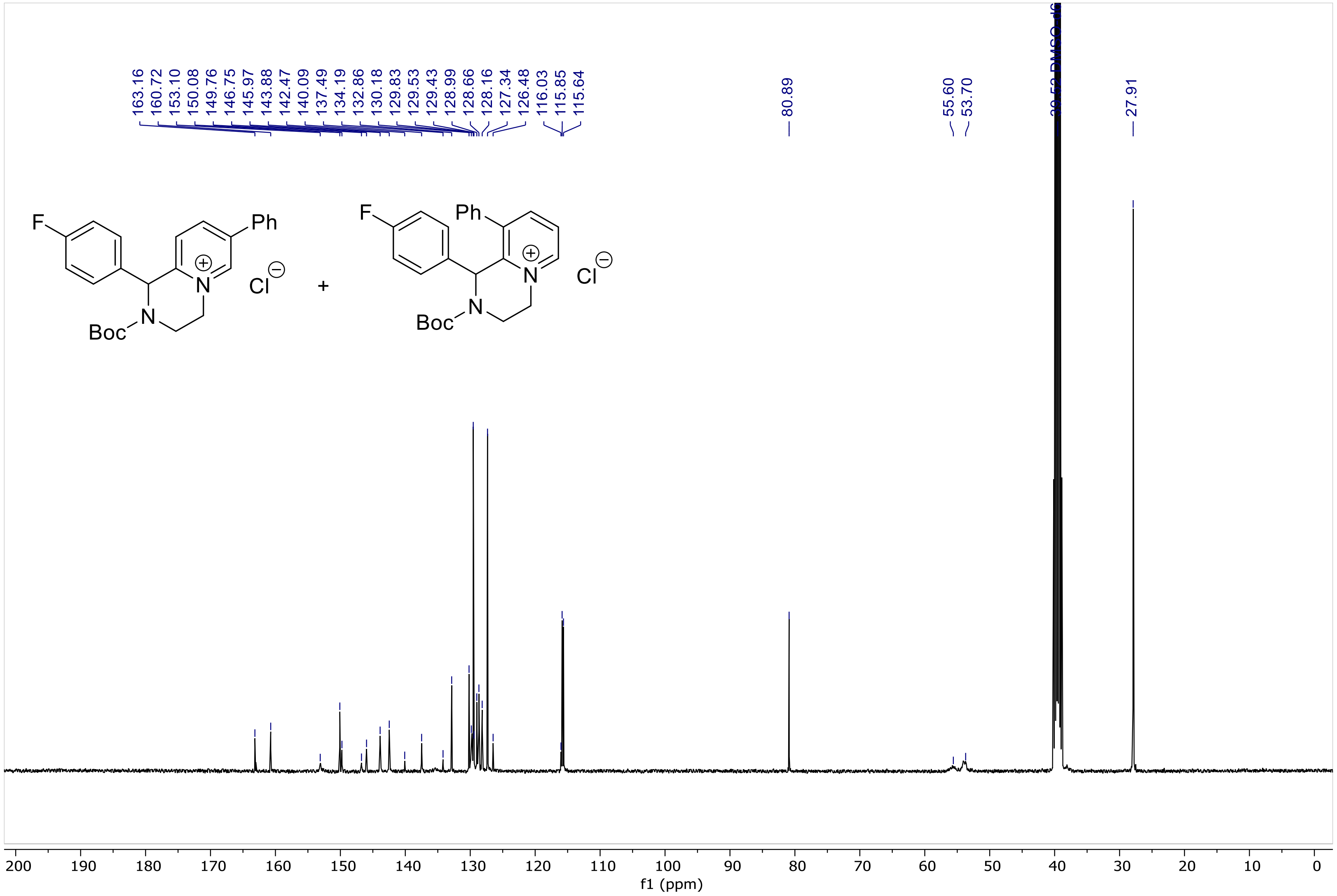

42: 2-(*tert*-Butoxycarbonyl)-1-(4-fluorophenyl)-7-phenyl-1,2,3,4-tetrahydropyrido[1,2-a]pyrazin-5-ium chloride and 2-(*tert*-butoxycarbonyl)-1-(4-fluorophenyl)-9-phenyl-1,2,3,4-tetrahydropyrido[1,2-a]pyrazin-5-ium chloride – ^{19}F NMR (376 MHz, CD_3OD)

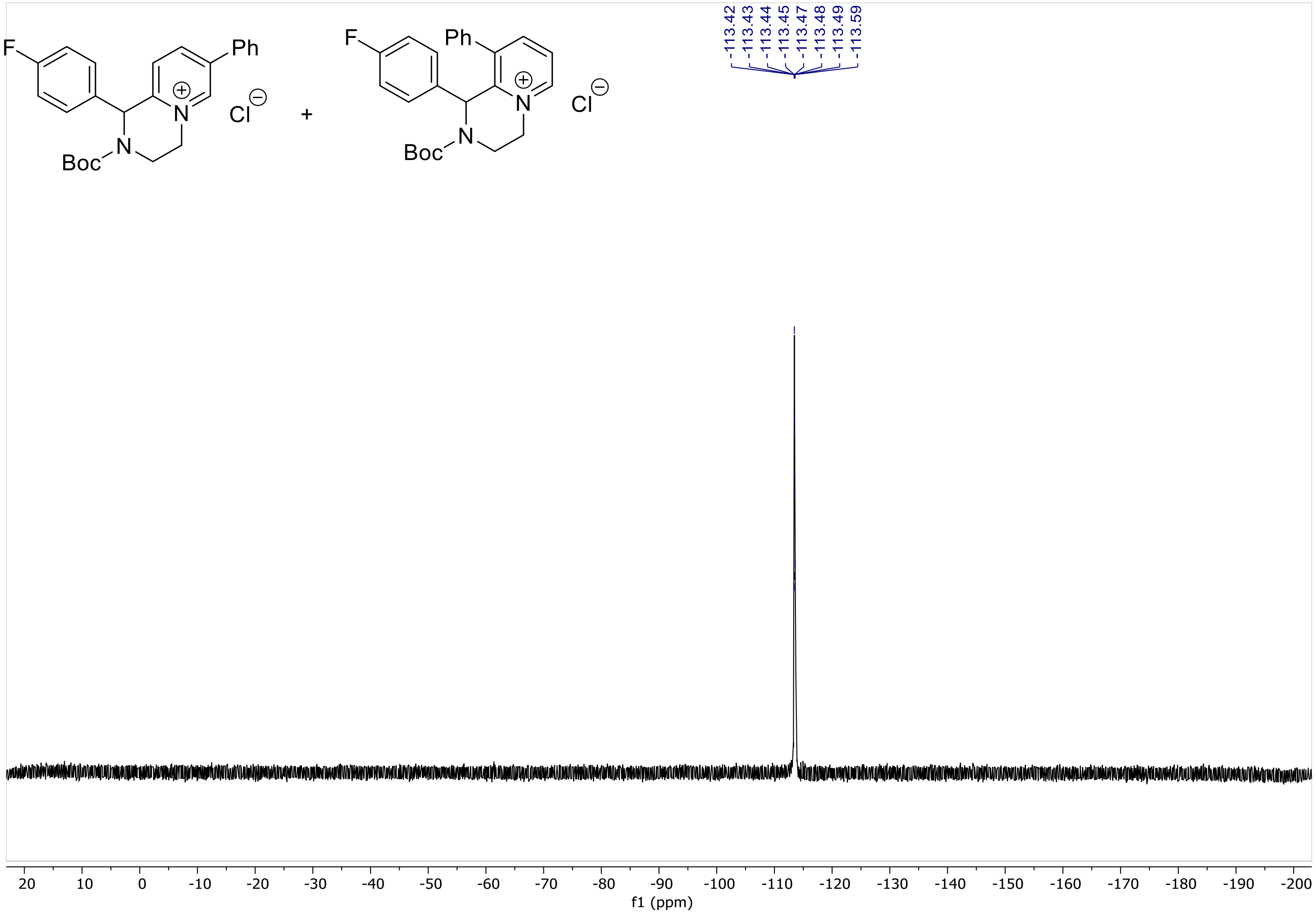

43: 1-Benzyl-2-phenylpyrimidin-1-ium chloride – ^{1}H NMR (DMSO-d_6)

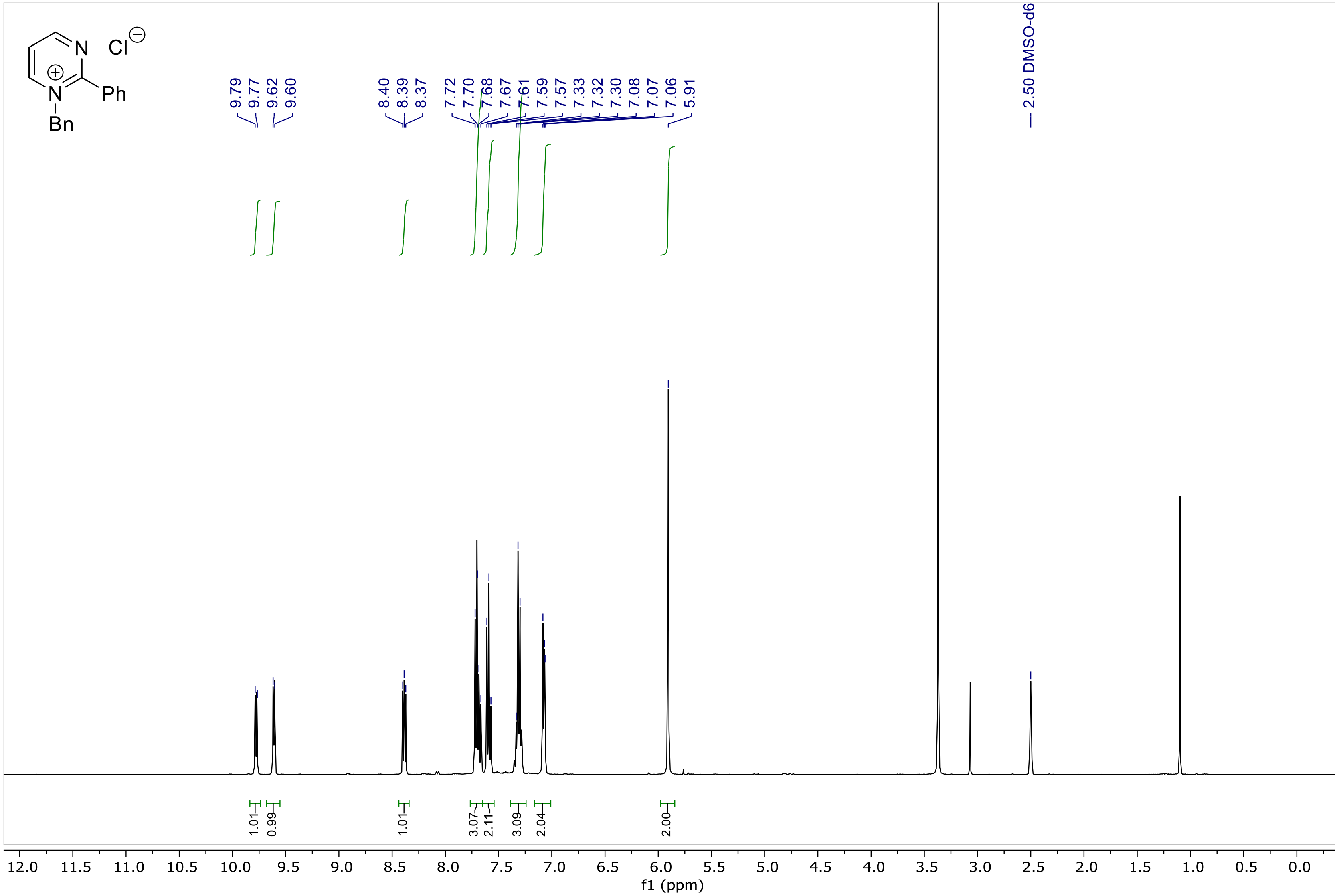

43: 1-Benzyl-2-phenylpyrimidin-1-ium chloride – $^{13}C\{^{1}H\}$ NMR (DMSO-d$_6$)

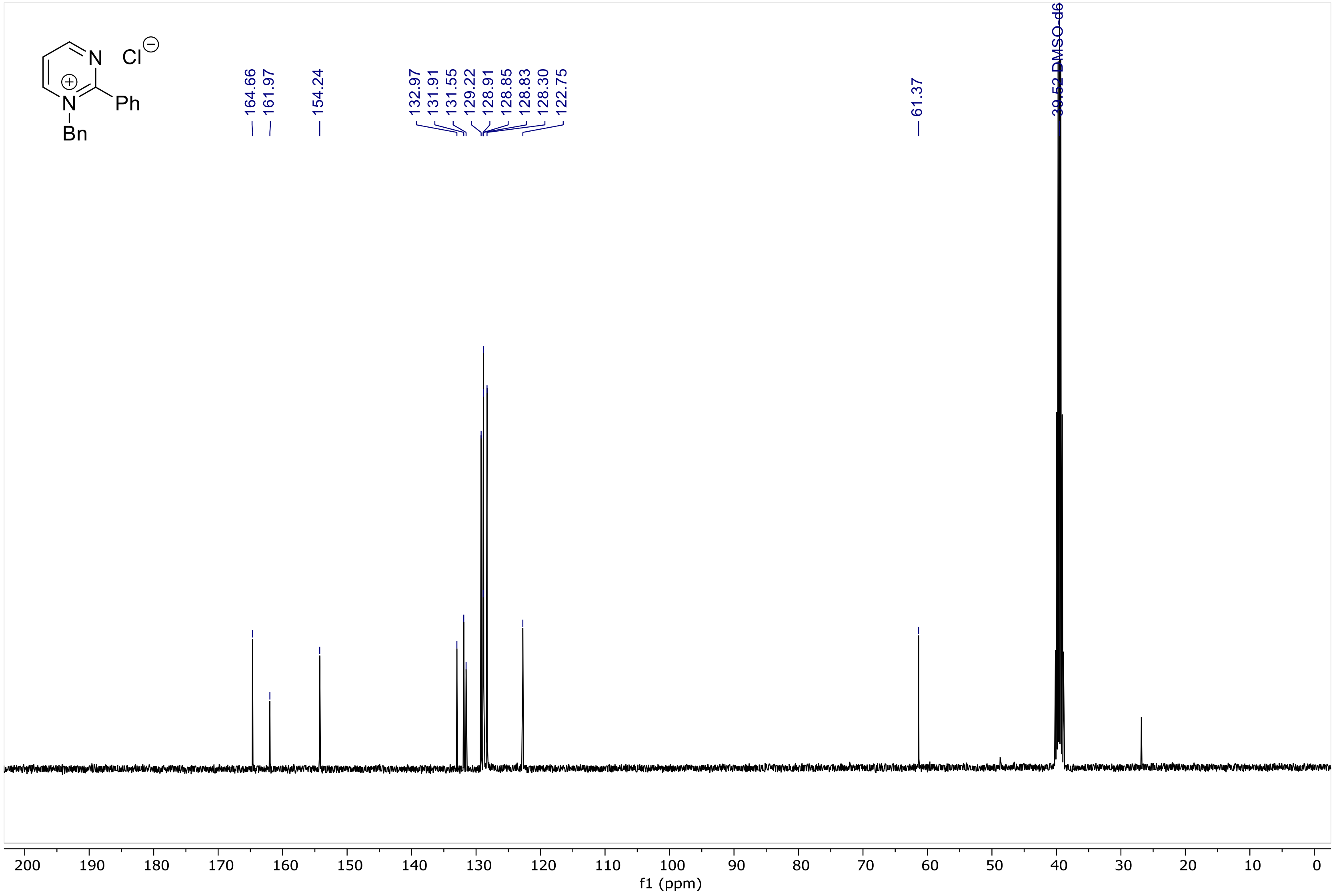

44: 1-Benzyl-2-phenyl-4,6-dimethylpyrimidin-1-ium chloride – ^{1}H NMR (CD_3OD)

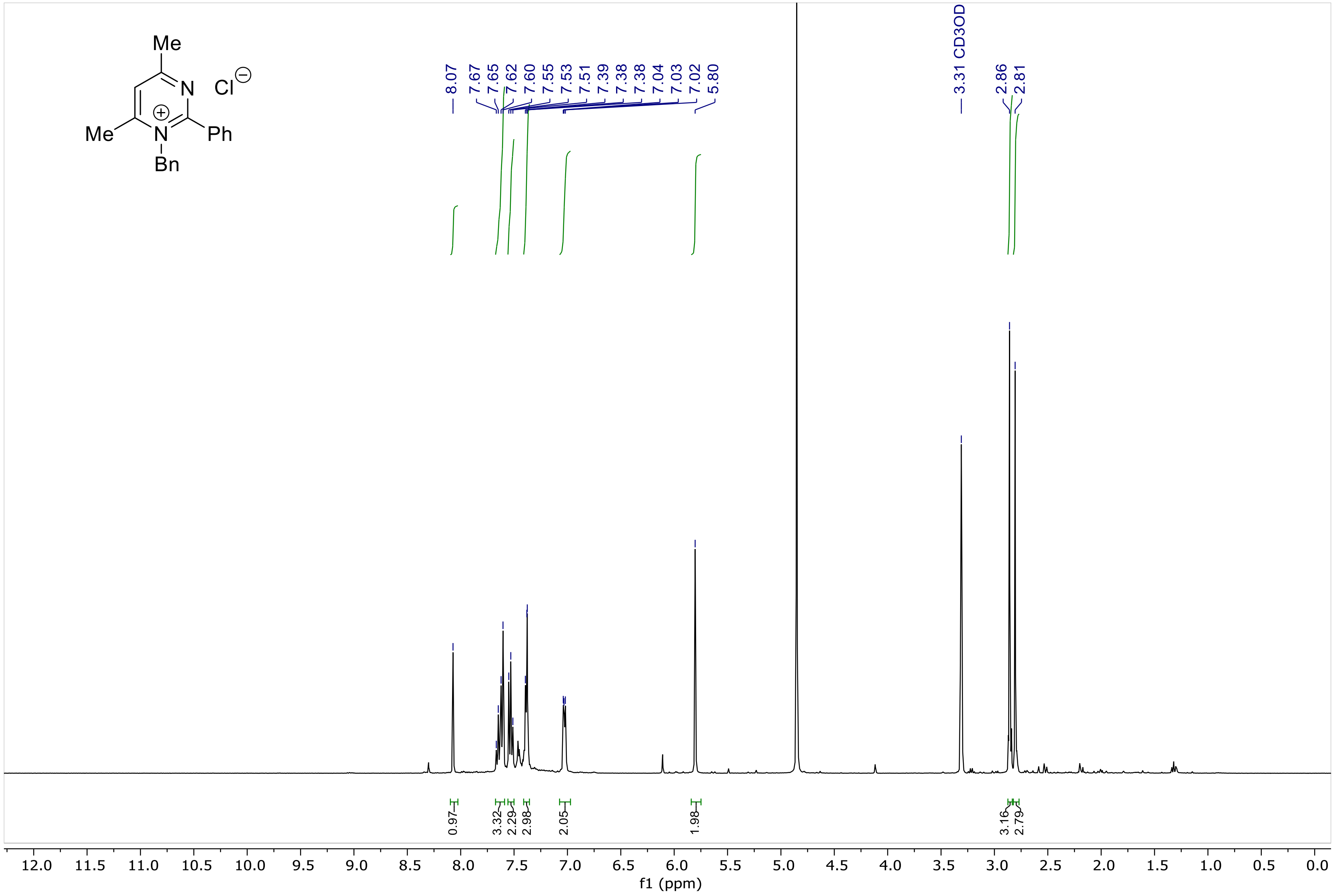

44: 1-Benzyl-2-phenyl-4,6-dimethylpyrimidin-1-ium chloride – $^{13}C\{^1H\}$ NMR (CD_3OD)

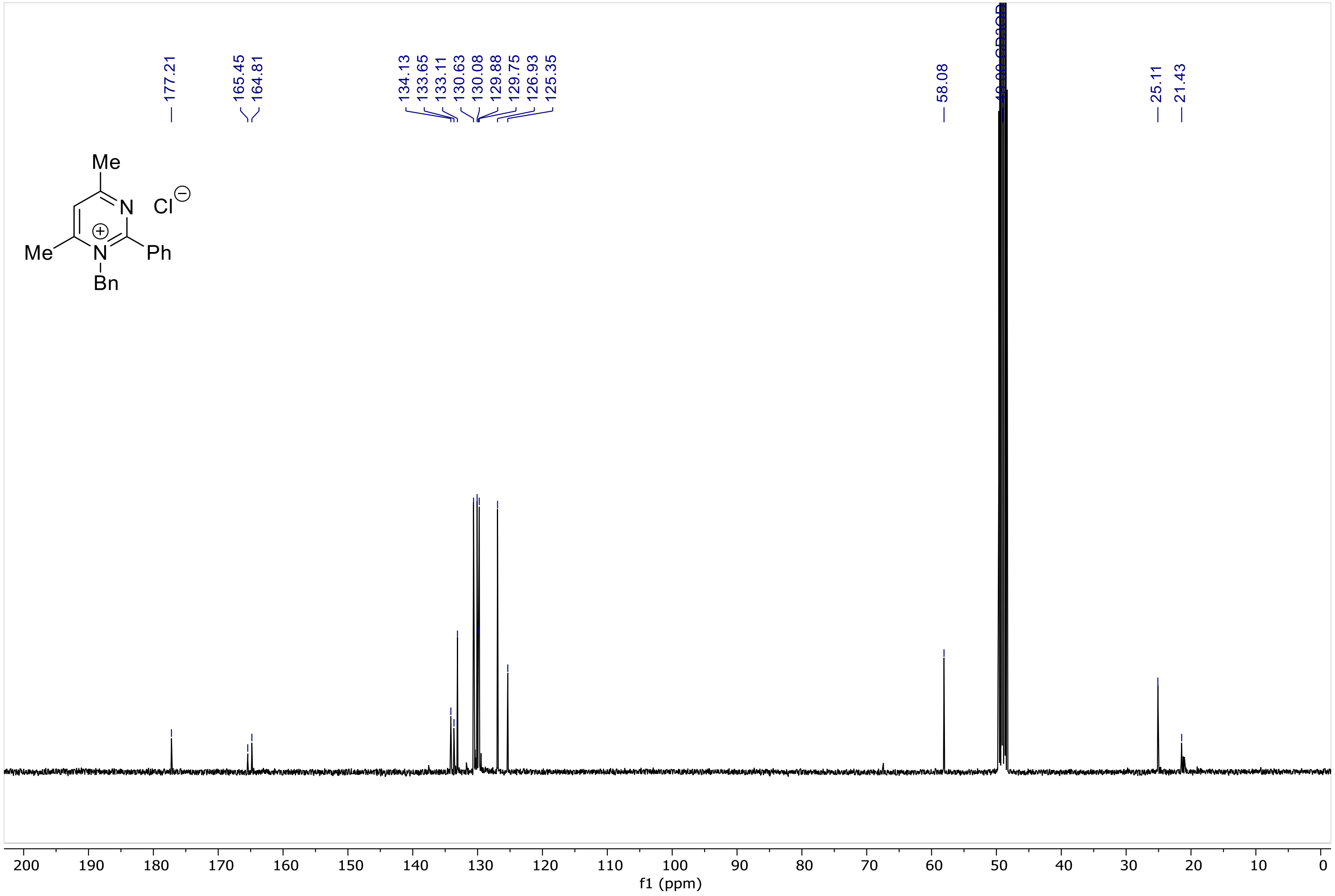

45: 1-Benzyl-2-phenyl-4,5,6-trimethylpyrimidin-1-ium chloride – ^{1}H NMR (DMSO-d$_6$)

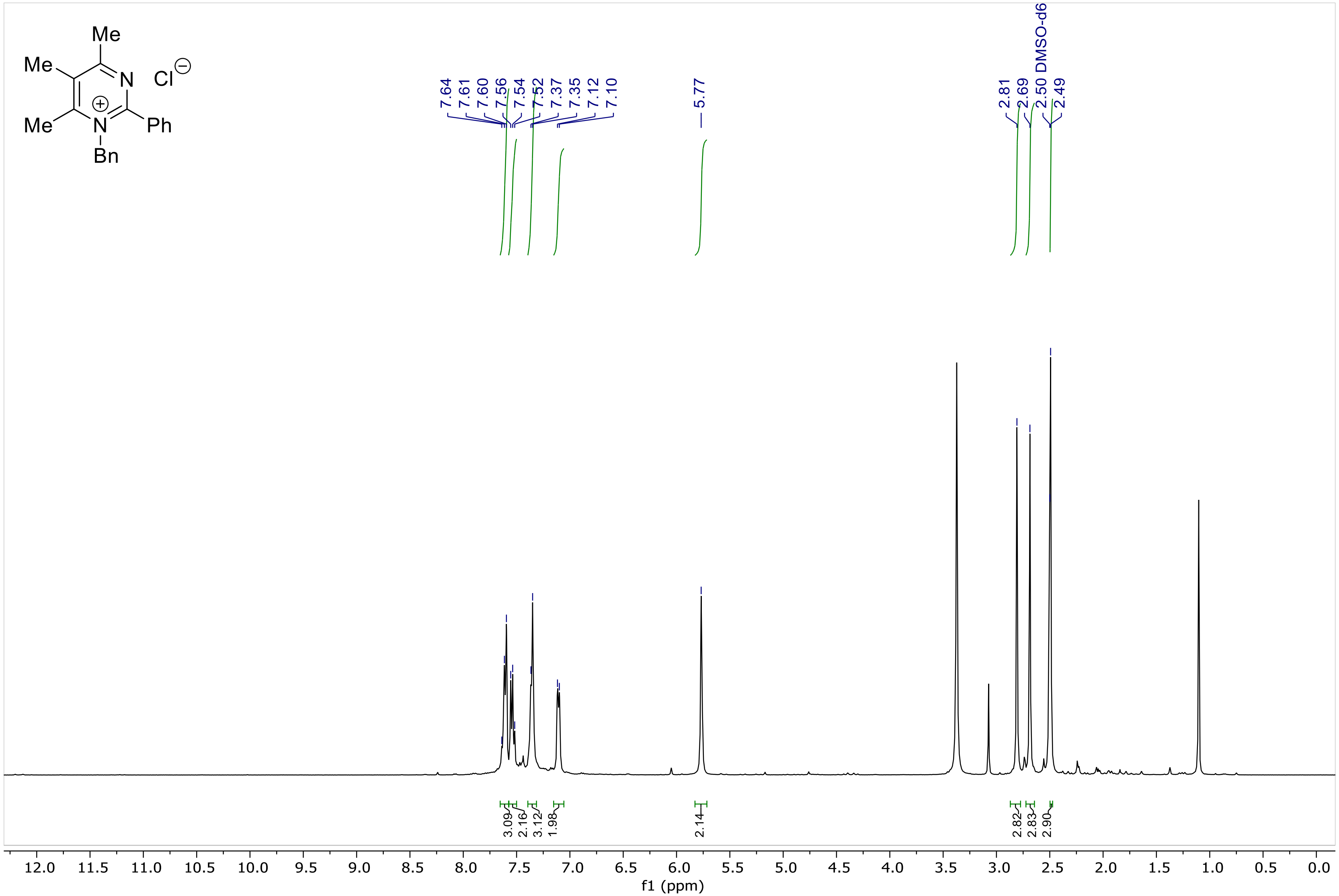

45: 1-Benzyl-2-phenyl-4,5,6-trimethylpyrimidin-1-ium chloride – $^{13}C\{^{1}H\}$ NMR (DMSO-d_6)

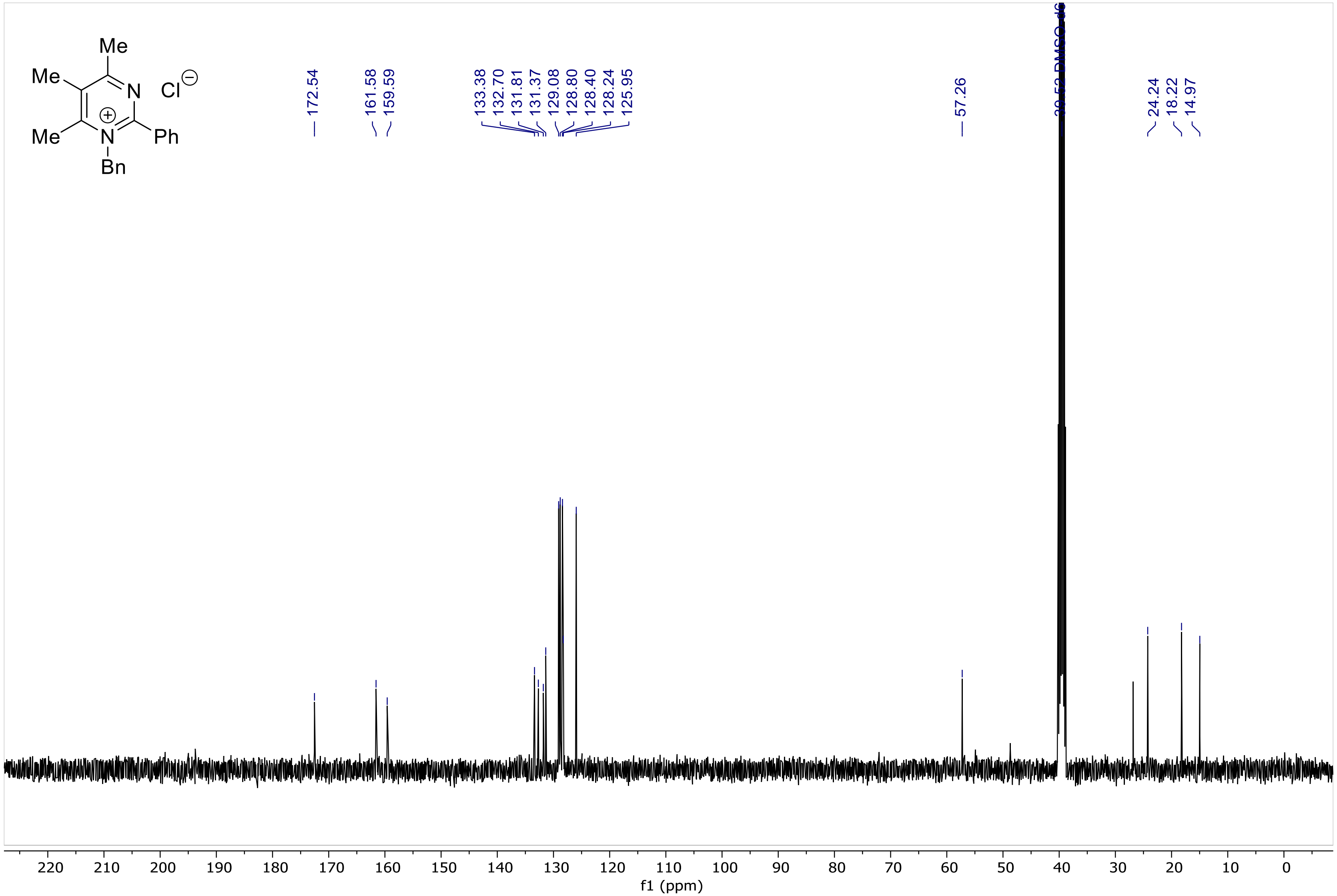

46: 1-Benzyl-2-phenyl-4,6-diethylpyrimidin-1-ium chloride – ^{1}H NMR (CD_3OD)

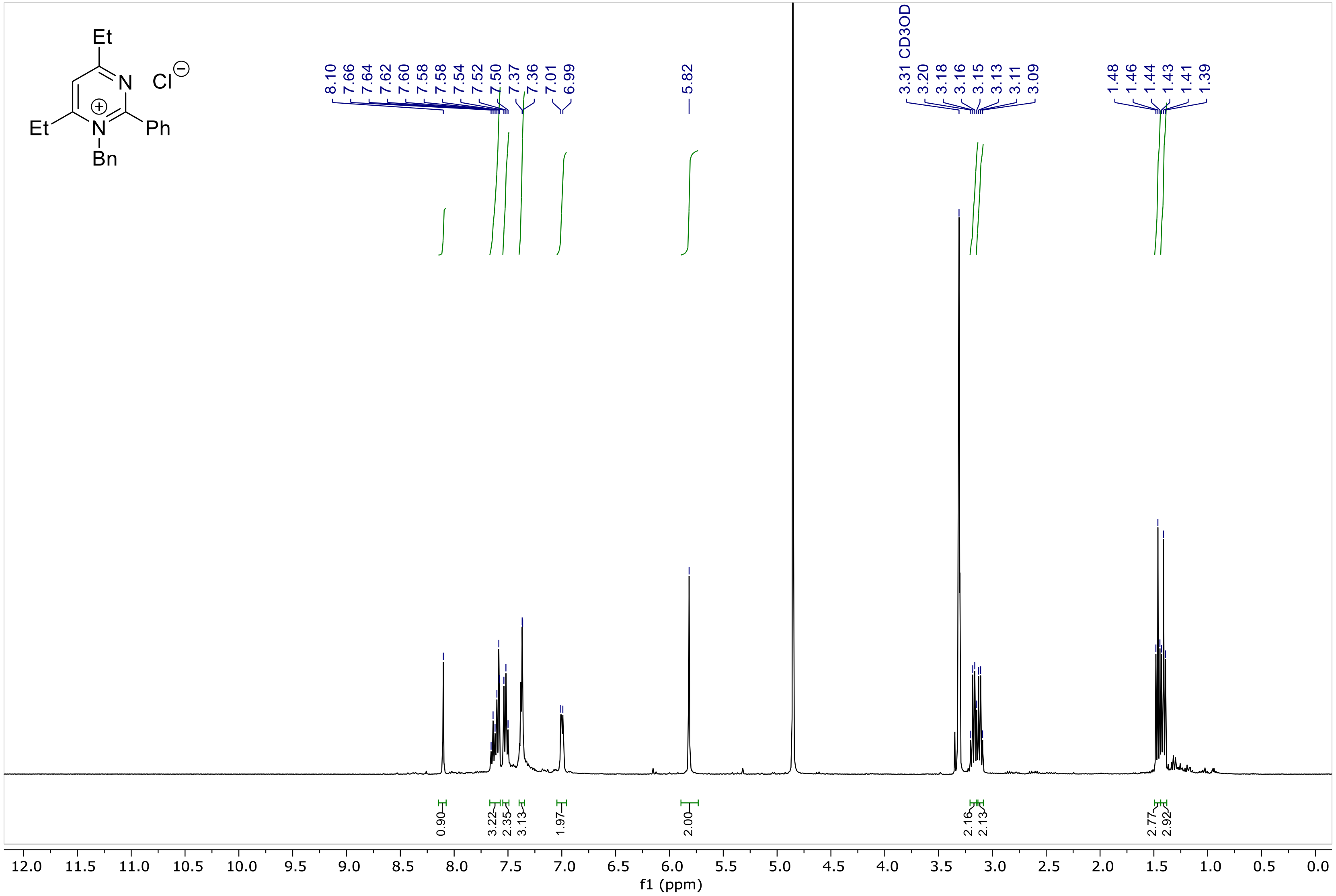

46: 1-Benzyl-2-phenyl-4,6-diethylpyrimidin-1-ium chloride – $^{13}C\{^{1}H\}$ NMR (CD_3OD)

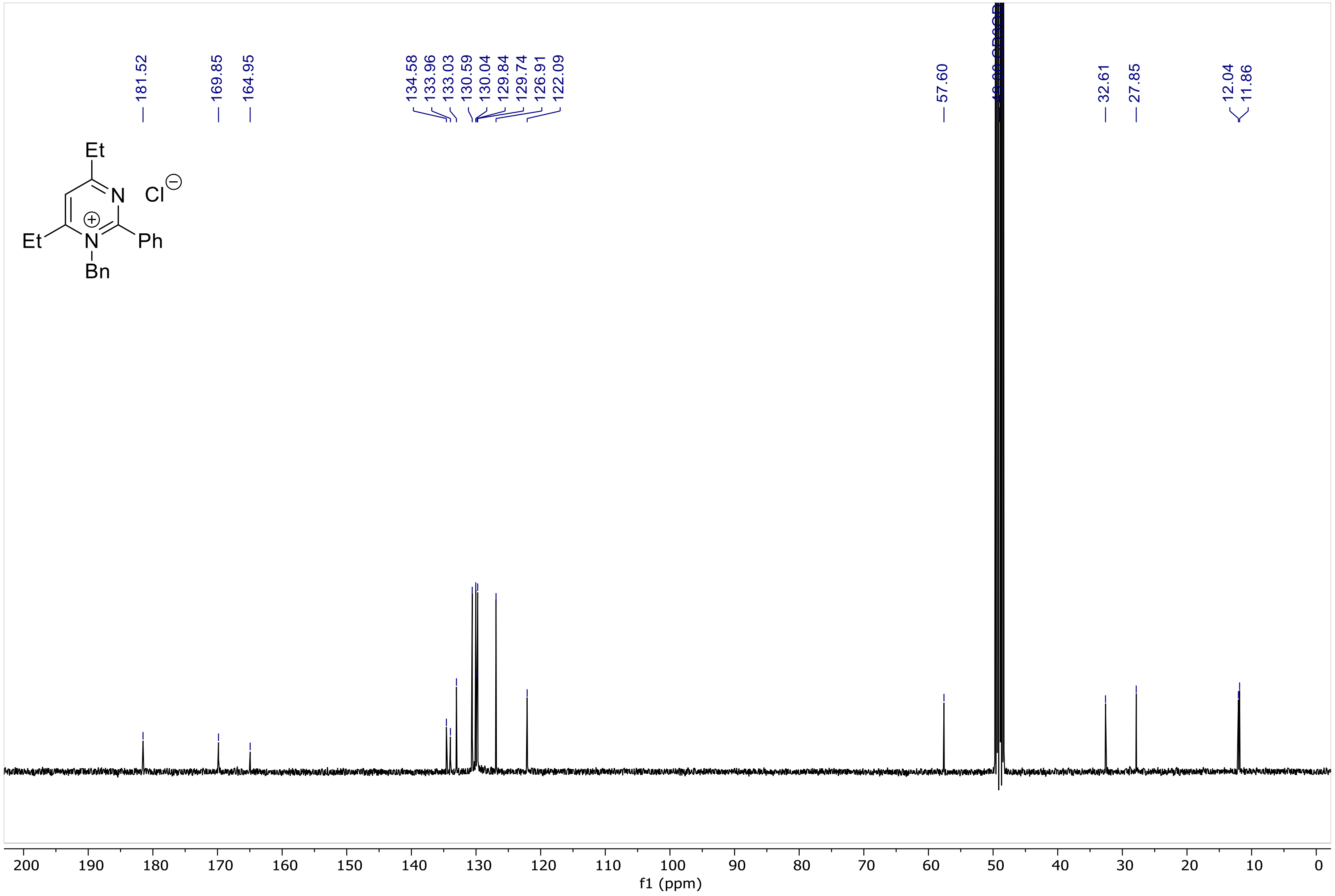

47: 1-Methyl-2-phenyl-5-iodopyrimidin-1-ium chloride – ^{1}H NMR (DMSO-d_6)

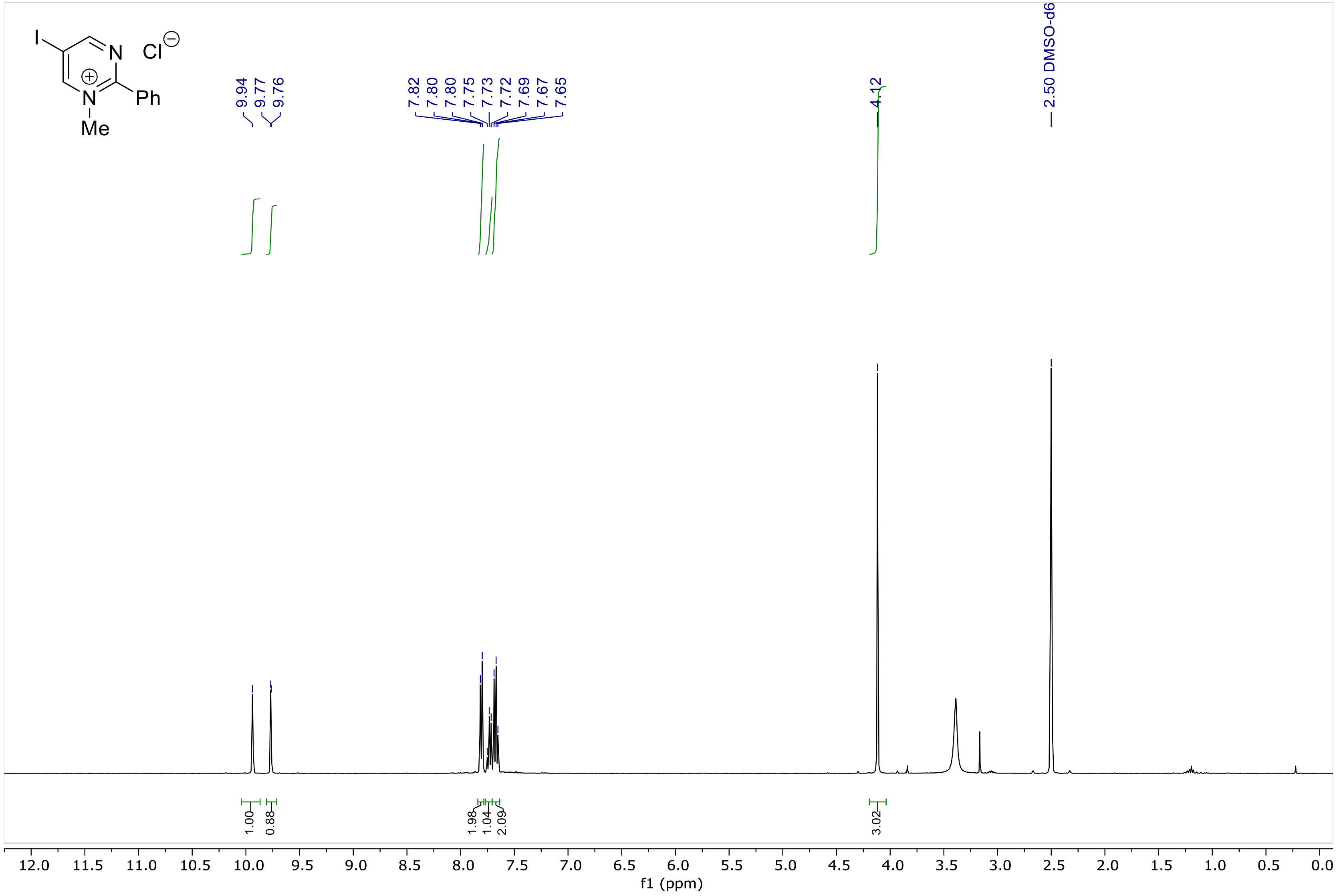

47: 1-Methyl-2-phenyl-5-iodopyrimidin-1-ium chloride – $^{13}C\{^1H\}$ NMR (DMSO-d$_6$)

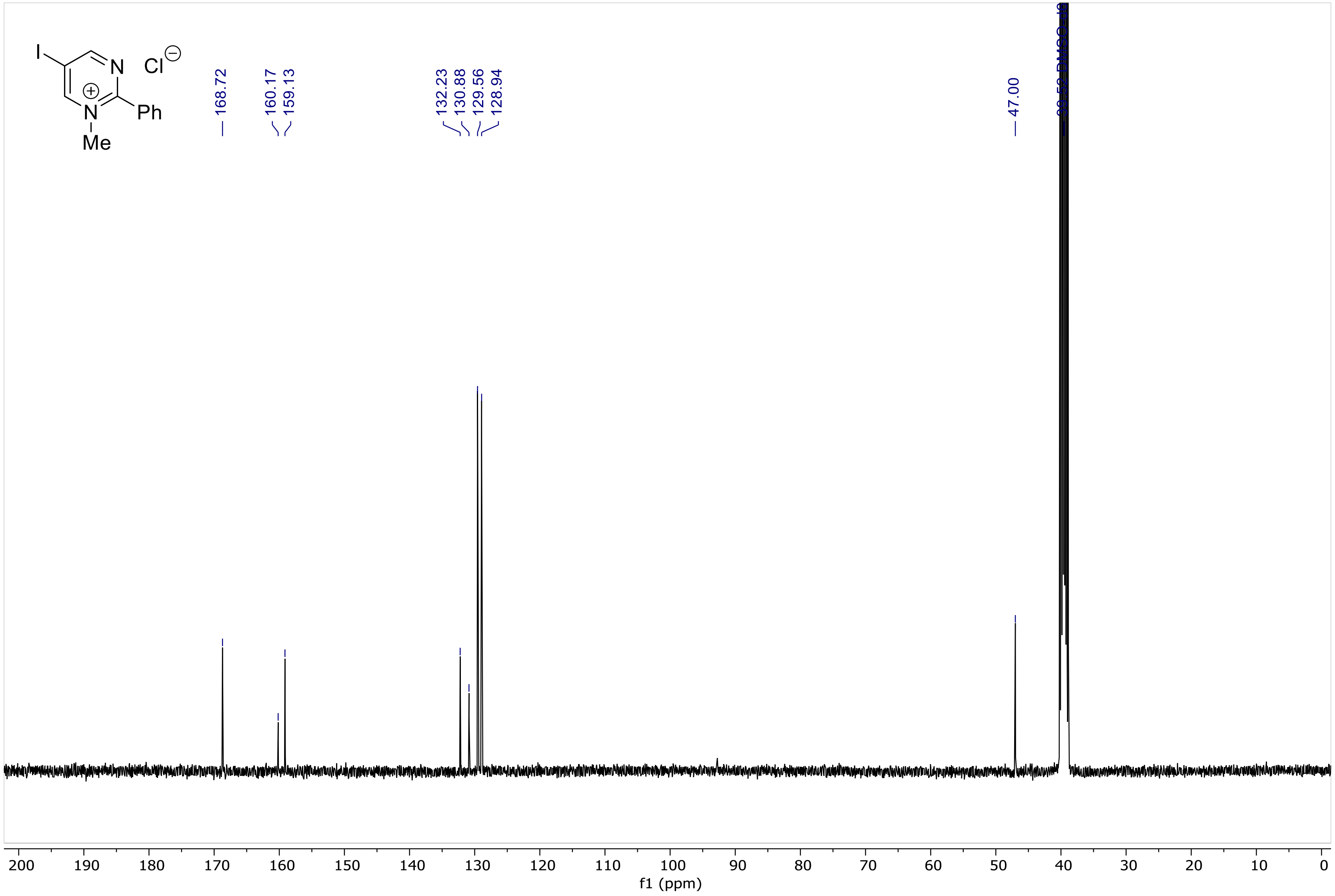

48: 1-Benzylindazole-diazirine adduct

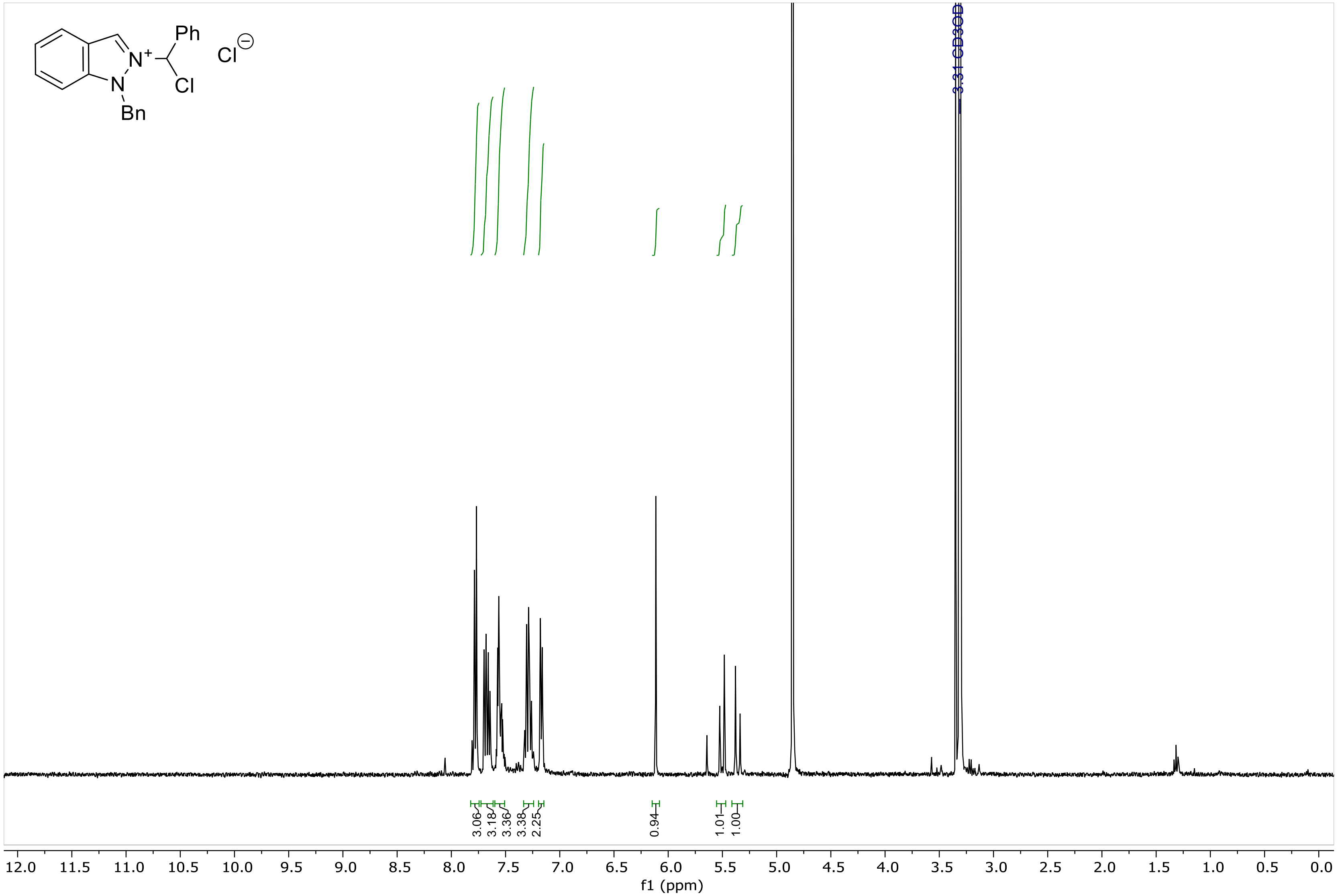

2: 1-Benzyl-5-fluoroindole – ^{1}H NMR (400 MHz, $CDCl_3$)

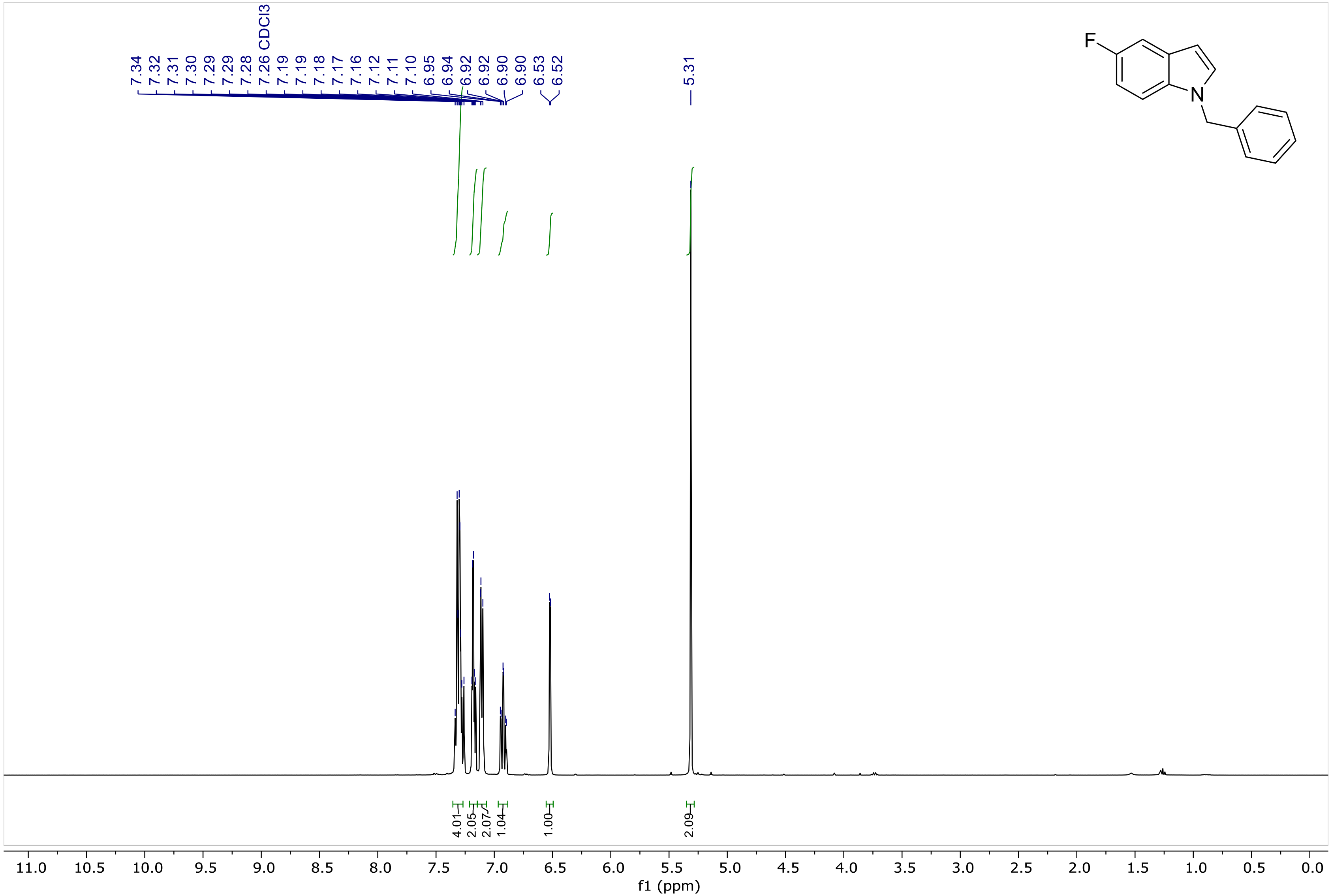

2: 1-Benzyl-5-fluoroindole – $^{13}C\{^1H\}$ NMR (101 MHz, $CDCl_3$)

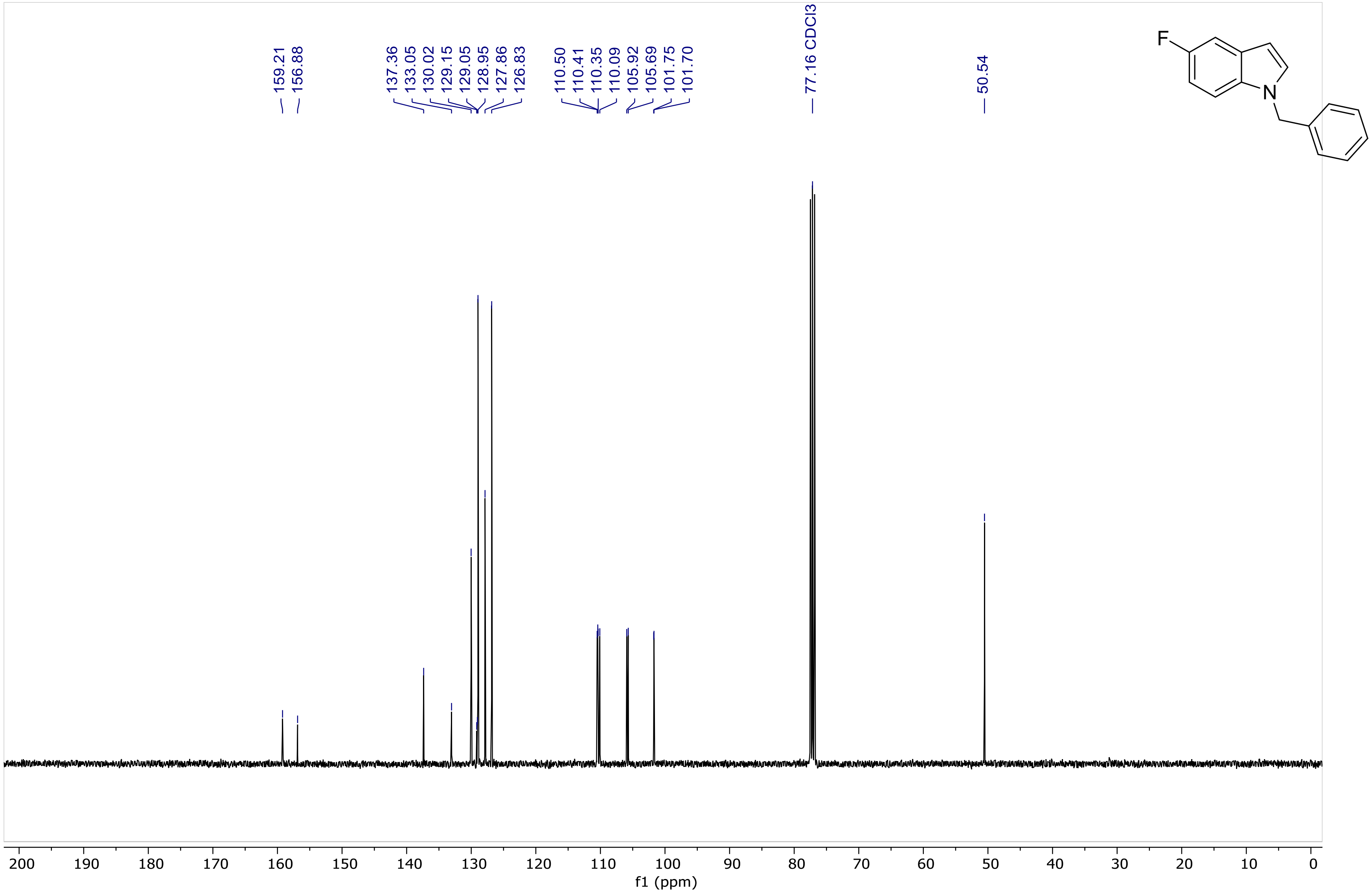

2: 1-Benzyl-5-fluoroindole – ^{19}F NMR (376 MHz, $CDCl_3$)

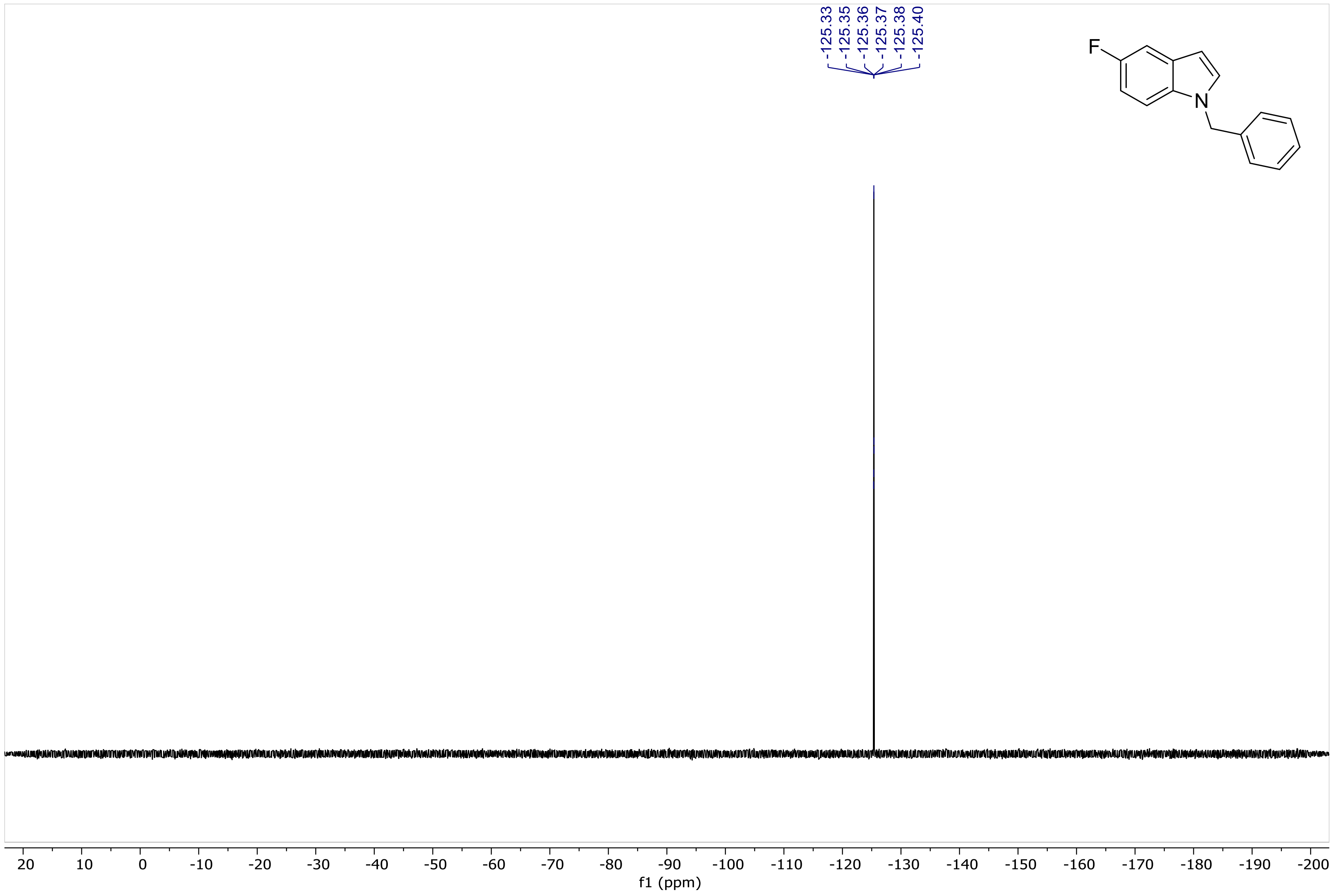

1-Benzyl-5-chloroindole – ^{1}H NMR (400 MHz, $CDCl_3$)

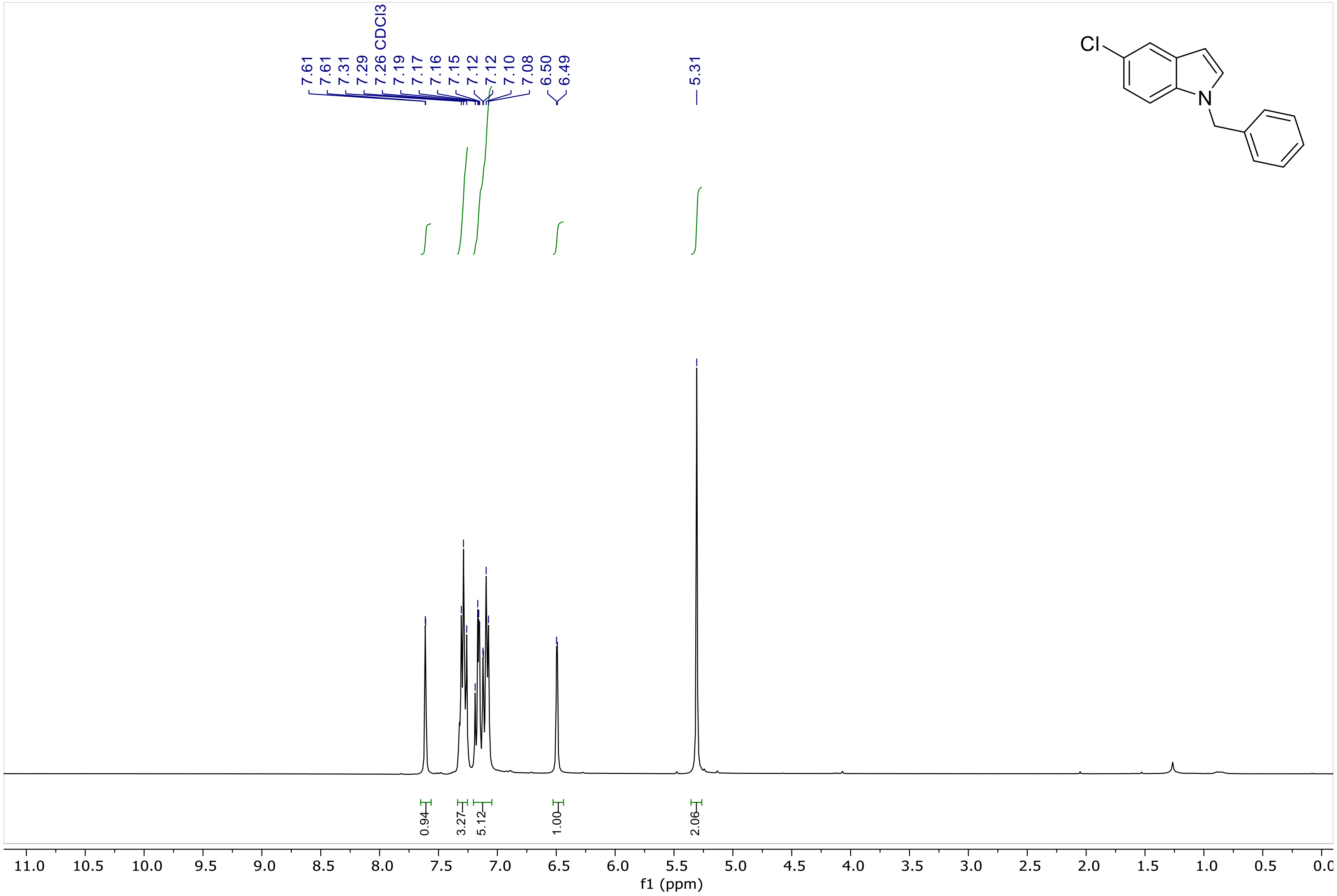

1-Benzyl-5-chloroindole – $^{13}C\{^1H\}$ NMR (101 MHz, $CDCl_3$)

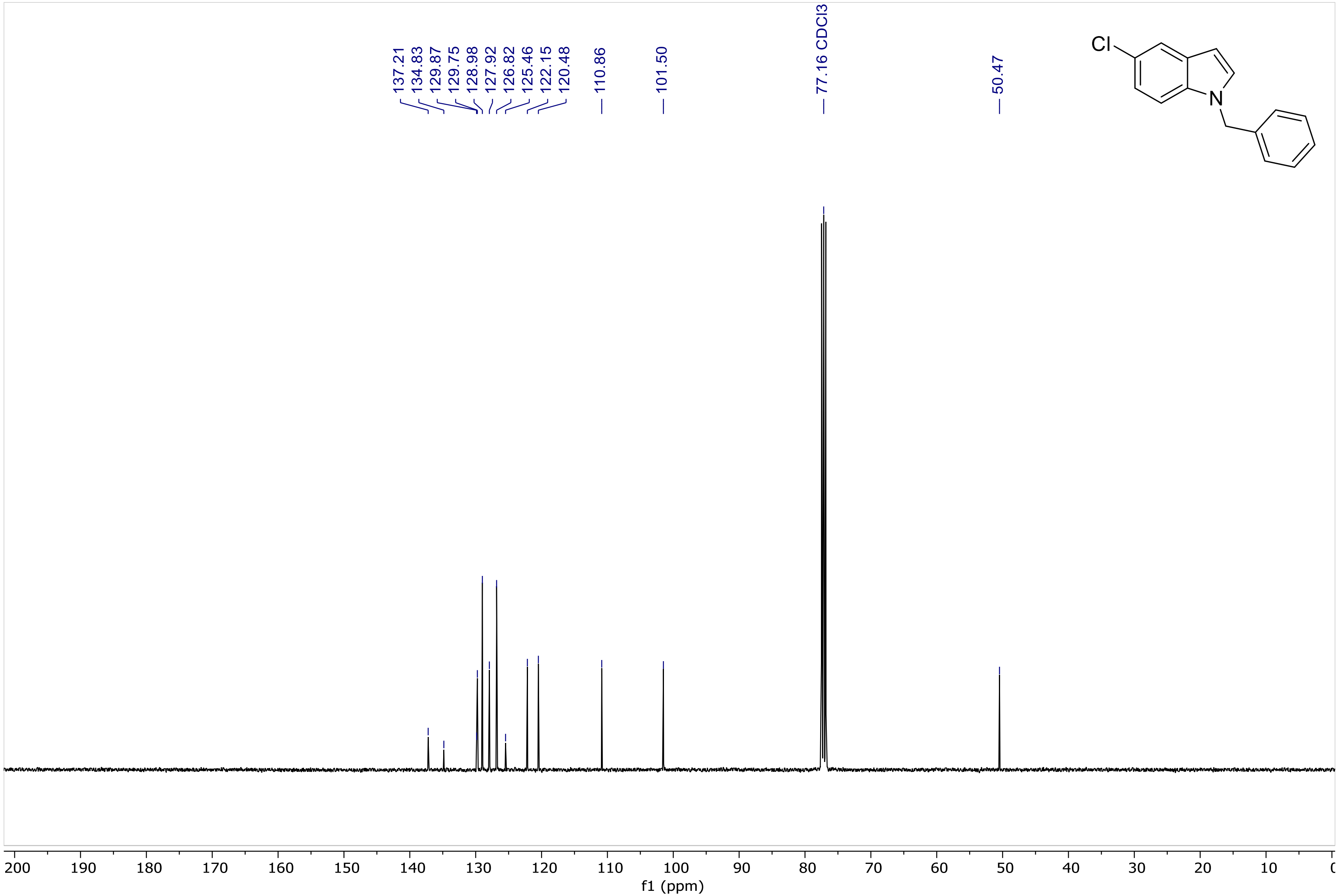

1-Benzyl-5-bromoindole – ^{1}H NMR (400 MHz, $CDCl_3$)

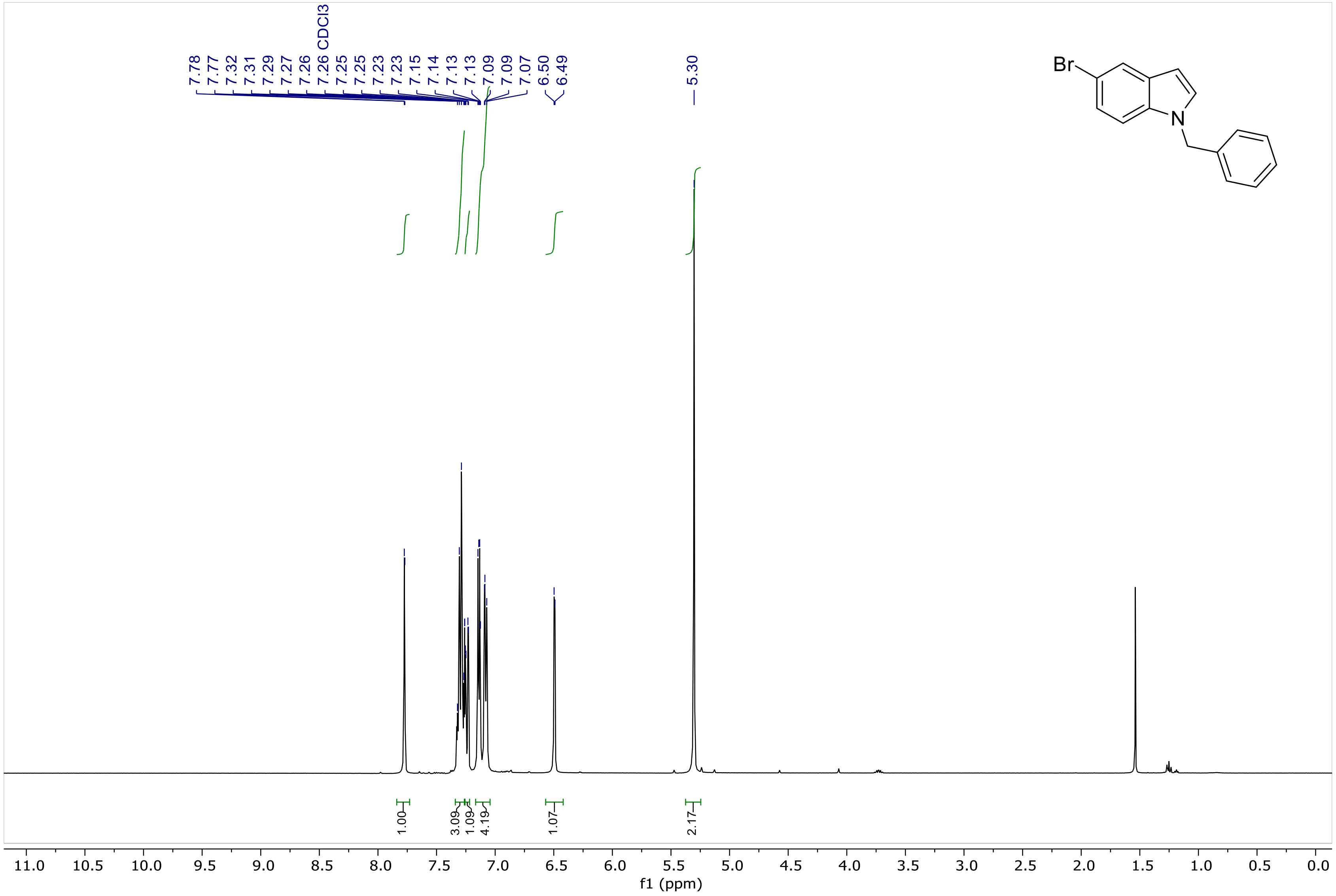

1-Benzyl-5-bromoindole – $^{13}C\{^{1}H\}$ NMR (101 MHz, $CDCl_3$)

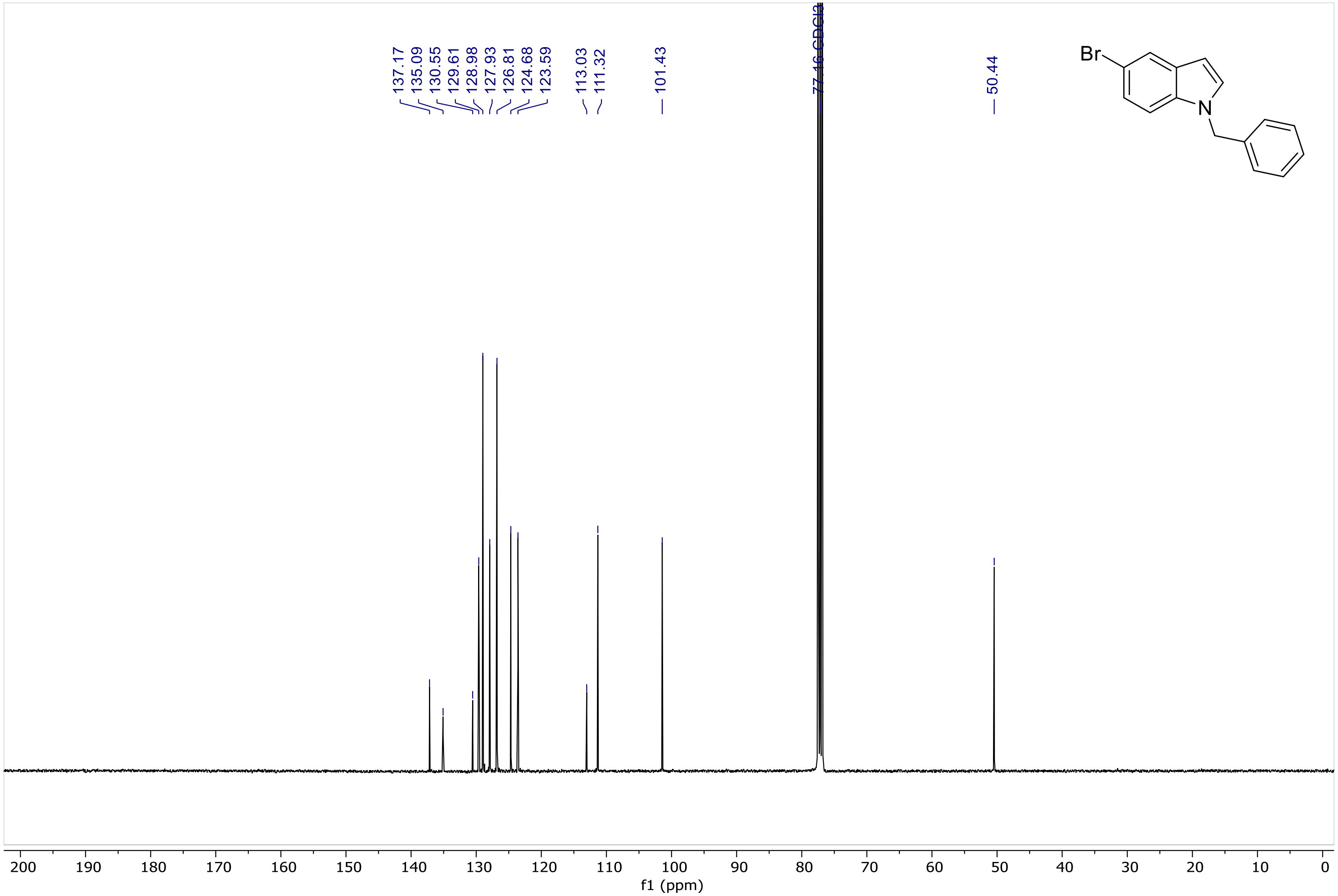

1-Benzyl-5-cyanoindole – ¹H NMR (400 MHz, CDCl₃)

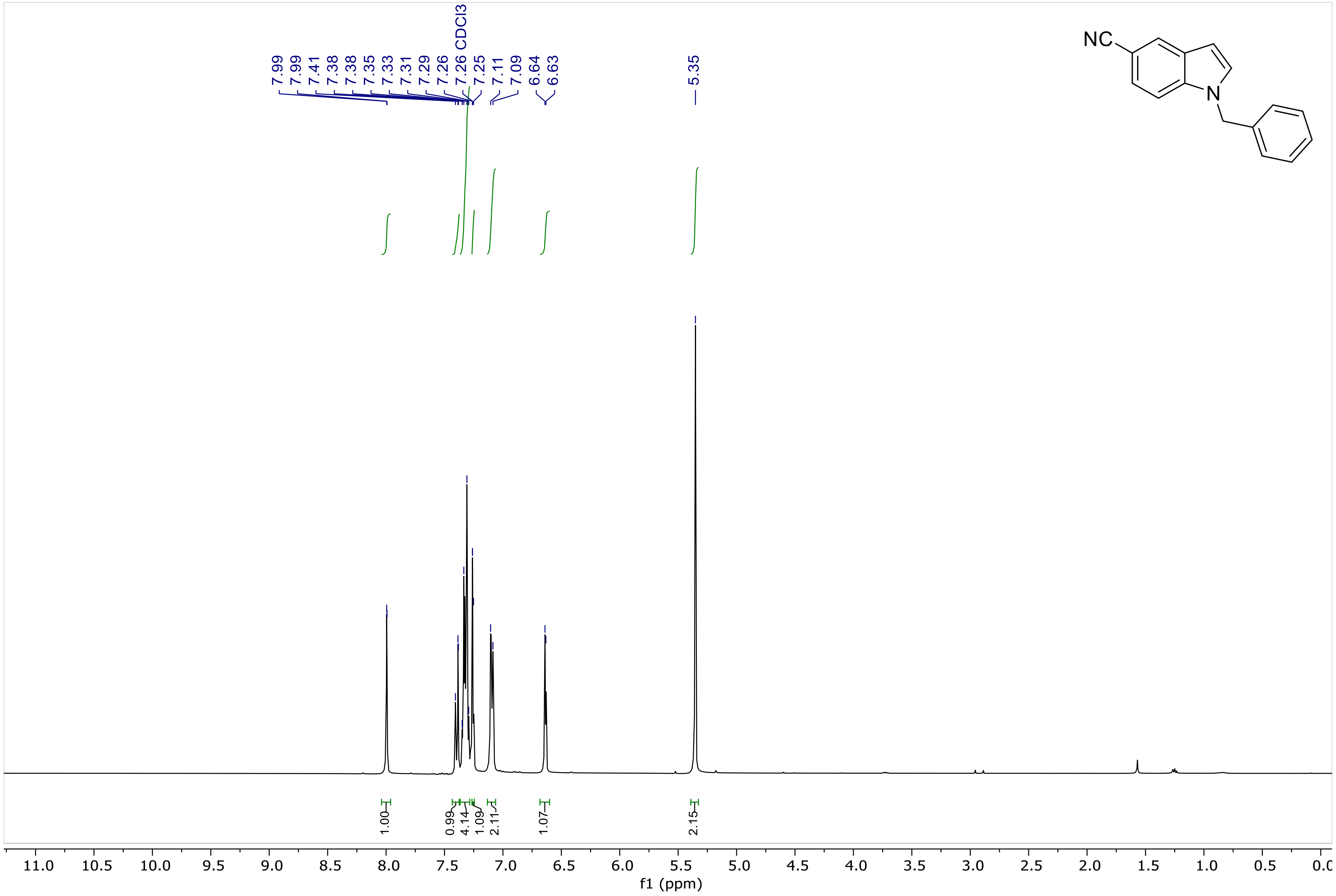

1-Benzyl-5-cyanoindole – $^{13}C\{^1H\}$ NMR (101 MHz, $CDCl_3$)

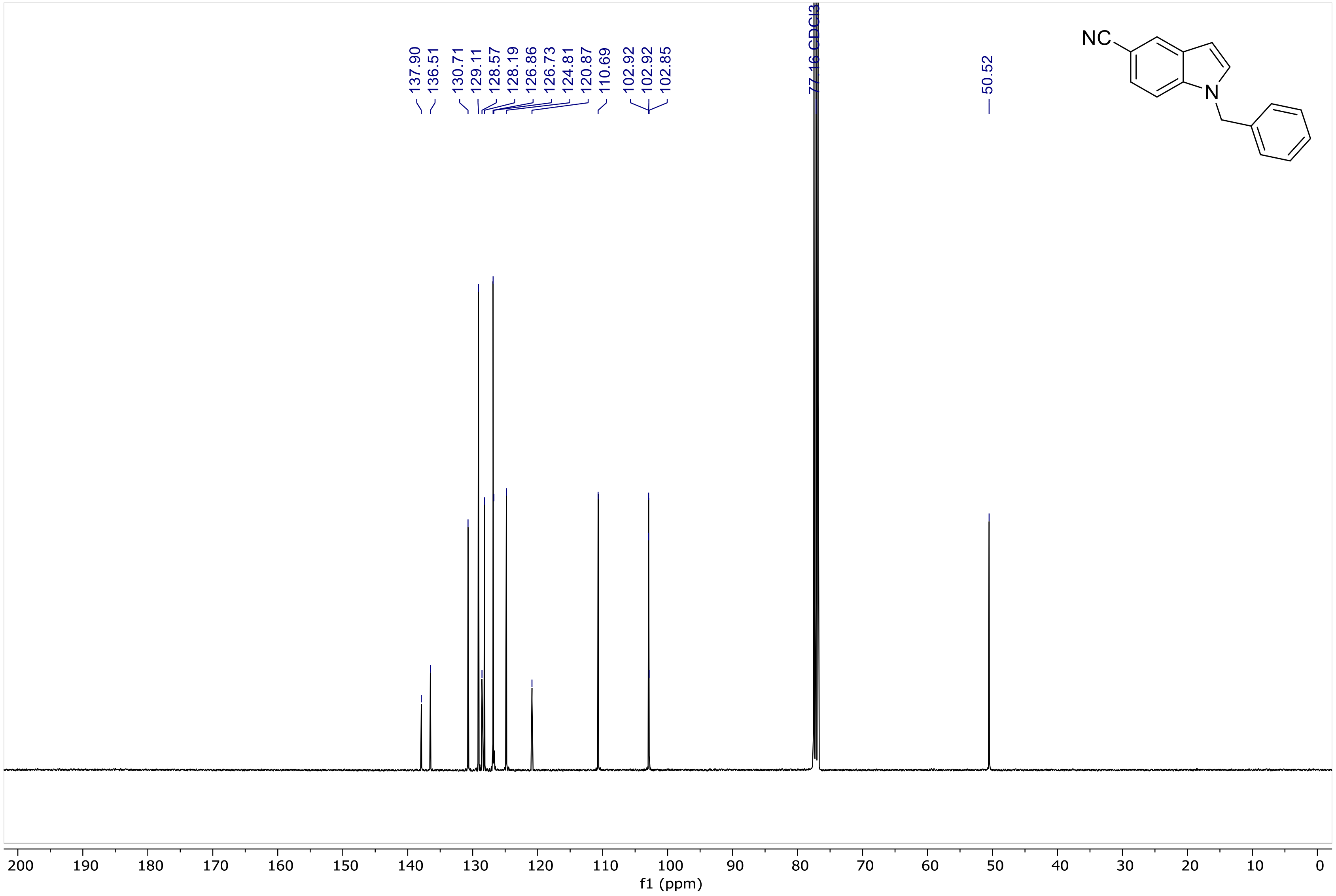

1-Benzyl-5-nitroindole – ^{1}H NMR (400 MHz, $CDCl_3$)

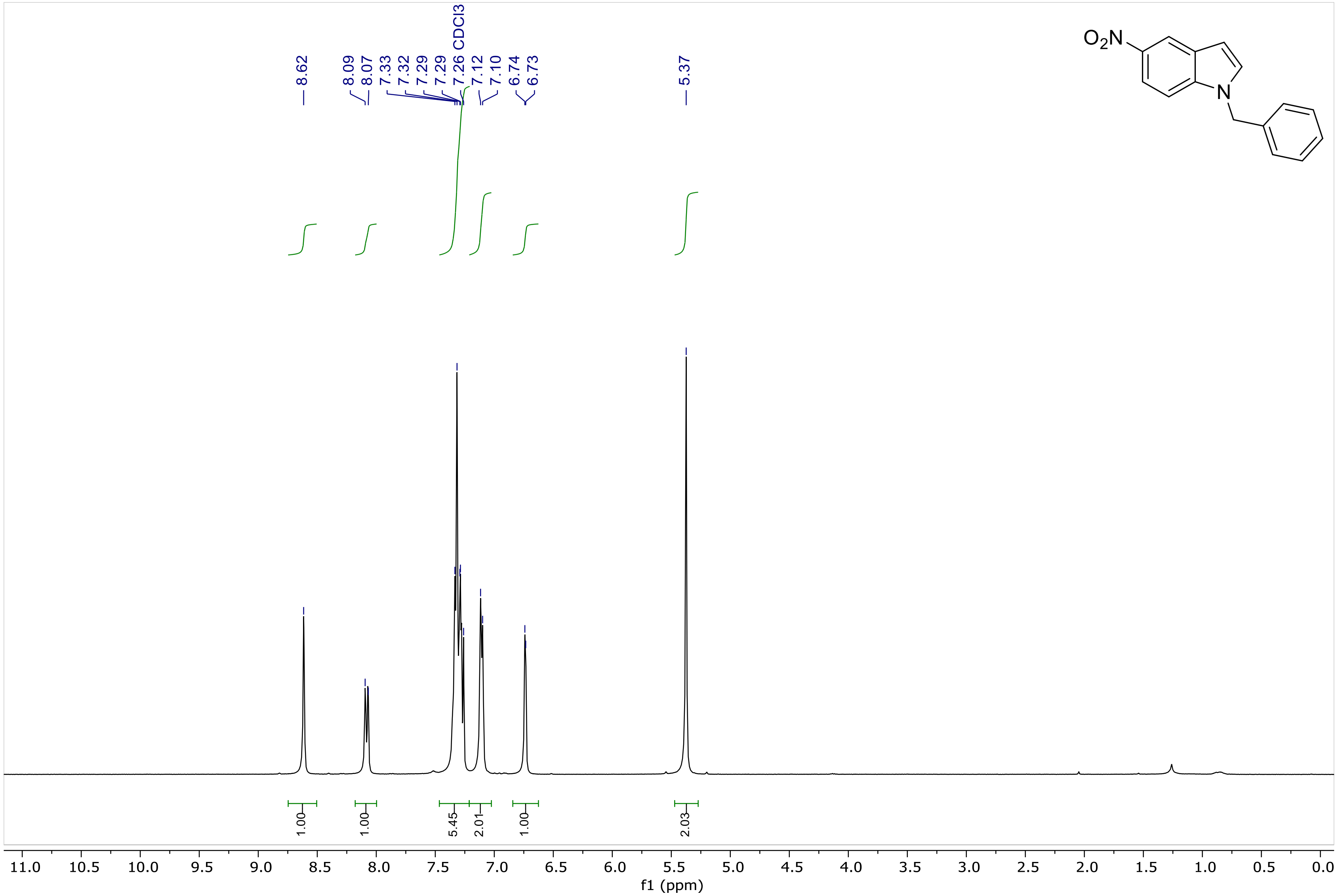

1-Benzyl-5-nitroindole – $^{13}C\{^1H\}$ NMR (101 MHz, $CDCl_3$)

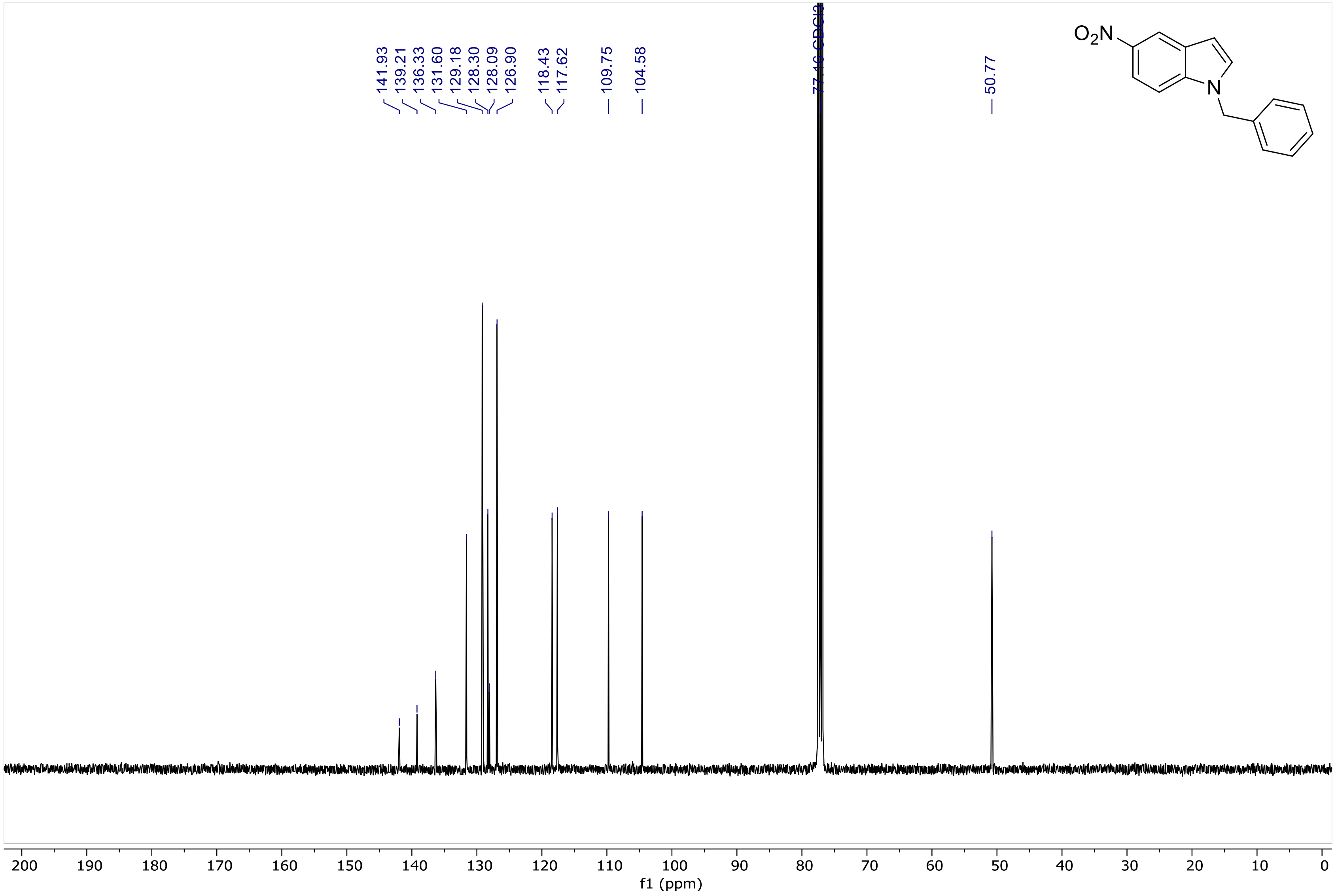

1-Benzylindole – ^{1}H NMR (400 MHz, $CDCl_3$)

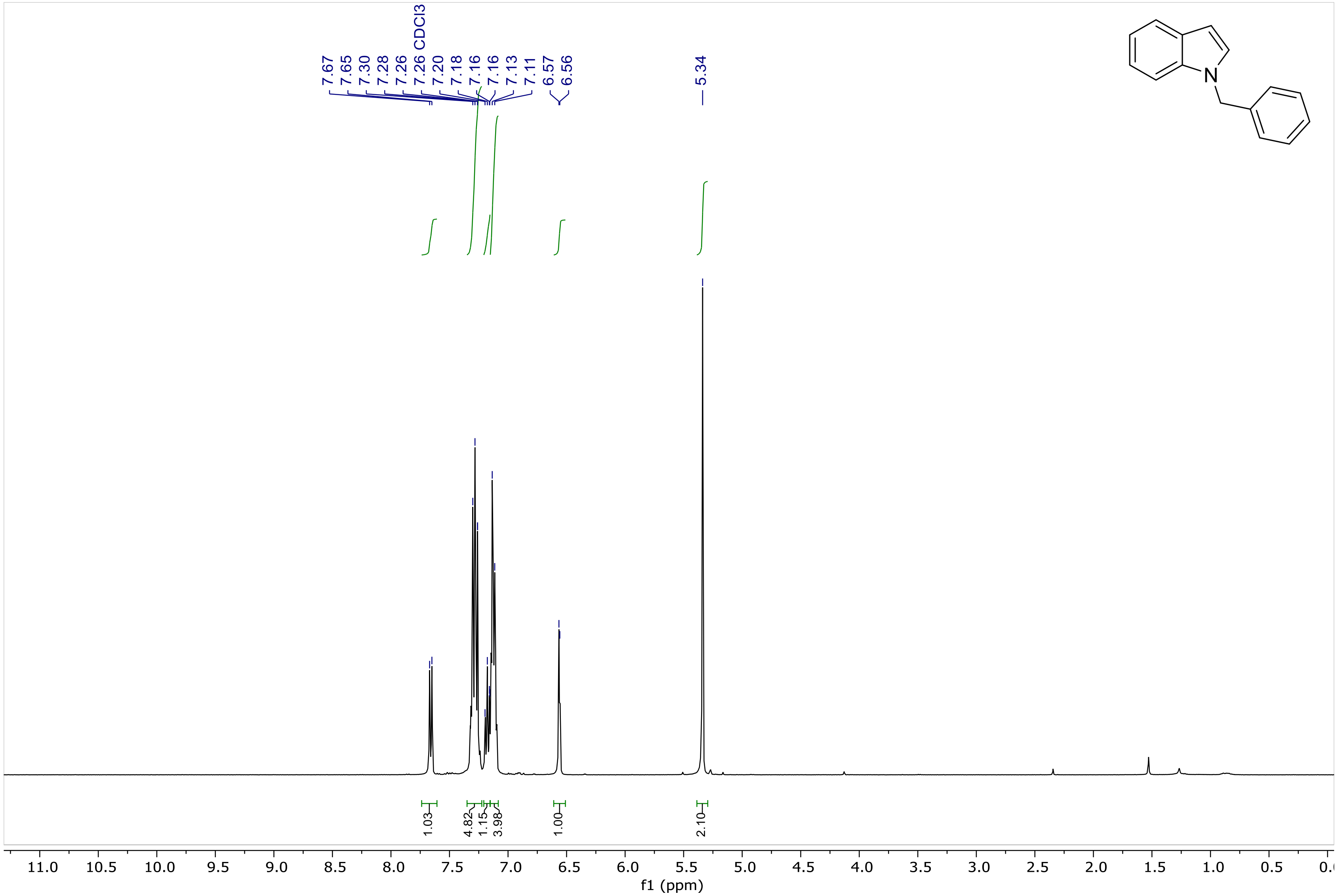

1-Benzylindole – $^{13}C\{^{1}H\}$ NMR (101 MHz, $CDCl_3$)

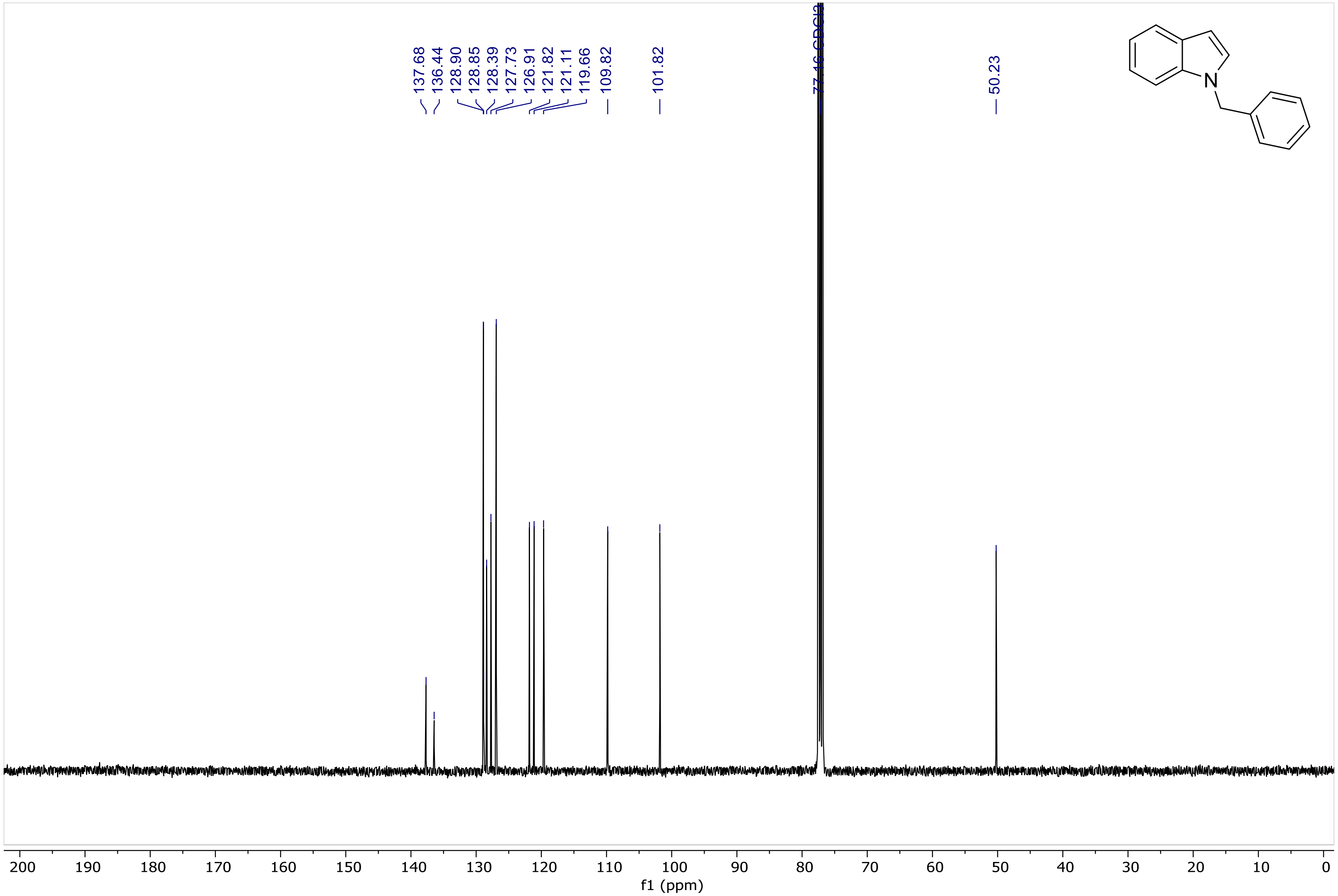

1-Benzyl-5-(benzyloxy)indole – ^{1}H NMR (400 MHz, $CDCl_3$)

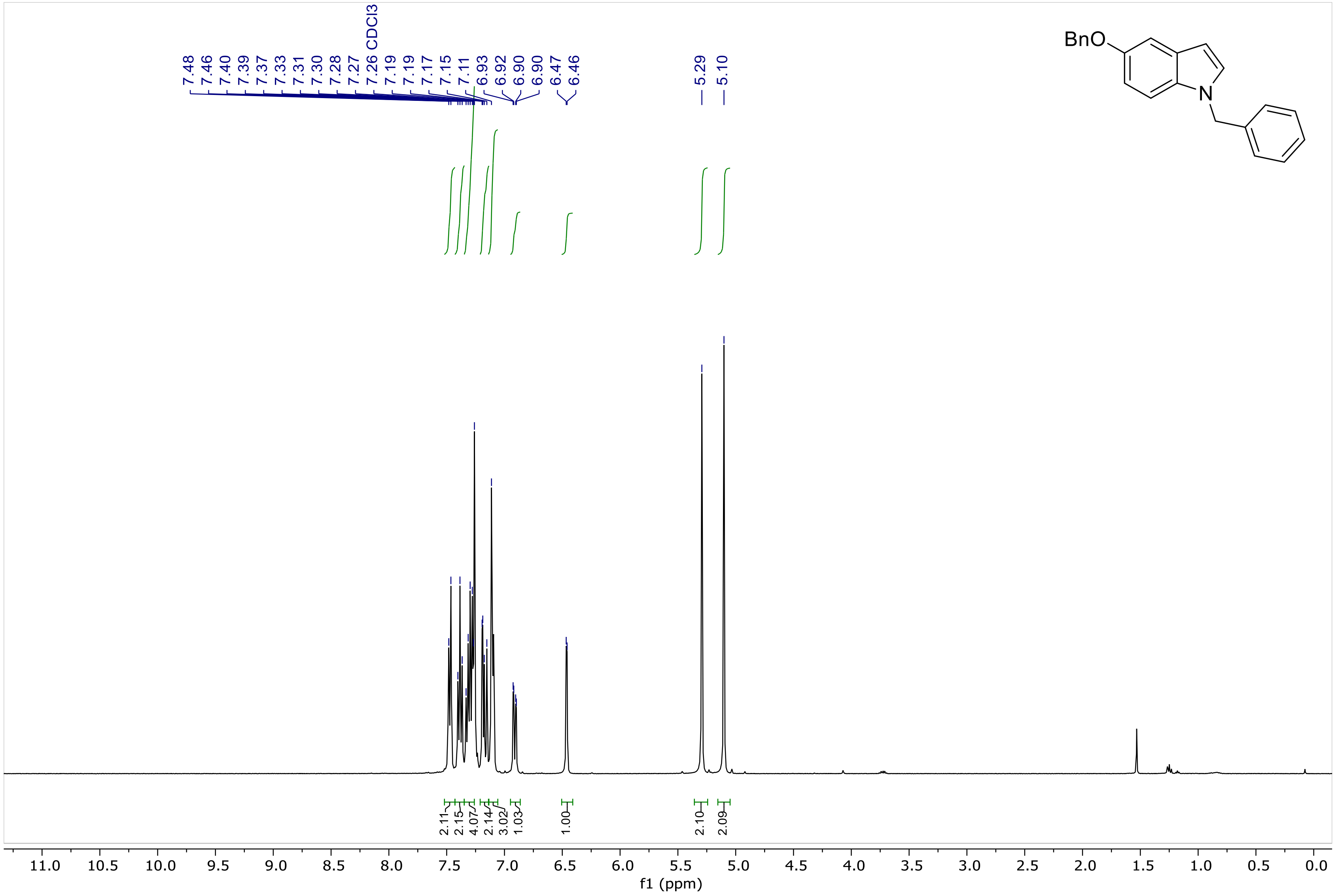

1-Benzyl-5-(benzyloxy)indole – $^{13}C\{^{1}H\}$ NMR (101 MHz, $CDCl_3$)

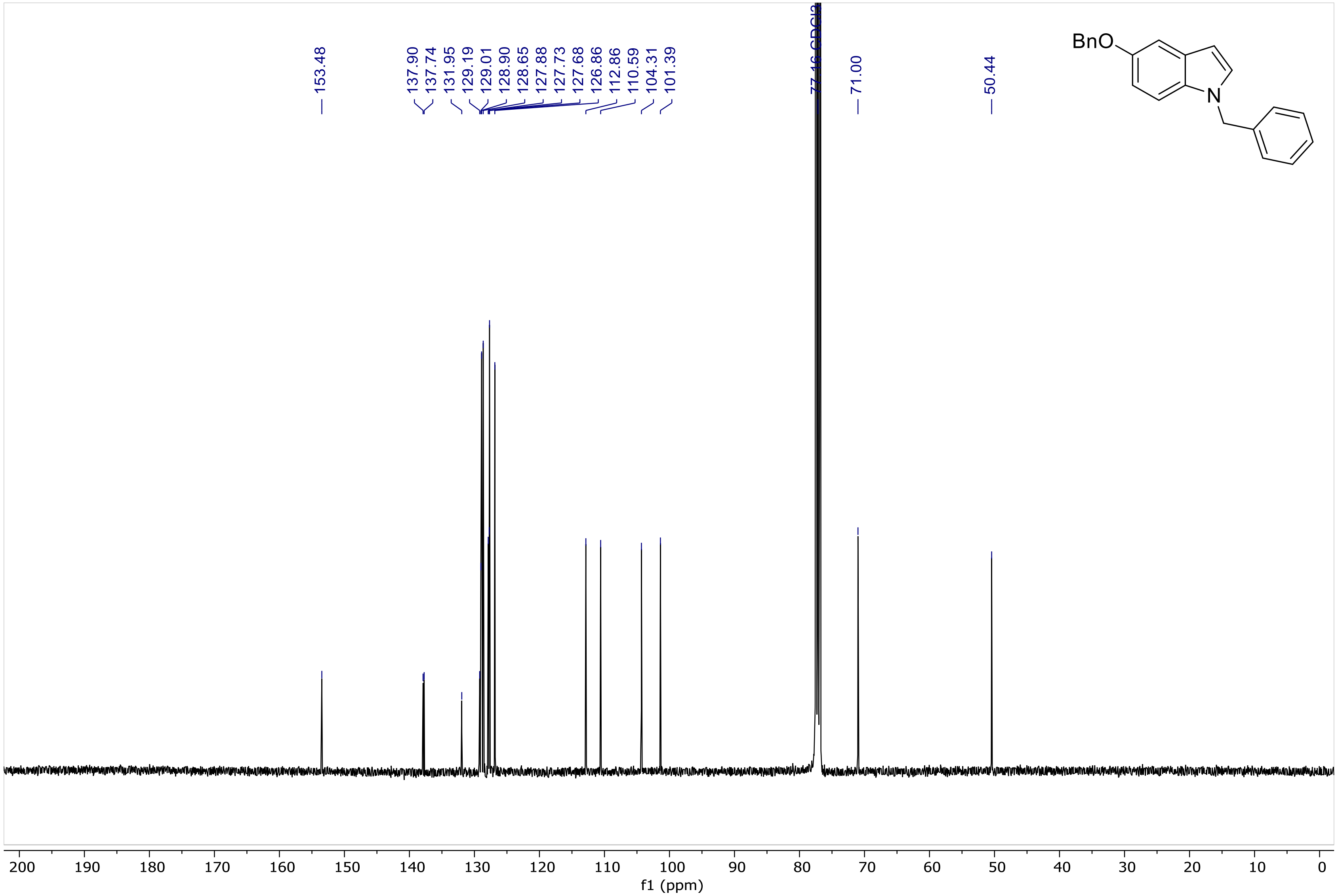

1-Benzyl-5-methoxylindole – ^{1}H NMR (400 MHz, $CDCl_3$)

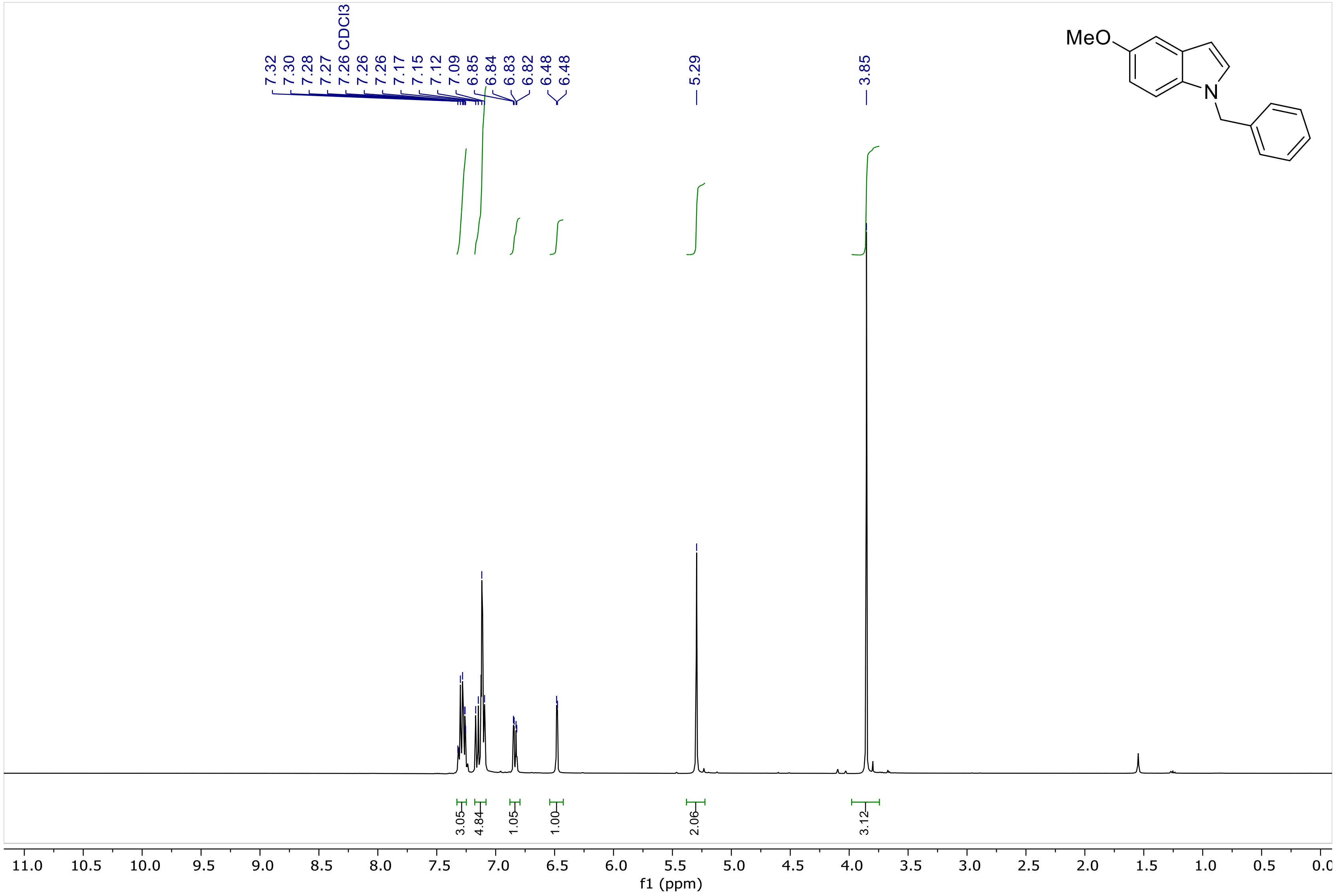

1-Benzyl-5-methoxylindole – $^{13}C\{^1H\}$ NMR (101 MHz, $CDCl_3$)

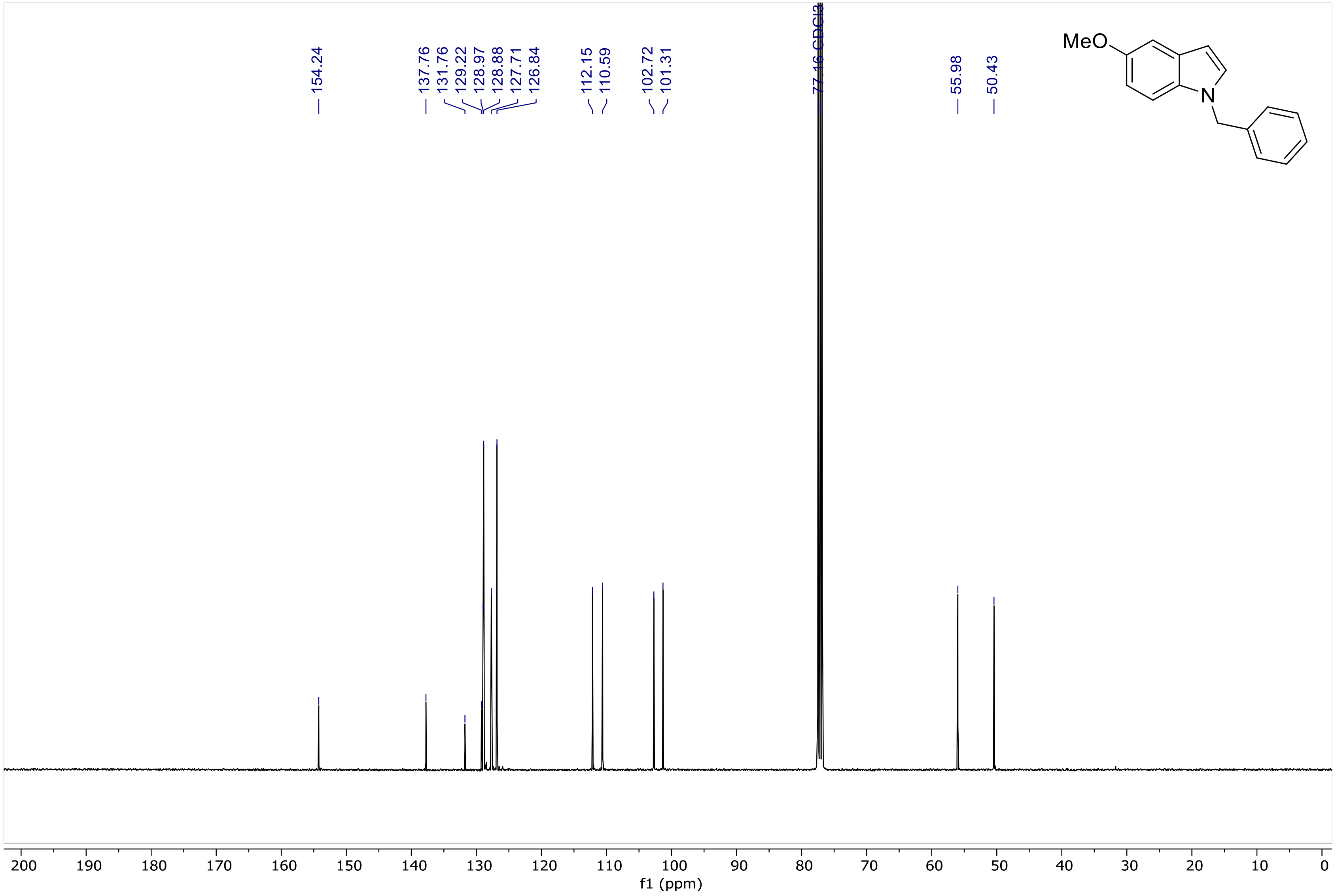

1-Benzyl-5-methylindole – ^{1}H NMR (400 MHz, CDCl₃)

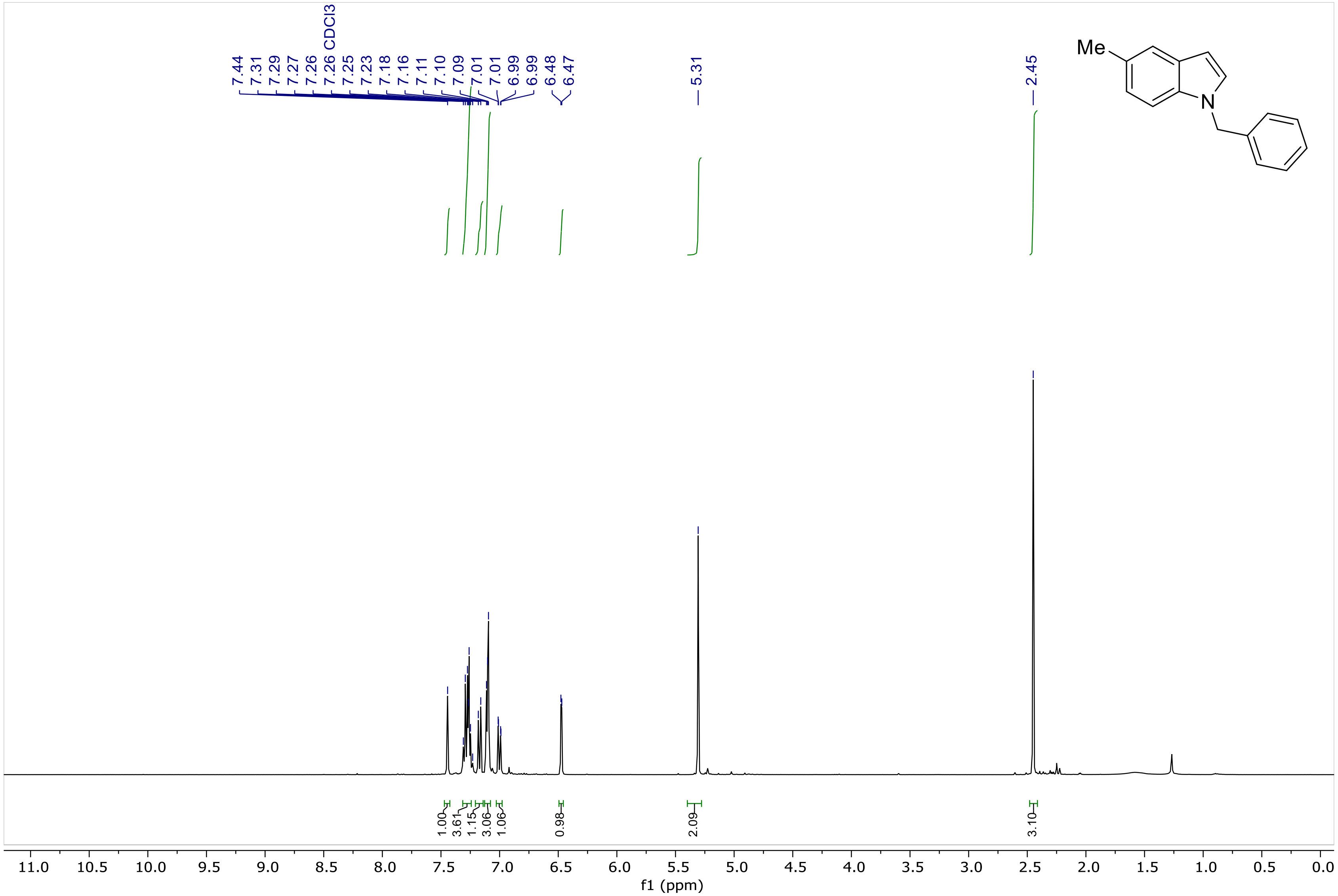

1-Benzyl-5-methylindole – $^{13}C\{^1H\}$ NMR (101 MHz, $CDCl_3$)

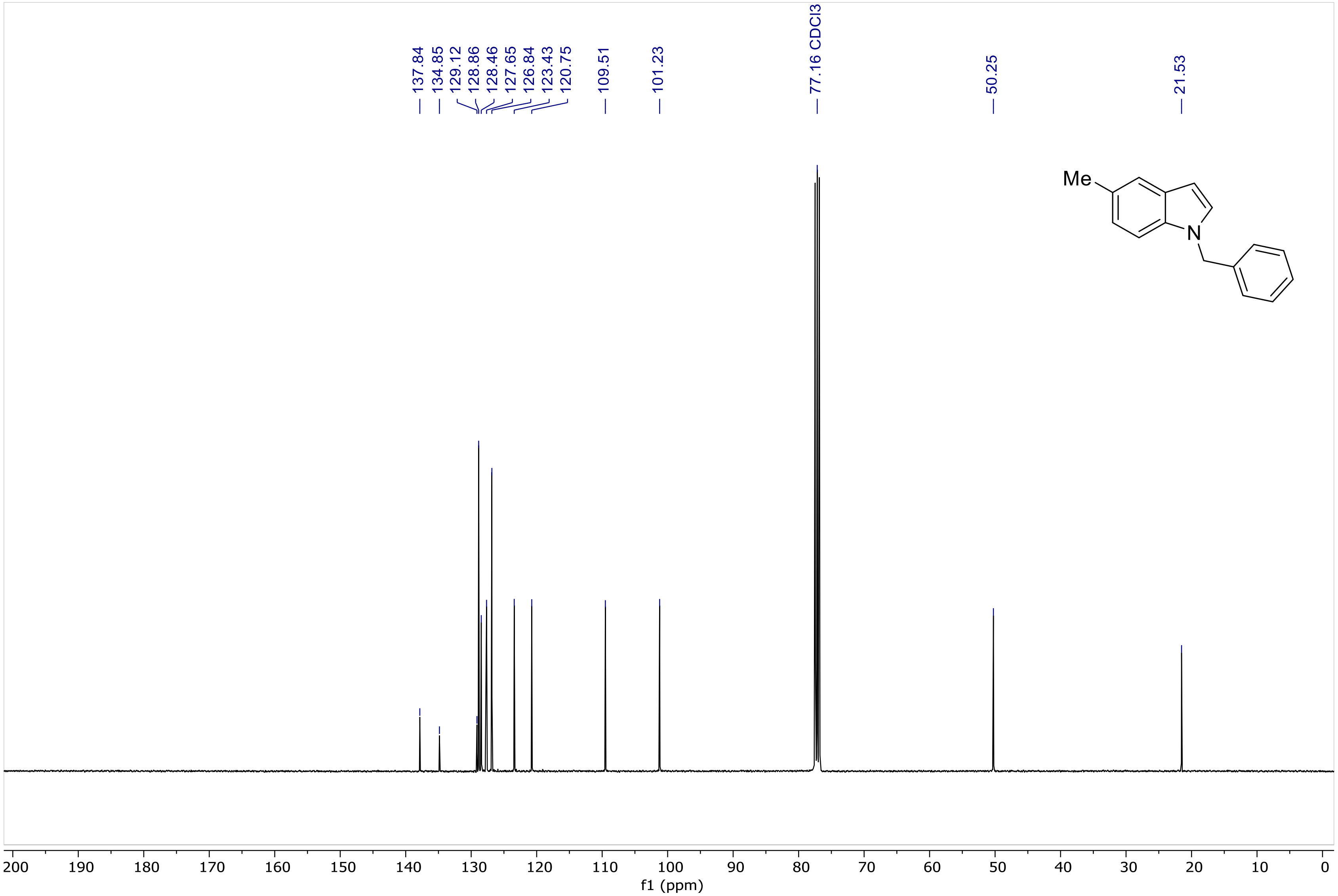

1-Benzyl-6-(benzyloxy)lindole – ^{1}H NMR (400 MHz, $CDCl_3$)

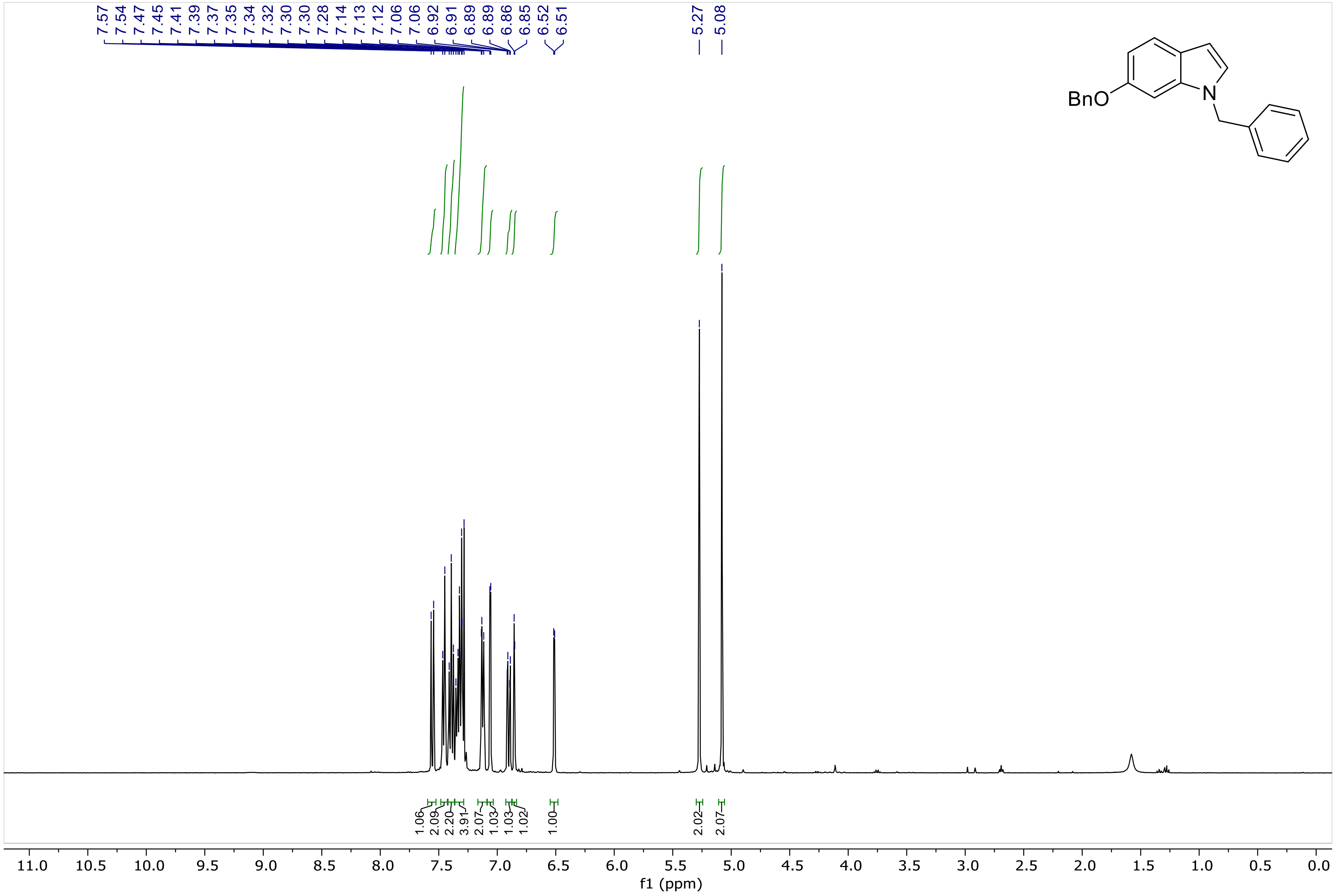

1-Benzyl-6-(benzyloxy)lindole – $^{13}C\{^{1}H\}$ NMR (101 MHz, $CDCl_3$)

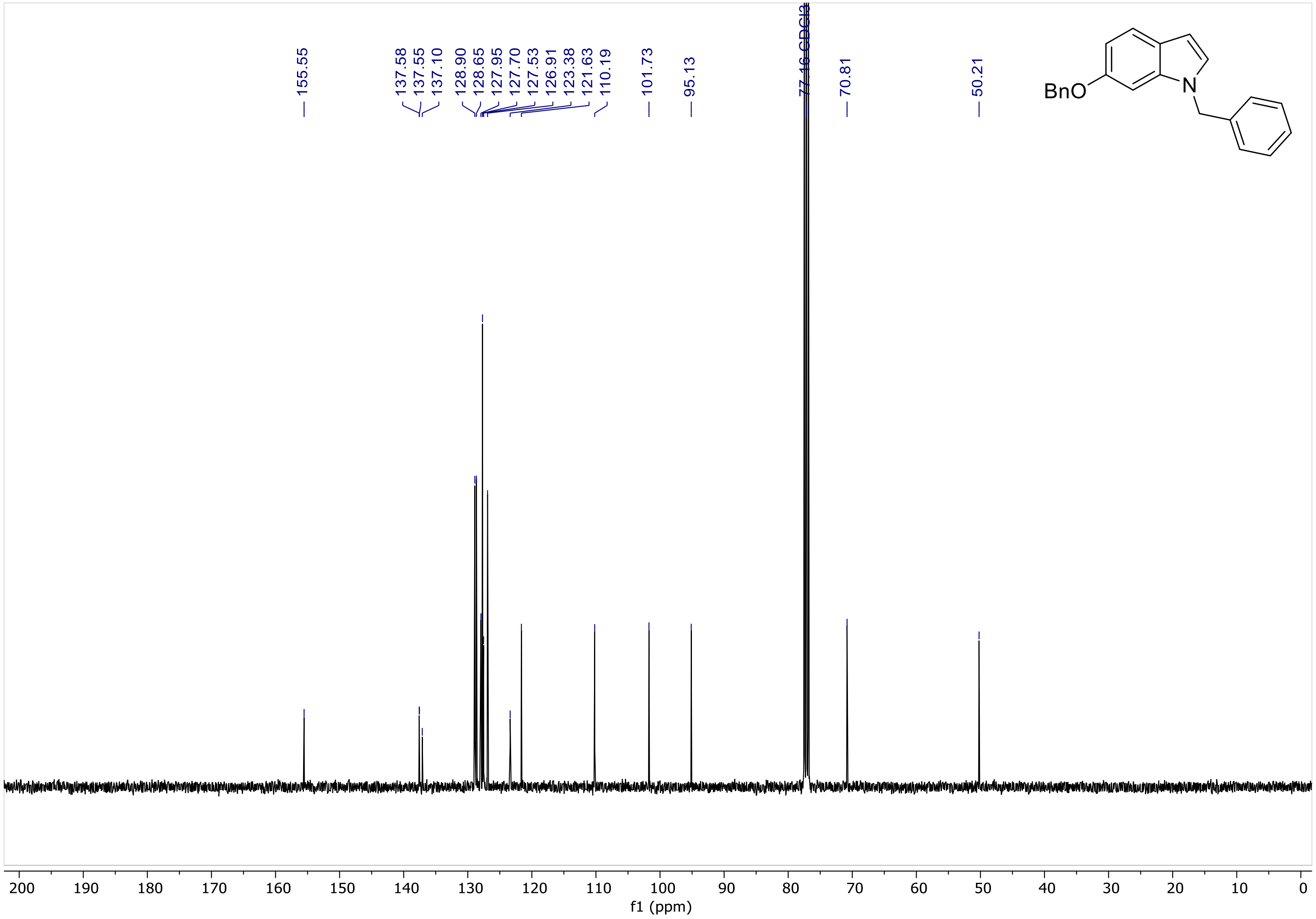

1-Benzyl-5,6-dimethoxylindole – ^{1}H NMR (400 MHz, $CDCl_3$)

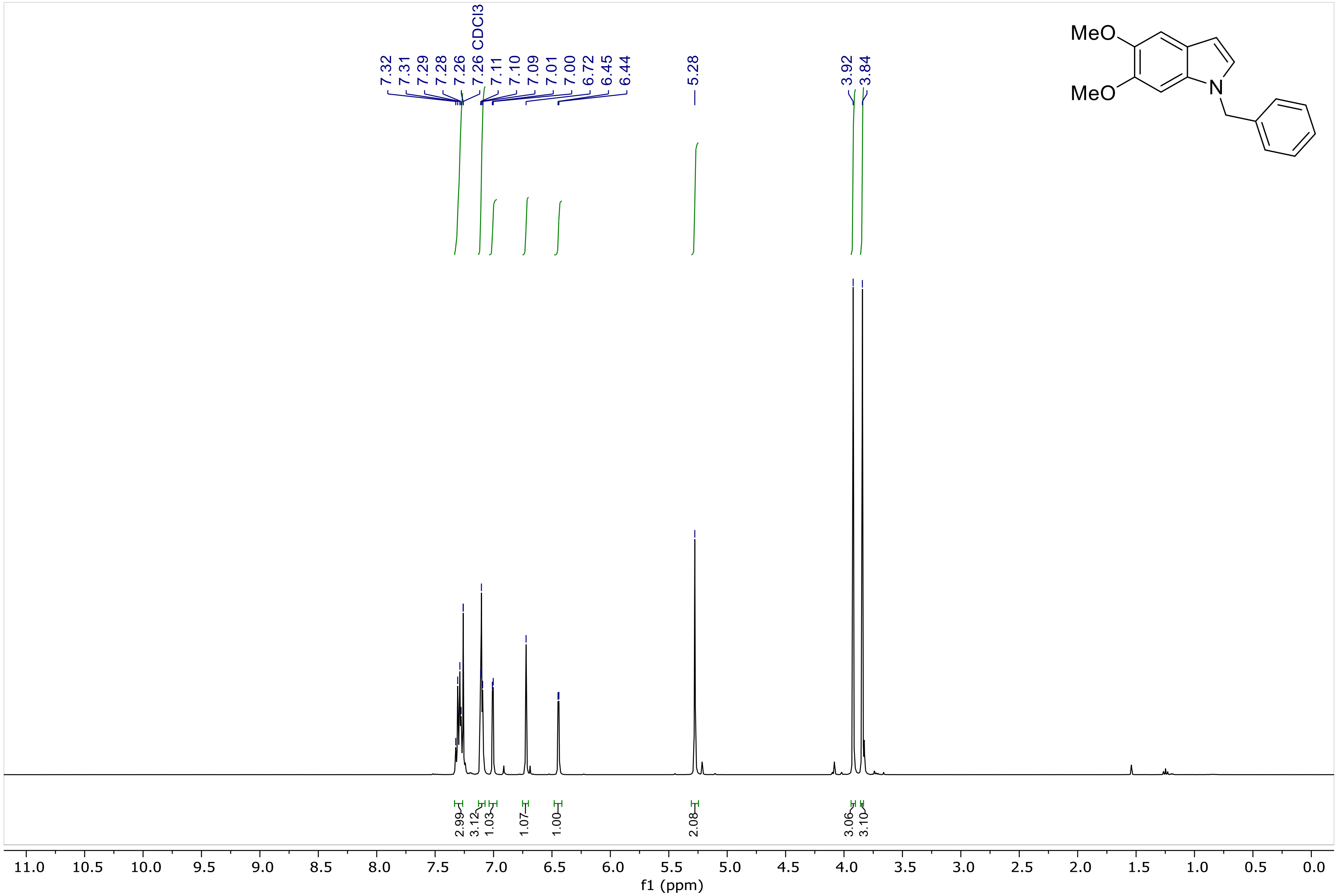

1-Benzyl-5,6-dimethoxylindole – $^{13}C\{^{1}H\}$ NMR (101 MHz, $CDCl_3$)

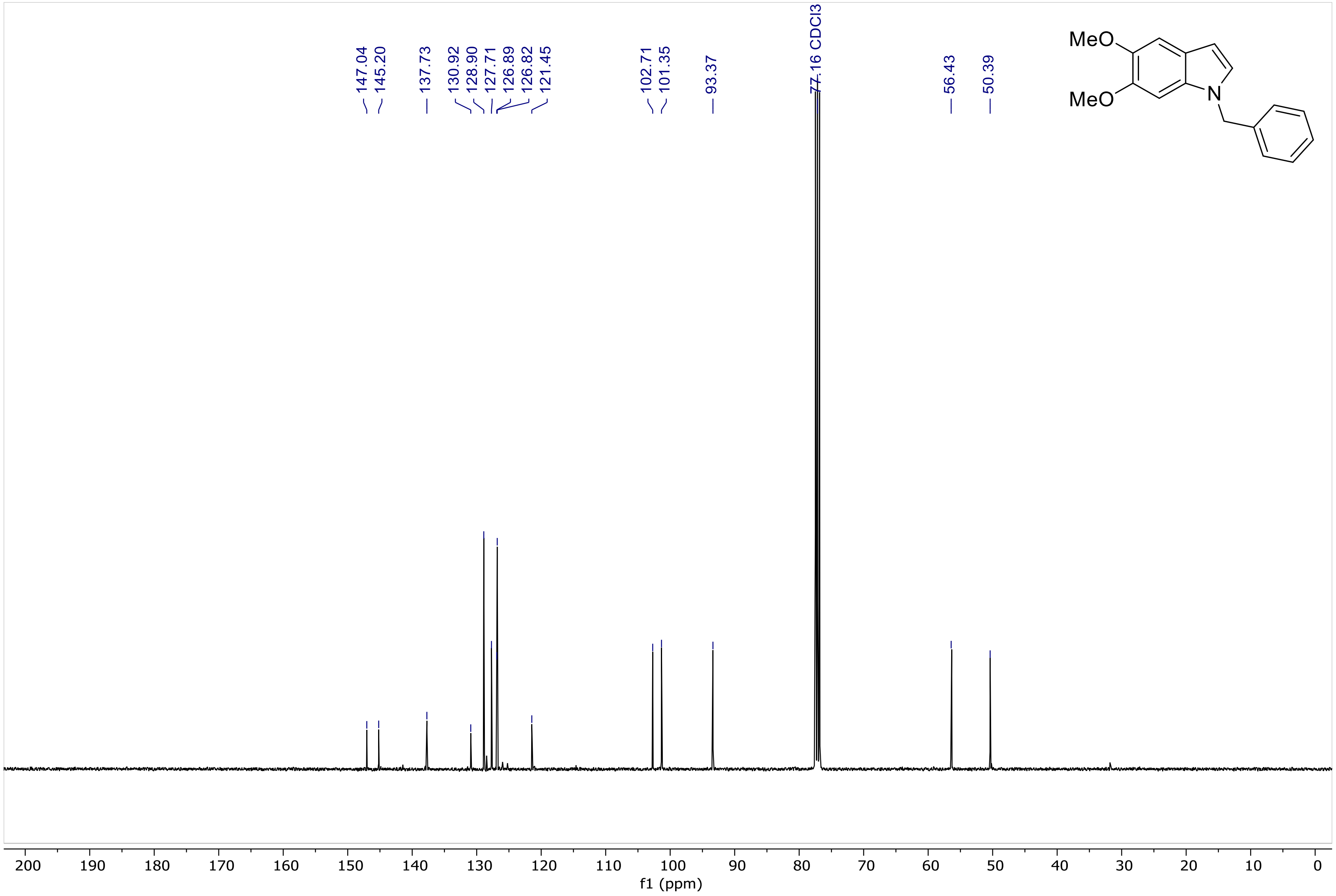

1-Benzyl-4-methylindole – ¹H NMR (400 MHz, $CDCl_3$)

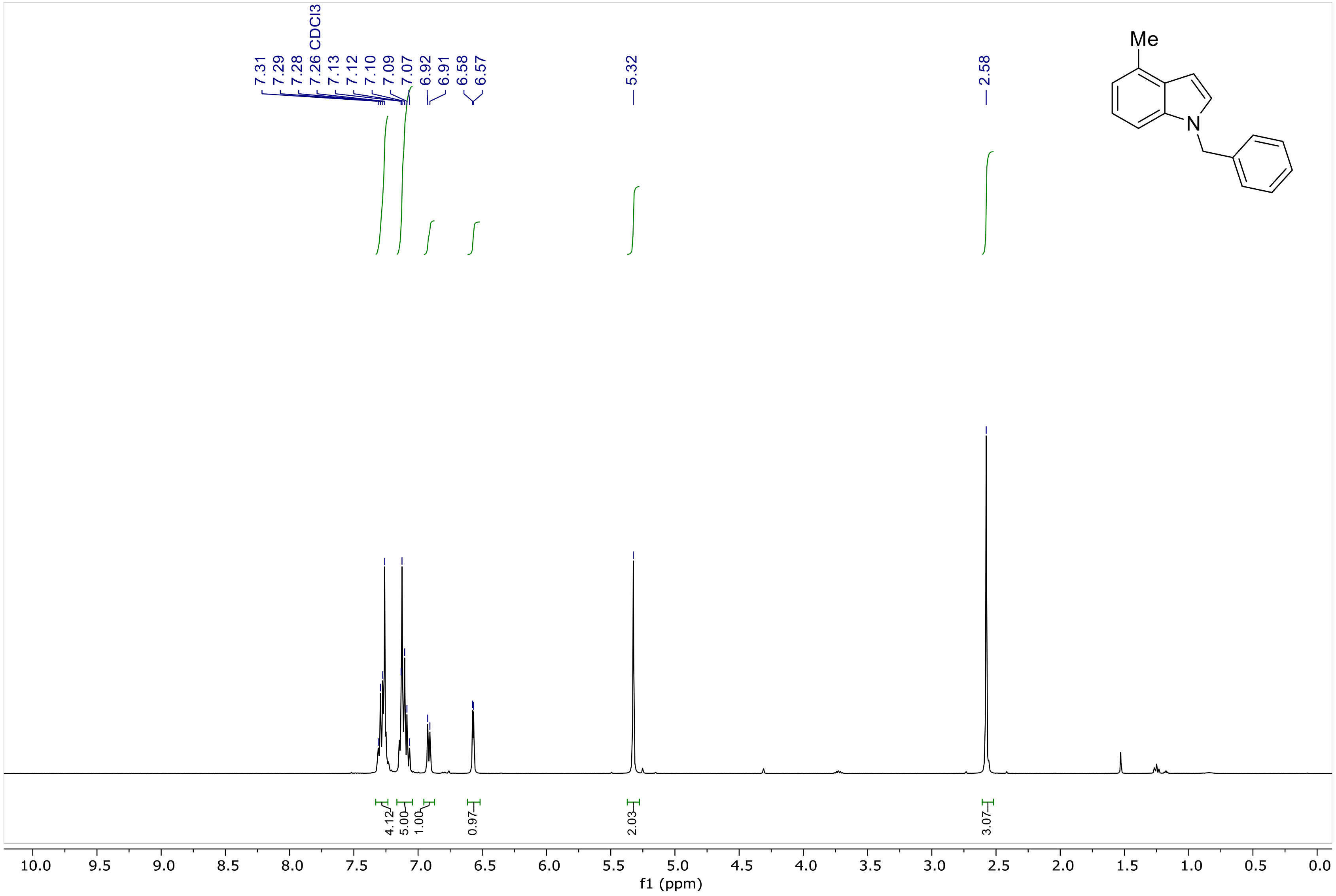

1-Benzyl-4-methylindole – $^{13}C\{^1H\}$ NMR (101 MHz, $CDCl_3$)

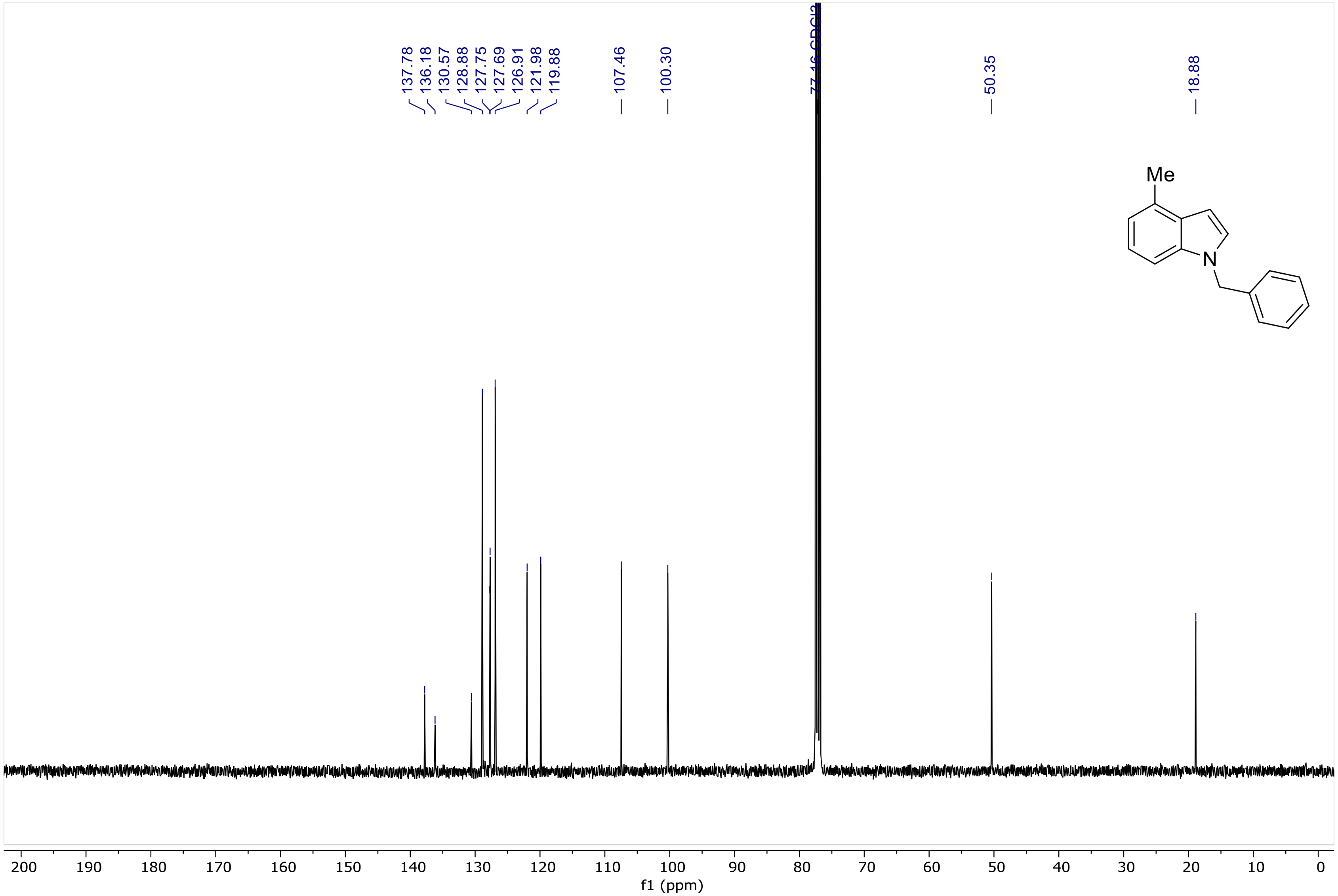

1-Benzyl-7-methylindole – ^{1}H NMR (400 MHz, $CDCl_3$)

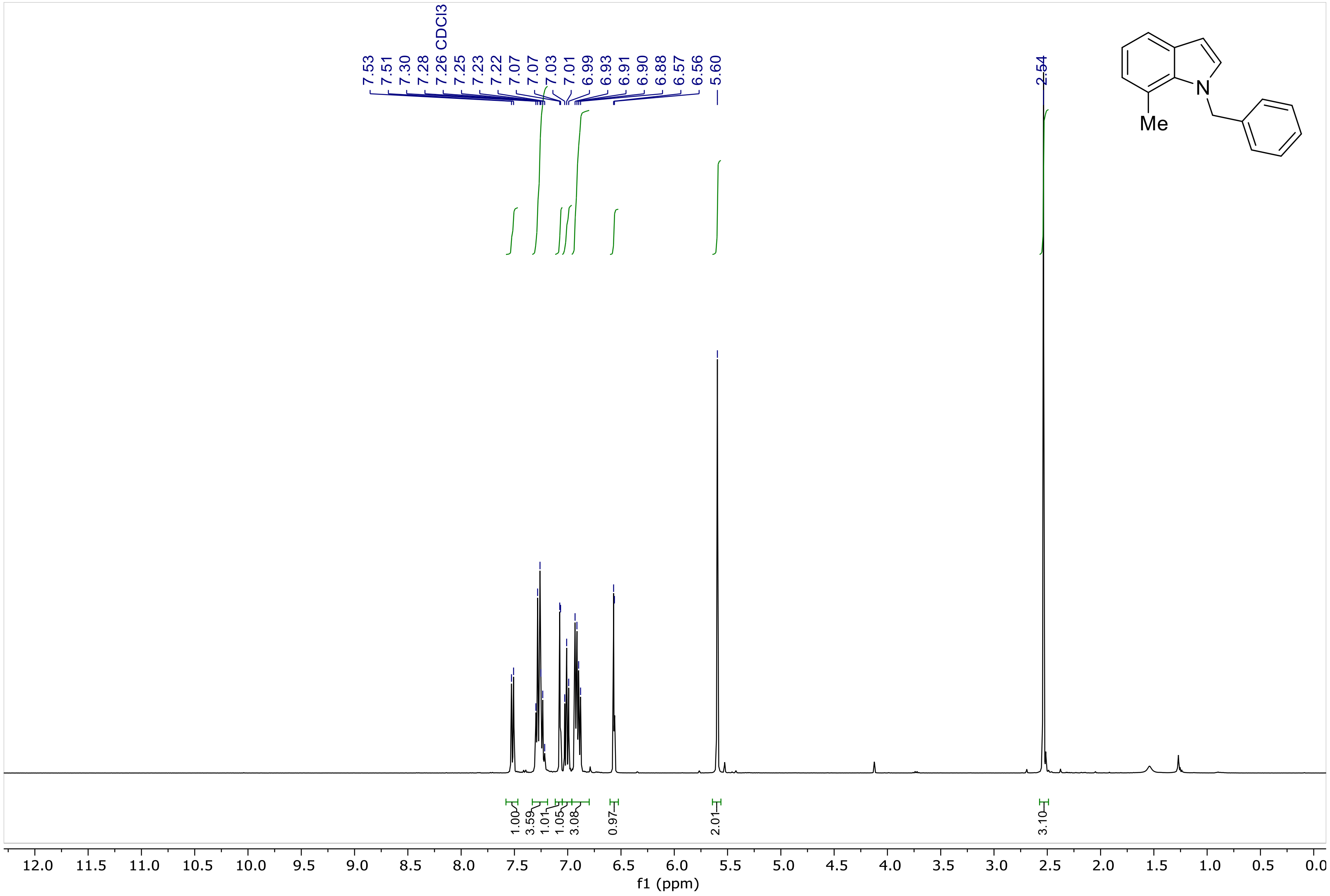

1-Benzyl-7-methylindole – $^{13}C\{^{1}H\}$ NMR (101 MHz, $CDCl_3$)

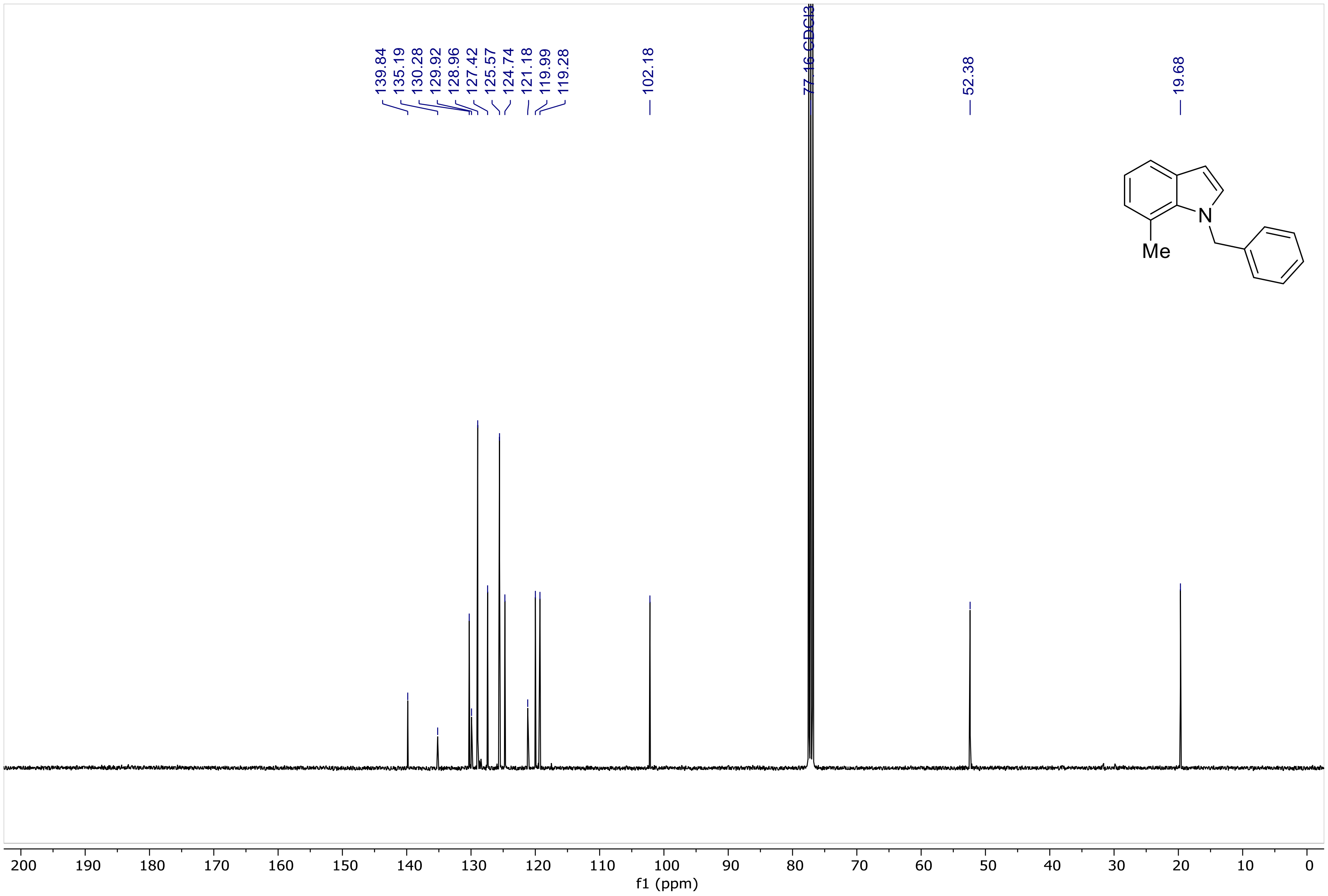

1-Benzyl-7-bromoindole – ^{1}H NMR (400 MHz, $CDCl_3$)

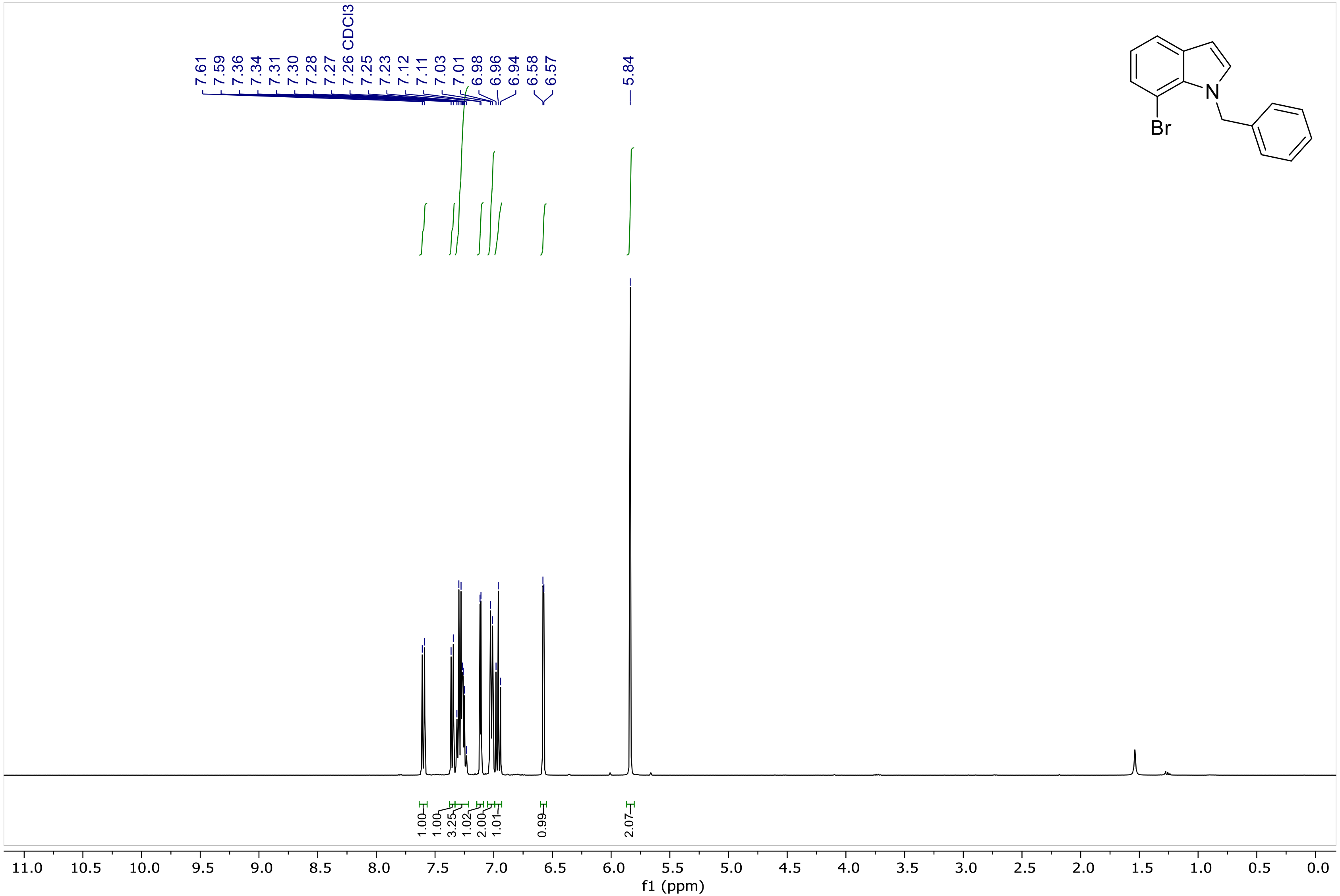

1-Benzyl-7-bromoindole – $^{13}C\{^{1}H\}$ NMR (101 MHz, $CDCl_3$)

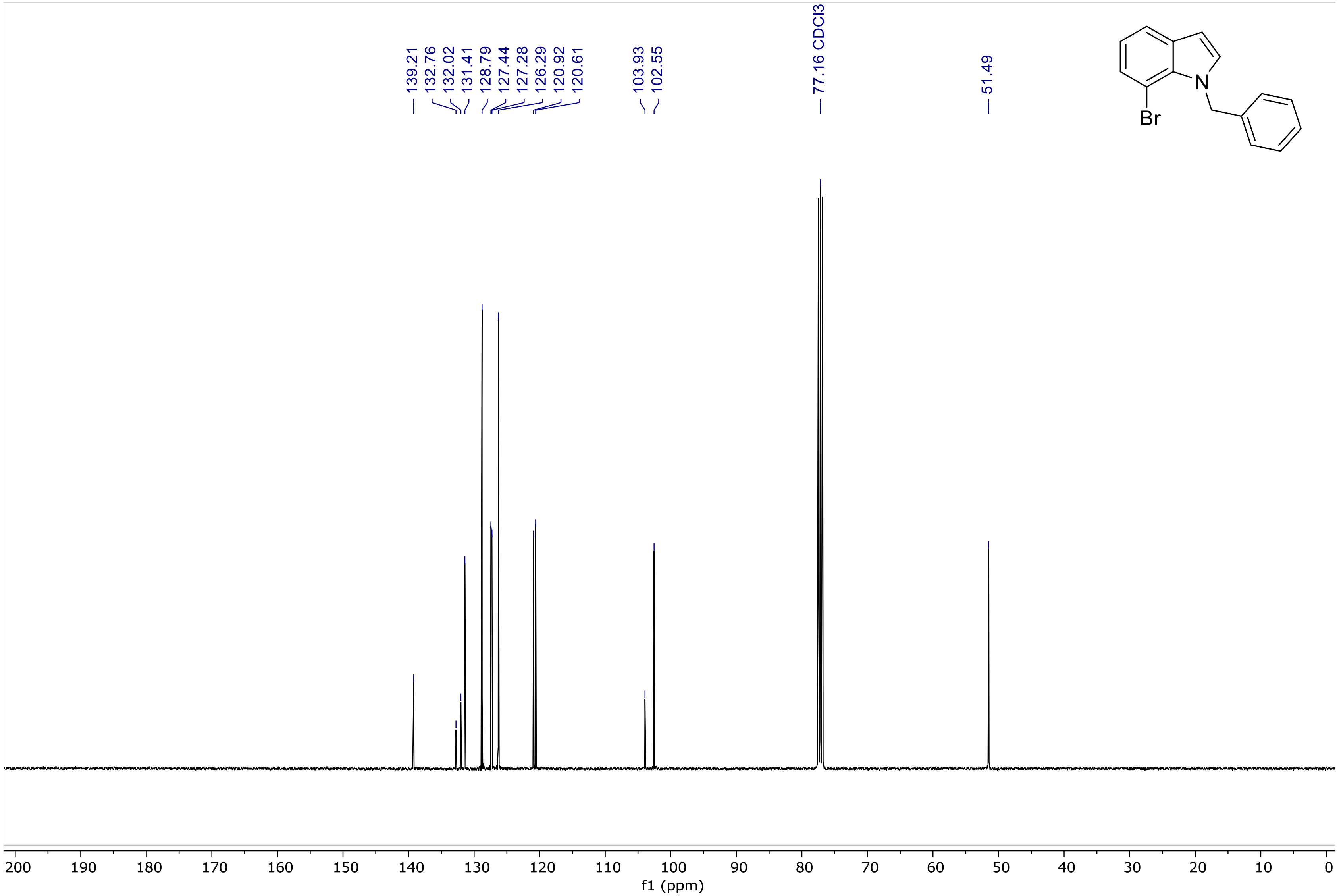

1-Benzyl-4-methoxyindole – ^{1}H NMR (400 MHz, CDCl$_3$)

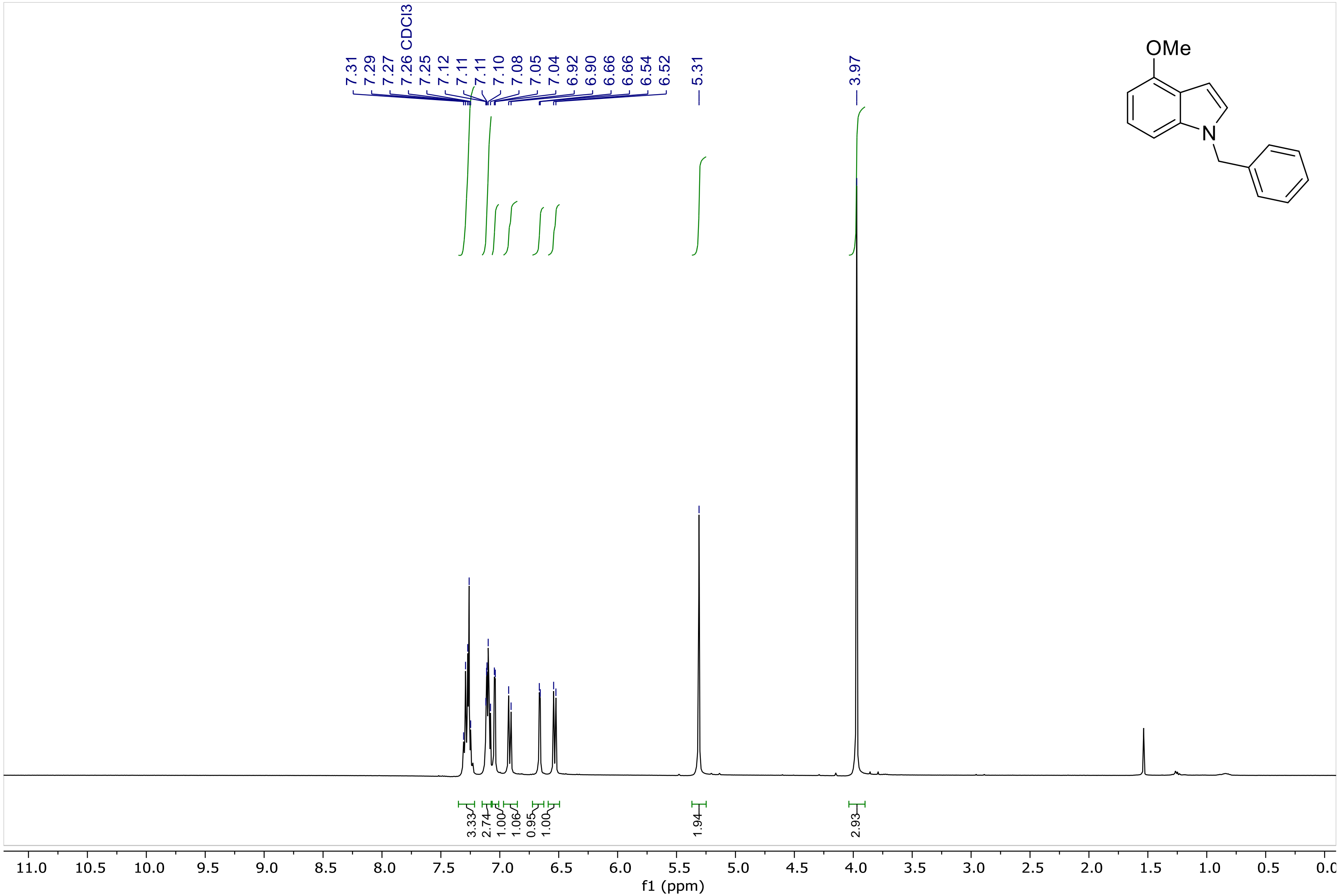

1-Benzyl-4-methoxyindole – $^{13}C\{^{1}H\}$ NMR (101 MHz, $CDCl_3$)

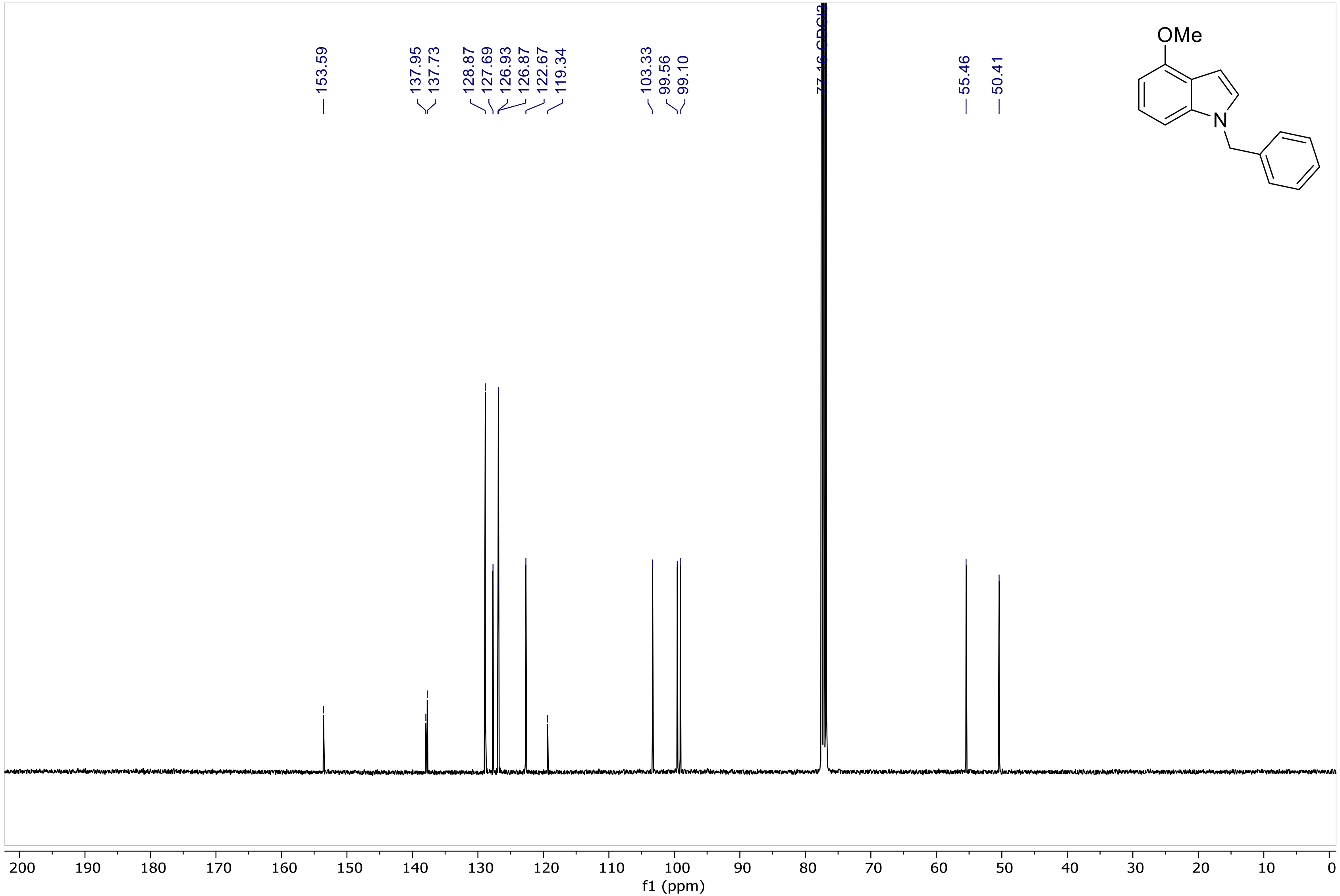

1-Benzyl-2,3-dimethylindole – ¹H NMR (400 MHz, $CDCl_3$)

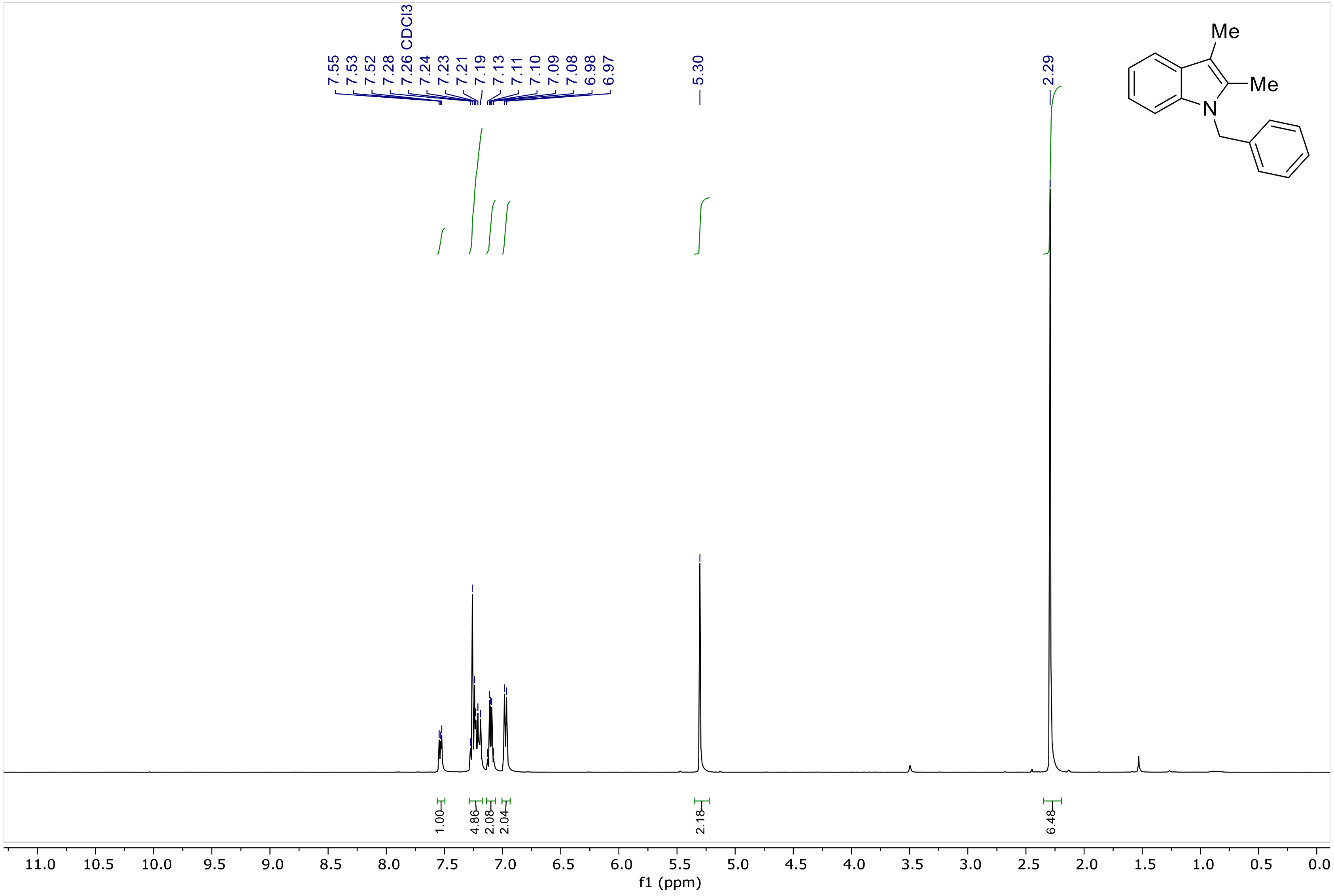

1-Benzyl-2,3-dimethylindole – $^{13}C\{^{1}H\}$ NMR (101 MHz, $CDCl_3$)

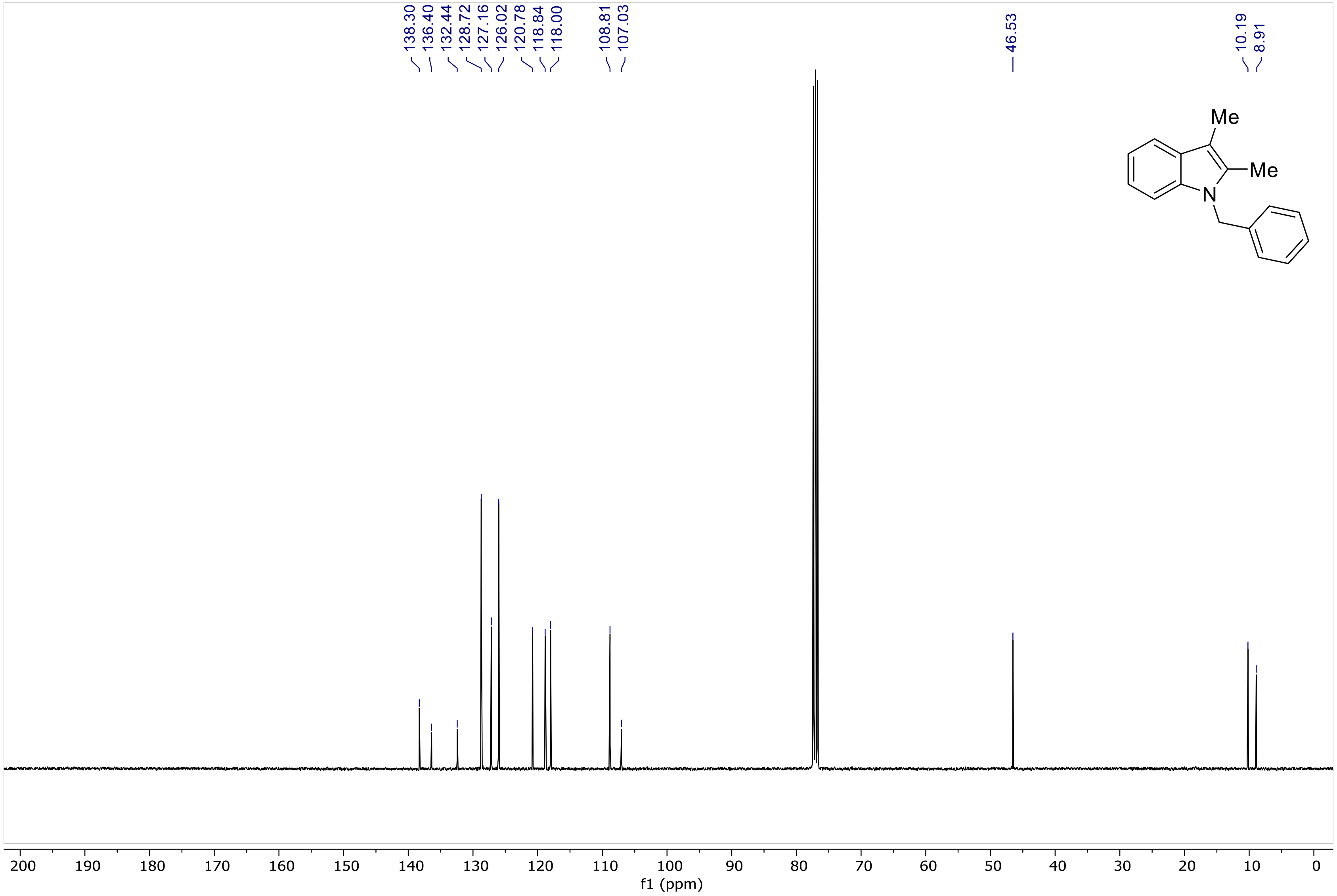

1-Methyl-5-fluoroindole – ^{1}H NMR (400 MHz, $CDCl_3$)

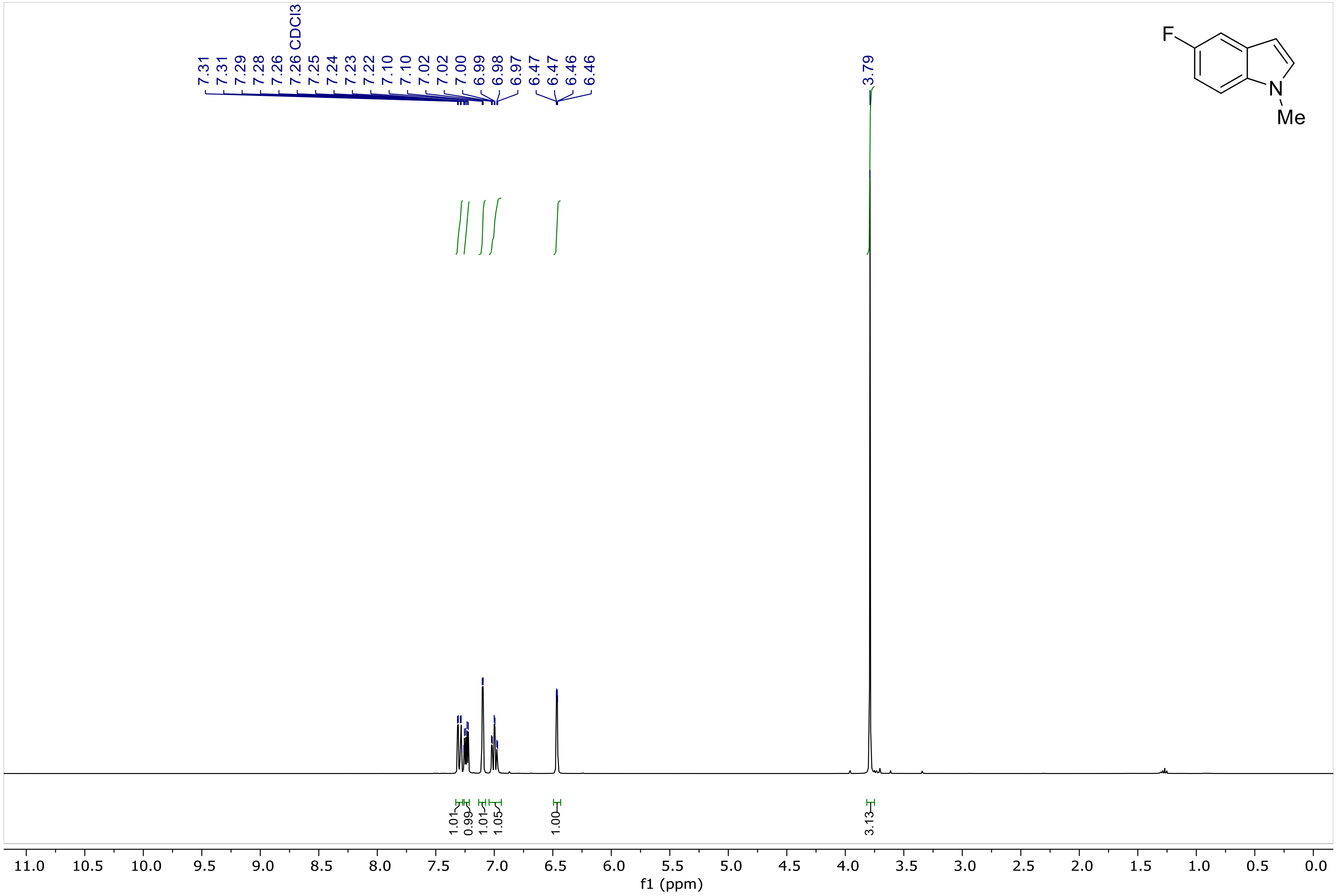

1-Methyl-5-fluoroindole – $^{13}C\{^{1}H\}$ NMR (101 MHz, $CDCl_3$)

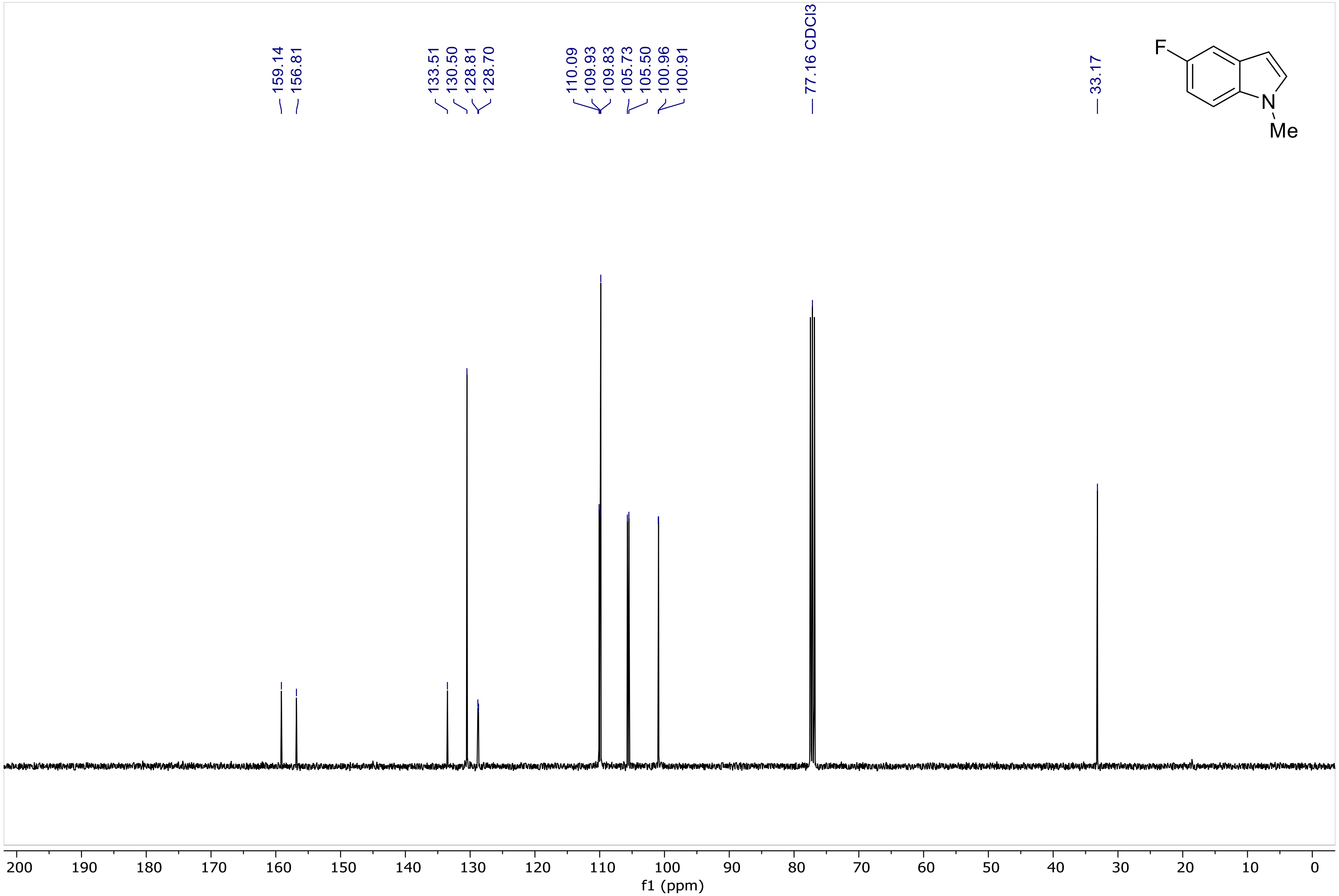

1-Methyl-5-fluoroindole – ^{19}F NMR (376 MHz, $CDCl_3$)

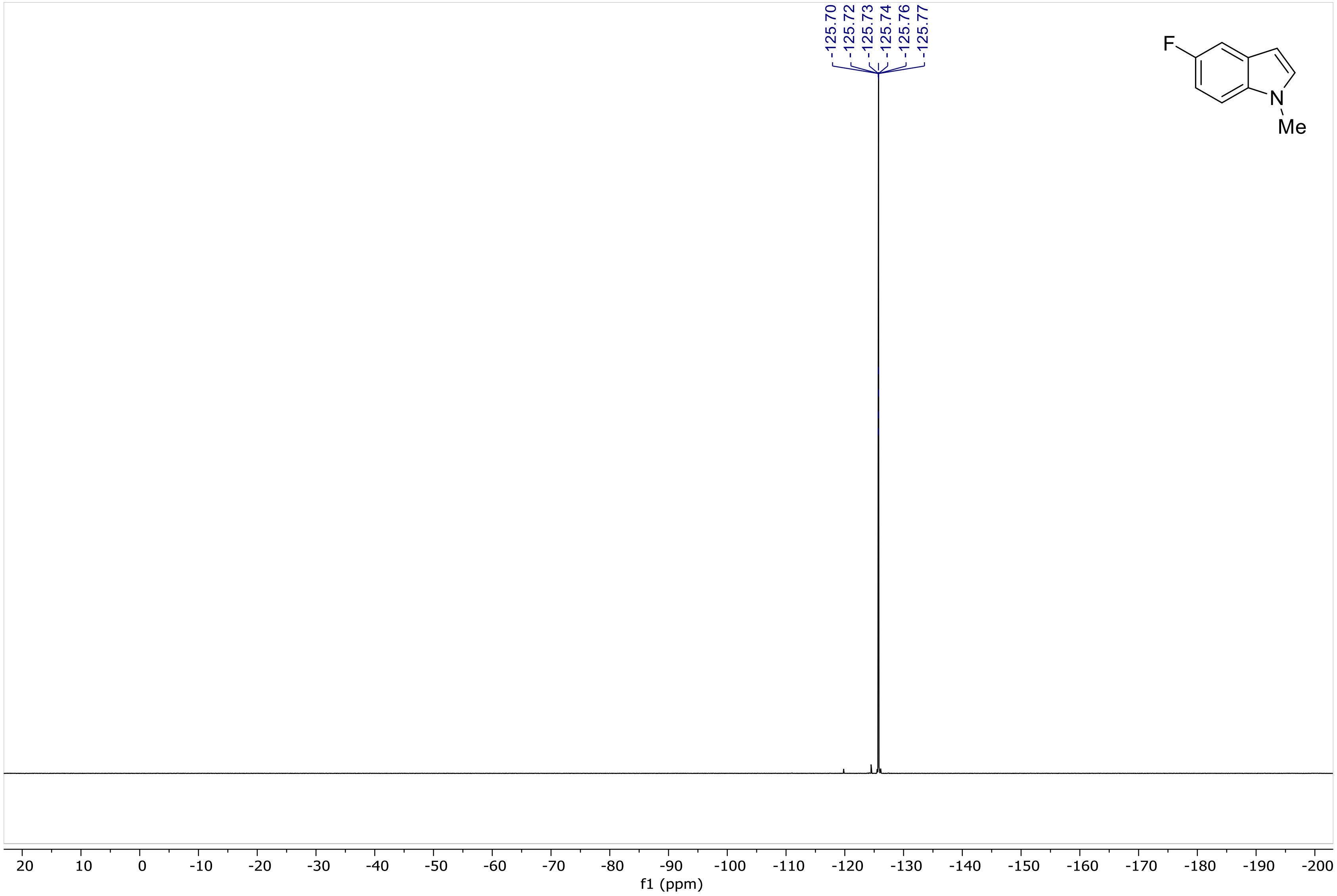

1-(4-Methoxybenzyl)-5-fluoroindole – ^{1}H NMR (400 MHz, $CDCl_3$)

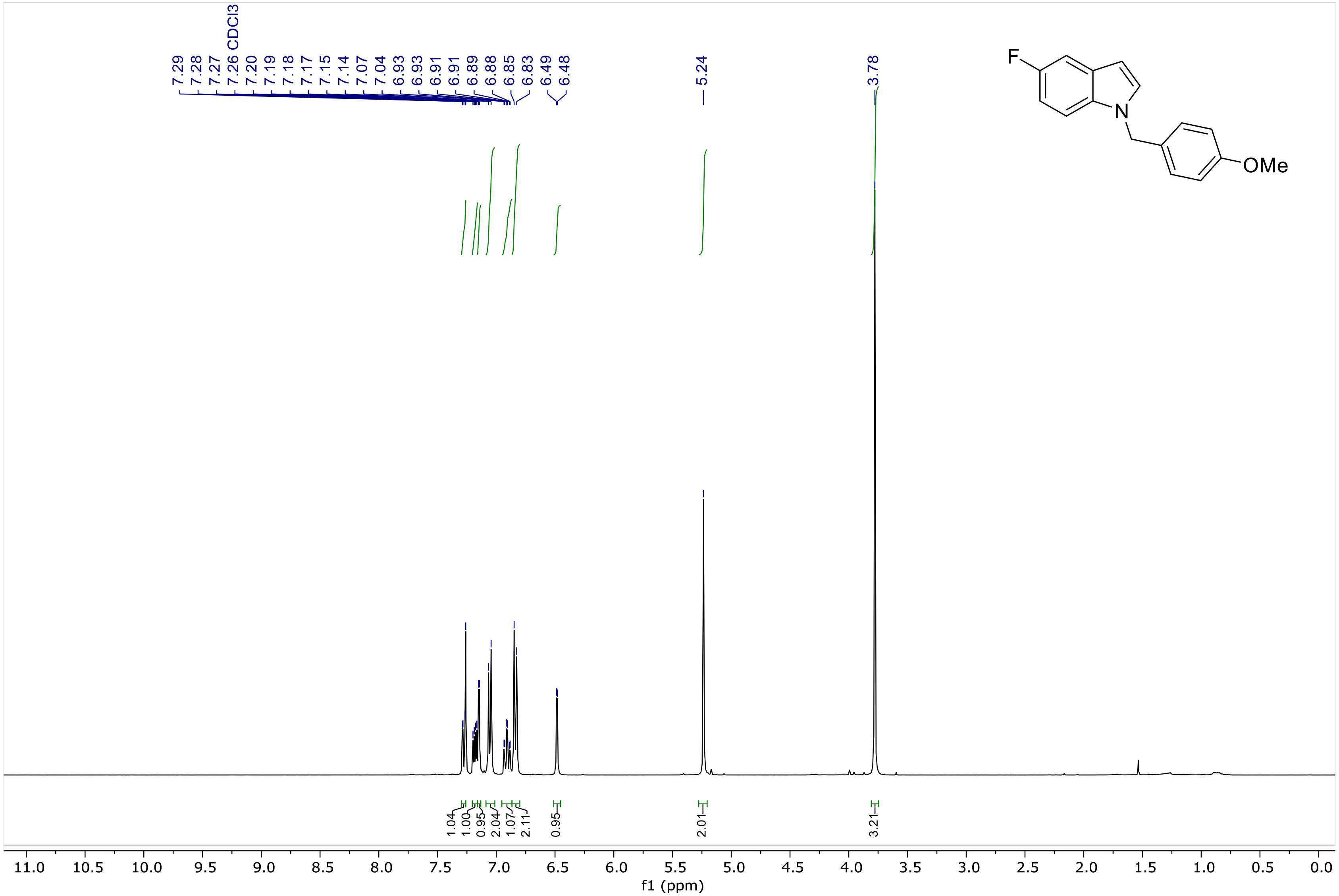

1-(4-Methoxybenzyl)-5-fluoroindole – $^{13}C\{^1H\}$ NMR (101 MHz, $CDCl_3$)

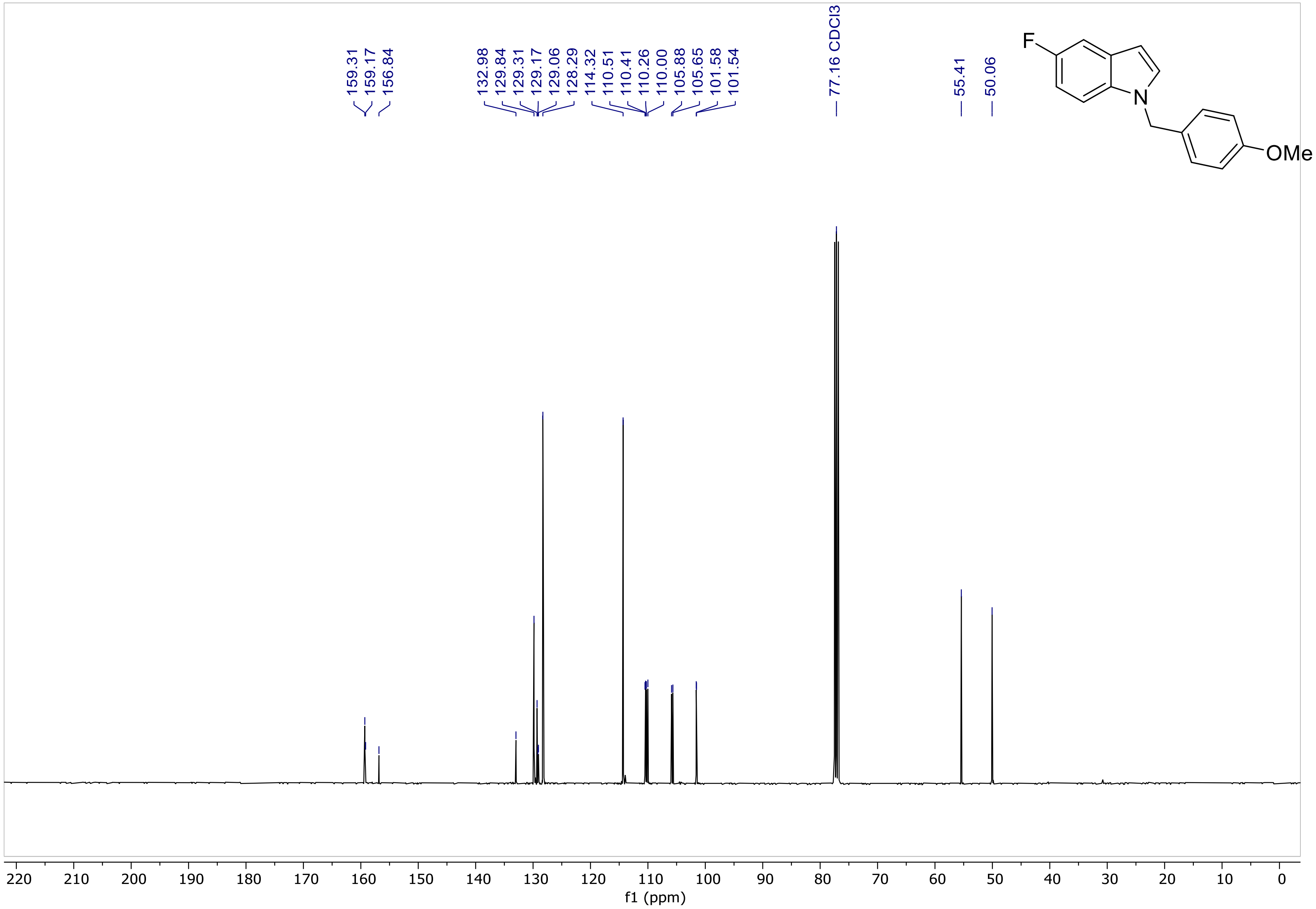

1-(4-Methoxybenzyl)-5-fluoroindole – ^{19}F NMR (376 MHz, $CDCl_3$)

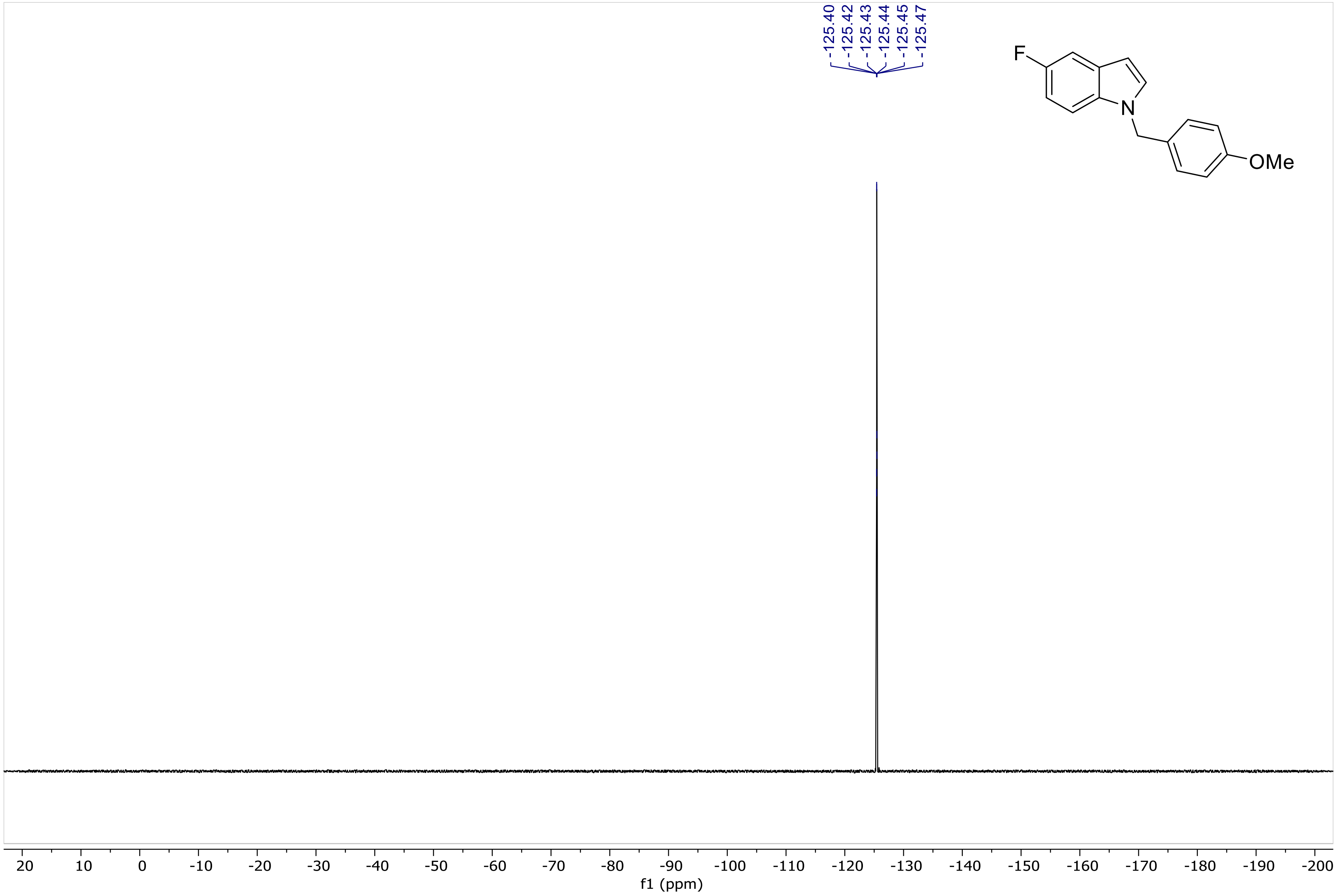

1-Benzyl-7-azaindole – ^{1}H NMR (400 MHz, $CDCl_3$)

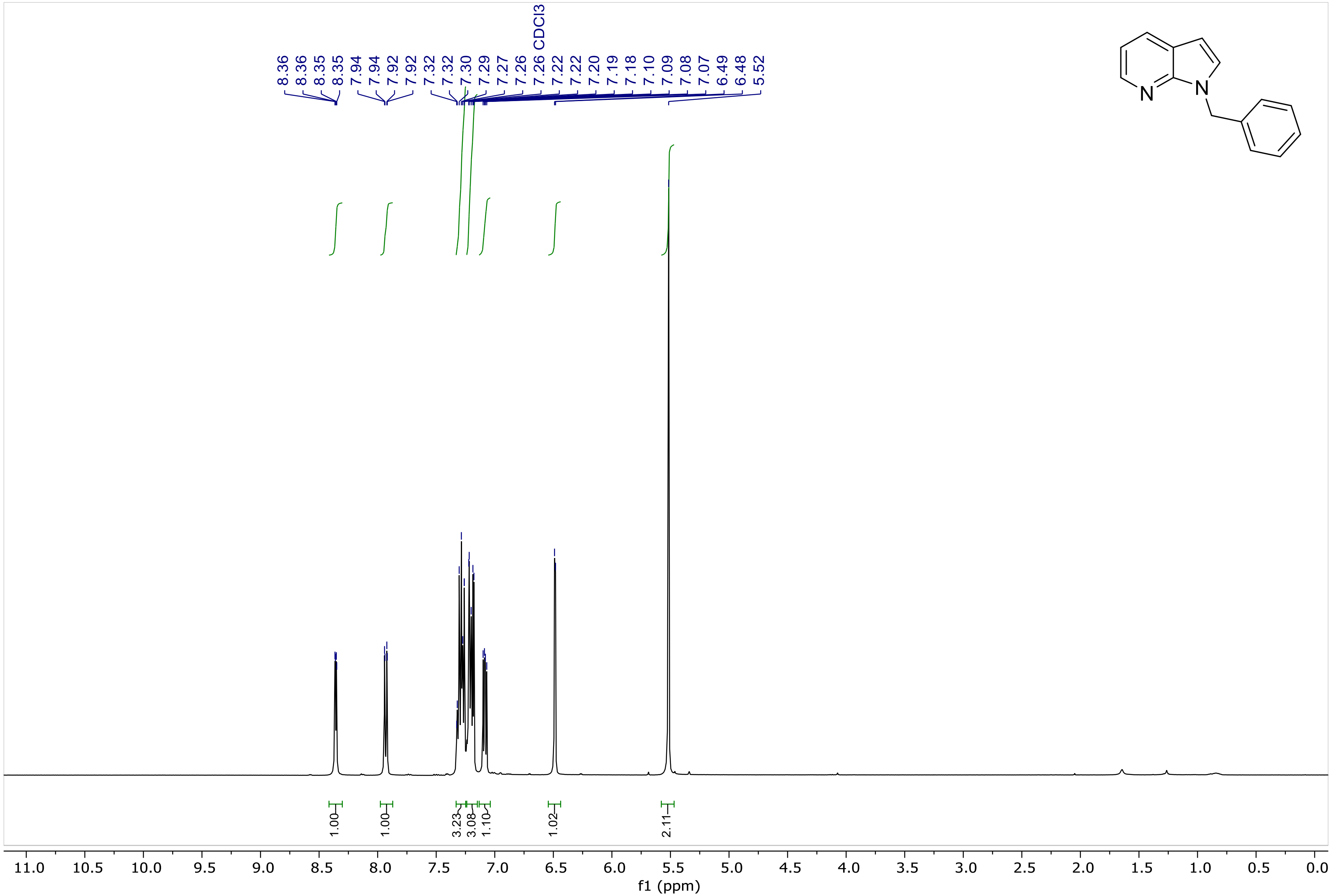

1-Benzyl-7-azaindole – $^{13}C\{^1H\}$ NMR (101 MHz, $CDCl_3$)

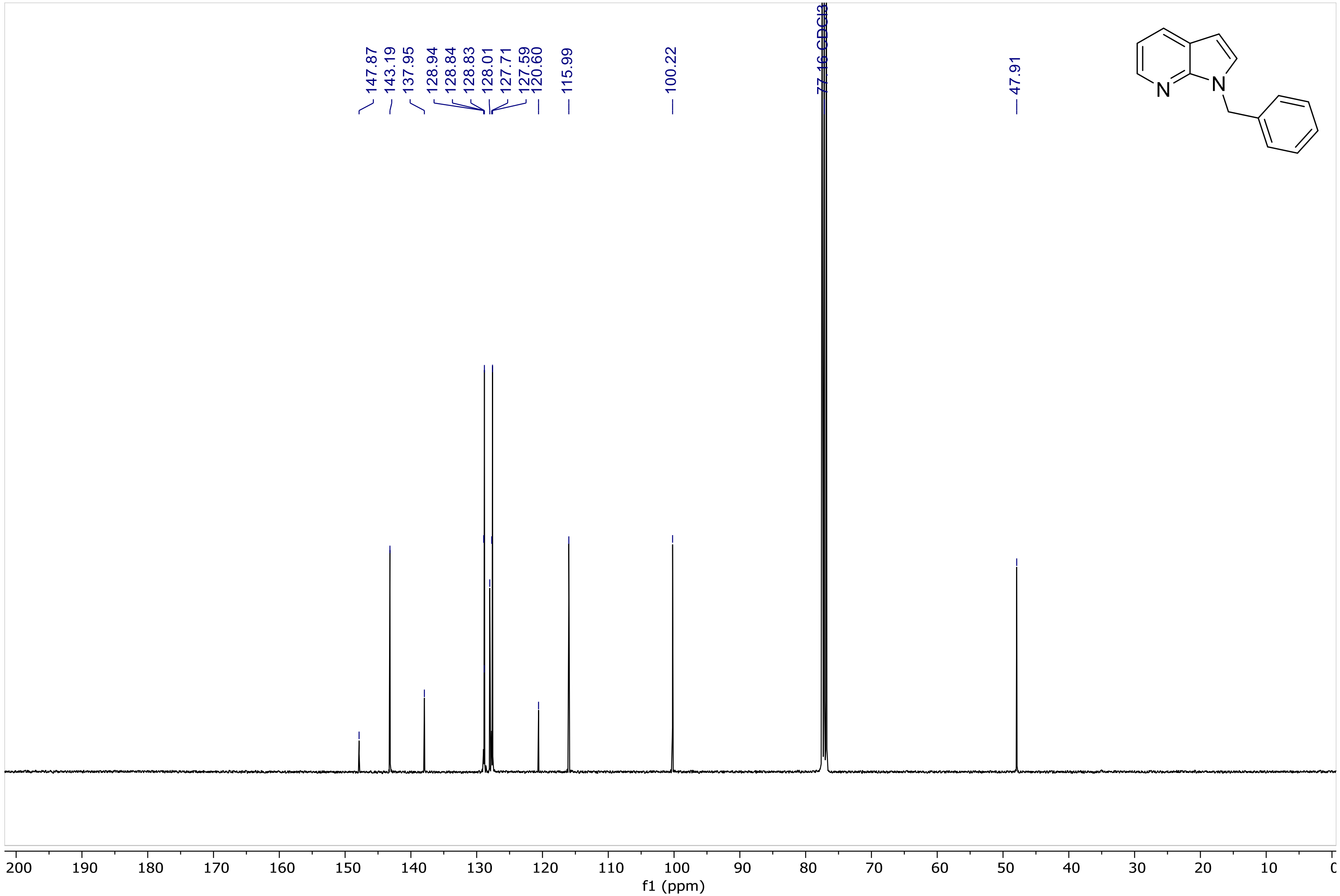

1-Benzylmetalonin – ^{1}H NMR (400 MHz, $CDCl_3$)

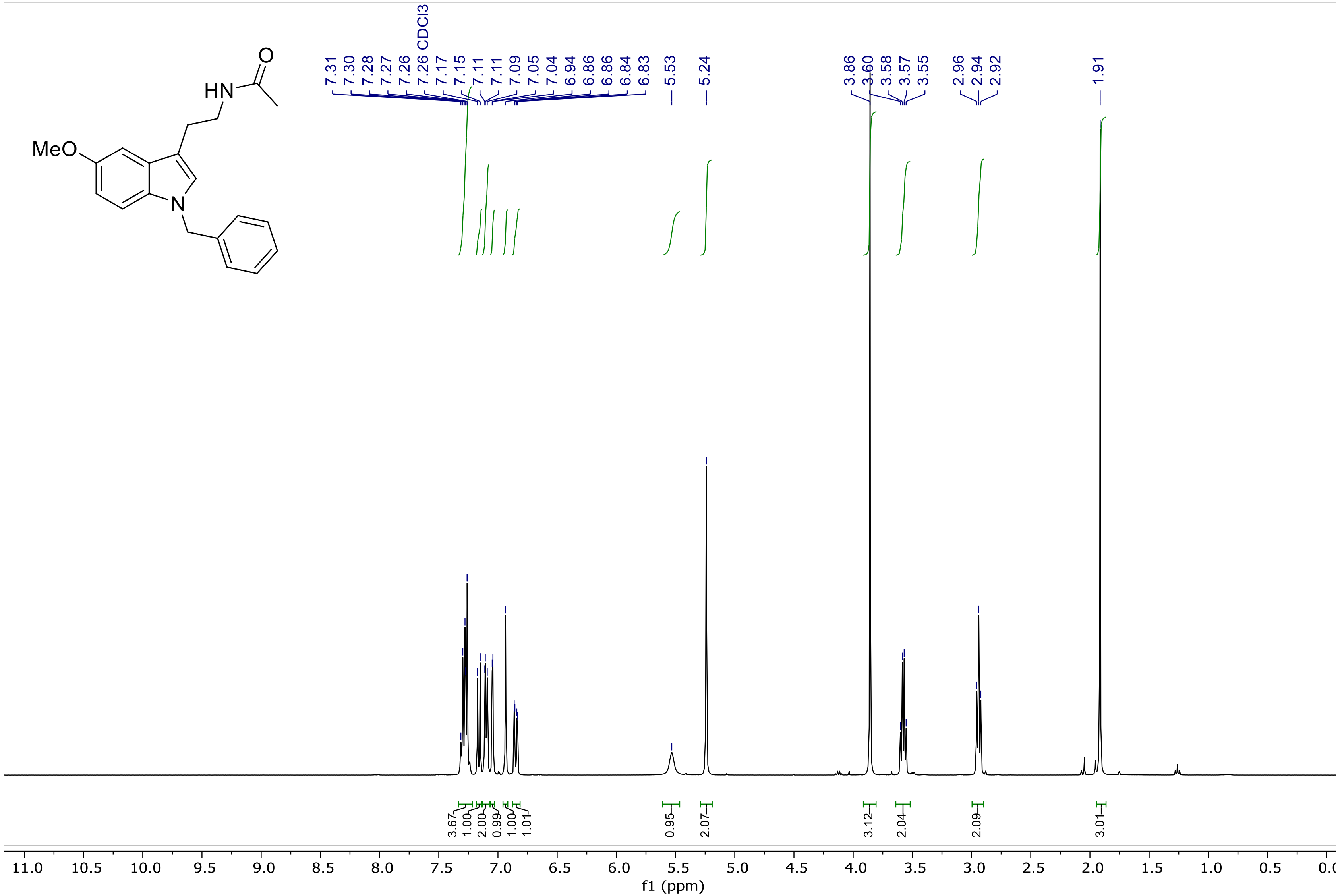

1-Benzylmetalonin – $^{13}C\{^{1}H\}$ NMR (101 MHz, $CDCl_3$)

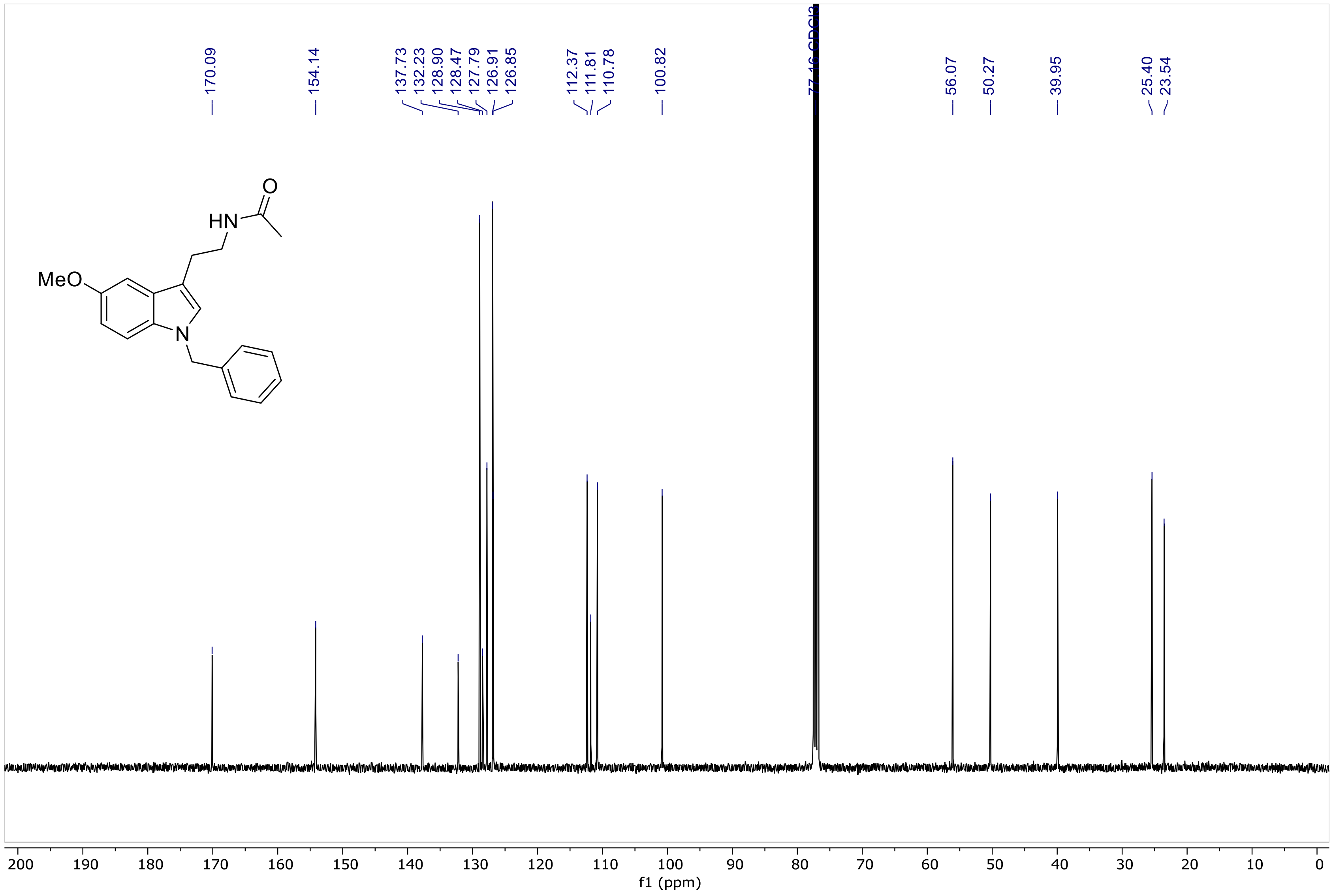

N^{α}-Acetyltryptophan – ^{1}H NMR (400 MHz, DMSO-d$_6$)

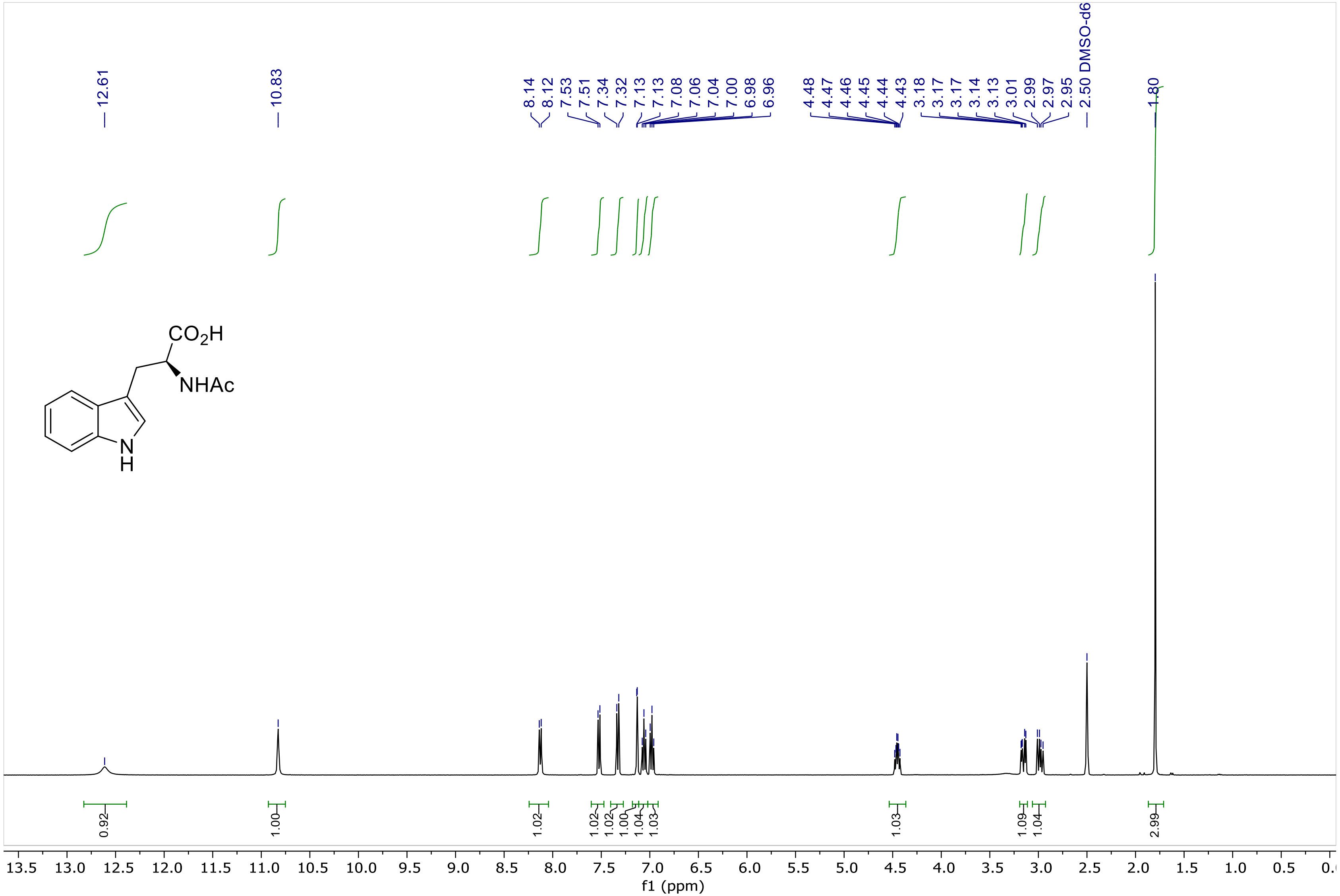

N^{α}-Acetyltryptophan – $^{13}C\{^1H\}$ NMR (101 MHz, DMSO-d$_6$)

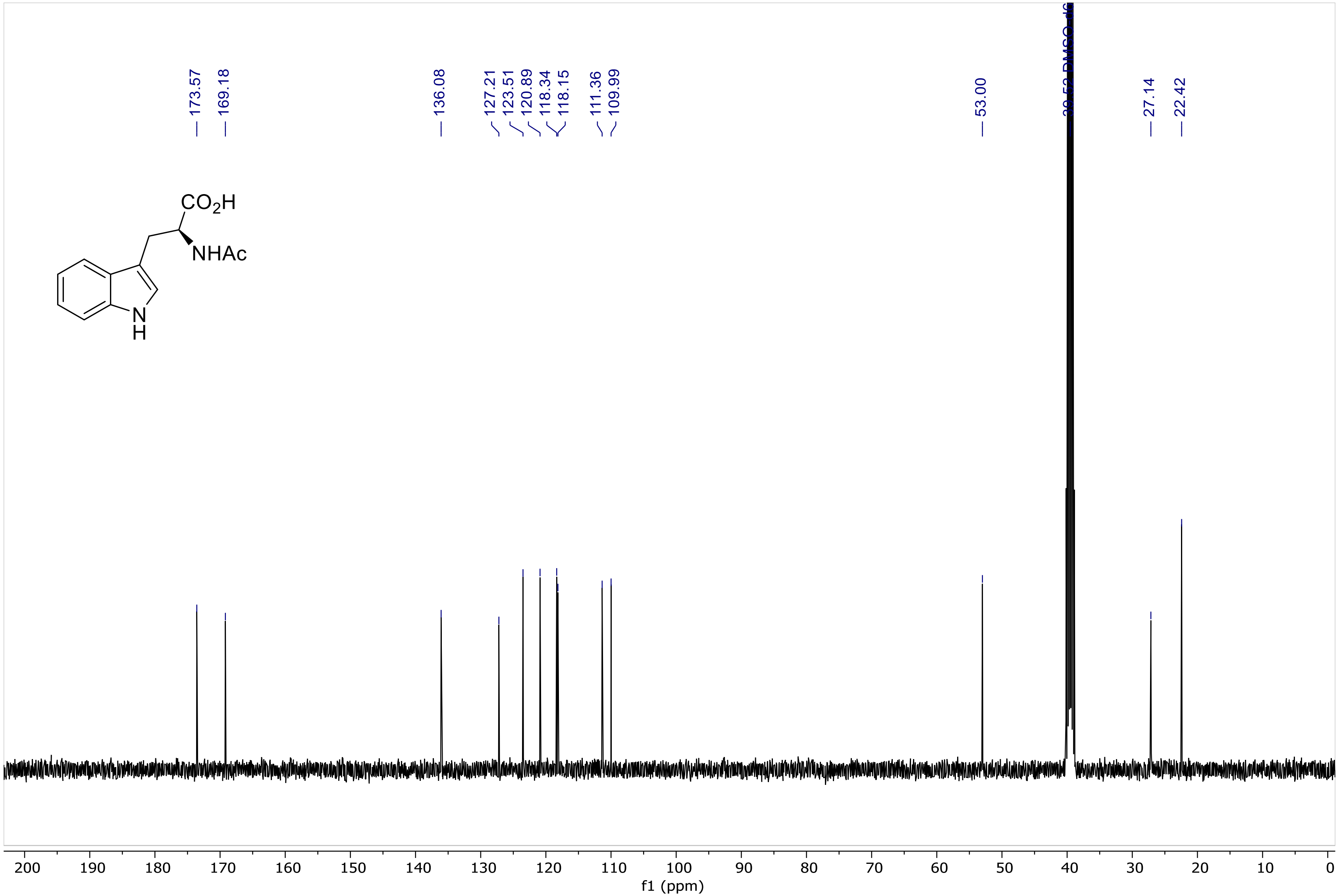

1-Benzyl-N^{α}-Acetyltryptophan – ^{1}H NMR (400 MHz, DMSO-d$_6$)

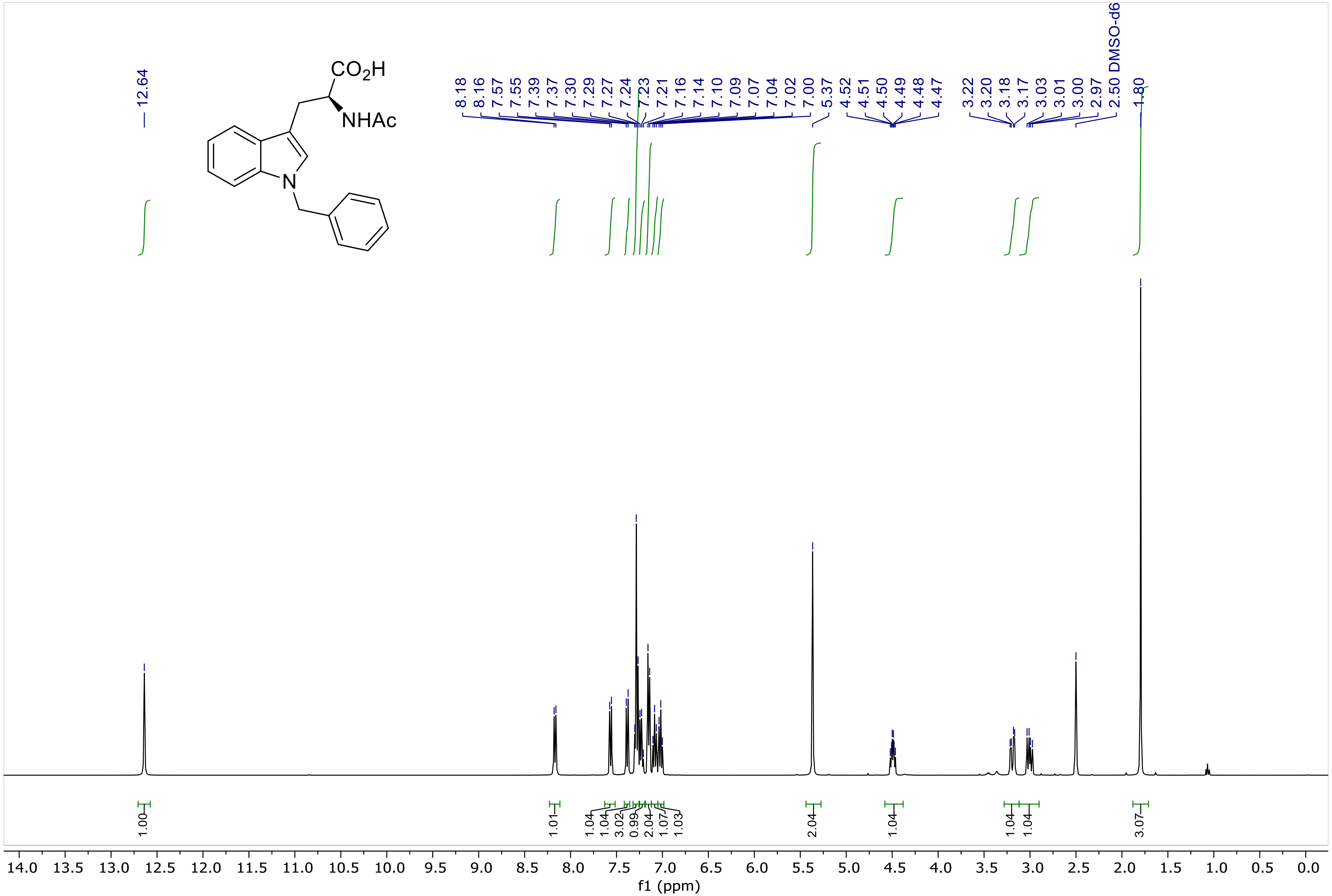

1-Benzyl-N^{α}-Acetyltryptophan – $^{13}C\{^1H\}$ NMR (101 MHz, DMSO-d$_6$)

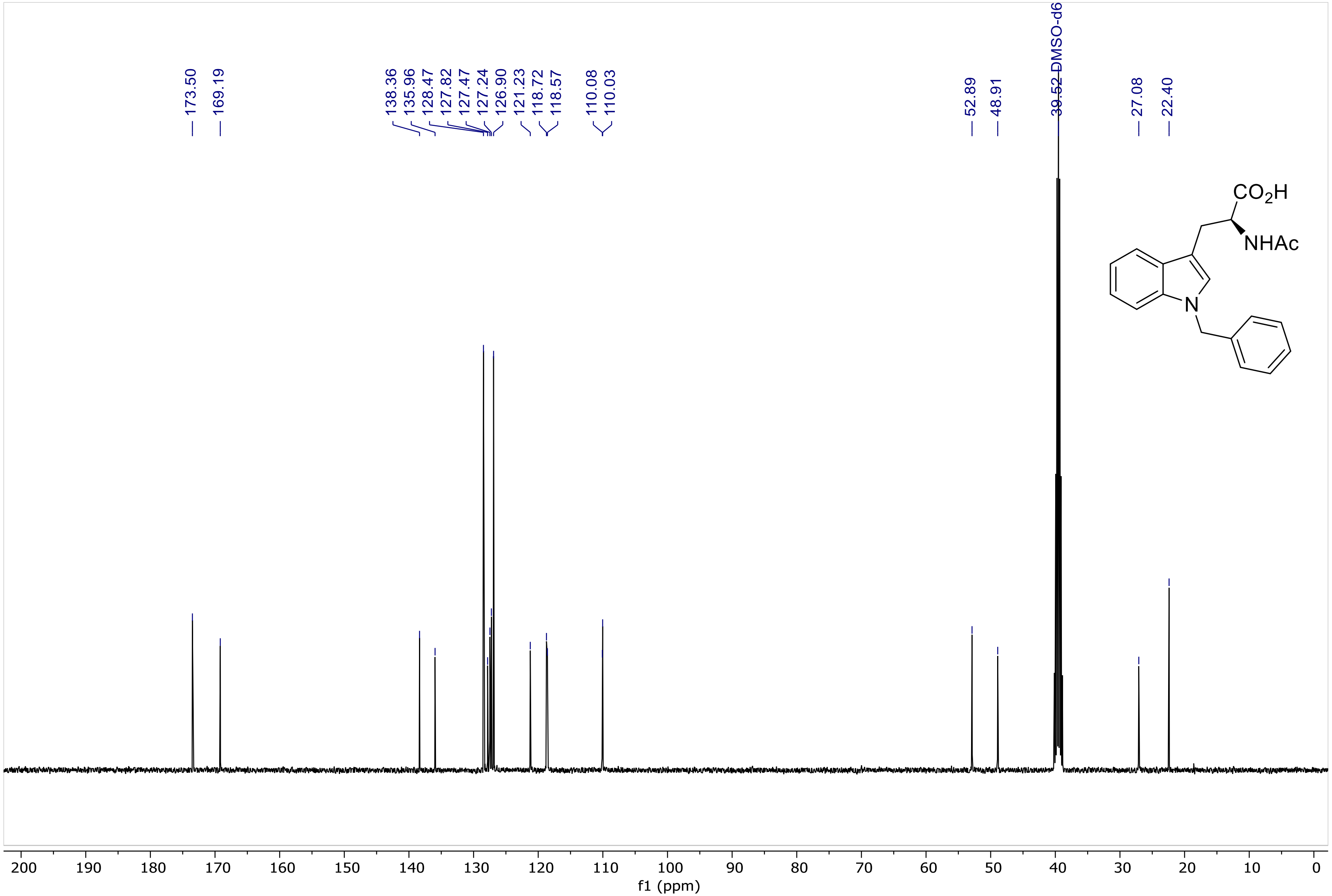

1-Benzyl-N^{α}-Acetyl-L-tryptophan methyl ester – ^{1}H NMR (400 MHz, $CDCl_3$)

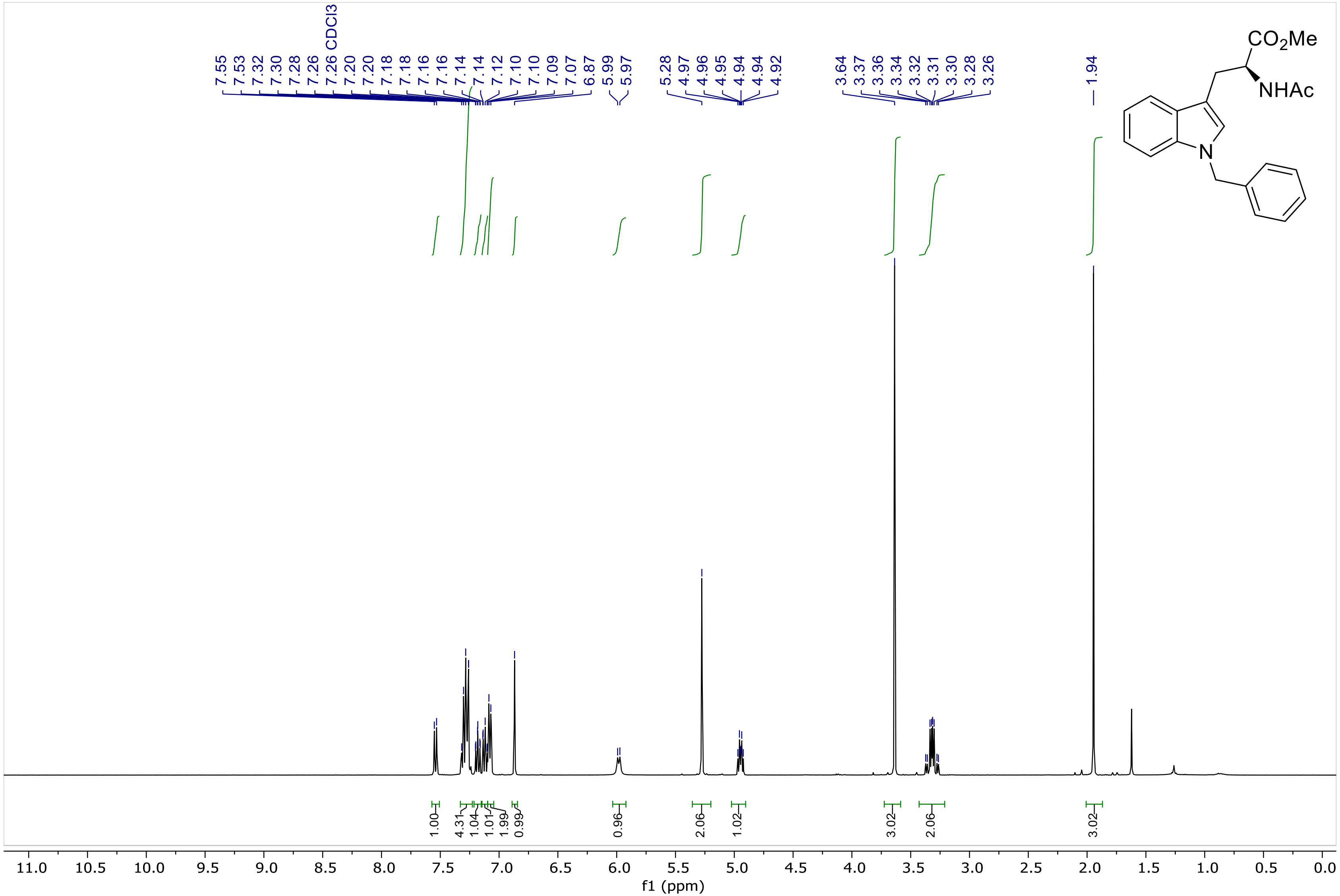

1-Benzyl-N^{α}-Acetyl-*L*-tryptophan methyl ester – $^{13}C\{^1H\}$ NMR (101 MHz, $CDCl_3$)

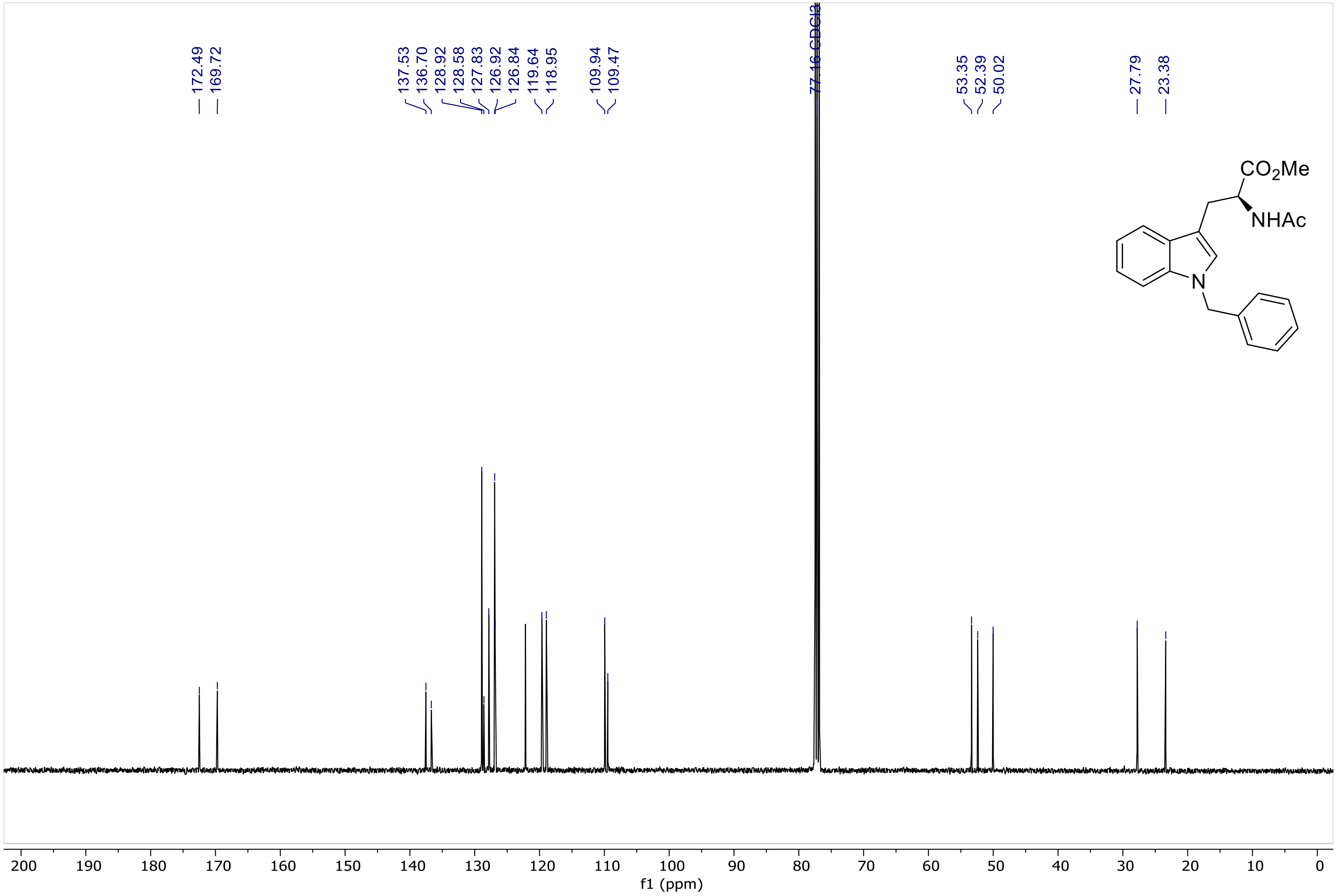

Methyl N$^{\alpha}$-acetyl-1-benzyl-*L*-tryptophylglycinate– ^{1}H NMR (400 MHz, DMSO-d$_6$)

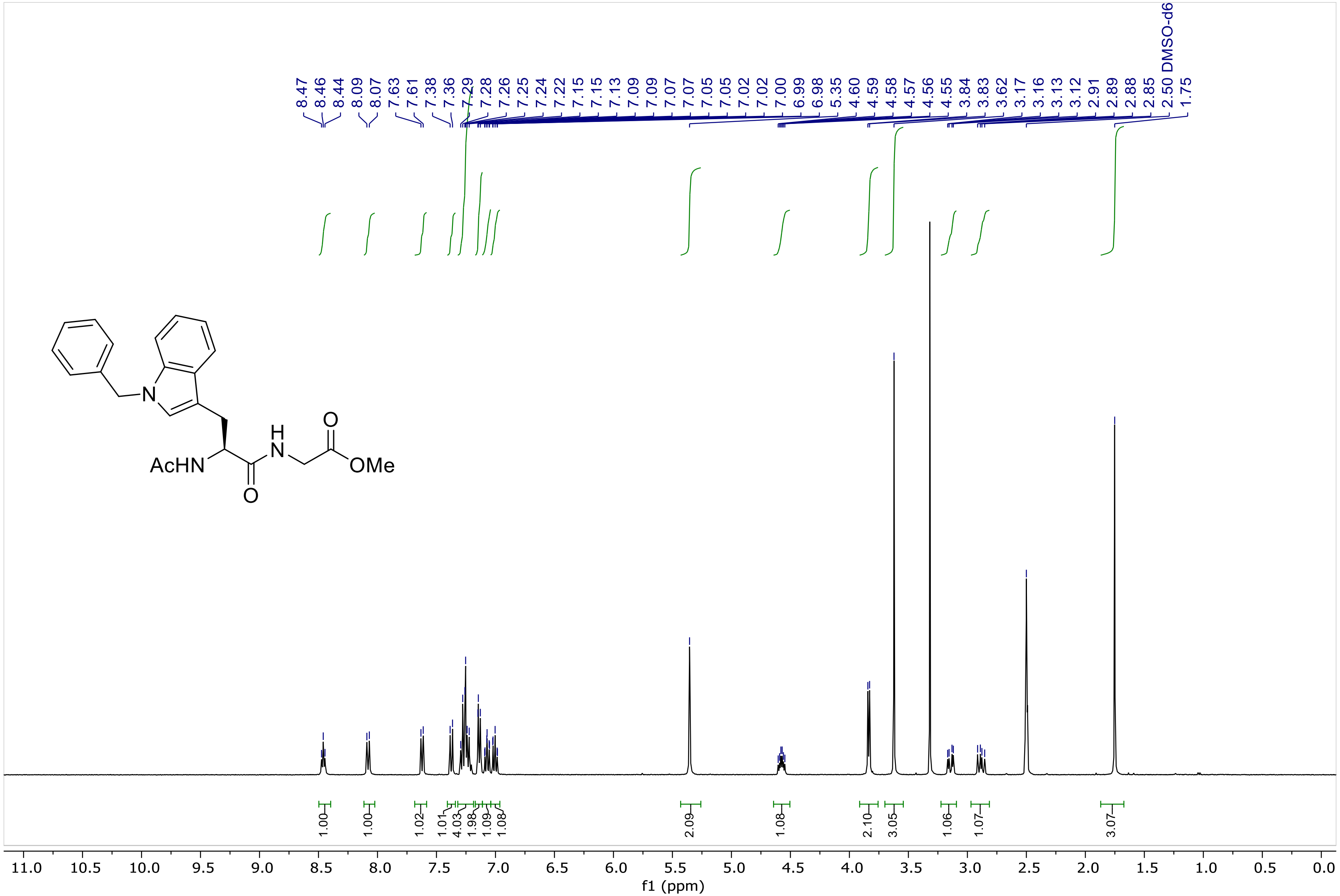

Methyl N$^{\alpha}$-acetyl-1-benzyl-*L*-tryptophylglycinate– $^{13}C\{^{1}H\}$ NMR (101 MHz, DMSO-d$_6$)

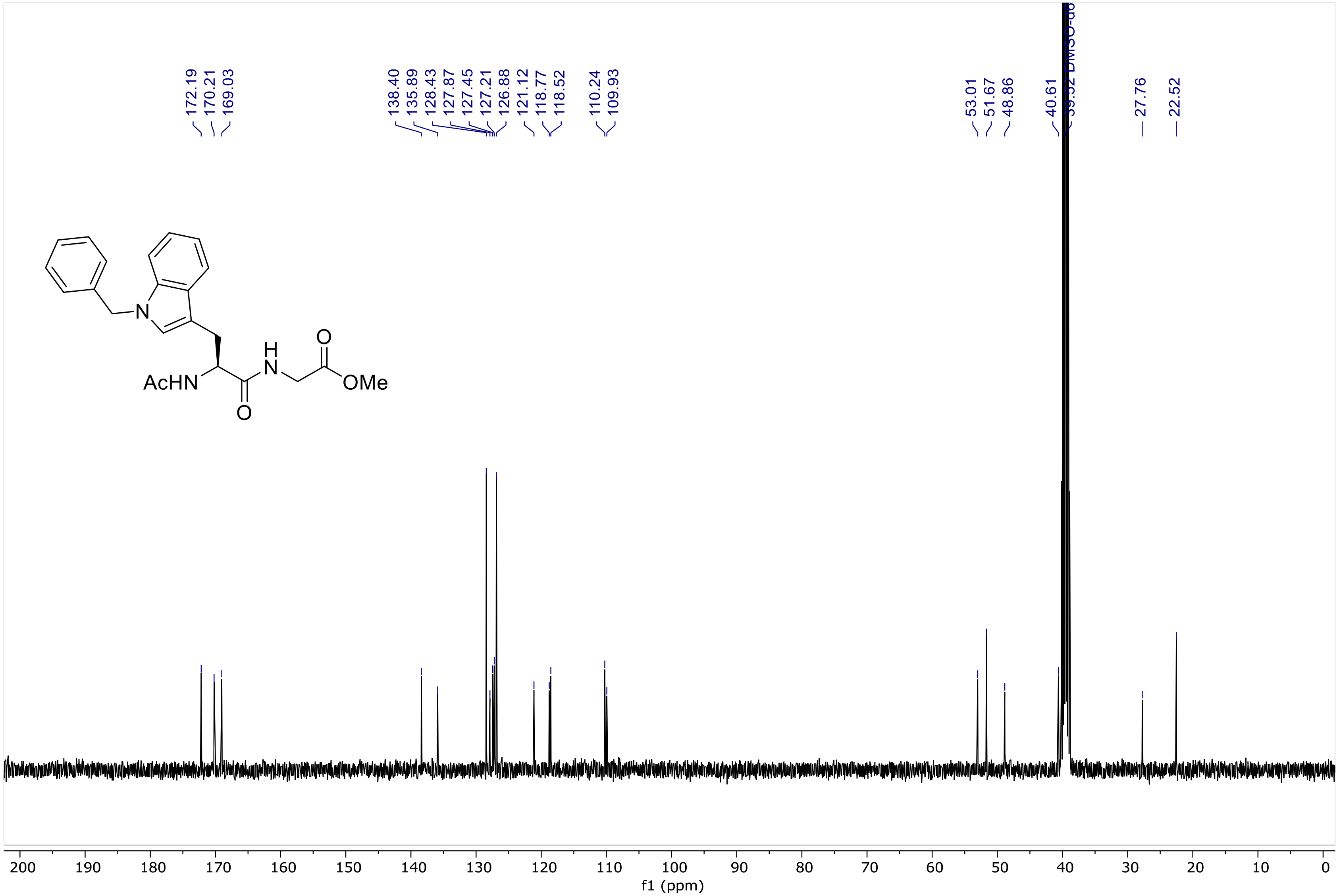

1-Benzylpyrrole – ^{1}H NMR (400 MHz, $CDCl_3$)

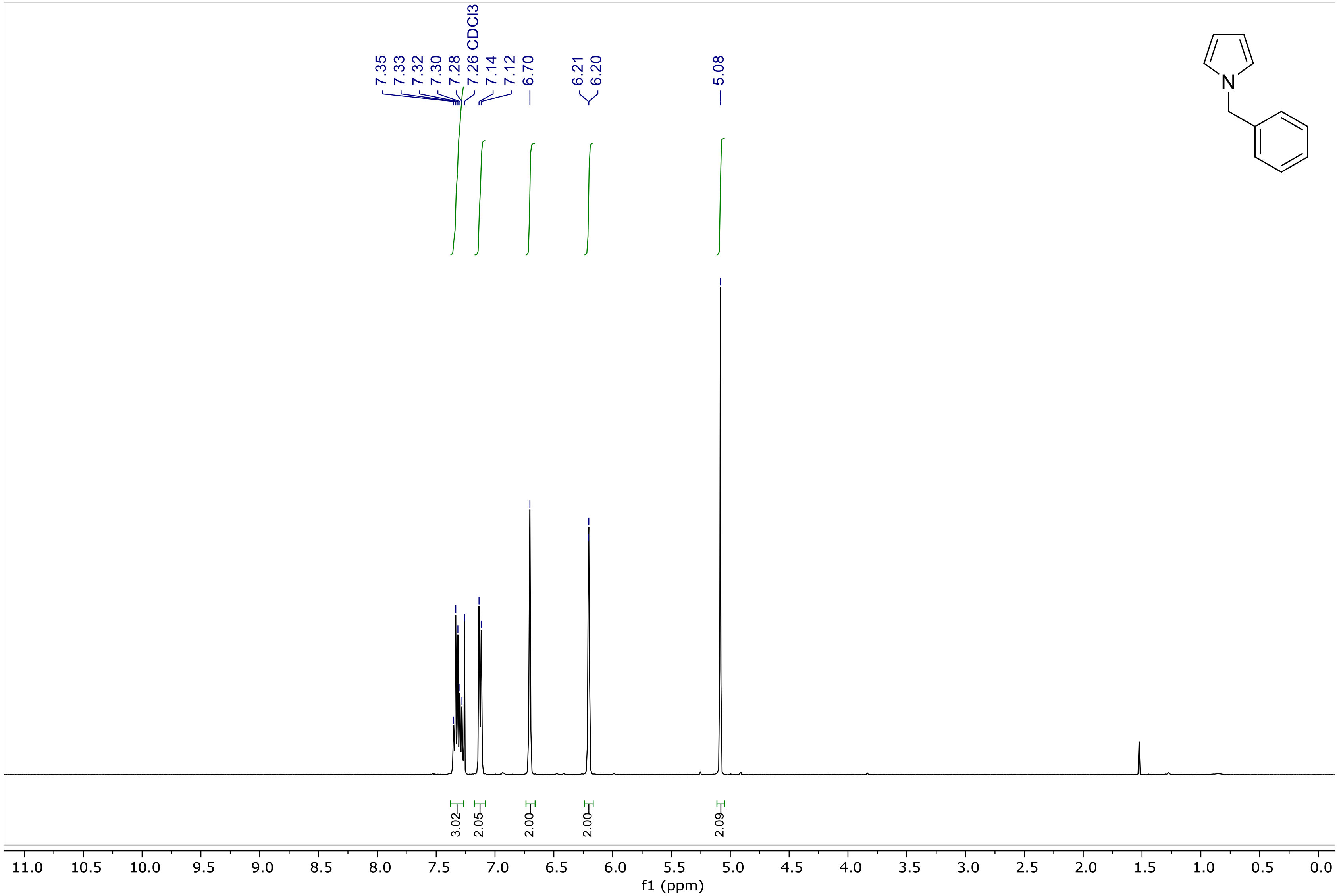

1-Benzylpyrrole – $^{13}C\{^{1}H\}$ NMR (101 MHz, $CDCl_3$)

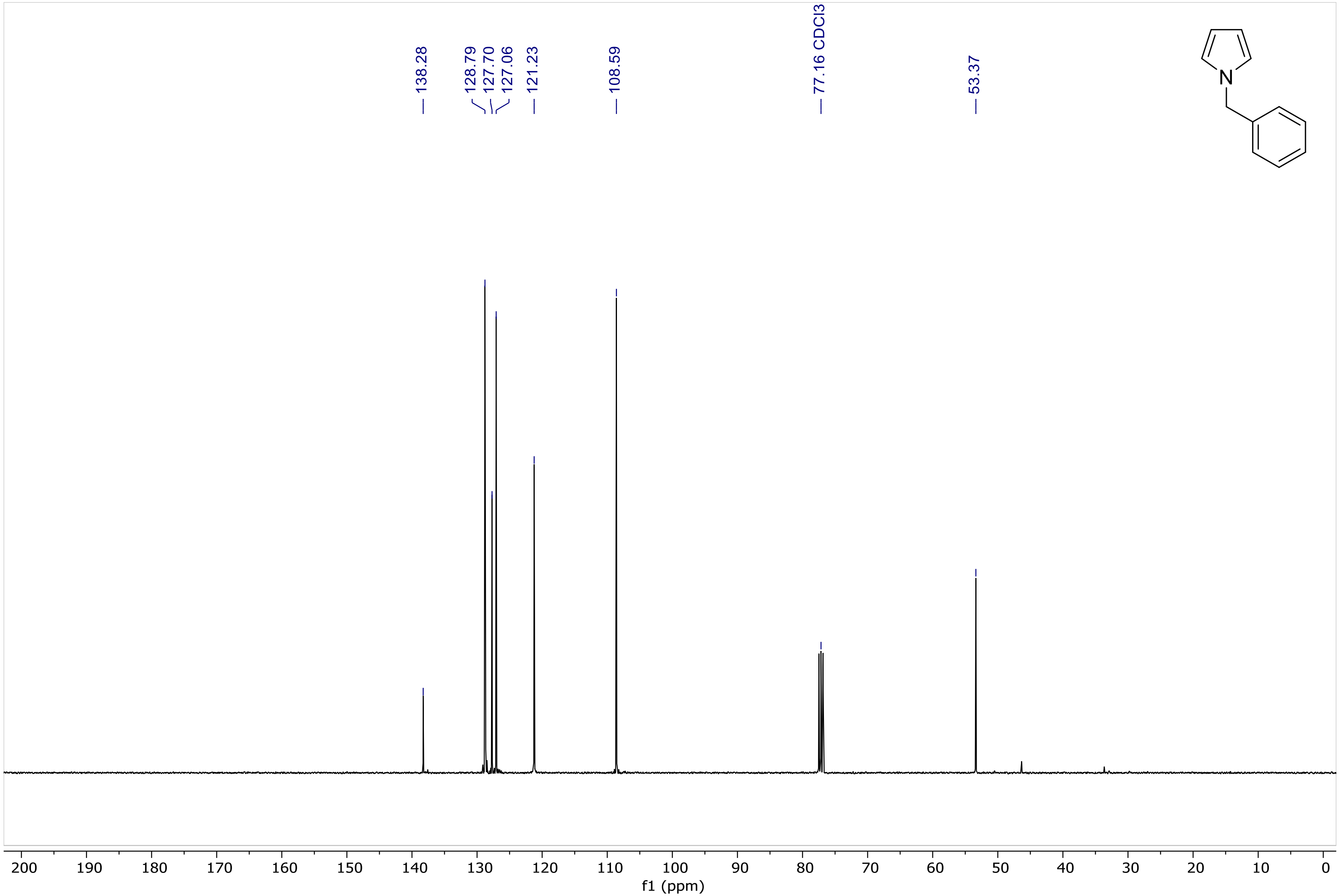

1-Benzyl-2,5-dimethylpyrrole – ^{1}H NMR (400 MHz, $CDCl_3$)

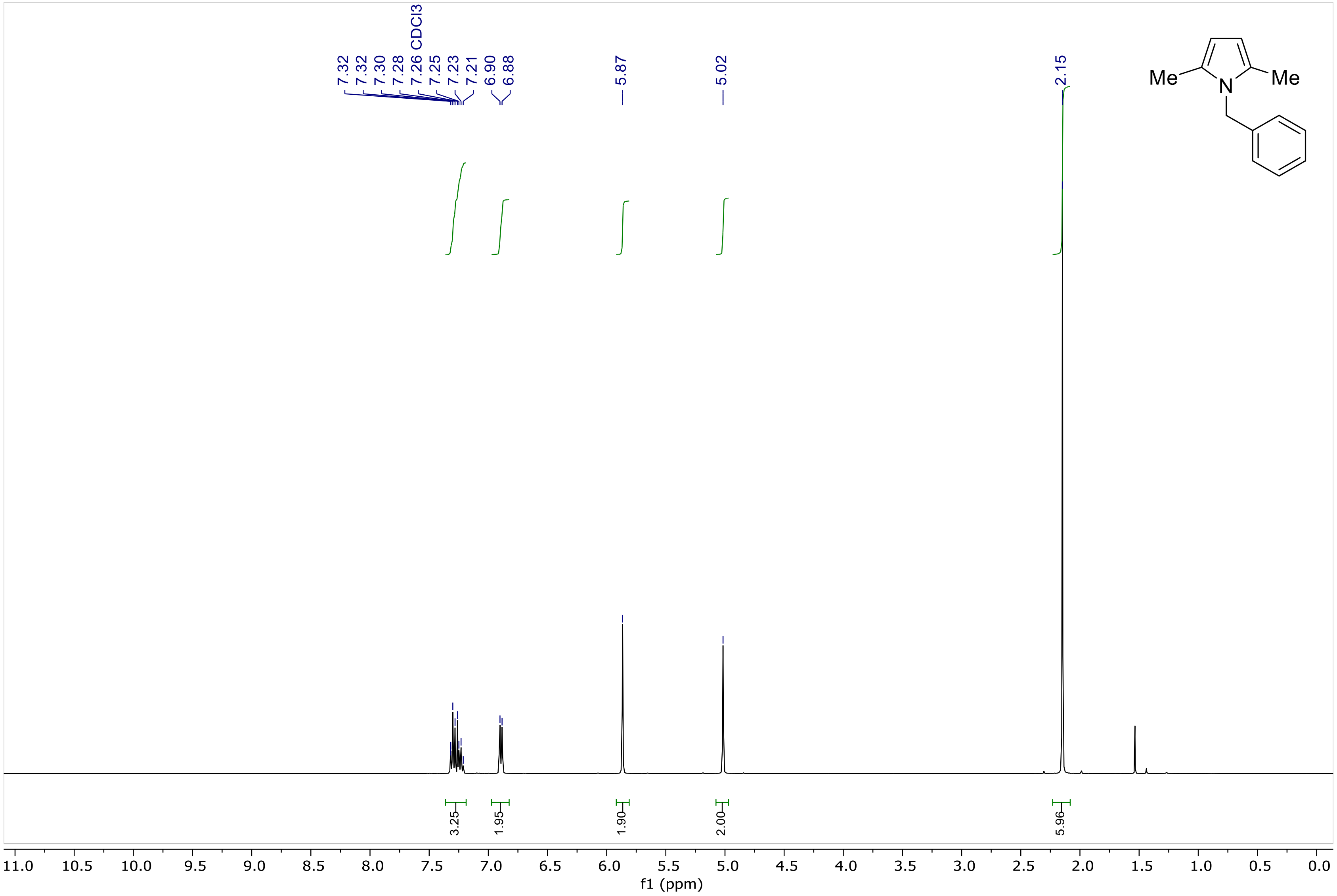

1-Benzyl-2,5-dimethylpyrrole – $^{13}C\{^{1}H\}$ NMR (101 MHz, $CDCl_3$)

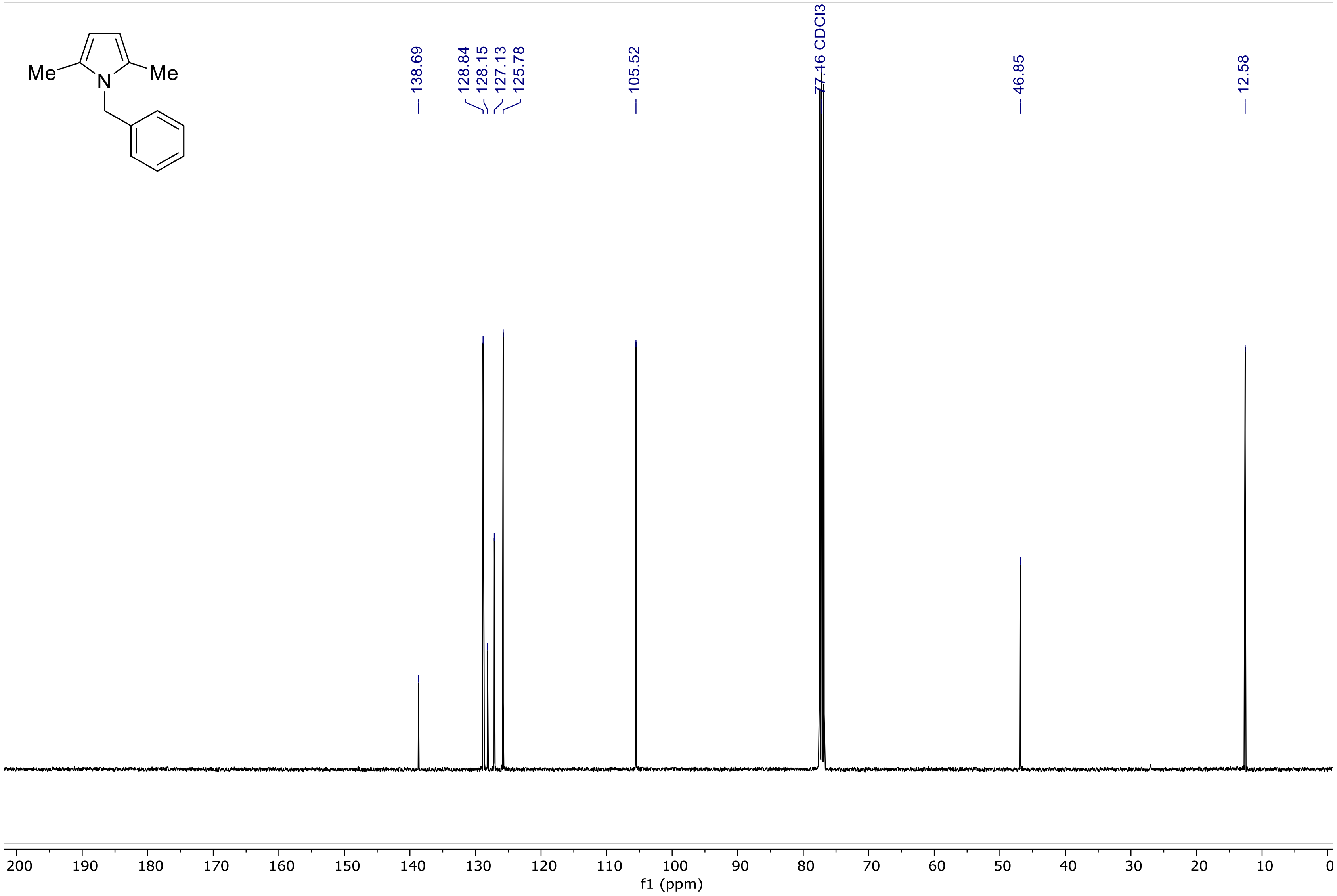

Pyrrole-3-methyl ester – ^{1}H NMR (400 MHz, $CDCl_3$)

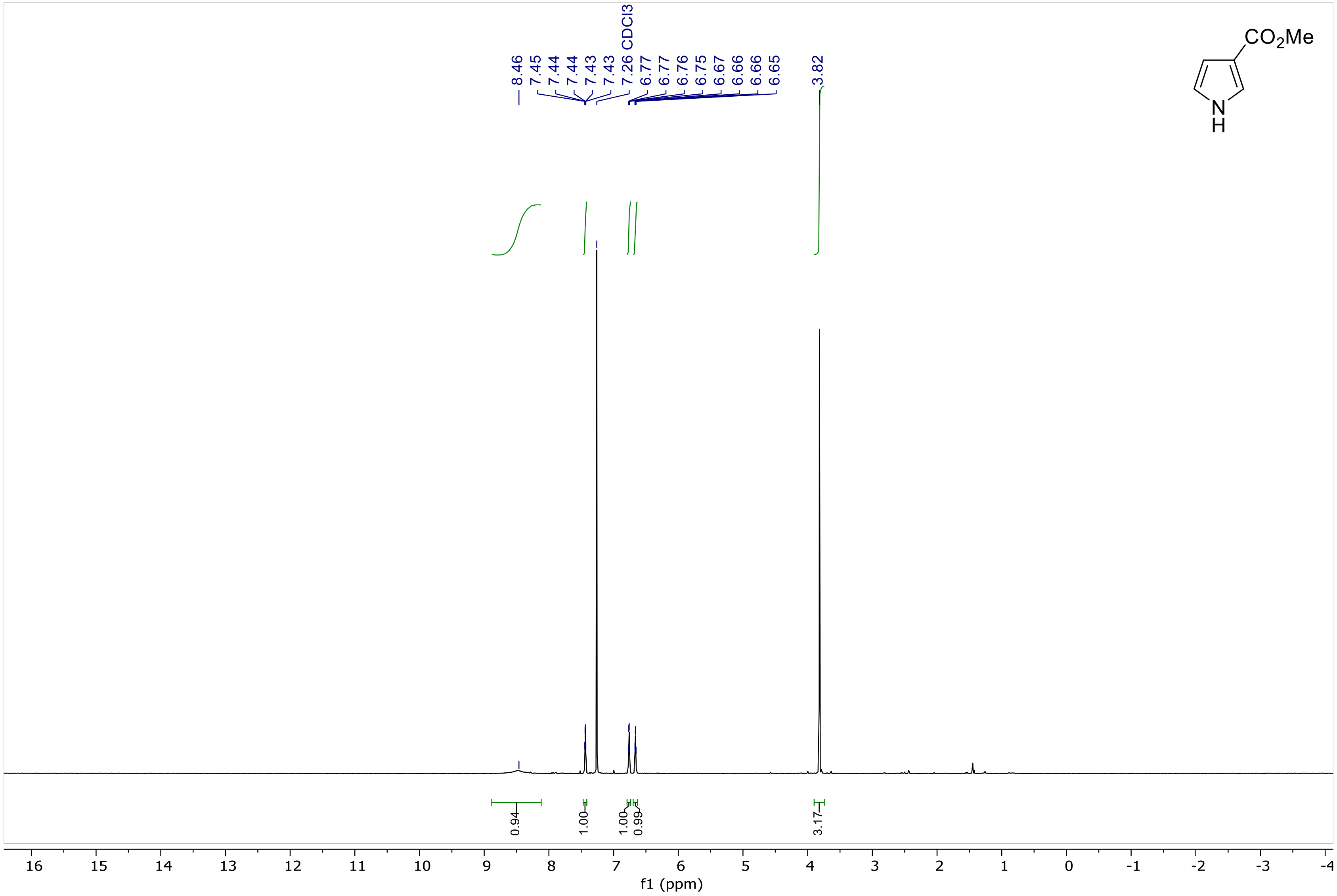

1-Benzylpyrrole-3-methyl ester – ^{1}H NMR (400 MHz, $CDCl_3$)

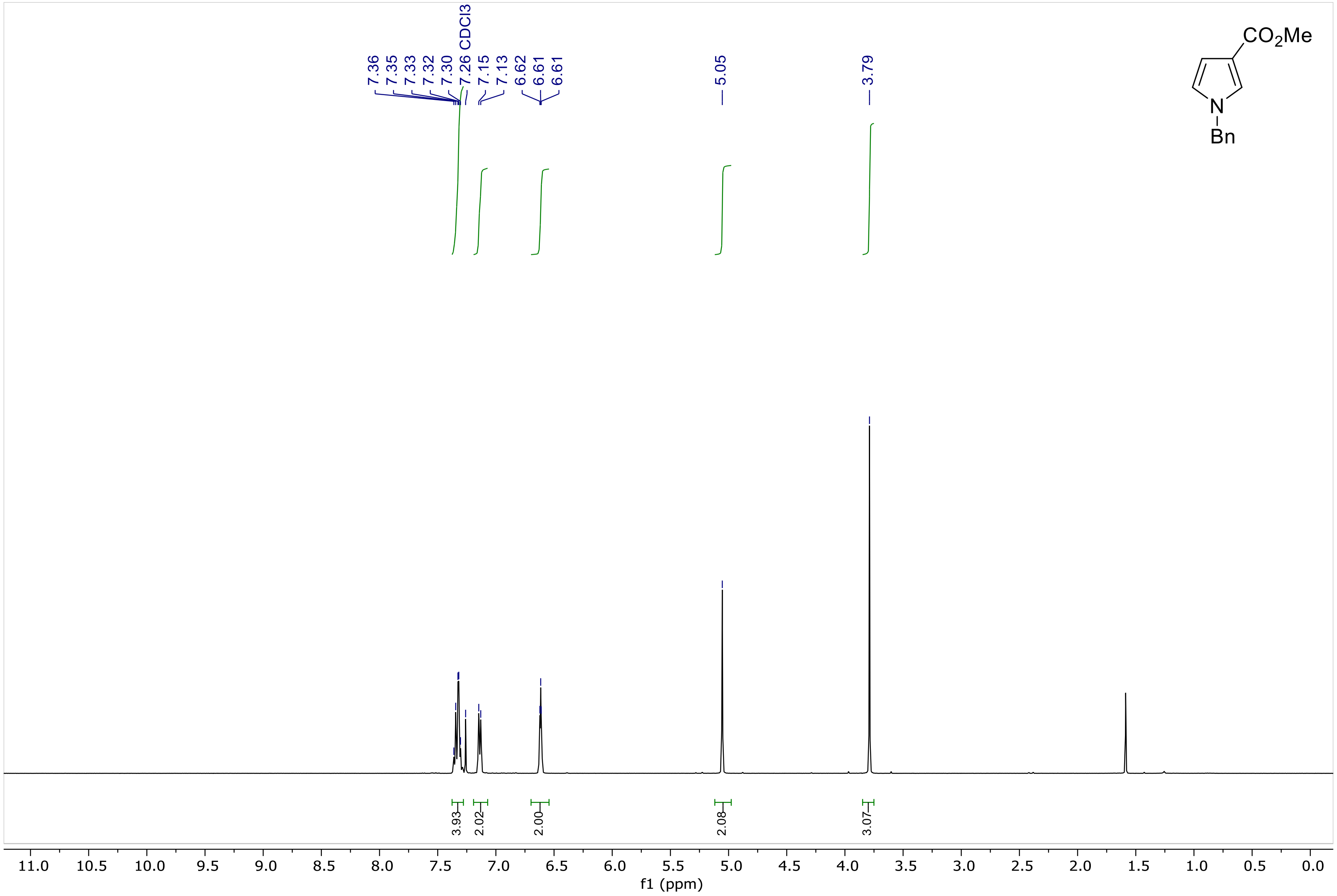

1-Benzylpyrrole-3-methyl ester – ^{1}H NMR (400 MHz, $CDCl_3$)

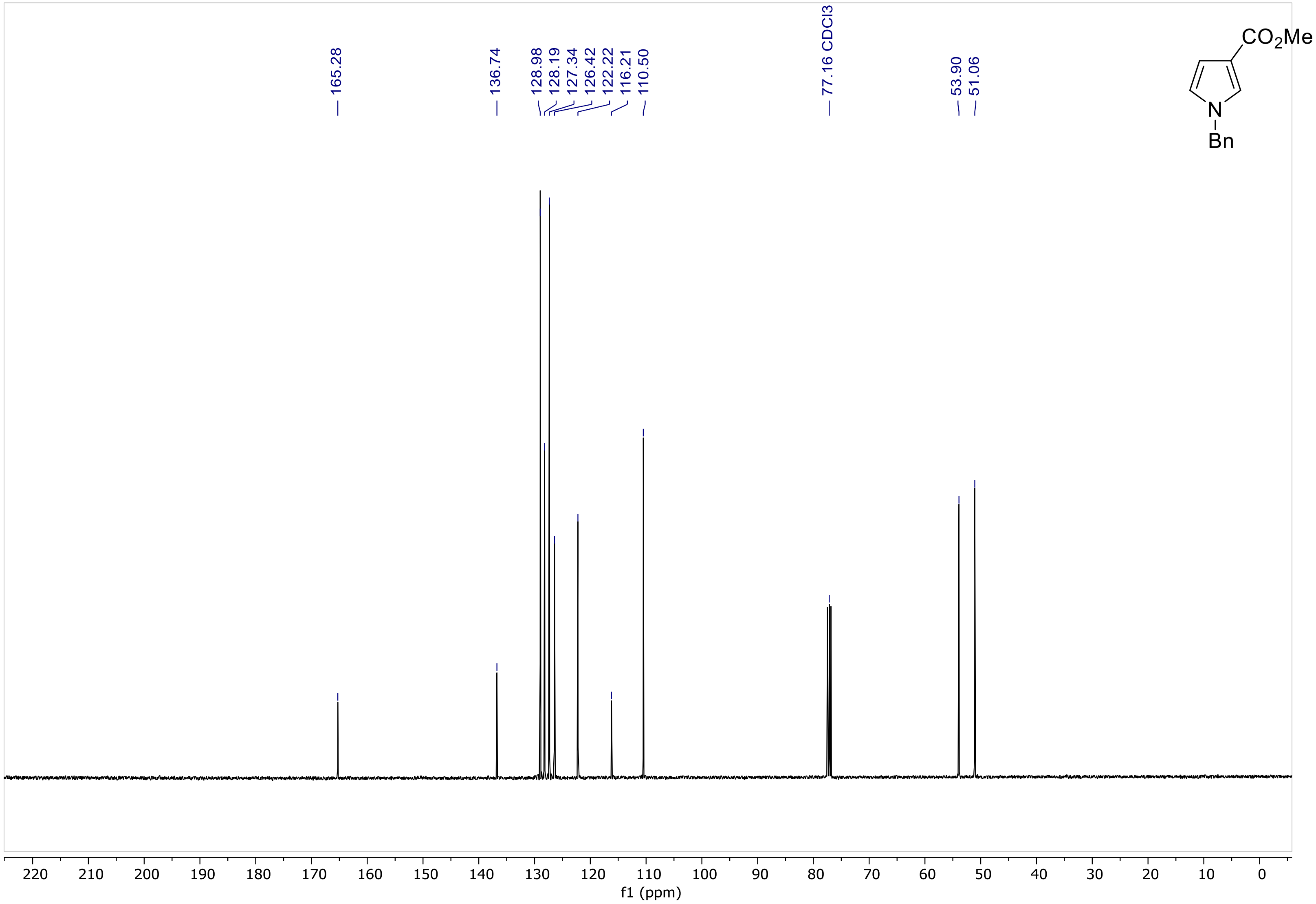

2,5,6,7-Tetrahydro-4*H*-isoindol-4-one – ^{1}H NMR (400 MHz, $CDCl_3$)

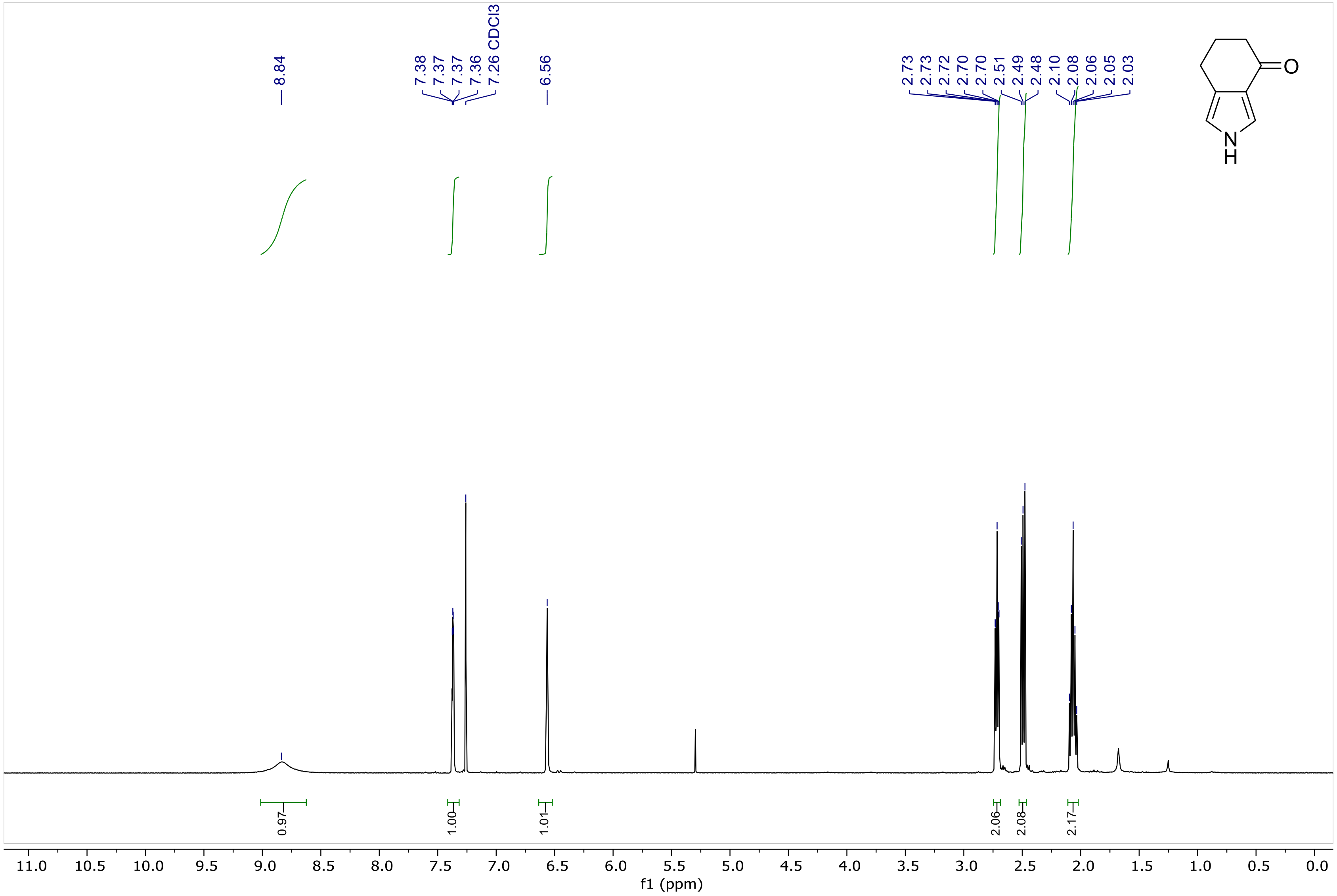

2,5,6,7-Tetrahydro-4*H*-isoindol-4-one– $^{13}C\{^{1}H\}$ NMR (101 MHz, CDCl$_3$)

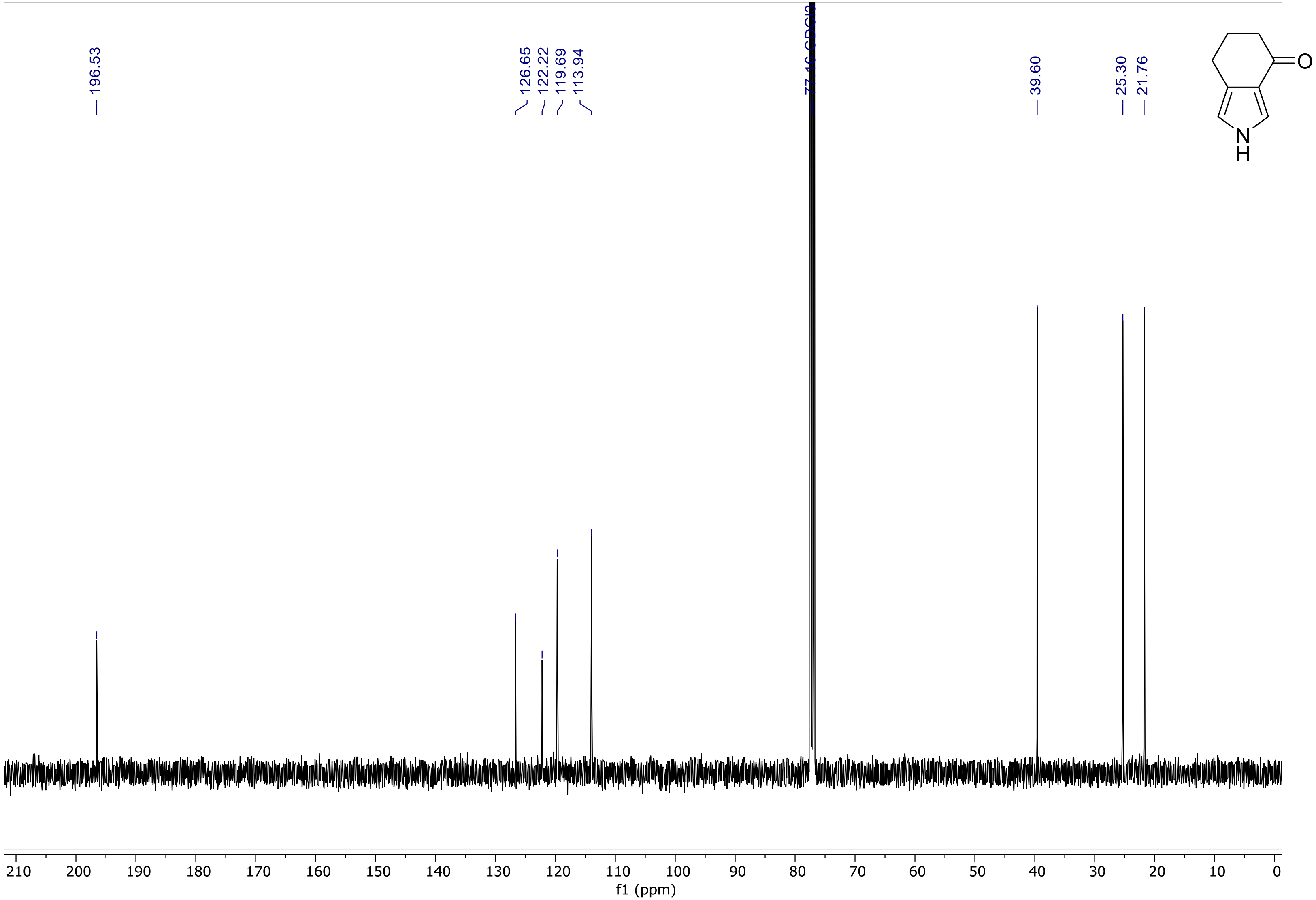

2-Benzyl-2,5,6,7-tetrahydro-4*H*-isoindol-4-one – ^{1}H NMR (400 MHz, $CDCl_3$)

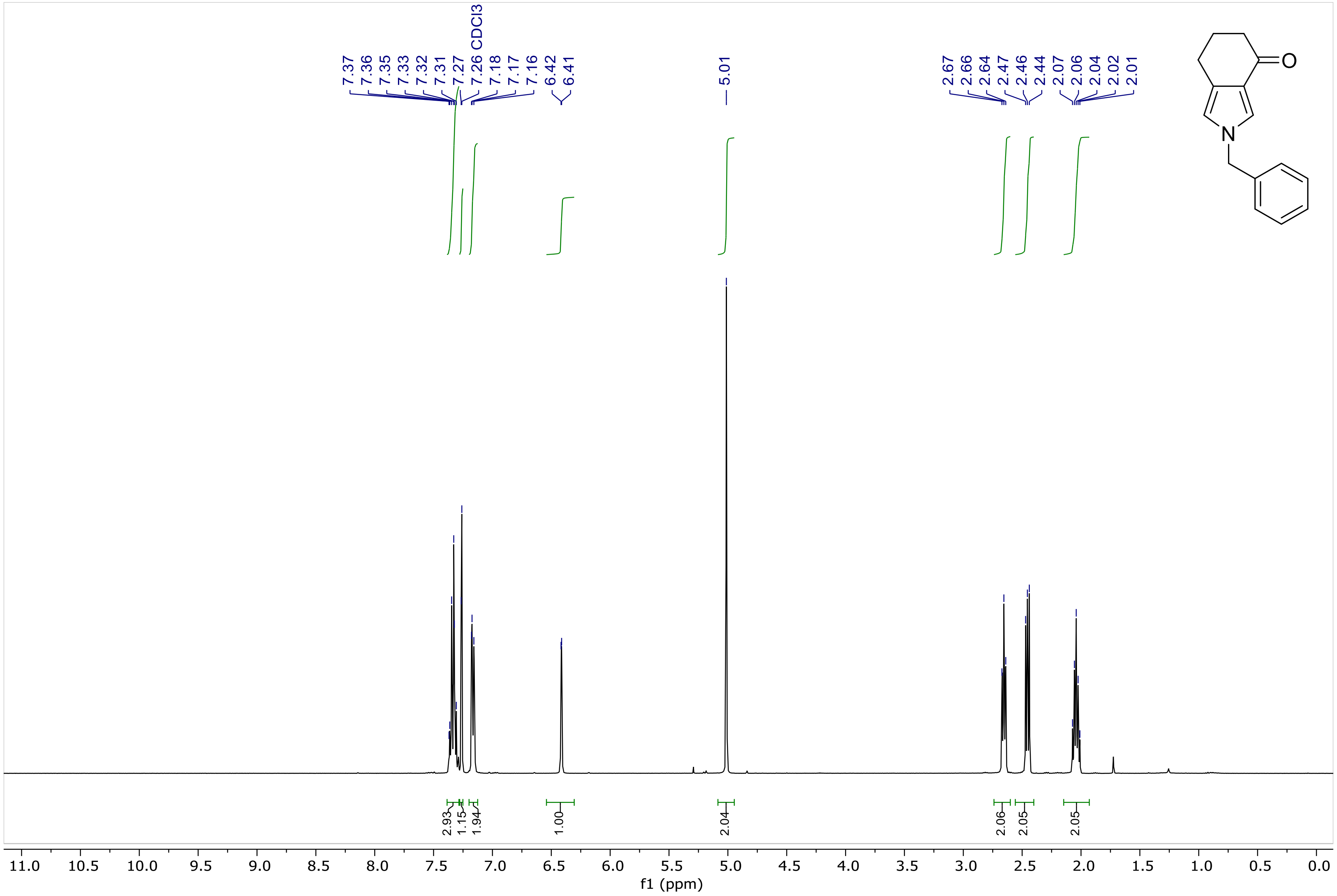

2-Benzyl-2,5,6,7-tetrahydro-4*H*-isoindol-4-one – $^{13}C\{^1H\}$ NMR (101 MHz, $CDCl_3$)

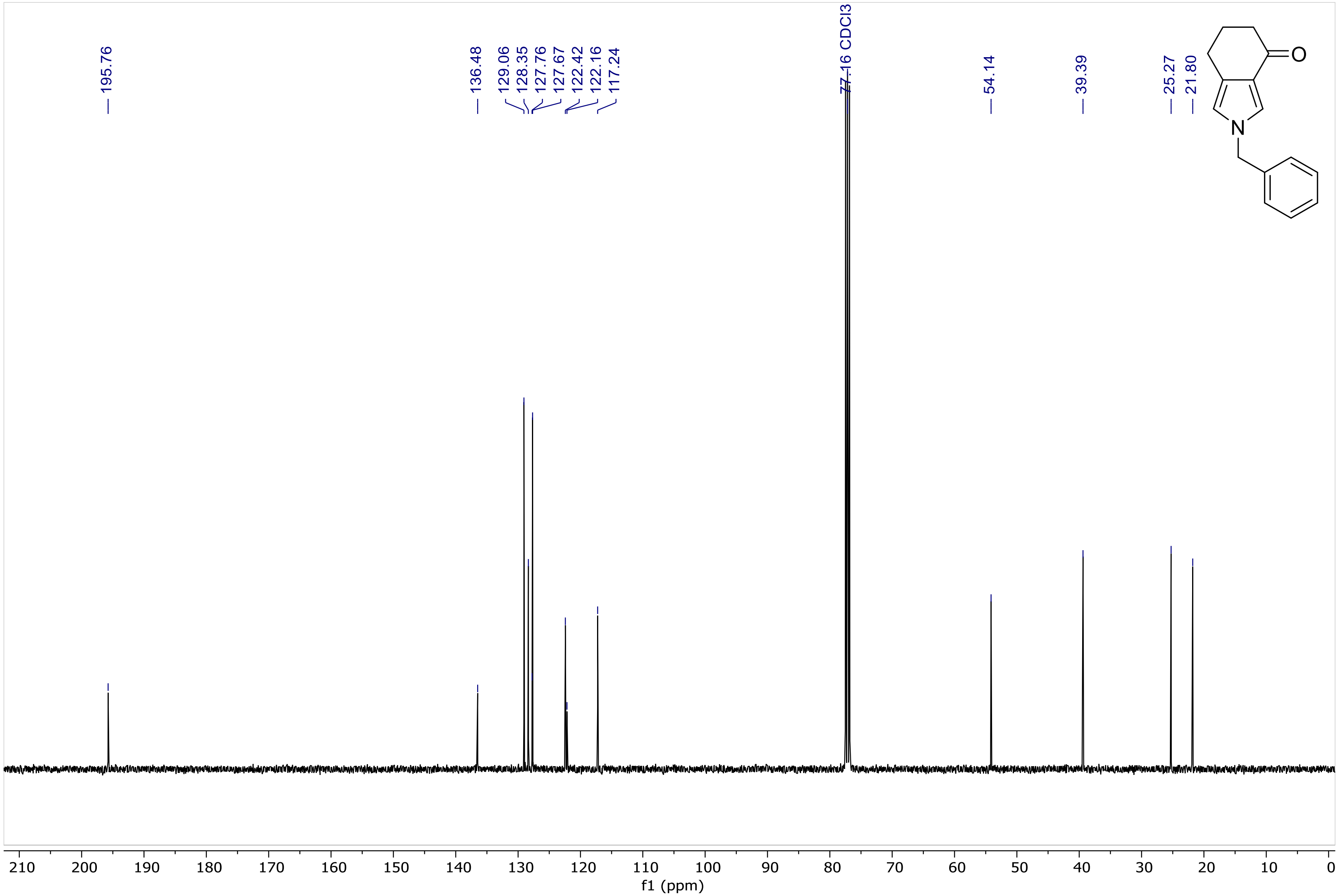

Methyl 4-phenyl-1*H*-pyrrole-3-carboxylate – ^{1}H NMR (400 MHz, $CDCl_3$)

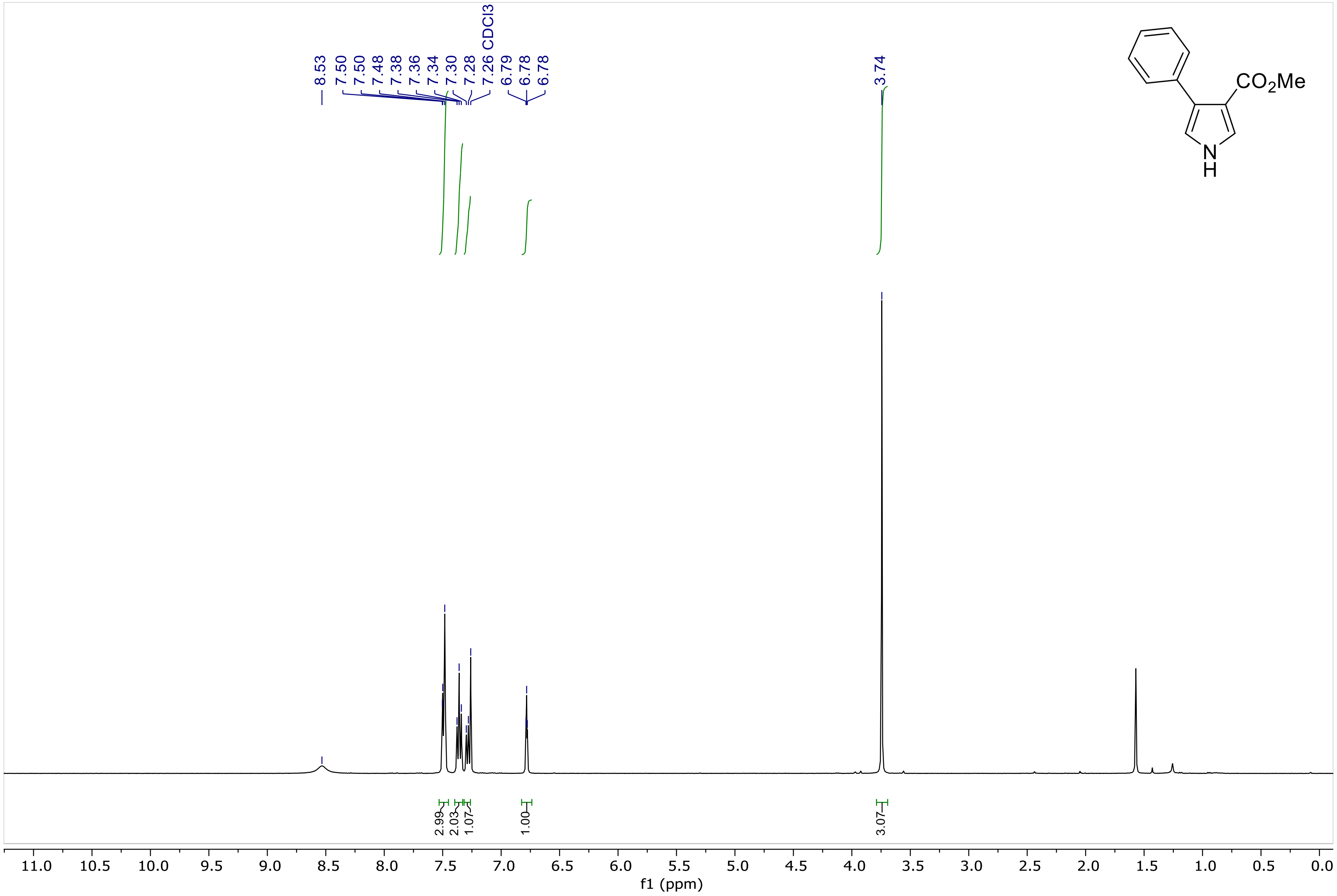

Methyl 4-phenyl-1*H*-pyrrole-3-carboxylate – $^{13}C\{^1H\}$ NMR (101 MHz, $CDCl_3$)

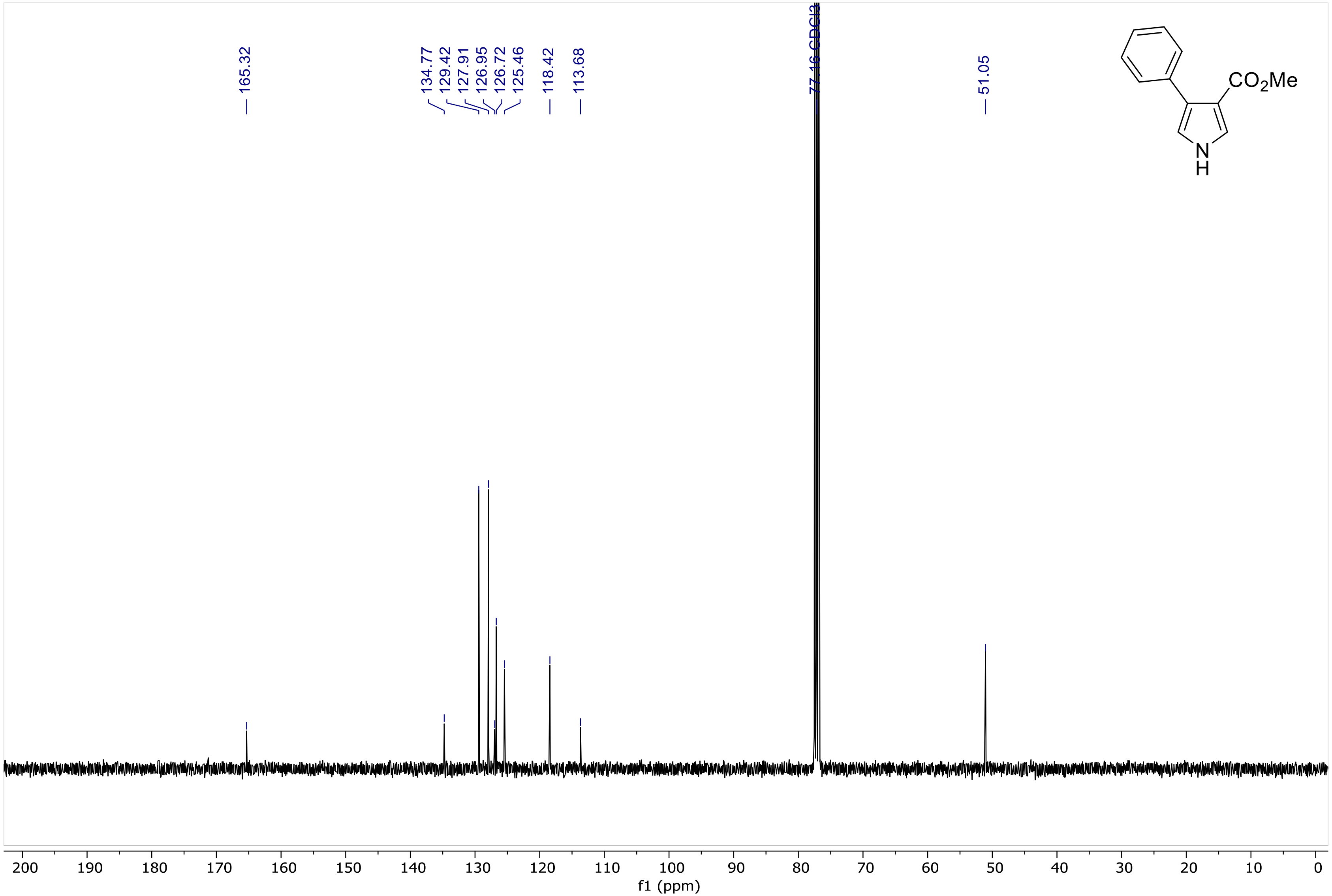

Methyl 2-benzyl-4-phenyl-1*H*-pyrrole-3-carboxylate – ^{1}H NMR (400 MHz, $CDCl_3$)

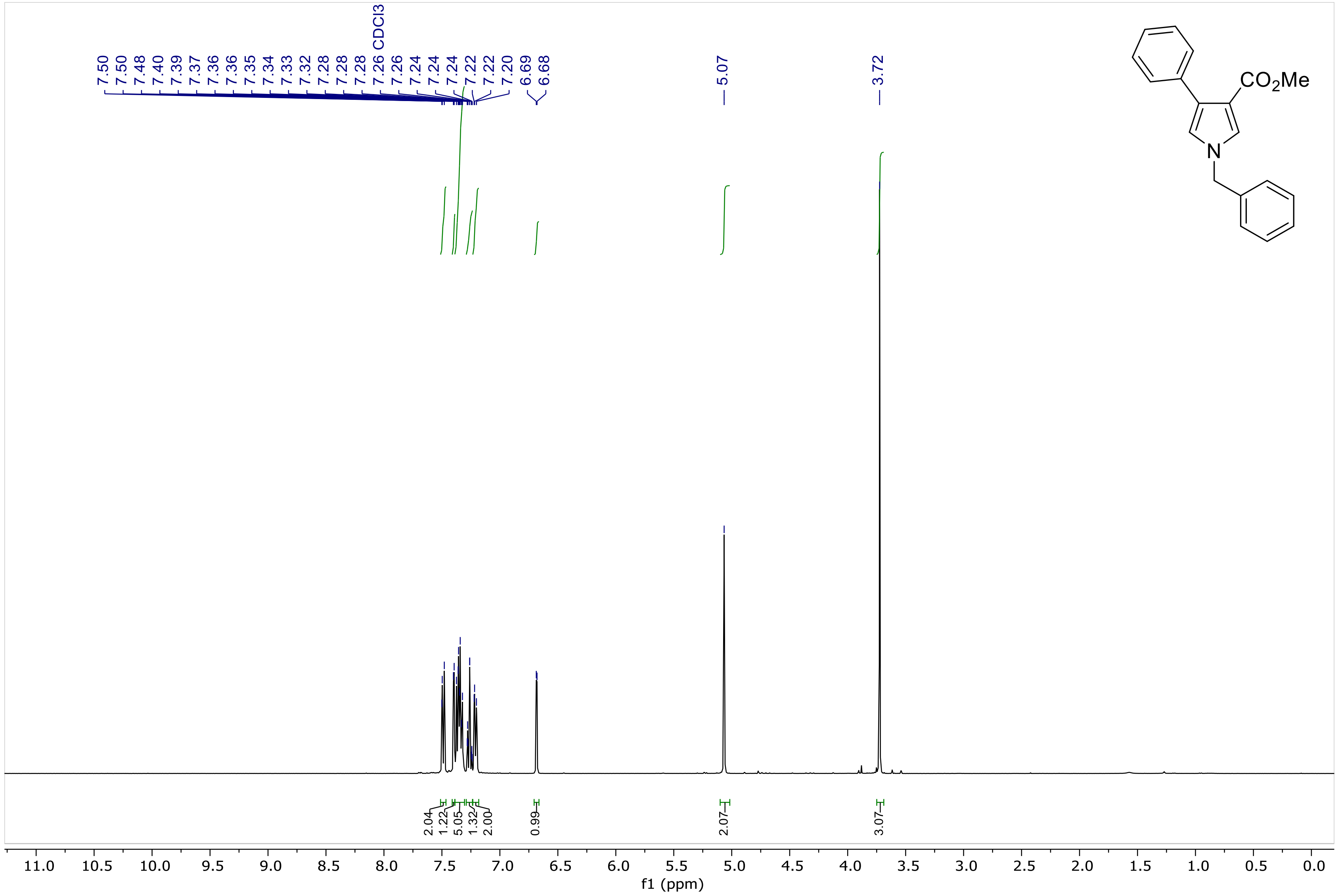

Methyl 2-benzyl-4-phenyl-1*H*-pyrrole-3-carboxylate – $^{13}C\{^{1}H\}$ NMR (101 MHz, $CDCl_3$)

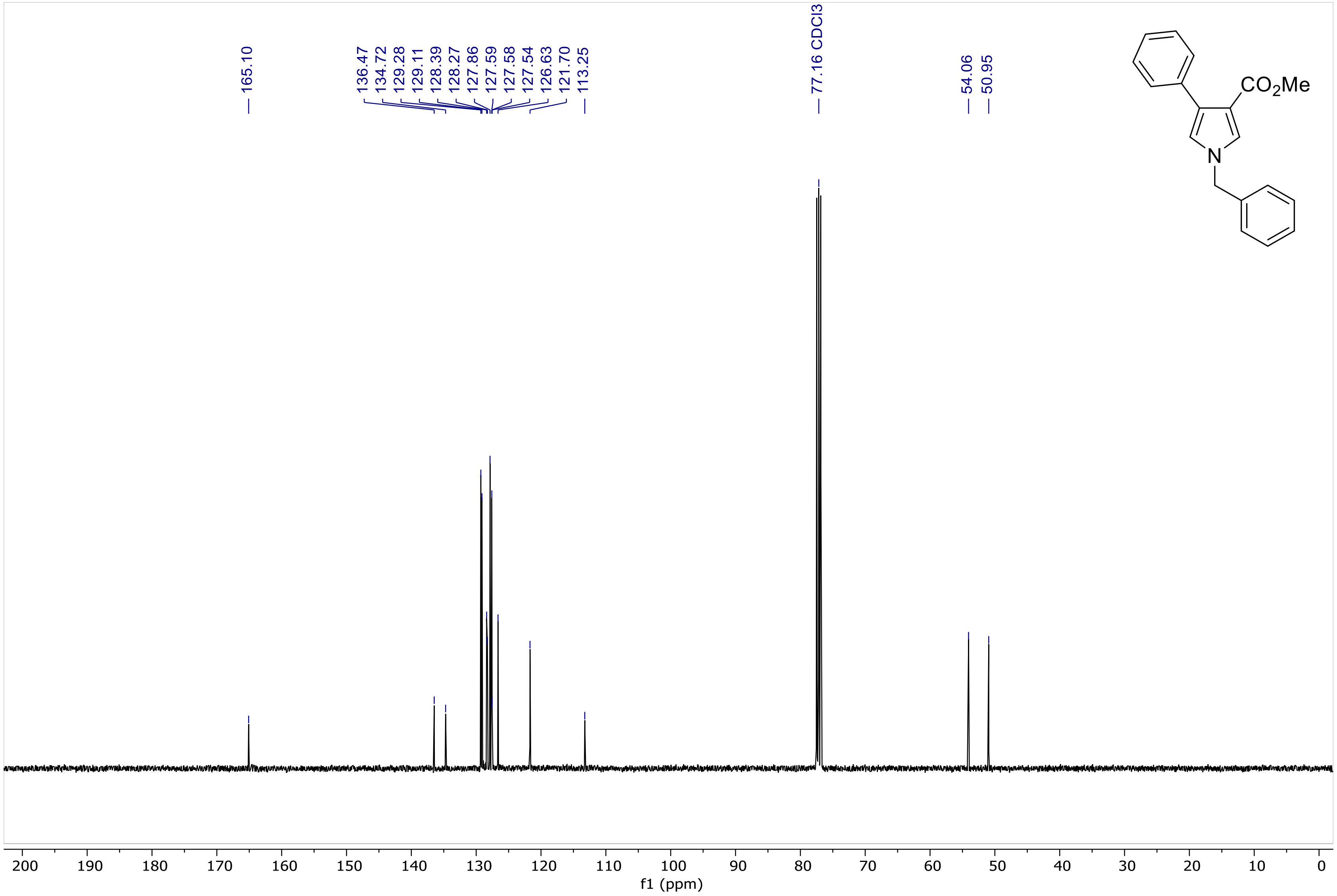

3-Phenylpyrrole – ^{1}H NMR (400 MHz, $CDCl_3$)

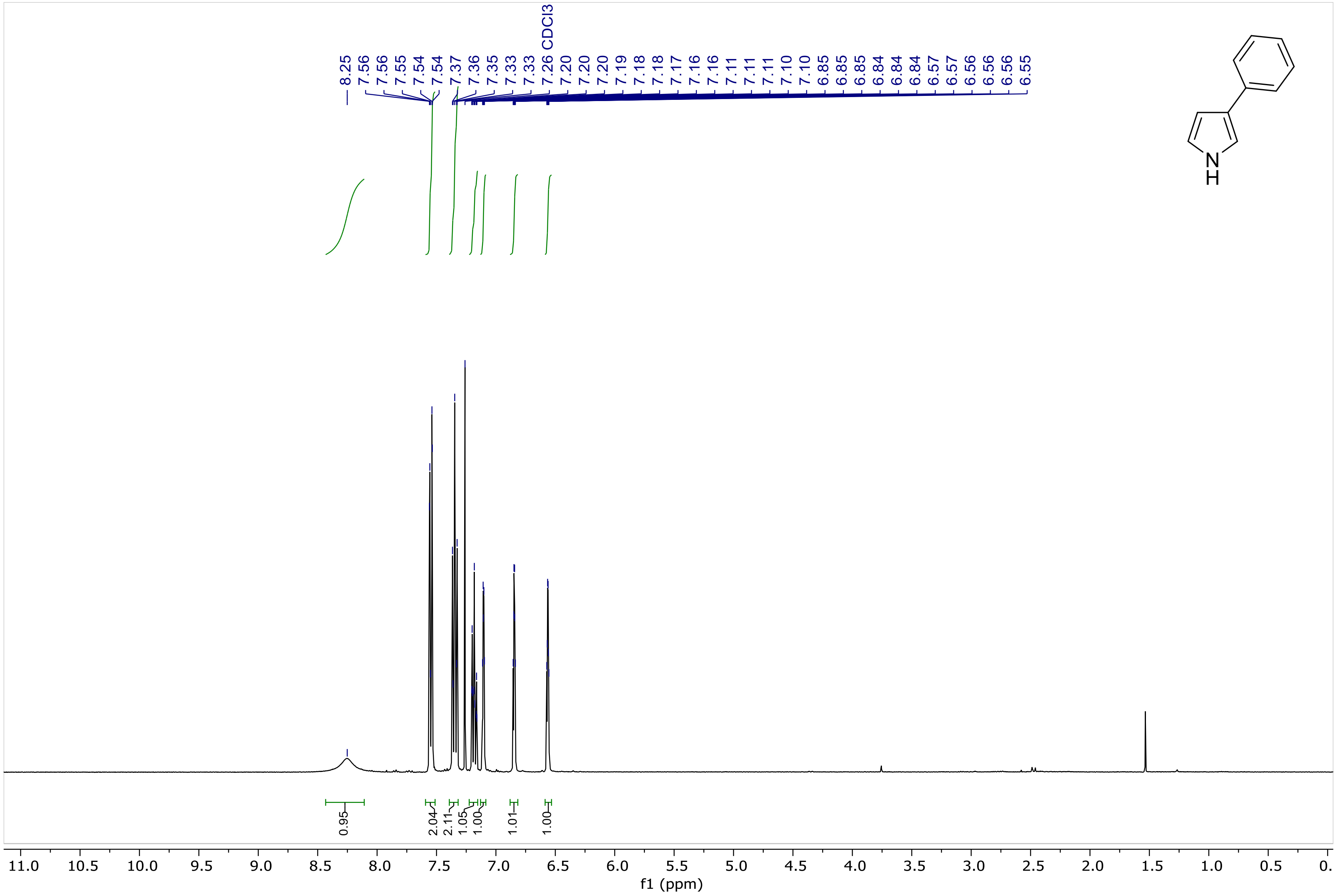

1-Benzyl-3-phenylpyrrole – ^{1}H NMR (400 MHz, $CDCl_3$)

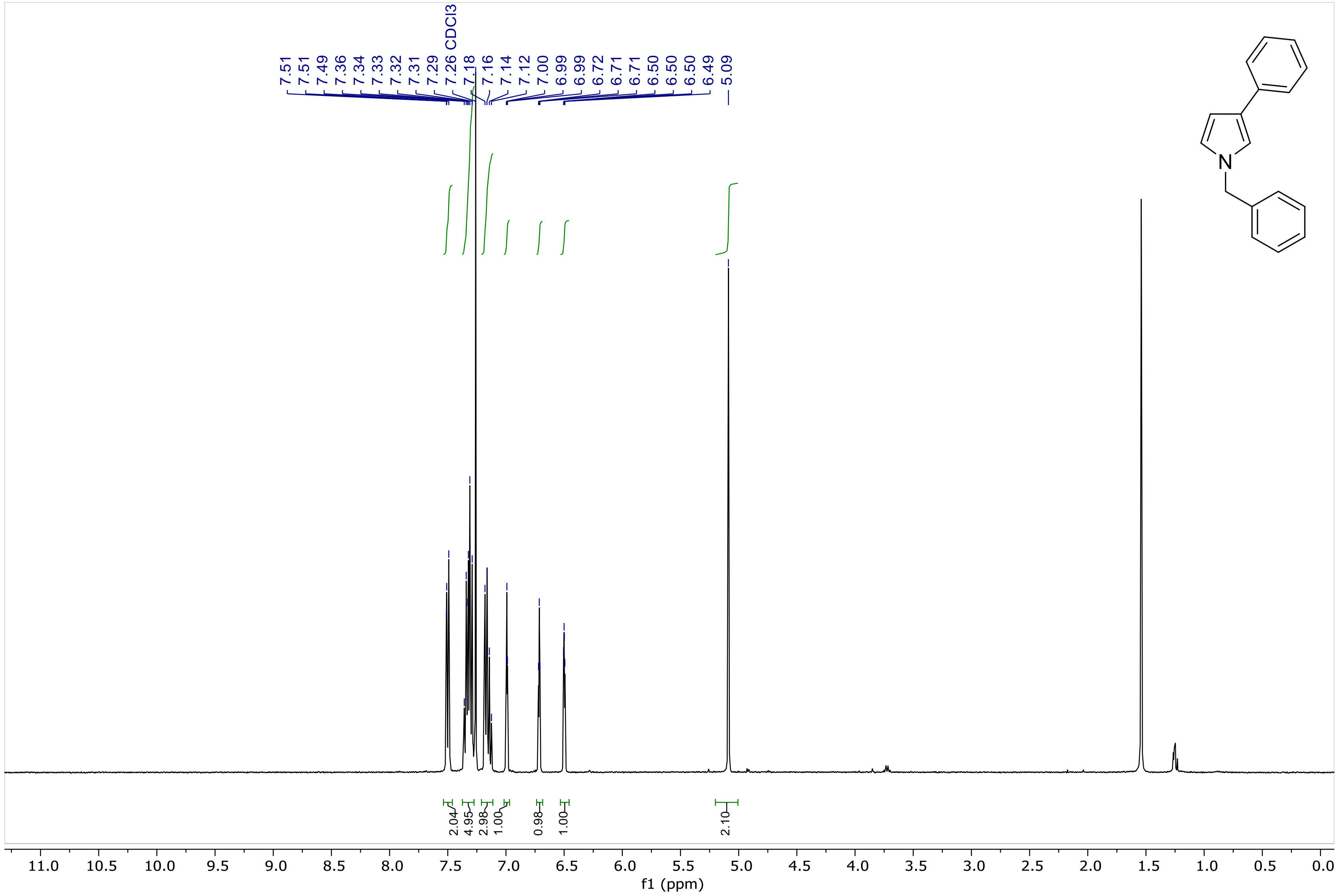

1-Benzyl-3-phenylpyrrole – $^{13}C\{^1H\}$ NMR (101 MHz, $CDCl_3$)

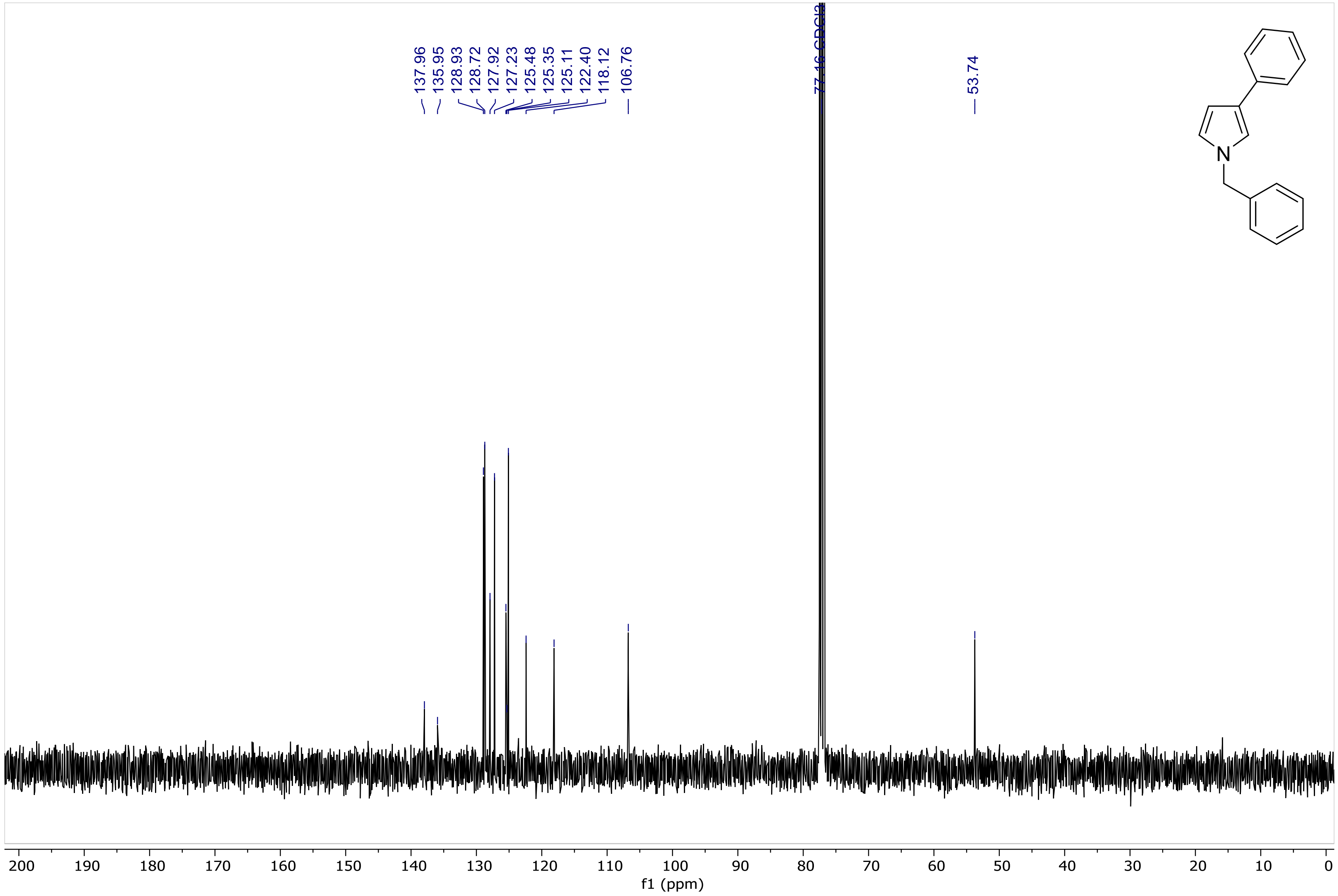

1-Benzyl-2-methylpyrrole – ^{1}H NMR (400 MHz, $CDCl_3$)

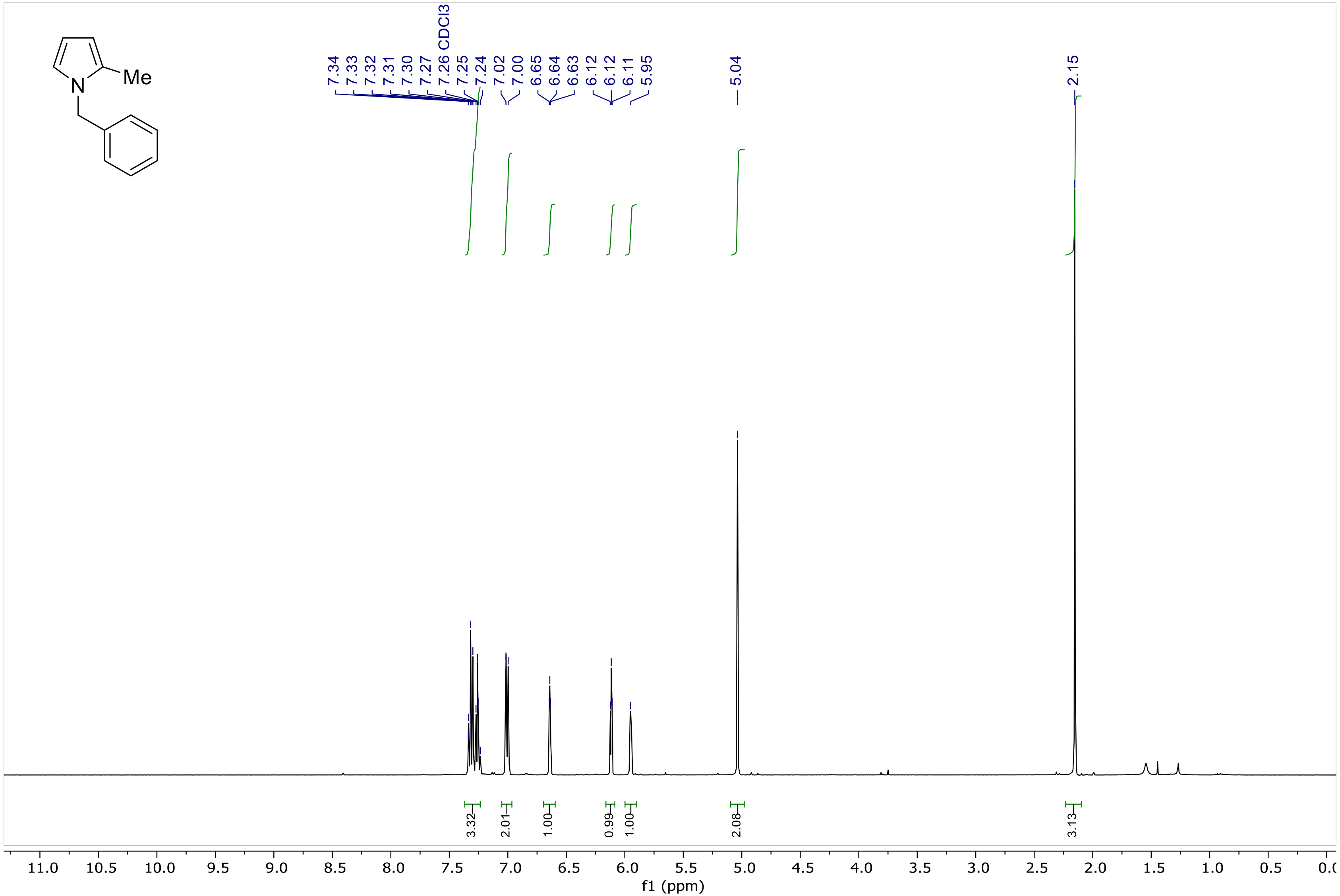

1-Benzyl-2-methylpyrrole – $^{13}C\{^1H\}$ NMR (101 MHz, $CDCl_3$)

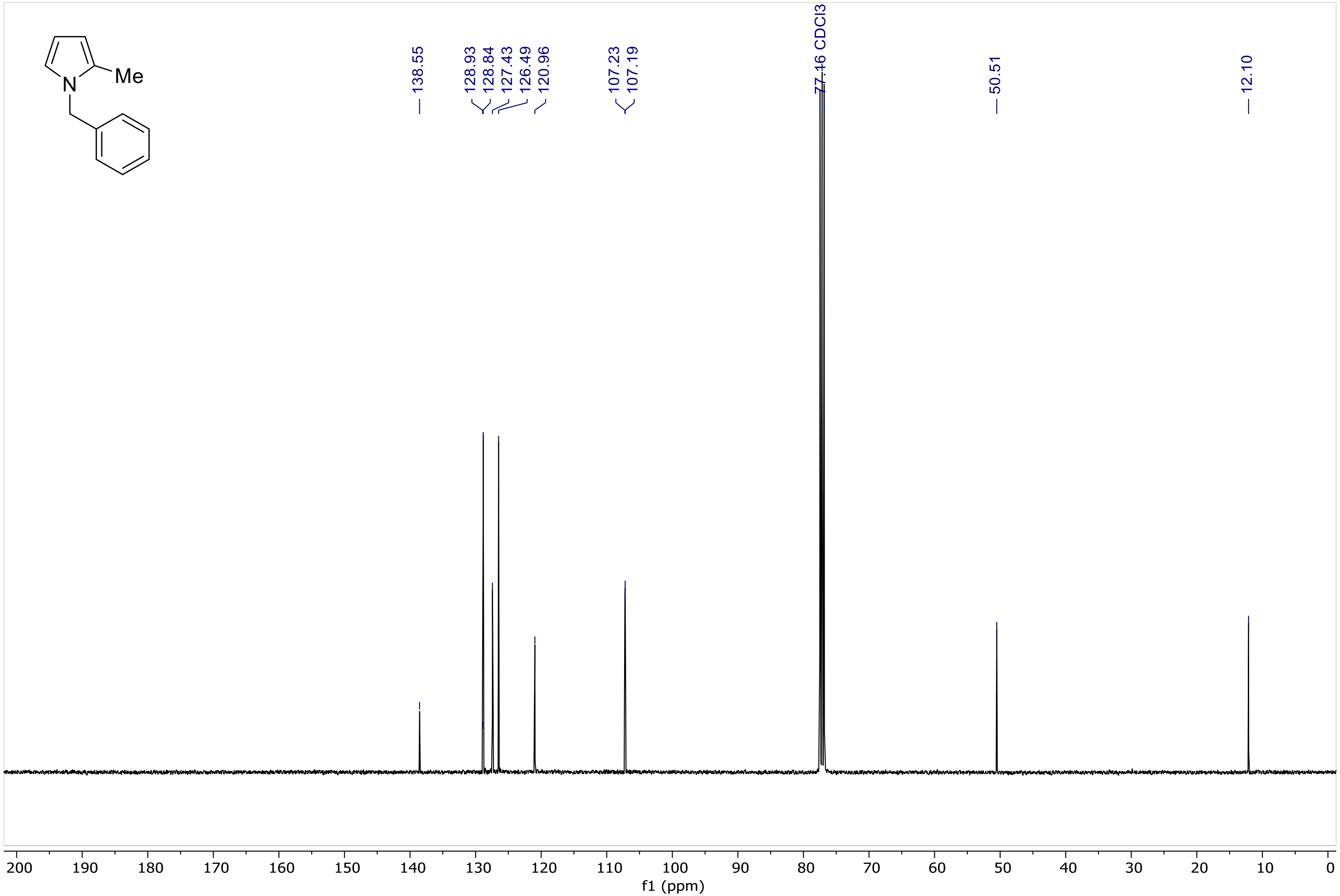

1-Benzyl-2-isopropylpyrrole – ^{1}H NMR (400 MHz, $CDCl_3$)

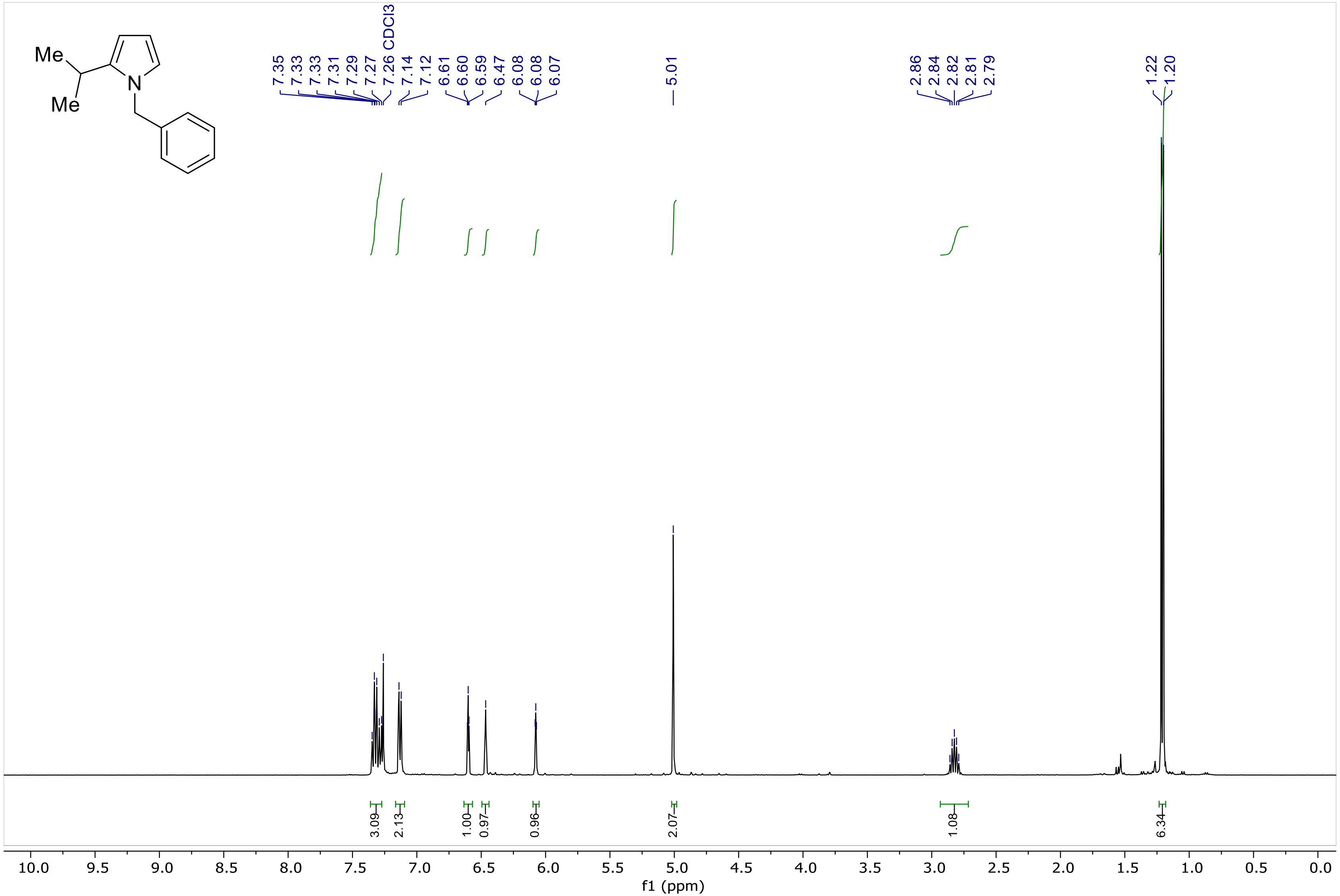

1-Benzyl-2-isopropylpyrrole – $^{13}C\{^1H\}$ NMR (101 MHz, $CDCl_3$)

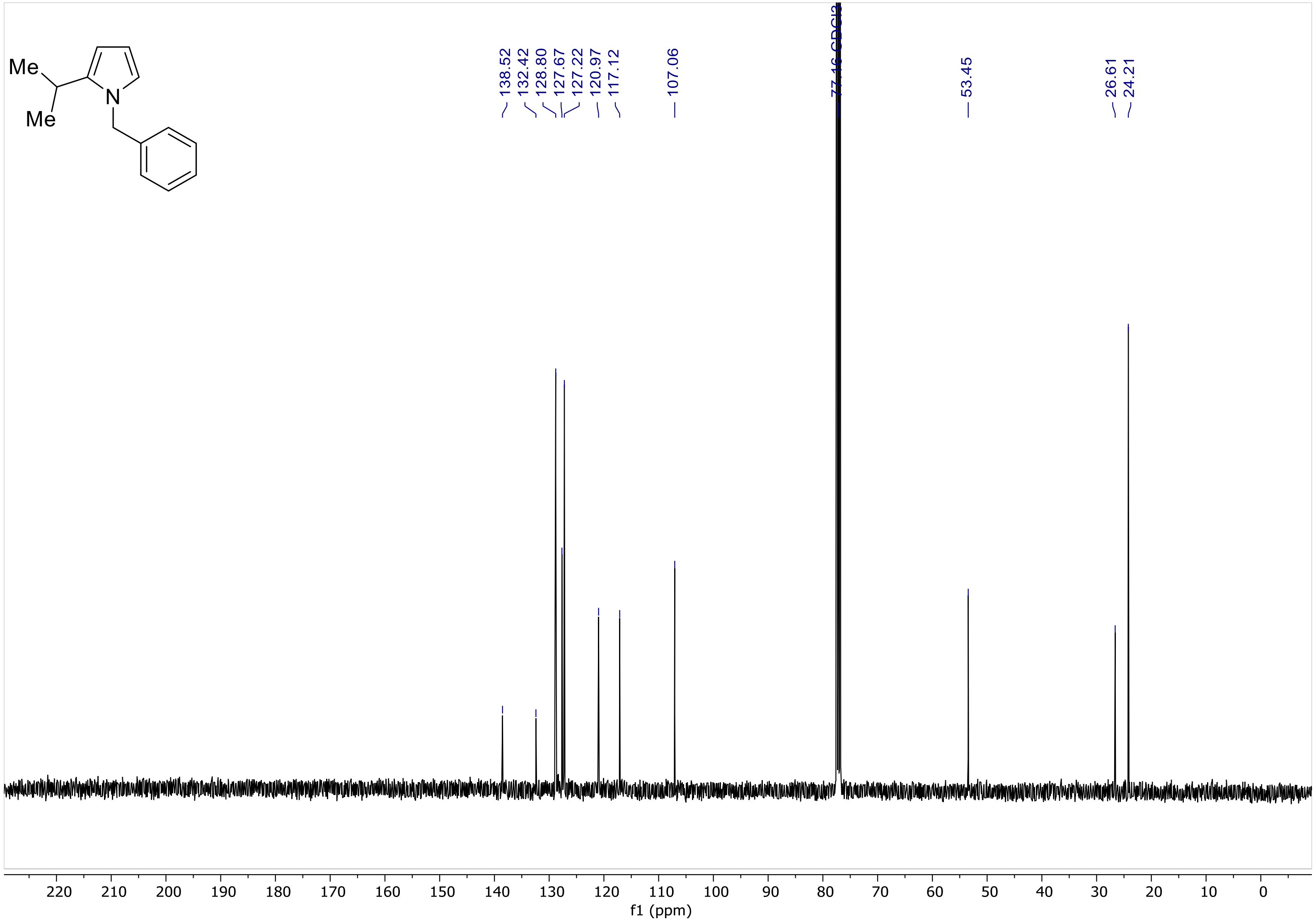

2-(1*H*-pyrrol-1-yl)ethan-1-amine – ^{1}H NMR (400 MHz, $CDCl_3$)

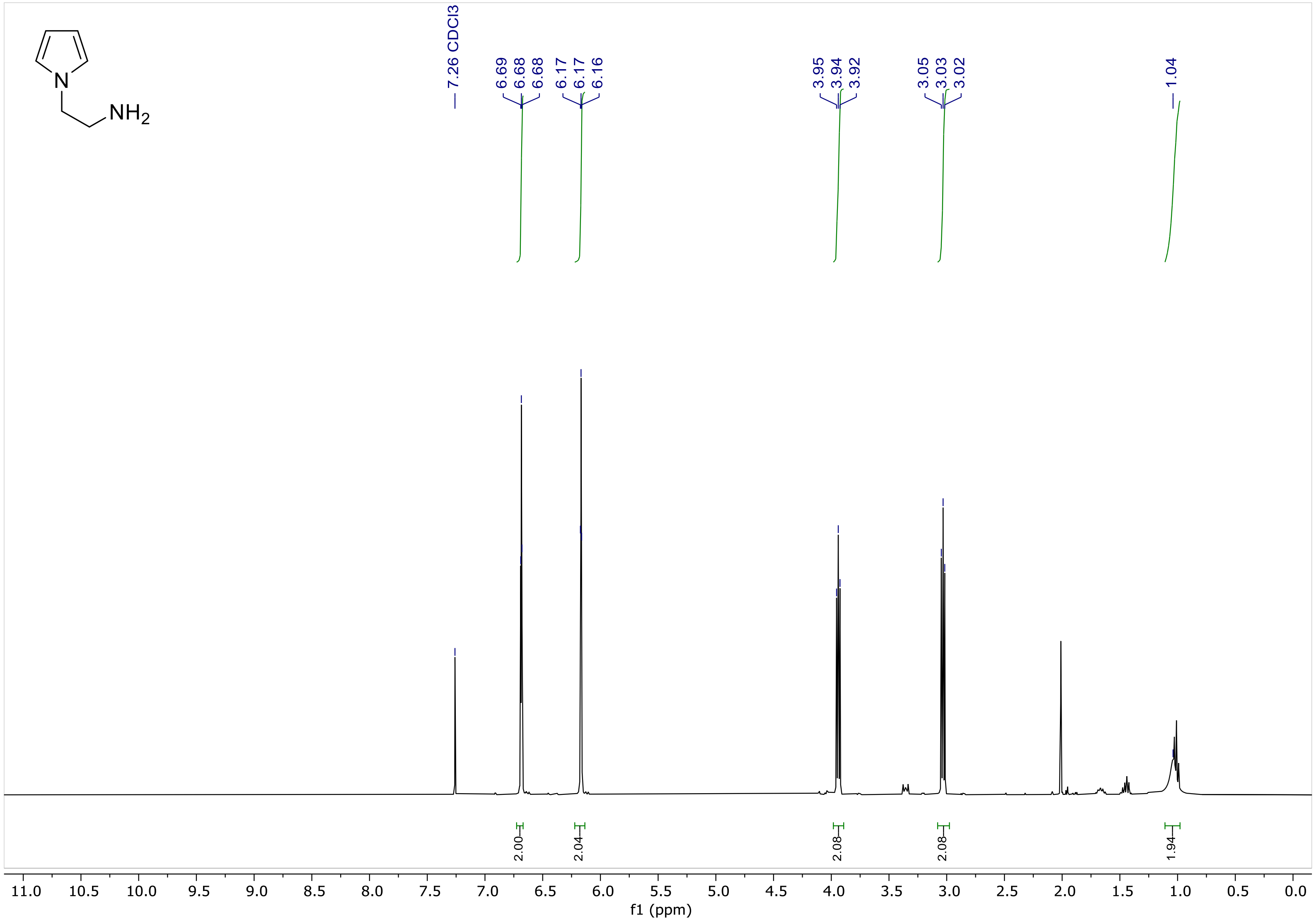

1-(4-Fluorophenyl)-1,2,3,4-tetrahydropyrrolo[1,2-*a*]pyrazine – ^{1}H NMR (400 MHz, $CDCl_3$)

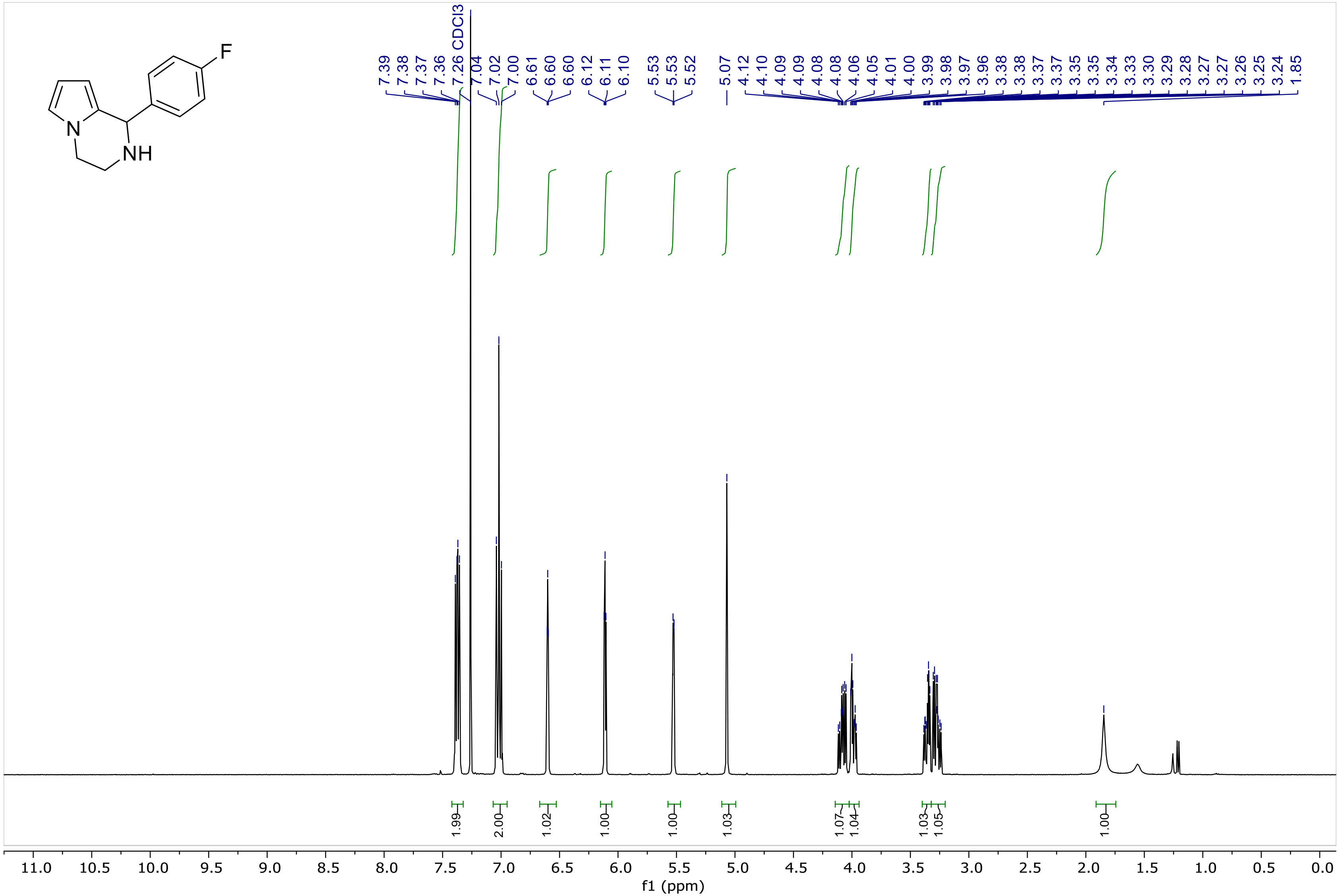

1-(4-Fluorophenyl)-1,2,3,4-tetrahydropyrrolo[1,2-*a*]pyrazine – $^{13}C\{^{1}H\}$ NMR (101 MHz, $CDCl_3$)

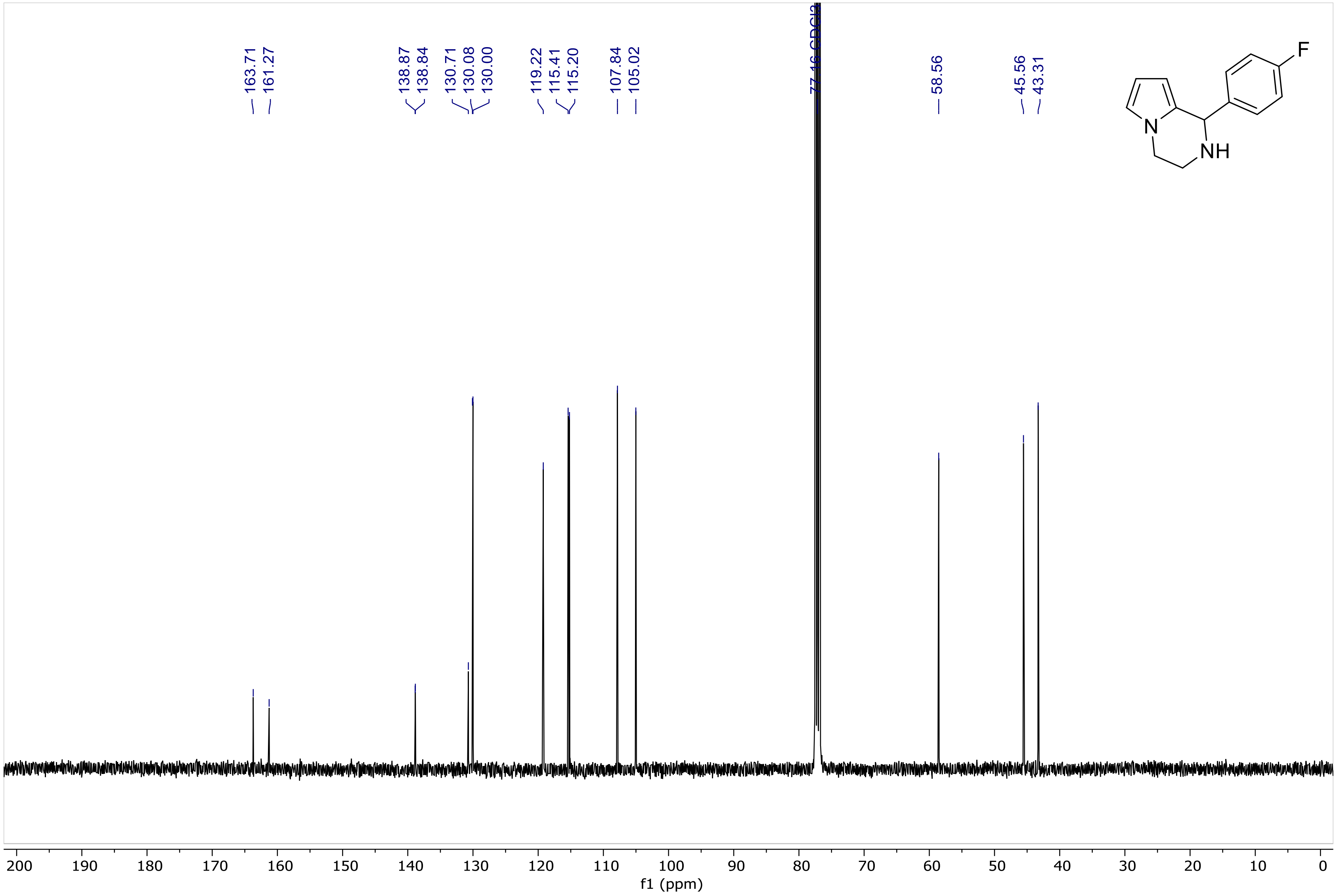

1-(4-Fluorophenyl)-1,2,3,4-tetrahydropyrrolo[1,2-*a*]pyrazine – ^{19}F NMR (376 MHz, $CDCl_3$)

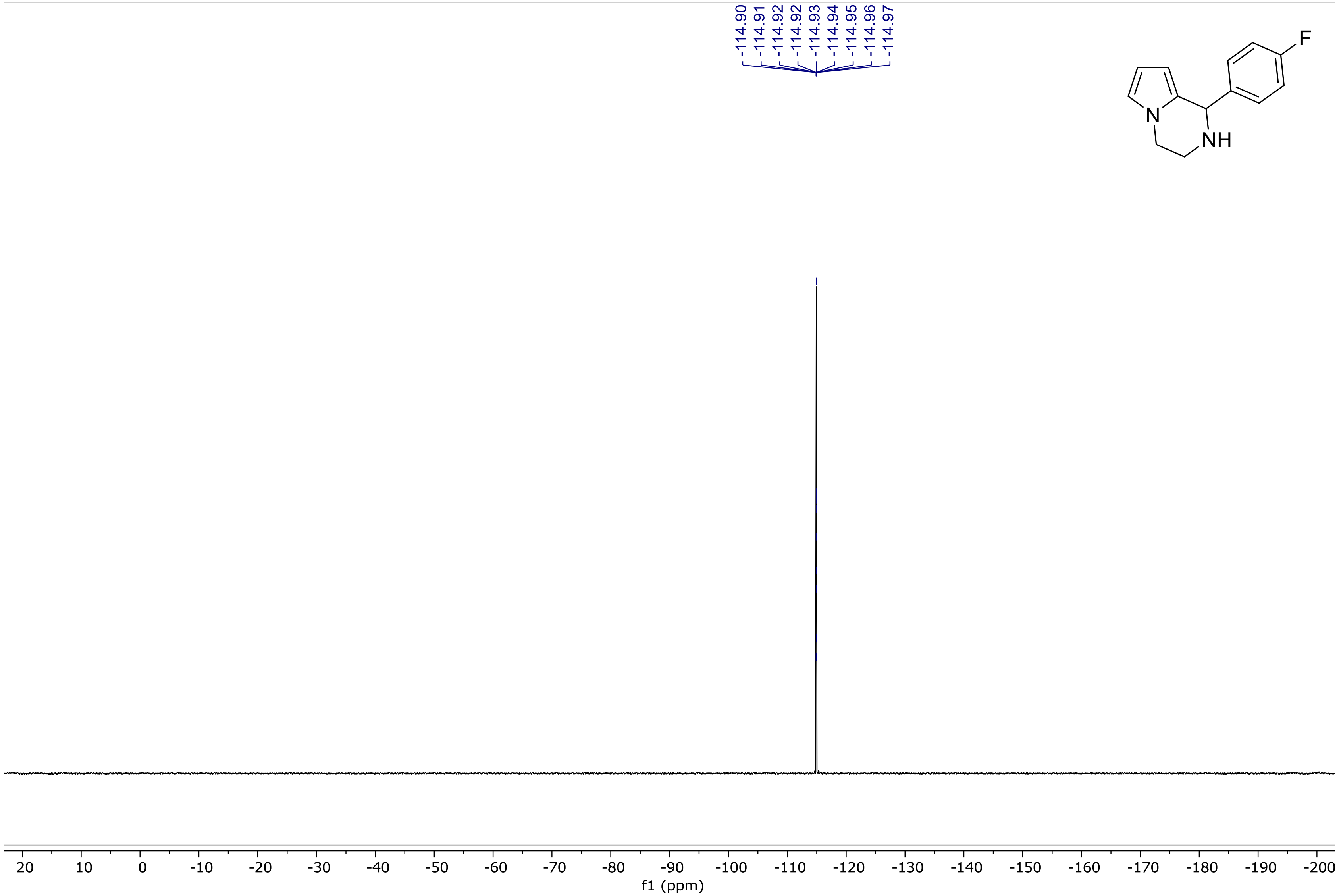

***tert*-Butyl 1-(4-fluorophenyl)-3,4-dihydropyrrolo[1,2-*a*]pyrazine-2(1*H*)-carboxylate – ^{1}H NMR (400 MHz, CDCl$_3$)**

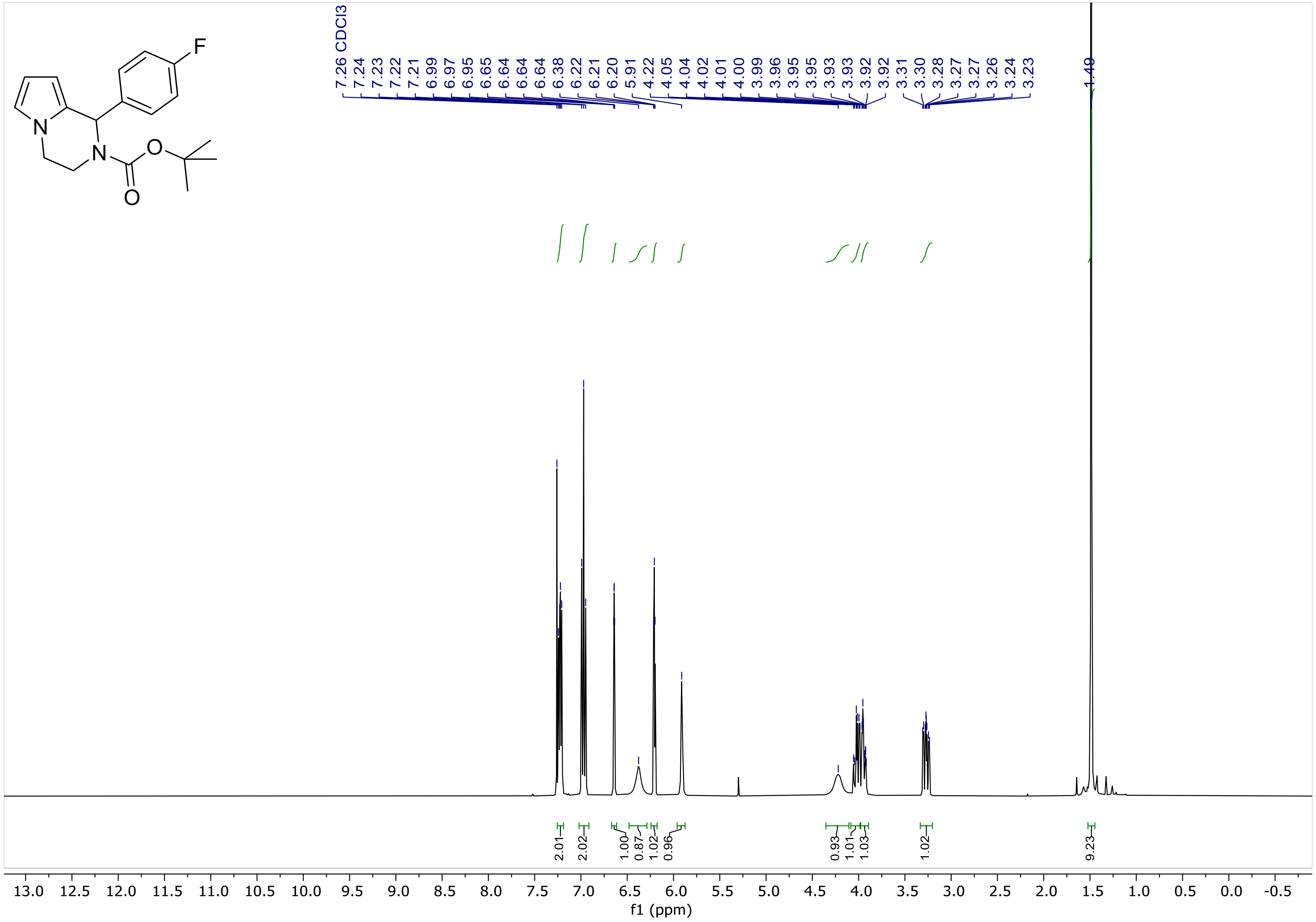

2-(tert-butoxycarbonyl)-1-(4-Fluorophenyl)-1,2,3,4-tetrahydropyrrolo[1,2-*a*]pyrazine – $^{13}C\{^{1}H\}$ NMR (101 MHz, $CDCl_3$)

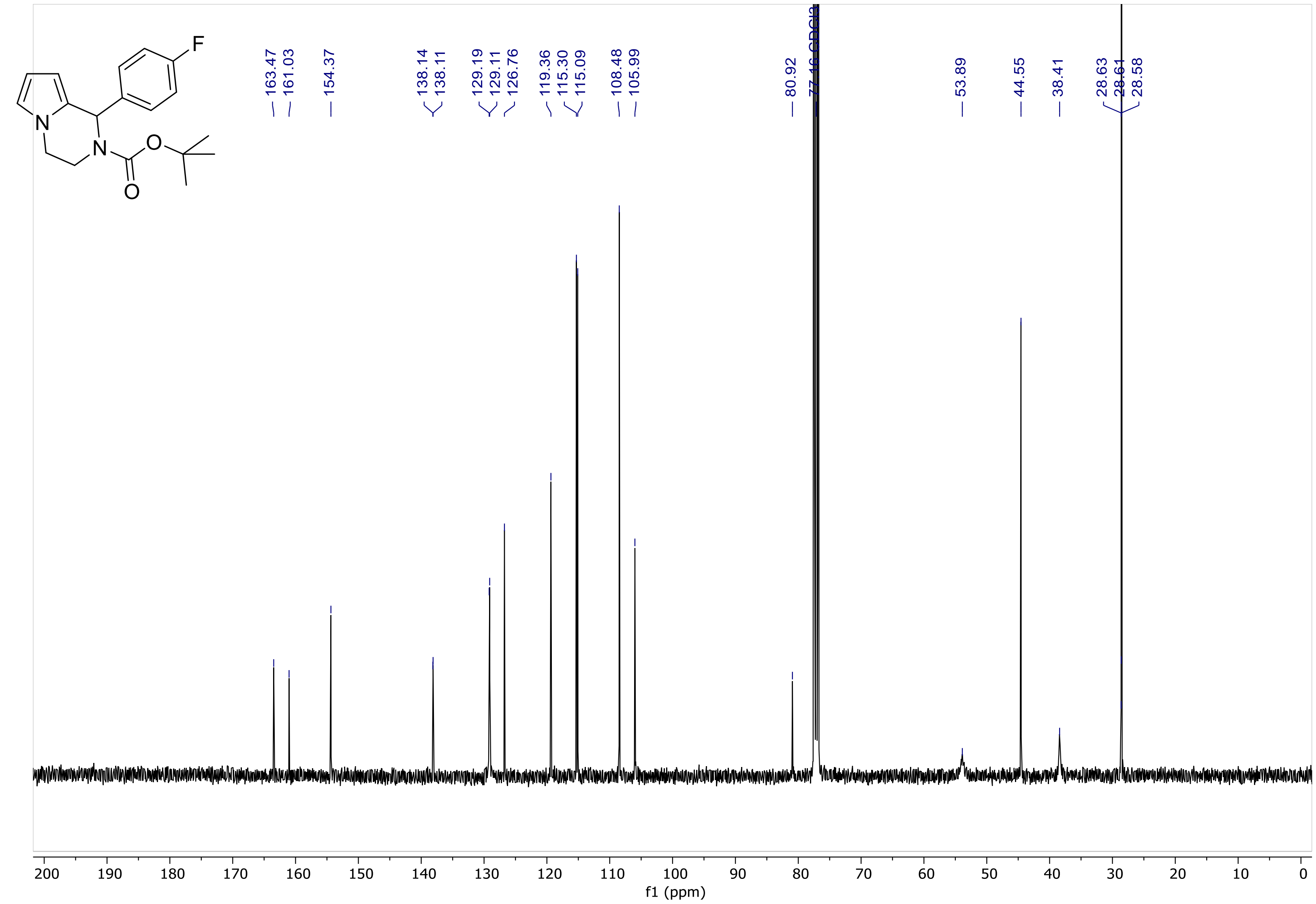

2-(tert-butoxycarbonyl)-1-(4-Fluorophenyl)-1,2,3,4-tetrahydropyrrolo[1,2-*a*]pyrazine – ^{19}F NMR (376 MHz, $CDCl_3$)

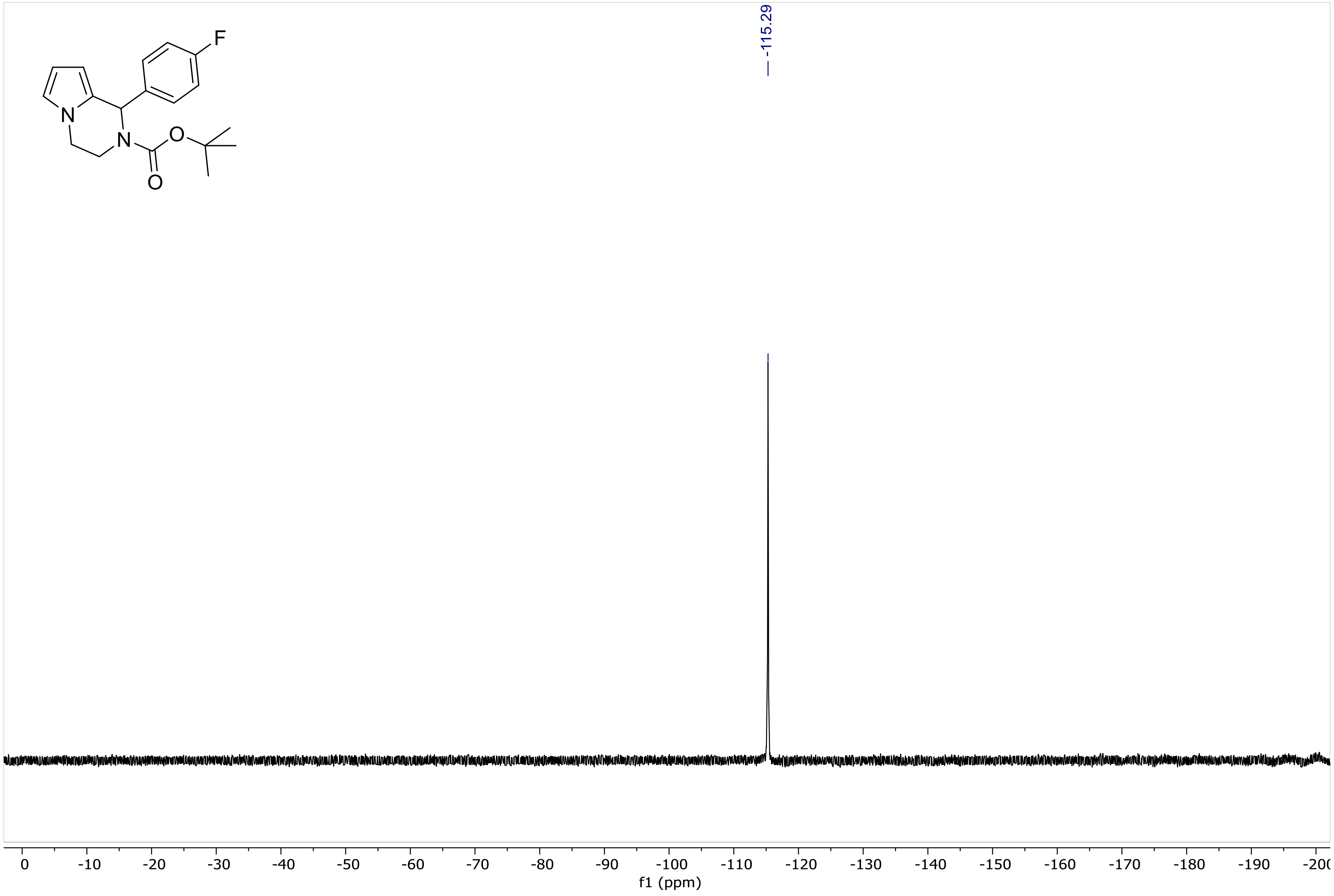

1-Benzyl-3,5-dimethylpyrazole - ^{1}H NMR ($CDCl_3$)

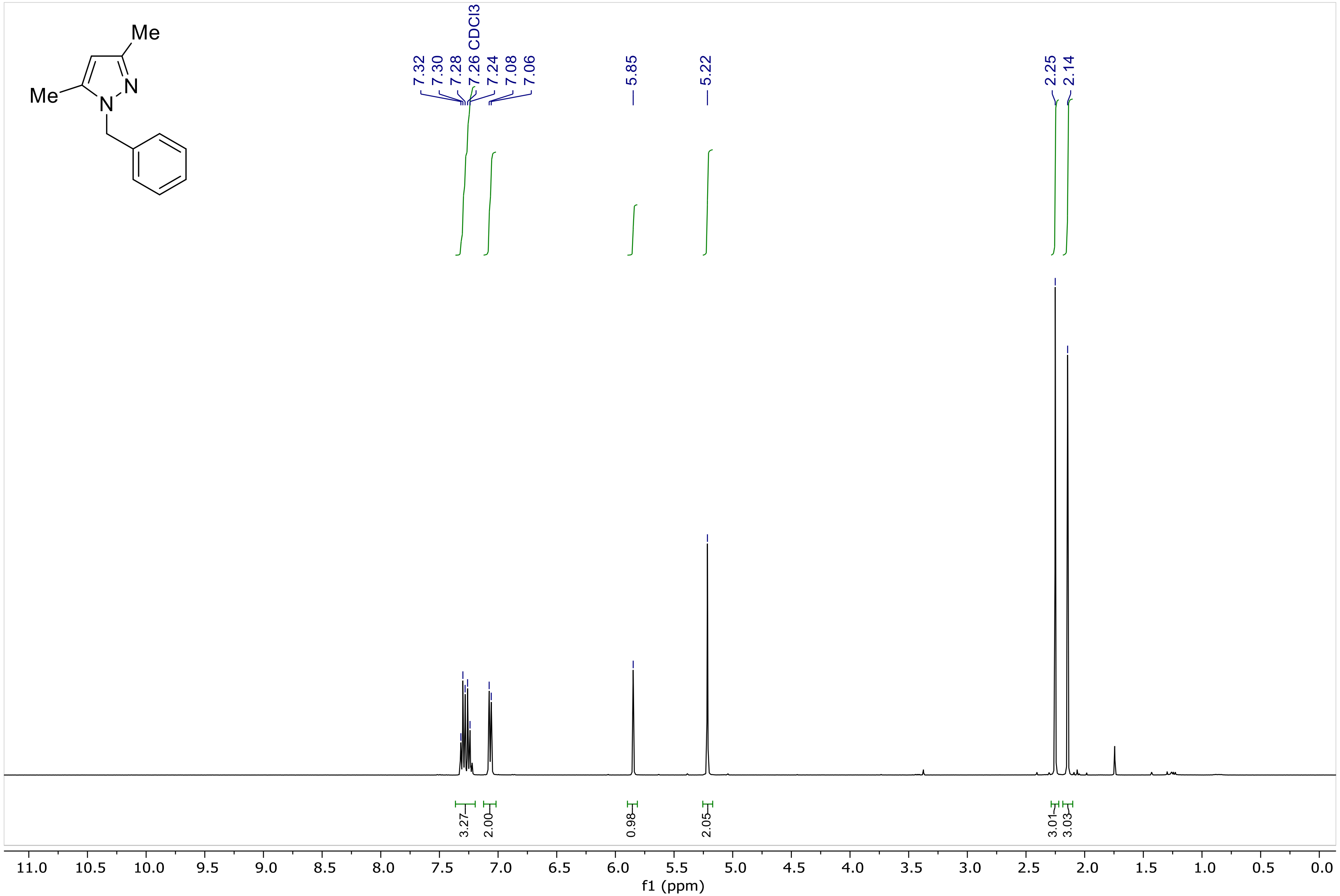

1-Benzyl-3,5-dimethylpyrazole – $^{13}C\{^1H\}$ NMR ($CDCl_3$)

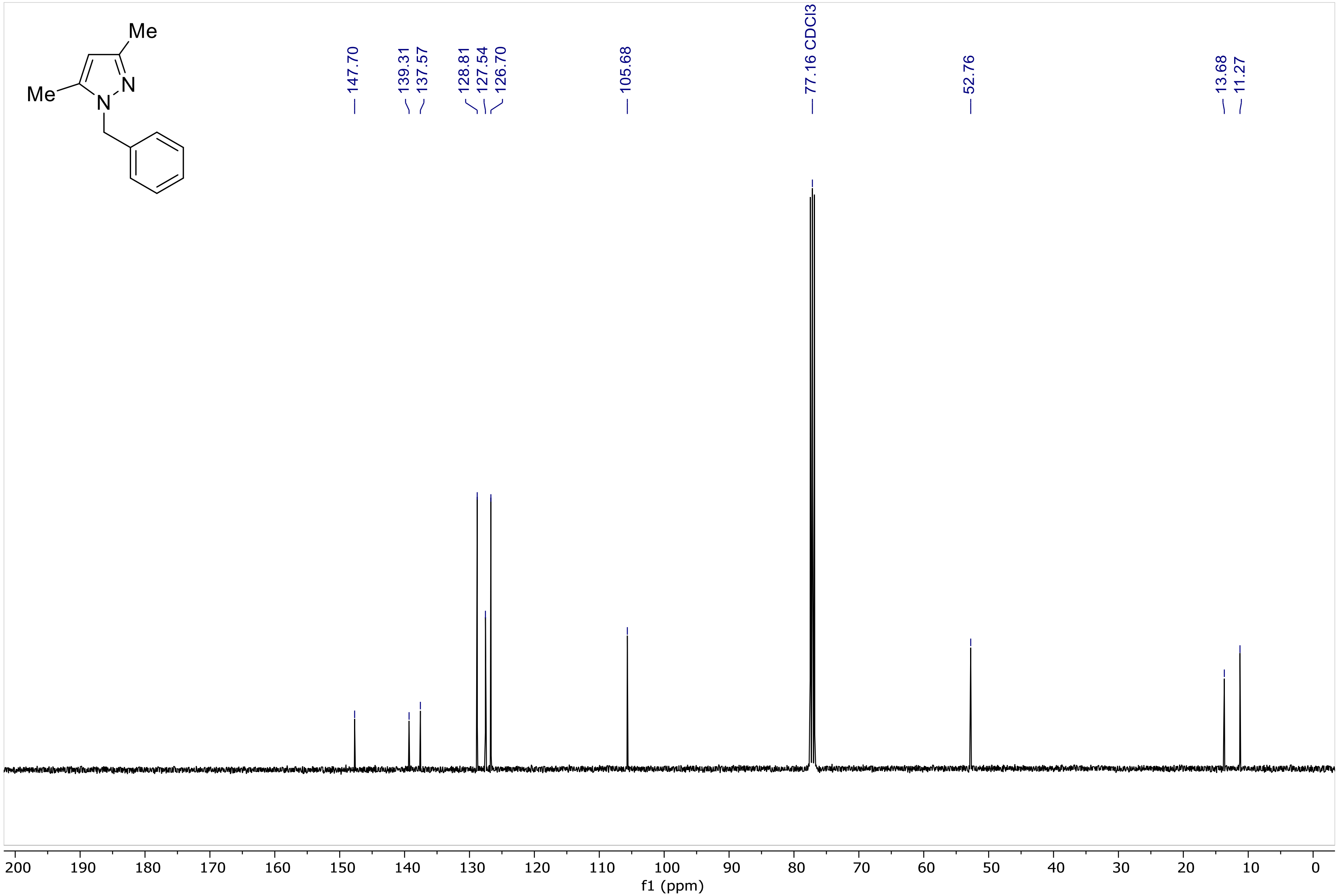

1-Benzylpyrazole - ^{1}H NMR ($CDCl_3$)

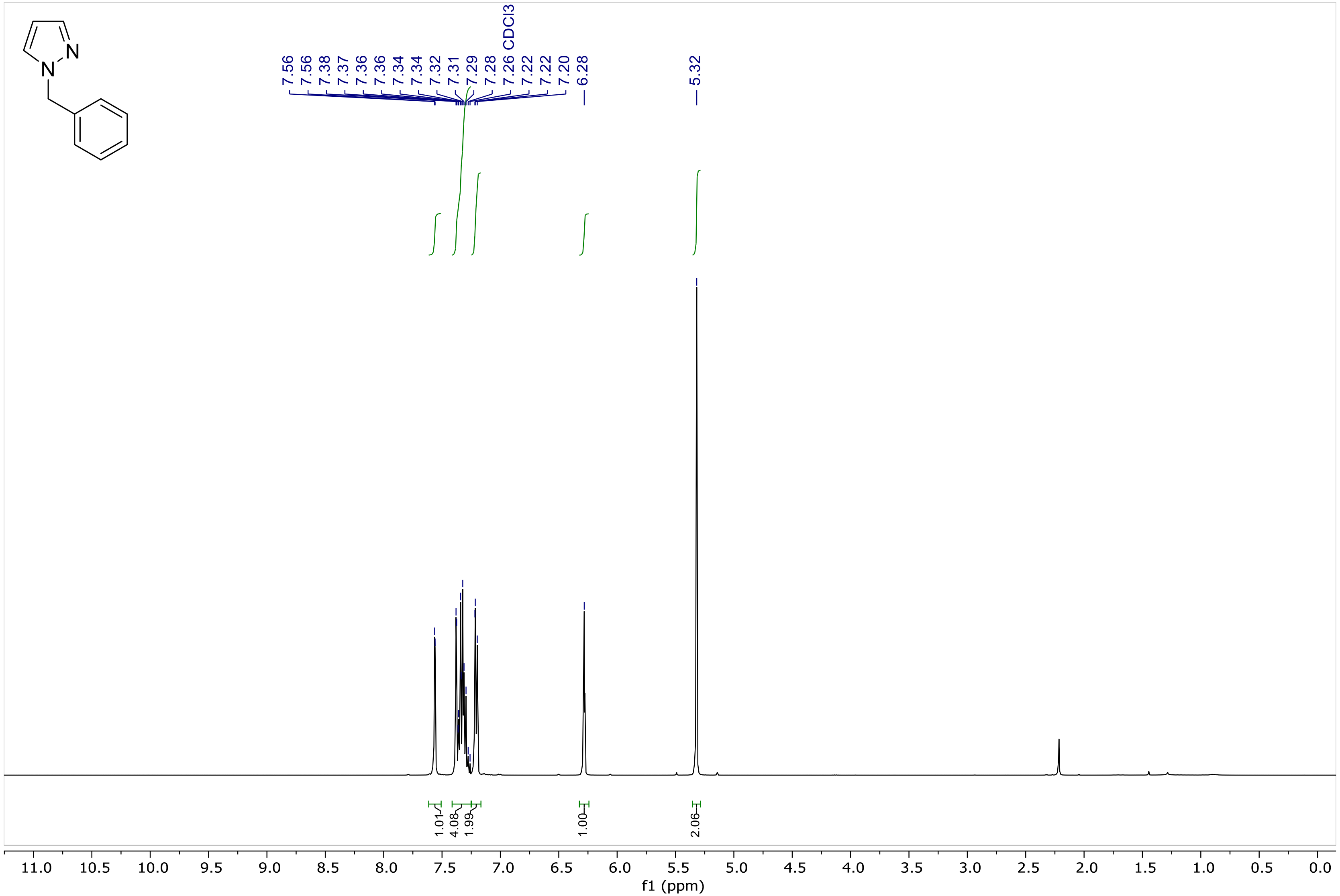

1-Benzylpyrazole – $^{13}C\{^1H\}$ NMR ($CDCl_3$)

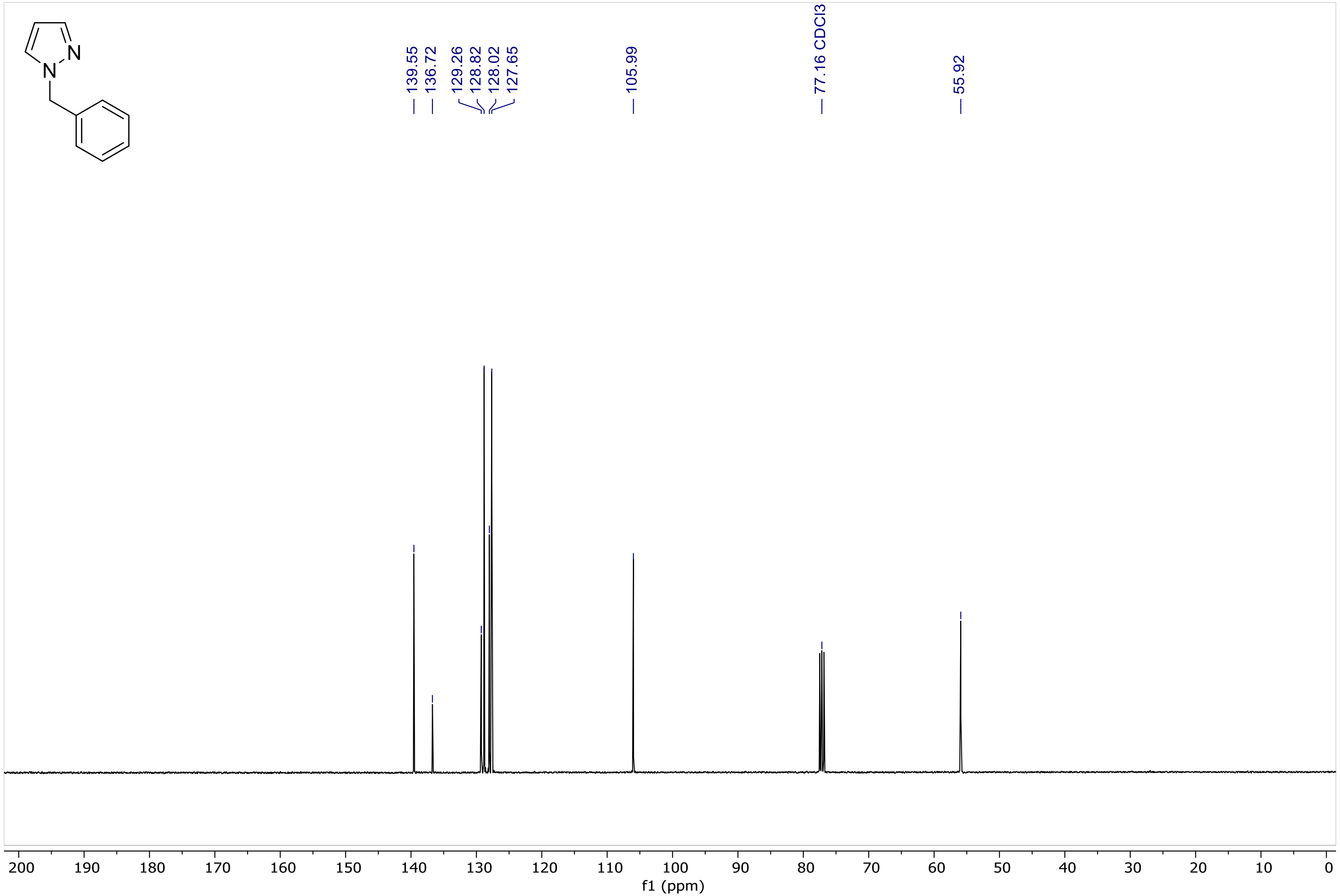

1-Benzyl-3,5-diethylpyrazole - ^{1}H NMR ($CDCl_3$)

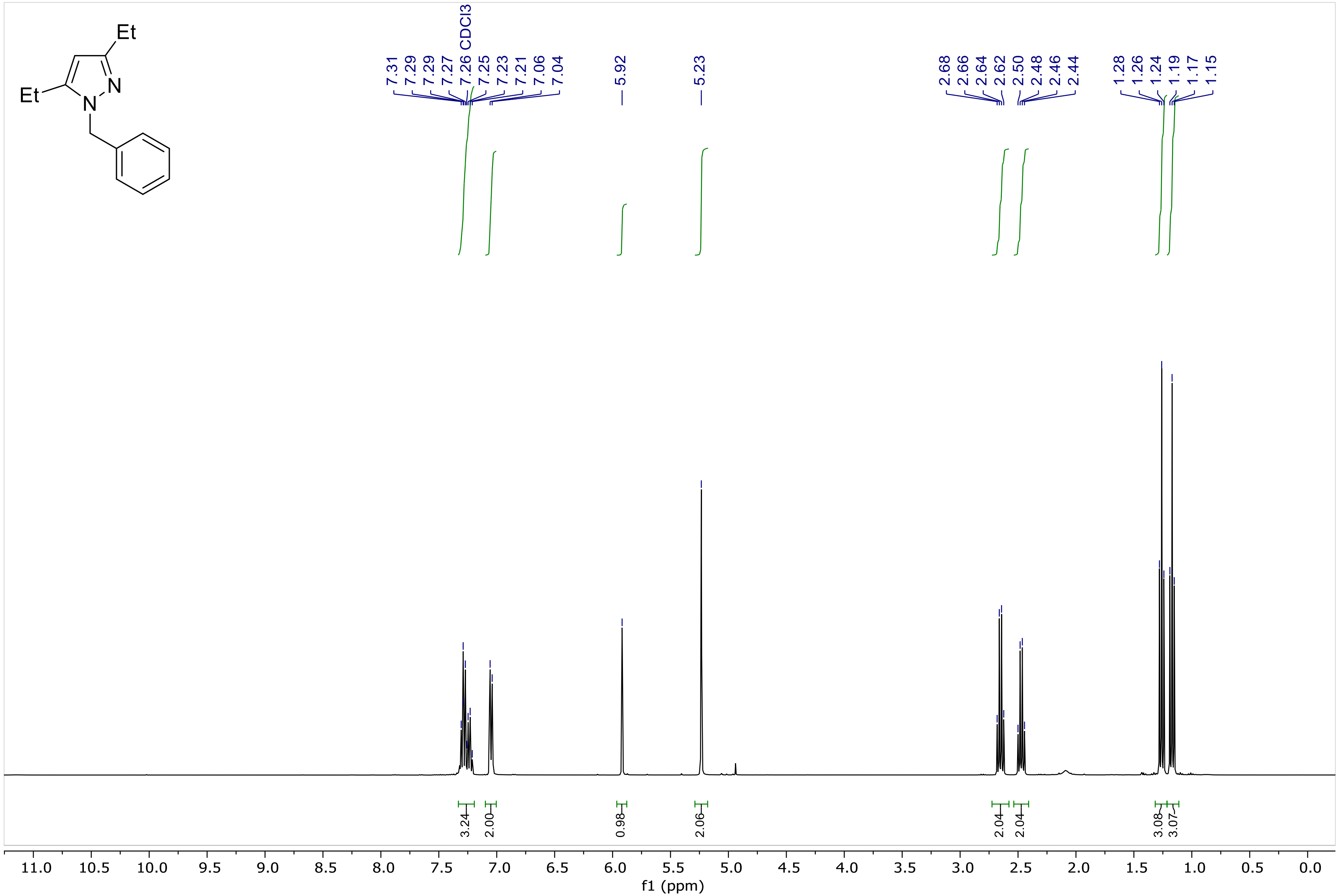

1-Benzyl-3,5-diethylpyrazole – $^{13}C\{^{1}H\}$ NMR ($CDCl_3$)

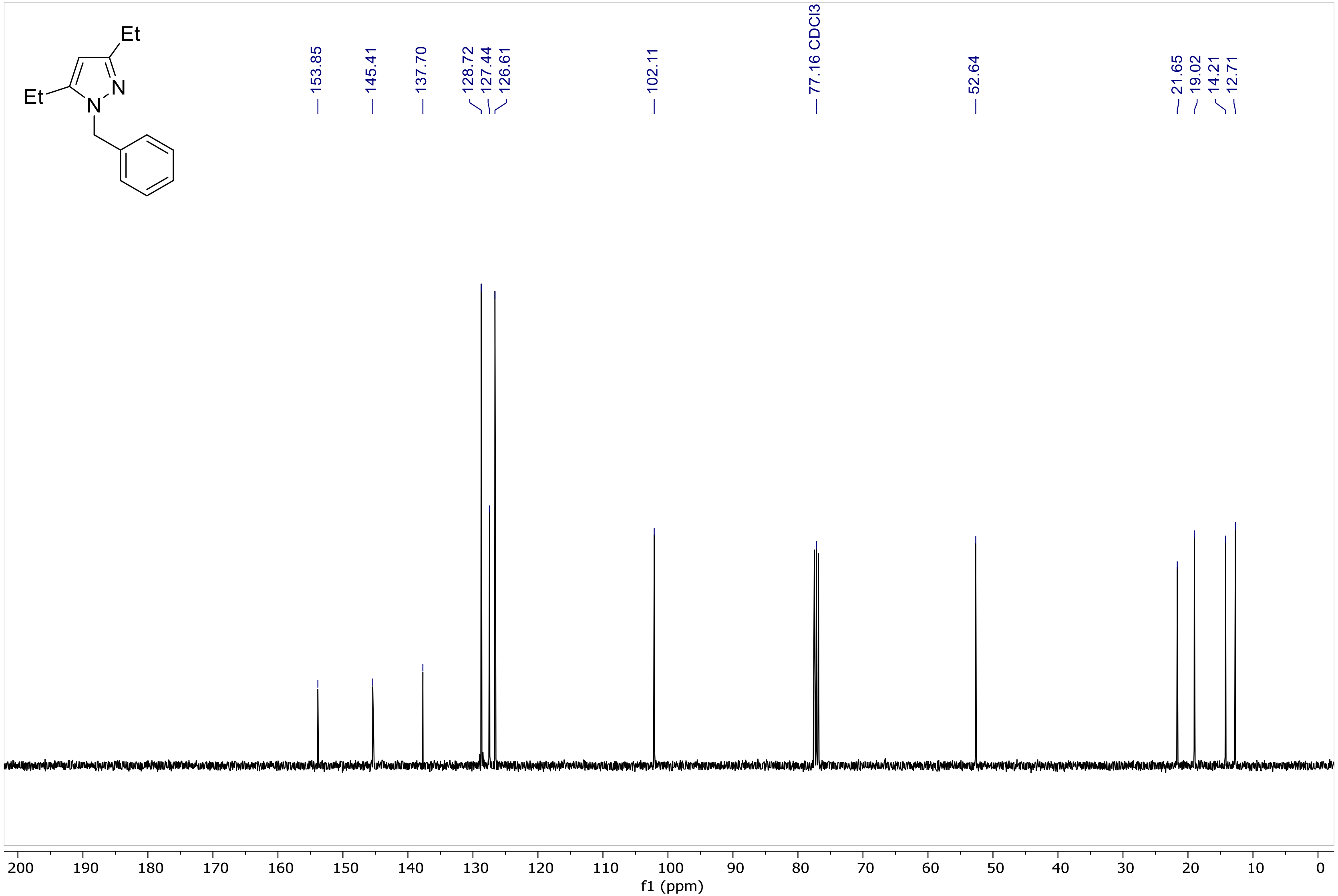

1-Benzyl-3,4,5-triethylpyrazole - ^{1}H NMR ($CDCl_3$)

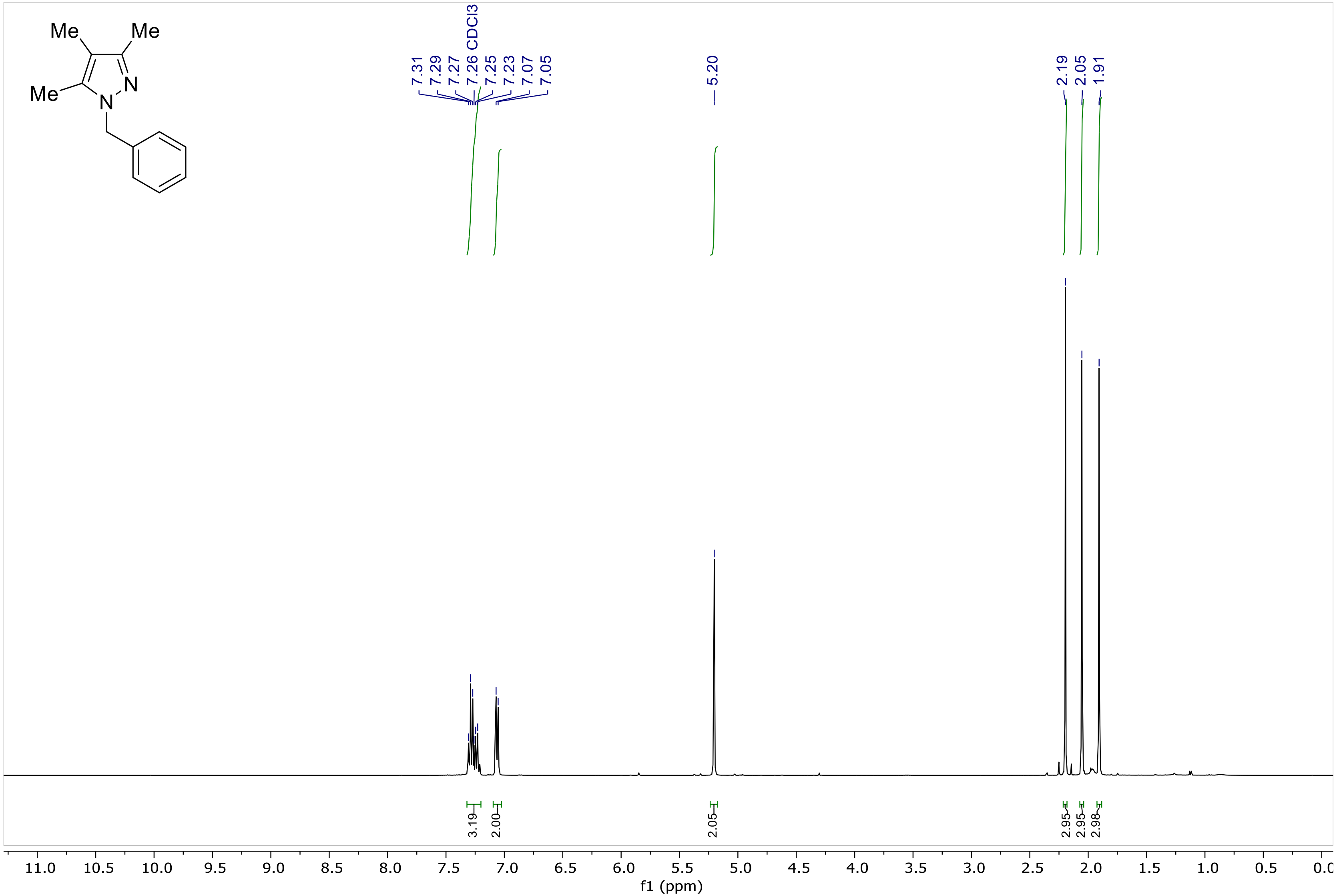

1-Benzyl-3,4,5-triethylpyrazole – $^{13}C\{^{1}H\}$ NMR ($CDCl_3$)

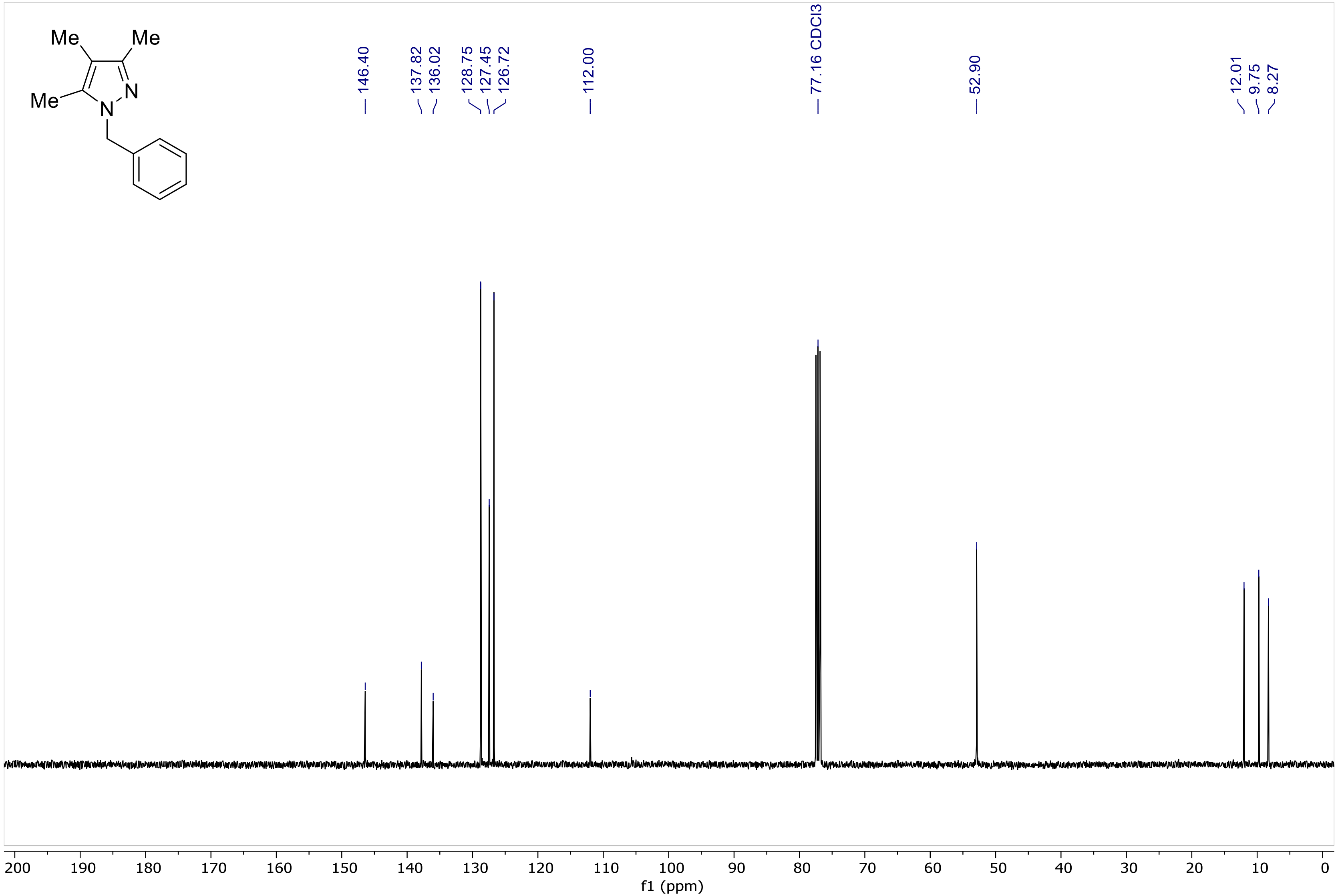

1-Benzyl-1*H*-indazole- ^{1}H NMR ($CDCl_3$)

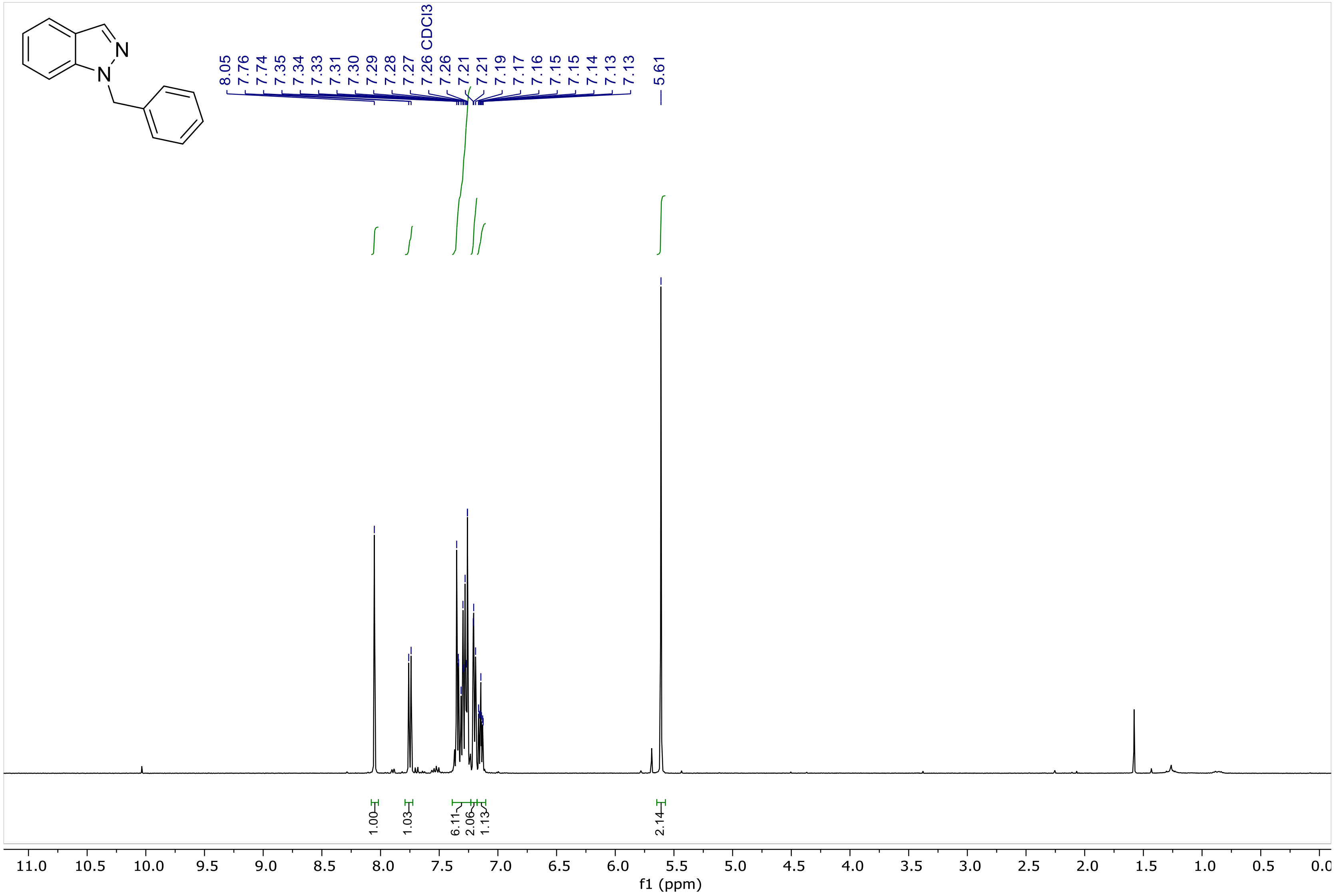

1-Benzyl-1*H*-indazole- $^{13}C\{^{1}H\}$ NMR ($CDCl_3$)

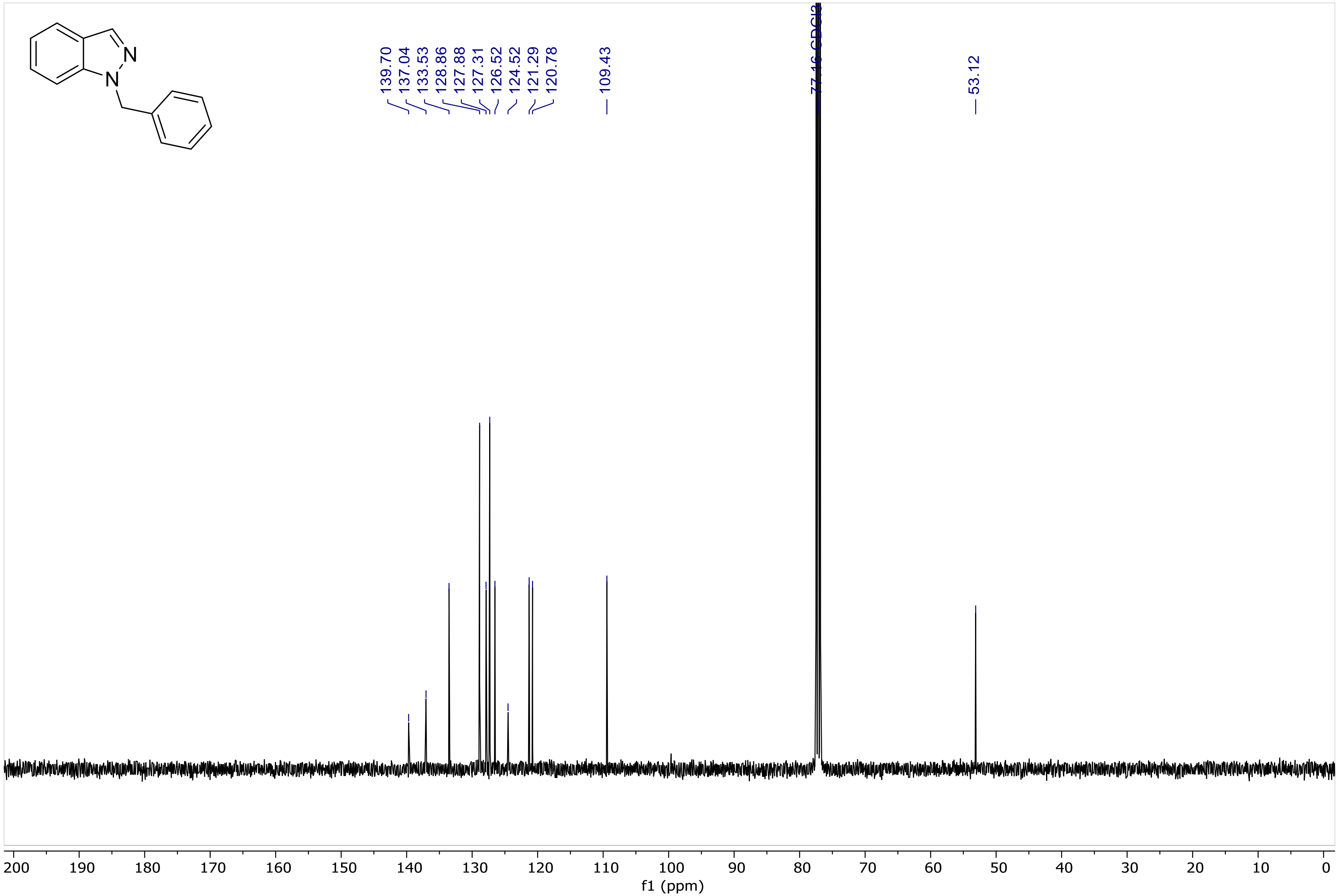

2-Benzyl-2*H*-indazole- ^{1}H NMR ($CDCl_3$)

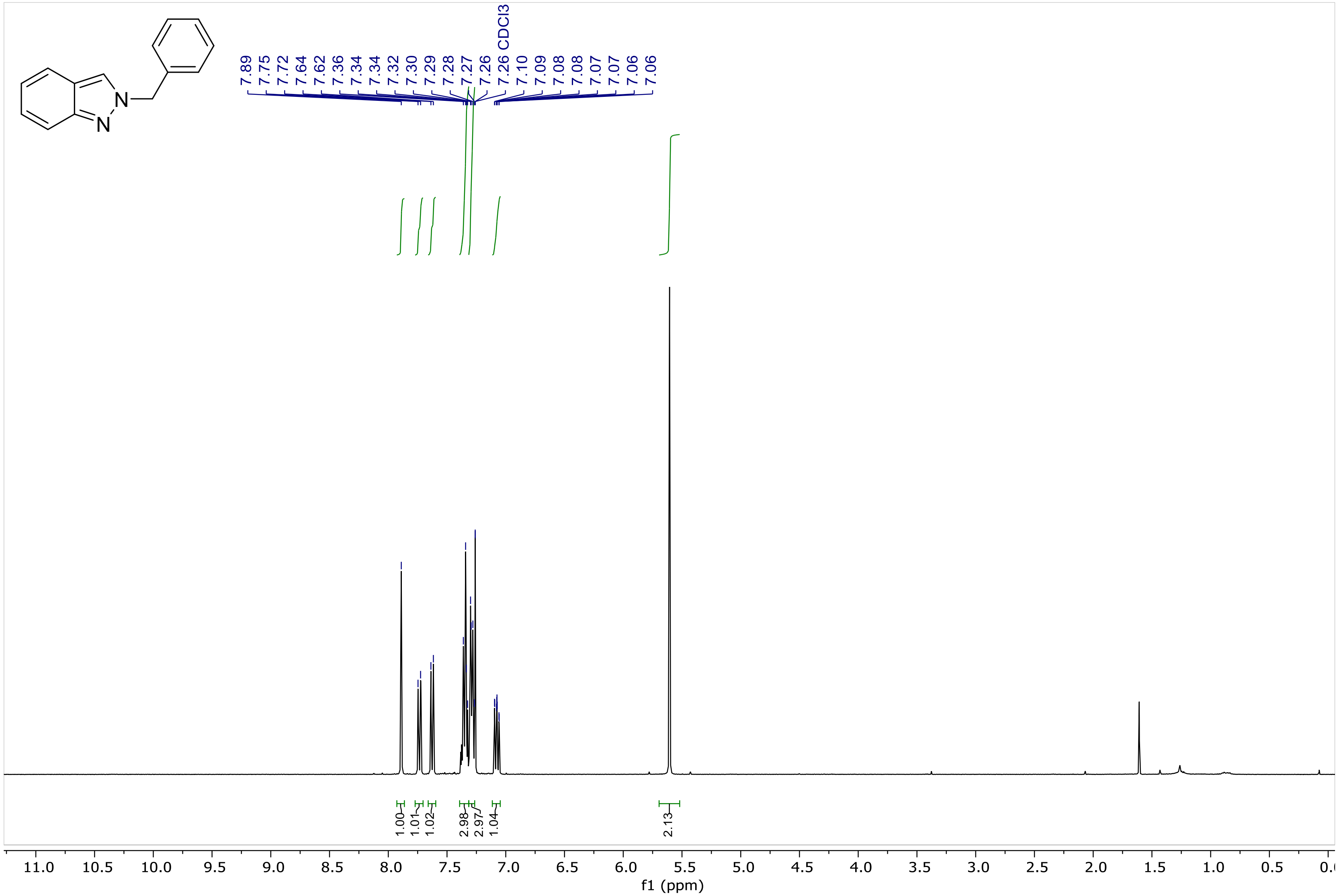

2-Benzyl-2*H*-indazole- $^{13}C\{^{1}H\}$ NMR ($CDCl_3$)

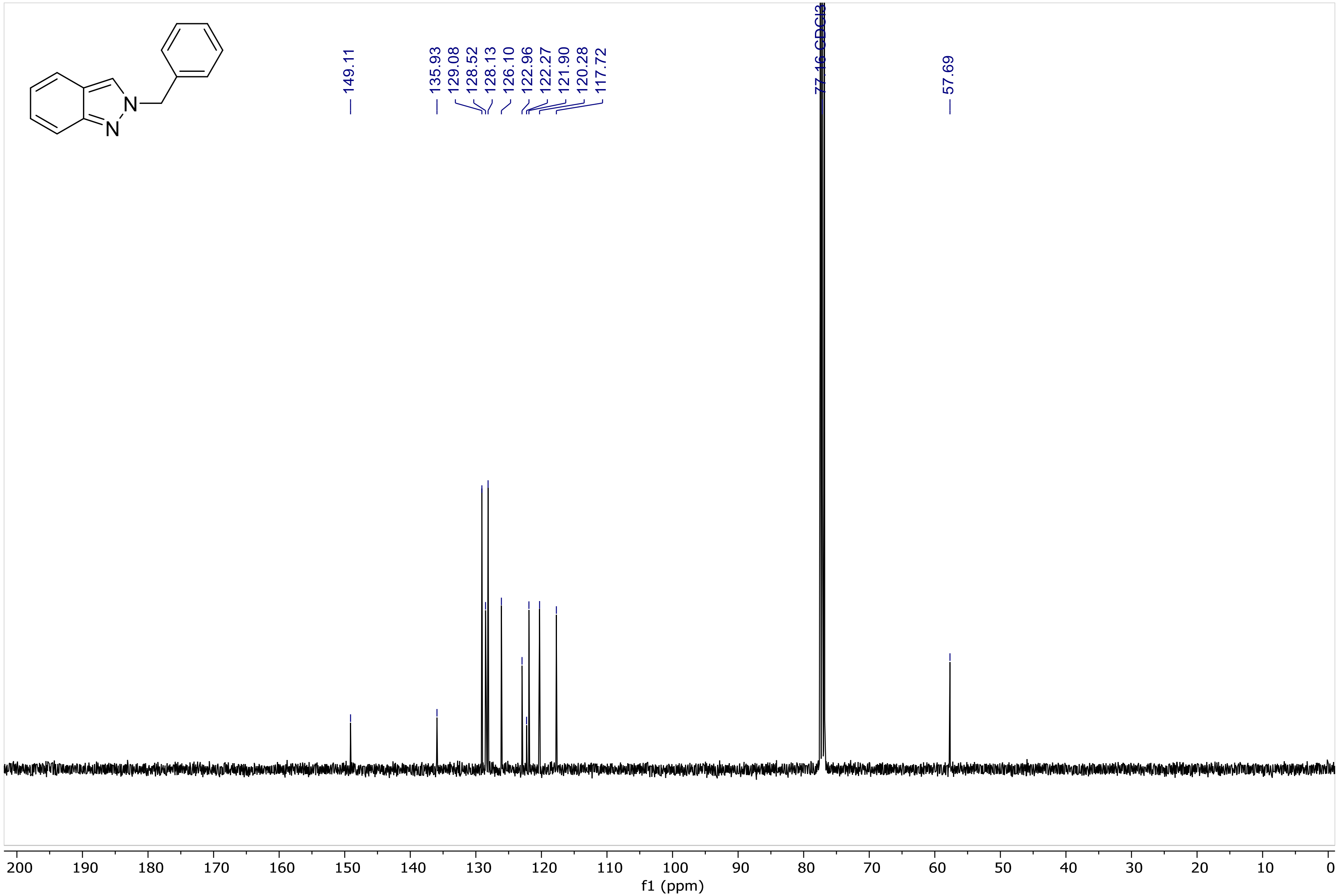

1: 3-Phenyl-3-chloro-3*H*-diazirine – ^{1}H NMR (400 MHz, $CDCl_3$)

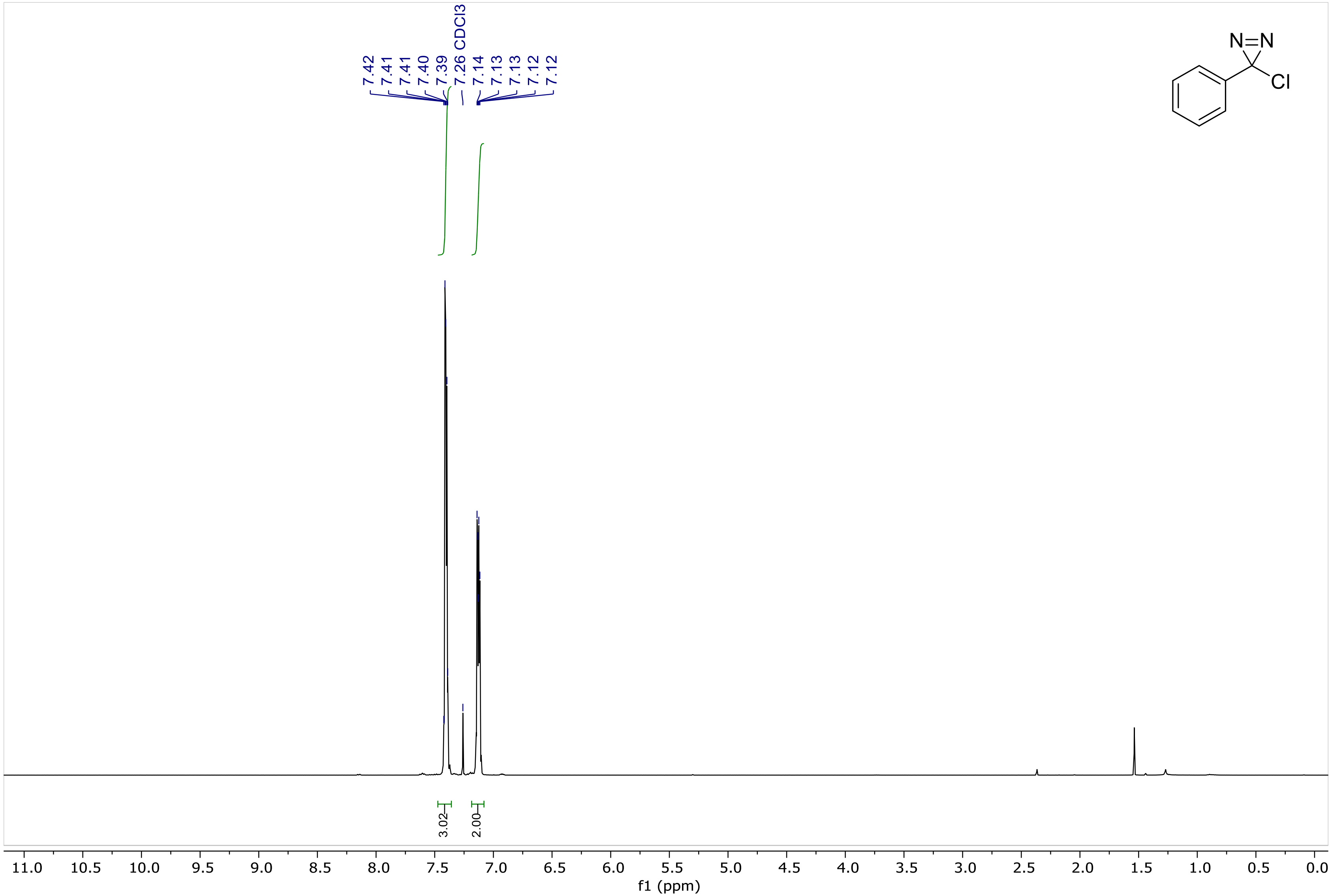

3-Phenyl-3-chloro-3*H*-diazirine – $^{13}C\{^{1}H\}$ NMR (101 MHz, $CDCl_3$)

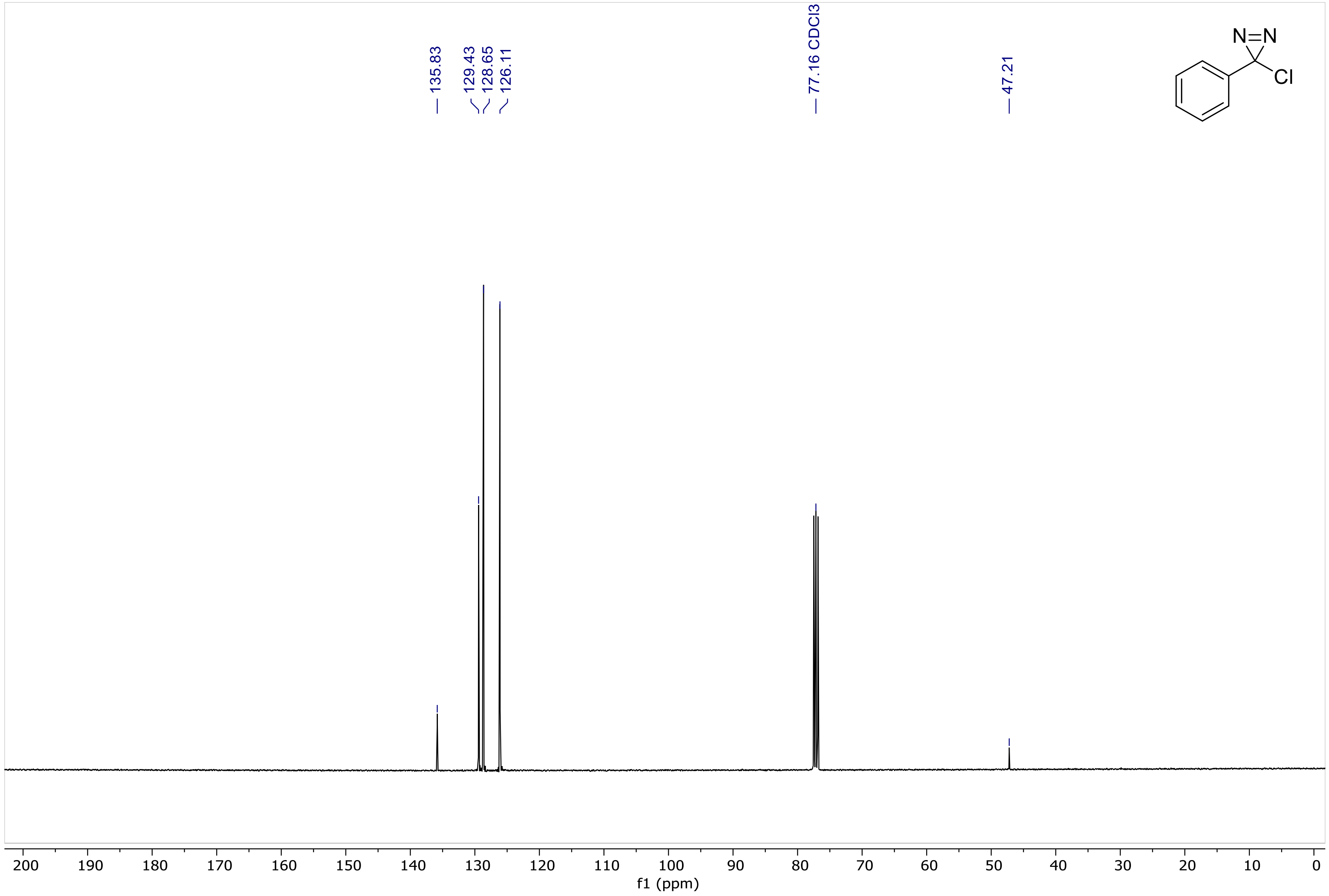

3-(4-Methylphenyl)-3-chloro-3*H*-diazirine – ^{1}H NMR (400 MHz, $CDCl_3$)

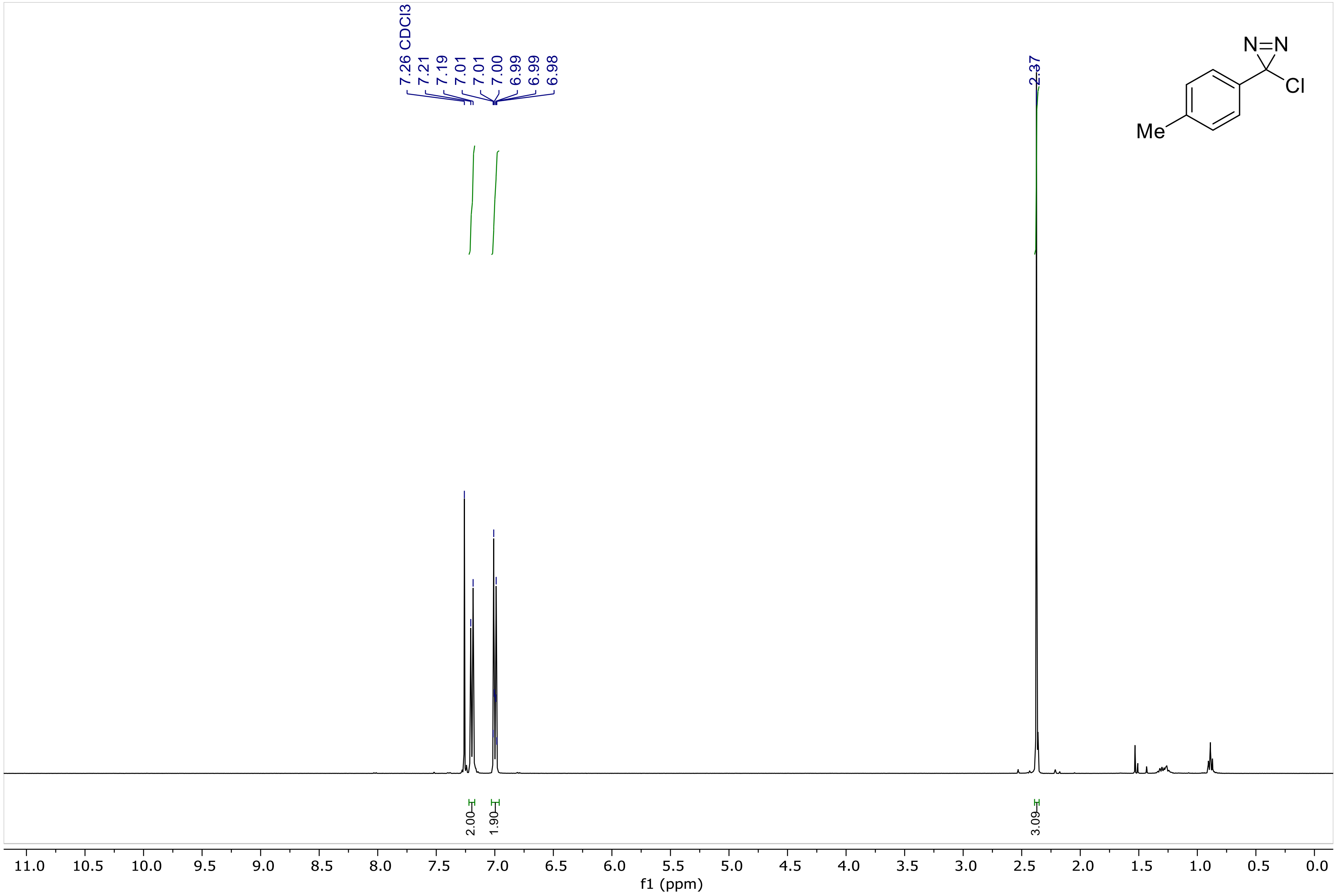

3-(4-Methylphenyl)-3-chloro-3*H*-diazirine – $^{13}C\{^{1}H\}$ NMR (101 MHz, $CDCl_3$)

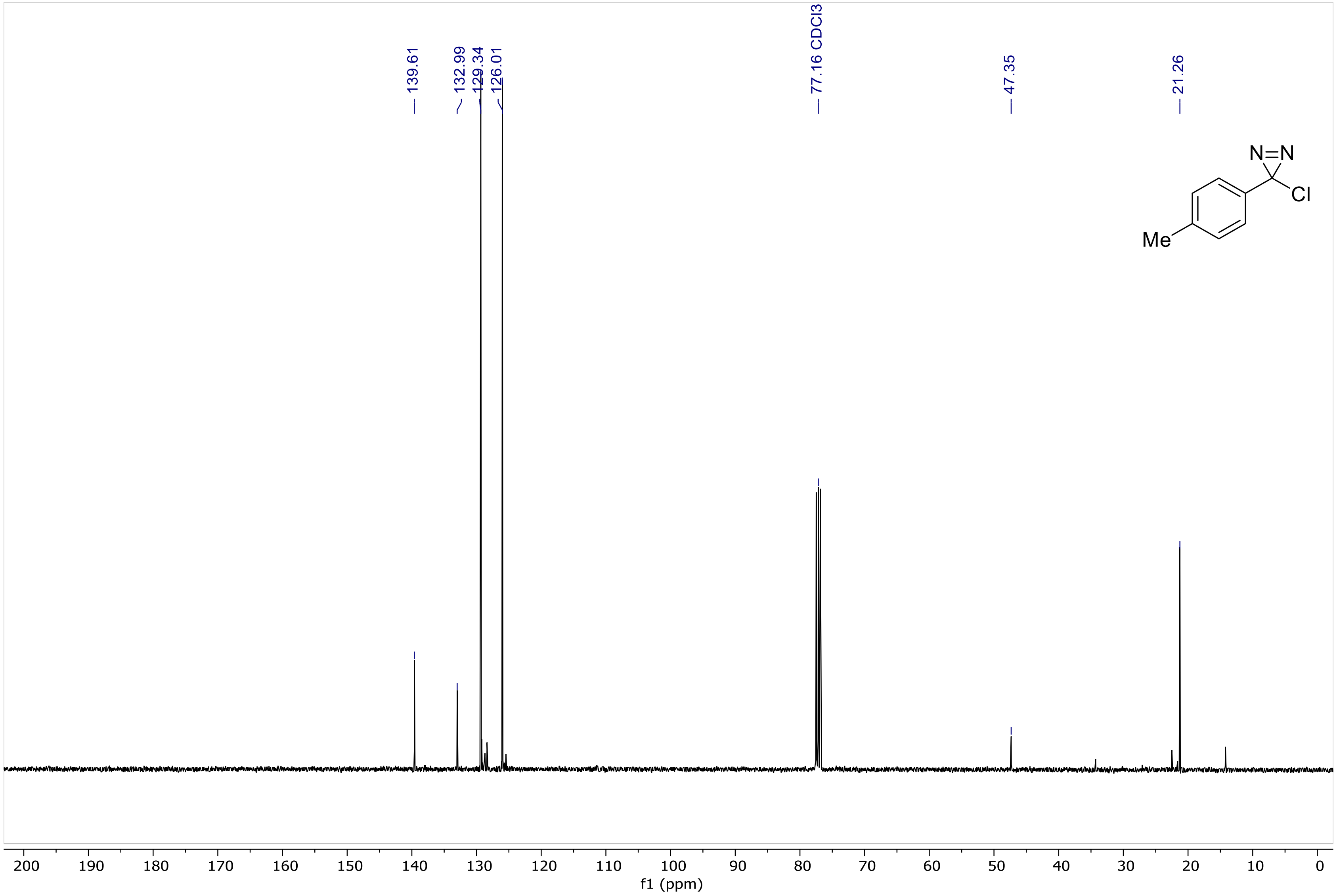

3-(4-Fluorophenyl)-3-chloro-3*H*-diazirine – ^{1}H NMR (400 MHz, $CDCl_3$)

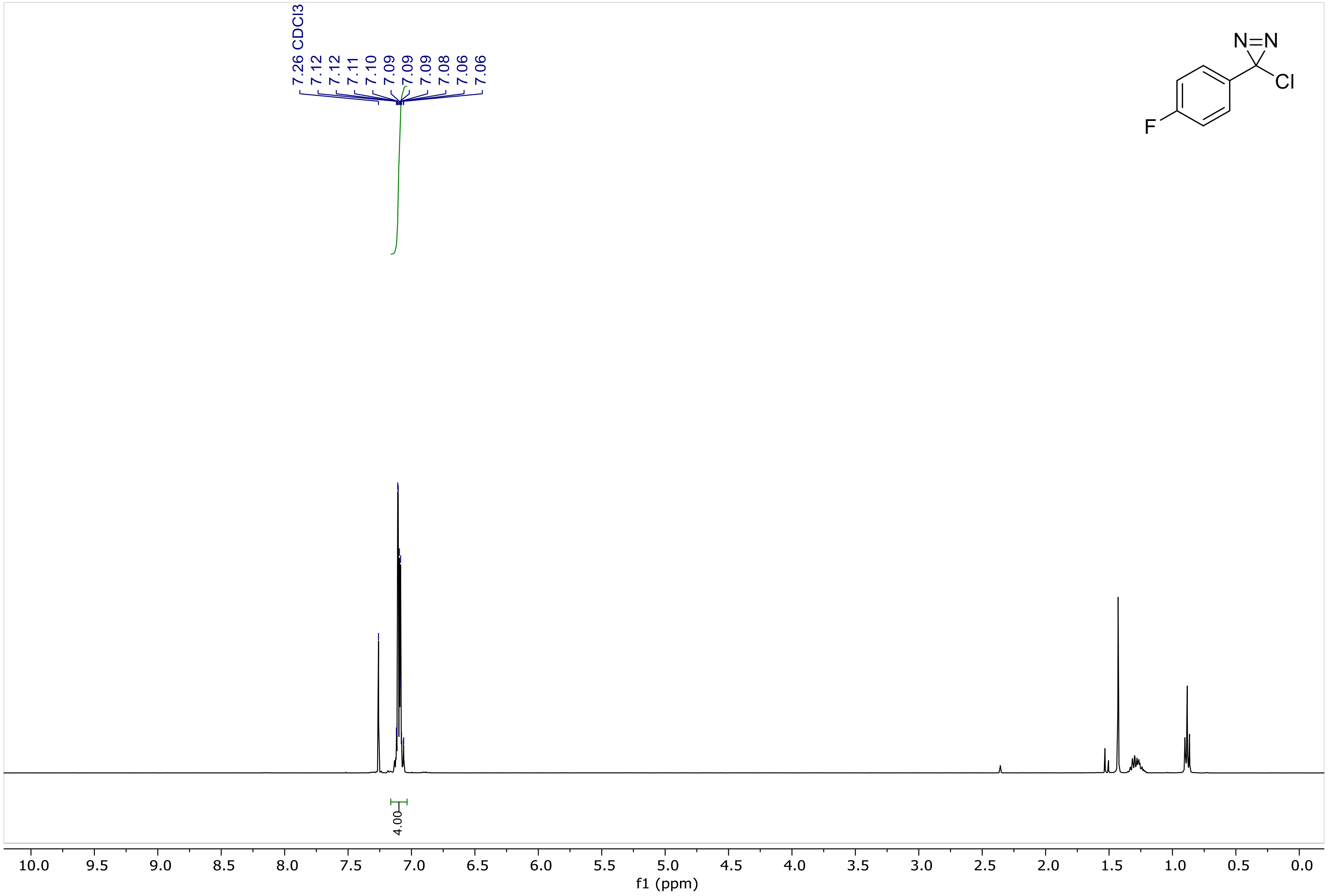

3-(4-Fluorophenyl)-3-chloro-3*H*-diazirine – $^{13}C\{^{1}H\}$ NMR (101 MHz, $CDCl_3$)

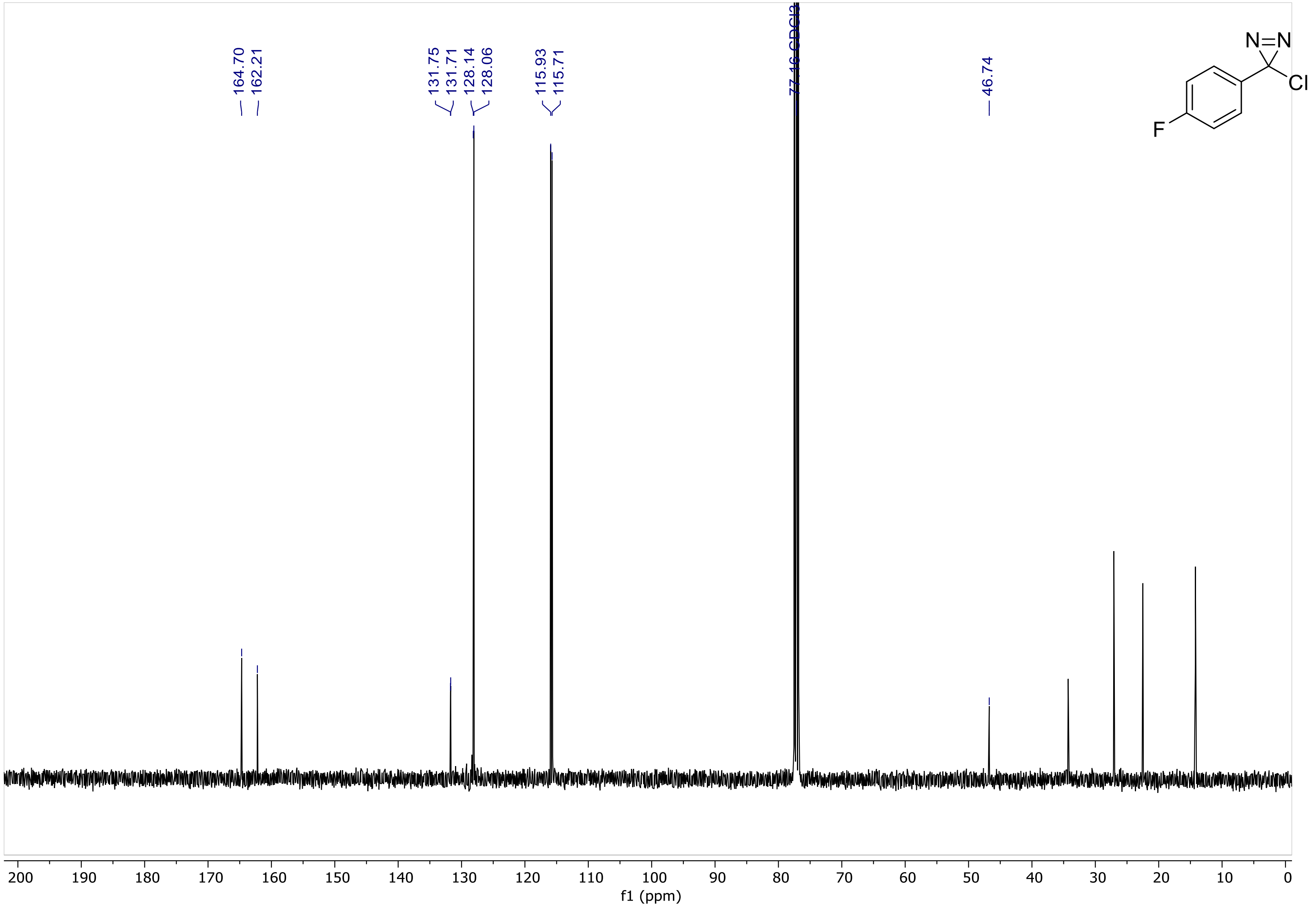

3-(4-Fluorophenyl)-3-chloro-3*H*-diazirine – ^{19}F NMR (376 MHz, $CDCl_3$)

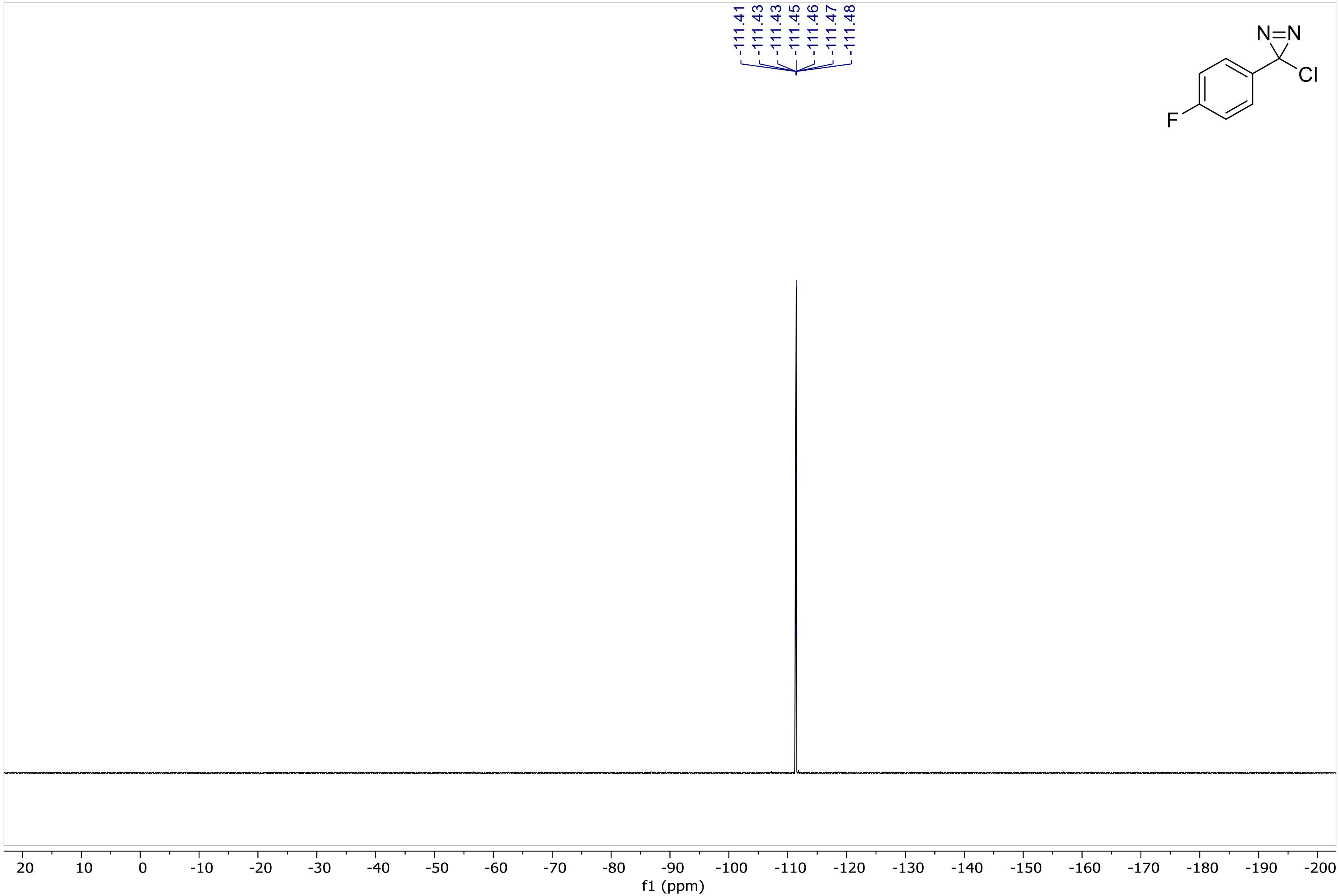

3-(4-Chlorophenyl)-3-chloro-3*H*-diazirine – ^{1}H NMR (400 MHz, $CDCl_3$)

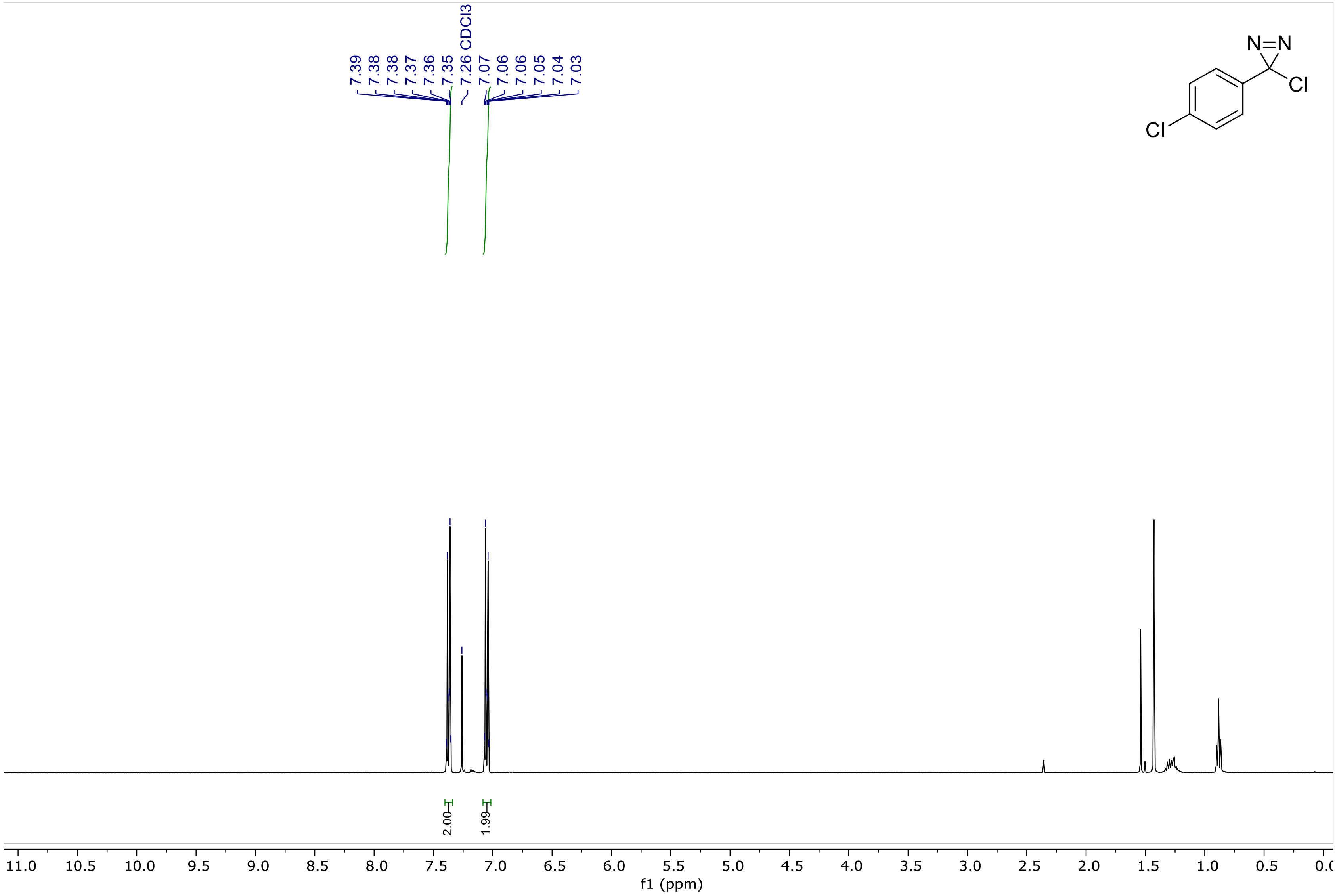

3-(4-Chlorophenyl)-3-chloro-3*H*-diazirine – $^{13}C\{^{1}H\}$ NMR (101 MHz, $CDCl_3$)

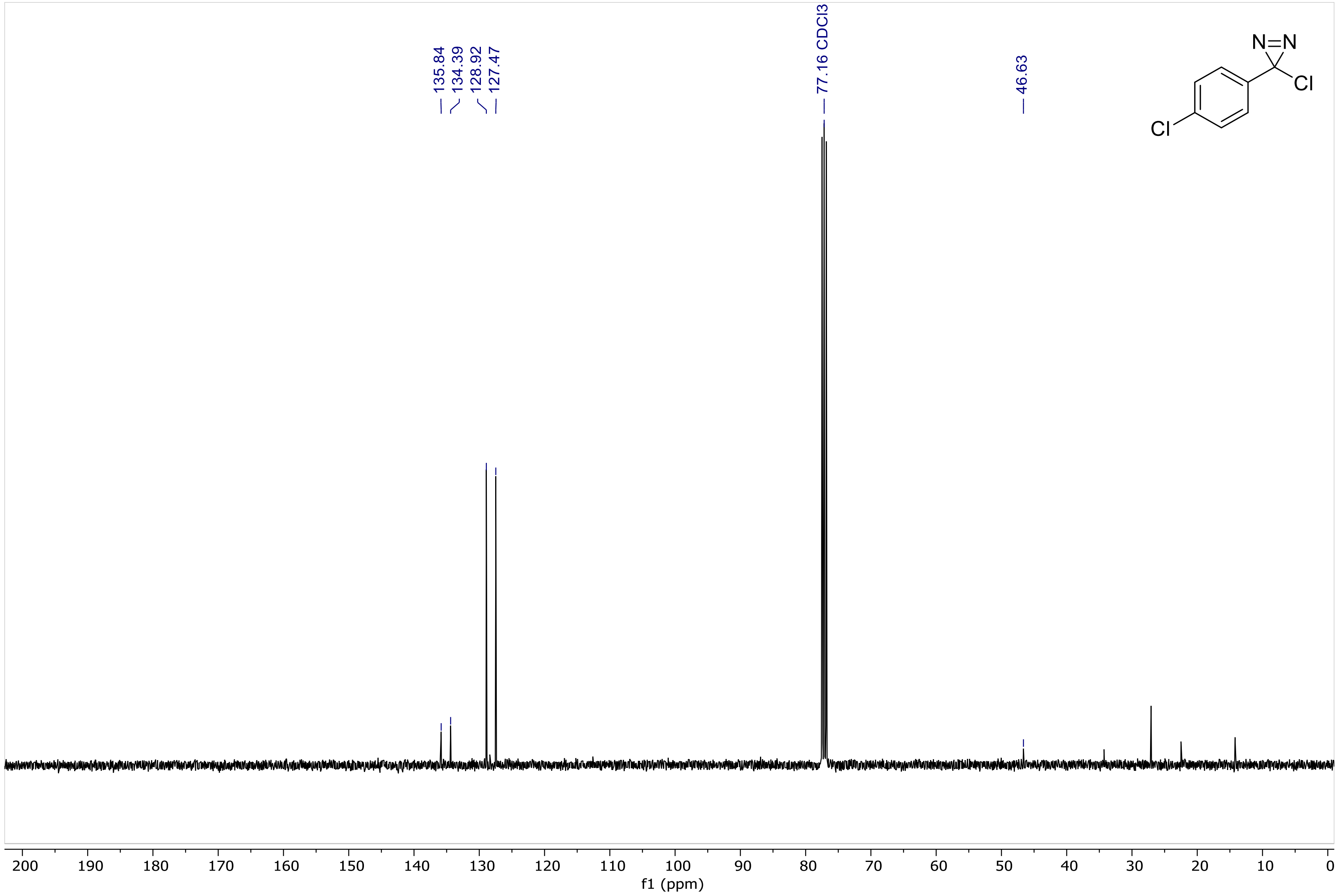

3-(4-Bromophenyl)-3-chloro-3*H*-diazirine – ^{1}H NMR (400 MHz, $CDCl_3$)

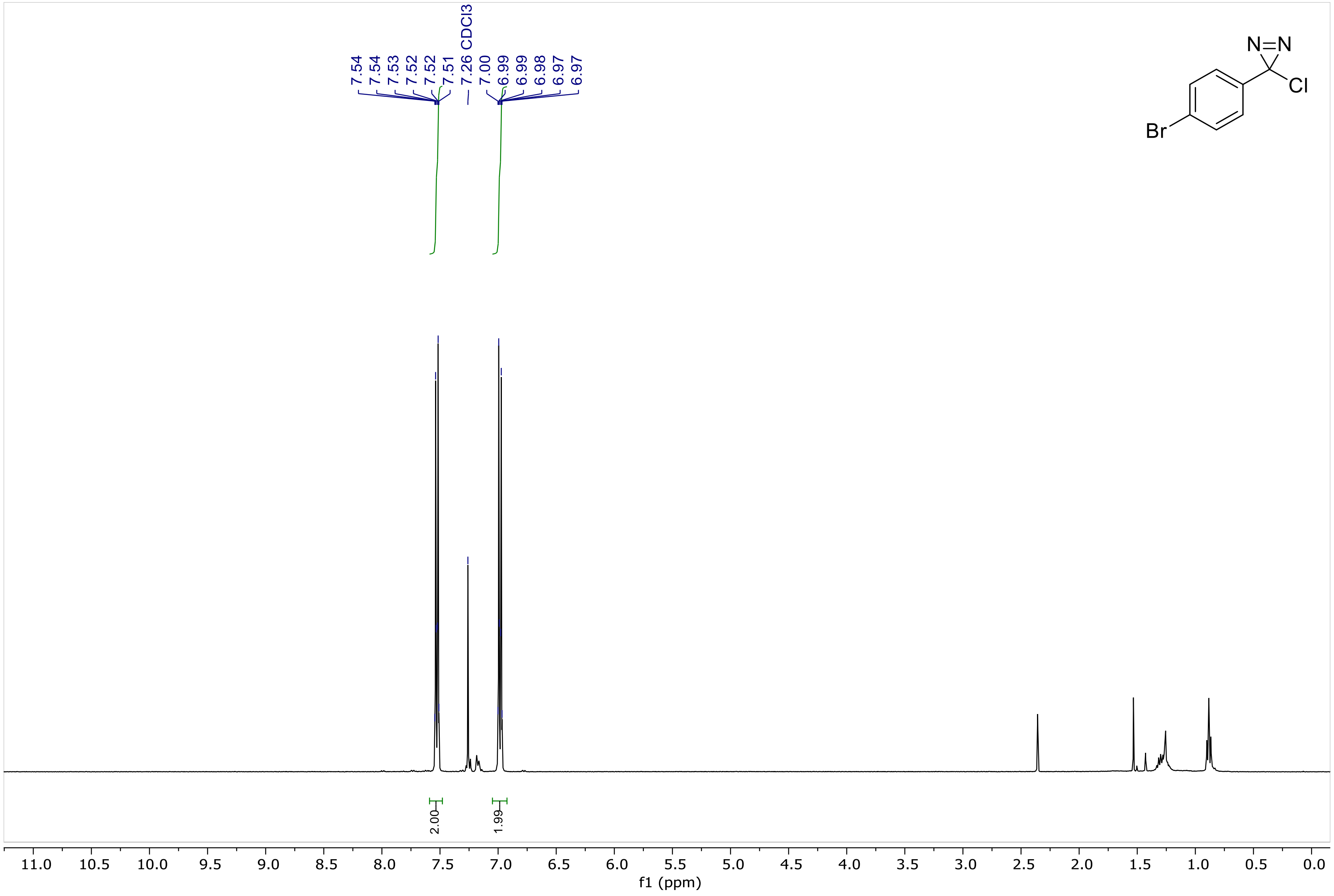

3-(4-Bromophenyl)-3-chloro-3*H*-diazirine – $^{13}C\{^1H\}$ NMR (101 MHz, $CDCl_3$)

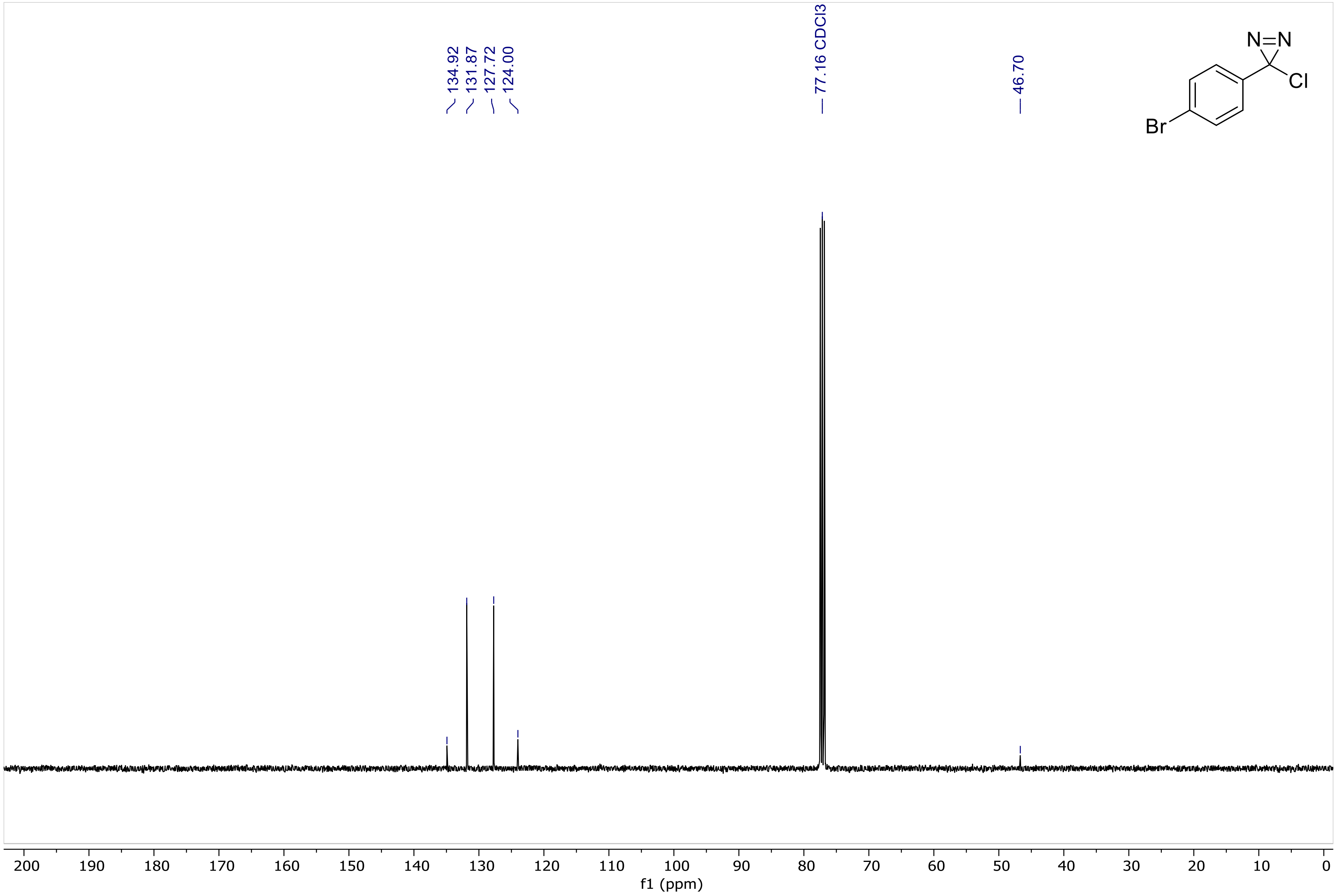

3-(4-Nitrophenyl)-3-chloro-3*H*-diazirine – ^{1}H NMR (400 MHz, $CDCl_3$)

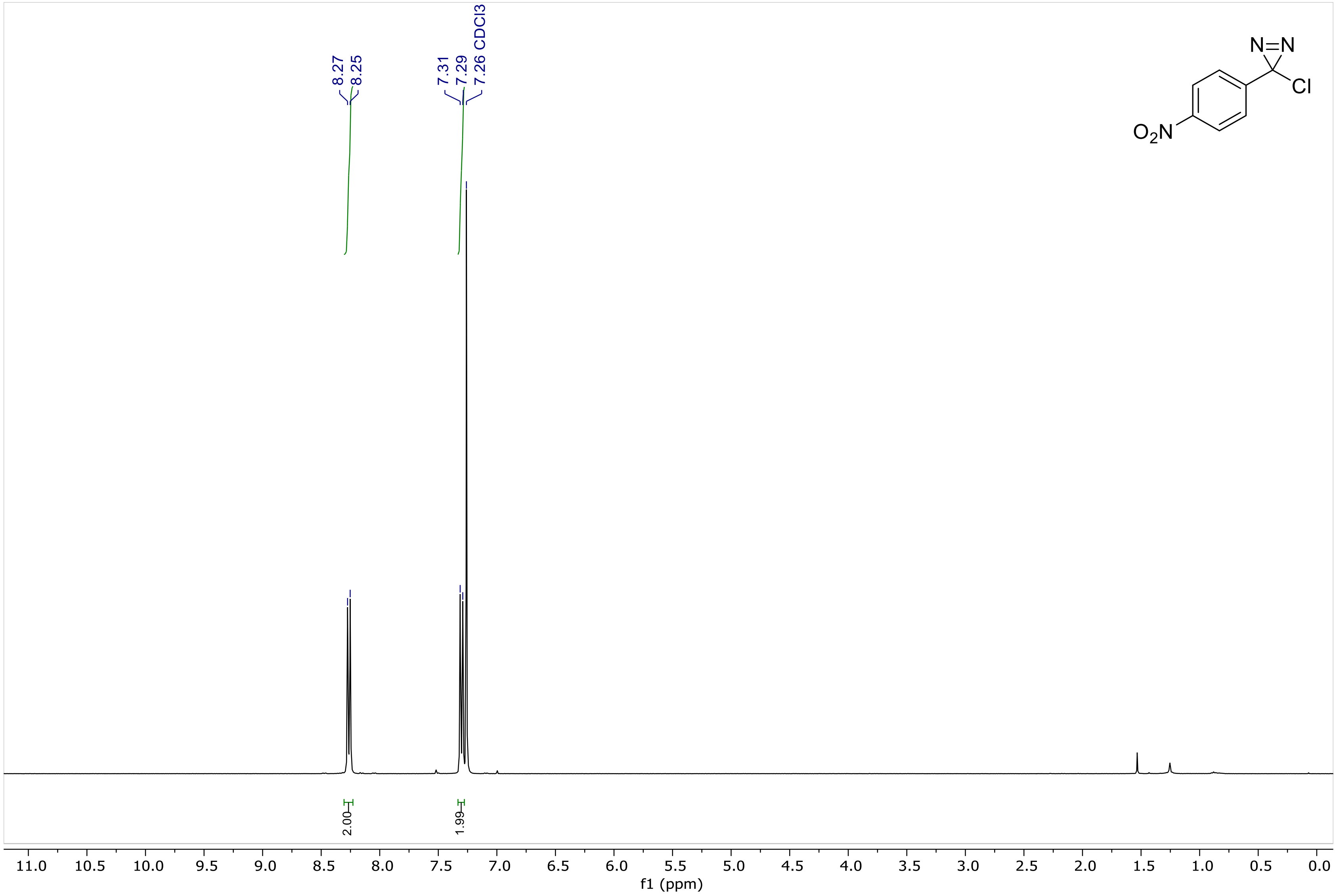

3-(4-Nitrophenyl)-3-chloro-3*H*-diazirine – $^{13}C\{^{1}H\}$ NMR (101 MHz, $CDCl_3$)

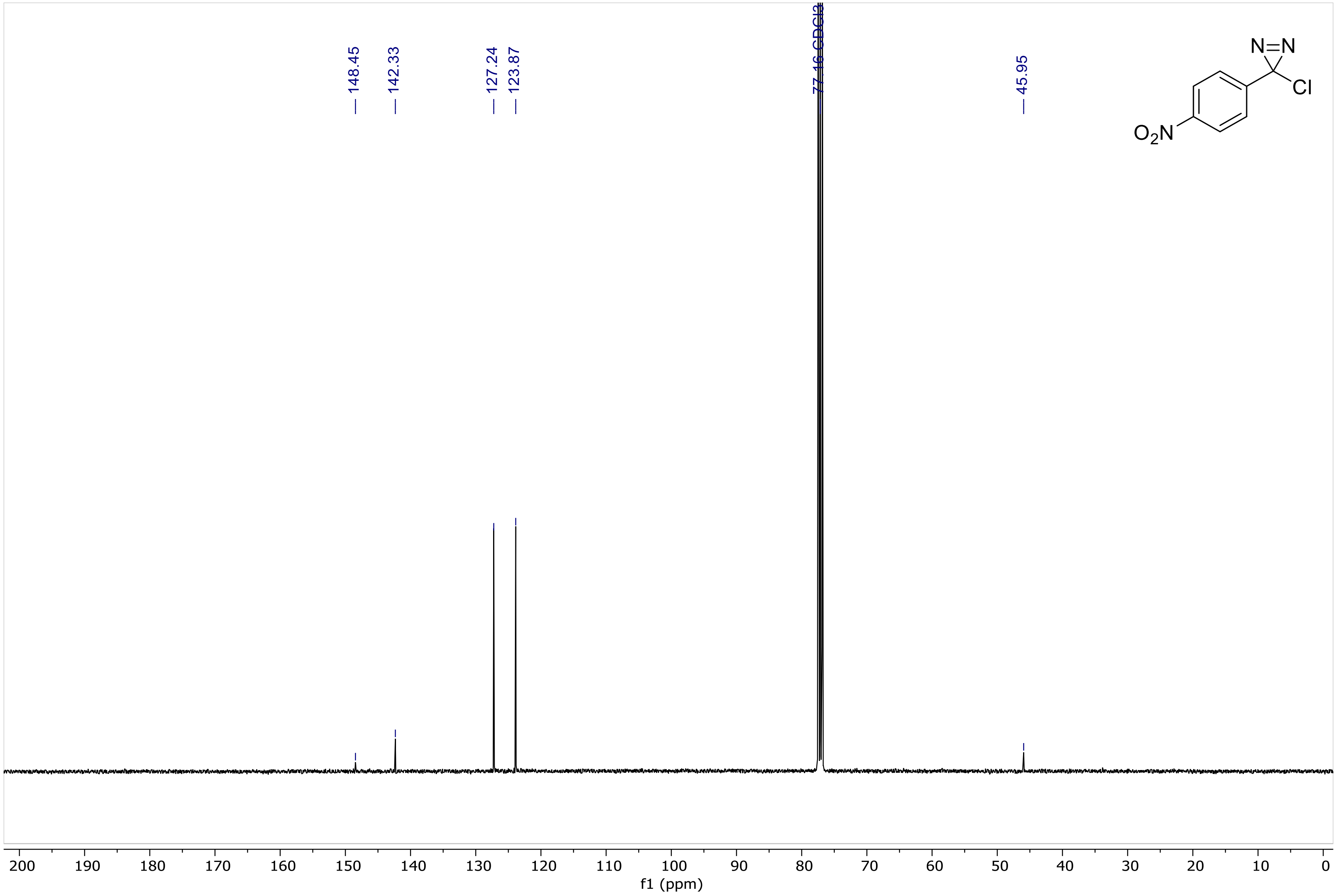

3-(3-Bromophenyl)-3-chloro-3H-diazirine – ^{1}H NMR (400 MHz, $CDCl_3$)

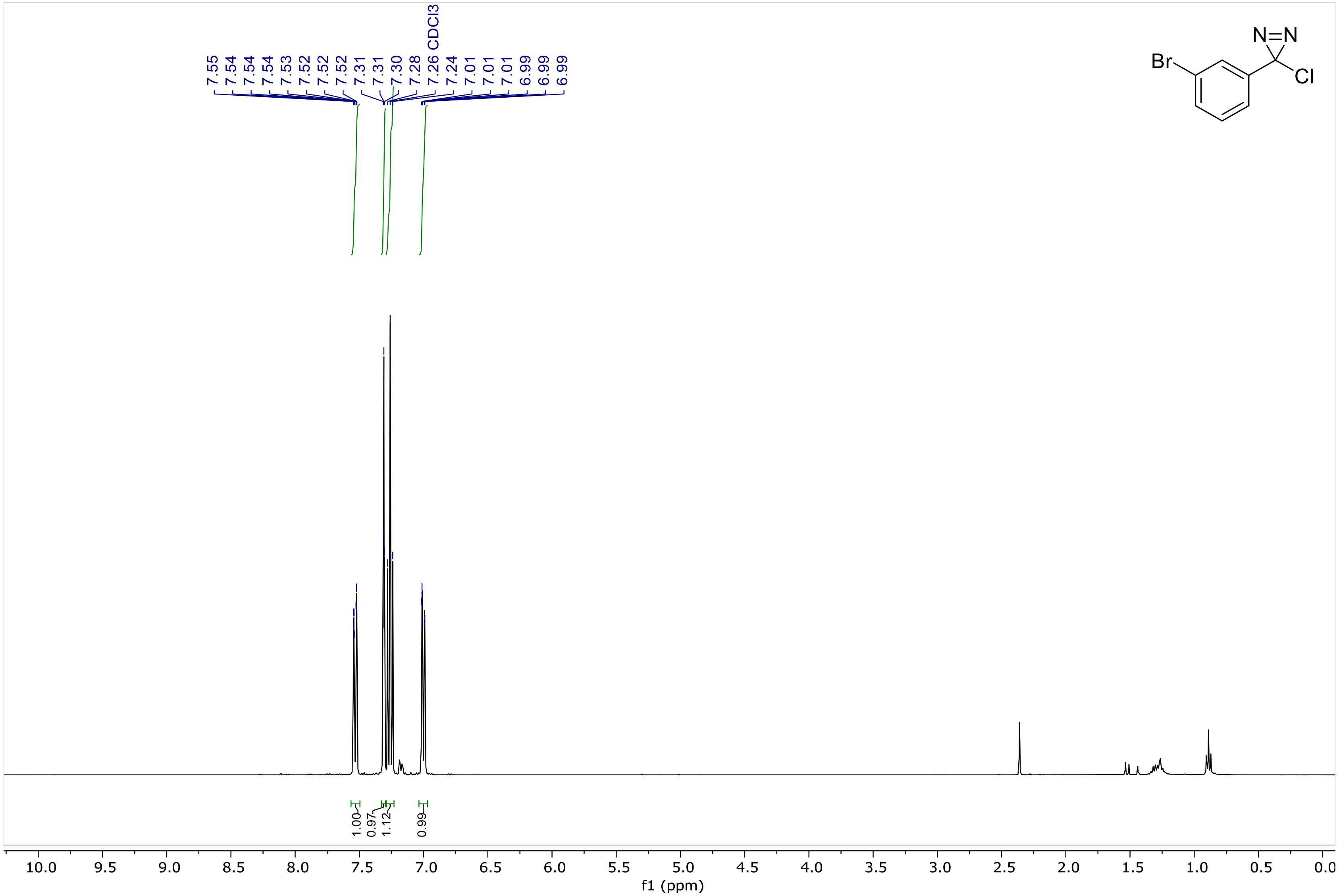

3-(3-Bromophenyl)-3-chloro-3*H*-diazirine – $^{13}C\{^1H\}$ NMR (101 MHz, $CDCl_3$)

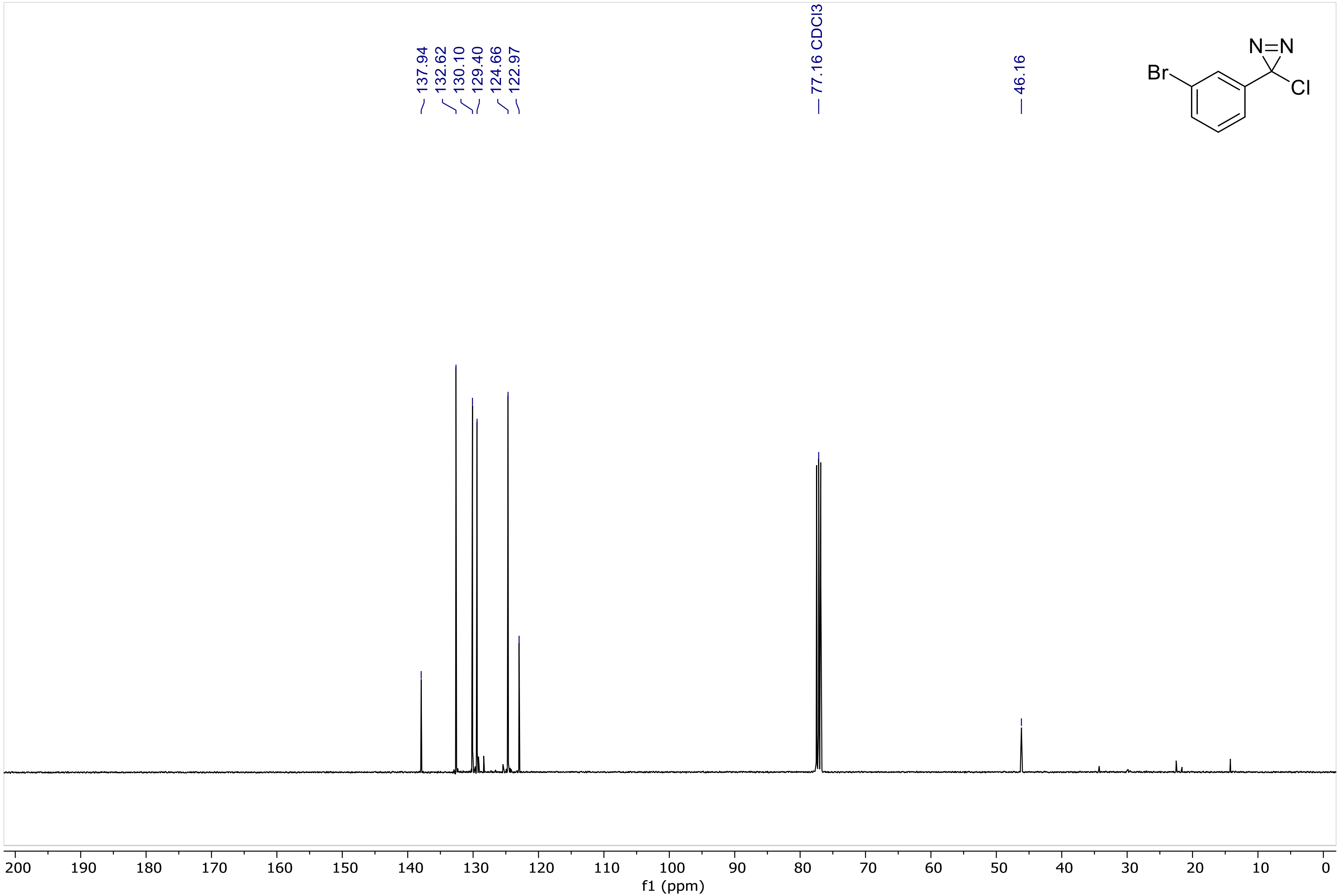

3-(2-trifluoromethoxyphenyl)-3-chloro-3*H*-diazirine – ^{1}H NMR (400 MHz, $CDCl_3$)

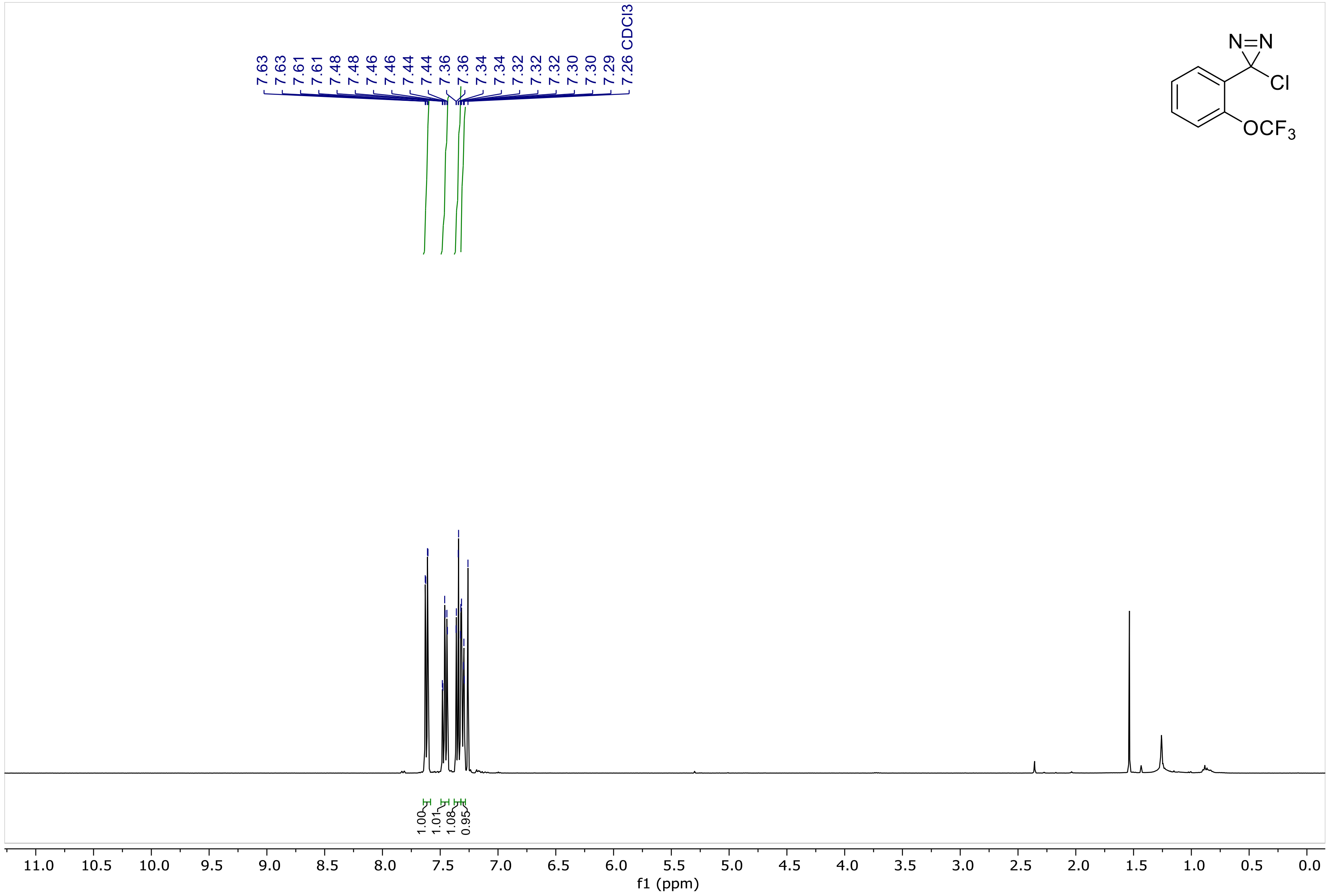

3-(2-trifluoromethoxyphenyl)-3-chloro-3*H*-diazirine – $^{13}C\{^1H\}$ NMR (101 MHz, $CDCl_3$)

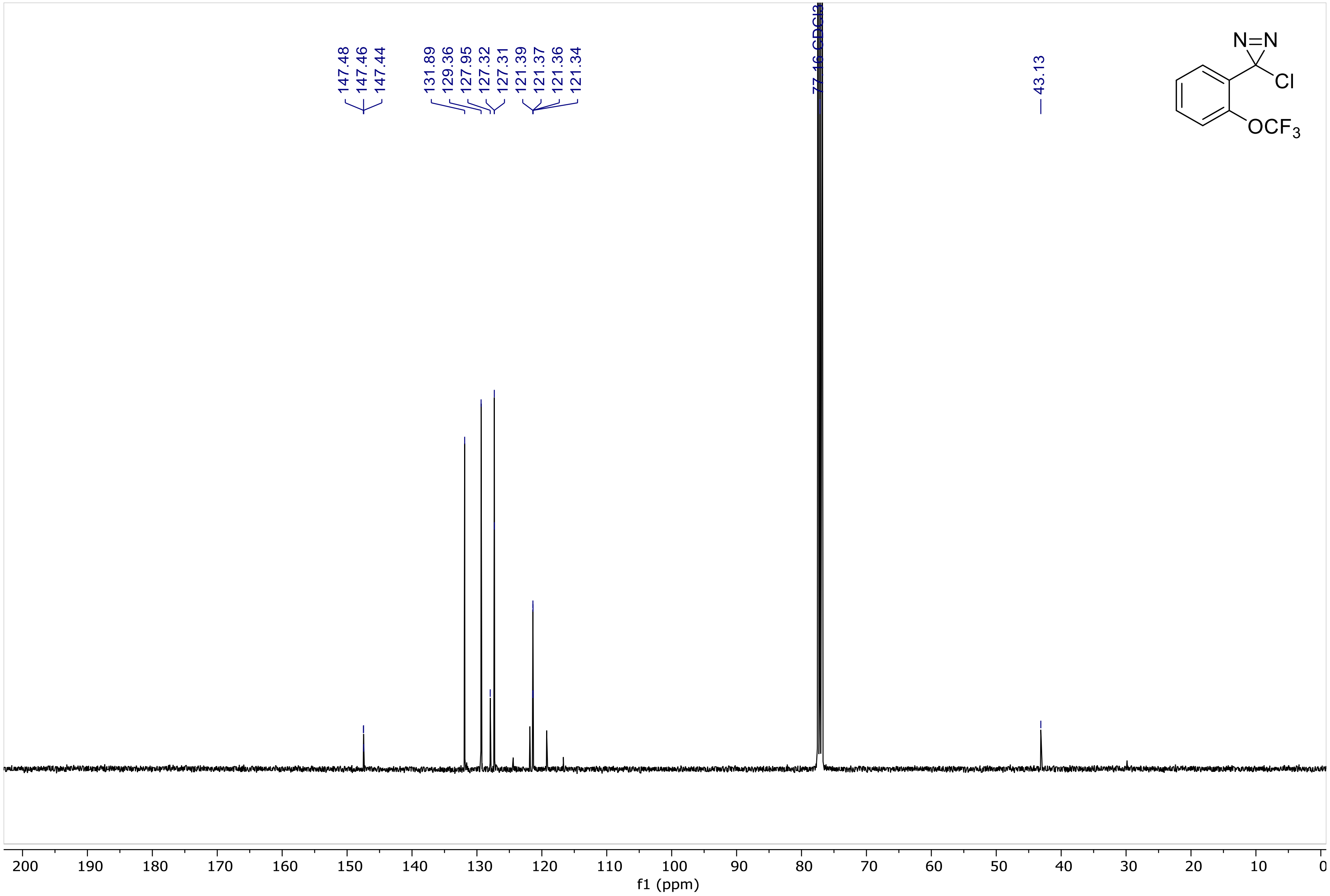

3-(2-trifluoromethoxyphenyl)-3-chloro-3*H*-diazirine – ^{19}F NMR (376 MHz, $CDCl_3$)

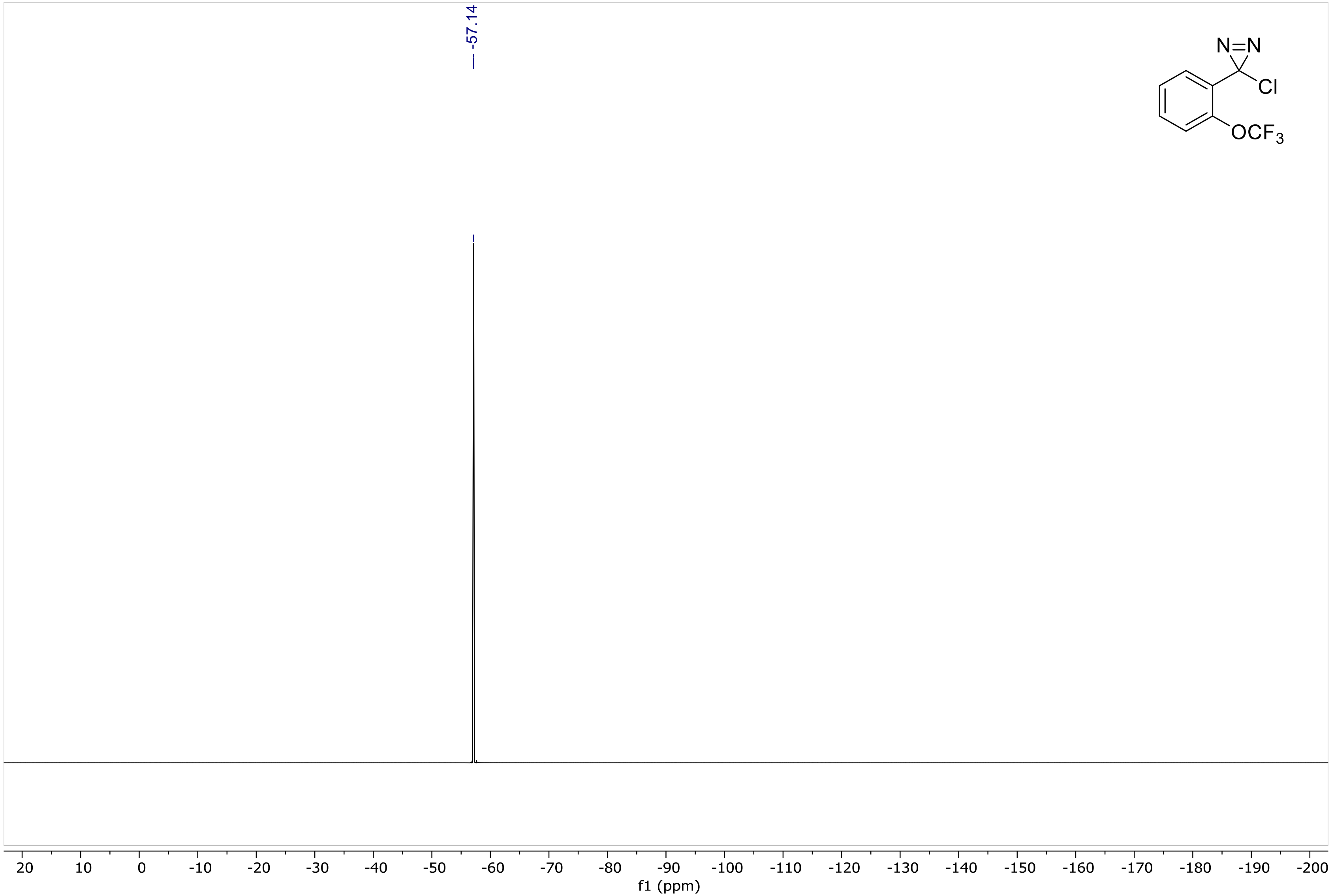

3-(2-Fluoro-5-chloro)-3-chloro-3*H*-diazirine – ^{1}H NMR (400 MHz, $CDCl_3$)

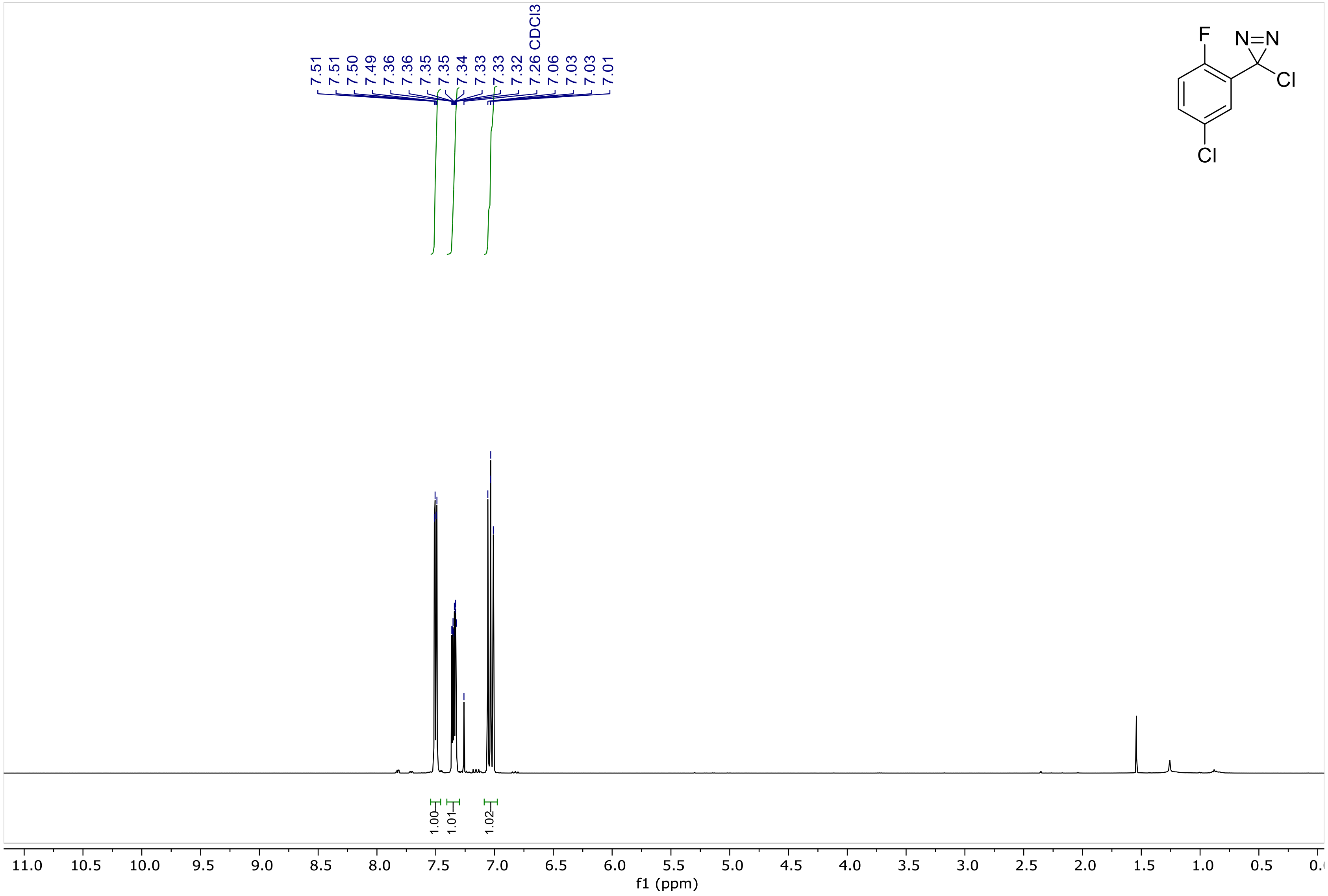

3-(2-Fluoro-5-chloro)-3-chloro-3*H*-diazirine – $^{13}C\{^1H\}$ NMR (101 MHz, $CDCl_3$)

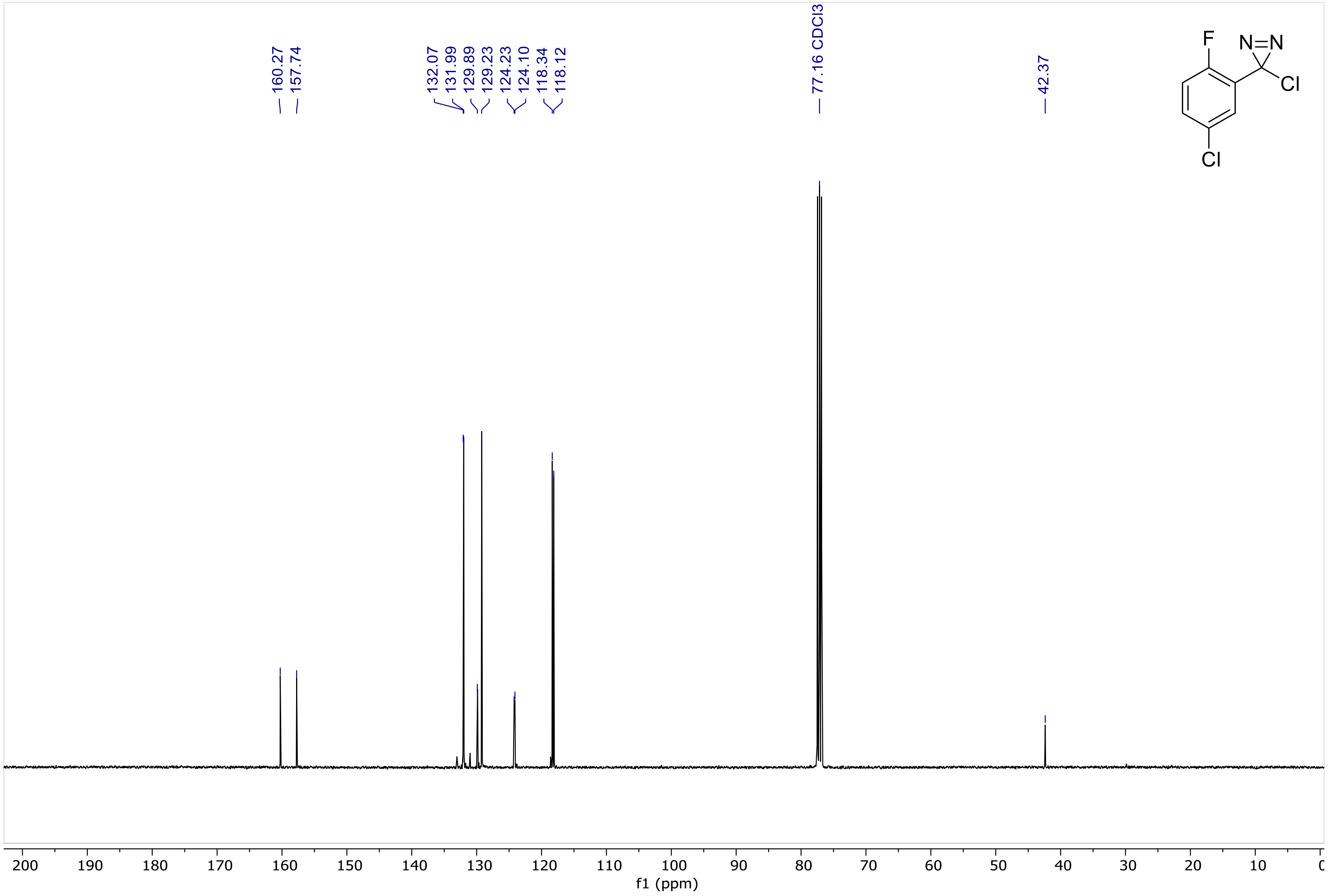

3-(2-Pyridyl)-3-chloro-3*H*-diazirine – ^{1}H NMR (400 MHz, $CDCl_3$)

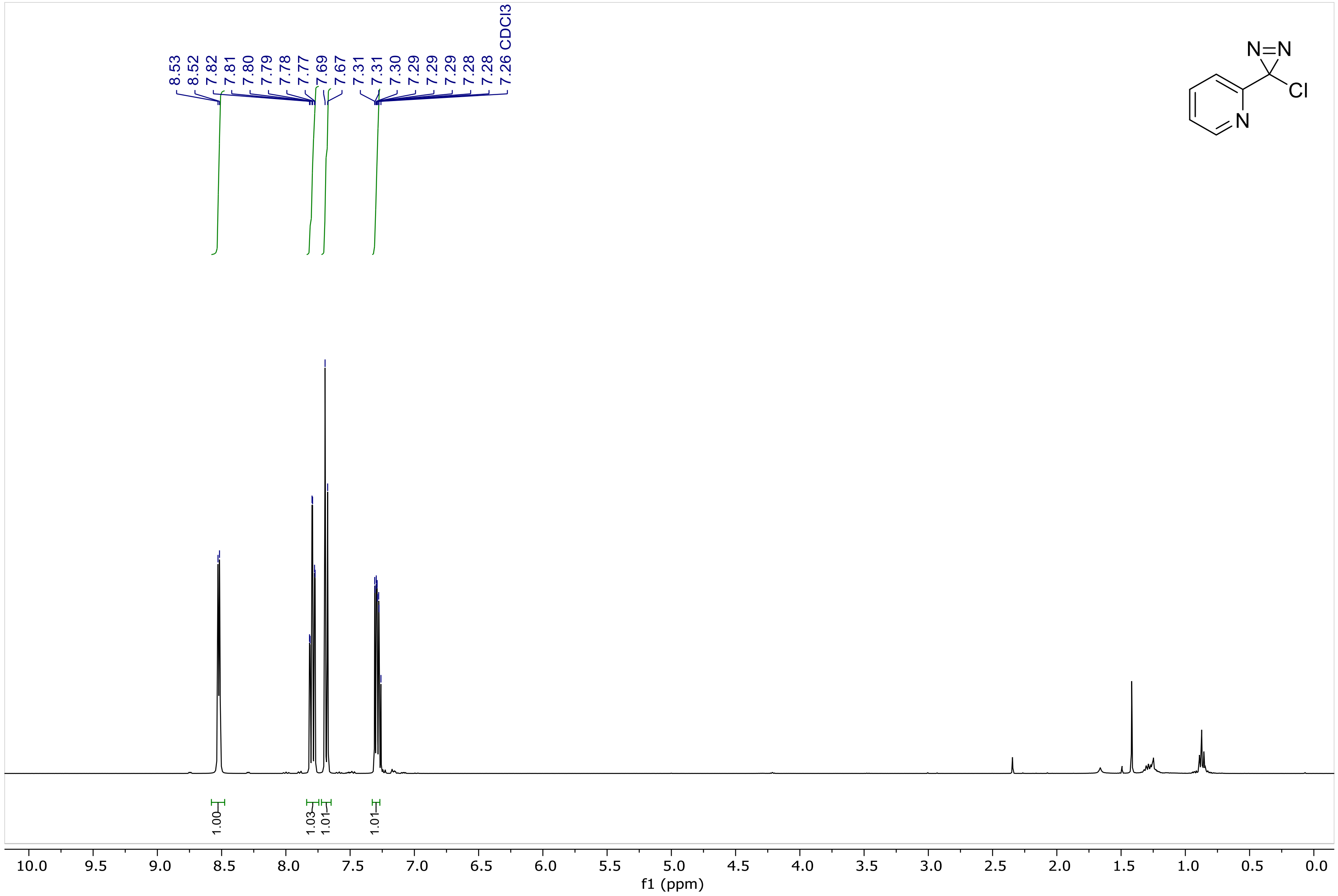

3-(2-Pyridyl)-3-chloro-3*H*-diazirine – $^{13}C\{^1H\}$ NMR (101 MHz, $CDCl_3$)

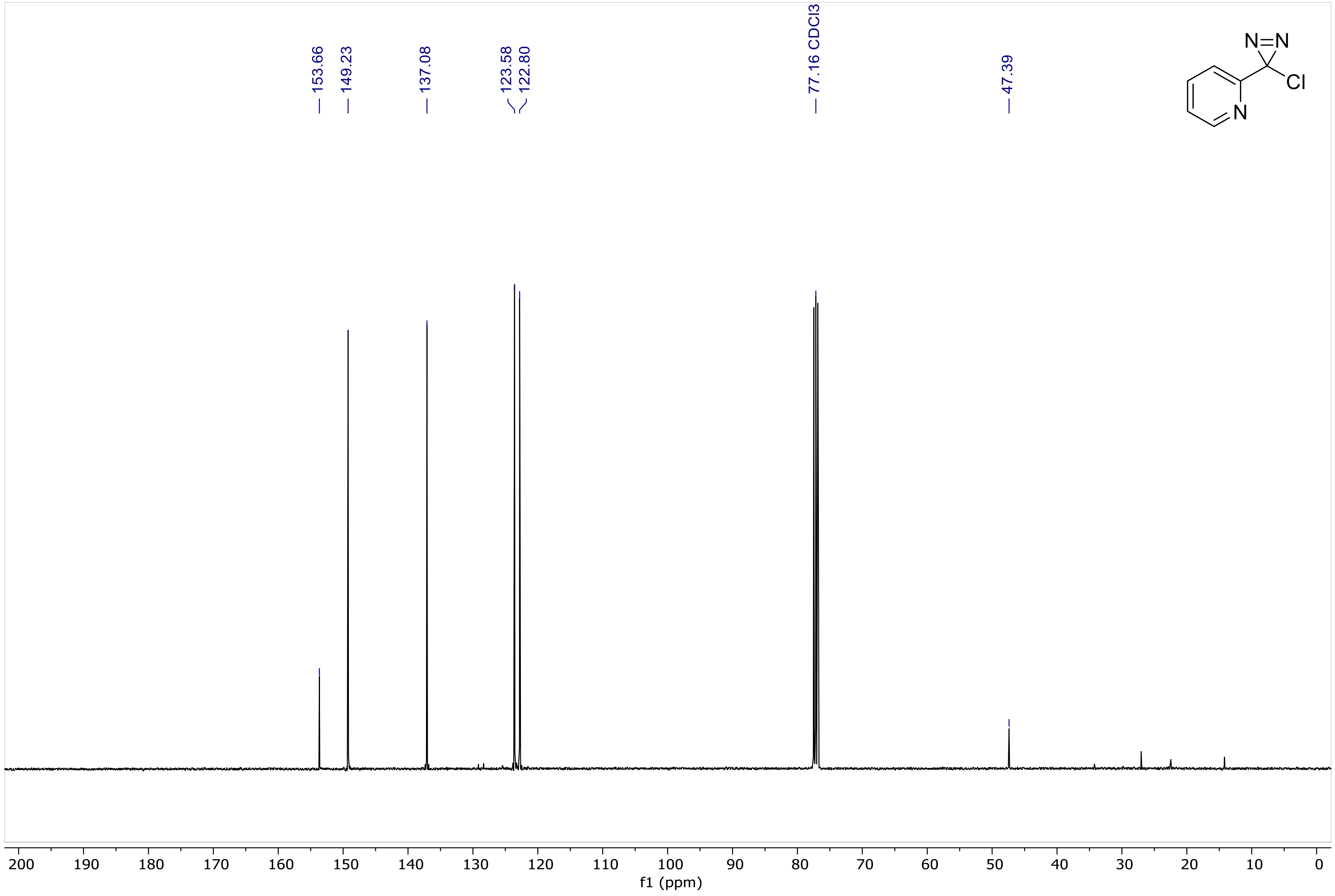

4-Fluorobenzamidine hydrochloride – ^{1}H NMR (400 MHz, DMSO-d_6)

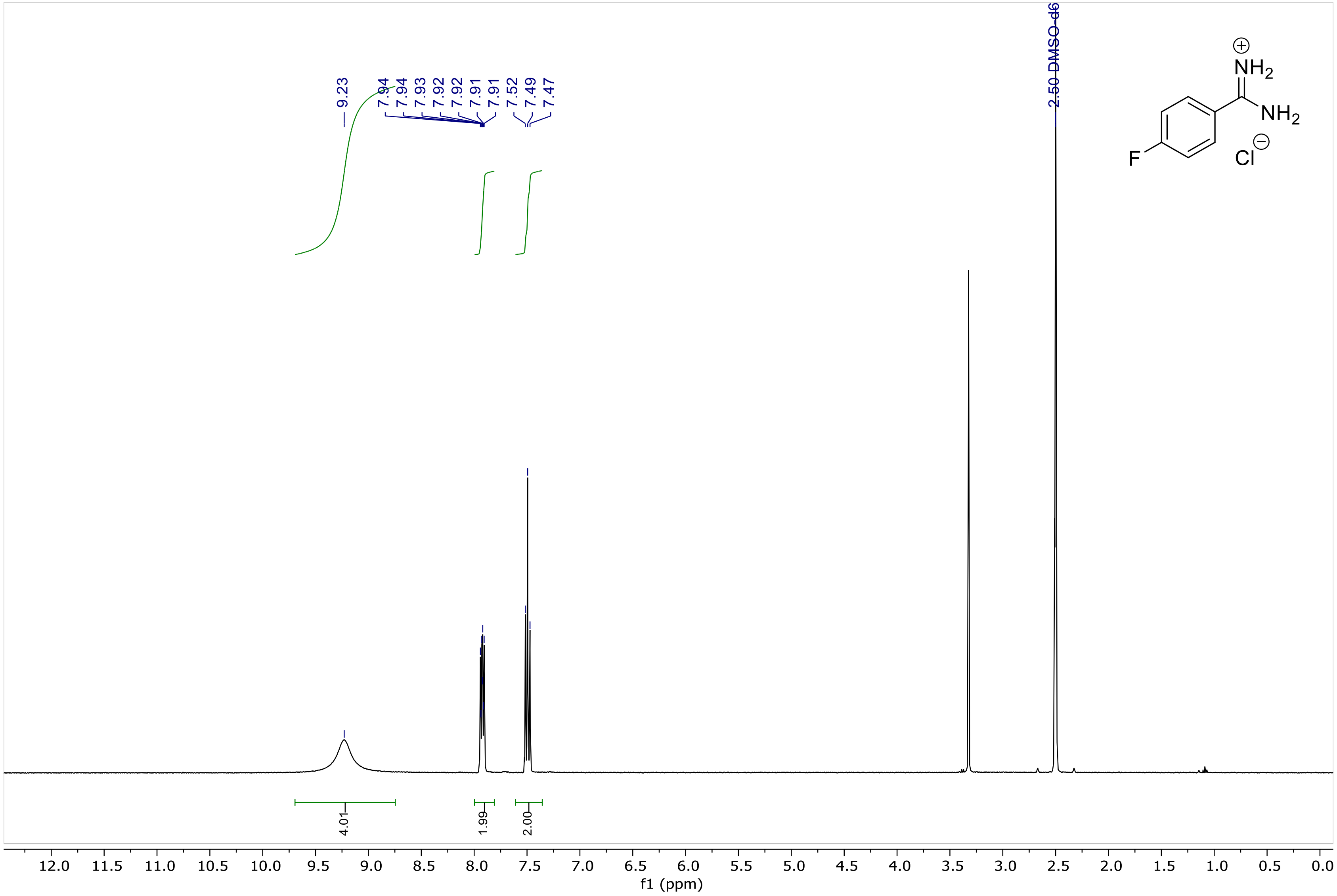

4-Fluorobenzamidine hydrochloride – $^{13}C\{^{1}H\}$ NMR (101 MHz, DMSO-d_6)

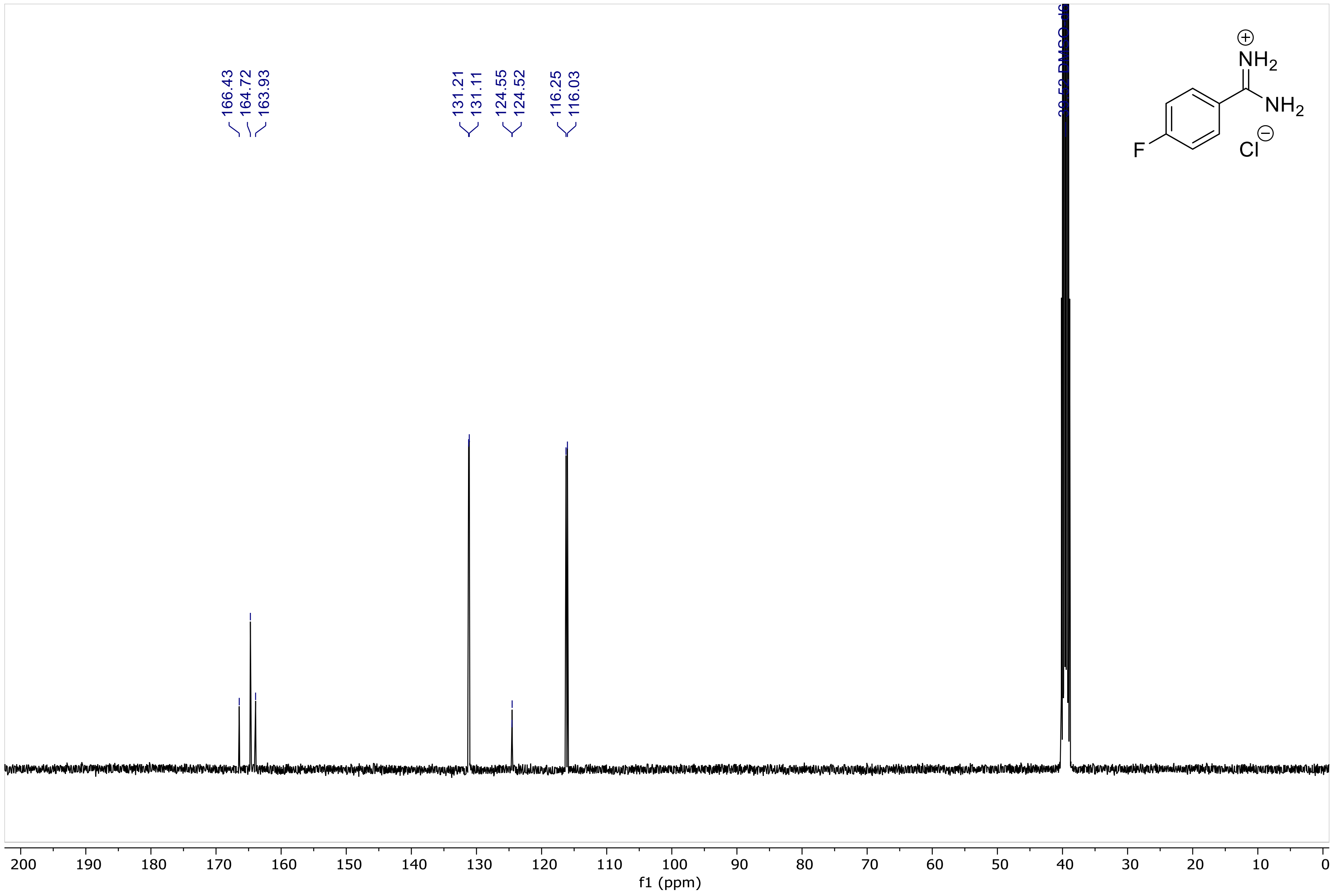

4-Fluorobenzamidine hydrochloride – ^{19}F NMR (376 MHz, DMSO-d_6)

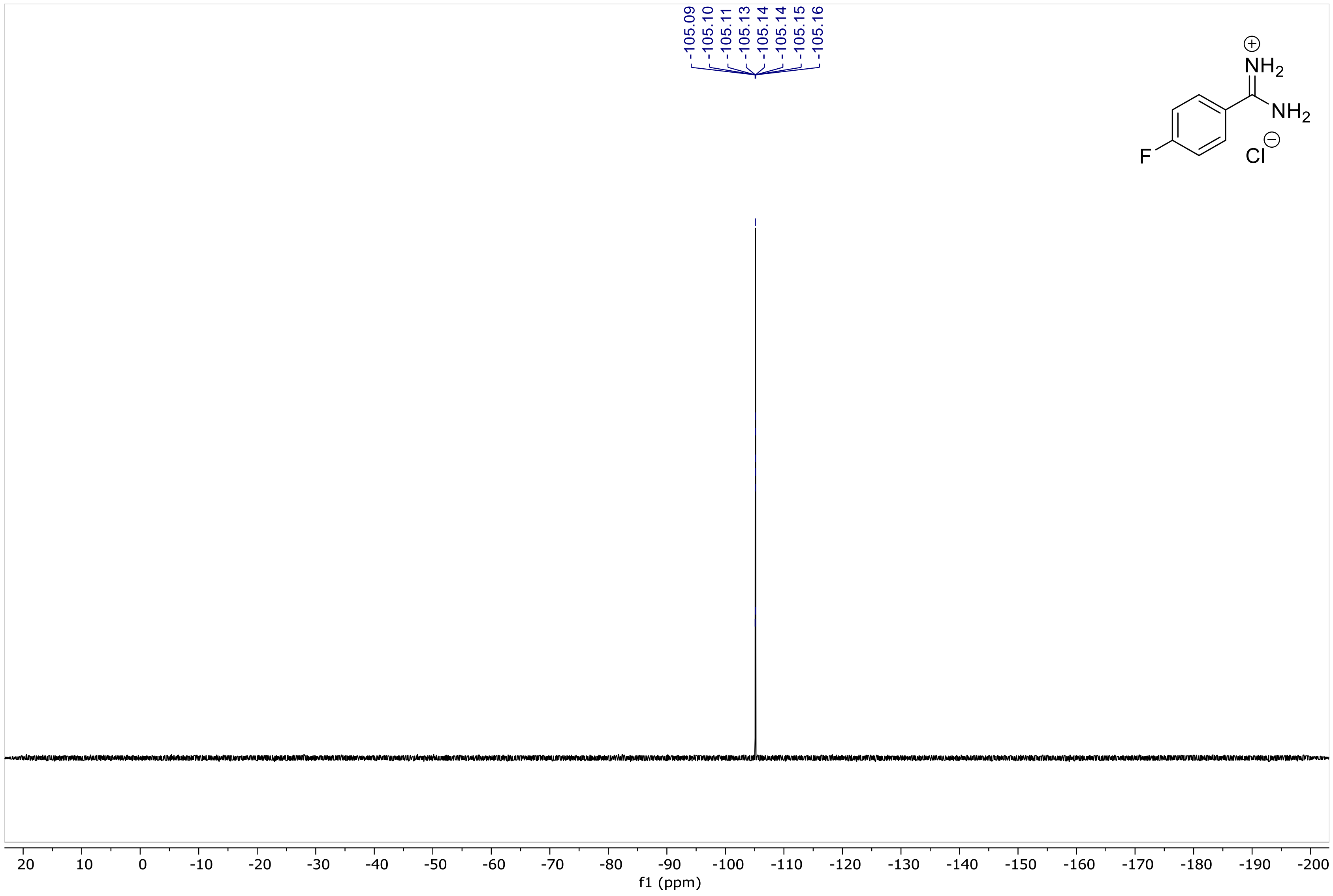

4-Chlorobenzamidine hydrochloride – ^{1}H NMR (400 MHz, DMSO-d_6)

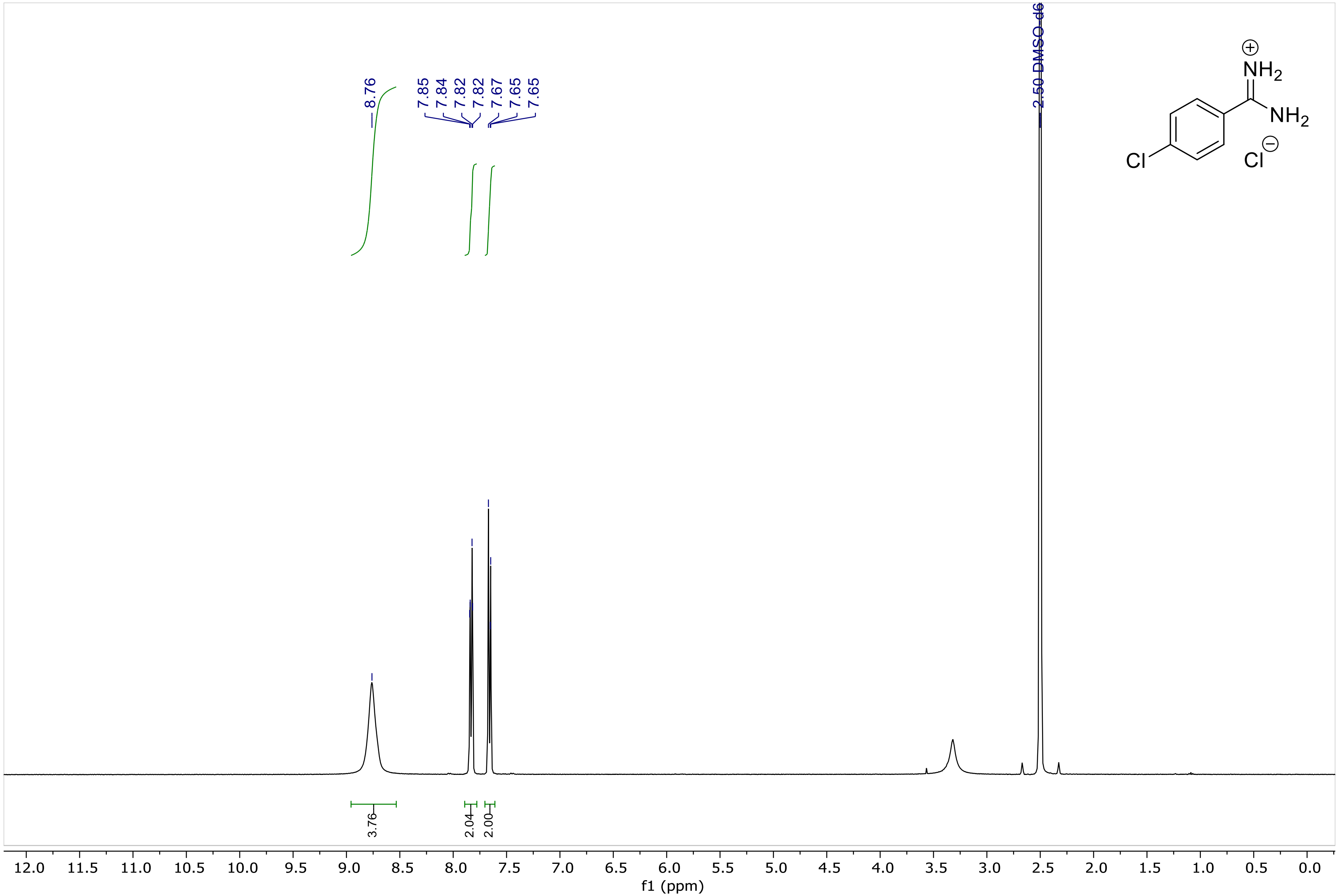

4-Chlorobenzamidine hydrochloride – $^{13}C\{^{1}H\}$ NMR (101 MHz, DMSO-d_6)

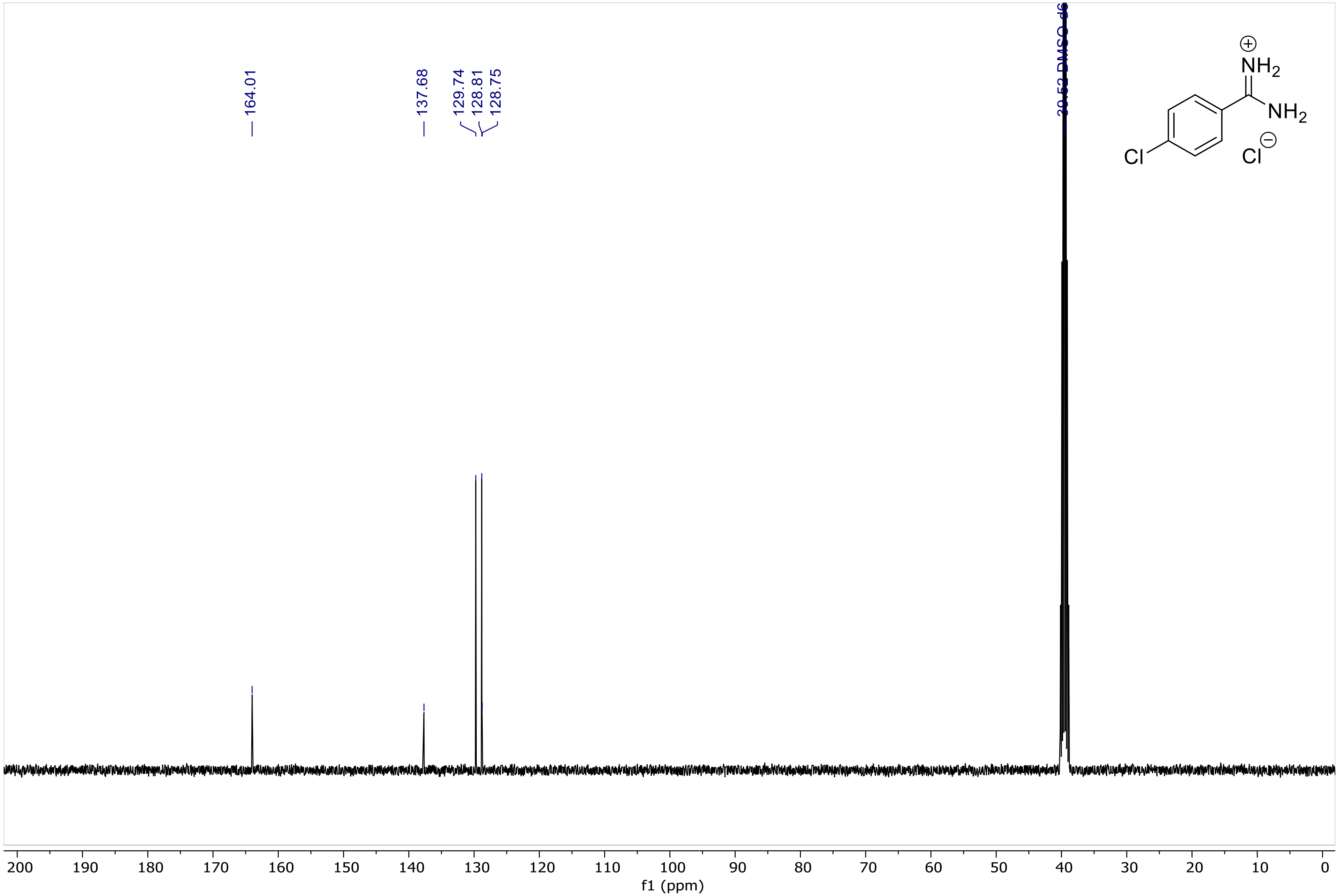

4-Bromobenzamidine hydrochloride – ^{1}H NMR (400 MHz, DMSO-d_6)

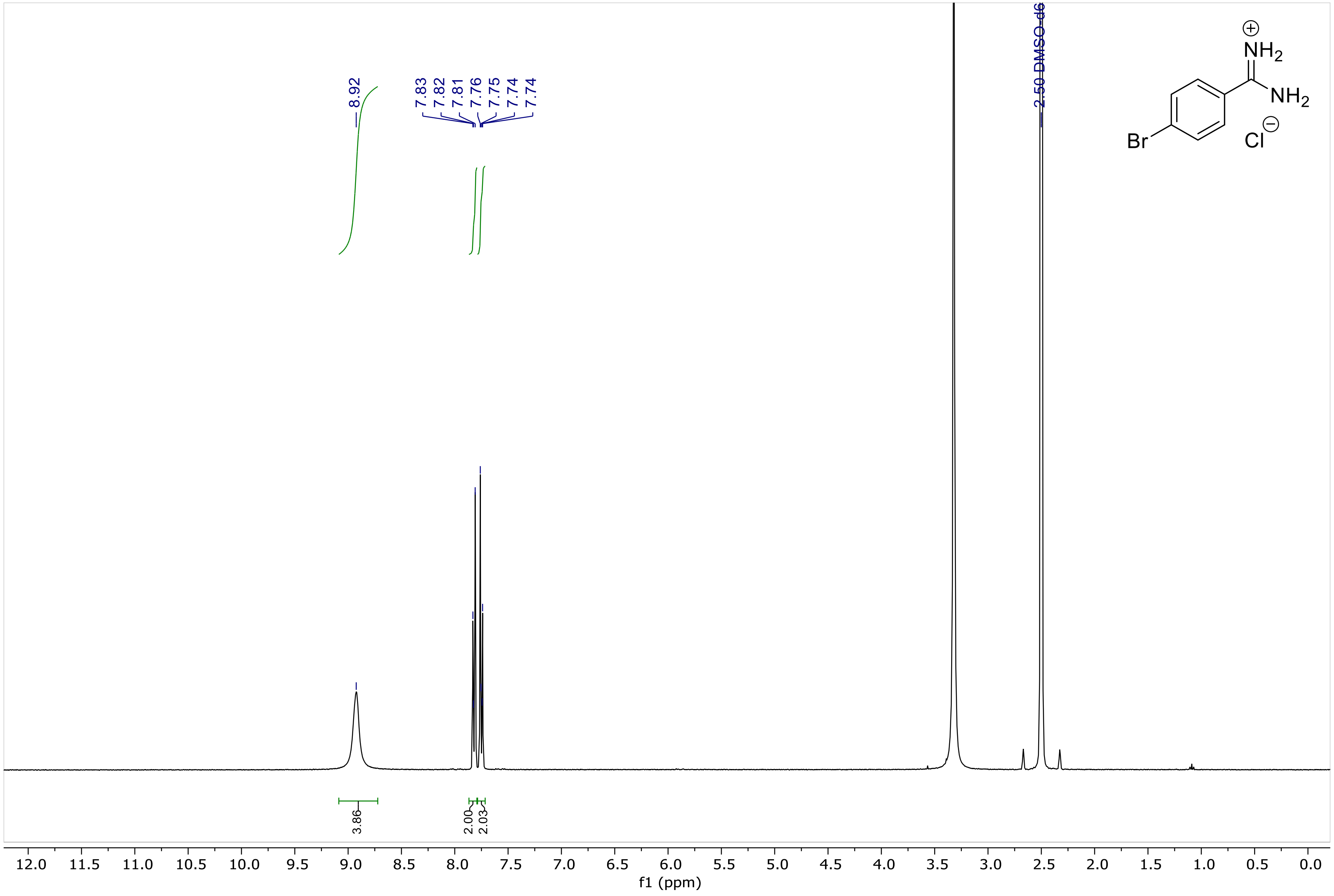

4-Bromobenzamidine hydrochloride – $^{13}C\{^{1}H\}$ NMR (101 MHz, DMSO-d_6)

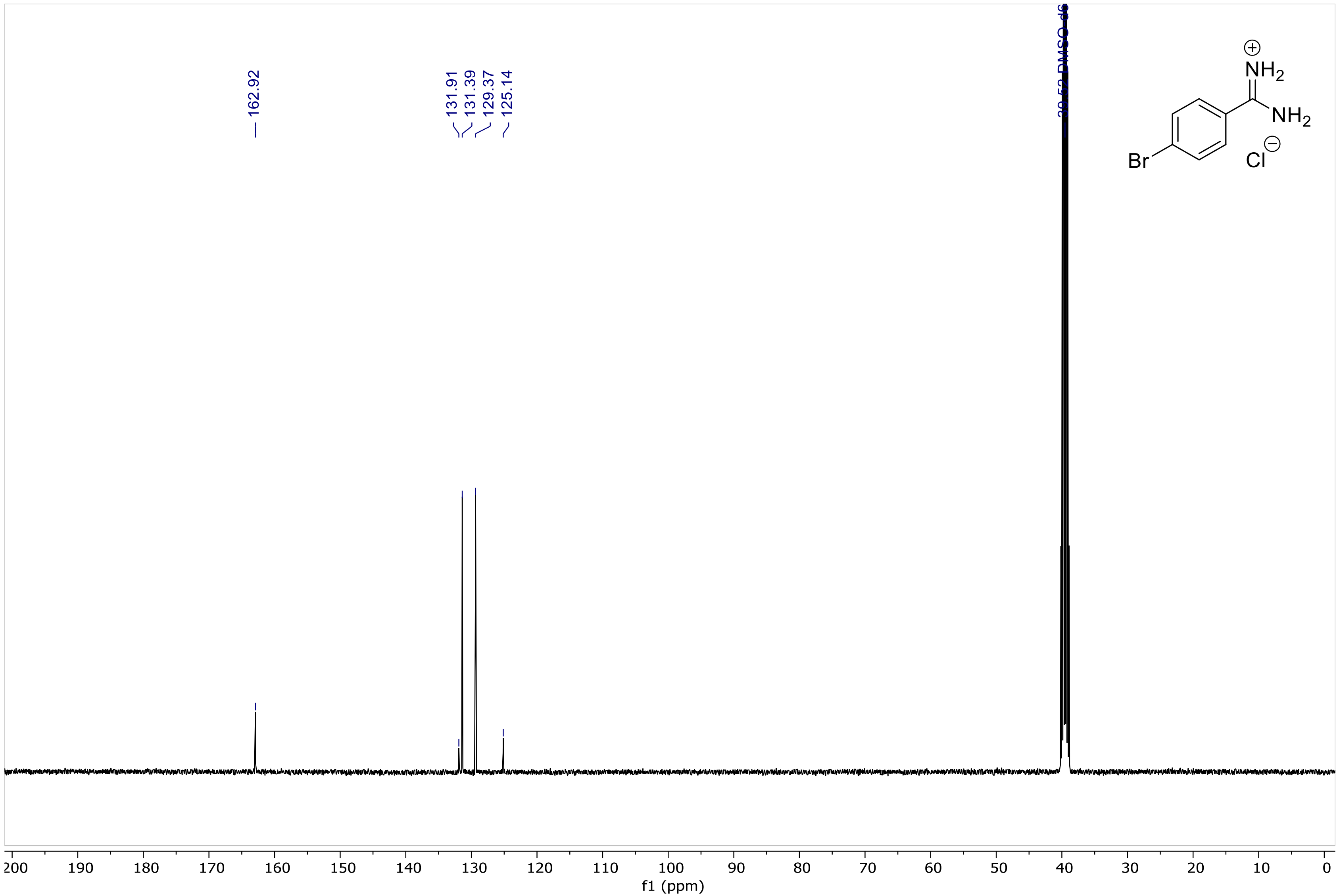

3-Bromobenzamidine hydrochloride – ^{1}H NMR (400 MHz, DMSO-d_6)

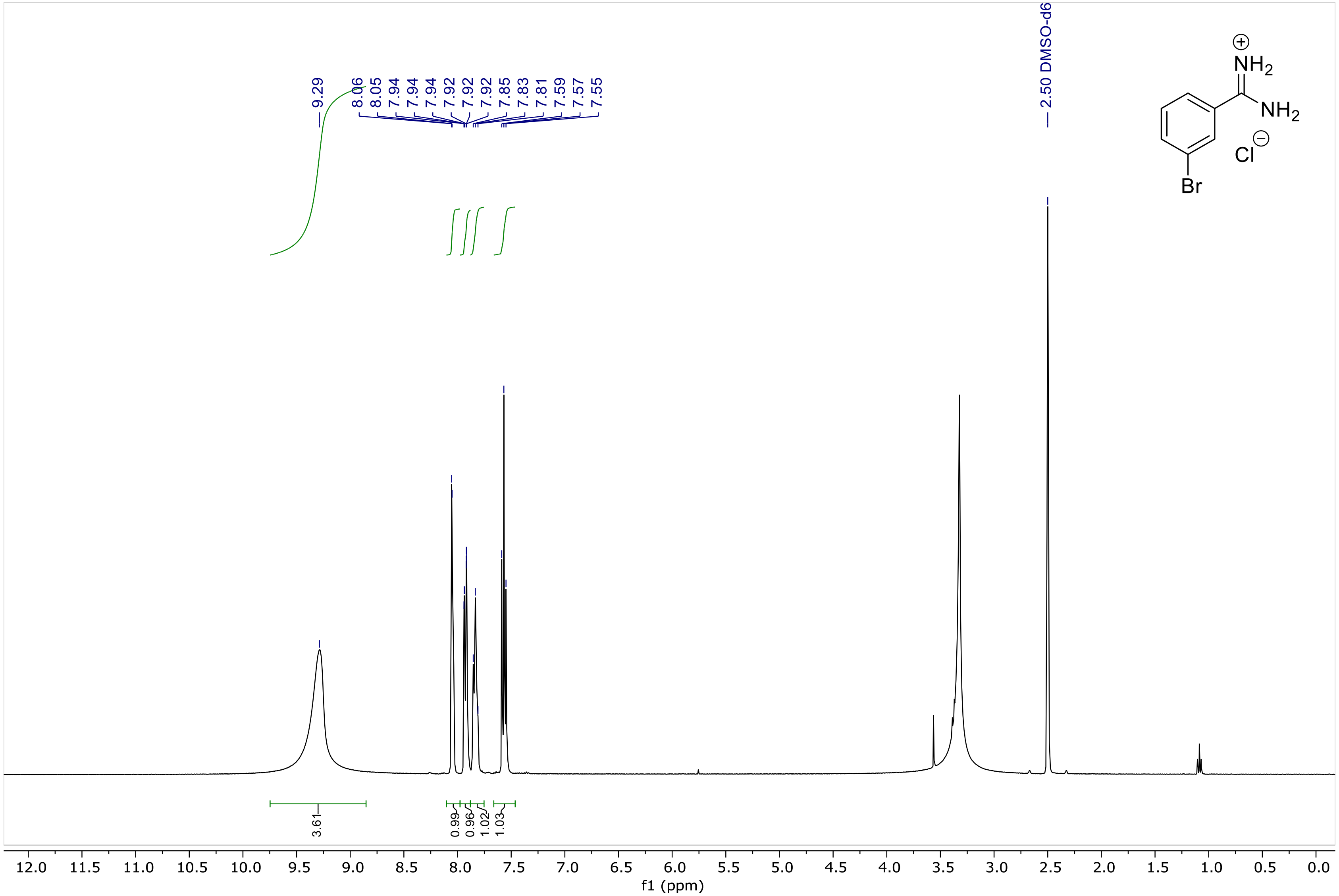

3-Bromobenzamidine hydrochloride – $^{13}C\{^1H\}$ NMR (101 MHz, DMSO-d_6)

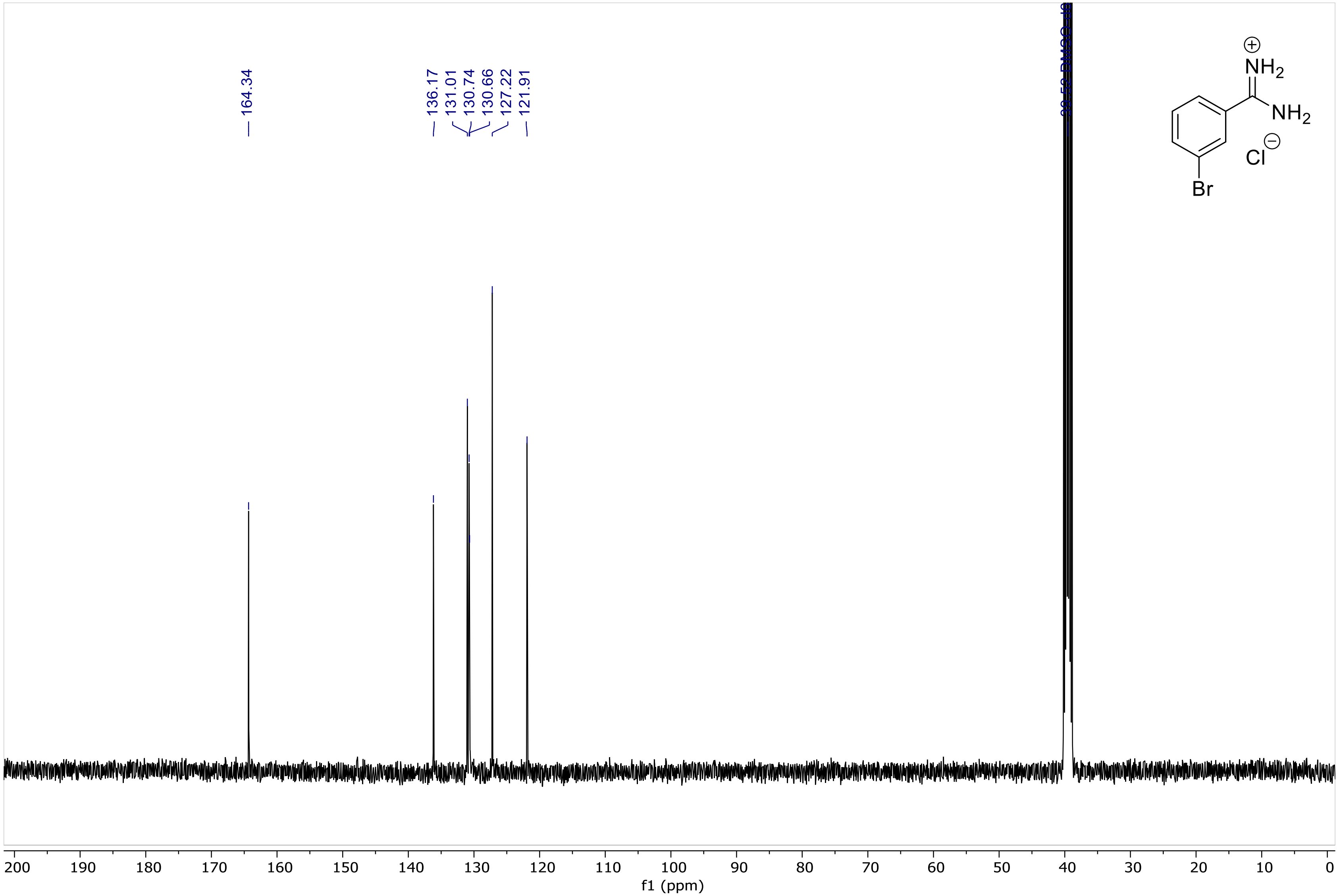

2-(Trifluoromethoxy)benzamidine hydrochloride – ^{1}H NMR (400 MHz, DMSO-d_6)

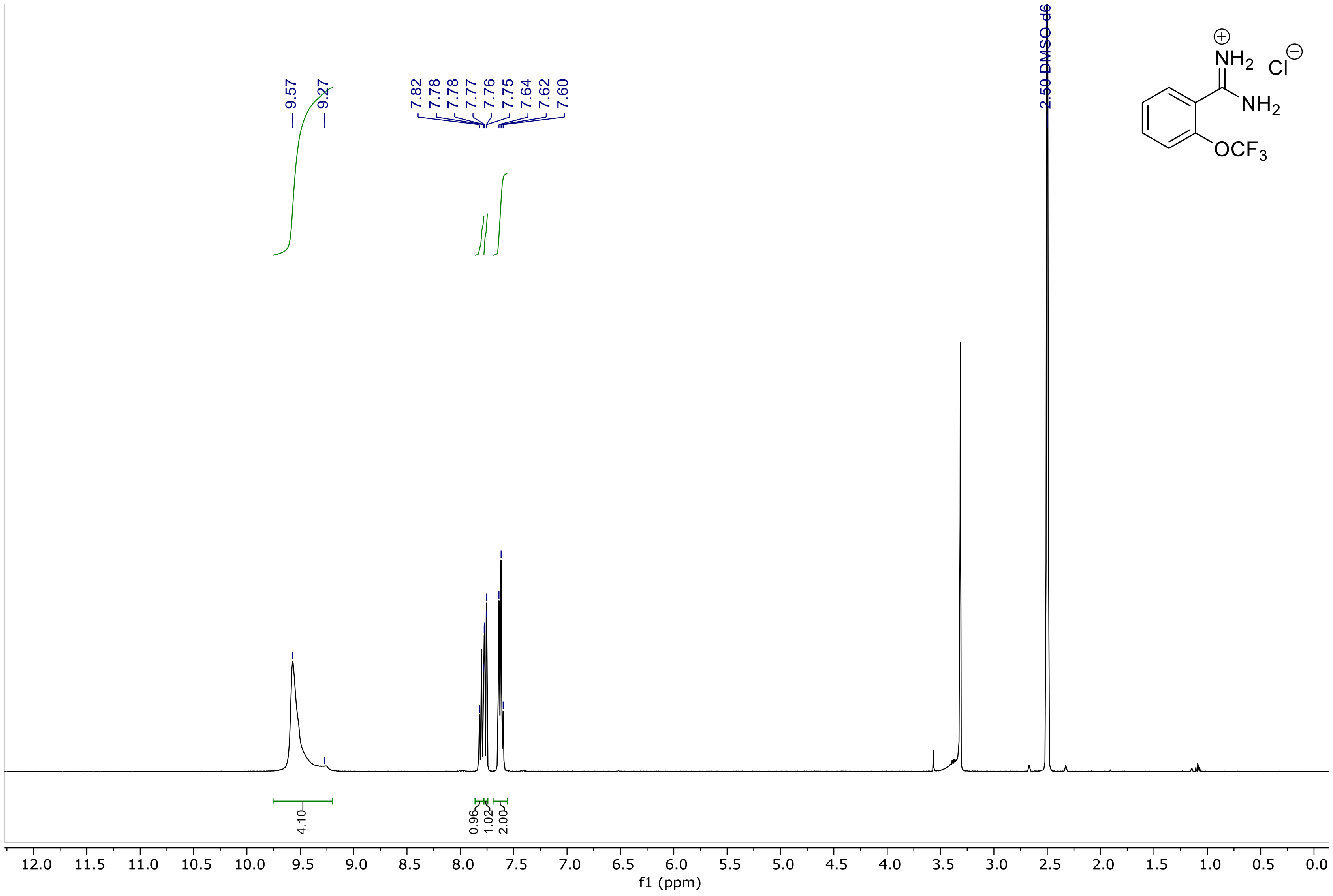

2-(Trifluoromethoxy)benzamidine hydrochloride – $^{13}C\{^1H\}$ NMR (101 MHz, DMSO-d_6)

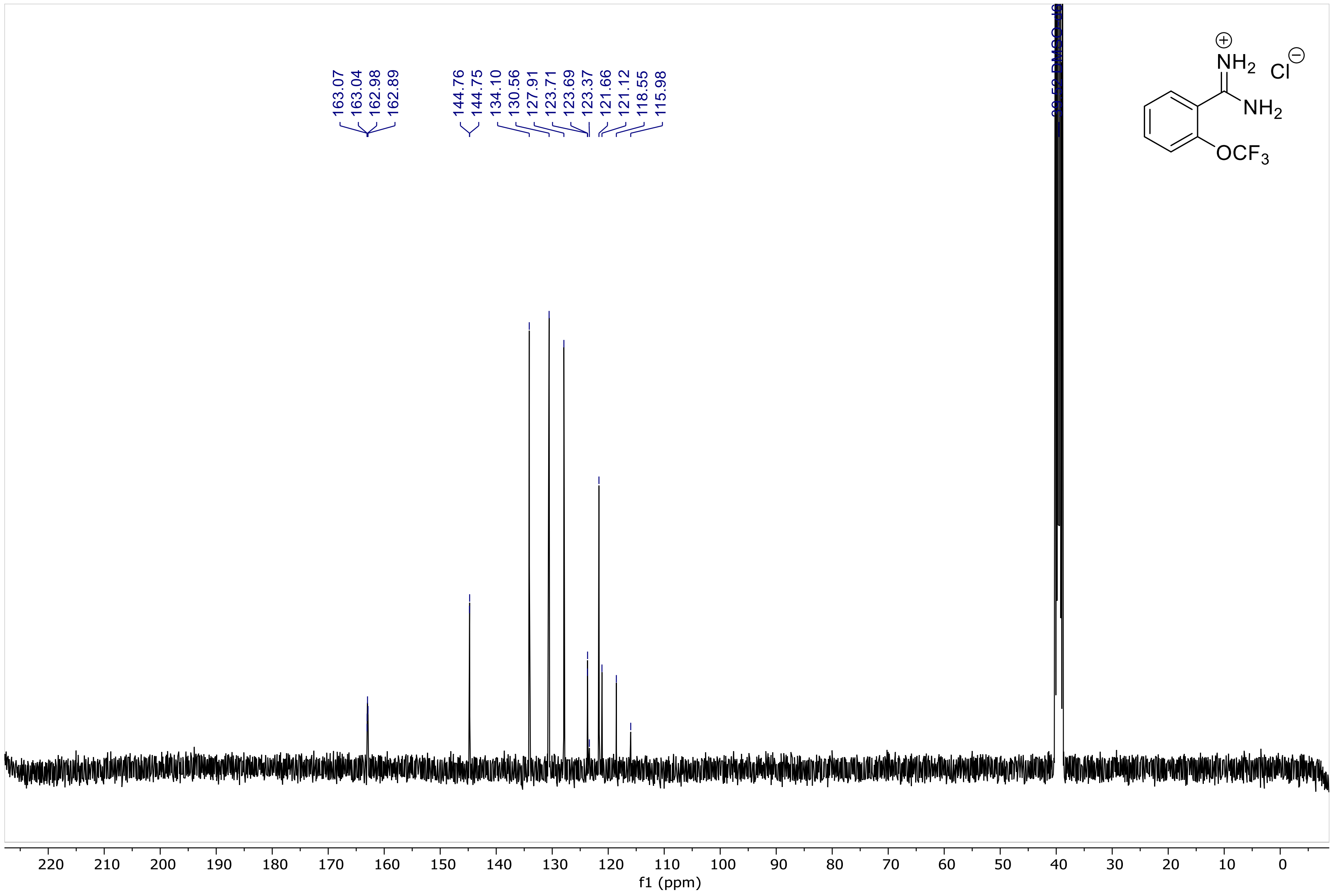

2-(Trifluoromethoxy)benzamidine hydrochloride – ^{19}F NMR (376 MHz, DMSO-d_6)

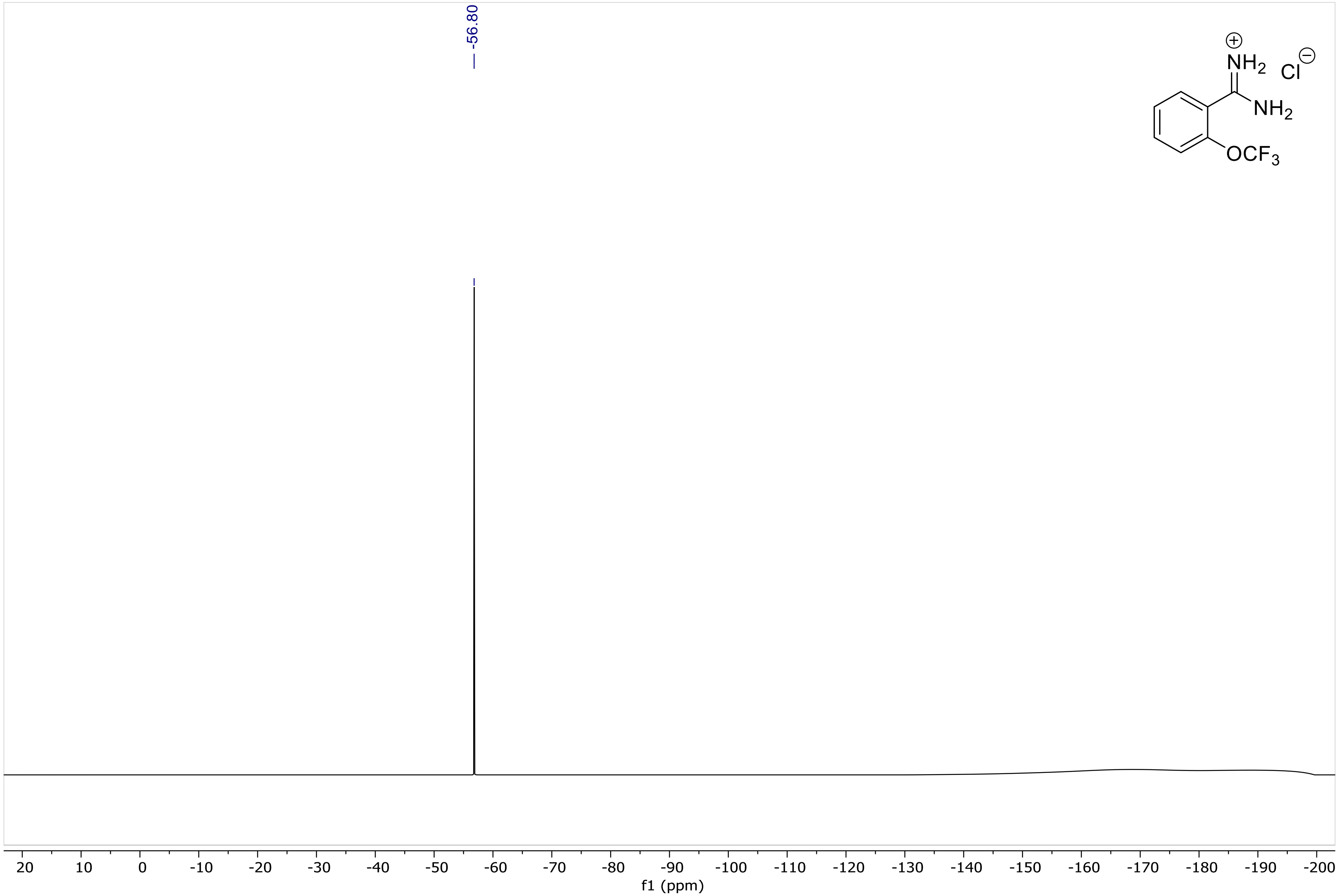

2-Fluoro-5-chlorobenzamidine hydrochloride – ^{1}H NMR (400 MHz, DMSO-d_6)

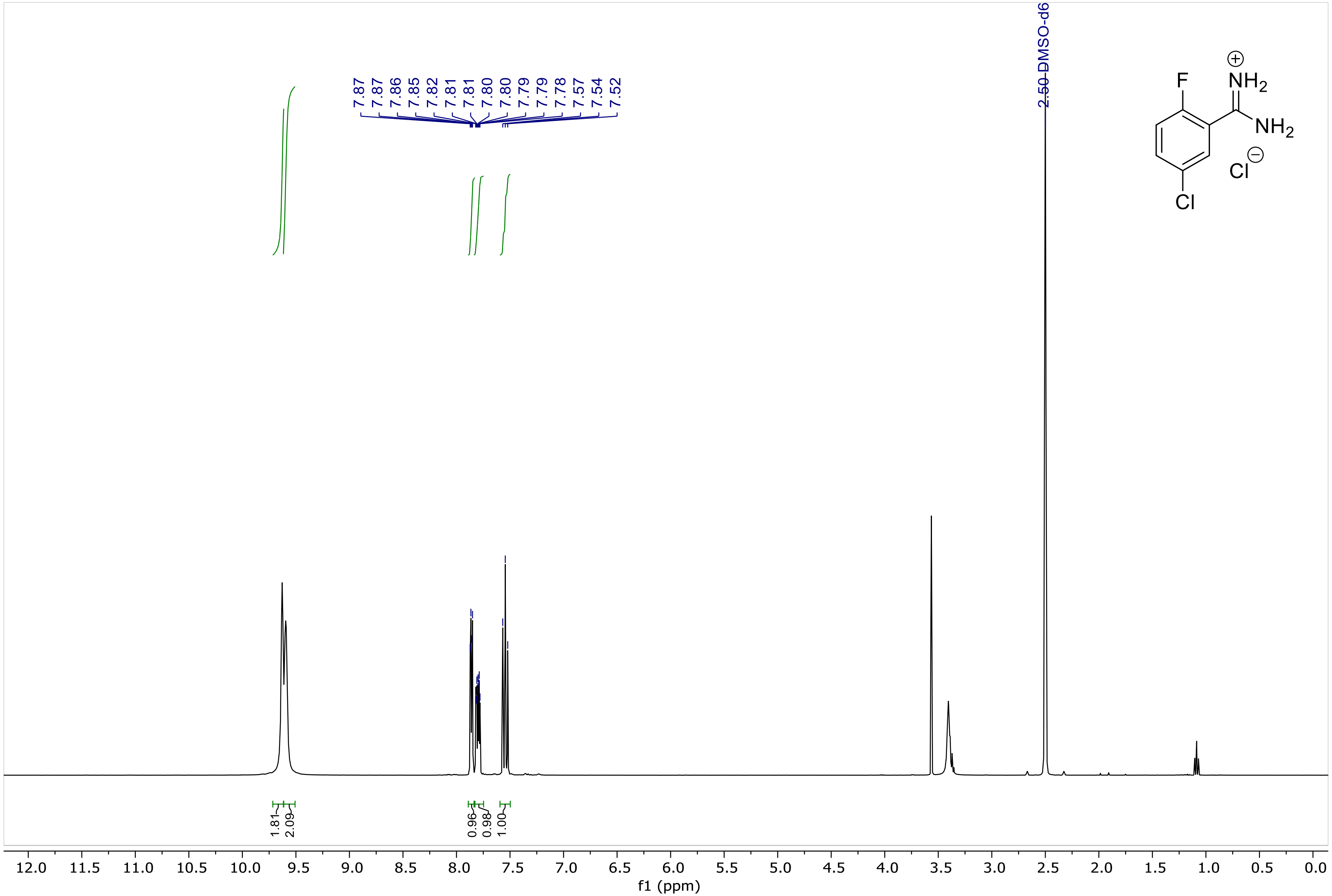

2-Fluoro-5-chlorobenzamidine hydrochloride – $^{13}C\{^{1}H\}$ NMR (101 MHz, DMSO-d_6)

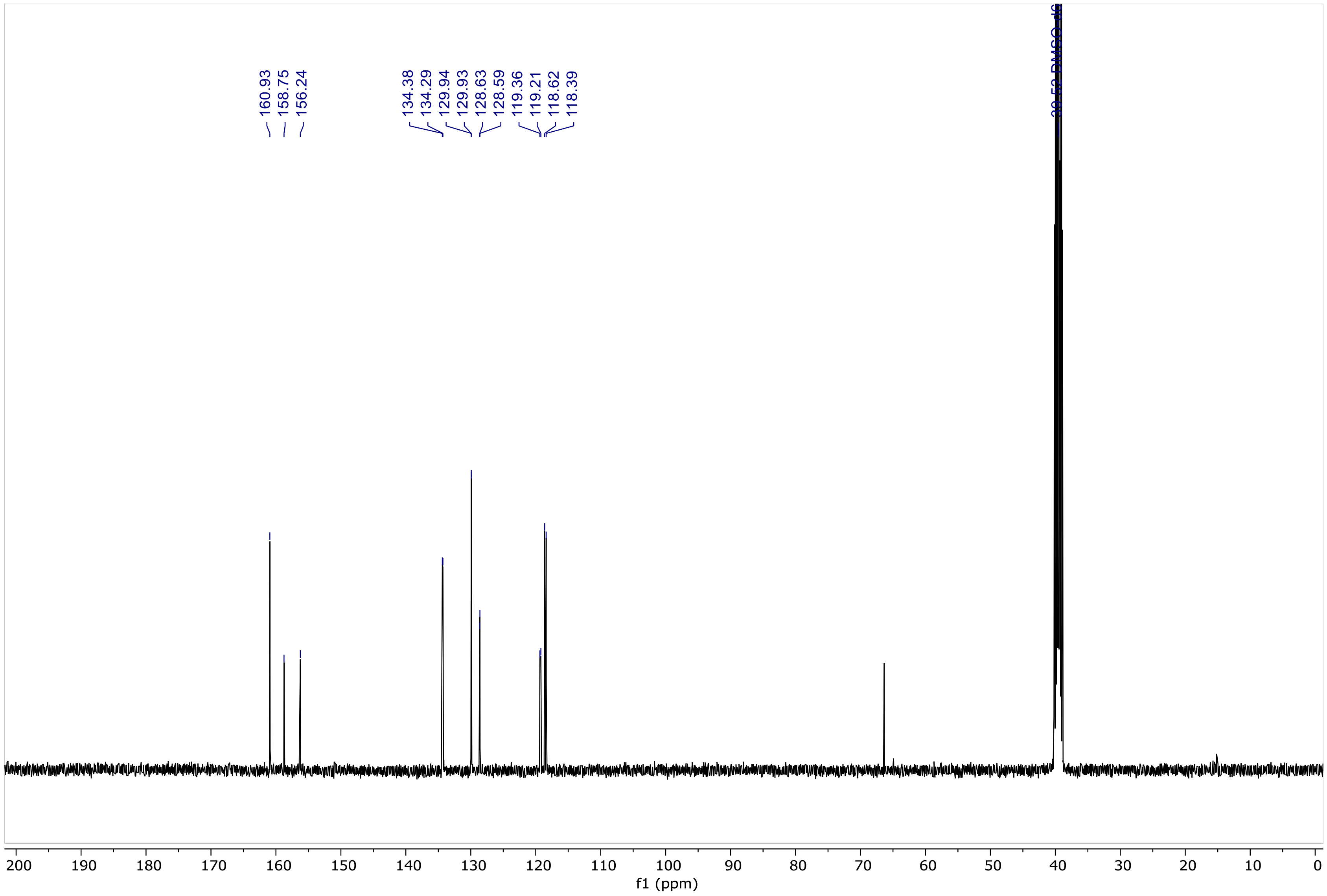

2-Fluoro-5-chlorobenzamidine hydrochloride – ^{19}F NMR (376 MHz, DMSO-d_6)

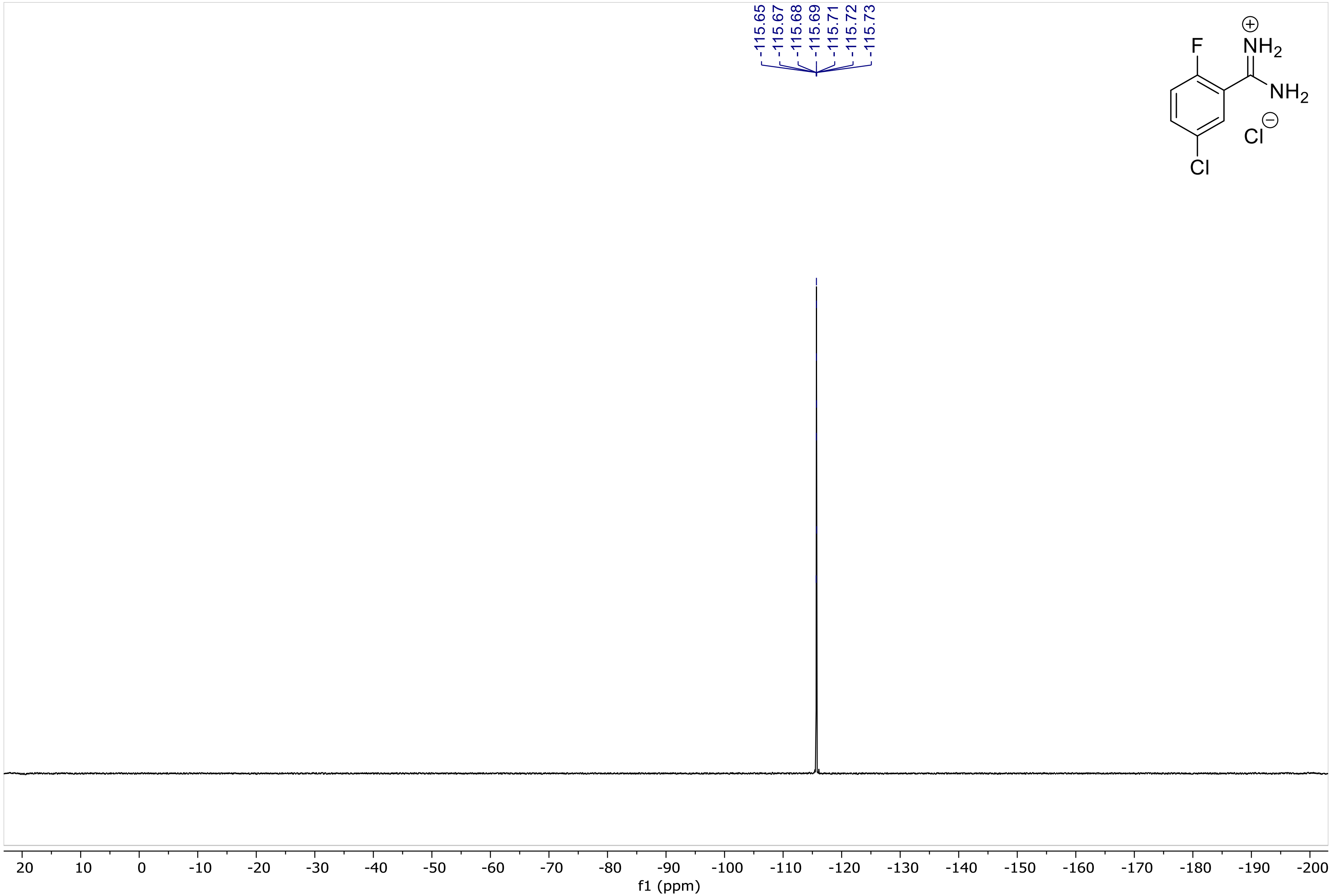

3-Phenylquinolinium hydrochloride – ^{1}H NMR (400 MHz, DMSO-d_6)

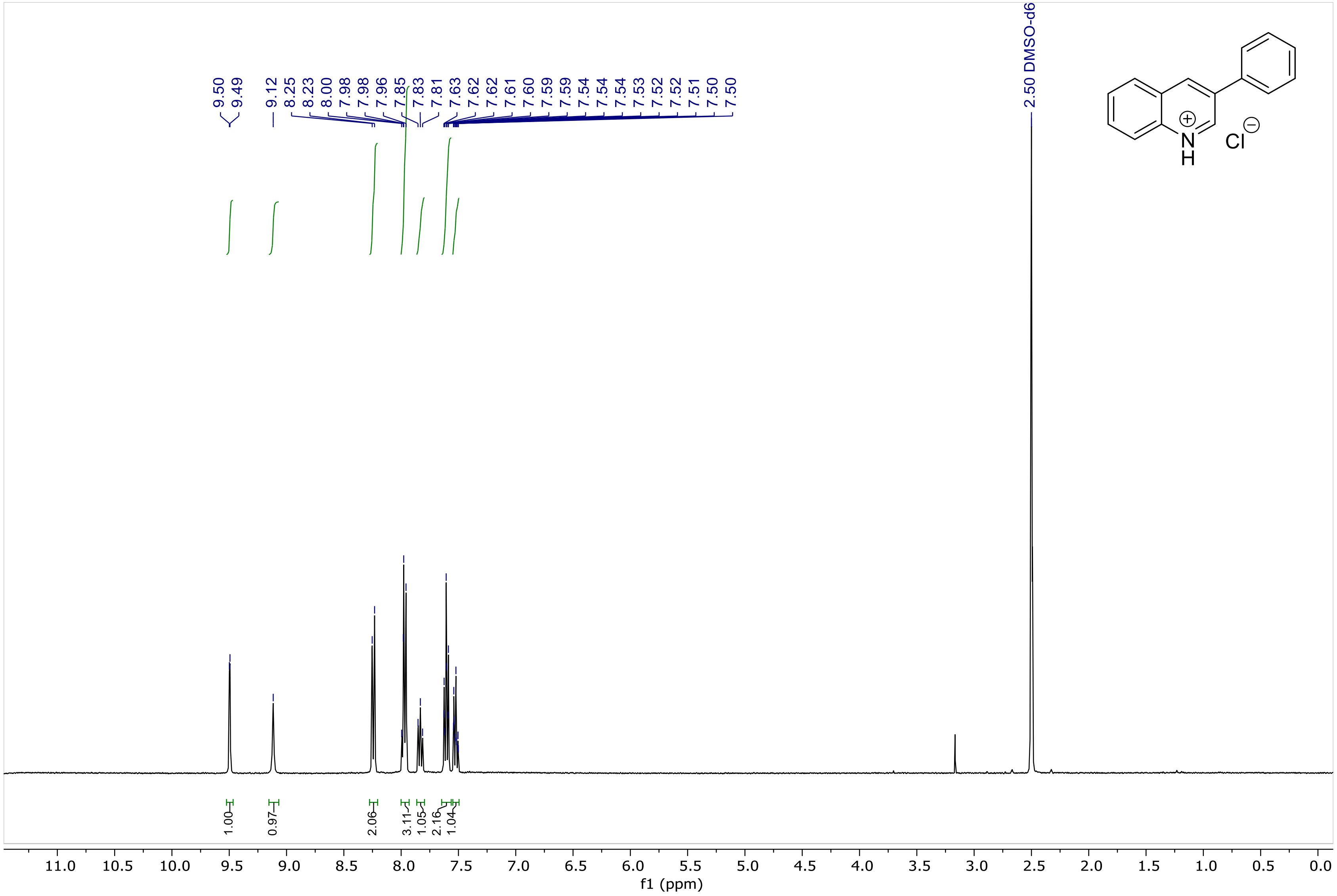

3-Phenylquinolinium hydrochloride – $^{13}C\{^1H\}$ NMR (101 MHz, DMSO-d_6)

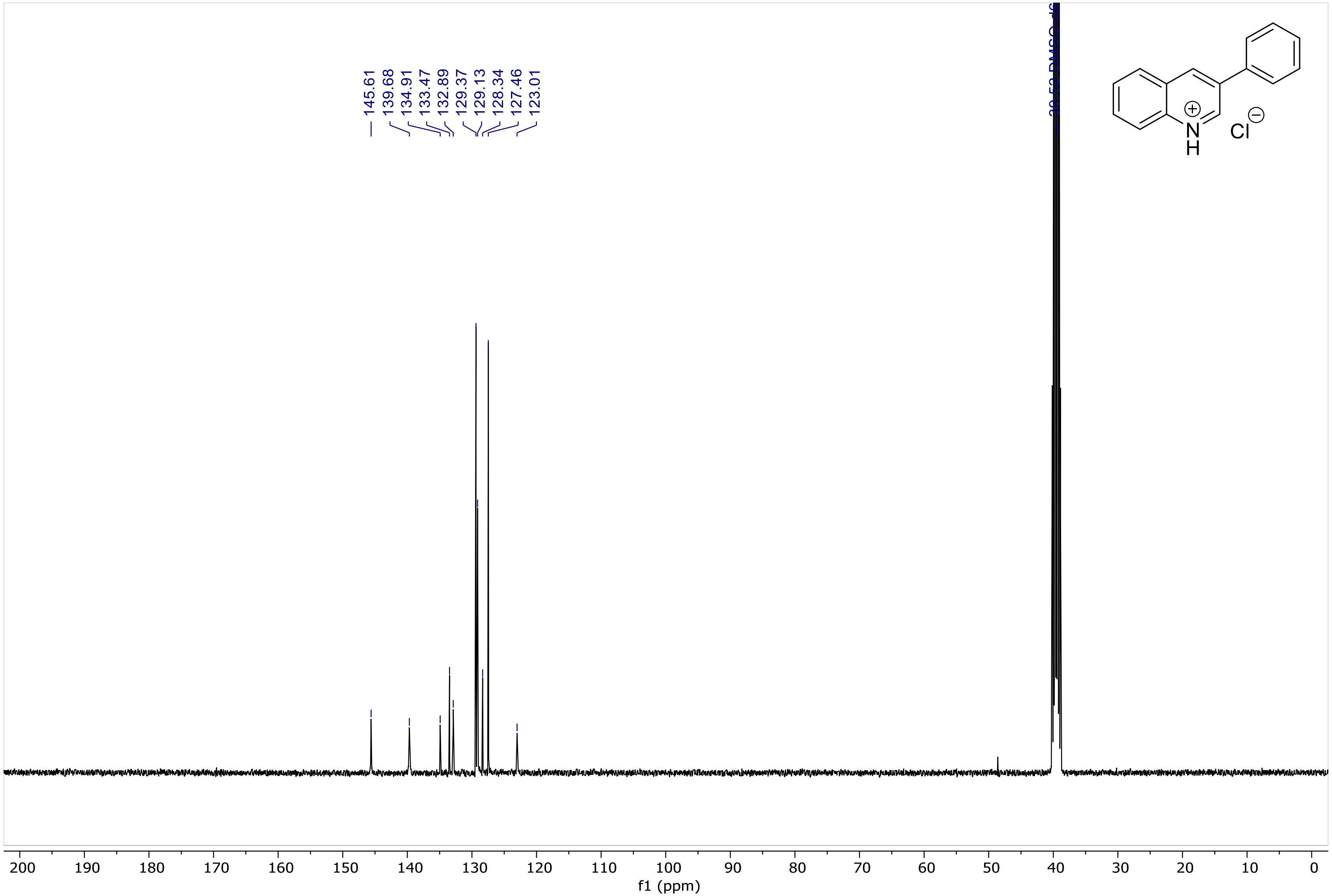

1-Benzyl-3-phenylquinolin-2(1H)-one – ^{1}H NMR (400 MHz, $CDCl_3$)

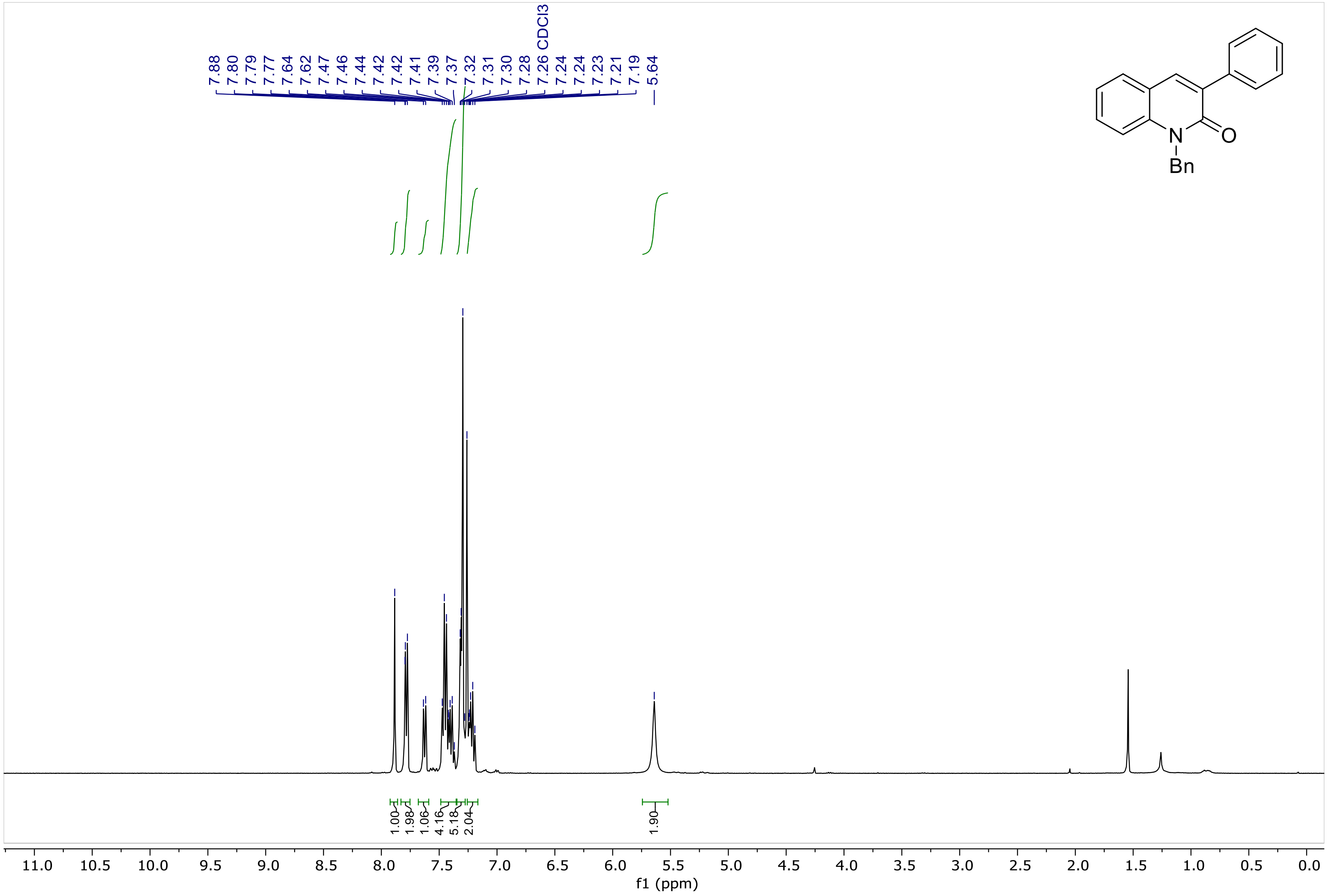

1-Benzyl-3-phenylquinolin-2(1*H*)-one – $^{13}C\{^1H\}$ NMR (101 MHz, $CDCl_3$)

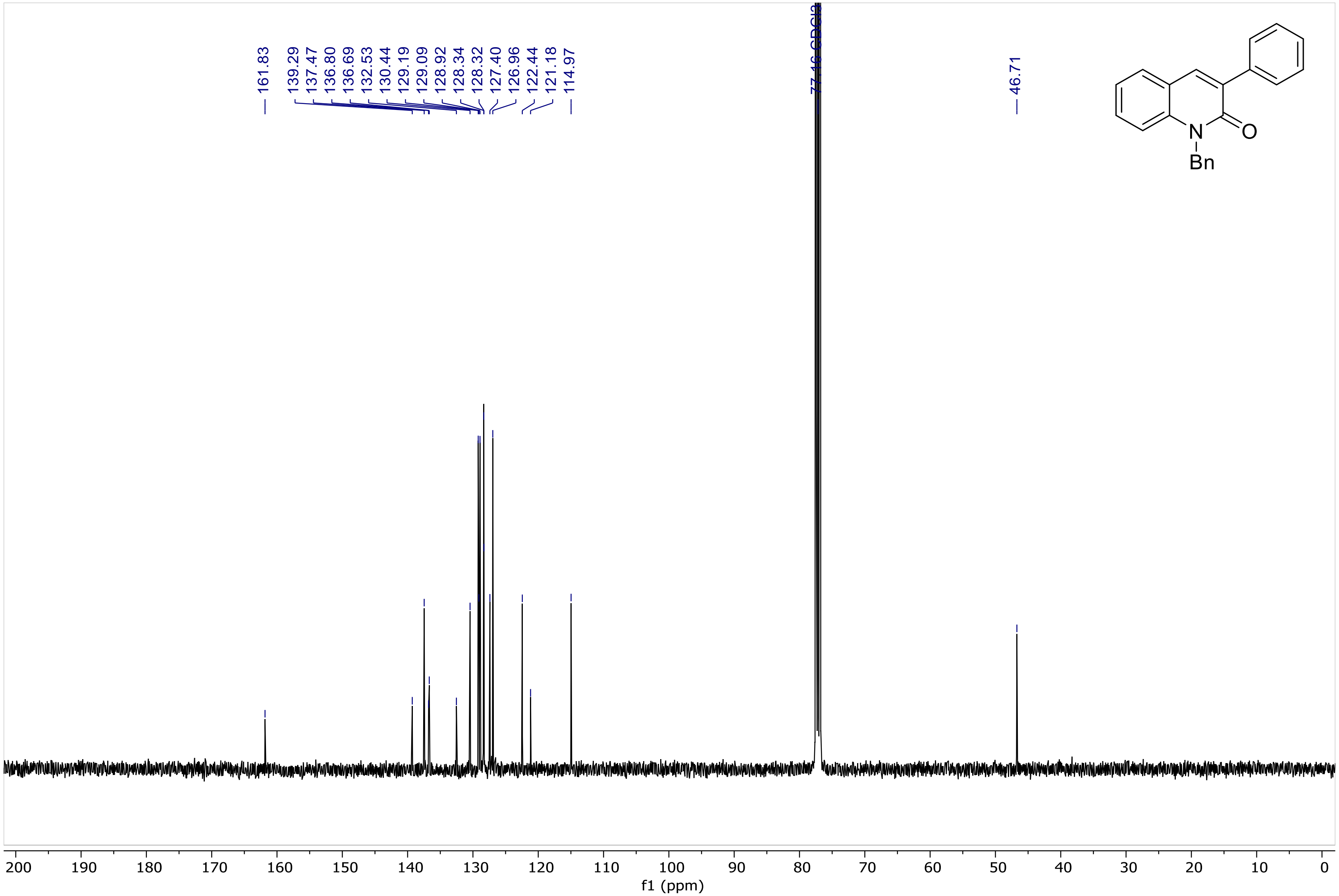

1-benzyl-3-phenyl-1,2-dihydroquinoline – ^{1}H NMR (400 MHz, $CDCl_3$)

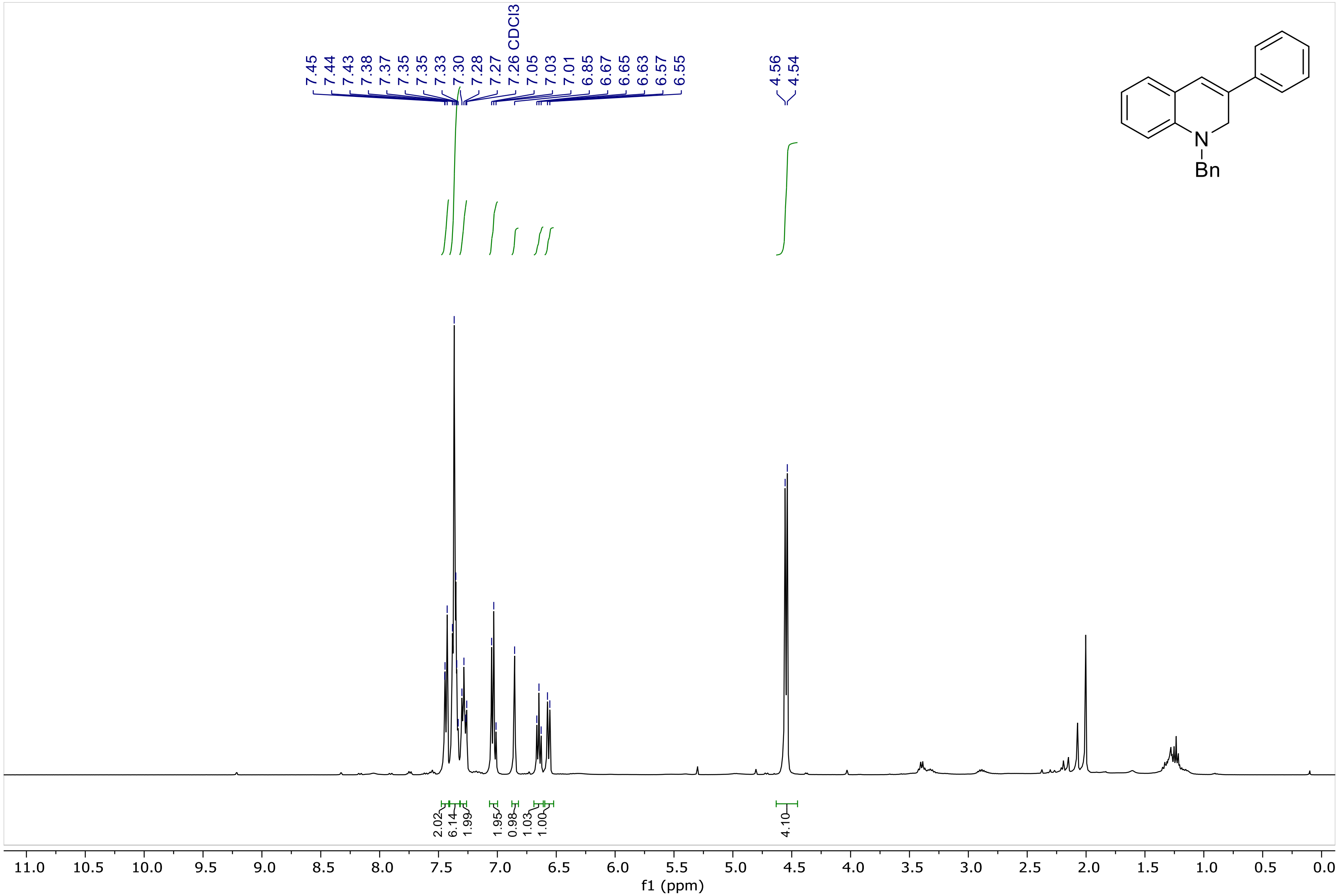

1-benzyl-3-phenyl-1,2-dihydroquinoline – $^{13}C\{^{1}H\}$ NMR (101 MHz, $CDCl_3$)

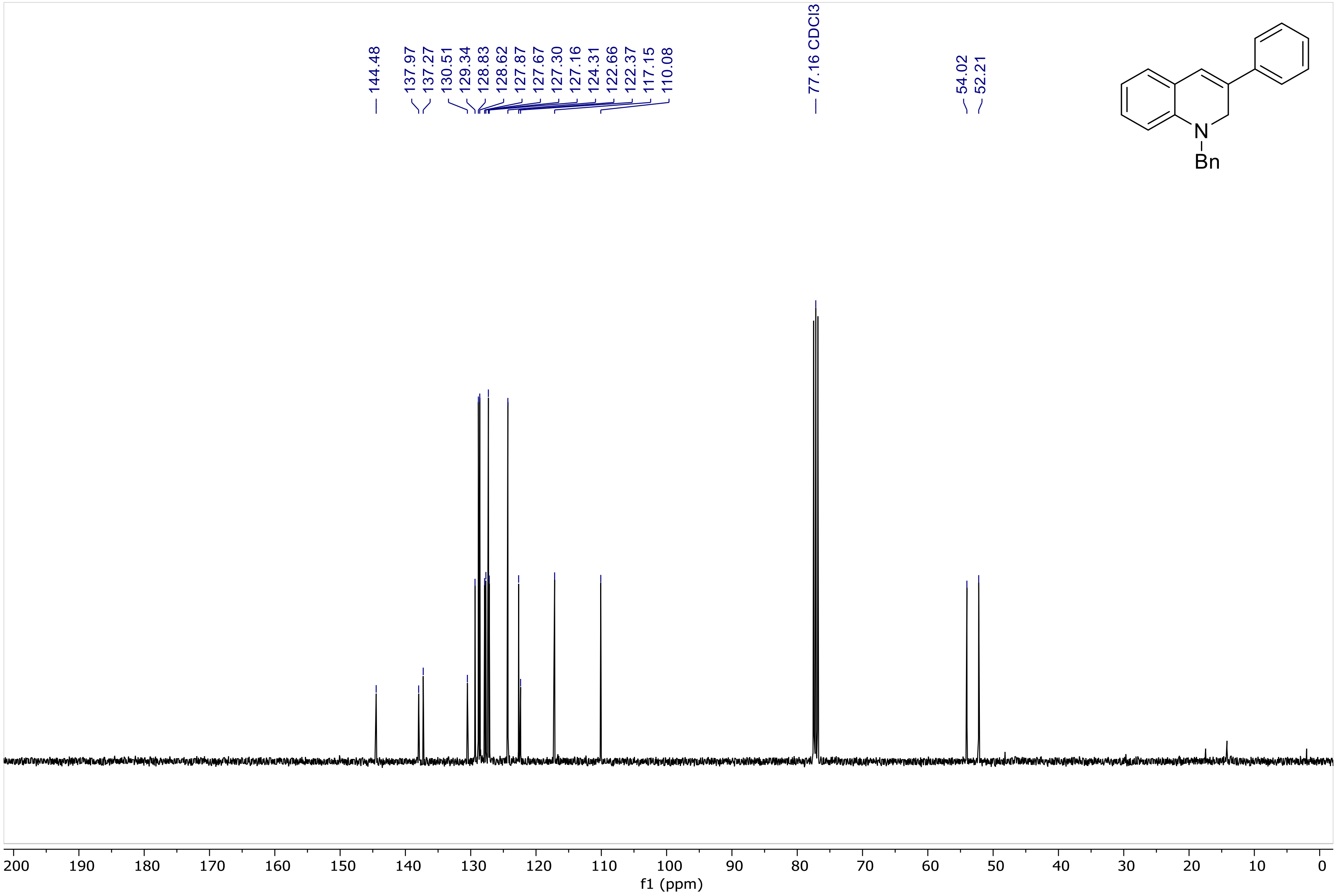

6-Fluoro-3-phenyl-1,2,3,4-tetrahydroquinoline – ^{1}H NMR (400 MHz, $CDCl_3$)

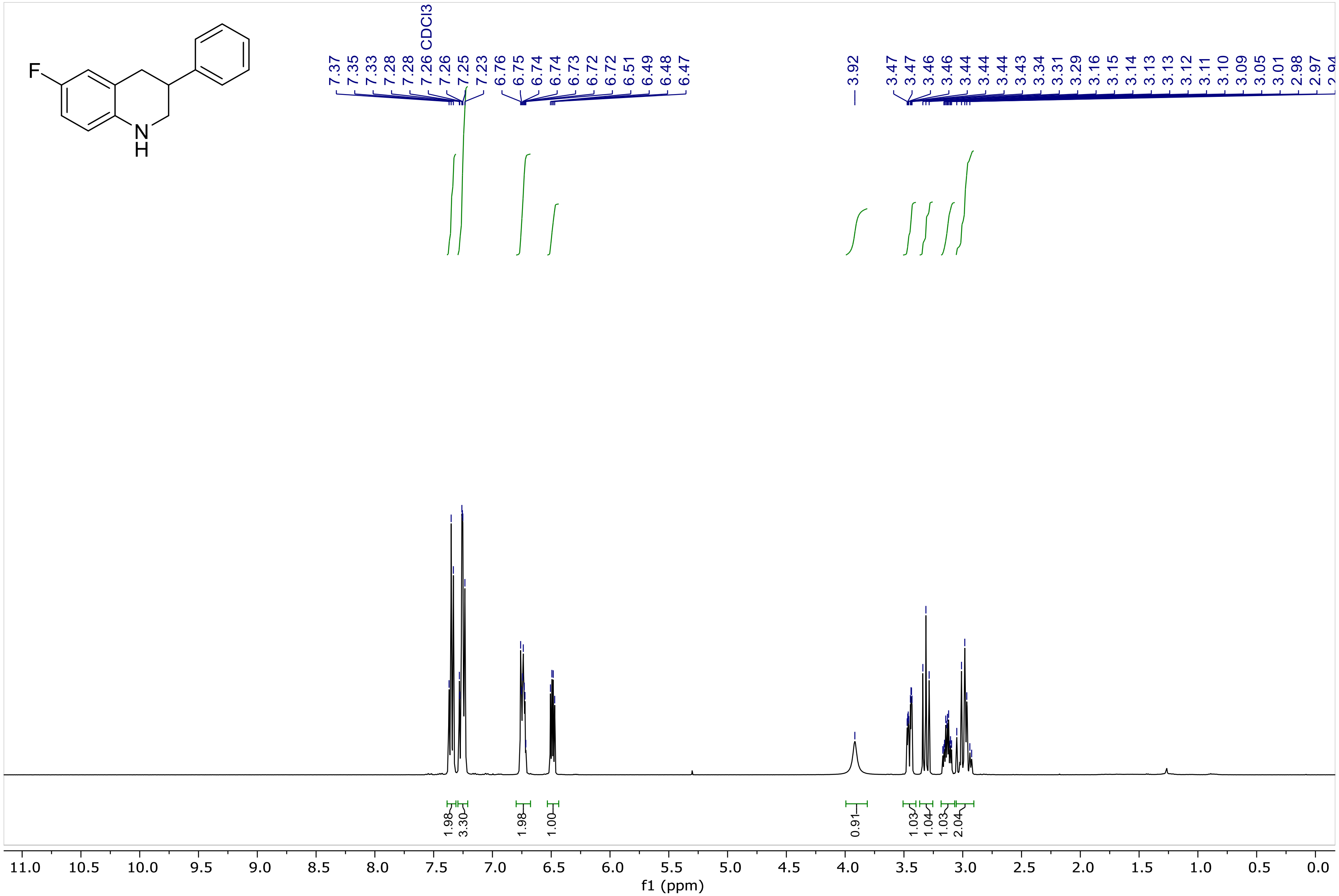

6-Fluoro-3-phenyl-1,2,3,4-tetrahydroquinoline – $^{13}C\{^1H\}$ NMR (101 MHz, $CDCl_3$)

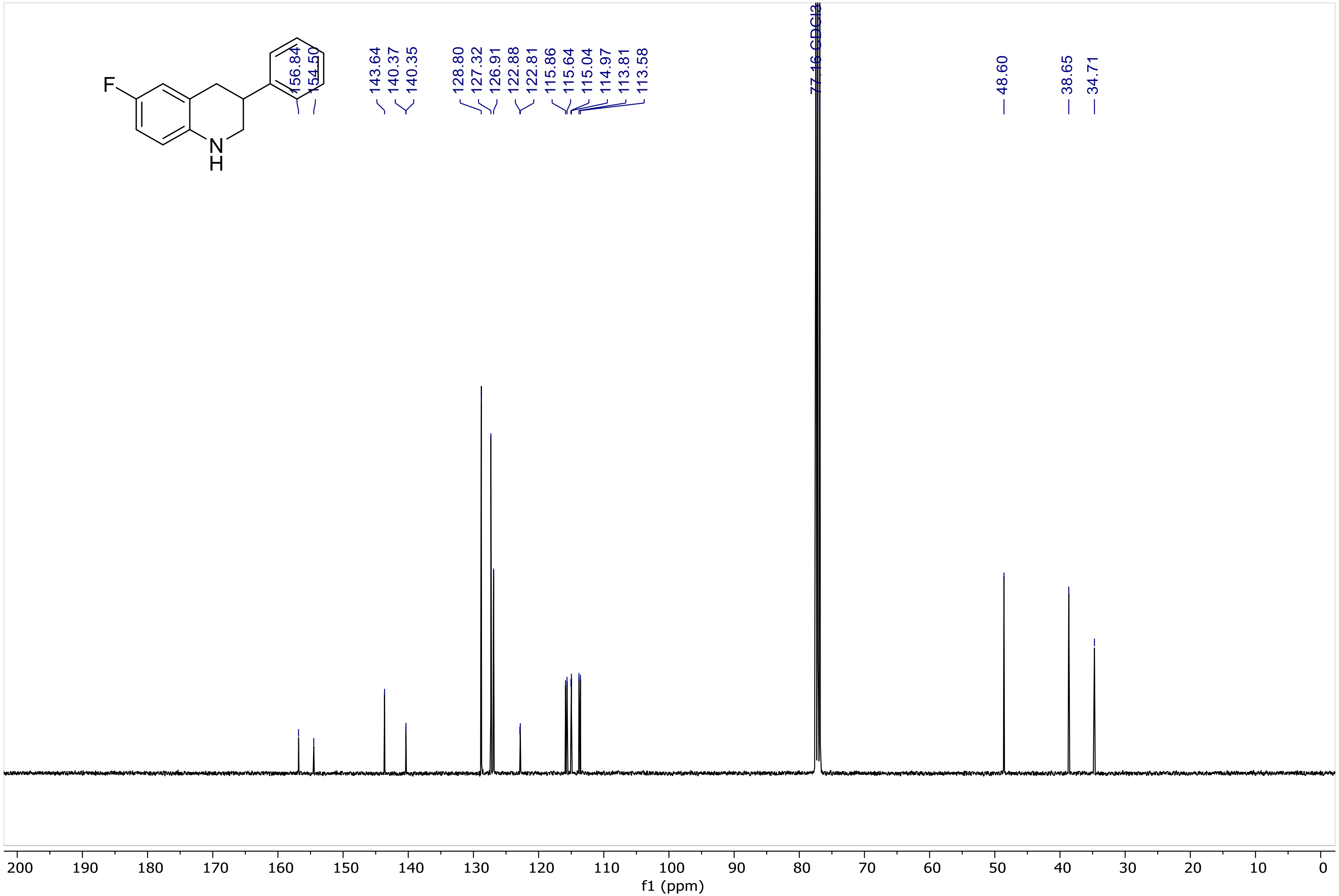

6-Fluoro-3-phenyl-1,2,3,4-tetrahydroquinoline – ^{19}F NMR (376 MHz, $CDCl_3$)

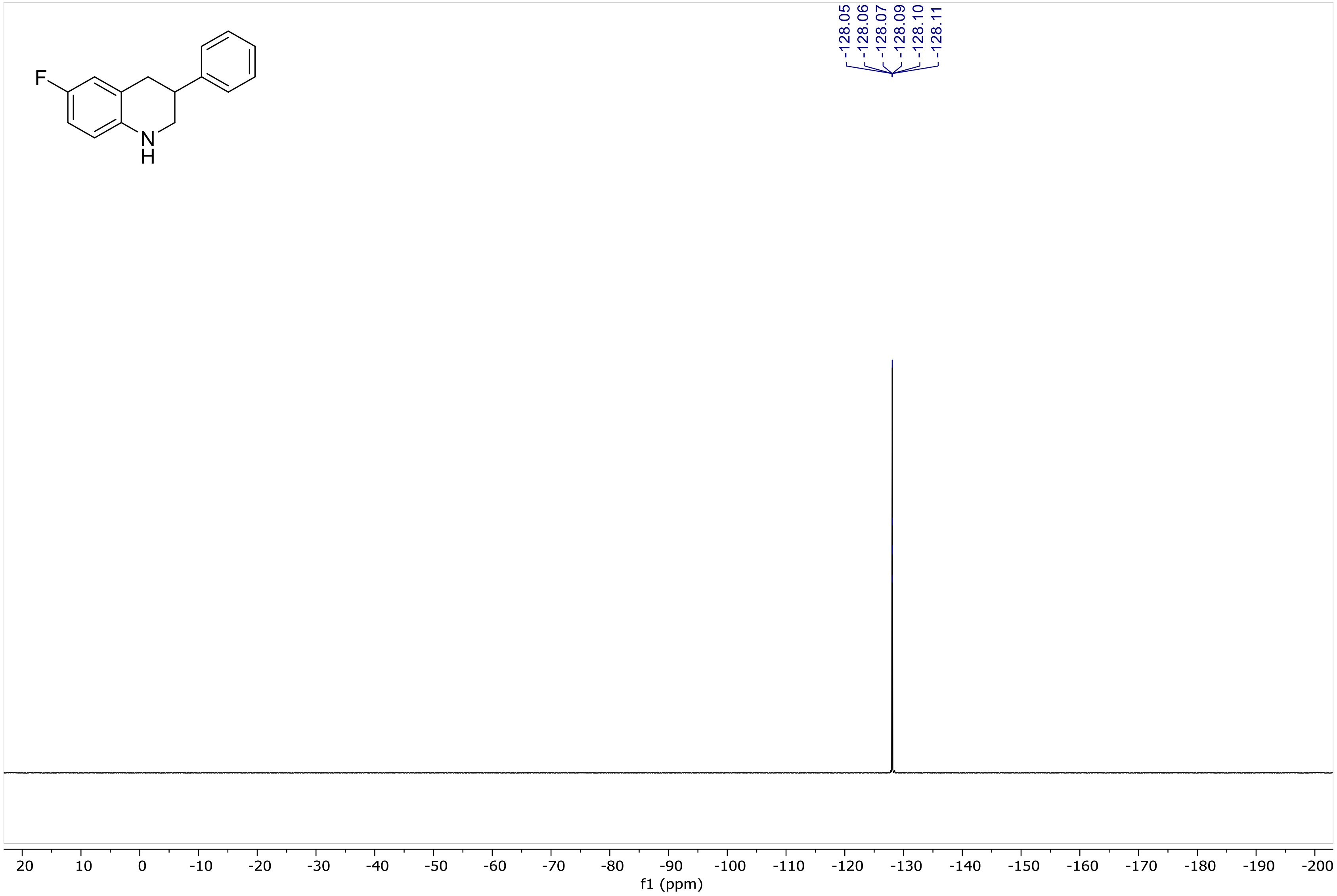

1-Benzyl-3-phenyl-1,2,3,4-tetrahydroquinoline – ^{1}H NMR (400 MHz, DMSO-d_6)

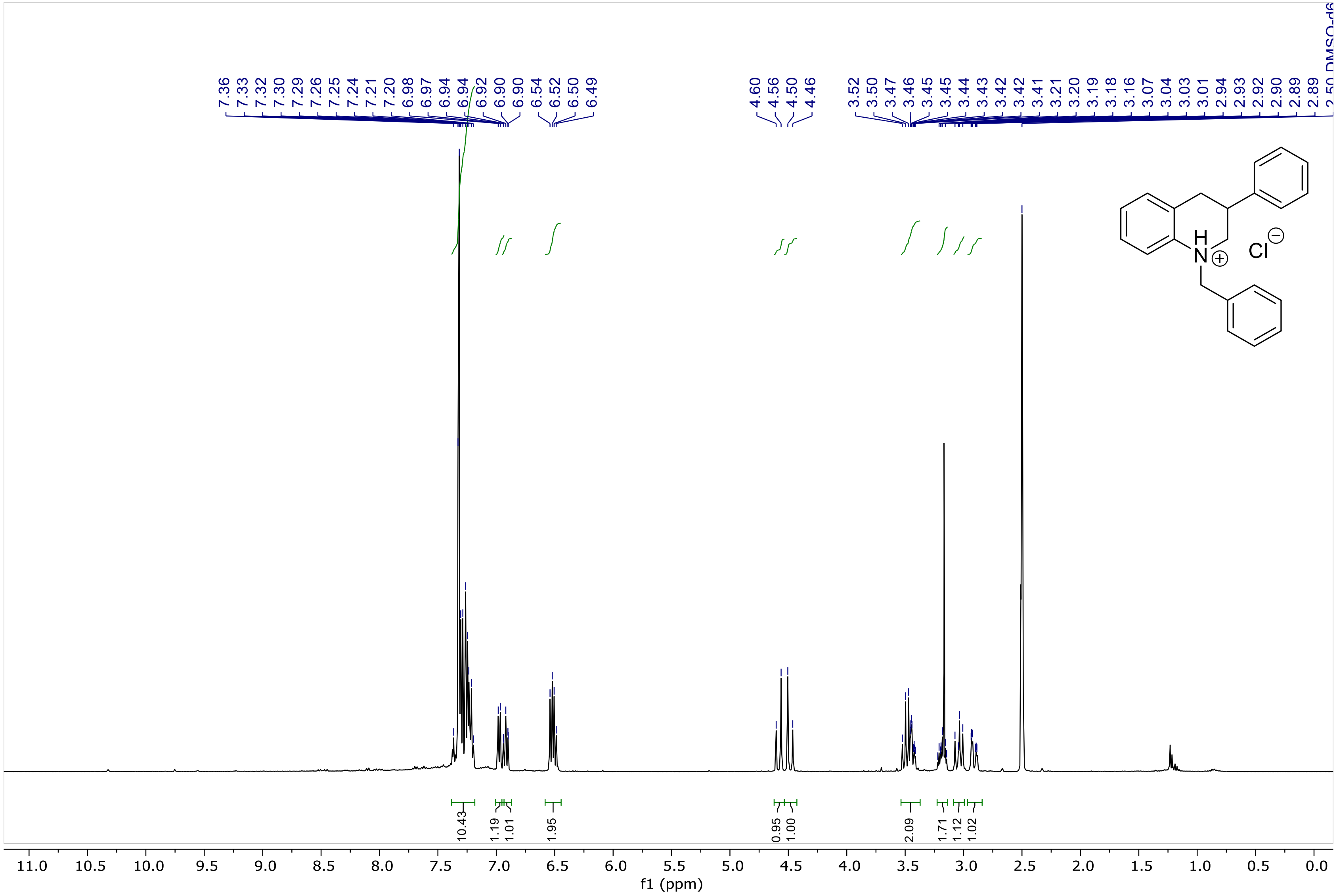

1-Benzyl-3-phenyl-1,2,3,4-tetrahydroquinoline – $^{13}C\{^{1}H\}$ NMR (101 MHz, DMSO-d_6)

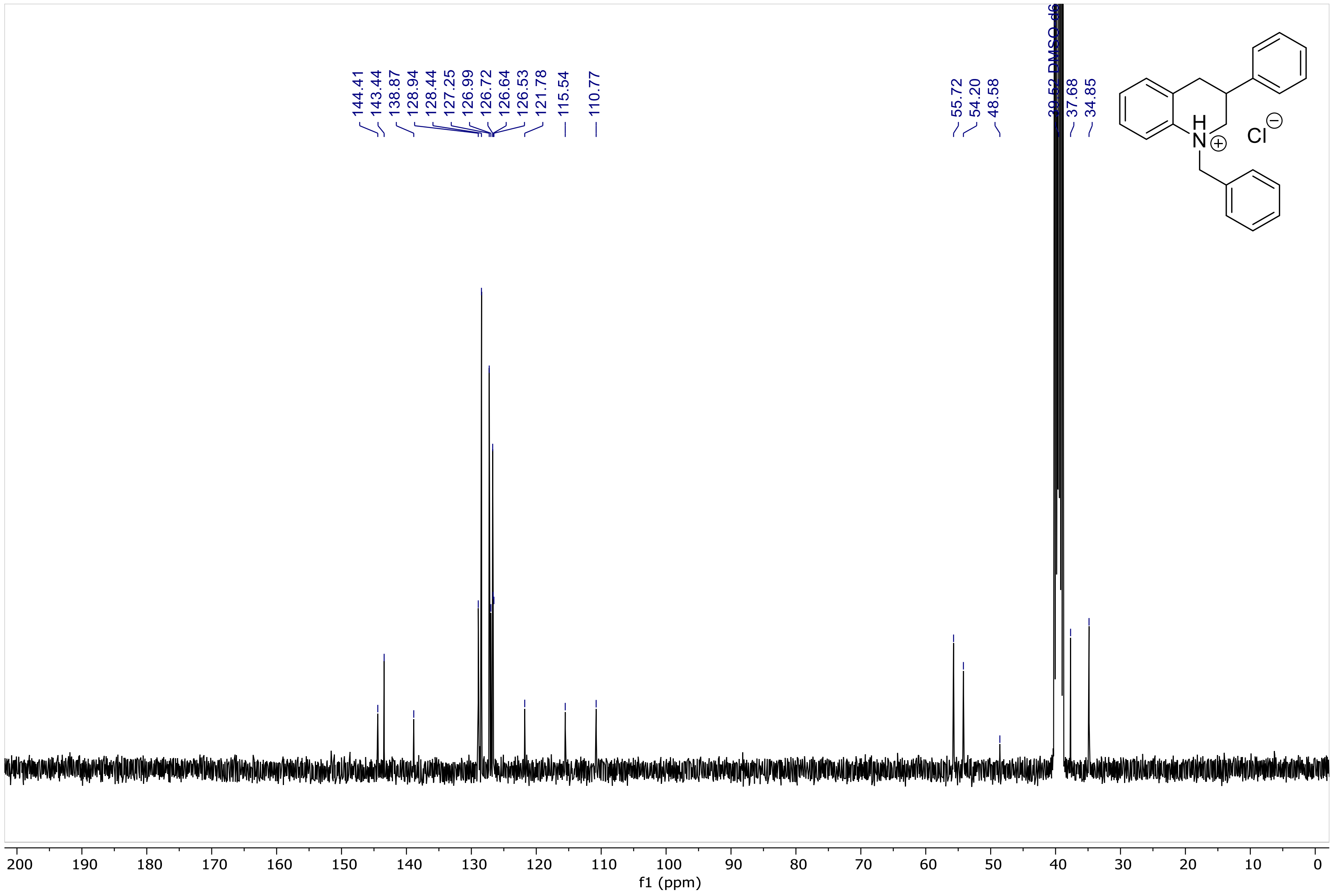

3-Cyclohexyl-1-(cyclohexylmethyl)-5,6,7,8-tetrahydroquinolin-1-ium trifluoroacetate – ^{1}H NMR (400 MHz, DMSO-d_6)

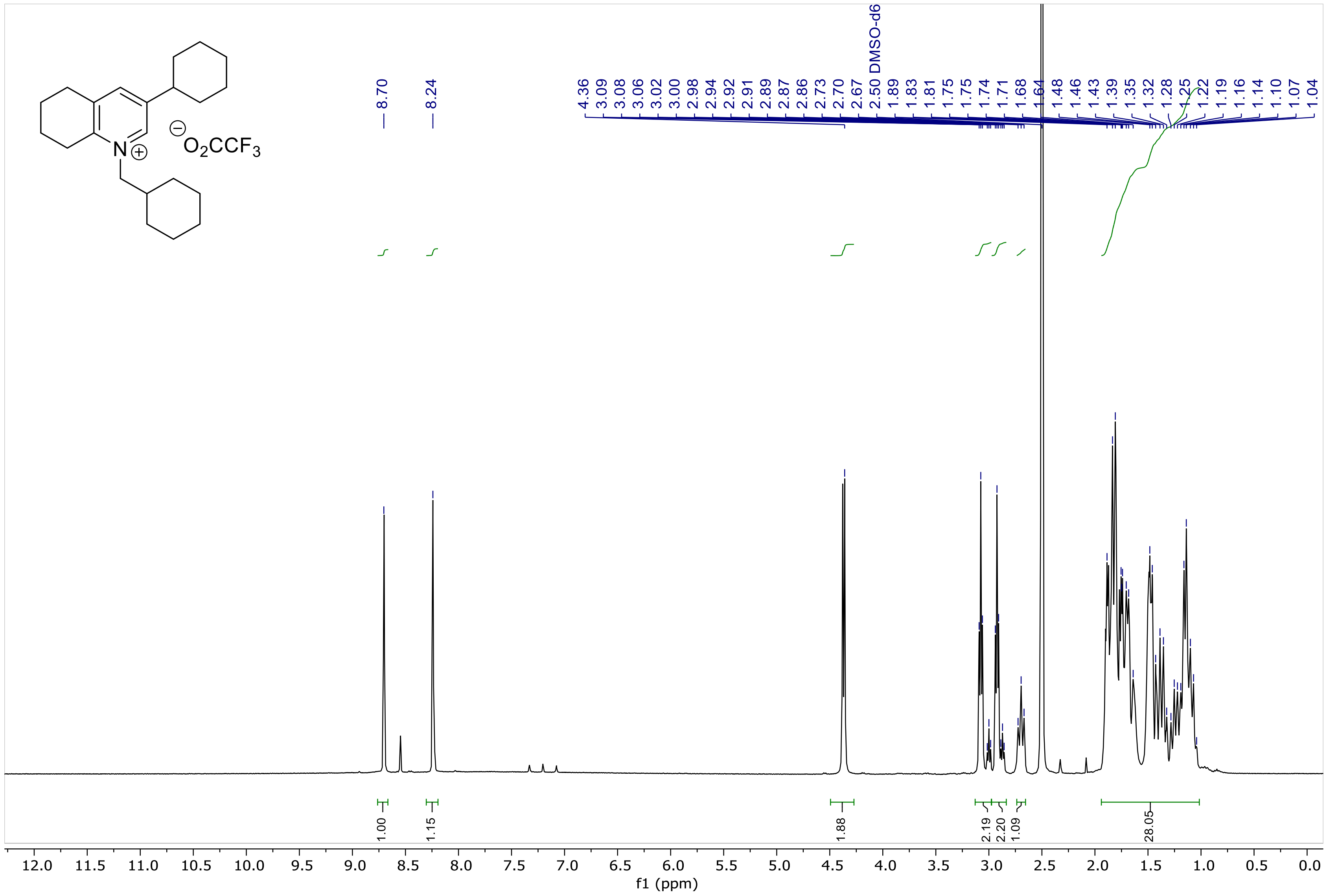

3-Cyclohexyl-1-(cyclohexylmethyl)-5,6,7,8-tetrahydroquinolin-1-ium trifluoroacetate – $^{13}C\{^1H\}$ NMR (101 MHz, DMSO-d_6)

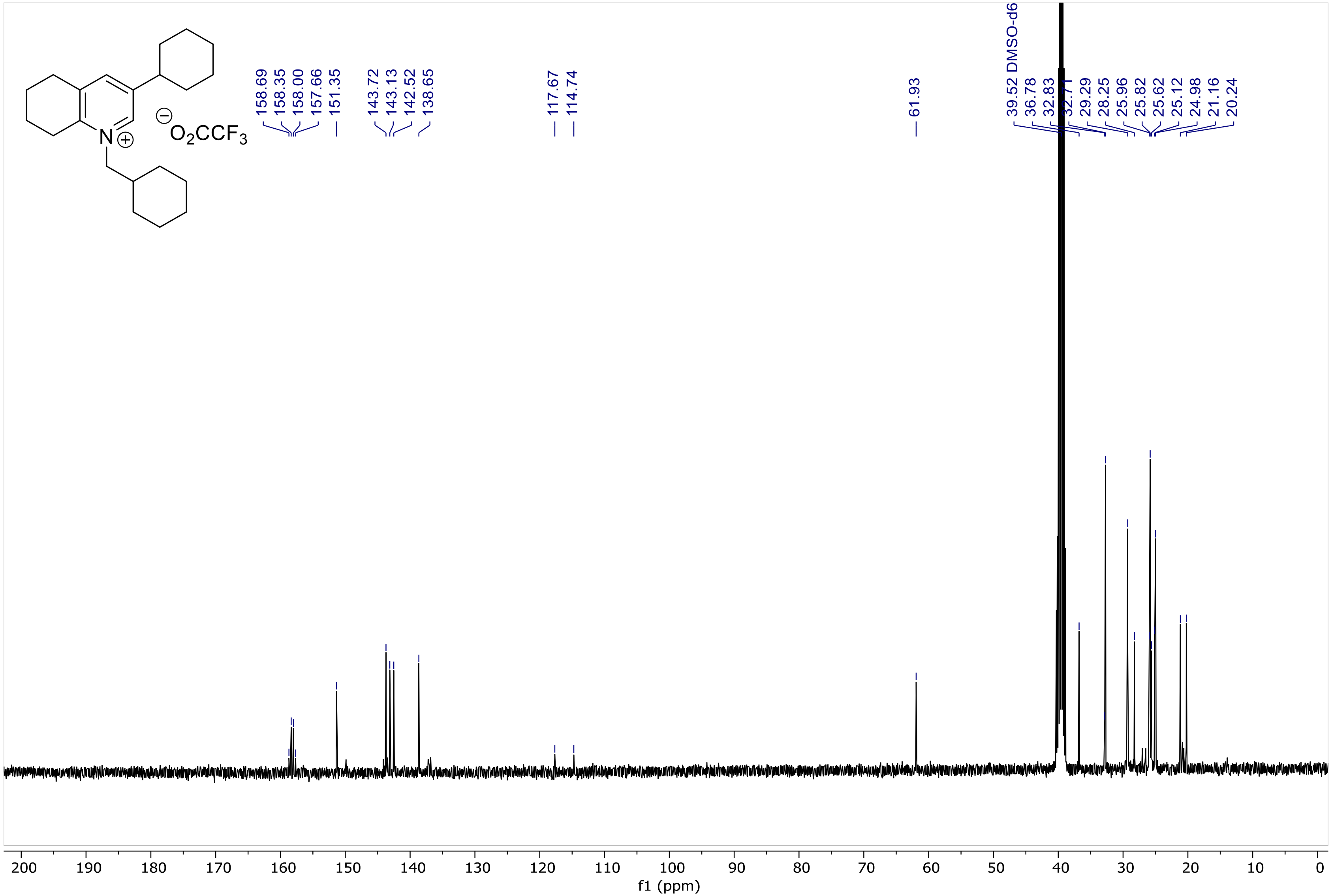

3-Cyclohexyl-1-(cyclohexylmethyl)-5,6,7,8-tetrahydroquinolin-1-ium trifluoroacetate – $^{13}C\{^{1}H\}$ NMR (101 MHz, DMSO-d_6)

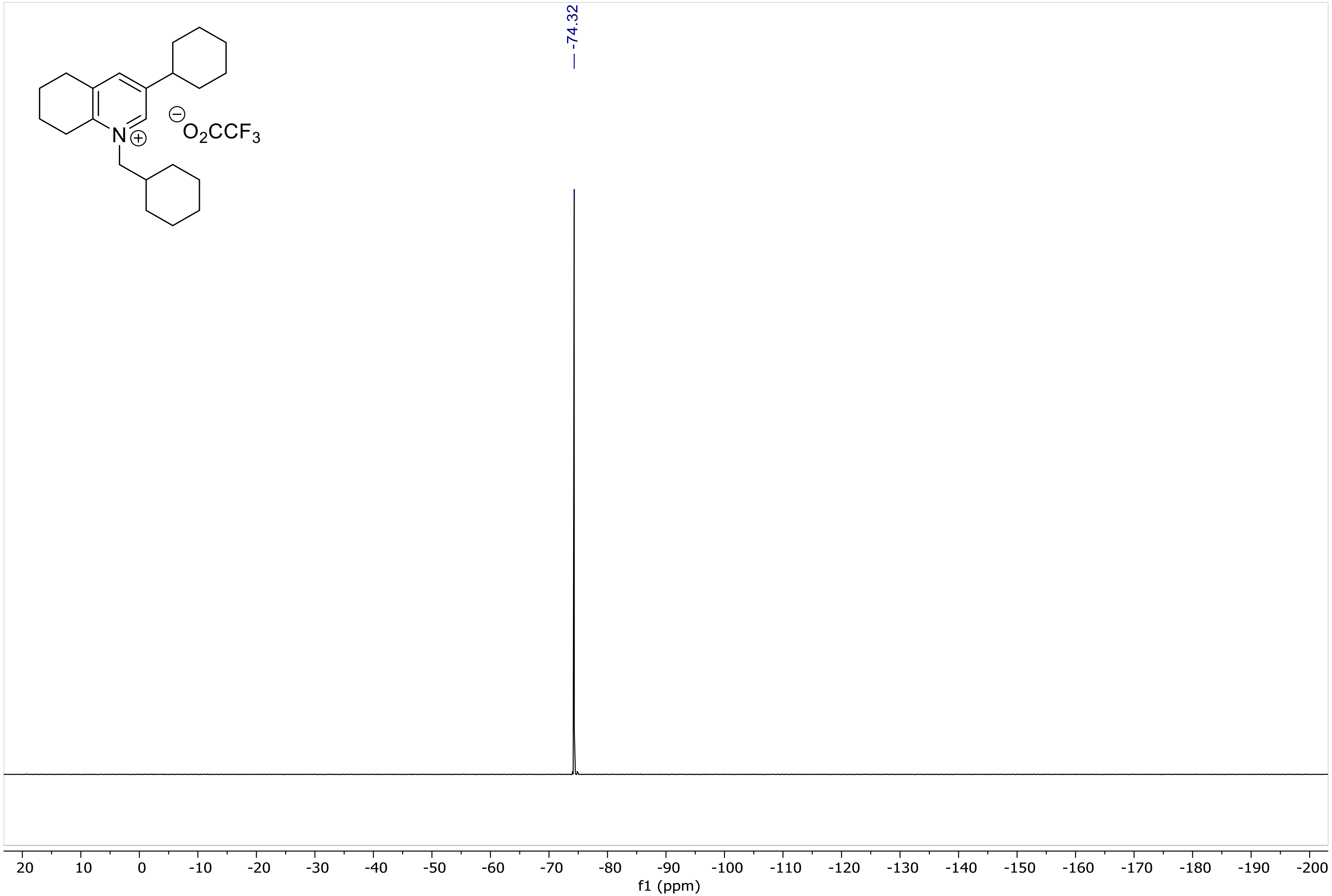

Supplement: Supplementary file 1 — Supporting Information [file ANIE-62-e202305081-s001.pdf]
